# Phosphonium Ions as Activating Groups for the Selective Alkylation of Pyridines and Polyazines

**DOI:** 10.1021/jacs.6c08076

**Published:** 2026-06-05

**Authors:** Dane A. Brunner, David C. Thomas, Kaila R. Steenback, Soren D. Rozema, Andrew McNally

**Affiliations:** Department of Chemistry, 3447 Colorado State University, Fort Collins, Colorado 80523, United States

## Abstract

The Minisci reaction is a broadly applicable method for coupling carbon-bearing groups to pyridines but suffers from poor regio- and site-selectivity, particularly in complex structures. Here, we show that C4-phosphonium salts serve as activating groups in Minisci alkylation processes. In this way, radicals add selectively to pyridines bearing phosphonium salts rather than to those without phosphonium salts in polypyridine structures. Furthermore, the phosphonium salt serves as a blocking group, ensuring regioselective alkylation at the 2-position. The C–P bond is easily cleavable in a simple workup procedure or can be used as a versatile functional handle to construct 2,4-disubstituted pyridines. We also show that site-selective switching reactions are possible by controlling the site of C–P bond formation in drug-like structures.

## Graphical abstract

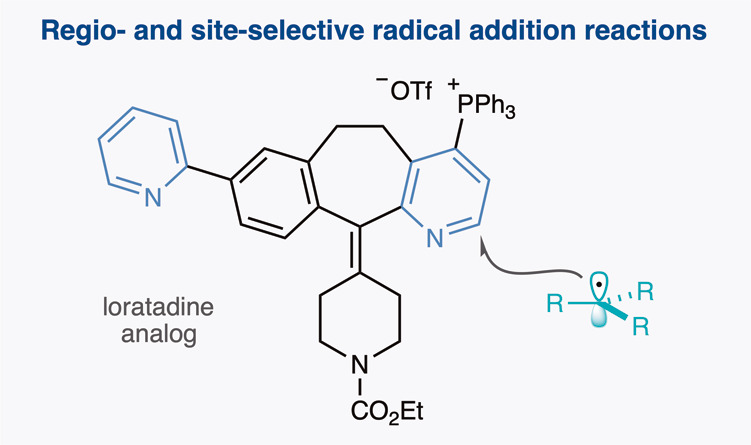

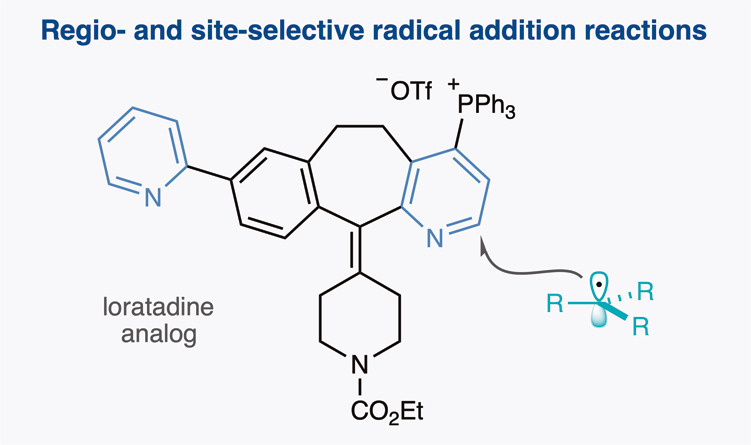

## Introduction

The Minisci reaction is among the most prominent methods to functionalize pyridine C–H bonds and is widely used to append carbon-bearing groups via radical intermediates.^1–4^ This method broadly applies to substituted pyridines, including complex structures found in drug and agrochemical programs.^5–7^ Advances in photochemical and electrochemical methods to generate radicals have further broadened its applicability, as have innovations in reagent design.^3,4,8,9^ As alkyl substitution can profoundly influence a candidate’s pharmacokinetic and pharmacodynamic profile, precise control of regioselectivity is crucial in drug campaigns (Scheme 1A).^10,11^ In that regard, a significant drawback of the Minisci reaction is that it often produces mixtures of regioisomers, particularly in complex substrates. Furthermore, controlling both regio- and site-selectivity when structures contain multiple azines is an unmet challenge.^12–14^ Here, we show that installing a phosphonium ion onto the 4-position of a pyridine serves as an activating group for selective Minisci alkylation on phosphonium-substituted pyridines over unactivated pyridines within the same molecule (Scheme 1B). The phosphonium group also behaves as a blocking group, directing radical additions to the pyridine 2-position. A simple workup cleaves the C–P bond, or the phosphonium ion can be exploited to form 2,4-disubstituted pyridines.

**Scheme 1:**
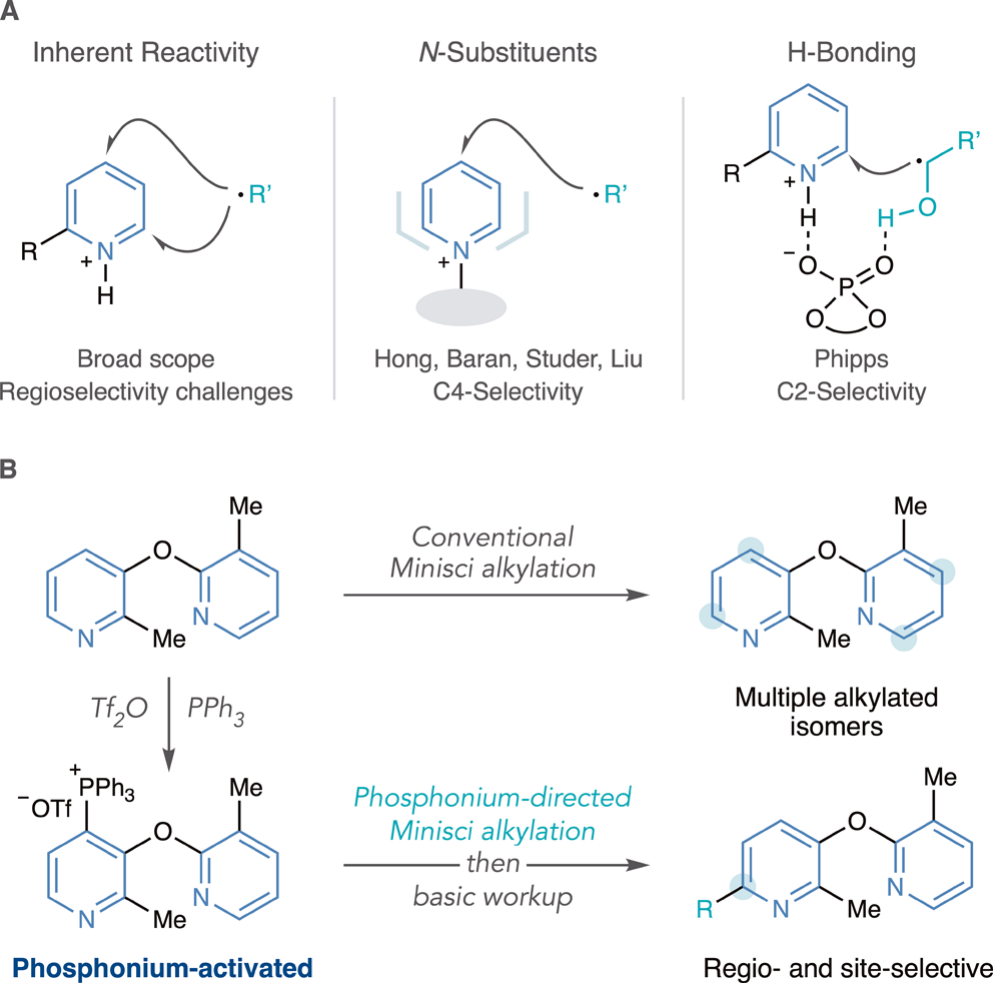
Controlling Minisci Regio- and Site-Selectivity Using Phosphonium Ions[Fn sch1-fn1]

i A. Regiocontrol in the pyridine Minisci alkylation reaction. B. This work: regio- and site-selective Minisci alkylations via phosphonium salt activation.

Recently, two strategies achieved regiocontrol in pyridine Minisci reactions. First, Hong, Baran, Studer, and Liu reported 4-selective processes enabled by *N*-functionalized pyridinium salts.^15–20^ Second, Phipps exploited the H-bonding capacity of alcohols and amides, resulting in 2-selective alkylation.^21–24^ However, to the best of our knowledge, there are no general 2-selective radical alkylation reactions that avoid the requirement for H-bond donors. Additionally, there are no strategies for controlling regio- and site-selectivity in polyazines. We developed a program for pyridine functionalization based on selectively installing phosphonium ions at the 4-position of pyridines.^25–29^ We hypothesized that the bulky phosphonium group would ensure radical addition at the 2-position and that its strong electron-withdrawing properties would increase the electrophilicity of the appended pyridine ring. In a previous report, we also showed that C–P bond formation can occur selectively in polyazines and is switchable between different pyridine rings. We envisioned that these combined properties would enable selective Minisci alkylation in polyazine substrates.

## Results and Discussion

We began our pyridine alkylation studies by selecting the silane-mediated protocol reported by Hong that employs alkyl bromides as radical precursors.^15,30,31^ Treating pyridylphosphonium salt **1a** with (TMS)_3_SiH, cyclohexyl bromide, TFA, and *t*BuOOH as a radical initiator resulted in alkylated phosphonium salt **2a** in modest yield (Table 1, entry 1). Notably, **2a** formed as a single regioisomer based on the crude ^1^H NMR spectrum. We found that switching from 455 nm light to broad-spectrum white LEDs significantly improved the yield (entry 2). Solvents other than MeCN either decreased the yield of **2a** or resulted in no product formation (entries 3–5). Using benzoyl peroxide instead of *t*BuOOH also negatively impacted the reaction, and only small amounts of product formed when we omitted the peroxide (entries 6 and 7). Diluting the reaction to 0.05 M in MeCN modestly increased the yield (entry 8). Excluding TFA from the reaction did not significantly affect the outcome, indicating that the phosphonium has a significant activating effect; therefore, we removed acid from the protocol (entry 9). Adding TFE as a cosolvent, however, gave a near-quantitative yield of **2a** (entry 10). We observed the same outcome when using a *t*BuOOH solution in decane, and we isolated the product on a 0.5 mmol scale (entry 11).

**Table 1:**
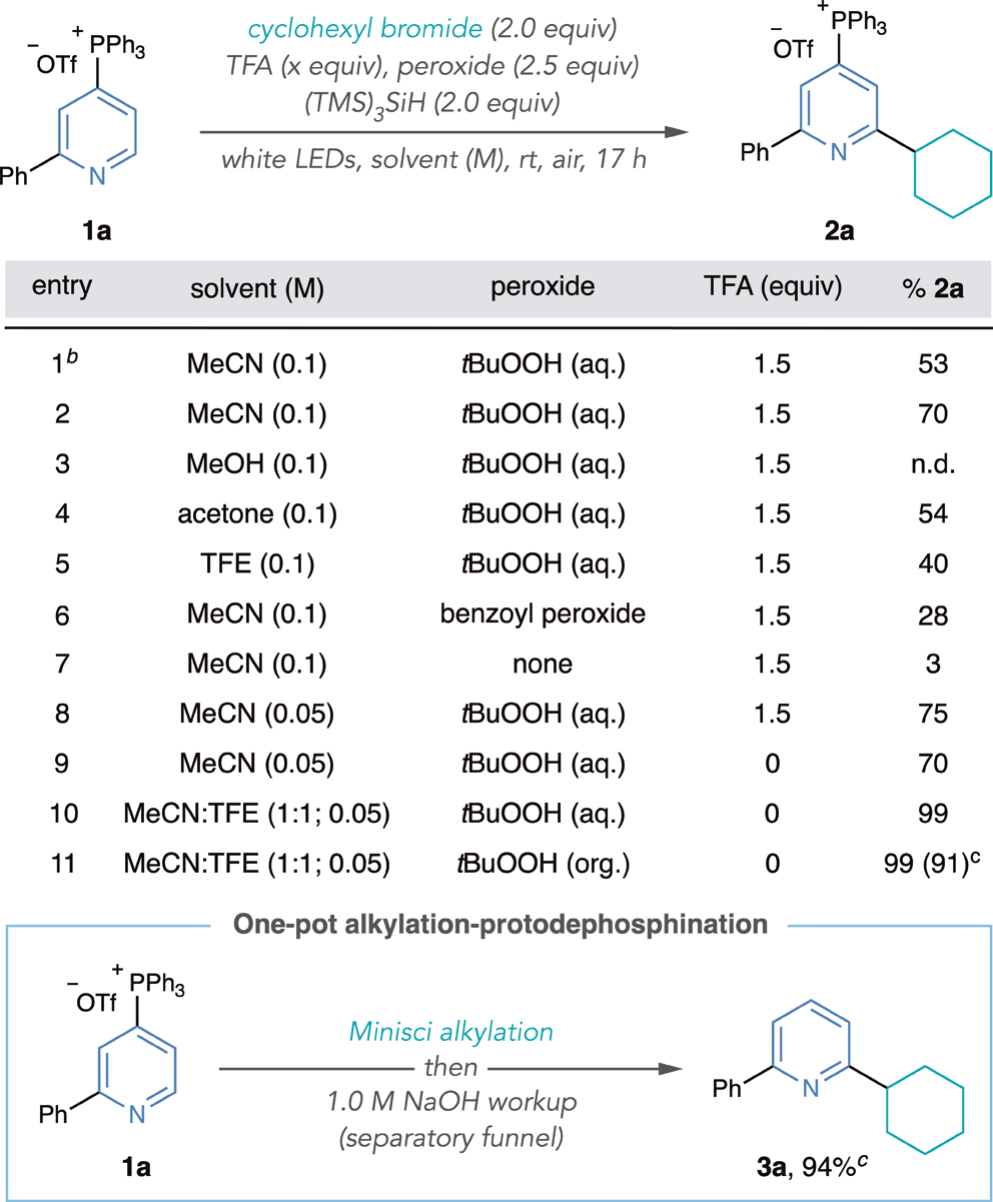
Pyridylphosphonium Alkylation Optimization Study^a^

a Yields of 0.1 mmol scale reactions calculated by ^1^H NMR spectroscopy using Ph_3_CH as an internal standard.

b 455 nm Kessil light used instead of white LEDs.

c Isolated yield on 0.5 mmol scale.

The bottom of Table 1 also shows a convenient method for cleaving the phosphonium group by performing aqueous NaOH washes in a separatory funnel during the workup, affording alkylated pyridine **3a** in 94% yield. We also performed control reactions on 2-phenylpyridine without phosphonium ion substitution. Without acid, the starting material persists; in the presence of TFA, mixtures of 2- and 4-cyclohexylated isomers form as anticipated (See the Supporting Information Section 1.4).

With the optimized conditions in hand, we next explored the scope of alkyl bromides for the pyridine alkylation process (Table 2). Acyclic secondary bromides are well tolerated (**3b** and **3c**), as are carbocycles of various ring sizes, with reduced yields in the case of cyclopropane (**3d**–**3f**). The reaction tolerates cyclic ketones (**3g**) as well as saturated heterocycles, such as examples **3h** and **3i**, which append tetrahydropyran and piperidine rings, respectively. We isolated the menthol derivative **3j** in modest yield, indicating that carbocyclic radicals with adjacent sterically encumbered groups can function in the protocol. Tertiary alkyl radicals are efficient partners, delivering **3k** and **3l** in excellent yields. Given previous studies on the rates of radical formation involving silyl radicals in XAT processes, we anticipated that coupling primary alkyl radicals would be more challenging.^32^ However, we were encouraged to see that linear alkyl chains as well as those bearing alcohols, esters, nitriles, and olefins were amenable, albeit in lower isolated yields (**3m–3r**). Phosphonate ester **3s** derives from a reaction with a primary benzylic bromide.

**Table 2:**
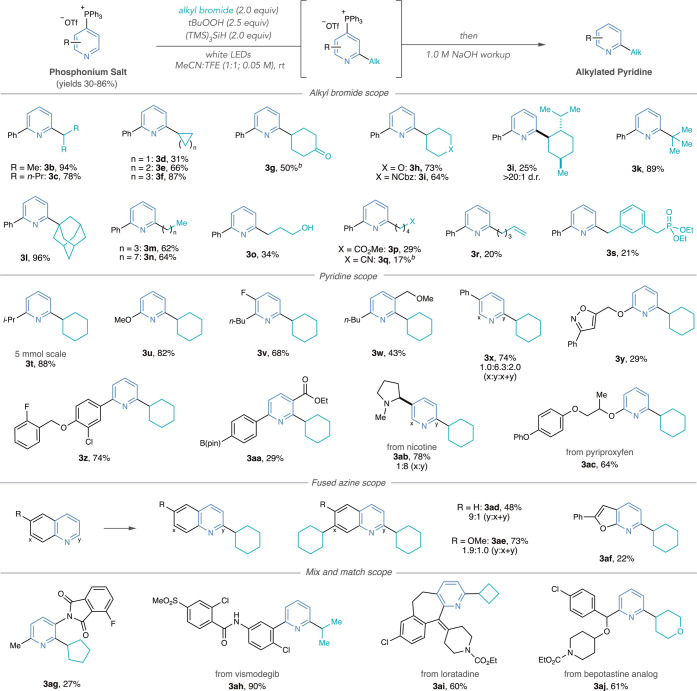
Scope of Alkylbromides and Pyridines in the 2-Selective Minisci Alkylation Reaction^a^

a Isolated yields of products are shown.

b Run using 5 equiv of alkyl bromide and silane.

Next, we investigated the scope of pyridylphosphonium salts using cyclohexyl bromide as the radical precursor (Table 2). The reaction tolerates alkyl groups at the pyridine 2-position, and we isolated **3t** in 88% yield on a 5 mmol scale. Electron-donating substituents, such as methoxy groups, are also compatible (**3u**). We tested 2,3- and 2,5-disubstituted pyridines, resulting in trisubstituted pyridines **3v** and **3w** in reasonable yields. 3-Substituted pyridines are competent substrates, although we observed a mixture of 2-, 6-, and 2,6-disubstituted products (**3x**); this example highlights a current limitation of this approach’s impact on regioselectivity. Alkylated pyridines **3y–3aa** contain isoxazole rings, multiple aryl groups, and BPin substituents. Bioactive compounds nicotine and pyriproxyfen also function in this alkylation protocol (**3ab** and **3ac**). We next examined fused azines. In these cases, quinolines **3ad** and **3ae** afforded mixtures of 2-alkylated and 2,7-dialkylated products, whereas furopyridine **3af** formed a single cyclohexylated isomer. In the final part of Table 2, we varied both the pyridine-containing structure and alkyl bromide. Cyclopentylation of an imide-containing pyridine resulted in **3ag** in low yield. Alkylated pyridines **3ah–3aj** are derived from vismodegib, loratadine, and a bepotastine analog; the single regioisomers obtained demonstrate that this protocol is an effective tool for late-stage alkylation of bioactive molecules.

We next investigated whether phosphonium ions could direct radicals in site-selective alkylations of polyazines. The activating effect of the phosphonium group witnessed in Table 1 in the absence of acid led us to postulate that those pyridines are more electrophilic than their unsubstituted counterparts and should react faster with nucleophilic radicals.^33^ Based on LUMO energies, the activating ability of the phosphonium salt approaches that of pyridine protonation.^34^ Therefore, practitioners could use our previously reported site-selective phosphonium salt-forming protocols for polyazines and direct alkylation to specific pyridine rings.^35^ Predicting the regio- and site-selective outcome when performing Minisci alkylation on polyazines is challenging, and mixtures of isomers can form. As a consequence, chemists are often forced into repetitive synthesis by installing alkyl groups onto a pyridine precursor.

We began our site-selectivity study by testing the Minisci alkylation on bipyridylether **4** as a model system without phosphonium ion substitution (Table 3A). Subjecting **4** to the standard reaction conditions gave predominantly unreacted starting material and 6% of products **5a**–**c**. It is likely that **3ak** forms in the reaction mixture, but it is not possible to report an assay yield via the crude ^1^H NMR spectrum due to peak overlap. Adding one equivalent of TfOH resulted in approximately the same amount of the starting material and 30% of a mixture of alkylated pyridines containing **5a**–**c**, with **3ak** likely present in a proportionate amount. Using two equivalents of acid preserved the starting material and gave no detectable alkylated products. Using TFA instead of TfOH produced mixtures of **3ak**, confirmed by isolation using two equivalents of TFA, and **5a**–**c**. We then prepared phosphonium salt **1ak** in a >20:1 isomeric ratio using our base-mediated selectivity protocol, which favors reactivity on pyridines bearing 3-oxy substituents over those bearing 3-alkyl groups.^35^ Testing this analog in the Minisci reaction gave a drastically different outcome, where 2-cyclohexylated product **3ak** formed in excellent yield and site selectivity toward the phosphonium-substituted ring (Table 3B). Although polyazine structures can vary considerably, this example was an encouraging starting point to assess the role of pyridylphosphinium salts in polyazine alkylation reactions more broadly.

**Table 3:**
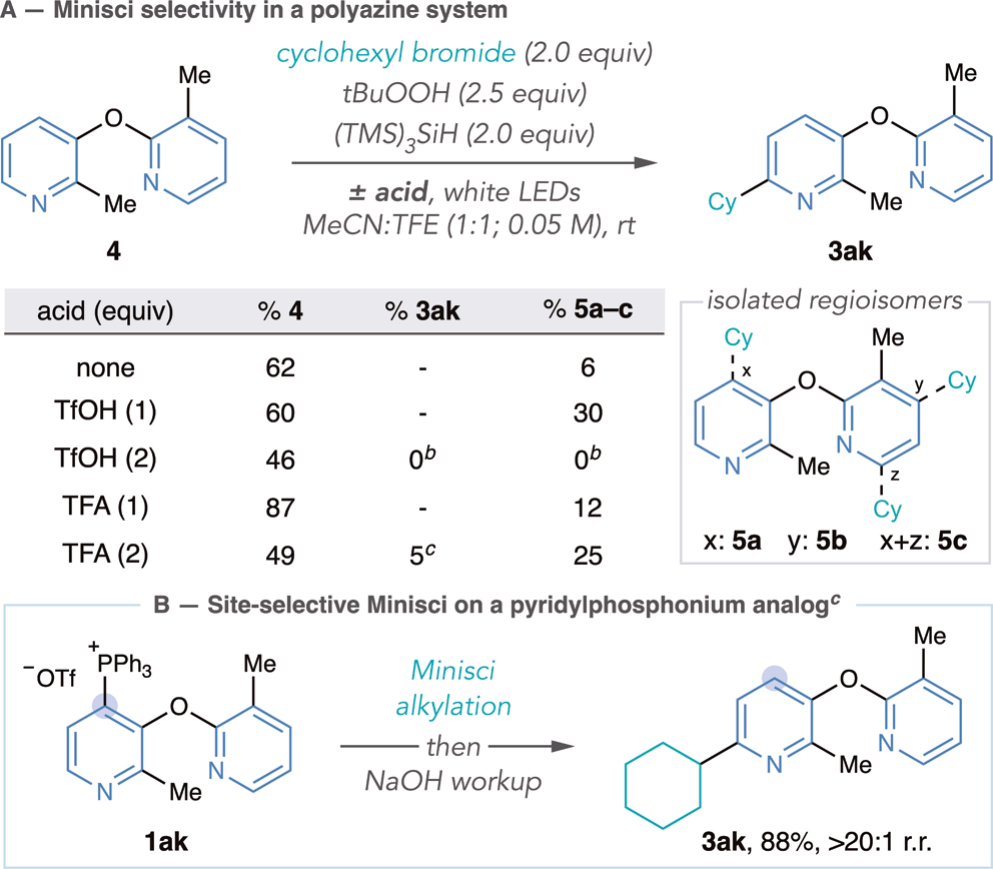
Model System for Polyazine Alkylation^a^

a ^1^H NMR yields based on 1,3,5-trimethoxybenzene as an internal standard.

b No alkylated products detected by LCMS.

c Isolated yield.

In Table 4, we assembled a series of polyazines to test the capacity of phosphonium ions to control site-selectivity. The table reports the isomeric ratios for phosphonium salt formation and the subsequent ratios after the Minisci alkylation and C–P bond cleavage. Bis-pyridine **3al** derives from a phosphonium salt that selectively forms on the mono-2-substituted pyridine versus the 2,6-disubstituted ring. This outcome occurs because of selective *N*Tf-activation of the less hindered heterocycle. Minisci alkylation occurs in good yield and with site-selectivity that mirrors the excellent selectivity observed during C–P bond formation. In the process of forming **3am**, phosphonium salt formation occurs preferentially on the 2-aryl substituted pyridine; in this case, we propose that *N*Tf-pyridinium salt formation selectively occurs on this ring because it is more Lewis basic than the 2-chloropyridine. We observed a decrease in site selectivity in the alkylation reaction compared to phosphonium salt formation. The initial 11:1 mixture of phosphonium salt isomers translates into a 6.9:1 ratio of alkylation products, favoring the 2-position of the pyridine. We did not observe alkylation of the 2-chloropyridine in this instance. In a related bis-pyridine system, **3an** results from a highly selective C–P bond-forming reaction followed by a site-selective Minisci process in good yield.

**Table 4:**
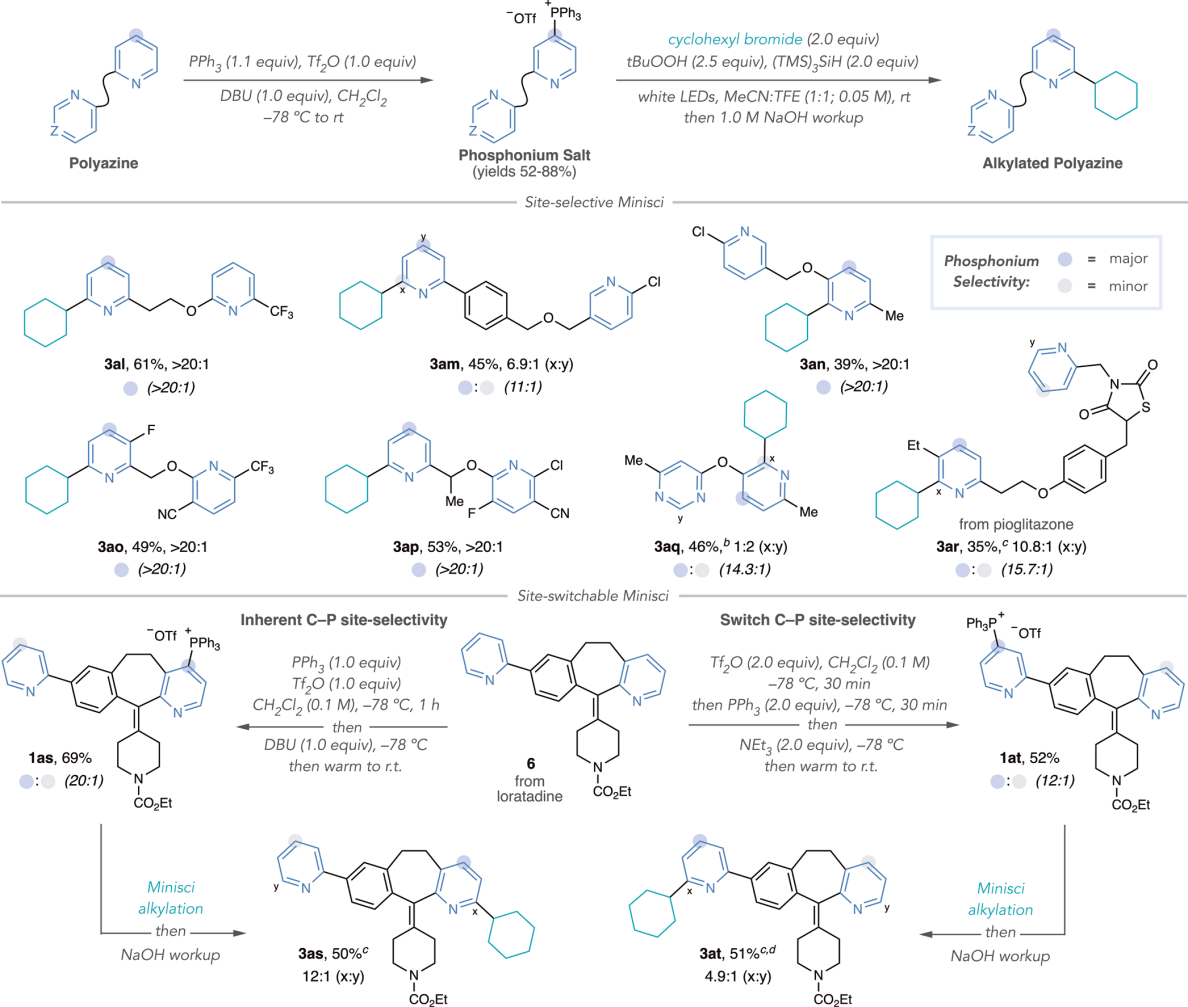
Site-Selective Alkylation of Polyazines^a^

a Isolated yields of products are shown unless stated. The x:y ratios refer to the crude ^1^H NMR ratio of isomeric alkylation products. Phosphonium salt ratios are derived from their ^1^H and ^31^P crude NMR spectra.

b Isolated as a 1:2 mixture of alkylated pyridine and pyrimidine isomers.

c Alkylation ran in MeCN/TFE (1:20, 0.02 M) and with 1.0 equiv of TfOH.

d Isolated with 4% of a *tert-*butylated product.

Bis-pyridine **3ao** formed in a >20:1 isomeric ratio after selective phosphonium salt formation and alkylation. In this case, it is instructive to examine the alkylation reaction of the same compound without phosphonium ion substitution. When we used the standard reaction conditions, we observed 16% of a 3:1 mixture of two indistinguishable alkylated isomers and 24% of residual starting material (see the Supporting Information, Section 1.4). Adding an equivalent of TfOH produced 24% of alkylated isomers and using one or two equivalents of TFA gave similar outcomes except in lower yields. However, it was possible to decipher that none of the alkylated isomers corresponded to 2-cyclohexylated pyridine **3ao**. This result is particularly surprising, as we expected the nucleophilic alkyl radical to add to the pyridine possessing electron-deficient cyano and CF_3_ groups. Bis-pyridine **3ap** also contains an electron-deficient ring, but we observed only one cyclohexylated isomer from the indicated phosphonium salt. These two examples contrast with the pyridine-pyrimidine structure **3aq**, where we observed a 1:2 mixture of isomers, with alkylation favoring the pyrimidine ring. However, when we tested the same structure without the phosphonium salt, we observed only minimal formation of alkylated products, except when TfOH or TFA was present, wherein a mixture of three isomers formed (see the Supporting Information, Section 1.4).

We transformed a pioglitazone analog into a phosphonium salt in a 15.7:1 mixture of isomers at the indicated pyridine C4 carbon atom. In the Minisci process, alkylation favored the pyridine 2-position of the major phosphonium isomer in modest yield (**3ar**). In our control reactions without phosphonium substitution, we found that alkylation occurs only when TFA is present, yielding a mixture of two cyclohexylated isomers in modest yield. Interestingly, alkylation occurs only on the monosubstituted pyridine, indicating that the phosphonium ion reverses the inherent selectivity of this compound (See the Supporting Information, Section 1.4). Collectively, these results undoubtedly show a substantial increase in controlled radical coupling when phosphonium ions are present.

In our previous report on selective pyridylphosphonium salt formation, we disclosed protocols for switching site-selectivity in polyazines.^35^ Here, we combined this approach with the regioselective Minisci process. As shown in Table 4, starting from loratadine analog **6**, we applied our standard C–P bond-forming protocol to obtain phosphonium salt **1as** as a 20:1 ratio of isomers. Minisci alkylation then resulted in 2-cyclohexylated isomer **3as** in a 12:1 isomeric ratio. Conversely, applying our base-mediated switch protocol formed phosphonium salt **1at**, again with high site selectivity. Minisci alkylation and phosphonium salt cleavage afforded cyclohexylated analog **3at**, with lower site selectivity than the initial C–P bond formation, but still with considerable bias toward the pyridine bearing the phosphonium salt. Testing **6** in the Minisci alkylation in the presence of TFA resulted in several mono- and polyalkylated products, including **3as** and **3at**, in low yields (See the Supporting Information, Section 1.4).

For the final part of the study, we showed a selection of pyridine difunctionalization reactions that exploit the versatile reactivity of the C–P bond (Scheme 2A). First, we performed a nucleophilic substitution reaction by adding an aryl thiolate to form thioether **7**.^26^ We then engaged phosphonium salt **2a** in a metal-catalyzed cross-coupling reaction to form Sonogashira product **8**.^36^ Aminated structure **9** was obtained in good yield using our previously reported azide coupling reaction, followed by hydrolysis, and we formed the C–O bond in **10** via coupling with an alkoxide.^25,37^ Expanding beyond triphenylphosphine enabled us to form products containing C–C bonds via phosphorus ligand coupling chemistry.^29^ After forming bis-heteroaryl phosphonium salt **2b′**, we subjected it to Minisci alkylation followed by acidic alcoholic conditions to promote ligand-coupling and obtained 4,4′-bipyridine **11** in good overall yield. We also obtained 4-deuteropyridine **12** in 81% yield by treating the intermediate *tert*-butylated pyridylphosphonium salt **2k** with MeOD/D_2_O (Scheme 2A).^28^ Lastly, Scheme 2B shows that a one-pot phosphonium salt formation, Minisci alkylation, C–P bond cleavage sequence is viable (**3a**).^38^

**Scheme 2:**
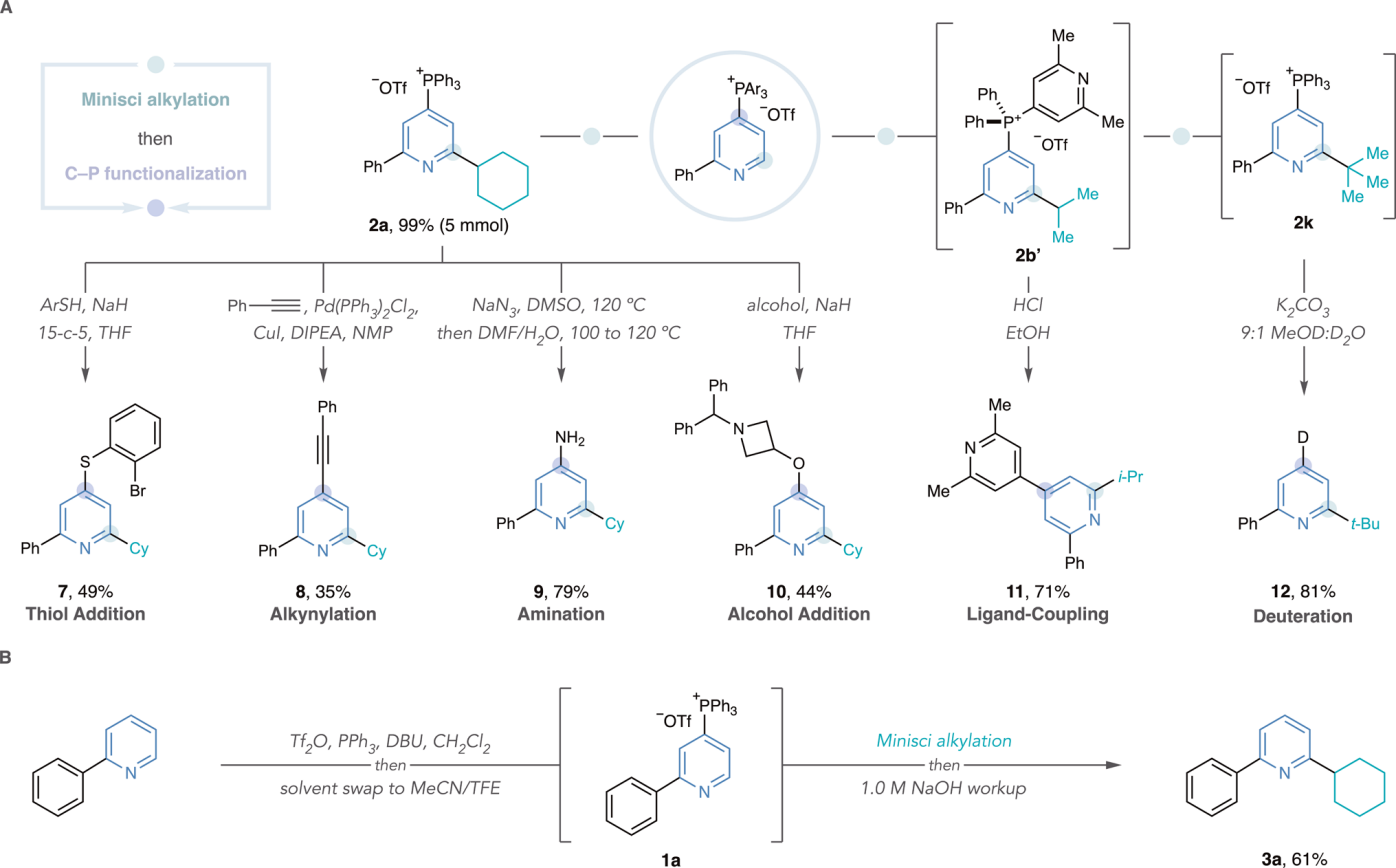
Difunctionalization and One-Pot Reactions^a^ ^,^ ^b^ ^,^ ^c^

a A. 2,4-Difunctionalization of pyridines via phosphonium salts. B. One-pot C2-selective alkylation of 2-phenylpyridine.
b Isolated yields of products are shown.
c Minisci alkylation conditions as shown in Table 4.

## Conclusions

In summary, we have discovered that phosphonium salts serve as activating and blocking groups in pyridine Minisci alkylation reactions. After installing the salts selectively at the 4-position, we found that they engage in 2-selective couplings with alkyl bromides under photoredox conditions without requiring acid activation. This protocol accommodates a range of pyridines, including those found in pharmaceutical and agrochemical compounds, and the C–P bond can be cleaved via a simple basic workup. Furthermore, installing a phosphonium salt into one pyridine within a polypyridine structure activates that ring toward Minisci alkylation over unactivated pyridines. This enables regio- and site-selective alkylation reactions in polypyridine systems, and selectivity is switchable between rings by controlling the site of phosphonium installation. The phosphonium salt also enables subsequent bond constructions resulting in 2,4-disubstituted pyridines. We anticipate that this method will be appealing to practitioners in drug and agrochemical discovery programs.

## Supplementary Material



## Notes

Experimental procedures, characterization data for reported compounds, and spectral data (PDF)

## Abbreviations

TMS: trimethylsilyl
TfOH: trifluoromethylsulfonic acid
Tf: triflyl
TFA: trifluoroacetic acid
DBU: 1,8-diazabicyclo­[5.4.0]­undec-7-ene
15-c-5: 15-crown-5
Ar: (hetero)­aryl
