# Supplementary material for: Phosphonium Ions as Activating Groups for the Selective Alkylation of Pyridines and Polyazines
Source: J Am Chem Soc. 2026 Jun 5;148(23):24531–7. doi: 10.1021/jacs.6c08076 (PMC13281535; doi:10.1021/jacs.6c08076)
Supplement: Supplementary file 1 [file ja6c08076_si_001.pdf]

Supporting Online Material for

Phosphonium Ions as Activating Groups for the Selective Alkylation of Pyridines and Polyazines

Dane A. Brunner, David C. Thomas, Kaila R. Steenback, Soren D. Rozema and Andrew McNally*

Department of Chemistry, Colorado State University,
Fort Collins, Colorado 80523, United States.
***andy.mcnally@colostate.edu**

TABLE OF CONTENTS

1. Experimental Section **2**

1.1 General Information **2**

1.2 Photoreactor Setup **4**

1.3 Optimization Studies **6**

1.4 Control Studies **9**

1.5 Preparation of Starting Materials **29**

1.6 Preparation of Heterocyclic Phosphonium Salts **36**

1.7 Alkylation of Heterocycles **55**

1.8 Control Experiments ^{1}H Spectra **92**

1.9 ^{1}H, ^{13}C, ^{19}F, and ^{31}P Spectra **114**

2 Computational Studies **368**

3 References **370**

1. Experimental Section

1.1 General Information

Proton nuclear magnetic resonance (^1H NMR) spectra were recorded at ambient temperature on a Varian 400 MR spectrometer (400 MHz), an Agilent Inova 400 (400 MHz) spectrometer, or a Bruker AV-111 400 (400 MHz) spectrometer. Chemical shifts (δ) are reported in ppm and quoted to the nearest 0.1 ppm relative to the residual protons in $CDCl_3$ (7.26 ppm), CD_3OD (3.31 ppm), $(CD_3)_2CO$ (2.05 ppm), CD_3CN (1.94 ppm), D_2O (4.79 ppm), or $(CD_3)_2SO$ (2.50 ppm) and coupling constants (*J*) are quoted in Hertz (Hz). Data are reported as follows: Chemical shift (multiplicity, coupling constants, number of protons). Coupling constants were quoted to the nearest 0.1 Hz and multiplicity reported according to the following convention: s = singlet, d = doublet, t = triplet, q = quartet, qn = quintet, sext = sextet, sp = septet, m = multiplet, br = broad. Where coincident coupling constants have been observed, the apparent (*app*) multiplicity of the proton resonance has been reported. Carbon nuclear magnetic resonance (^{13}C NMR) spectra were recorded at ambient temperature on a Varian 400 MR spectrometer (100 MHz), an Agilent Inova 400 (100 MHz) spectrometer, or a Bruker AV-111 400 (100 MHz) spectrometer. Chemical shift (δ) was measured in ppm and quoted to the nearest 0.01 ppm relative to the residual solvent peaks in $CDCl_3$ (77.16 ppm), CD_3OD (49.00 ppm), $(CD_3)_2CO$ (29.84 ppm), CD_3CN (1.32 ppm), D_2O, or $(CD_3)_2SO$ (39.52 ppm).

Low-resolution mass spectra (LRMS) were measured on an Agilent 6310 Quadrupole Mass Spectrometer. Infrared (IR) spectra were recorded on a Nicolet IS-50 FT-IR spectrometer as either solids or neat films, either through direct application or deposited in $CHCl_3$, with absorptions reported in wavenumbers (cm^{-1}). Analytical thin layer chromatography (TLC) was performed using pre-coated Silicycle glass backed silica gel plates (Silicagel 60 F254). Manual flash column chromatography was undertaken on Silicycle silica gel Siliaflash P60 40-63 mm (230-400 mesh) under a positive pressure of air unless otherwise stated. Automated flash column chromatography was undertaken using a Teledyne Isco CombiFlash NextGen 300+ using 12 g RediSep Gold Normal-Phase Silica cartridges. Visualization was achieved using ultraviolet light (254 nm) and chemical staining with a chamber of I_2 in SiO_2, ceric ammonium molybdate, or basic potassium permanganate solutions as appropriate. Melting points (mp) were recorded using a Büchi B-450 melting point apparatus and are reported uncorrected.

Acetonitrile (MeCN), 2,2,2-trifluoroethan-1-ol (TFE), and dichloromethane (CH_2Cl_2) were dried and distilled using standard methods.[1] Methanol, ethyl acetate, hexanes, chloroform, CH_2Cl_2, and acetone were purchased anhydrous from Sigma Aldrich chemical company. All reagents were purchased at the highest

commercial quality. Reactions were carried out under an atmosphere of nitrogen unless otherwise stated. All reactions were monitored by TLC, ^{1}H NMR spectra taken from reaction samples, and liquid chromatography mass spectrometry (LCMS) using an Agilent 6310 Quadrupole Mass Spectrometer for MS analysis. Trifluoromethanesulfonic anhydride (Tf_2O) (99%), trifluoromethanesulfonic acid (TfOH), triphenylphosphine (PPh_3), 2-hydroperoxy-2-methylpropane (*t*BuOOH) (5-6 M in decane), tris(trimethylsilyl)silane ($(TMS)_3SiH$) were purchased from Oakwood Chemical and used without further purification but were routinely stored in a –20 °C freezer. Triethylamine (Et_3N) and 1,8-diazabicyclo[5.4.0]undec-7-ene (DBU) were distilled before use. Trifluoroacetic acid (TFA; 98%) and 2-hydroperoxy-2-methylpropane (*t*BuOOH) (90% in H_2O) were purchased from Sigma Aldrich chemical company and used without further purification but was routinely stored in a –20 °C freezer. Deuterated solvents were purchased from Cambridge Isotope Laboratories, Inc. and were used without further purification.

1.2 Photoreactor Setup

All photochemical reactions were conducted under air. Ambient temperature was maintained by using a compressed air line.

If using the two 455 nm kessil lamps (Kessil PR160 set at 100% intensity or Kessil H150-Blue), vials containing the reaction mixture were centered on the stir plate approximately 6 inches away from the light source (Figure S1). Be sure to wear proper protective eyewear that blocks harmful wavelengths of light when lights are in use until the light has been properly covered or blocked. An example of appropriate protective goggles from HepatoChem is provided here: Skyper eyewear protective goggles.[2] Note: the reactions shown below were blocked fully with cardboard after the lights were turned on to prevent human exposure to harmful wavelengths of light.

Photochemical reactor setups consisted of an LED ribbon wrapped around the inside of a Pyrex beaker or LED bulbs adhered to the inside of a Pyrex beaker. The different LED ribbons (red, white, and blue LEDs) were purchased from Amazon.[3] If using the LED ribbons or LED bulbs, vials containing reaction mixture were placed in the center of the photoreactor beaker that was centered on a stir plate (Figures S2 and S3). Ambient temperature was maintained by using a compressed air line placed above the photoreactor setup. No difference in yield is observed using either the LED ribbon or LED bulb photoreactor setups.

Figure S1. Kessil Light Photoreactor Setup with Light Off and On

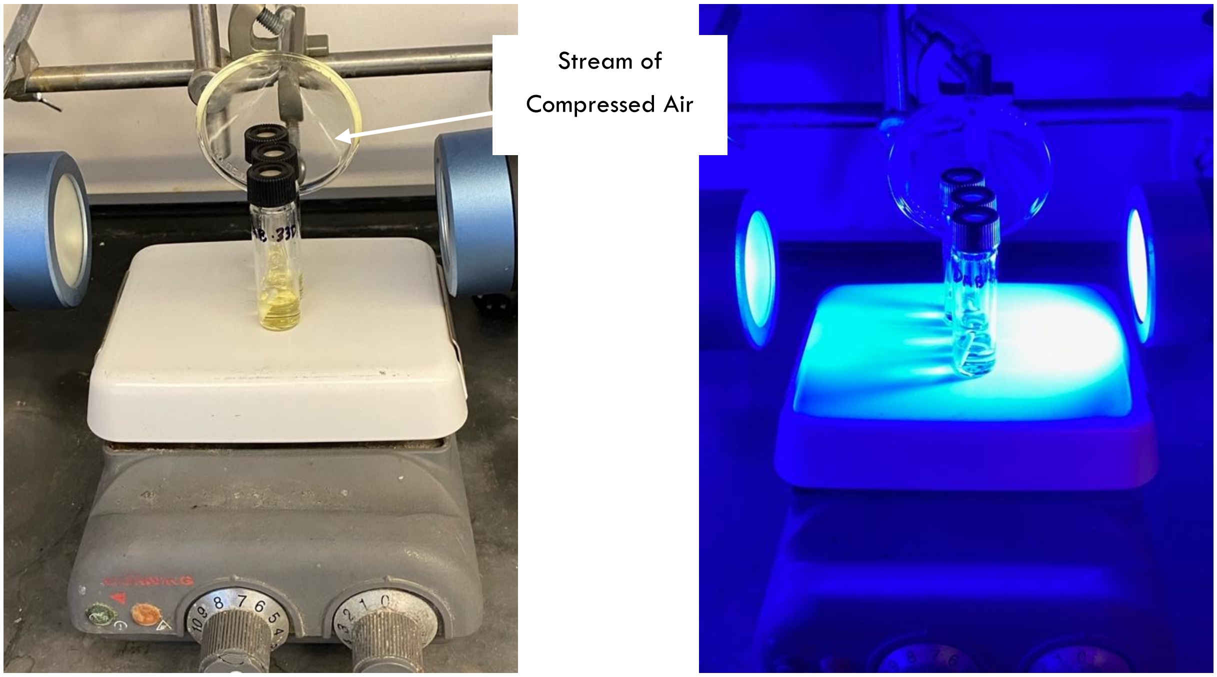

Figure S2. White LED Ribbon Photoreactor Setup with Lights On

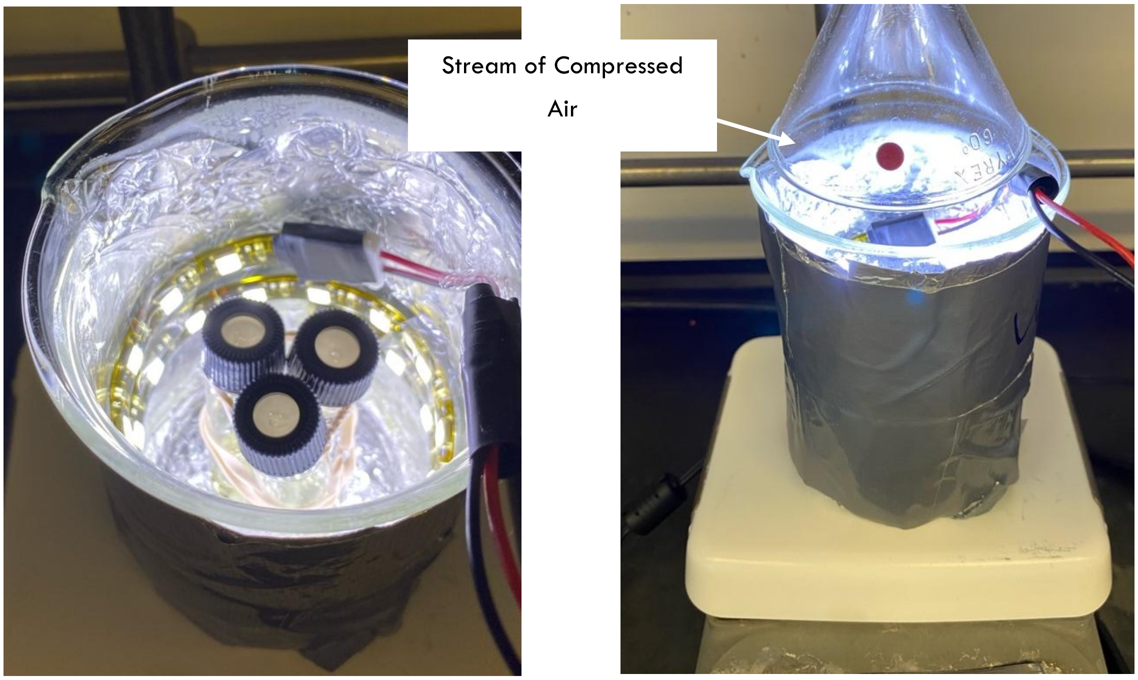

Figure S3. White LED Bulb Photoreactor Setup with Lights On

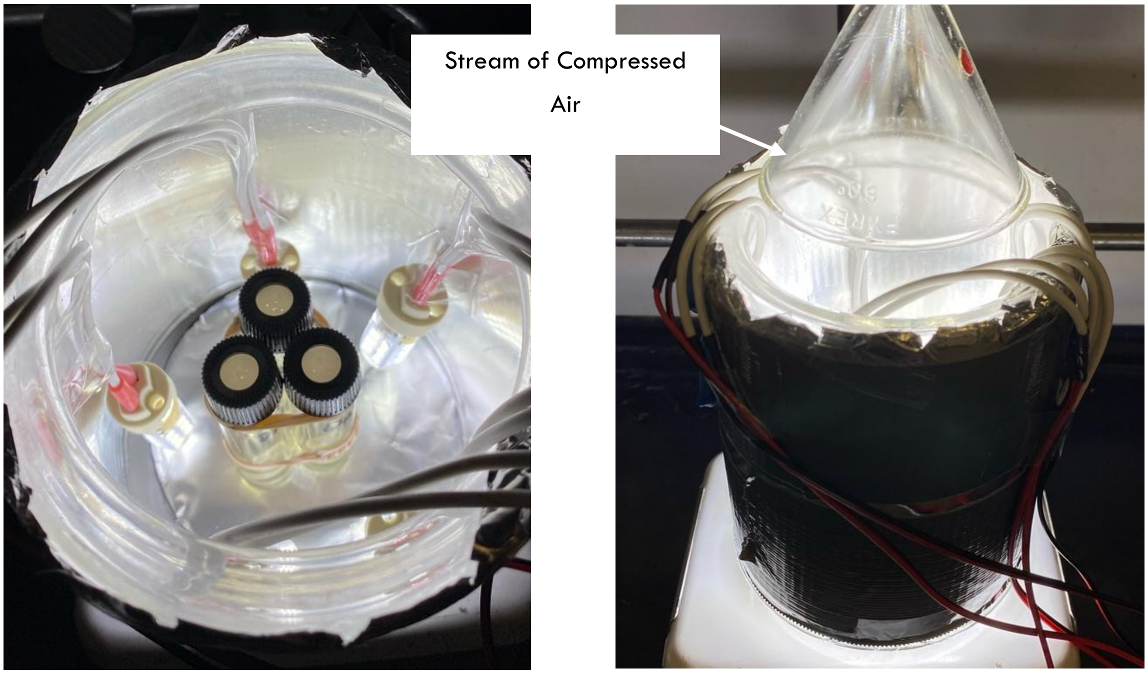

1.3 Optimization Studies

Reaction optimization adhered to the following procedure: an oven-dried 8 mL vial equipped with a magnetic stir bar was charged with triphenyl(2-phenylpyridin-4-yl)phosphonium trifluoromethanesulfonate (56.6 mg, 0.10 mmol, 1.00 equiv), solvent, trifluoroacetic acid, peroxide, bromocyclohexane, and $(TMS)_3SiH$ added via syringe. The vial was sealed and irradiated with the stated light source according to the photoreactor setups shown in Figure S1–3 for 16-24 hours. Triphenylmethane (24.4 mg, 0.10 mmol, 1.00 equiv) was added as a 1.00 M solution in CH_2Cl_2 as an internal standard. After vigorous stirring, an aliquot from the reaction mixture was then concentrated *in vacuo* and redissolved in $CDCl_3$ for ^1H and ^{31}P NMR and LRMS analysis. The ^1H NMR integral values were used to calculate the data give in Tables S1–3.

Table S1. Effect of different light sources on the alkylation of 2-phenylpyridine triphenylphosphonium salt.[a]

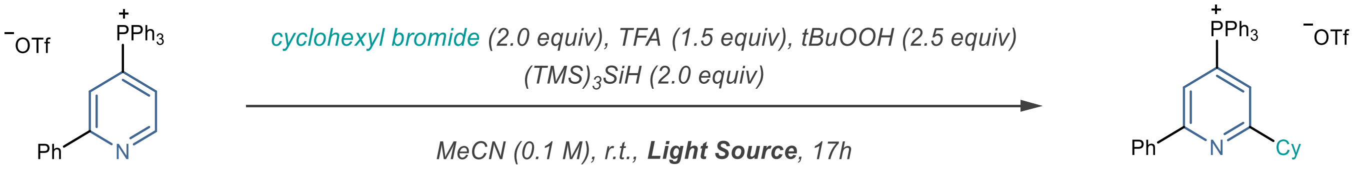

Entry	*Light Source*	*SM Phosphonium*	*2-Phenylpyridine*	*Pdt Phosphonium*
1	Blue LEDs	35	2	41
2	White LEDs	3	n.d.	74
3	Red LEDs	37	1	43
4	455 nm Kessil	n.d.	2	53

[a]r.t.: Room temperature; SM: Starting material; Pdt: Product; n.d.: Not detected.

Table S2. Effect of different solvents and peroxides on the alkylation of 2-phenylpyridine triphenylphosphonium salt.[a]

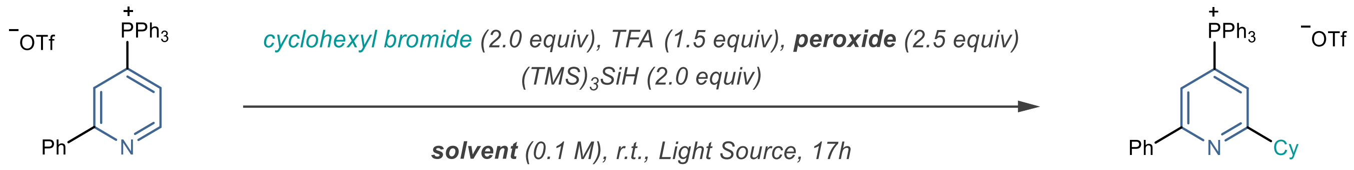

Entry	*Solvent*	*Peroxide*	*SM Phosphonium*	*2-Phenylpyridine*	*Pdt Phosphonium*
1	MeCN	*t*BuOOH (90% in H_2O)	16	n.d.	70
2	MeOH	*t*BuOOH (90% in H_2O)	n.d.	n.d.	n.d.
3	Acetone	*t*BuOOH (90% in H_2O)	24	1	54
4	CH_2Cl_2	*t*BuOOH (90% in H_2O)	48	1	32
5	TFE	*t*BuOOH (90% in H_2O)	54	n.d.	40
6	MeCN	*t*BuOOH (5.5 M in decane)	15	n.d.	66
7	MeCN	H_2O_2 (30% in H_2O)	17	n.d.	23
8	MeCN	Benzoyl Peroxide	57	n.d.	28
9	MeCN	Cumene Hydroperoxide	59	n.d.	32
10	MeCN	None	54	10	3

[a]r.t.: Room temperature; SM: Starting material; Pdt: Product; n.d.: Not detected.

Table S3. Effect of different concentrations, acid equivalents, peroxide equivalents, and bromide/silane equivalents on the alkylation of 2-phenylpyridine triphenylphosphonium salt.[a]

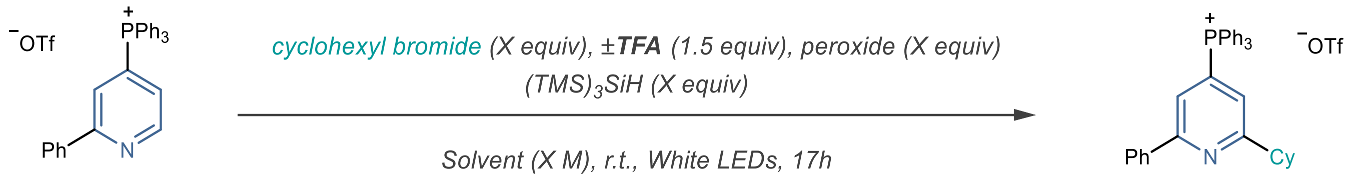

Entry	*MeCN (M)*	*± TFA equiv*	*tBuOOH (90% in H_2O) equiv*	*Bromide/ Silane equiv*	*SM Phosphonium*	*2-Phenylpyridine*	*Pdt Phosphonium*
1	0.1	1.5	2.0	2	n.d.	n.d.	68
2	0.1	1.5	2.5	2	3	n.d.	74
3	0.1	1.5	3.0	2	18	n.d.	68
4	0.05	1.5	2.5	2	9	n.d.	75
5	0.03	1.5	2.5	2	18	n.d.	65
6	0.05	1.5	2.5	1	64	1	16
7	0.05	1.5	2.5	3	1	3	81
8	0.05	0	2.5	2	15	n.d.	70
9	0.05 (1:1 MeCN: TFE)	0	2.5	2	n.d.	n.d.	99
10	0.05 (1:1 MeCN: TFE)	0	2.5 (5.5 M in decane)	2	n.d.	n.d.	99 (90)[b]

[a]r.t.: Room temperature; SM: Starting material; Pdt: Product; n.d.: Not detected. [b]Isolated yield.

1.4 Control Studies

The following series of experiments show a Minisci alkylation protocol applied to polyazines without phosphonium substitution. We conducted these experiments in the presence and absence of Bronsted acids. Yields in Table S4-8 are based on the crude ^{1}H NMR spectra with 1,3,5-trimethoxybenzene as the internal standard, unless stated. We either use azine protons integrations to calculate 1H NMR yields, or the cyclohexyl methine protons. In each reaction, we purified the crude mixture by preparative TLC to characterize the alkylated isomers. However, in most cases it is not possible to completely separate the alkylated mixtures and so we have grouped alkylated isomers together when reporting yields.

Table S4. Minisci alkylation example on ***3ak*** C–H precursor.[a]

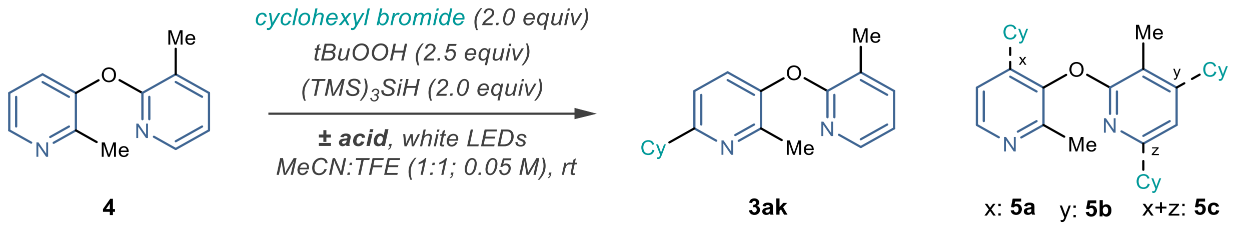

Entry	*Acid*	*SM Pyridine*	*Mono Alkylated Pyridines*	***3ak***
1	none	62	6	0
2	TFA (1 equiv)	87	12	0
3	TFA (2 equiv)	49[b]	25 (16)[b]	5[b]
4	TfOH (1 equiv)	60	30	0
5	TfOH (2 equiv)	46	0	0

[a]r.t.: Room temperature; SM: Starting material; n.d.: Not detected. [b]Isolated yields from 1 mmol scale reaction reported.

We were able to separate and characterize **3ak**, **5a**, **5b**, and **5c** (see spectra below), and we elected to group **5a-c** and calculate combined ^{1}H NMR yields.

Crude, ^{1}H NMR ($CDCl_3$, 400 MHz)

2 equiv TFA

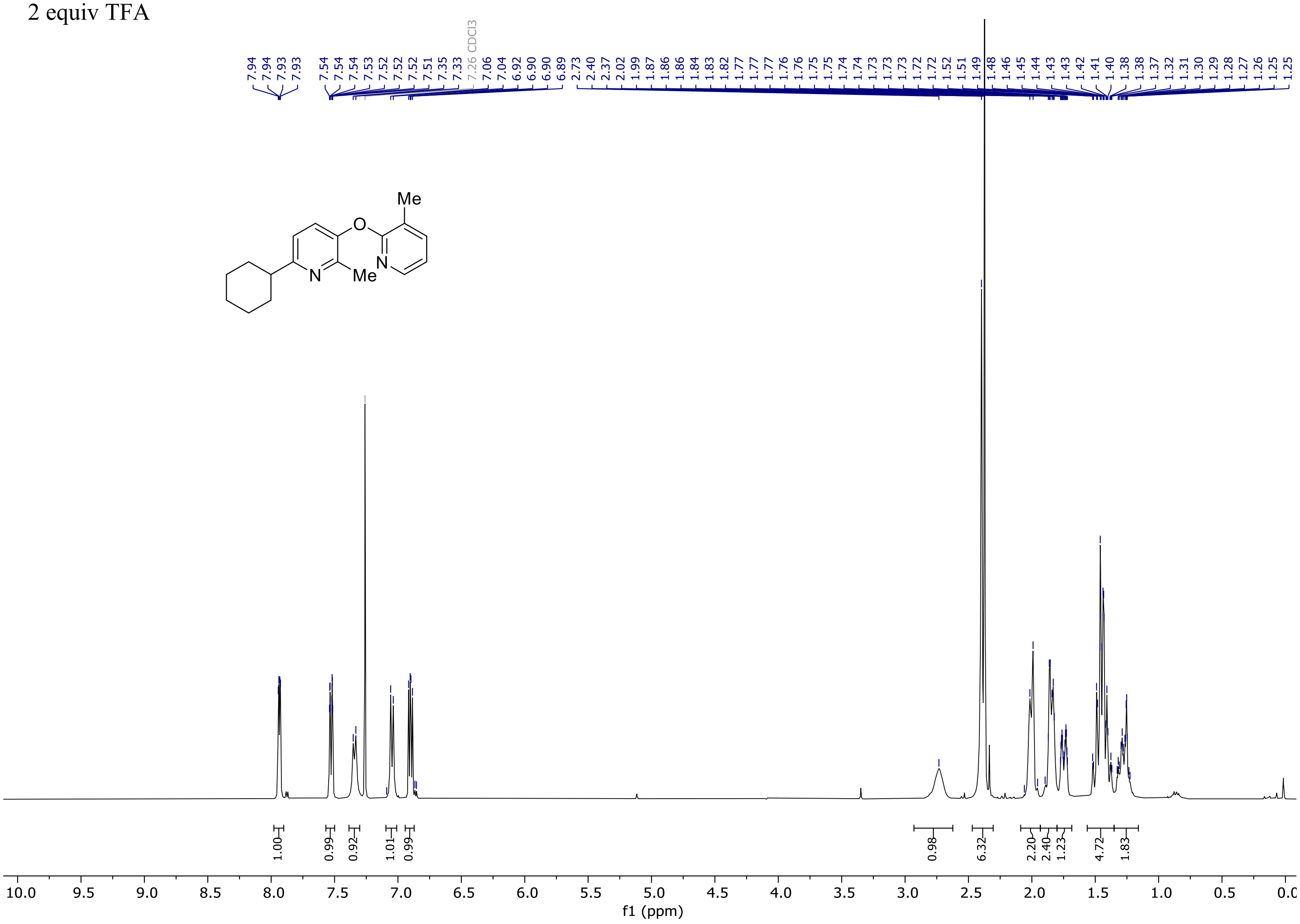

Crude, ^{1}H NMR ($CDCl_3$, 400 MHz)

2 equiv TFA

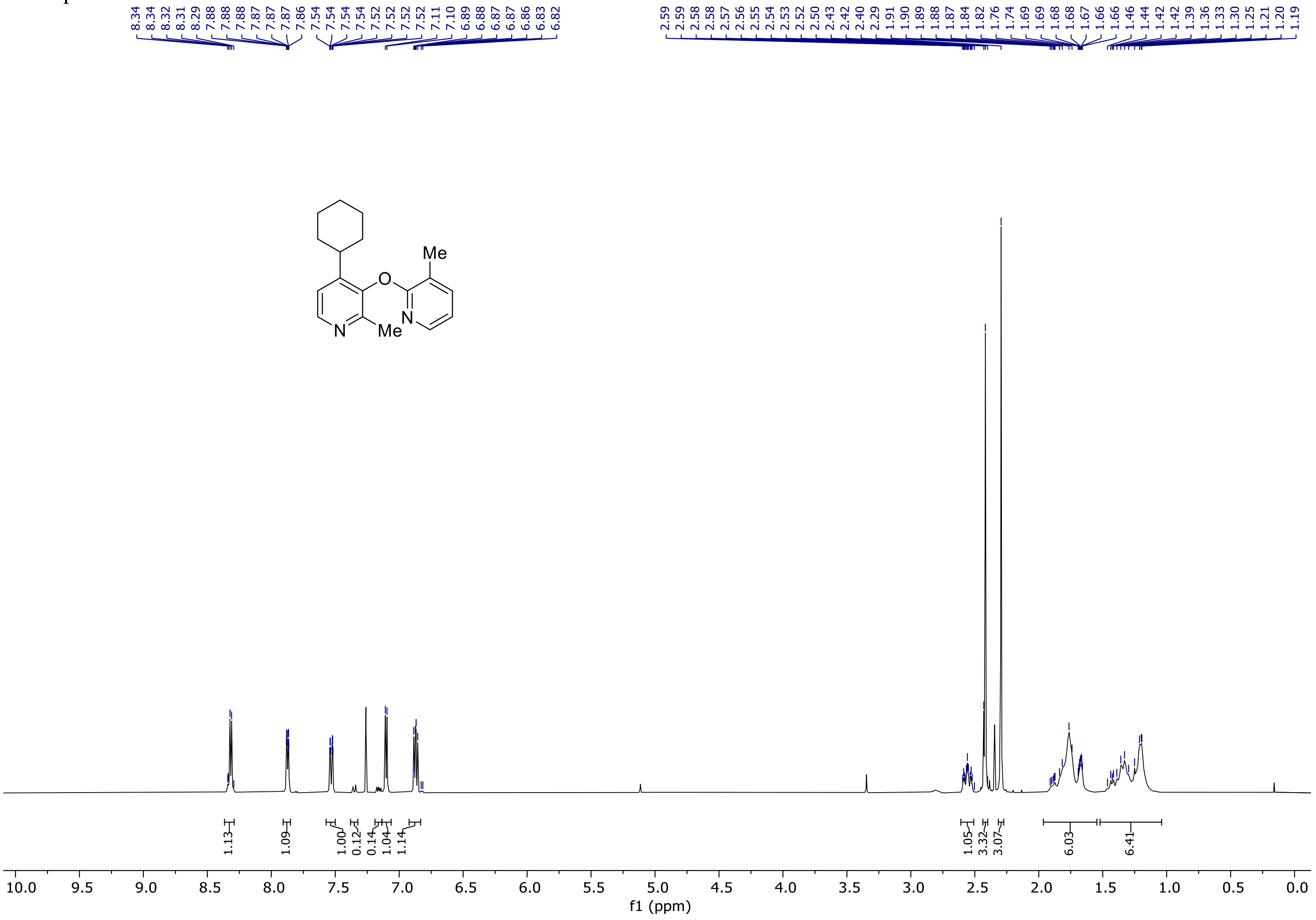

Crude, ^{1}H NMR ($CDCl_3$, 400 MHz)

2 equiv TFA

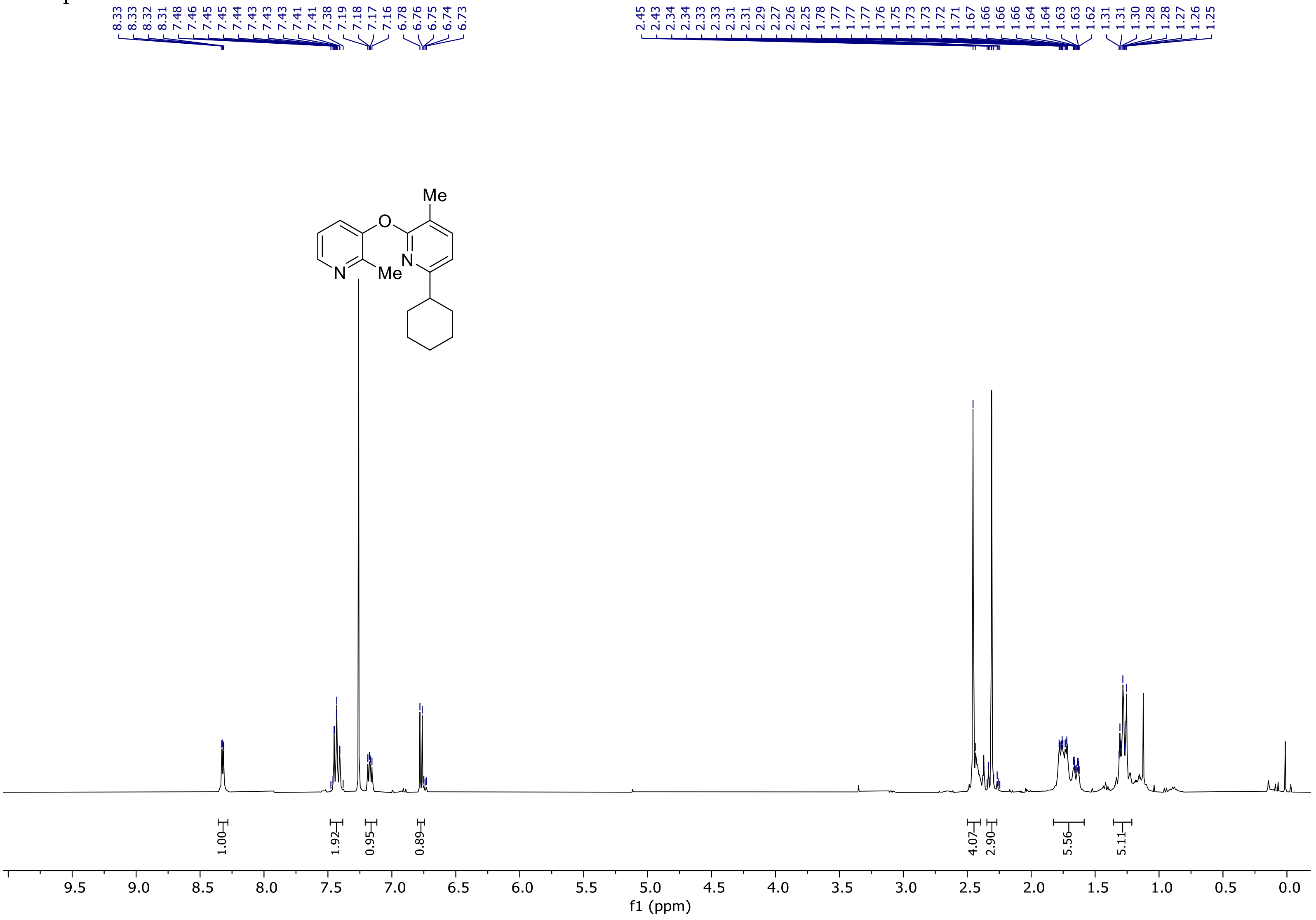

Table S5. Minisci alkylation example on ***3at*** C–H precursor. [a]

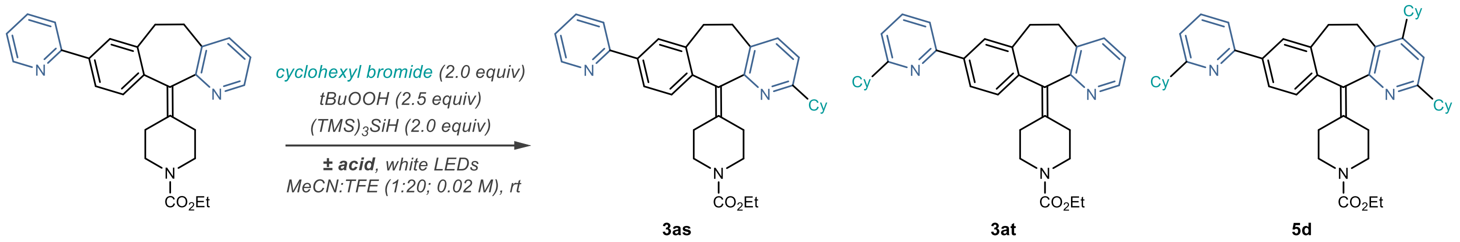

Entry	*Acid*	*SM Pyridine*	*Mono Alkylated Pyridines*	***3at***
1	none	93	0	0
2	TFA (1 equiv)	76	6	9
3[b]	TFA (2 equiv)	41	15	6
4	TfOH (1 equiv)	90	0	0
5	TfOH (2 equiv)	70	0	0

[a]r.t.: Room temperature; SM: Starting material; n.d.: Not detected. [b]Isolated yields from 1 mmol scale reaction reported.

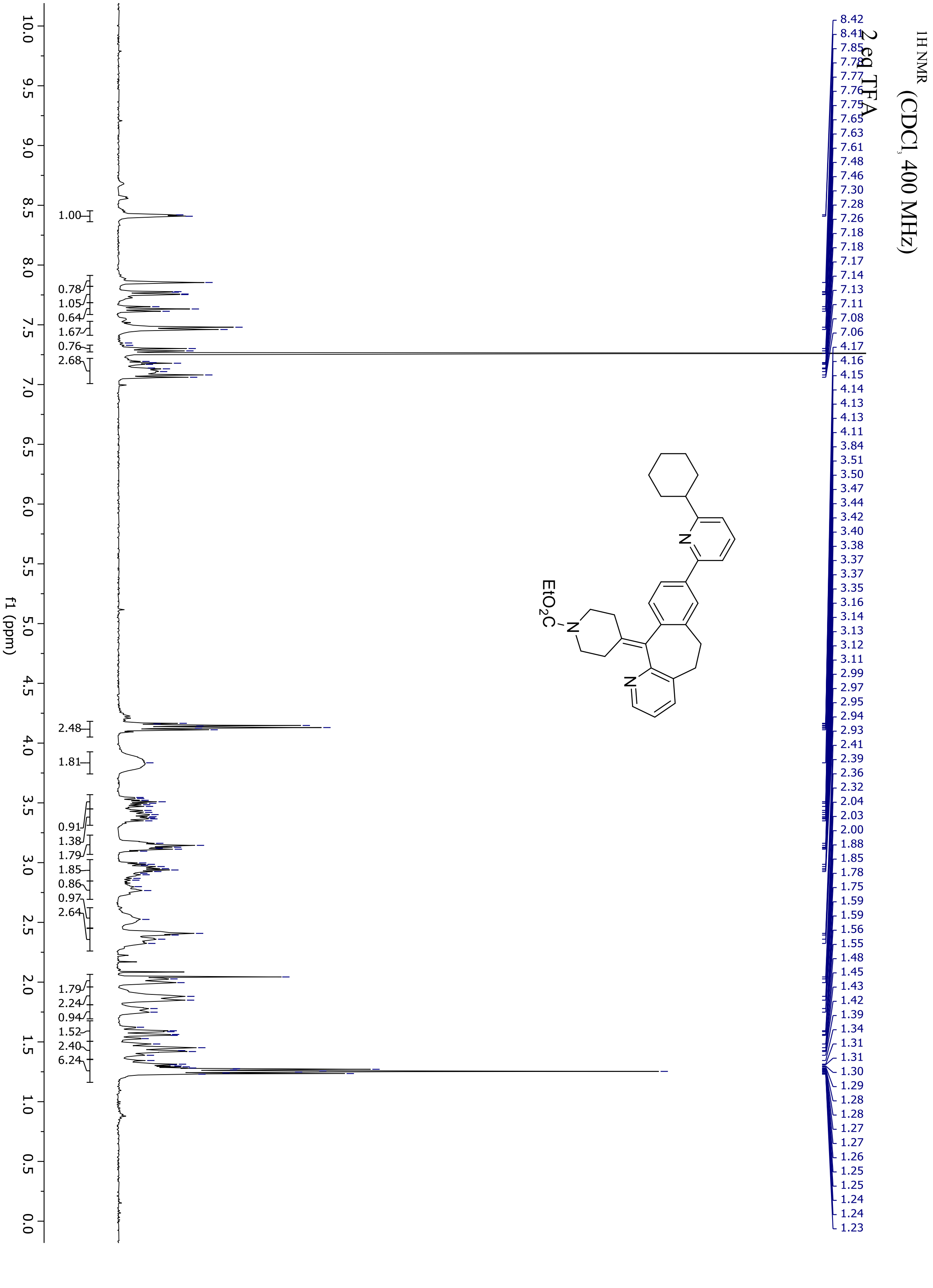

1H NMR (CDCl3, 400 MHz)
2 eq TFA
EtO2C
N
N
N
f1 (ppm)

^{1}H NMR ($CDCl_3$ 400 MHz)

2 eq TFA

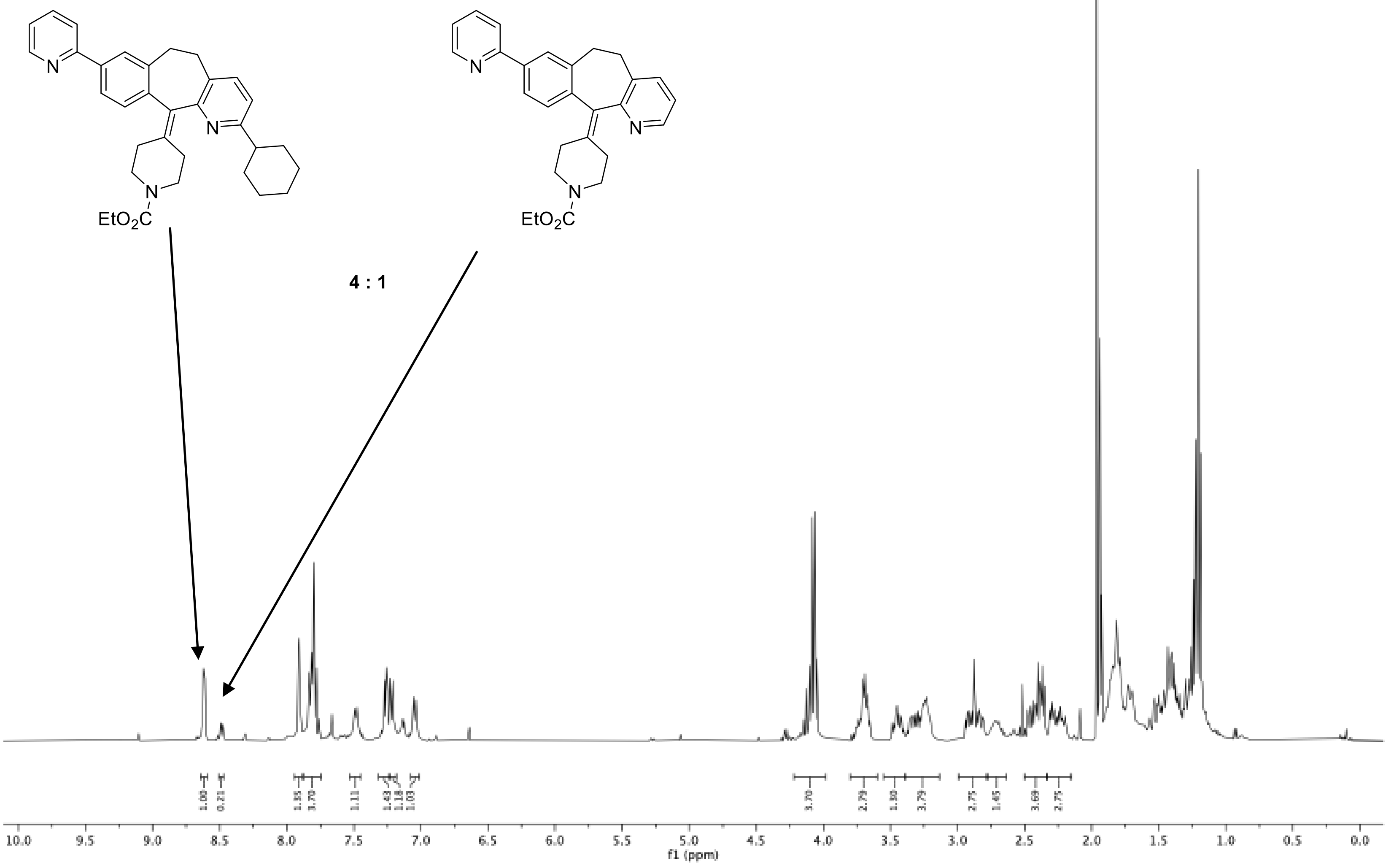

1H NMR (CDCl$_3$ 400 MHz)
2 eq TFA

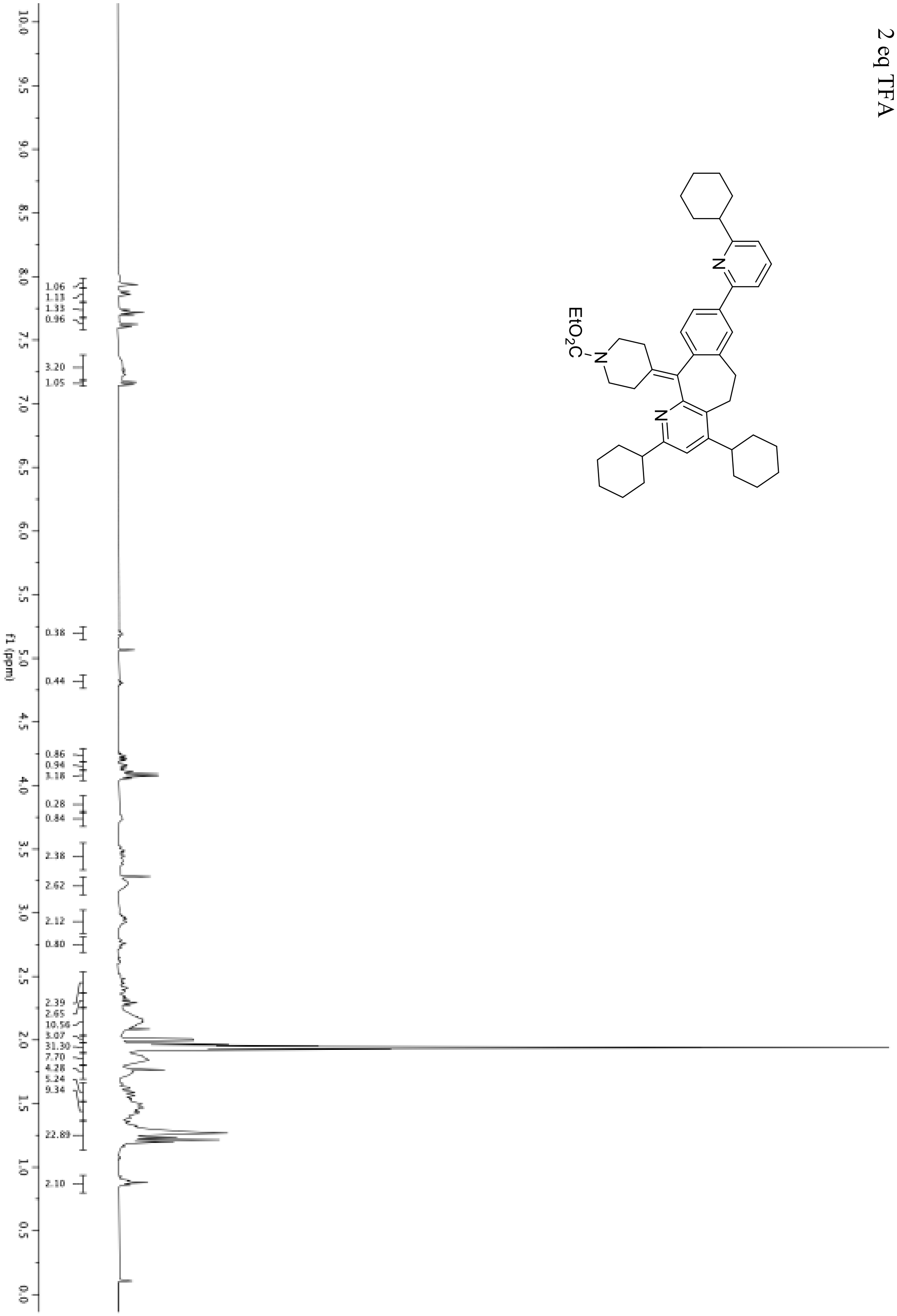

Table S6. Minisci alkylation example on ***3ao*** C–H precursor. [a]

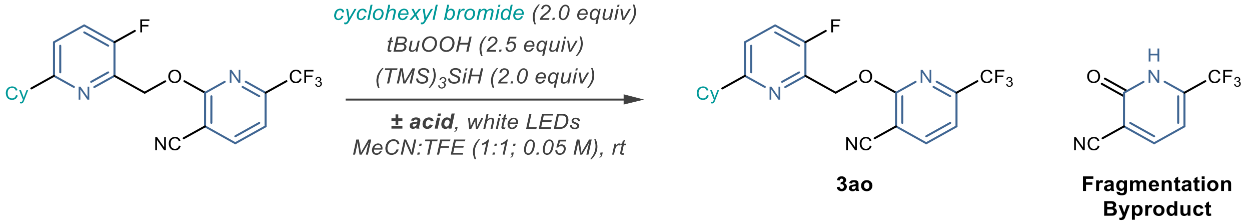

Entry	*Acid*	*SM Pyridine*	*Fragmentation Byproduct(s)*	*Mono Alkylated Pyridines*	***3ao***
1	none	24	58	16	0
2	TFA (1 equiv)	6	53	7	0
3	TFA (2 equiv)	8	51	5	0
4	TfOH (1 equiv)	33	47	24	0
5	TfOH (2 equiv)	41	47	0	0

[a]r.t.: Room temperature; SM: Starting material; n.d.: Not detected

In this reaction, we observed byproducts derived from cleavage of the heterobenzylic C-O bond. We tentatively assign these byproducts as the following structures. Note that similar cleavage products also form when the phosphonium ion is present.

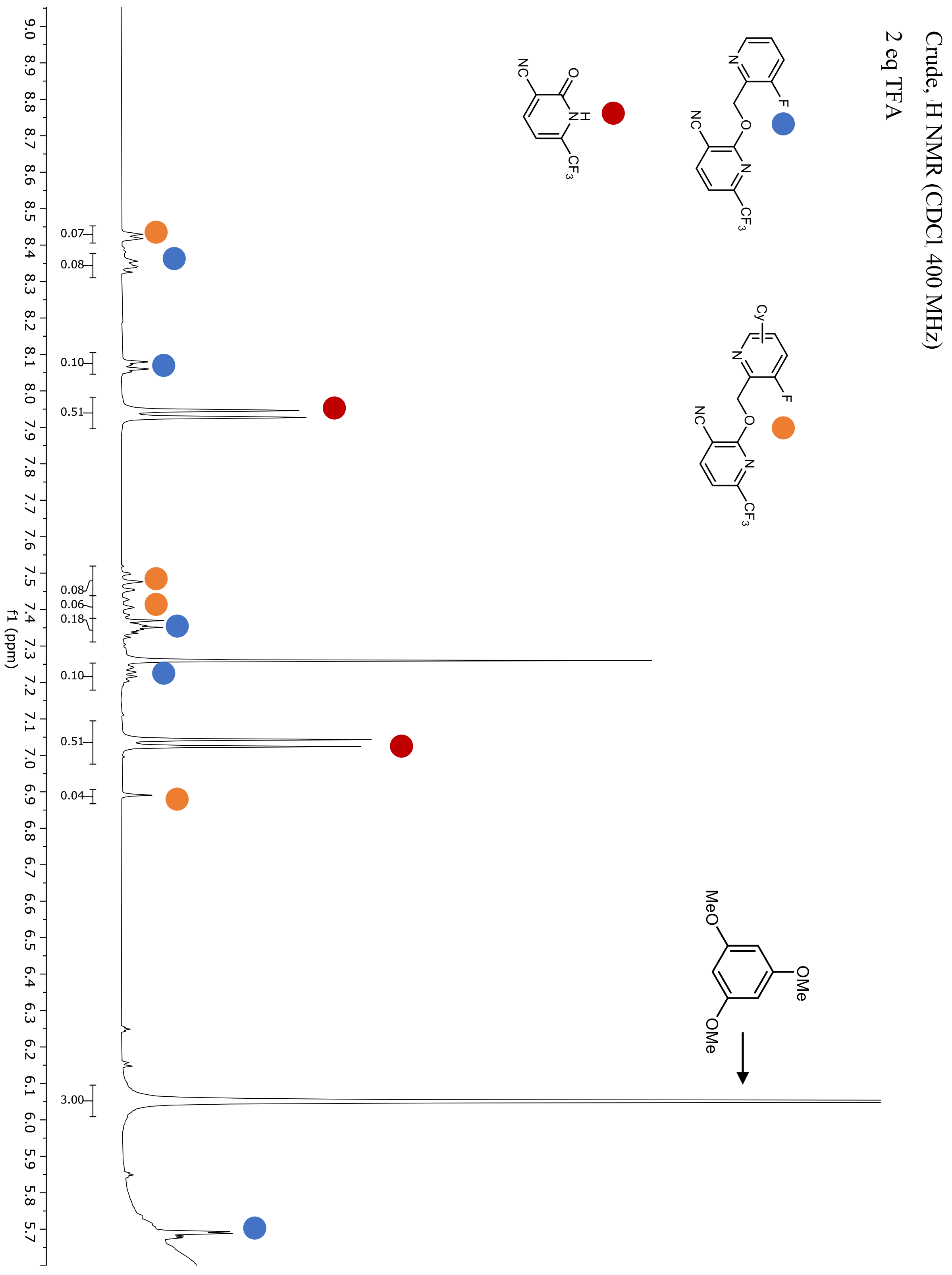

Crude, 1H NMR (CDCl3, 400 MHz)
2 eq TFA
f1 (ppm)
0.07
0.08
0.10
0.51
0.08
0.06
0.18
0.10
0.51
0.04
3.00
MeO
OMe
OMe
NC
CF3
Cy
F

Table S7. Minisci alkylation example on ***3ar*** C–H precursor. [a]

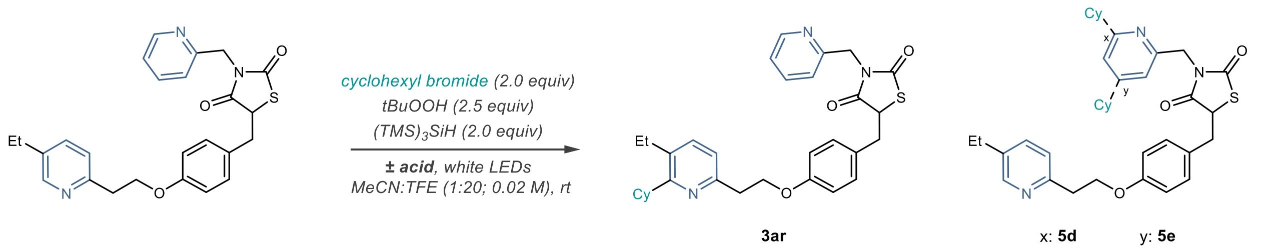

Entry	*Acid*	*SM Pyridine*	*Mono Alkylated Pyridines*	***3ar***
1	none	90	0	0
2	TFA (1 equiv)	87	13	0
3[b]	TFA (2 equiv)	42	31	0
4	TfOH (1 equiv)	84	0	0
5	TfOH (2 equiv)	80	0	0

[a]r.t.: Room temperature; SM: Starting material; n.d.: Not detected. [b]Isolated yields from 1 mmol scale reaction reported.

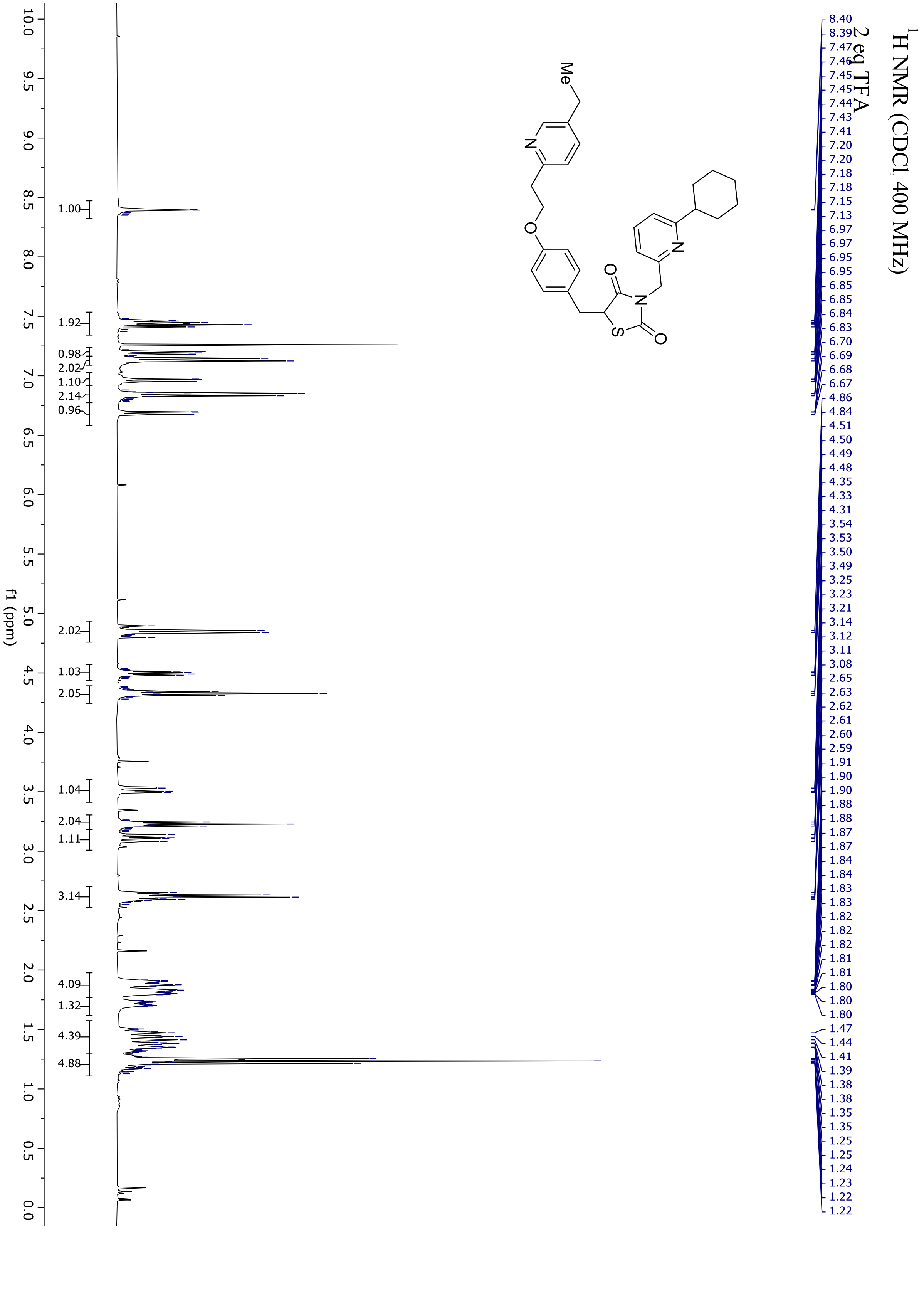

1H NMR (CDCl3 400 MHz)
2 eq TFA
f1 (ppm)

1H NMR (CDCl 400 MHz)

2 eq TFA

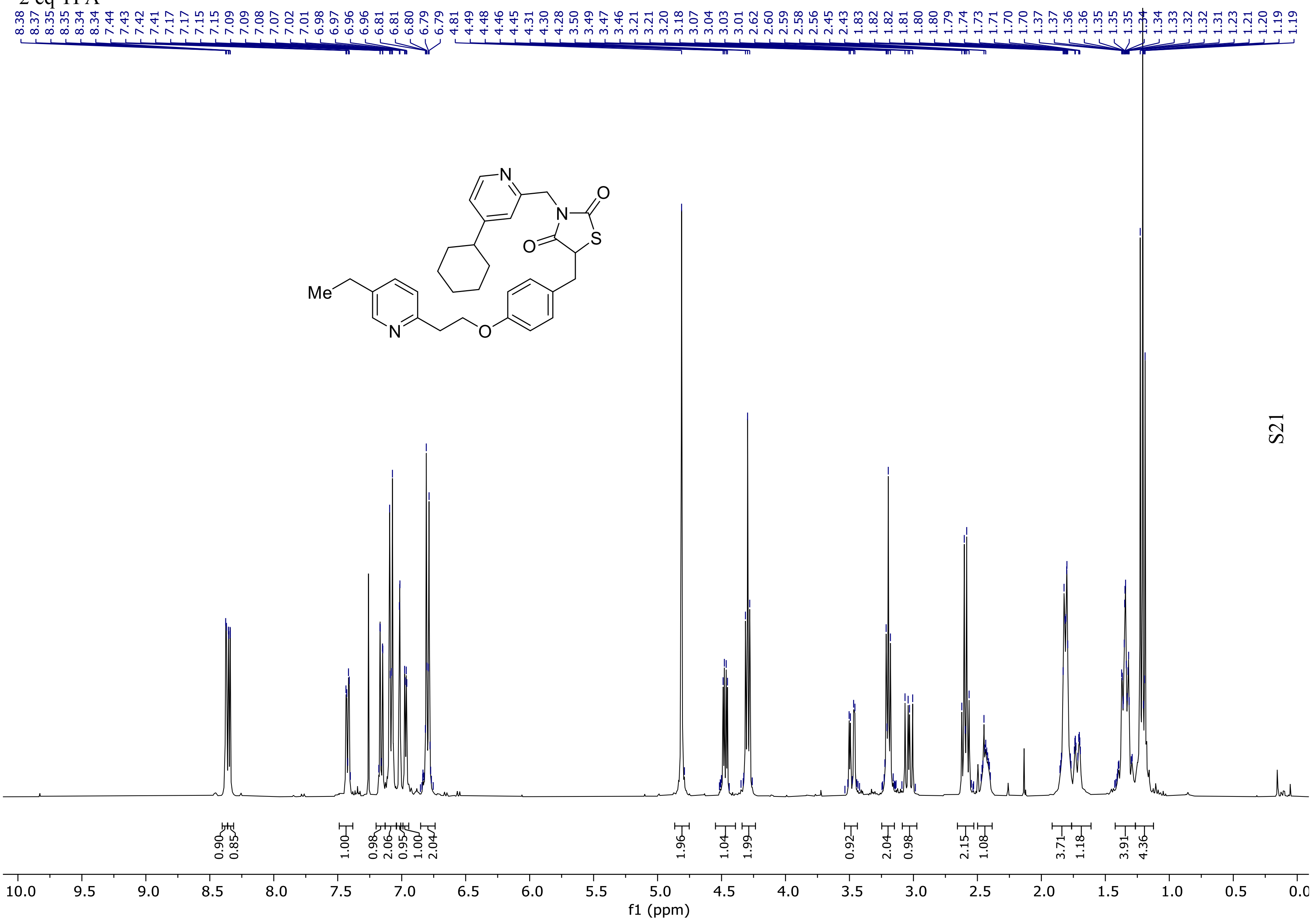

Table S8. Minisci alkylation example on ***3aq*** C–H precursor. [a]

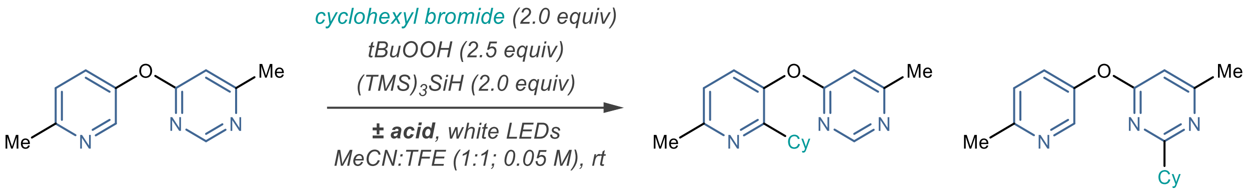

Entry	*Acid*	*SM Pyridine*	*Mono Alkylated azines*	***3aq***
1	none	33	12	0
2	TFA (1 equiv)	33	20	3
3[b]	TFA (2 equiv)	24	20	8
4	TfOH (1 equiv)	26	9	0
5	TfOH (2 equiv)	43	0	0

[a]r.t.: Room temperature; SM: Starting material; n.d.: Not detected. [b]Isolated yields from 1 mmol scale reaction reported.

1H NMR (CDCl 400 MHz)

2 eq TFA

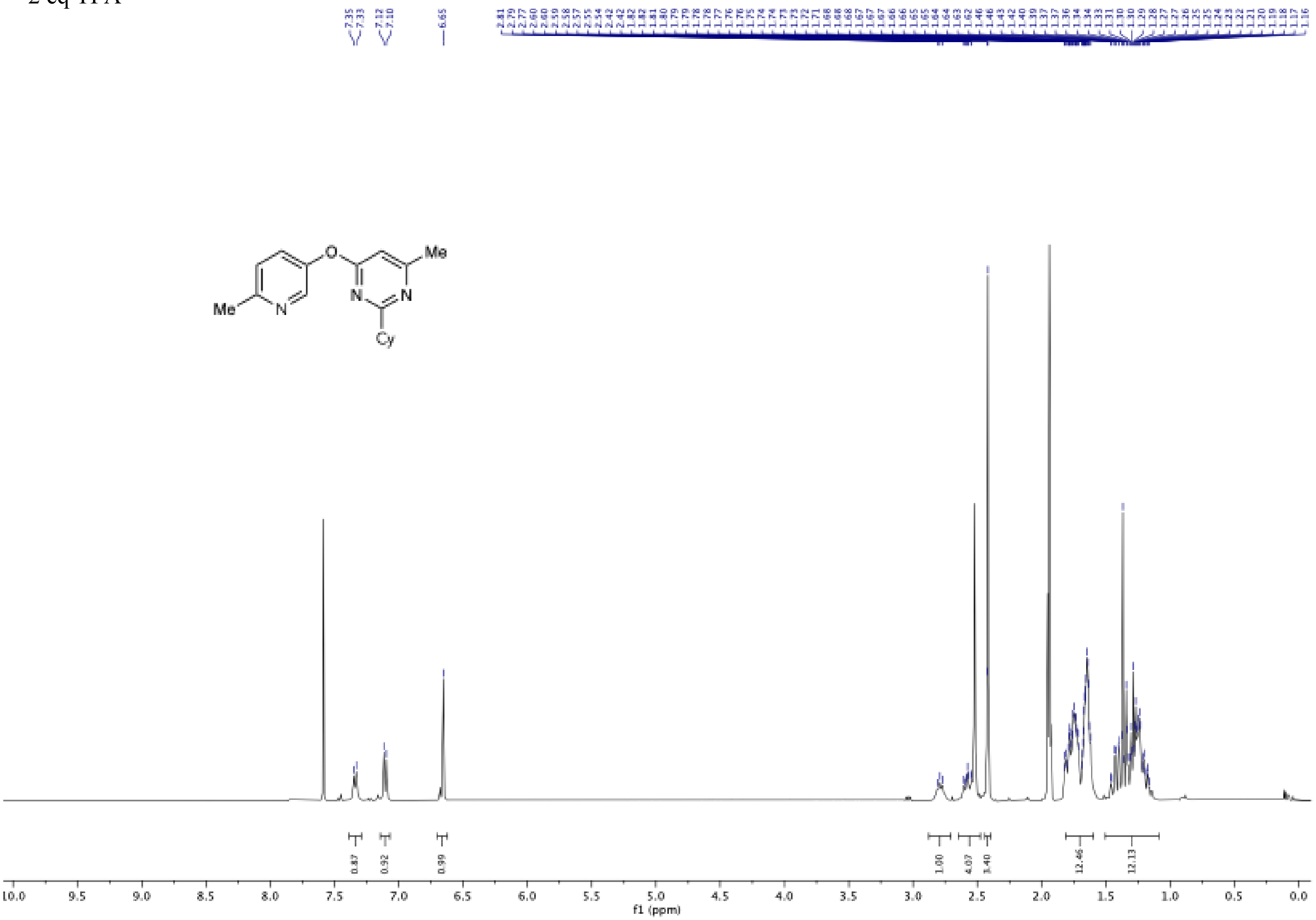

1H NMR (CDCl 400 MHz)

2 eq TFA

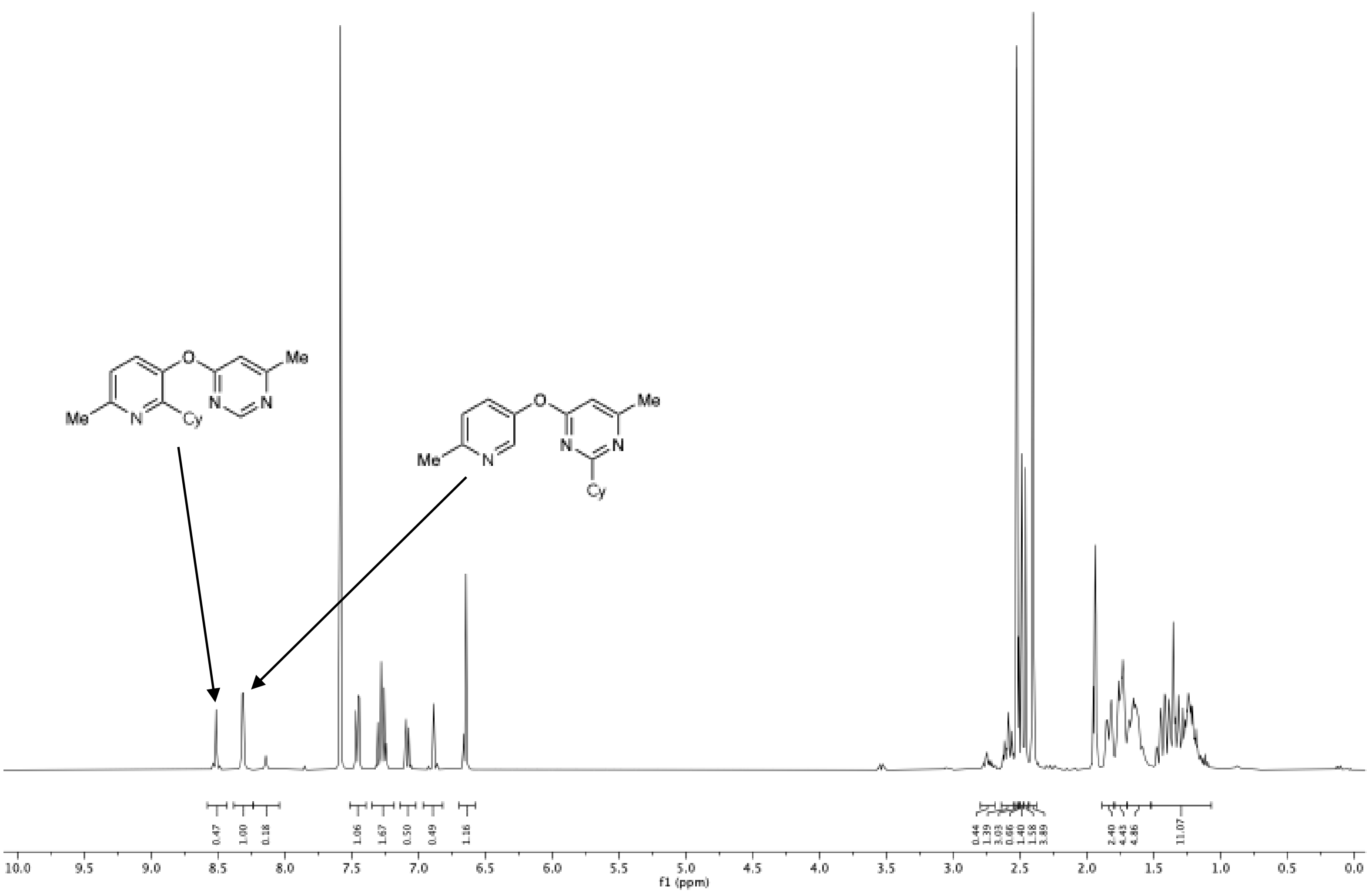

1H NMR (CDCl 400 MHz)

2 eq TFA

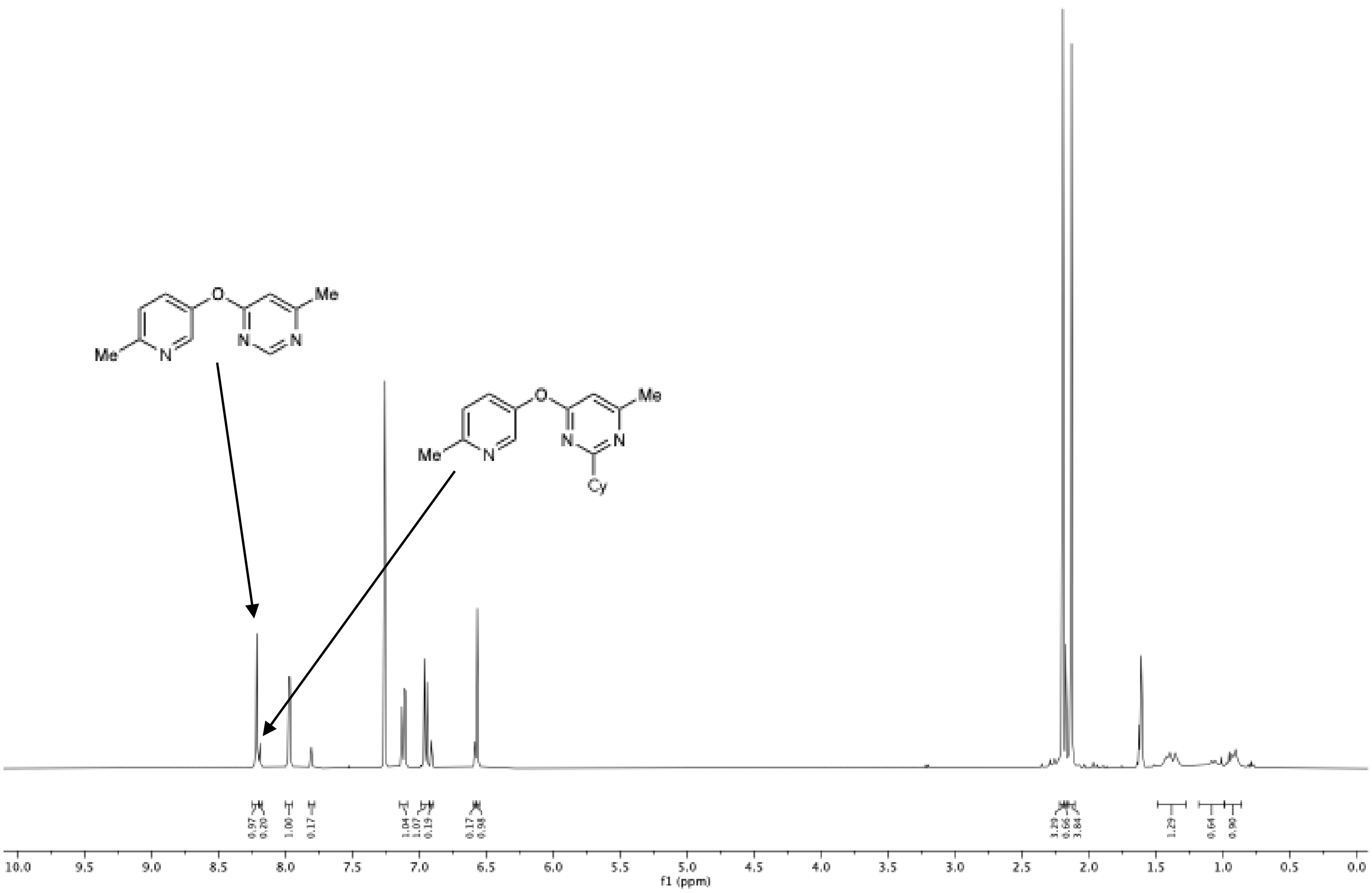

Table S9. Minisci alkylation example on ***3a*** C–H precursor. [a]

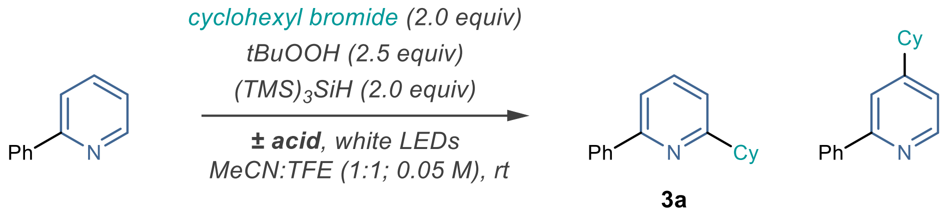

Entry	*Acid*	*SM Pyridine*	*C4-Alkylated*	***3a***
1	none	86	2	0
2	TFA (1 equiv)	56	26	16
3[b]	TFA (2 equiv)	43	23	11
4	TfOH (1 equiv)	34	0	0
5	TfOH (2 equiv)	36	0	0

[a]r.t.: Room temperature; SM: Starting material; n.d.: Not detected. [b]Isolated yields from 1 mmol scale reaction reported.

Crude, ^{1}H NMR ($CDCl_3$ 400 MHz)

2 eq TFA

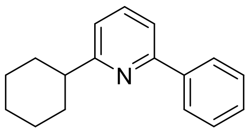

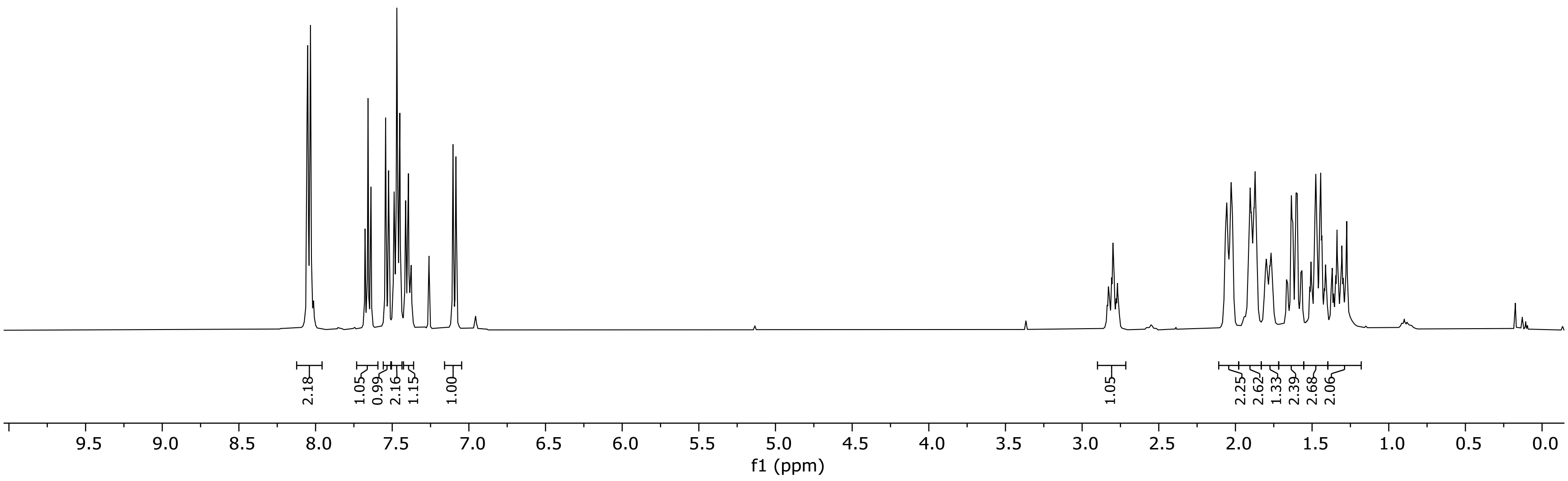

Crude, ^{1}H NMR ($CDCl_3$ 400 MHz)

2 eq TFA

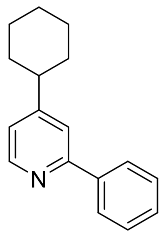

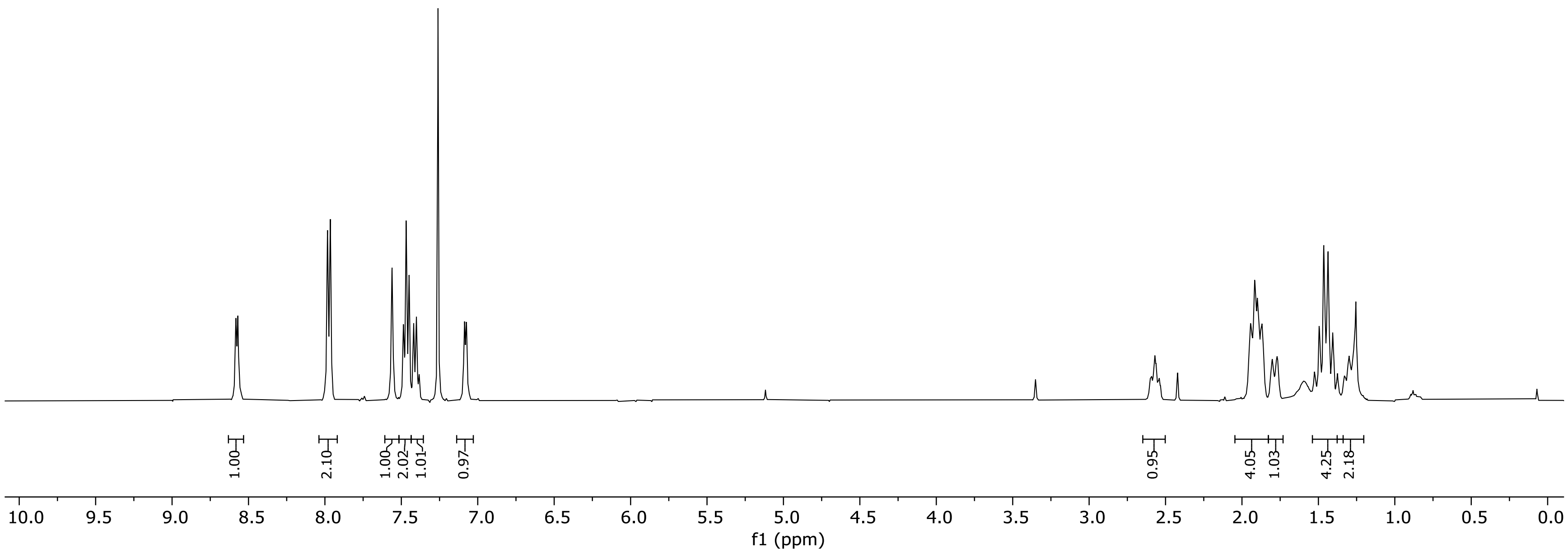

1.5 Preparation of Starting Materials

(1*S*,2*S*,4*R*)-2-Bromo-1-isopropyl-4-methylcyclohexane

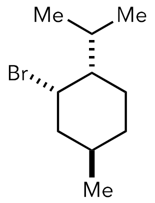

Prepared according to literature procedure.[9] An oven-dried round bottom flask equipped with a stir bar under an atmosphere of N_2 was charged with PPh_3 (5.12 g, 19.5 mmol) dissolved in CH_2Cl_2 (119 mL, 0.16 M) and the solution was cooled to 0 °C. Br_2 (1.00 mL, 19.5 mmol) was added to the reaction mixture and stirred for 30 minutes at 0 °C. Then, triethylamine (2.70 mL, 19.4 mmol) was added dropwise, followed by the addition of tosyl chloride (601 mg, 3.15 mmol) in one solid portion. The reaction was subjected to three rapid cycles of vacuum/nitrogen backfill and was stirred for 10 minutes at 0 °C. (–)-Menthol (2.34 g, 15.0 mmol) was added in one solid portion. The reaction was subjected to three rapid cycles of vacuum/nitrogen backfill, then warmed to room temperature, and stirred for 1 hour. The reaction was quenched with approximately 50.0 mL H_2O and extracted with EtOAc (3x). The organic layer was dried (Na_2SO_4), filtered, and concentrated *in vacuo*. The crude mixture was then subjected to vacuum distillation at 60 °C to remove reaction by-products, providing the pure product as a colorless oil (1.16 g, 5.29 mmol, 35% yield). ^{1}H NMR (400 MHz, $CDCl_3$) δ: 4.65 (dd, *J* = 3.1, 1.7 Hz, 1H), 2.15 (dtd, *J* = 14.3, 3.4, 2.2 Hz, 1H), 1.95 (dddt, *J* = 15.1, 11.9, 6.5, 3.3 Hz, 1H), 1.73 (dtt, *J* = 14.5, 3.6, 2.0 Hz, 2H), 1.58 – 1.29 (m, 3H), 0.97 – 0.82 (m, 10H), 0.77 (ddt, *J* = 12.1, 9.3, 3.0 Hz, 1H); ^{13}C NMR (100 MHz, $CDCl_3$) δ: 60.68, 49.37, 44.05, 34.97, 31.51, 26.91, 25.20, 21.92, 20.79, 20.22.

2-Methyl-3-((3-methylpyridin-2-yl)oxy)pyridine (4)

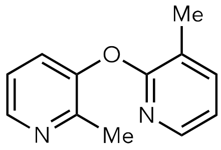

To an oven-dried round bottom flask equipped with a stir bar was added 2-methylpyridin-3-ol (2.18 g, 20.0 mmol), Co(acac)$_2$ (514 mg, 2.00 mmol), CuI (381 mg, 2.00 mmol), Cs_2CO_3 (13.0 g, 40.0 mmol), and 2-bromo-3-methylpyridine (2.23 mL, 20.0 mmol). The reaction vessel was subjected to three rapid cycles of vacuum/nitrogen backfill before adding *N*-methyl-2-pyrrolidone (60.0 mL, 0.30 M). The reaction vessel was heated to 110 °C with stirring for 18 hours. After cooling to room temperature, the reaction was diluted in water and extracted with EtOAc (3x). The combined organic extracts were then washed with water (5x) and dried (Na_2SO_4), filtered, and concentrated *in vacuo*. The crude material was purified by a flash

chromatography column (silica gel: 50% EtOAc in hexanes) to provide the title compound as a green solid (3.32 g, 16.6 mmol, 83% yield). mp: 26–27 °C; ^{1}H NMR (400 MHz, $CDCl_3$) δ: 8.38 (*br* s, 1H), 7.87 (dd, *J* = 5.0, 1.9 Hz, 1H), 7.47 (ddd, *J* = 7.3, 1.9, 1.0 Hz, 1H), 7.35 (d, *J* = 8.0 Hz, 1H), 7.15 (*br* s, 1H), 6.85 (dd, *J* = 7.2, 4.9 Hz, 1H), 2.37 (s, 3H), 2.33 (s, 3H); ^{13}C NMR (100 MHz, $CDCl_3$) δ: 161.04, 151.94, 149.28, 144.84, 144.46, 139.82, 129.04, 122.14, 121.25, 118.70, 19.62, 15.85; IR υ_{max}/cm^{-1} (film): 3061, 2925, 230, 2341, 1586, 1570, 1444, 1410, 1294, 1244, 1166, 1116, 896, 787, 750, 718; m/z LRMS (ESI + APCI): $[M+H]^+$ calculated for $C_{12}H_{13}N_2O^+$ = 201.1, found 201.1.

2-(2-(Pyridin-2-yl)ethoxy)-6-(trifluoromethyl)pyridine

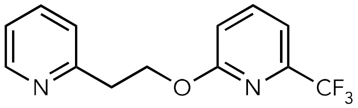

To an oven-dried round bottom flask equipped with a stir bar was added NaH (60 % in oil; 1.01 g, 30.0 mmol). The reaction vessel was subjected to three rapid cycles of vacuum/nitrogen backfill before adding THF (33.0 mL, 0.90 M) and cooling the reaction vessel to 0 °C. To this suspension was added 2-(pyridin-2-yl)ethan-1-ol (1.13 mL, 10.0 mmol) dropwise as a 4.00 M solution in THF and the reaction was allowed to warm to room temperature over 30 minutes with stirring. The reaction was then cooled to 0 °C and 2-chloro-6-(trifluoromethyl)pyridine (1.82 g, 10.0 mmol) was added dropwise as a 4.00 M solution in THF with stirring. The reaction was allowed to warm to room temperature and was stirred for 2 hours. The reaction was then quenched by slowly pouring over ice. The aqueous layer was extracted with EtOAc (3x). The combined organic extracts were then washed with water (3x), dried (Na_2SO_4), filtered, and concentrated *in vacuo*. The crude material was purified by a flash chromatography column (silica gel: 10 to 25% EtOAc in hexanes) to provide the title compound as a yellow liquid (388 mg, 1.45 mmol, 14% yield). ^{1}H NMR (400 MHz, $CDCl_3$) δ: 8.56 (dd, *J* = 4.9, 1.0 Hz, 1H), 7.66 (ddd, *J* = 8.2, 7.3, 0.8 Hz, 1H), 7.61 (td, *J* = 7.7, 1.9 Hz, 1H), 7.25 (d, *J* = 7.8 Hz, 1H), 7.22 (d, *J* = 7.2 Hz, 1H), 7.14 (ddd, *J* = 7.6, 4.9, 1.2 Hz, 1H), 6.86 (dt, *J* = 8.5, 0.7 Hz, 1H), 4.74 (t, *J* = 6.8 Hz, 2H), 3.28 (t, *J* = 6.8 Hz, 2H); ^{13}C NMR (100 MHz, $CDCl_3$) δ: 163.77, 158.62, 149.51, 145.62 (q, *J* = 34.6 Hz), 139.42, 136.55, 123.69, 121.69, 121.49 (q, *J* = 273.8 Hz), 114.76, 113.31 (q, *J* = 3.2 Hz), 65.77, 37.75; ^{19}F NMR (376 MHz, $CDCl_3$) δ: -68.45; IR υ_{max}/cm^{-1} (film): 2360, 2341, 1606, 1592, 1577, 1456, 1347, 1284, 1182, 1134, 1010, 987, 809, 739; m/z LRMS (ESI + APCI): $[M+H]^+$ calculated for $C_{13}H_{12}F_3N_2O^+$ = 269.1, found 269.1.

2-Chloro-5-(((4-(pyridin-2-yl)benzyl)oxy)methyl)pyridine

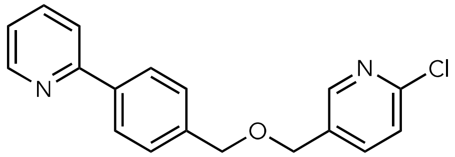

Prepared according to our previous report[5] using sodium hydride (60% in mineral oil, 2.63 g, 65.8 mmol), DMF (106 mL) and THF (318 mL), (4–(pyridin–2–yl)phenyl)methanol (5.89 g, 31.80 mmol in THF (20 mL)), and 2–chloro–5–(chloromethyl)pyridine (5.41 g, 33.40 mmol in DMF (20 mL)). The crude material was purified by flash chromatography (silica gel: 25% EtOAc in hexanes) to provide the title compound as a light yellow solid (6.91 g, 22.2 mmol, 70% yield). ^{1}H NMR (400 MHz, $CDCl_3$) δ: 8.69 (ddd, J = 4.8, 1.8, 1.1 Hz, 1H), 8.37 (dd, J = 2.4, 0.8 Hz, 1H), 8.00 (d, J = 8.3 Hz, 2H), 7.80 – 7.71 (m, 2H), 7.68 (dd, J = 8.2, 2.5 Hz, 1H), 7.46 (d, J = 8.5 Hz,2H), 7.32 (dd, J = 8.1, 0.7 Hz, 1H), 7.24 (ddd, J = 6.7, 4.8, 1.9 Hz, 1H), 4.64 (s, 2H), 4.55 (s, 2H); m/z LRMS (ESI + APCI): $[M+H]^+$ calculated for $C_{18}H_{16}ClN_2O^+$ = 311.1, found 311.1.

2-Chloro-5-(((6-methylpyridin-3-yl)oxy)methyl)pyridine

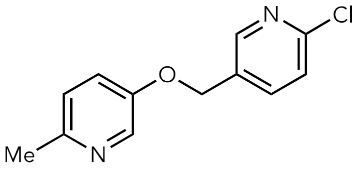

To an oven-dried round bottom flask equipped with a stir bar was added NaH (60 % in oil; 1.41 g, 42.0 mmol). The reaction vessel was subjected to three rapid cycles of vacuum/nitrogen backfill before adding DMF (67.0 mL, 0.63 M) and THF (200 mL, 0.16 M). The reaction vessel was then cooled to 0 °C. To this suspension was added 6-methylpyridin-3-ol (2.18 g, 20.0 mmol) dropwise as a 1.00 M solution in DMF and the reaction was allowed to warm to room temperature over 1 hour with stirring. The reaction was then cooled to 0 °C and 2-chloro-5-(chloromethyl)pyridine (3.40 g, 21.0 mmol) was added dropwise as a 1.00 M solution in DMF with stirring. The reaction was allowed to warm to room temperature and was stirred for 17 hours. The reaction was then quenched by adding water (200 mL) and the aqueous layer was extracted with EtOAc (3x). The combined organic extracts were then washed with water (3x), dried (Na_2SO_4), filtered, and concentrated *in vacuo*. The crude material was purified by a flash chromatography column (silica gel: 50 to 60% EtOAc in hexanes with 1% Et_3N) to provide the title compound as a yellow solid (1.41 g, 6.00 mmol, 30% yield). mp: 104–106 °C; ^{1}H NMR (400 MHz, $CDCl_3$) δ: 8.44 (dd, J = 2.5, 0.8 Hz, 1H), 8.24 (dd, J = 3.0, 0.7 Hz, 1H), 7.74 (dd, J = 8.2, 2.5 Hz, 1H), 7.36 (dd, J = 8.2, 0.7 Hz, 1H), 7.15 (dd, J = 8.5, 3.0 Hz, 1H), 7.08 (d, J = 8.4 Hz, 1H), 5.06 (s, 2H), 2.49 (s, 3H); ^{13}C NMR (100 MHz, $CDCl_3$) δ: 152.46, 151.61, 151.56, 148.90, 138.23, 136.93, 131.09, 124.49, 123.63, 122.71, 67.35, 23.54;

IR υ_{max}/cm^{-1} (film): 3024, 2923, 2360, 2341, 1569, 1495, 1458, 1403, 1270, 1250, 1207, 1107, 1053, 1024, 832, 752; m/z LRMS (ESI + APCI): [M+H]$^+$ calculated for $C_{12}H_{12}ClN_2O^+$ = 235.1, found 235.1.

2-((3-Fluoropyridin-2-yl)methoxy)-6-(trifluoromethyl)nicotinonitrile

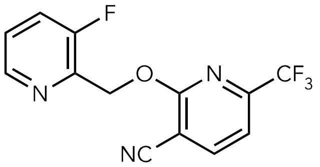

To an oven-dried round bottom flask equipped with a stir bar was added (3-fluoropyridin-2-yl)methanol (0.890 g, 7.00 mmol) and potassium *tert*-butoxide (0.864 g, 7.70 mmol). The reaction vessel was subjected to three rapid cycles of vacuum/nitrogen backfill before adding THF (18.0 mL, 0.40 M) at 0 °C. The reaction was stirred for 10 minutes at 0 °C before adding 2-chloro-6-(trifluoromethyl)nicotinonitrile (1.45 g, 7.00 mmol) dropwise as a 0.20 M solution in THF at –78 °C with stirring. The reaction was stirred at –78 °C for 30 minutes before being warmed to 0 °C for 3 hours with stirring. After warming to room temperature, the reaction was diluted in water and extracted with EtOAc (3x). The combined organic extracts were then washed with water (3x), dried (Na_2SO_4), filtered, and concentrated *in vacuo*. The crude material was purified by a flash chromatography column (silica gel: 40% EtOAc in hexanes) and heating the isolated residue to 50 °C on high vacuum to provide the title compound as a white solid (1.60 g, 5.38 mmol, 77% yield). mp: 93–95 °C; ^{1}H NMR (400 MHz, $CDCl_3$) δ: 8.42 (dt, *J* = 4.7, 1.4 Hz, 1H), 8.08 (dd, *J* = 7.7, 0.7 Hz, 1H), 7.46 (ddd, *J* = 9.6, 8.4, 1.4 Hz, 1H), 7.38 (d, *J* = 7.7 Hz, 1H), 7.33 (dt, *J* = 8.6, 4.4 Hz, 1H), 5.71 (d, *J* = 2.0 Hz, 2H); ^{13}C NMR (100 MHz, $CDCl_3$) δ: 163.25, 158.42 (d, *J* = 260.3 Hz), 149.07 (q, *J* = 36.3 Hz), 145.50 (d, *J* = 5.4 Hz), 144.91, 142.93 (d, *J* = 14.1 Hz), 125.35 (d, *J* = 4.0 Hz), 123.62 (d, *J* = 18.7 Hz), 120.45 (q, *J* = 274.8 Hz), 113.81, 113.53 (q, *J* = 3.0 Hz), 100.71, 65.39 (d, *J* = 1.6 Hz); ^{19}F NMR (376 MHz, $CDCl_3$) δ: -69.06, -124.26 (ddd, *J* = 9.5, 4.2, 2.0 Hz); IR υ_{max}/cm^{-1} (film): 2360, 2341, 2237, 1587, 1455, 1428, 1361, 1346, 1265, 1185, 1145, 1117, 1102, 988, 642, 804; m/z LRMS (ESI + APCI): [M+H]$^+$ calculated for $C_{13}H_8F_4N_3O^+$ = 298.1, found 298.1.

2-Chloro-5-fluoro-6-(1-(pyridin-2-yl)ethoxy)nicotinonitrile

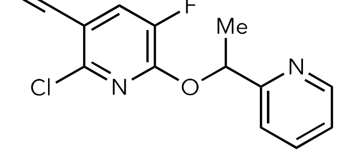

Prepared according to a modified literature procedure.[10] To an oven-dried round bottom flask equipped with a stir bar was added 1-(pyridin-2-yl)ethan-1-ol (1.12 mL, 10.0 mmol) and potassium *tert*-butoxide

(1.23 g, 11.0 mmol). The reaction vessel was subjected to three rapid cycles of vacuum/nitrogen backfill before adding THF (25.0 mL, 0.40 M) at 0 °C. The reaction was stirred for 10 minutes at 0 °C before adding 2,6-dichloro-5-fluoronicotinonitrile (1.91 g, 10.0 mmol) dropwise as a 0.20 M solution in THF at –78 °C with stirring. The reaction was stirred at –78 °C for 30 minutes before being warmed to 0 °C for 3 hours with stirring. After warming to room temperature, the reaction was diluted in water and extracted with CH_2Cl_2 (3x). The combined organic extracts were then washed with water (3x), dried (Na_2SO_4), filtered, and concentrated *in vacuo*. The crude material was purified by a flash chromatography column (silica gel: 5 to 15% EtOAc in hexanes) to provide the title compound as a white solid (1.85 g, 6.66 mmol, 67% yield). mp: 90–92 °C; ^{1}H NMR (400 MHz, $CDCl_3$) δ: 8.58 (ddd, *J* = 4.8, 1.8, 1.0 Hz, 1H), 7.69 (td, *J* = 7.7, 1.9 Hz, 1H), 7.57 (d, *J* = 8.4 Hz, 1H), 7.43 (dt, *J* = 7.8, 1.1 Hz, 1H), 7.21 (ddd, *J* = 7.6, 4.9, 1.2 Hz, 1H), 6.30 (q, *J* = 6.6 Hz, 1H), 1.76 (d, *J* = 6.6 Hz, 3H); ^{13}C NMR (100 MHz, $CDCl_3$) δ: 159.42, 154.20 (d, *J* = 12.7 Hz), 149.52, 145.63 (d, *J* = 3.9 Hz), 145.56 (d, *J* = 263.1 Hz), 136.99, 128.07 (d, *J* = 19.9 Hz), 123.11, 120.64, 114.28 (d, *J* = 1.2 Hz), 102.28 (d, *J* = 3.2 Hz), 77.34, 20.91; ^{19}F NMR (376 MHz, $CDCl_3$) δ: -138.33 (d, *J* = 8.5 Hz); IR υ_{max}/cm^{-1} (film): 2987, 2360, 2341, 2235, 1607, 1589, 1563, 1456, 1433, 1326, 1221, 1165, 1064, 1046, 1008, 903, 844, 745; m/z LRMS (ESI + APCI): $[M+H]^+$ calculated for $C_{13}H_{10}ClFN_3O^+$ = 278.0, found 278.1.

4-Methyl-6-((6-methylpyridin-3-yl)o

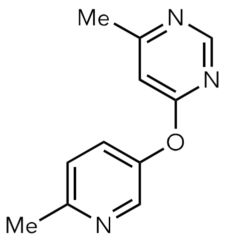

To an oven-dried round bottom flask equipped with a stir bar was added 4-chloro-6-methylpyrimidine (1.29 g, 10.0 mmol), 6-methylpyridin-3-ol (1.20 g, 11.0 mmol), K_2CO_3 (1.52 g, 11.0 mmol), and dimethylacetamide (14.0 mL, 0.71 M). The reaction was heated to 85 °C for 18 hours. After cooling to room temperature, the reaction was diluted in water and extracted with EtOAc (3x). The combined organic extracts were then washed with water (5x), dried (Na_2SO_4), filtered, and concentrated *in vacuo*. The crude material was purified by a flash chromatography column (silica gel: 50 to 100% EtOAc in hexanes) to provide the title compound as a yellow solid (743 mg, 3.70 mmol, 37% yield). mp: 69–71 °C; ^{1}H NMR (400 MHz, $CDCl_3$) δ: 8.61 (d, *J* = 1.1 Hz, 1H), 8.34 (d, *J* = 2.8 Hz, 1H), 7.38 (dd, *J* = 8.4, 2.8 Hz, 1H), 7.19 (d, *J* = 8.4 Hz, 1H), 6.77 (s, 1H), 2.55 (s, 3H), 2.49 (s, 3H); ^{13}C NMR (100 MHz, $CDCl_3$) δ: 169.35, 169.32, 158.08, 155.75, 146.92, 142.78, 129.69, 123.75, 107.20, 24.13, 23.95; IR υ_{max}/cm^{-1} (film): 2360, 2341,

1602, 1588, 1577, 1552, 1484, 1458, 1383, 1360, 1271, 1212, 1150, 1025, 951; m/z LRMS (ESI + APCI): $[M+H]^+$ calculated for $C_{11}H_{12}N_3O^+$ = 202.1, found 202.1.

5-(4-(2-(5-Ethylpyridin-2-yl)ethoxy)benzyl)-3-(pyridin-2-ylmethyl)thiazolidine-2,4-dione

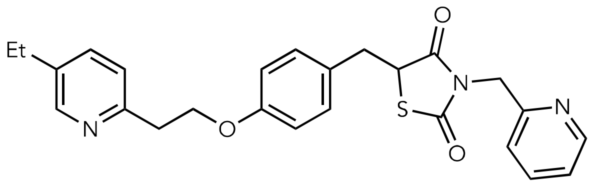

To an oven-dried round bottom flask equipped with a stir bar was added NaH (60 % in oil; 1.08 g, 32.0 mmol). The reaction vessel was subjected to three rapid cycles of vacuum/nitrogen backfill before adding DMF (100 mL, 0.32 M). The reaction vessel was then cooled to 0 °C. To this suspension was added 2-(2-(4-((2,4-dioxothiazolidin-5-yl)methyl)phenoxy)ethyl)-5-ethylpyridin-1-ium chloride (3.93 g, 10.0 mmol) in one solid portion and the reaction was allowed to warm to room temperature over 15 minutes with stirring. The reaction was then cooled to 0 °C and 2-(bromomethyl)pyridin-1-ium bromide (2.78 g, 11.0 mmol) was added in one solid portion with stirring. The reaction was allowed to warm to room temperature and stirred for 17 hours. The reaction was then quenched by slowly pouring over ice and stirring for 30 minutes, after which time a white precipitate formed. The precipitate was filtered off and washed with copious amounts of water and EtOAc. The aqueous layer was extracted with EtOAc (3x). The combined organic extracts were then washed with water (3x), dried (Na_2SO_4), filtered, and concentrated *in vacuo*. The crude material was purified by a flash chromatography column (silica gel: 50 to 60% EtOAc in hexanes) to provide the title compound as a white solid (1.62 g, 3.62 mmol, 36% yield). mp: 97–98 °C; ^1H NMR (400 MHz, CD_3CN with $CDCl_3$) δ: 8.43 (dd, *J* = 5.0, 1.3 Hz, 1H), 8.37 (d, *J* = 2.3 Hz, 1H), 7.61 (tt, *J* = 7.7, 1.6 Hz, 1H), 7.52 (dt, *J* = 8.0, 1.8 Hz, 1H), 7.22 (d, *J* = 7.9 Hz, 1H), 7.19 (dd, *J* = 7.6, 4.8 Hz, 1H), 7.13 (d, *J* = 8.7 Hz, 2H), 6.97 (d, *J* = 7.8 Hz, 1H), 6.80 (d, *J* = 8.7 Hz, 2H), 4.76 (t, *J* = 1.7 Hz, 2H), 4.68 (ddd, *J* = 8.5, 4.4, 1.4 Hz, 1H), 4.33 (td, *J* = 6.5, 1.4 Hz, 2H), 3.38 (dd, *J* = 14.3, 4.3 Hz, 1H), 3.17 (dd, *J* = 7.3, 6.0 Hz, 2H), 2.62 (q, *J* = 7.6 Hz, 2H), 2.16 (s, 1H), 1.21 (t, *J* = 7.6 Hz, 3H); ^{13}C NMR (100 MHz, CD_3CN with $CDCl_3$) δ: 174.86, 172.23, 159.25, 156.89, 155.40, 150.35, 149.94, 138.25, 137.81, 136.77, 131.80, 129.15, 124.23, 123.68, 122.23, 115.49, 68.14, 52.52, 46.86, 38.24, 37.84, 26.40, 16.00; IR υ_{max}/cm^{-1} (film): 2965, 2930, 2360, 2341, 1750, 1679, 1151, 1418, 1379, 1328, 1244, 1154, 1028, 824, 748; m/z LRMS (ESI + APCI): $[M+H]^+$ calculated for $C_{25}H_{26}N_3O_3S^+$ = 448.2, found 448.2.

Ethyl 4-(8-(pyridin-2-yl)-5,6-dihydro-11*H*-benzo [5,6] cyclohepta[1,2-*b*]pyridin-11-ylidene) piperidine-1-carboxylate (6)

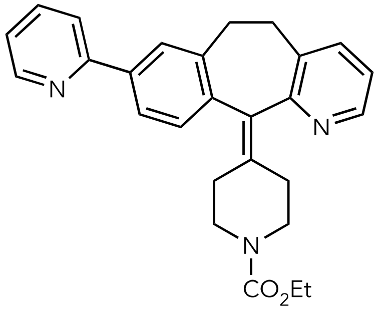

Prepared according to our previous report[5] using loratadine (ethyl4–(8–chloro–5,6–dihydro–11H–benzo[5,6]cyclohepta[1,2–b]pyridin–11–ylidene)piperidine–1–carboxylate) (3.83 g, 10.0 mmol), $Pd_2(dba)_3$ (916 mg, 1.00 mmol), tri–tert–butylphosphonium tetrafluoroborate (580 mg, 2.00 mmol), and cesium fluoride (3.34 g, 22.0 mmol), 1,4–dioxane (83.0 mL, 0.12 M). The title compound was isolated as a pale–yellow solid (3.39 g, 8.00 mmol, 80% yield). ^{1}H NMR (400 MHz, $CDCl_3$) δ: 8.66 (d, *J* = 4.2 Hz, 1H), 8.40 (dd, *J* = 4.8, 1.6 Hz, 1H), 7.87 (d, *J* = 1.9 Hz, 1H), 7.75 – 7.62 (m, 3H), 7.44 (dd, *J* = 7.7, 1.7 Hz, 1H), 7.30 (d, *J* = 8.0 Hz, 1H), 7.20 (ddd, *J* = 6.9, 4.8, 1.5 Hz, 1H), 7.09 (dd, *J* = 7.7, 4.8 Hz, 1H), 4.13 (q, *J* = 7.1 Hz, 2H), 3.81 (d, *J* = 17.8 Hz, 2H), 3.50 (ddd, *J* = 15.8, 10.0, 4.6 Hz, 1H), 3.39 (ddd, *J* = 15.7, 8.3, 4.6 Hz, 1H), 3.14 (ddd, *J* = 13.1, 7.4, 5.6 Hz, 2H), 2.93 (tq, *J* = 14.7, 4.7 Hz, 2H), 2.51 (ddd, *J* = 14.1, 9.5, 4.6 Hz, 1H), 2.44 – 2.29 (m, 3H), 1.25 (t, *J* = 7.1 Hz, 3H); m/z LRMS (ESI + APCI): $[M+H]^+$ calculated for $C_{27}H_{28}N_3O_2^+$ = 426.2, found 426.3.

The spectroscopic data matches the previously reported synthesis.

4-(Diphenylphosphaneyl)-2,6-dimethylpyridine

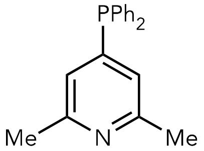

Prepared according to our previous report[11] using 4-chloro-2,6-dimethylpyridine (382 μL, 3.00 mmol), diphenylphosphine (626 μL, 3.60 mmol), TfOH (265 μL, 3.00 mmol), and chlorobenzene (1.50 mL, 2.00 M) . The title compound was isolated as a colorless oil (0.857 g, 2.94 mmol, 98% yield). ^{1}H NMR (400 MHz, $CDCl_3$) δ: 7.36 (m, 10H), 6.79 (d, *J* = 7.1 Hz, 2H), 2.45 (s, 6H); ^{31}P NMR (162 MHz, $CDCl_3$) δ: -7.01; m/z LRMS (ESI + APCI): $[M+H]^+$ calculated for $C_{19}H_{19}NP^+$ = 292.1, found 292.2.

1.6 Preparation of Heterocyclic Phosphonium Salts

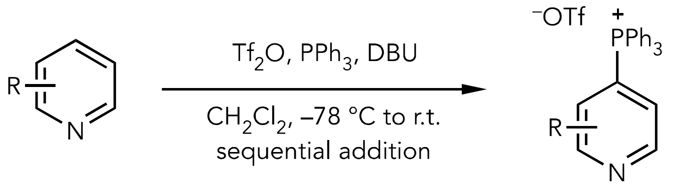

General Procedure A

An oven-dried 8 mL vial (≤ 0.50 mmol scale) or a round bottom flask (> 0.50 mmol scale) equipped with a stir bar was charged with the heterocycle (1.00 equiv) and PPh_3 (1.10 equiv) before being placed under a nitrogen atmosphere. CH_2Cl_2 (0.10 M) was added, the reaction vessel cooled to –78 °C, and Tf_2O (1.00 equiv) was added dropwise over 5 minutes. The reaction was stirred for 30 minutes at –78 °C before the stated organic base (Et_3N or DBU, 1.00 equiv) was added dropwise via syringe. The cooling bath was removed, and the reaction was allowed to warm to room temperature while stirring (approximately 15-30 minutes). The reaction mixture was quenched with H_2O (approximately the same volume as CH_2Cl_2) and the mixture was transferred to a separatory funnel. The mixture was diluted with CH_2Cl_2 and the resulting organic layer was washed three times with H_2O. The organic layer was dried (Na_2SO_4), filtered, and concentrated *in vacuo*. Approximately 2-10 mL (depending on the scale of the reaction) of CH_2Cl_2 was added to the residue and this was added dropwise to an excess of chilled Et_2O (0 °C). The flask was then placed in a –20 °C refrigerator for approximately 1 hour. The resulting suspension was filtered on a frit, the solid washed with chilled Et_2O (0 °C), and dried *in vacuo* to provide the pure phosphonium salt.

General Procedure B

An oven-dried 8 mL vial (≤ 0.50 mmol scale) or a round bottom flask (> 0.50 mmol scale) equipped with a stir bar was charged with the heterocycle (1.00 equiv) and placed under a nitrogen atmosphere. CH_2Cl_2 (0.10 M) was added, the reaction vessel cooled to –78 °C, and Tf_2O (1.00 equiv) was added dropwise over 5 minutes. The reaction was stirred for 30 minutes before PPh_3 (1.10 equiv) was added in one solid portion. The reaction was subjected to three rapid cycles of vacuum/nitrogen backfill and was stirred for a further 30 minutes at –78 °C. The stated organic base (Et_3N or DBU, 1.00 equiv) was added dropwise via syringe, the cooling bath was removed, and the reaction was allowed to warm to room temperature while stirring (approximately 15-30 minutes). The reaction mixture was quenched with H_2O (approximately the same volume as CH_2Cl_2) and the mixture was transferred to a separatory funnel. The mixture was diluted with CH_2Cl_2 and the resulting organic layer was washed three times with H_2O. The organic layer was dried (Na_2SO_4), filtered, and concentrated *in vacuo*. Approximately 2-10 mL (depending on the scale of the

reaction) of CH_2Cl_2 was added to the residue and this was added dropwise to an excess of chilled Et_2O (0 °C). The flask was then placed in a –20 °C refrigerator for approximately 1 hour. The resulting suspension was filtered on a frit, the solid washed with chilled Et_2O (0 °C), and dried *in vacuo* to provide the pure phosphonium salt.

General Procedure C

An oven-dried 8 mL vial (≤ 0.50 mmol scale) or a round bottom flask (> 0.50 mmol scale) equipped with a stir bar was charged with the heterocycle (1.00 equiv) and placed under a nitrogen atmosphere. CH_2Cl_2 (0.10 M) was added, the reaction vessel cooled to –78 °C, and Tf_2O (2.00 equiv) was added dropwise over 5 minutes. The reaction was stirred for 30 minutes before PPh_3 (2.00 equiv) was added in one solid portion. The reaction was subjected to three rapid cycles of vacuum/nitrogen backfill and was stirred for a further 30 minutes at –78 °C. Et_3N (2.00 equiv) was added dropwise via syringe, the cooling bath was removed, and the reaction was allowed to warm to room temperature while stirring (approximately 15-30 minutes). The reaction mixture was quenched with H_2O (approximately the same volume as CH_2Cl_2) and the mixture was transferred to a separatory funnel. The mixture was diluted with CH_2Cl_2 and the resulting organic layer was washed three times with H_2O. The organic layer was dried (Na_2SO_4), filtered, and concentrated *in vacuo*. Approximately 2-10 mL (depending on the scale of the reaction) of CH_2Cl_2 was added to the residue and this was added dropwise to an excess of chilled Et_2O (0 °C). The flask was then placed in a –20 °C refrigerator for approximately 1 hour. The resulting suspension was filtered on a frit, the solid washed with chilled Et_2O (0 °C), and dried *in vacuo* to provide the pure phosphonium salt.

General Procedure D

An oven dried 8 mL vial (< 0.5 mmol scale) or a round bottom flask (> 0.5 mmol scale) equipped with a stir bar was charged with the heterocycle (1.00 equiv) and placed under a nitrogen atmosphere. CH_2Cl_2 (0.10 M) was added, the reaction vessel cooled to –78 ºC, and Tf_2O (1.00 equiv) was added dropwise over 5 minutes. The reaction was stirred for 30 minutes before PPh_3 (1.10 equiv) was added in one portion. The reaction was subjected to three rapid cycles of vacuum/nitrogen backfill and was stirred for a further 30 min at –78 ºC. NEt_3 (1.00 equiv) was added dropwise via syringe, the reaction was stirred at –50 ºC for 2 hours, then the cooling bath was removed, and the reaction was allowed to warm to room temperature while stirring (approximately 30 minutes). The reaction was quenched with H_2O (approximately the same volume of CH_2Cl_2) and the mixture was transferred to a separatory funnel. The mixture was diluted with CH_2Cl_2, and the resulting organic layer was dried (Na_2SO_4), filtered, and concentrated *in vacuo*. Approximately 2-10 mL (depending on reaction scale) was added to the residue, and this was added dropwise to an excess

of chilled Et_2O (0 ºC). The flask was then placed in a –20 ºC refrigerator for approximately 1 hour. The resulting suspension was filtered on a frit, the solid washed with chilled Et_2O (0 ºC), and dried *in vacuo* to provide the pure phosphonium salt.

Reaction Notes

1) PPh_3 was crushed into a powder prior to use.
2) In a small number of cases, residual CH_2Cl_2 or Et_2O can become trapped in the phosphonium salt products. In these cases, heating the salts under vacuum (50 – 100 °C) can help to remove the residual solvent.

Triphenyl(2-phenylpyridin-4-yl)phosphonium trifluoromethanesulfonate (1a)

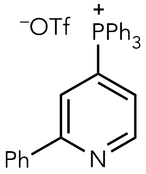

Prepared according to our previous report[8] using 2-phenylpyridine (2.57 mL, 18.0 mmol), Tf_2O (3.04 mL, 18.0 mmol), PPh_3 (5.19 g, 19.8 mmol), Et_3N (2.51 mL, 18.0 mmol), and CH_2Cl_2 (180 mL, 0.10 M). The title compound was isolated as a white solid (8.53 g, 15.1 mmol, 84% yield). ^{1}H NMR (400 MHz, $CDCl_3$) δ: 9.08 (*app* t, J = 5.2 Hz, 1H), 8.00 – 7.87 (m, 5H), 7.87 – 7.74 (m, 7H), 7.70 (dd, J = 13.3, 7.8 Hz, 6H), 7.53 (dd, J = 12.8, 5.0 Hz, 1H), 7.46 (dd, J = 4.5, 2.5 Hz, 3H); ^{19}F NMR (376 MHz, $CDCl_3$) δ: -78.10; ^{31}P NMR (162 MHz, $CDCl_3$) δ: 22.83; m/z LRMS (ESI + APCI): [M−OTf]$^+$ calculated for $C_{29}H_{23}NP^+$ = 416.2 , found 416.2.

(2-Isopropylpyridin-4-yl)triphenylphosphonium trifluoromethanesulfonate (1t)

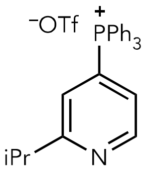

Prepared according to general procedure B using 2-isopropylpyridine (2.51 mL, 30.0 mmol), Tf_2O (5.05 mL, 30.0 mmol), PPh_3 (8.65 g, 33.0 mmol), DBU (4.48 mL, 30.0 mmol), and CH_2Cl_2 (150 mL, 0.2 M). After the purification procedure (three precipitations were required), the title compound was isolated as a salmon–colored solid (5.50 g, 10.3 mmol, 34% yield). mp: 124–126 °C; ^{1}H NMR (400 MHz, $CDCl_3$) δ: 8.92 (*app* td, J = 5.2, 0.9 Hz, 1H), 7.96 – 7.89 (m, 3H), 7.80 (td, J = 7.8, 3.7 Hz, 6H), 7.67 – 7.59 (m, 6H), 7.38 (ddd, J = 12.8, 5.1, 1.7 Hz, 1H), 7.27 (d, J = 13.9 Hz, 1H), 3.13 (hept, J = 6.9 Hz, 1H), 1.27 (d, J = 6.9 Hz, 6H); ^{13}C NMR (100 MHz, $CDCl_3$) δ: 170.08 (d, J = 9.3 Hz), 151.33 (d, J = 10.6 Hz), 136.36 (d, J = 3.1 Hz), 134.57 (d, J = 10.5 Hz), 131.15 (d, J = 13.0 Hz), 128.70 (d, J = 83.3 Hz), 124.70 (d, J = 8.1 Hz), 124.40 (d, J = 8.1 Hz), 120.96 (q, J = 321.0 Hz), 115.97 (d, J = 89.4 Hz), 36.63 (d, J = 1.4 Hz), 22.34; ^{19}F NMR (376 MHz, $CDCl_3$) δ: -78.11; ^{31}P NMR (162 MHz, $CDCl_3$) δ: 22.55; IR υ_{max}/cm^{-1} (film): 3011, 2971, 2360, 2342, 1576, 1438, 1259, 1223, 1155, 1107, 1028, 997, 749, 725, 635; m/z LRMS (ESI + APCI): [M−OTf]$^+$ calculated for $C_{26}H_{25}NP^+$ = 382.2, found 382.3.

(2-Methoxypyridin-4-yl)triphenylphosphonium trifluoromethanesulfonate (1u)

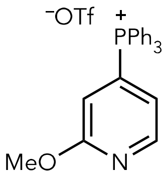

Prepared according to our previous report[9] using 2-methoxypyridine (52.5 µL, 0.50 mmol), PPh_3 (144 mg, 0.55 mmol), Tf_2O (84.0 µL, 0.50 mmol), DBU (75.0 µL, 0.50 mmol) and CH_2Cl_2 (5.00 mL, 0.10 M). The title compound was isolated as a white solid (174 mg, 0.34 mmol, 67% yield). ^{1}H NMR (400 MHz, $CDCl_3$) δ: 8.57 (*app* t, *J* = 5.4 Hz, 1H), 7.97 – 7.87 (m, 3H), 7.80 (td, *J* = 7.9, 3.8 Hz, 6H), 7.66 (ddd, *J* = 18.4, 1.3 Hz, 6H), 7.13 (ddd, *J* = 11.6, 5.2, 1.5 Hz, 1H), 6.84 (d, *J* = 14.3 Hz, 1H), 4.02 (s, 3H); ^{19}F NMR (376 MHz, $CDCl_3$) δ: -78.14; ^{31}P NMR (162 MHz, $CDCl_3$) δ: 22.39; m/z LRMS (ESI + APCI): $[M-OTf]^+$ calculated for $C_{24}H_{21}NOP^+$ = 370.1, found 370.2.

(2-Butyl-3-fluoropyridin-4-yl)triphenylphosphonium trifluoromethanesulfonate (1v)

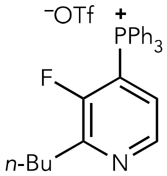

Prepared according to our previous report[10] using 2-butyl-3-fluoropyridine (498 mg, 3.25 mmol), Tf_2O (546 µL, 3.25 mmol), PPh_3 (938 mg, 3.58 mmol), Et_3N (455 µL, 3.25 mmol) and CH_2Cl_2 (32.5 mL, 0.10 M). The title compound was isolated as a white solid (1.28 g, 2.28 mmol, 70% yield). ^{1}H NMR (400 MHz, $CDCl_3$) δ: 8.83 – 8.49 (m, 1H), 7.98 – 7.88 (m, 3H), 7.80 (tt, *J* = 7.2, 2.9 Hz, 6H), 7.66 (dd, *J* = 13.9, 7.9 Hz, 6H), 7.12 (dt, *J* = 14.3, 4.9 Hz, 1H), 2.94 (t, *J* = 7.7 Hz, 2H), 1.72 (t, *J* = 11.1 Hz, 2H), 1.36 (h, *J* = 7.6 Hz, 2H), 0.92 (t, *J* = 7.4 Hz, 3H); ^{19}F NMR (376 MHz, $CDCl_3$) δ: -78.16, -108.90; ^{31}P NMR (162 MHz, $CDCl_3$) δ: 20.58 (d, *J* = 4.1 Hz); m/z LRMS (ESI + APCI): $[M-OTf]^+$ calculated for $C_{27}H_{26}FNP^+$ = 414.2, found 414.3.

(2-Butyl-5-(methoxymethyl)pyridin-4-yl)triphenylphosphonium trifluoromethanesulfonate (1w)

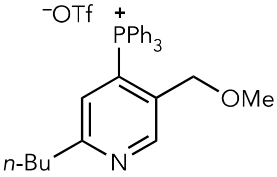

Prepared according to our previous report[10] using 2-butyl-5-(methoxymethyl)pyridine (897 mg, 5.00 mmol), Tf_2O (840 µL, 5.00 mmol), PPh_3 (1.44 g, 5.50 mmol), DBU (750 µL, 5.00 mmol), and CH_2Cl_2 (50.0

mL, 0.10 M). The title compound was isolated as a light yellow solid (2.51 g, 4.25 mmol, 85% yield). ^1H NMR (400 MHz, $CDCl_3$) δ: 8.93 (*app* d, *J* = 6.7 Hz, 1H), 7.91 – 7.82 (m, 3H), 7.78 (ddd, *J* = 9.3, 7.1, 3.8 Hz, 6H), 7.68 – 7.61 (m, 6H), 6.89 (d, *J* = 16.1 Hz, 1H), 4.07 (s, 2H), 2.86 – 2.81 (m, 2H), 2.54 (s, 3H), 1.62 – 1.50 (m, 2H), 1.25 (hept, *J* = 7.4 Hz, 2H), 0.83 (t, *J* = 7.3 Hz, 3H); ^{19}F NMR (376 MHz, $CDCl_3$) δ: -78.10; ^{31}P NMR (162 MHz, $CDCl_3$) δ: 24.92; m/z LRMS (ESI + APCI): $[M{-}OTf]^+$ calculated for $C_{29}H_{31}NOP^+$ = 440.2, found 440.3.

Triphenyl(3-phenylpyridin-4-yl)phosphonium trifluoromethanesulfonate (1x)

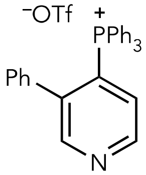

Prepared according to our previous report[8] using 3-phenylpyridine (400 mg, 2.58 mmol), Tf_2O (435 µL, 2.58 mmol), PPh_3 (744 mg, 2.84 mmol), DBU (285 µL, 2.58 mmol) and CH_2Cl_2 (26.0 mL, 0.10 M). The title compound was isolated as a white solid (1.05 g, 1.86 mmol, 72% yield). ^1H NMR (400 MHz, $CDCl_3$) δ: 8.98 (*app* t, *J* = 4.8 Hz, 1H), 8.76 (d, *J* = 6.8 Hz, 1H), 7.80 (dd, *J* = 8.5, 6.4 Hz, 3H), 7.67 (td, *J* = 7.7, 3.7 Hz, 6H), 7.60 (dd, *J* = 13.0, 7.9 Hz, 6H), 7.49 (dd, *J* = 15.1, 5.3 Hz, 1H), 7.12 (t, *J* = 7.5 Hz, 1H), 6.92 (t, *J* = 7.6 Hz, 2H), 6.73 (d, *J* = 7.6 Hz, 2H); ^{19}F NMR (376 MHz, $CDCl_3$) δ: -78.12; ^{31}P NMR (162 MHz, $CDCl_3$) δ: 21.52; m/z LRMS (ESI + APCI): $[M{-}OTf]^+$ calculated for $C_{29}H_{23}NP^+$ = 416.2, found 416.2.

Triphenyl(2-((3-phenylisoxazol-5-yl)methoxy)pyridin-4-yl)phosphonium trifluoromethanesulfonate (1y)

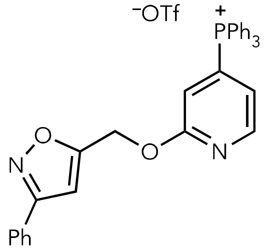

Prepared according to general procedure B using 3-phenyl-5-((pyridin-2-yloxy)methyl)isoxazole (995 mg, 3.94 mmol), Tf_2O (663 µL, 3.94 mmol), PPh_3 (1.14 g, 4.34 mmol), DBU (588 µL, 3.94 mmol), and CH_2Cl_2 (39.0 mL, 0.10 M). After the purification procedure, the title compound was isolated as a tan solid (1.45 g, 2.19 mmol, 56% yield). mp: 62–64 °C; ^1H NMR (400 MHz, $CDCl_3$) δ: 8.60 (*app* t, *J* = 5.3 Hz, 1H), 7.94 – 7.85 (m, 3H), 7.84 – 7.70 (m, 8H), 7.70 – 7.55 (m, 6H), 7.42 (dd, *J* = 4.9, 2.0 Hz, 3H), 7.22 (ddd, *J* = 11.6, 5.3, 1.5 Hz, 1H), 6.93 (d, *J* = 14.8 Hz, 1H), 6.78 (s, 1H), 5.58 (s, 2H); ^{13}C NMR (100 MHz, $CDCl_3$) δ:

167.39, 163.23 (d, J = 15.9 Hz), 162.77, 150.04 (d, J = 12.2 Hz), 136.36 (d, J = 3.2 Hz), 134.62 (d, J = 10.5 Hz), 131.15 (d, J = 13.2 Hz), 130.61 (d, J = 80.7 Hz), 130.25, 129.05, 128.72, 126.95, 121.01 (q, J = 321.2 Hz), 120.38 (d, J = 8.4 Hz), 116.54 (d, J = 10.2 Hz), 115.79 (d, J = 89.5 Hz), 102.89, 59.09; ^{19}F NMR (376 MHz, $CDCl_3$) δ: -78.08; ^{31}P NMR (162 MHz, $CDCl_3$) δ: 22.40; IR υ_{max}/cm^{-1} (film): 2924, 2850, 2359, 2342, 1595, 1574, 1440, 1278, 1252, 1047, 1000, 798, 766, 691; m/z LRMS (ESI + APCI): [M−OTf]$^+$ calculated for $C_{33}H_{26}N_2O_2P^+$ = 513.2, found 513.3.

(2-(3-Chloro-4-((2-fluorobenzyl)oxy)phenyl)pyridin-4-yl)triphenylphosphonium trifluoromethanesulfonate (1z)

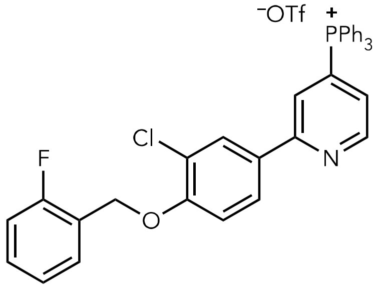

Prepared according to our previous report[11] using 2-(3-chloro-4-((2- fluorobenzyl)oxy)phenyl)pyridine (800.0 mg, 2.55 mmol), Tf_2O (430 μL, 2.55 mmol), PPh_3 (734 mg, 2.80 mmol), EtN_3 (360 μL, 2.55 mmol) and CH_2Cl_2 (25.5 mL, 0.10 M). The title compound was isolated as a white solid (1.09 g, 1.50 mmol, 59% yield). ^{1}H NMR (400 MHz, $CDCl_3$) δ: 9.02 (*app* t, J = 5.2 Hz, 1H), 8.03 (d, J = 2.0 Hz, 1H), 7.94 (t, J = 7.6 Hz, 3H), 7.85 – 7.75 (m, 8H), 7.74 – 7.66 (m, 6H), 7.57 (t, J = 7.6 Hz, 1H), 7.47 (dd, J = 12.7, 5.1 Hz, 1H), 7.32 (q, J = 7.1 Hz, 1H), 7.17 (t, J = 7.6 Hz, 1H), 7.13 – 7.04 (m, 2H), 5.26 (s, 2H); ^{19}F NMR (376 MHz, $CDCl_3$) δ: -78.13, -118.53 (dt, J = 12.8, 6.2 Hz); ^{31}P NMR (162 MHz, $CDCl_3$) δ: 22.85; m/z LRMS (ESI + APCI): [M−OTf]$^+$ calculated for $C_{36}H_{27}ClFNOP^+$ = 574.1, found 574.3.

(5-(Ethoxycarbonyl)-2-(4-(4,4,5,5-tetramethyl-1,3,2-dioxaborolan-2-yl)phenyl)pyridin-4-yl)triphenylphosphonium trifluoromethanesulfonate (1aa)

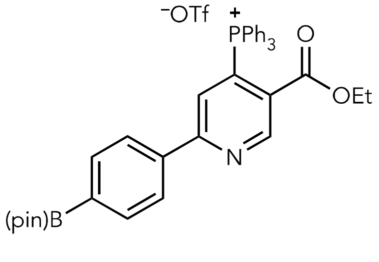

Prepared according to general procedure B using ethyl 6-(4-(4,4,5,5-tetramethyl-1,3,2-dioxaborolan-2-yl)phenyl)nicotinate (1.87 g, 5.29 mmol), Tf_2O (892 μL, 5.29 mmol), PPh_3 (1.52 g, 5.82 mmol), DBU

(584 μL, 5.29 mmol), and CH_2Cl_2 (30.0 mL, 0.30 M). After the purification procedure, the title compound was isolated as a white solid (1.18 g, 1.55 mmol, 30% yield). ^{1}H NMR (400 MHz, $CDCl_3$) δ: 9.61 (*app* d, *J* = 6.4 Hz, 1H), 7.86 (t, *J* = 8.7 Hz, 5H), 7.80 – 7.71 (m, 8H), 7.67 (dd, *J* = 13.3, 7.9 Hz, 6H), 7.56 (d, *J* = 16.4 Hz, 1H), 4.01 (q, *J* = 7.2 Hz, 2H), 1.34 (d, *J* = 1.6 Hz, 12H), 1.05 (t, *J* = 7.6 Hz, 3H); ^{19}F NMR (376 MHz, $CDCl_3$) δ: -78.14; ^{31}P NMR (162 MHz, $CDCl_3$) δ: 29.25; m/z LRMS (ESI + APCI): $[M{-}OTf]^+$ calculated for $C_{38}H_{38}BNO_4P^+$ = 614.3, found 614.4.

(*S*)-(3-(1-methylpyrrolidin-2-yl)pyridin-4-yl)triphenylphosphonium trifluoromethanesulfonate (1ab)

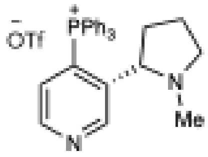

Prepared according to previously reported method[13] using (-)-nicotine (803 μL, 5.00 mmol), Tf_2O (840 μL, 5.00 mmol), PPh_3 (1.44 g, 5.50 mmol), DBU (0.75 mL, 5.00 mmol), and CH_2Cl_2 (50.0 mL, 0.1 M). After the purification procedure the title compound was isolated as a yellow solid (2.19 g, 3.82 mmol, 76% yield). Spectroscopic data matches previously reported example in literature[2]: ^{1}H NMR (400 MHz, $CDCl_3$) δ: 9.38 (d, *J* = 7.0 Hz, 1H), 8.80 (dd, *J* = 5.2, 4.1 Hz, 1H), 7.95 – 7.86 (m, 3H), 7.81 (td, *J* = 7.8, 3.9 Hz, 6H), 7.78 – 7.68 (m, 6H), 7.12 (dd, *J* = 15.6, 5.1 Hz, 1H), 3.08 – 2.96 (m, 2H), 1.99 (td, *J* = 9.5, 7.3 Hz, 1H), 1.80 (s, 3H), 1.79 – 1.69 (m, 1H), 1.49 – 1.37 (m, 1H), 1.30 (tdd, *J* = 11.6, 7.2, 4.5 Hz, 1H), 0.99 – 0.86 (m, 1H); ^{19}F NMR (376 MHz, $CDCl_3$) δ: -78.11; ^{31}P NMR (162 MHz, $CDCl_3$) δ: 20.73; m/z LRMS (ESI + APCI): $[M{-}OTf\ ]^+$ calculated for $C_{28}H_{28}N_2P^+$ = 423.3, found 423.3.

(2-((1-(4-Phenoxyphenoxy)propan-2-yl)oxy)pyridin-4-yl)triphenylphosphonium trifluoromethanesulfonate (1ac)

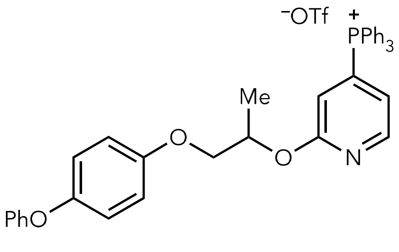

Prepared according to our previous report[9] using 2-((1-(4-phenoxyphenoxy)propan-2-yl)oxy)pyridine (1.61 g, 5.00 mmol), Tf_2O (841 μL, 5.00 mmol), PPh_3 (1.44 g, 5.50 mmol), DBU (746 μL, 5.00 mmol) and CH_2Cl_2 (50.0 mL, 0.10 M). The title compound was isolated as a white solid (1.71 g, 2.35 mmol, 47% yield). ^{1}H NMR (400 MHz, $CDCl_3$) δ: 8.52 (*app* t, *J* = 5.6 Hz, 1H), 7.87 (t, *J* = 7.7 Hz, 3H), 7.80 – 7.72 (m, 6H), 7.61 (dd, *J* = 13.2, 7.8 Hz, 6H), 7.30 – 7.18 (m, 2H), 7.11 (dd, *J* = 11.7, 5.3 Hz, 1H), 6.99 (t, *J* = 7.4 Hz, 1H),

6.93 – 6.82 (m, 6H), 6.78 (d, J = 15.0 Hz, 1H), 5.64 (q, J = 5.8 Hz, 1H), 4.10 (pd, J = 9.2, 4.4 Hz, 2H), 1.46 (dd, J = 6.5, 1.7 Hz, 3H); ^{19}F NMR (376 MHz, $CDCl_3$) δ: -78.08; ^{31}P NMR (162 MHz, $CDCl_3$) δ: 22.32; m/z LRMS (ESI + APCI): $[M-OTf]^+$ calculated for $C_{38}H_{33}NO_3P^+$ = 582.2, found 582.3.

triphenyl(quinolin-4-yl)phosphonium trifluoromethanesulfonate (1ad)

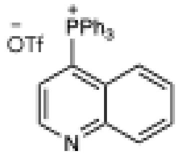

Prepared according to general procedure D using quinoline (1.18 mL, 10.0 mmol), Tf_2O (1.68 mL, 10.0 mmol), PPh_3 (2.89 g, 11.0 mmol), NEt_3 (1.39 mL, 10.0 mmol), and CH_2Cl_2 (100.0 mL, 0.1 M). After the purification procedure, column chromatography was required (5% acetone in CH_2Cl_2), and the title compound was isolated as a white solid (1.36 g, 2.50 mmol, 25% yield). Spectroscopic data matches previously reported example in literature[14]: ^{1}H NMR (400 MHz, $CDCl_3$) δ: 9.20 (t, J = 4.4 Hz, 1H), 8.37 (dt, J = 8.6, 1.7 Hz, 1H), 7.95 – 7.82 (m, 4H), 7.77 (td, J = 7.9, 3.9 Hz, 6H), 7.73 – 7.63 (m, 6H), 7.51 – 7.41 (m, 2H), 7.38 (d, J = 8.5 Hz, 1H); ^{19}F NMR (376 MHz, $CDCl_3$) δ: -78.13; ^{31}P NMR (162 MHz, $CDCl_3$) δ: 21.73; m/z LRMS (ESI + APCI): $[M-OTf]^+$ calculated for $C_{27}H_{21}NP^+$ = 390.1, found 390.1.

(6-Methoxyquinolin-4-yl)triphenylphosphonium trifluoromethanesulfonate (1ae)

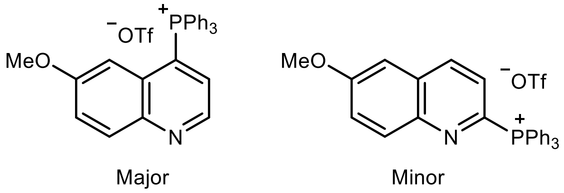

>20:1 Mixture of isomers

Prepared according to general procedure B using 6-methoxyquinoline (692 μL, 5.00 mmol), Tf_2O (841 μL, 5.00 mmol), PPh_3 (1.44 g, 5.50 mmol), DBU (746 μL, 5.00 mmol), and CH_2Cl_2 (50.0 mL, 0.10 M). After the purification procedure (two precipitations were required), the title compound was isolated as a pale–yellow solid (1.62 g, 2.84 mmol, 57% combined yield). mp: 174–177 °C; ^{1}H NMR (Major; 400 MHz, $CDCl_3$) δ: 8.99 (*app* t, J = 4.4 Hz, 1H), 8.20 (dd, J = 9.3, 2.0 Hz, 1H), 7.94 – 7.87 (m, 3H), 7.78 (td, J = 7.9, 3.9 Hz, 6H), 7.74 – 7.60 (m, 6H), 7.45 (dd, J = 9.3, 2.6 Hz, 1H), 7.36 (dd, J = 16.9, 4.5 Hz, 1H), 6.48 (d, J = 2.6 Hz, 1H), 3.23 (s, 3H); ^{13}C NMR (Major; 100 MHz, $CDCl_3$) δ: 159.57, 147.40 (d, J = 12.2 Hz), 145.23 (d, J = 7.0 Hz), 136.27 (d, J = 3.2 Hz), 134.52 (d, J = 10.5 Hz), 133.43 (d, J = 2.4 Hz), 131.31 (d, J = 13.1 Hz), 130.32 (d, J = 8.9 Hz), 127.96 (d, J = 5.9 Hz), 124.26, 122.38 (d, J = 83.1 Hz), 120.99 (q, J = 321.0 Hz), 116.53 (d, J = 89.0 Hz), 104.22 (d, J = 6.7 Hz), 55.69; ^{19}F NMR (Both isomers; 376 MHz,

$CDCl_3$) δ: -78.12; ^{31}P NMR (162 MHz, $CDCl_3$) δ: 21.57 (major), 14.30 (minor); IR $υ_{max}$/cm^{-1} (film): 3010, 2360, 2342, 1618, 1499, 1438, 1262, 1223, 1151, 1106, 1029, 991, 828, 745, 722, 635; m/z LRMS (ESI + APCI): [M−OTf]$^+$ calculated for $C_{28}H_{23}NOP^+$= 420.2, found 420.2.

Triphenyl(2-phenylfuro[2,3-*b*]pyridin-4-yl)phosphonium trifluoromethanesulfonate (1af)

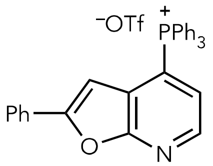

Prepared according to general procedure B using 2-phenylfuro[2,3-*b*]pyridine (1.95 g, 10.0 mmol), Tf_2O (1.68 mL, 10.0 mmol), PPh_3 (2.89 g, 11.0 mmol), DBU (1.49 mL, 10.0 mmol), and CH_2Cl_2 (100 mL, 0.10 M). After the purification procedure (two precipitations were required), the title compound was isolated as a pale–yellow solid (4.06 g, 6.70 mmol, 67% yield). mp: 262–264 °C; ^{1}H NMR (400 MHz, $CDCl_3$ with CD_3CN) δ: 8.47 (*app* t, *J* = 4.6 Hz, 1H), 7.88 (dt, *J* = 8.1, 4.5 Hz, 3H), 7.72 (td, *J* = 7.8, 3.8 Hz, 6H), 7.67 – 7.51 (m, 8H), 7.37 (dd, *J* = 5.4, 2.0 Hz, 3H), 7.18 (dd, *J* = 13.9, 5.8 Hz, 1H), 6.03 (s, 1H); ^{13}C NMR (100 MHz, $CDCl_3$) δ: 161.37 (d, *J* = 12.6 Hz), 159.87, 144.26 (d, *J* = 11.4 Hz), 136.10 (d, *J* = 3.2 Hz), 134.18 (d, *J* = 10.7 Hz), 131.09, 130.87 (d, *J* = 13.1 Hz), 129.02, 127.25, 125.77, 124.64 (d, *J* = 8.5 Hz), 124.30 (d, *J* = 8.7 Hz), 119.64 (q, *J* = 321.2 Hz), 118.54 (d, *J* = 88.0 Hz), 115.73 (d, *J* = 89.7 Hz), 98.68 (d, *J* = 2.4 Hz); ^{19}F NMR (376 MHz, $CDCl_3$) δ: -78.40 (d, *J* = 2.7 Hz); ^{31}P NMR (162 MHz, $CDCl_3$) δ: 19.85; IR $υ_{max}$/cm^{-1} (film): 3065, 3012, 2360, 2342, 1584, 1559, 1485, 1437, 1358, 1262, 1225, 1148, 1108, 1029, 751, 724, 667, 635; m/z LRMS (ESI + APCI): [M−OTf]$^+$ calculated for $C_{31}H_{23}NOP^+$ = 456.2, found 456.2.

(5-(4-Fluoro-1,3-dioxoisoindolin-2-yl)-2-methylpyridin-4-yl)triphenylphosphonium trifluoromethanesulfonate (1ag)

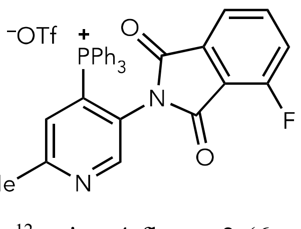

Prepared according to our previous report[12] using 4-fluoro-2-(6-methylpyridin-3-yl)isoindoline-1,3-dione (1.24 g, 4.57 mmol), Tf_2O (769 µL, 4.57 mmol), PPh_3 (1.32 g, 5.03 mmol), DBU (682 µL, 4.57 mmol) and CH_2Cl_2 (46.0 mL, 0.10 M). The title compound was isolated as a pale–tan solid (2.62 g, 3.93 mmol, 86% yield).^{1}H NMR (400 MHz, $CDCl_3$) δ: 8.66 (*app* d, *J* = 6.9 Hz, 1H), 7.82 (td, *J* = 8.0, 4.2 Hz, 1H), 7.73 – 7.54 (m, 15H), 7.54 (d, *J* = 15.0 Hz, 1H), 7.48 (d, *J* = 7.4 Hz, 1H), 7.42 (t, *J* = 8.5 Hz, 1H), 2.77 (s, 3H);

^{19}F NMR (376 MHz, $CDCl_3$) δ: -78.12, -110.64 (dd, *J* = 8.7, 4.4 Hz); ^{31}P NMR (162 MHz, $CDCl_3$) δ: 19.56; m/z LRMS (ESI + APCI): [M−OTf]$^+$ calculated for $C_{32}H_{23}FN_2O_2P^+$ = 517.1, found 517.2.

(2-(2-Chloro-5-(2-chloro-4-(methylsulfonyl)benzamido)phenyl)pyridin-4-yl)triphenylphosphonium trifluoromethanesulfonate (1ah)

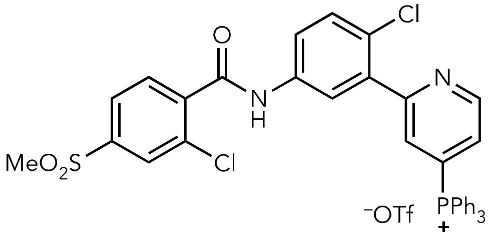

Prepared according to our previous report[12] using 2-chloro-*N*-(4-chloro-3-(pyridin-2-yl)phenyl)-4-(methylsulfonyl)benzamide (1.60 g, 3.80 mmol), Tf_2O (639 µL, 3.80 mmol), PPh_3 (1.10 g, 4.18 mmol), DBU (567 µL, 3.80 mmol) and CH_2Cl_2 (38.0 mL, 0.10 M). The title compound was isolated as a yellow solid (1.54 g, 1.86 mmol, 49% yield). ^1H NMR (400 MHz, CD_3CN) δ: 9.22 (s, 1H), 9.06 (*app* t, *J* = 5.1 Hz, 1H), 8.21 (d, *J* = 2.5 Hz, 1H), 8.04 (s, 1H), 7.96 (t, *J* = 7.1 Hz, 4H), 7.83 – 7.69 (m, 14H), 7.69 – 7.61 (m, 2H), 7.49 (dd, *J* = 8.7, 1.8 Hz, 1H), 3.13 (s, 3H); ^{19}F NMR (376 MHz, CD_3CN) δ: -79.31; ^{31}P NMR (162 MHz, CD_3CN) δ: 22.51; m/z LRMS (ESI + APCI): [M−OTf]$^+$ calculated for $C_{37}H_{28}Cl_2N_2O_3PS^+$ = 681.1, found 681.2.

(8-Chloro-11-(1-(ethoxycarbonyl)piperidin-4-ylidene)-6,11-dihydro-5*H*-benzo[5,6]cyclohepta[1,2-*b*]pyridin-4-yl)triphenylphosphonium trifluoromethanesulfonate (1ai)

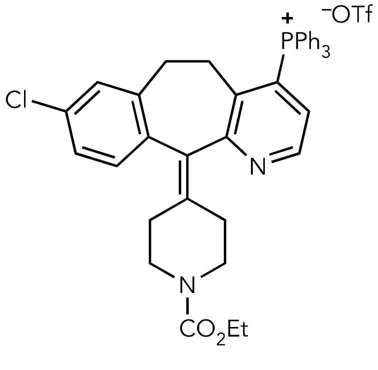

Prepared according to our previous report[8] using loratadine (ethyl-4-(8-chloro-5,6-dihydro-11H-benzo[5,6]cyclohepta[1,2-b]pyridine-11-ylidene)-1-piperidinecarboxylate) (1.92 g, 5.00 mmol), Tf_2O (841 µL, 5.00 mmol), PPh_3 (1.44 g, 5.50 mmol), DBU (746 µL, 5.00 mmol) and CH_2Cl_2 (50.0 mL, 0.10 M). The title compound was isolated as a white solid (2.85 g, 3.60 mmol, 72% yield). ^1H NMR (400 MHz, $CDCl_3$) δ: 8.74 (*app* t, *J* = 5.9 Hz, 1H), 7.93 (t, *J* = 7.7 Hz, 3H), 7.90 – 7.77 (m, 6H), 7.69 (dd, *J* = 13.3, 7.8 Hz,

6H), 7.20 – 6.94 (m, 3H), 6.72 (s, 1H), 4.15 (q, J = 7.1 Hz, 2H), 3.82 – 3.58 (m, 2H), 3.42 – 3.19 (m, 3H), 2.76 (d, J = 17.8 Hz, 1H), 2.59 (d, J = 15.2 Hz, 1H), 2.54 – 2.32 (m, 3H), 2.20 (s, 1H), 1.60 – 1.44 (m, 1H), 1.27 (td, J = 7.1, 2.0 Hz, 3H); ^{19}F NMR (376 MHz, $CDCl_3$) δ: -78.12; ^{31}P NMR (162 MHz, $CDCl_3$) δ: 21.24; m/z LRMS (ESI + APCI): $[M-OTf]^+$ calculated for $C_{40}H_{37}ClN_2O_2P^+$ = 643.2, found 643.3.

(*S*)-(2-((4-Chlorophenyl)((1-(ethoxycarbonyl)piperidin-4-yl)oxy)methyl)pyridin-4-yl)triphenylphosphonium trifluoromethanesulfonate (1aj)

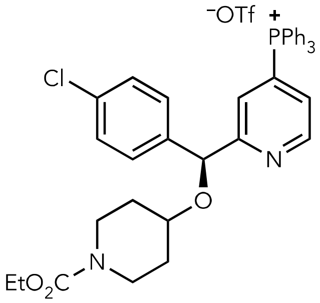

Prepared according to our previous report[9] using ethyl 4-((4-chlorophenyl)(pyridin-2-yl)methoxy)piperidine-1-carboxylate (889 mg, 2.37 mmol), Tf_2O (399 μL, 2.37 mmol), PPh_3 (684 mg, 2.61 mmol), DBU (384 μL, 2.37 mmol) and CH_2Cl_2 (23.7 mL, 0.10 M). The title compound was isolated as a tan solid (981.2 mg, 1.19 mmol, 50% yield). ^{1}H NMR (400 MHz, $CDCl_3$) δ: 8.91 (*app* t, J = 5.3 Hz, 1H), 7.95 – 7.87 (m, 3H), 7.78 (tdd, J = 7.7, 6.1, 2.9 Hz, 6H), 7.67 (dt, J = 12.9, 5.9 Hz, 7H), 7.49 (dd, J = 12.7, 5.1 Hz, 1H), 7.34 – 7.27 (m, 4H), 5.72 (s, 1H), 4.12 (dt, J = 8.1, 6.4 Hz, 2H), 3.66 (s, 1H), 3.55 – 3.44 (m, 2H), 3.19 (s, 2H), 1.72 (s, 1H), 1.64 (s, 1H), 1.47 (d, J = 11.5 Hz, 2H), 1.26 (td, J = 7.2, 1.8 Hz, 3H); ^{19}F NMR (376 MHz, $CDCl_3$) δ: -78.16; ^{31}P NMR (162 MHz, $CDCl_3$) δ: 22.72; m/z LRMS (ESI + APCI): $[M-OTf]^+$ calculated for $C_{38}H_{37}ClN_2O_3P^+$ = 635.2, found 635.3.

(2-Methyl-3-((3-methylpyridin-2-yl)oxy)pyridin-4-yl)triphenylphosphonium trifluoromethanesulfonate (1ak)

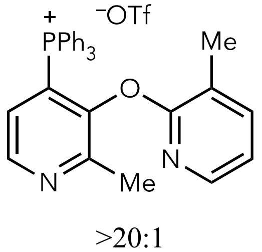

>20:1

Prepared according to general procedure A using 2-methyl-3-((3-methylpyridin-2-yl)oxy)pyridine (1.20 g, 6.00 mmol), Tf_2O (1.01 mL, 6.00 mmol), PPh_3 (1.73 g, 6.60 mmol), DBU (896 μL, 6.00 mmol), and CH_2Cl_2 (60.0 mL, 0.10 M). After the purification procedure, the title compound was isolated as a white solid (3.05

g, 5.00 mmol, 83% yield). mp: 211–213 °C; ^{1}H NMR (400 MHz, $CDCl_3$) δ: 8.71 (*app* t, *J* = 4.6 Hz, 1H), 7.77 (tt, *J* = 6.9, 1.6 Hz, 3H), 7.72 – 7.46 (m, 13H), 7.24 – 7.19 (m, 1H), 7.10 (dd, *J* = 14.0, 5.0 Hz, 1H), 6.79 (dd, *J* = 7.3, 4.9 Hz, 1H), 2.27 (s, 3H), 1.65 (s, 3H); ^{13}C NMR (100 MHz, $CDCl_3$) δ: 157.97, 156.70 (d, *J* = 5.0 Hz), 149.64, 146.95 (d, *J* = 11.6 Hz), 143.69, 140.52, 135.67 (d, *J* = 3.1 Hz), 134.02 (d, *J* = 10.7 Hz), 130.50 (d, *J* = 13.3 Hz), 125.71 (d, *J* = 7.6 Hz), 121.42 (d, *J* = 86.0 Hz), 120.90 (q, *J* = 322.2 Hz) 120.16, 119.62, 115.83 (d, *J* = 91.1 Hz), 21.01 (d, *J* = 1.9 Hz), 15.15; ^{19}F NMR (376 MHz, $CDCl_3$) δ: -78.05; ^{31}P NMR (162 MHz, $CDCl_3$) δ: 21.58; IR υ_{max}/cm^{-1} (film): 3010, 2360, 2341, 1585, 1438, 1392, 1262, 1222, 1180, 1151, 1107, 1029, 997, 745, 723, 635; m/z LRMS (ESI + APCI): [M−OTf]$^+$ calculated for $C_{30}H_{26}N_2OP^+$ = 461.2, found 461.3.

Triphenyl(2-(2-((6-(trifluoromethyl)pyridin-2-yl)oxy)ethyl)pyridin-4-yl)phosphonium trifluoromethanesulfonate (1al)

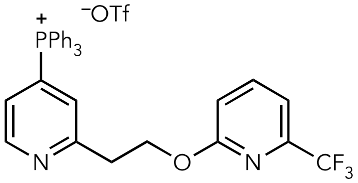

>20:1

Prepared according to general procedure A using 2-(2-(pyridin-2-yl)ethoxy)-6-(trifluoromethyl)pyridine (322 mg, 1.20 mmol), Tf_2O (202 μL, 1.20 mmol), PPh_3 (346 mg, 1.32 mmol), DBU (179 μL, 1.20 mmol), and CH_2Cl_2 (12.0 mL, 0.10 M). After the purification procedure, the title compound was isolated as a pale–yellow solid (496 mg, 0.73 mmol, 61% yield). mp: 41–43 °C; ^{1}H NMR (400 MHz, $CDCl_3$) δ: 8.91 (*app* t, *J* = 5.1 Hz, 1H), 7.88 (dt, *J* = 7.6, 4.5 Hz, 3H), 7.75 (td, *J* = 7.8, 3.6 Hz, 7H), 7.61 (d, *J* = 7.7 Hz, 3H), 7.57 (d, *J* = 7.9 Hz, 3H), 7.45 – 7.35 (m, 2H), 7.20 (d, *J* = 7.3 Hz, 1H), 6.71 (d, *J* = 8.4 Hz, 1H), 4.67 (t, *J* = 6.2 Hz, 2H), 3.36 (t, *J* = 6.2 Hz, 2H); ^{13}C NMR (100 MHz, $CDCl_3$) δ: 163.23, 161.56 (d, *J* = 10.0 Hz), 151.41 (d, *J* = 10.6 Hz), 145.18 (q, *J* = 34.8 Hz), 140.03, 136.28 (d, *J* = 3.0 Hz), 134.46 (d, *J* = 10.5 Hz), 131.04 (d, *J* = 13.1 Hz), 128.82 (d, *J* = 83.7 Hz), 126.81 (d, *J* = 8.3 Hz), 125.15 (d, *J* = 8.0 Hz), 121.26 (d, *J* = 273.6 Hz), 120.85 (q, *J* = 321.2 Hz), 115.72 (d, *J* = 89.7 Hz), 114.49, 113.66 (q, *J* = 3.2 Hz), 64.75, 37.58 (d, *J* = 1.5 Hz); ^{19}F NMR (376 MHz, $CDCl_3$) δ: -68.29, -78.14; ^{31}P NMR (162 MHz, $CDCl_3$) δ: 22.42; IR υ_{max}/cm^{-1} (film): 3011, 2360, 2341, 1607, 1578, 1457, 1438, 1348, 1260, 1223, 1137, 1120, 1108, 1029, 997, 813, 748, 725, 667; m/z LRMS (ESI + APCI): [M−OTf]$^+$ calculated for $C_{31}H_{25}F_3N_2OP^+$ = 529.2, found 529.3.

(2-(4-(((6-Chloropyridin-3-yl)methoxy)methyl)phenyl)pyridin-4-yl)triphenylphosphonium trifluoromethanesulfonate (1am)

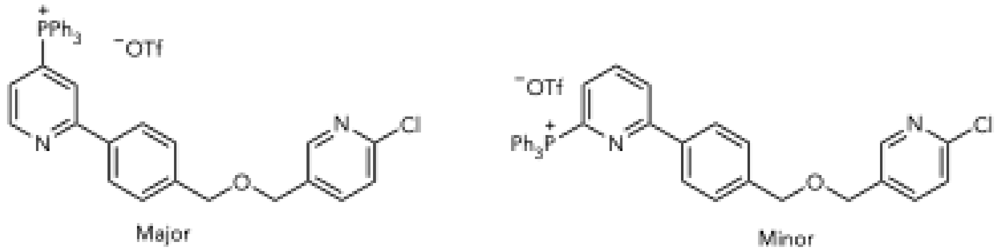

11:1 Mixture of isomers

Prepared according to our previous report[5] using 2–chloro–5–(((4–(pyridin–2–yl)benzyl)oxy)methyl)pyridine (466 µL, 1.50 mmol), Tf_2O (253 µL, 1.50 mmol), PPh_3 (433 mg, 1.65 mmol), DBU (227 µL, 1.50 mmol) and CH_2Cl_2 (15.0 mL, 0.10 M). The title compound was isolated as a white solid (957 mg, 1.33 mmol, 88% combined yield). ^{1}H NMR (Major; 400 MHz, $CDCl_3$) δ: 9.07 (*app* t, *J* = 5.2 Hz, 1H), 8.34 (s, 1H), 7.98 – 7.87 (m, 5H), 7.86 – 7.79 (m, 6H), 7.77 – 7.58 (m, 8H), 7.53 (dd, *J* = 12.7, 5.2 Hz, 1H), 7.46 (d, *J* = 7.9 Hz, 2H), 7.31 (d, *J* = 8.1 Hz, 1H), 4.62 (s, 2H), 4.55 (s, 2H); ^{19}F NMR (Both isomers; 376 MHz, $CDCl_3$) δ: -78.12; ^{31}P NMR (162 MHz, $CDCl_3$) δ: 22.84 (major), 15.47 (minor).

(5-((6-Chloropyridin-3-yl)methoxy)-2-methylpyridin-4-yl)triphenylphosphonium trifluoromethanesulfonate (1an)

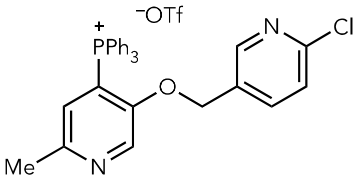

>20:1

Prepared according to general procedure A using 2-chloro-5-(((6-methylpyridin-3-yl)oxy)methyl) pyridine (1.17 g, 5.00 mmol), Tf_2O (841 µL, 5.00 mmol), PPh_3 (1.44 g, 5.50 mmol), Et_3N (746 µL, 5.00 mmol), and CH_2Cl_2 (50.0 mL, 0.10 M). After the purification procedure, the title compound was isolated as a tan solid (2.23 g, 3.46 mmol, 69% yield). mp: 77–81 °C; ^{1}H NMR (400 MHz, $CDCl_3$) δ: 8.74 (*app* d, *J* = 7.0 Hz, 1H), 7.83 (td, *J* = 7.4, 1.8 Hz, 3H), 7.69 (td, *J* = 7.8, 3.7 Hz, 6H), 7.62 (d, *J* = 2.4 Hz, 1H), 7.55 (dd, *J* = 13.6, 7.4 Hz, 6H), 7.22 (dd, *J* = 8.2, 2.5 Hz, 1H), 7.06 (d, *J* = 8.2 Hz, 1H), 6.80 (d, *J* = 15.2 Hz, 1H), 5.16 (s, 2H), 2.50 (s, 3H); ^{13}C NMR (100 MHz, $CDCl_3$) δ: 153.48 (d, *J* = 11.0 Hz), 152.84, 151.43, 149.05, 139.12, 136.44 (d, *J* = 5.0 Hz), 135.69 (d, *J* = 3.2 Hz), 133.96 (d, *J* = 10.8 Hz), 130.73 (d, *J* = 13.3 Hz), 128.91, 127.27 (d, *J* = 7.0 Hz), 124.14, 120.93 (q, *J* = 321.0 Hz), 116.50 (d, *J* = 91.2 Hz), 115.25 (d, *J* = 86.5 Hz), 68.65, 23.84; ^{19}F NMR (376 MHz, $CDCl_3$) δ: -78.13; ^{31}P NMR (162 MHz, $CDCl_3$) δ: 21.46; IR υ_{max}/cm^{-1} (film): 3010, 2360, 2341, 1484, 1460, 1438, 1351, 1260, 1222, 1106, 1029, 996, 746, 722, 688, 667, 636; m/z LRMS (ESI + APCI): [M−OTf]$^+$ calculated for $C_{30}H_{25}ClN_2OP^+$ = 495.1, found 495.2.

(2-(((3-Cyano-6-(trifluoromethyl)pyridin-2-yl)oxy)methyl)-3-fluoropyridin-4-yl)triphenylphosphonium trifluoromethanesulfonate (1ao)

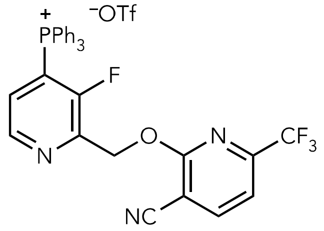

>20:1

Prepared according to general procedure B using 2-((3-fluoropyridin-2-yl)methoxy)-6-(trifluoromethyl)nicotinonitrile (892 mg, 3.00 mmol), Tf_2O (505 μL, 3.00 mmol), PPh_3 (865 mg, 3.30 mmol), DBU (448 μL, 3.00 mmol), and CH_2Cl_2 (30.0 mL, 0.10 M). After the purification procedure, the title compound was isolated as a white solid (1.58 g, 2.23 mmol, 74% yield). mp: 219–221 °C; ^{1}H NMR (400 MHz, $CDCl_3$) δ: 8.71 (*app* t, *J* = 4.2 Hz, 1H), 8.12 (d, *J* = 7.7 Hz, 1H), 7.90 (td, *J* = 7.6, 2.1 Hz, 3H), 7.75 (td, *J* = 7.8, 3.8 Hz, 6H), 7.62 (dd, *J* = 13.8, 7.6 Hz, 6H), 7.40 (d, *J* = 7.7 Hz, 1H), 7.26 – 7.19 (m, 1H), 5.74 (d, *J* = 1.7 Hz, 2H); ^{13}C NMR (100 MHz, $CDCl_3$) δ: 162.15, 157.10 (d, *J* = 268.7 Hz), 148.04 (q, *J* = 36.1 Hz), 147.14 (dd, *J* = 10.7, 6.5 Hz), 146.11 (dd, *J* = 14.9, 4.4 Hz), 145.37, 136.11 (d, *J* = 3.1 Hz), 133.90 (d, *J* = 11.0 Hz), 130.79 (d, *J* = 13.5 Hz), 128.51 (dd, *J* = 6.1, 1.9 Hz), 120.56 (q, *J* = 321.0 Hz), 120.01 (d, *J* = 274.7 Hz), 116.72 (d, *J* = 84.3 Hz), 114.81 (d, *J* = 91.0 Hz), 114.09 (q, *J* = 3.0 Hz), 113.21, 100.35, 64.57; ^{19}F NMR (376 MHz, $CDCl_3$) δ: -69.01, -78.38, -108.08; ^{31}P NMR (162 MHz, $CDCl_3$) δ: 20.76 (d, *J* = 3.7 Hz); IR υ_{max}/cm^{-1} (film): 2360, 2341, 1440, 1417, 1347, 1258, 1185, 1147, 1118, 1104, 1029, 637; m/z LRMS (ESI + APCI): [M−OTf]$^+$ calculated for $C_{31}H_{21}F_4N_3OP^+$ = 558.1, found 558.2.

(2-(1-((6-Chloro-5-cyano-3-fluoropyridin-2-yl)oxy)ethyl)pyridin-4-yl)triphenylphosphonium trifluoromethanesulfonate (1ap)

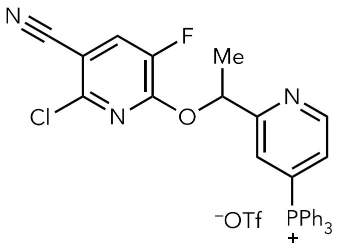

>20:1

Prepared according to general procedure B using 2-chloro-5-fluoro-6-(1-(pyridin-2-yl)ethoxy)nicotinonitrile (1.11 g, 4.00 mmol), Tf_2O (673 μL, 4.00 mmol), PPh_3 (1.15 g, 4.40 mmol), DBU (597 μL, 4.00 mmol), and CH_2Cl_2 (40.0 mL, 0.10 M). After the purification procedure, the title compound

was isolated as an off–white solid (2.15 g, 3.13 mmol, 78% yield). mp: 74–76 °C; ^{1}H NMR (400 MHz, $CDCl_3$) δ: 9.01 (*app* t, *J* = 5.0 Hz, 1H), 7.99 – 7.89 (m, 3H), 7.79 (td, *J* = 7.9, 3.8 Hz, 6H), 7.73 – 7.54 (m, 9H), 6.27 (q, *J* = 6.6 Hz, 1H), 1.81 (d, *J* = 6.6 Hz, 3H); ^{13}C NMR (100 MHz, $CDCl_3$) δ: 161.91 (d, *J* = 10.1 Hz), 153.51 (d, *J* = 12.8 Hz), 151.62 (d, *J* = 10.3 Hz), 145.39 (d, *J* = 263.4 Hz), 145.15 (d, *J* = 3.9 Hz), 136.34 (d, *J* = 3.1 Hz), 134.54 (d, *J* = 10.5 Hz), 131.09 (d, *J* = 13.2 Hz), 129.57 (d, *J* = 84.0 Hz), 128.50 (d, *J* = 19.8 Hz), 126.76 (d, *J* = 8.1 Hz), 124.22 (d, *J* = 9.0 Hz), 120.80 (q, *J* = 322.2 Hz), 115.59 (d, *J* = 89.6 Hz), 113.94, 102.75 (d, *J* = 3.3 Hz), 76.62 (d, *J* = 1.7 Hz), 20.48; ^{19}F NMR (376 MHz, $CDCl_3$) δ: -78.19, -138.04 (d, *J* = 8.3 Hz); ^{31}P NMR (162 MHz, $CDCl_3$) δ: 22.67; IR υ_{max}/cm^{-1} (film): 3011, 2360, 2341, 1608, 1584, 1565, 1456, 1439, 1396, 1326, 1262, 1222, 1152, 1108, 1029, 745, 725, 688, 636; m/z LRMS (ESI + APCI): [M−OTf]$^+$ calculated for $C_{31}H_{23}ClFN_3OP^+$ = 538.1, found 538.2.

(2-Methyl-5-((6-methylpyrimidin-4-yl)oxy)pyridin-4-yl)triphenylphosphonium trifluoromethanesulfonate (1aq)

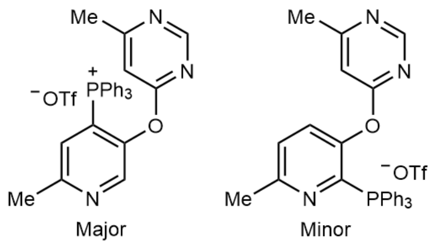

14.3:1 Mixture of Isomers

Prepared according to general procedure A using 4-methyl-6-((6-methylpyridin-3-yl)oxy)pyrimidine (604 mg, 3.00 mmol), Tf_2O (505 μL, 3.00 mmol), PPh_3 (866 mg, 3.30 mmol), DBU (448 μL, 3.00 mmol), and CH_2Cl_2 (30.0 mL, 0.10 M). After the purification procedure, the title compound was isolated as a pale–yellow solid (1.48 g, 2.42 mmol, 81% combined yield). mp: 70–74 °C; ^{1}H NMR (Major; 400 MHz, $CDCl_3$) δ: 8.77 (*app* d, *J* = 6.5 Hz, 1H), 8.40 (s, 1H), 7.80 (td, *J* = 7.1, 1.9 Hz, 3H), 7.72 – 7.58 (m, 12H), 7.08 (d, *J* = 14.6 Hz, 1H), 6.16 (s, 1H), 2.65 (s, 3H), 2.32 (s, 3H); ^{13}C NMR (Major; 100 MHz, $CDCl_3$) δ: 170.52, 166.56, 157.99 (d, *J* = 10.3 Hz), 157.13, 147.37, 146.63 (d, *J* = 5.1 Hz), 135.96 (d, *J* = 3.2 Hz), 134.31 (d, *J* = 10.9 Hz), 130.92 (d, *J* = 13.3 Hz), 127.47 (d, *J* = 6.9 Hz), 120.99 (q, *J* = 319.0 Hz), 120.97 (d, *J* = 85.0 Hz), 115.78 (d, *J* = 90.9 Hz), 107.02, 24.41, 23.90; ^{19}F NMR (Both isomers; 376 MHz, $CDCl_3$) δ: -78.12; ^{31}P NMR (162 MHz, $CDCl_3$) δ: 20.99 (major), 20.60 (minor), 15.65 (minor); IR υ_{max}/cm^{-1} (film): 3009, 2360, 2341, 1595, 1555, 1472, 1438, 1432, 1262, 1223, 1199, 1146, 1107, 1029, 749, 723, 635; m/z LRMS (ESI + APCI): [M−OTf]$^+$ calculated for $C_{29}H_{25}N_3OP^+$ = 462.2, found 462.2.

(2-(2-(4-((2,4-Dioxo-3-(pyridin-2-ylmethyl)thiazolidin-5-yl)methyl)phenoxy)ethyl)-5-ethylpyridin-4-yl)triphenylphosphonium trifluoromethanesulfonate (1ar)

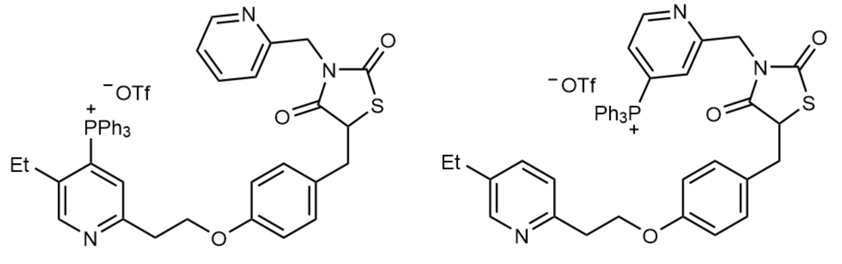

15.7:1 Mixture of Isomers

Prepared according to general procedure A using 5-(4-(2-(5-ethylpyridin-2-yl)ethoxy)benzyl)-3-(pyridin-2-ylmethyl)thiazolidine-2,4-dione (671 mg, 1.50 mmol), Tf_2O (252 µL, 1.50 mmol), PPh_3 (432 mg, 1.65 mmol), DBU (224 µL, 1.50 mmol), and CH_2Cl_2 (15.0 mL, 0.10 M). After the purification procedure, the title compound was isolated as a >15:1 mixture of isomers as a white solid (1.03 g, 1.20 mmol, 80% combined yield). mp: 89–94 °C; ^{1}H NMR (Major; 400 MHz, $CDCl_3$) δ: 8.84 (*app* d, *J* = 7.1 Hz, 1H), 8.49 (d, *J* = 4.6 Hz, 1H), 7.89 – 7.81 (m, 3H), 7.75 (td, *J* = 7.8, 3.7 Hz,7H), 7.69 – 7.62 (m, 6H), 7.16 (dd, *J* = 7.5, 4.9 Hz, 1H), 7.10 (d, *J* = 8.4 Hz, 2H), 7.05 (s, 1H), 7.02 (d, *J* = 6.4 Hz, 1H), 6.65 (d, *J* = 8.5 Hz, 2H), 4.85 (s, 2H), 4.54 (dd, *J* = 9.1, 4.1 Hz, 1H), 4.28 (t, *J* = 6.0 Hz, 2H), 3.47 (dd, *J* = 14.2, 4.1 Hz, 1H), 3.28 (t, *J* = 5.9 Hz, 2H), 3.13 (dd, *J* = 14.2, 9.1 Hz, 1H), 2.38 (q, *J* = 7.4 Hz, 2H), 0.76 (t, *J* = 7.4 Hz, 3H); ^{13}C NMR (Major; 100 MHz, $CDCl_3$) δ: 173.75, 171.04, 159.02 (d, *J* = 10.7 Hz), 157.90, 153.90, 152.64 (d, *J* = 8.7 Hz), 149.62, 139.76 (d, *J* = 7.1 Hz), 136.82, 136.02 (d, *J* = 3.1 Hz), 134.27 (d, *J* = 10.4 Hz), 131.10 (d, *J* = 13.0 Hz), 130.57, 128.21, 127.60 (d, *J* = 10.6 Hz), 126.32 (d, *J* = 82.9 Hz), 122.75, 121.52, 120.96 (q, *J* = 321.3 Hz),116.67 (d, *J* = 88.6 Hz), 114.70, 65.87, 51.82, 46.16, 37.74, 37.43, 26.73 (d, *J* = 5.1 Hz), 13.70; ^{19}F NMR (Both isomers; 376 MHz, $CDCl_3$) δ: -78.09; ^{31}P NMR (162 MHz, $CDCl_3$) δ: 22.37 (minor), 21.55 (major); IR υ_{max}/cm^{-1} (film): 3011, 2360, 2341, 1680, 1585, 1511, 1478, 1437, 1381, 1262, 1223, 1178, 1152, 1106, 1029, 996, 745, 723, 689, 636; m/z LRMS (ESI + APCI): [M−OTf]$^+$ calculated for $C_{43}H_{39}N_3O_3PS^+$ = 708.2, found 708.3.

(11-(1-(Ethoxycarbonyl)piperidin-4-ylidene)-8-(pyridin-2-yl)-6,11-dihydro-5*H*-benzo[5,6]cyclohepta[1,2-*b*]pyridin-4-yl)triphenylphosphonium trifluoromethanesulfonate (1as)

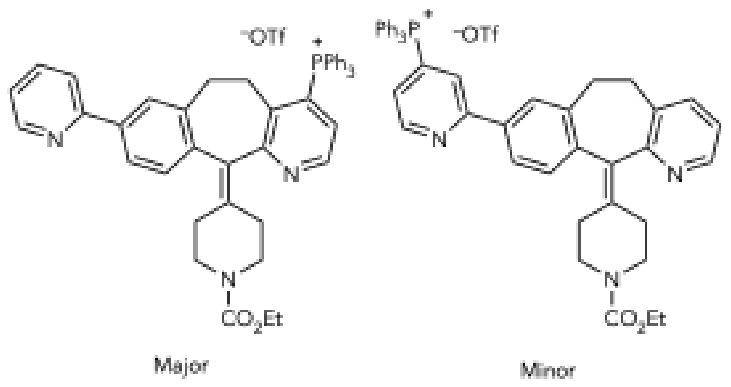

20:1 Mixture of Isomers

Prepared according to our previous report[5] using general procedure A with ethyl 4-(8-(pyridin-2-yl)-5,6-dihydro-11H-benzo[5,6]cyclohepta[1,2-b]pyridin-11-ylidene)piperidine-1-carboxylate (1.06 g, 2.50 mmol), Tf_2O (421 µL, 2.50 mmol), PPh_3 (656 mg, 2.50 mmol), DBU (373 µL, 2.50 mmol), and CH_2Cl_2 (25.0 mL, 0.10 M). After the purification procedure, the title compound was isolated as a pale–yellow solid (1.44 g, 1.72 mmol, 69% combined yield). ^{1}H NMR (Major; 400 MHz, $CDCl_3$) δ: 8.74 (*app* t, *J* = 4.8 Hz, 1H), 8.65 (ddd, *J* = 4.9, 1.9, 0.9 Hz, 1H), 7.97 – 7.89 (m, 3H), 7.80 (td, *J* = 7.8, 3.8 Hz, 7H), 7.71 – 7.64 (m, 8H), 7.43 (d, *J* = 1.8 Hz, 1H), 7.28 (d, *J* = 8.0 Hz, 1H), 7.26 – 7.21 (m, 1H), 7.04 (dd, *J* = 14.8, 5.1 Hz, 1H), 4.15 (q, *J* = 7.0 Hz, 2H), 3.84 – 3.66 (m, 2H), 3.36 (dp, *J* = 12.6, 4.1 Hz, 3H), 2.88 (dt, *J* = 17.4, 5.0 Hz, 1H), 2.67 – 2.51 (m, 2H), 2.49 – 2.36 (m, 2H), 2.20 (dd, *J* = 13.0, 7.0 Hz, 1H), 1.74 (ddd, *J* = 17.3, 12.1, 5.0 Hz, 1H), 1.26 (t, *J* = 7.1 Hz, 3H); ^{19}F NMR (Both isomers; 376 MHz, $CDCl_3$) δ: -78.09; ^{31}P NMR (162 MHz, $CDCl_3$) δ: 22.81 (minor), 21.26 (major); m/z LRMS (ESI + APCI): $[M{-}OTf]^+$ calculated for $C_{45}H_{41}N_3O_2P^+$ = 686.3, found 686.4.

(2-(11-(1-(Ethoxycarbonyl)piperidin-4-ylidene)-6,11-dihydro-5*H*-benzo[5,6]cyclohepta[1,2-*b*]pyridin-8-yl)pyridin-4-yl)triphenylphosphonium trifluoromethanesulfonate (1at)

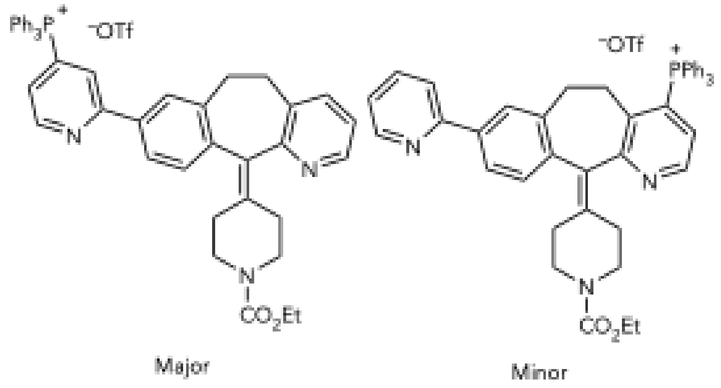

12:1 Mixture of Isomers

Prepared according to our previous report[5] using general procedure C with ethyl 4-(8-(pyridin-2-yl)-5,6-dihydro-11H-benzo[5,6]cyclohepta[1,2-b]pyridin-11-ylidene)piperidine-1-carboxylate (1.06 g, 2.50 mmol), Tf_2O (841 μL, 5.00 mmol), PPh_3 (1.31 g, 5.00 mmol), Et_3N (698 μL, 5.00 mmol), and CH_2Cl_2 (25.0 mL, 0.10 M). After the purification procedure, the title compound was isolated as a red solid (1.08 g, 1.29 mmol, 52% combined yield). ^{1}H NMR (Major; 400 MHz, $CDCl_3$) δ: 9.05 (*app* t, *J* = 5.1 Hz, 1H), 8.44 (dd, *J* = 5.4, 1.6 Hz, 1H), 7.96 – 7.90 (m, 5H), 7.81 (td, *J* = 7.8, 3.7 Hz, 7H), 7.73 – 7.65 (m, 7H), 7.53 – 7.40 (m, 2H), 7.37 (d, *J* = 8.0 Hz, 1H), 4.12 (q, *J* = 7.1 Hz, 2H), 3.93 (d, *J* = 12.6 Hz, 1H), 3.82 (s, 1H), 3.47 (ddt, *J* = 13.7, 6.9, 4.3 Hz, 2H), 3.17 – 2.96 (m, 4H), 2.57 (ddd, *J* = 14.3, 9.7, 4.8 Hz, 1H), 2.43 (ddd, *J* = 14.1, 9.4, 4.7 Hz, 1H), 2.34 (dt, *J* = 14.1, 4.6 Hz, 1H), 2.29 – 2.14 (m, 1H), 1.24 (t, *J* = 7.1 Hz, 3H); ^{19}F NMR (Both isomers; 376 MHz, $CDCl_3$) δ: -78.15; ^{31}P NMR (162 MHz, $CDCl_3$) δ: 22.83 (major), 21.29 (minor); m/z LRMS (ESI + APCI): $[M{-}OTf]^+$ calculated for $C_{45}H_{41}N_3O_2P^+$ = 686.3, found 686.4.

(2,6-Dimethylpyridin-4-yl)diphenyl(2-phenylpyridin-4-yl)phosphonium trifluoromethanesulfonate (2b')

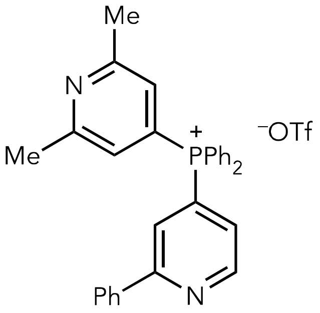

Prepared according to general procedure B using 2-phenylpyridine (357 *μL*, 2.50 mmol), Tf_2O (421 μL, 2.50 mmol), 4-(diphenylphosphaneyl)-2,6-dimethylpyridine (801 mg, 2.75 mmol), DBU (373 μL, 2.50 mmol), and CH_2Cl_2 (25.0 mL, 0.10 M). After the purification procedure (two precipitations were required), the title compound was isolated as a white solid (866 mg, 1.46 mmol, 58% combined yield). mp: 69–72 °C; ^{1}H NMR (400 MHz, $CDCl_3$) δ: 9.11 (*app* td, *J* = 5.2, 0.9 Hz, 1H), 8.00 – 7.88 (m, 4H), 7.87 – 7.80 (m, 4H), 7.79 (t, *J* = 1.2 Hz, 1H), 7.76 – 7.63 (m, 4H), 7.57 (ddd, *J* = 12.9, 5.0, 1.6 Hz, 1H), 7.47 (dt, *J* = 4.6, 2.9 Hz, 3H), 7.21 (d, *J* = 13.7 Hz, 2H), 2.66 (s, 6H); ^{13}C NMR (100 MHz, $CDCl_3$) δ: 161.29 (d, *J* = 11.0 Hz), 159.61 (d, *J* = 10.2 Hz), 152.13 (d, *J* = 10.8 Hz), 136.99, 136.78 (d, *J* = 3.1 Hz), 134.85 (d, *J* = 10.7 Hz), 131.42 (d, *J* = 13.2 Hz), 130.83, 129.37, 128.19 (d, *J* = 84.0 Hz), 127.38, 127.05 (d, *J* = 83.6 Hz), 125.63 (d, *J* = 8.2 Hz), 123.66 (d, *J* = 8.3 Hz), 123.46 (d, *J* = 8.7 Hz), 120.82 (q, *J* = 321.2 Hz), 114.50 (d, *J* = 89.3 Hz), 25.09 (d, *J* = 1.7 Hz); ^{19}F NMR (376 MHz, $CDCl_3$) δ: -78.21 (d, *J* = 2.4 Hz); ^{31}P NMR (162 MHz, $CDCl_3$) δ: 22.04; IR υ_{max}/cm^{-1} (film): 3009, 2360, 2342, 1574, 1439, 1377, 1260, 1222, 1150, 1029, 745, 635; m/z LRMS (ESI + APCI): $[M{-}OTf]^+$ calculated for $C_{30}H_{26}N_2P^+$ = 445.2, found 445.3.

1.7 Alkylation of Heterocycles

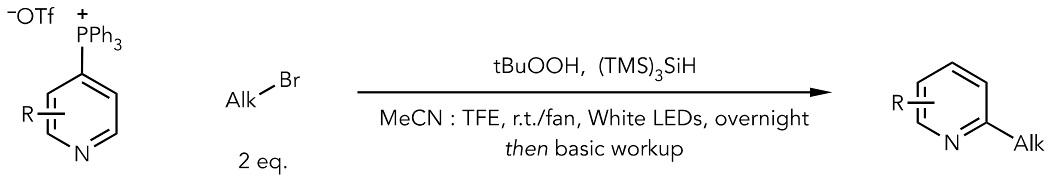

General Procedure E

An oven-dried 16 mL vial (≤ 0.50 mmol scale) or a round bottom flask (> 0.50 mmol scale) equipped with a stir bar was charged with the heterocyclic phosphonium salt (1.00 equiv), MeCN:TFE (1:1; 0.05 M), *t*BuOOH (2.50 equiv), alkyl bromide (2.00 equiv), and $(TMS)_3SiH$ (2.00 equiv). The reaction was sealed under an atmosphere of air and placed in the white LED photoreactor setup with stirring (refer to Figures S1–3) while cooling the reaction vessel with a stream of compressed air. After the stated time, the reaction vessel was removed from the photoreactor and the crude reaction mixture was transferred to a separatory funnel. The reaction mixture was washed with hexanes (6x), then diluted in CH_2Cl_2 and washed with a 1.0 M aqueous solution of NaOH (3x) before being dried (Na_2SO_4), filtered, and concentrated *in vacuo*. The residue was purified via flash column chromatography under the stated conditions to provide the pure alkylated heterocycle.

General Procedure F

An oven-dried 16 mL vial (≤ 0.50 mmol scale) or a round bottom flask (> 0.50 mmol scale) equipped with a stir bar was charged with the heterocyclic phosphonium salt (1.00 equiv), MeCN:TFE (1:20; 0.02 M), TfOH (1.00 equiv.), *t*BuOOH (2.50 equiv), alkyl bromide (2.00 equiv), and $(TMS)_3SiH$ (2.00 equiv). The reaction was sealed under an atmosphere of air and placed in the white LED photoreactor setup with stirring (refer to Figures S1–3) while cooling the reaction vessel with a stream of compressed air. After the stated time, the reaction vessel was removed from the photoreactor and the crude reaction mixture was transferred to a separatory funnel. The reaction mixture was washed with hexanes (6x), then diluted in CH_2Cl_2 and washed with a 1.0 M aqueous solution of NaOH (3x) before being dried (Na_2SO_4), filtered, and concentrated *in vacuo*. The residue was purified via flash column chromatography under the stated conditions to provide the pure alkylated heterocycle.

Reaction Notes

1) Partitioning the crude reaction between the MeCN:TFE mixture and hexanes and washing repeatedly with hexanes removes silane by-products that can co-elute with the alkylated heterocyclic products in chromatographic separations and should be done before subjecting the crude reaction to aqueous NaOH.

2) Washing the crude reaction with 1.0 M aqueous NaOH decomposes heterocyclic phosphonium salts, providing the parent heterocycle. In a small number of cases, decomposition of the alkylated phosphonium salt by shaking with 1.0 M aqueous NaOH did not proceed to completion; it is recommended to ensure complete phosphonium decomposition via ^{31}P NMR at this step.
3) In cases where phosphonium salt decomposition does not proceed to completion, it is recommended to transfer the crude reaction mixture to a round bottom flask in CH_2Cl_2 with 1.0 M aqueous NaOH (~20 mL for a 0.50 mmol scale reaction) and concentrate the organic layer *in vacuo* on a rotary evaporator at 40 °C, followed by extraction of the aqueous layer with CH_2Cl_2 (3x). Instances of where this phenomenon was observed are noted below.
4) For substrates where the alkylation yield is low, yields can generally be improved by washing the crude reaction with hexanes (6x), then adding *t*BuOOH (2.50 equiv), alkyl bromide (2.00 equiv), and $(TMS)_3SiH$ (2.00 equiv) and re-placing the reaction in the white LED photoreactor setup with stirring for another 18–24 hours.
5) Some substrates undergo background alkylation with the peroxide radical initiator, producing *t*-butyl byproducts. Cases where this was observed are noted and were generally isolable from the desired alkylated products.
6) In some cases where Lewis basic atoms are present in the pyridylphosphonium salt, adding 1.0 equivalent of TfOH can help promote the desired reactivity.
7) If regiomeric mixtures of phosphoniums were formed, generally regiomeric mixtures of alkylated products were observed that correspond to location of the minor phosphonium impurity. If mixtures were obtained, the crude NMRs are shown later in this document with the observed ratios, and the major alkylated product was isolated and characterized.

(2-cyclohexyl-6-phenylpyridin-4-yl)triphenylphosphonium trifluoromethanesulfonate (2a)

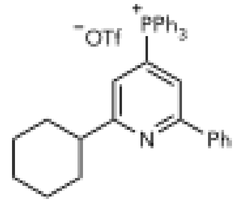

Prepared according to a modified general procedure E *without* the basic NaOH workup. An oven-dried 16 mL vial equipped with a stir bar was charged with **1a** (283 mg, 0.50 mmol), MeCN:TFE (1:1; 0.05 M, 10.0 mL), *t*BuOOH (227 µL, 1.25 mmol), cyclohexyl bromide (123 µL, 1.00 mmol), and $(TMS)_3SiH$ (309 µL, 1.00 mmol). The reaction was sealed under an atmosphere of air and placed in the white LED photoreactor setup with stirring for 17 hours while cooling the reaction vessel with a stream of compressed air. The crude reaction mixture was transferred to a separatory funnel and washed with hexanes (6x), then diluted in

approximately 4-5 mL of CH_2Cl_2 and was added dropwise to an excess of chilled Et_2O (0 °C). The flask was then placed in a –20 °C refrigerator for approximately 1 hour. The resulting suspension was filtered on a frit, the solid washed with chilled Et_2O (0 °C), and dried *in vacuo* to provide the title compound as a yellow solid (292 mg, 0.45 mmol, 90% yield). ^{1}H NMR (400 MHz, CD_3OD) δ: 8.03 – 7.95 (m, 6H), 7.88 – 7.83 (m, 12H), 7.59 (d, *J* = 13.3 Hz, 1H), 7.54 – 7.47 (m, 3H), 2.99 (tt, *J* = 11.8, 3.4 Hz, 1H), 2.02 (d, *J* = 12.8 Hz, 2H), 1.88 (d, *J* = 12.8 Hz, 2H), 1.77 (d, *J* = 12.8 Hz, 1H), 1.63 (qd, *J* = 12.4, 3.3 Hz, 2H), 1.55 – 1.41 (m, 2H), 1.38 – 1.26 (m, 1H), 1.48 (qt, *J* = 12.6, 3.2 Hz, 2H), 1.40 – 1.26 (m, 1H); ^{13}C NMR (100 MHz, CD_3OD) δ: 167.25 (d, *J* = 10.6 Hz), 157.75 (d, *J* = 11.6 Hz), 137.23 (d, *J* = 3.2 Hz), 136.23 (d, *J* = 10.8 Hz), 134.98, 132.59, 131.93, 130.23, 129.59, 128.06, 126.87 (dd, *J* = 9.7, 5.0 Hz), 121.70 (q, *J* = 319.2 Hz), 117.60, 116.71, 45.63, 33.21, 27.04, 26.47.; ^{19}F NMR (376 MHz, CD_3OD) δ: -80.22 ; ^{31}P NMR (162 MHz, CD_3OD) δ: 23.20; m/z LRMS (ESI + APCI): $[M{-}OTf]^+$ calculated for $C_{35}H_{33}NP^+$ = 498.2, found 498.3

2-Cyclohexyl-6-phenylpyridine (3a)

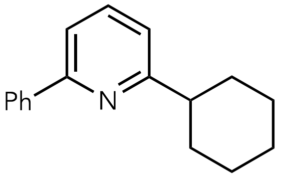

One-pot synthesis:

An oven-dried 16 mL vial equipped with a stir bar under a nitrogen atmosphere was charged with 2-phenylpyridine (72.0 µL, 0.50 mmol) and CH_2Cl_2 (1.00 mL, 0.50 M). The reaction vessel was cooled to –78 °C and Tf_2O (84.0 µL, 0.50 mmol) was added dropwise over 5 minutes. The reaction was stirred at –78 °C for 30 minutes before PPh_3 (144 mg, 0.55 mmol) was added in one solid portion. The reaction was subjected to three rapid cycles of vacuum/nitrogen backfill and was stirred for a further 30 minutes at –78 °C. Et_3N (70.0 µL, 0.50 mmol) was added dropwise via syringe, the cooling bath was removed, and the reaction was allowed to warm to room temperature while stirring (approximately 15-30 minutes). The reaction was concentrated *in vacuo* before adding *t*BuOOH (90% in H_2O; 138 µL 1.25 mmol), bromocyclohexane (124 µL, 1.00 mmol), $(TMS)_3SiH$ (309 µL, 1.00 mmol), and MeCN:TFE (1:1; 10.0 mL). The reaction was sealed under an atmosphere of air and placed in the white LED photoreactor setup with stirring for 18 hours. The crude reaction mixture was transferred to a separatory funnel and washed with hexanes (6x), then diluted in CH_2Cl_2 and washed with a 1.0 M aqueous solution of NaOH (3x) before being dried (Na_2SO_4), filtered, and concentrated *in vacuo*. The crude material was purified by a flash chromatography column (silica gel: 0 to 3% Et_2O in hexanes) to provide the title compound as a colorless

oil (72.0 mg, 0.30 mmol, 61% yield). ^{1}H NMR (400 MHz, $CDCl_3$) δ: 8.07 (d, J = 7.1 Hz, 2H), 7.66 (t, J = 7.7 Hz, 1H), 7.54 (dd, J = 7.9, 1.0 Hz, 1H), 7.49 (t, J = 7.4 Hz, 2H), 7.45 – 7.33 (m, 1H), 7.10 (dd, J = 7.7, 0.9 Hz, 1H), 2.82 (tt, J = 11.9, 3.5 Hz, 1H), 2.06 (dtq, J = 10.7, 3.6, 1.7 Hz, 2H), 1.91 (dt, J = 12.7, 3.3 Hz, 2H), 1.80 (dtt, J = 11.3, 3.2, 1.5 Hz, 1H), 1.64 (qd, J = 12.4, 3.2 Hz, 2H), 1.48 (qt, J = 12.6, 3.2 Hz, 2H), 1.40 – 1.26 (m, 1H); ^{13}C NMR (100 MHz, $CDCl_3$) δ: 166.45, 156.46, 140.07, 136.97, 128.72, 128.71, 127.07, 119.34, 117.75, 46.74, 33.08, 26.73, 26.30; IR υ_{max}/cm^{-1} (film): 3060, 2923, 2849, 2360, 2341, 1568, 1445, 1157, 1026, 758, 774, 691; m/z LRMS (ESI + APCI): $[M+H]^+$ calculated for $C_{17}H_{20}N^+$ = 238.2, found 238.2.

Two-pot synthesis:

Prepared according to general procedure E using **1a** (283 mg, 0.50 mmol), *t*BuOOH (90% in H_2O; 138 µL, 1.25 mmol), bromocyclohexane (124 *µL*, 1.00 mmol), $(TMS)_3SiH$ (309 µL, 1.00 mmol), and MeCN:TFE (1:1; 10.0 mL, 0.05 M). The reaction mixture was placed in the white LED photoreactor setup with stirring for 17 hours. After the stated workup procedure, the crude material was purified by a flash chromatography column (silica gel: 0 to 3% Et_2O in hexanes) to provide the title compound as a colorless oil (112 mg, 0.47 mmol, 94% yield).

The spectroscopic data matches the previously reported synthesis.[13]

2-Isopropyl-6-phenylpyridine (3b)

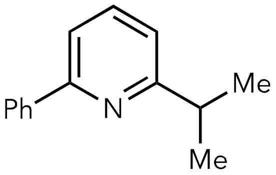

Prepared according to general procedure E using **1a** (283 mg, 0.50 mmol), *t*BuOOH (90% in H_2O; 138 µL, 1.25 mmol), 2-bromopropane (94.0 µL, 1.00 mmol), $(TMS)_3SiH$ (309 µL, 1.00 mmol), and MeCN:TFE (1:1; 10.0 mL, 0.05 M). The reaction mixture was placed in the white LED photoreactor setup with stirring for 19 hours. After the modified workup procedure of concentrating the crude reaction mixture in CH_2Cl_2 with 20.0 mL 1.00 M aqueous NaOH, the crude material was purified by a flash chromatography column (silica gel: 0 to 5% Et_2O in hexanes) to provide the title compound as a colorless oil (93.0 mg, 0.47 mmol, 94% yield). ^{1}H NMR (400 MHz, $CDCl_3$) δ: 8.09 (d, J = 7.0 Hz, 2H), 7.67 (t, J = 7.7 Hz, 1H), 7.56 (dd, J = 7.8, 1.0 Hz, 1H), 7.49 (t, J = 7.4 Hz, 2H), 7.45 – 7.39 (m, 1H), 7.13 (dd, J = 7.7, 1.0 Hz, 1H), 3.18 (hept, J = 6.9 Hz, 1H), 1.40 (d, J = 6.9 Hz, 6H); ^{13}C NMR (100 MHz, $CDCl_3$) δ: 167.22, 156.41, 140.01, 137.03, 128.75, 128.73, 127.05, 119.01, 117.70, 36.61, 22.78; IR υ_{max}/cm^{-1} (film): 3061, 2961, 2868, 2360, 2342,

1570, 1444, 1161, 1026, 815, 759, 691; m/z LRMS (ESI + APCI): $[M+H]^+$ calculated for $C_{14}H_{16}N^+$ = 198.1, found 198.2.

The spectroscopic data matches the previously reported synthesis.[14]

2-(Heptan-4-yl)-6-phenylpyridine (3c)

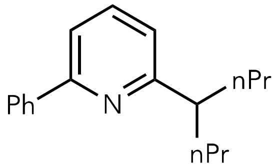

Prepared according to general procedure E using **1a** (283 mg, 0.50 mmol), *t*BuOOH (90% in H_2O; 138 µL, 1.25 mmol), 4-bromoheptane (157 µL, 1.00 mmol), $(TMS)_3SiH$ (309 µL, 1.00 mmol), and MeCN:TFE (1:1; 10.0 mL, 0.05 M). The reaction mixture was placed in the white LED photoreactor setup with stirring for 21 hours. After the stated workup procedure, the crude material was purified by a flash chromatography column (silica gel: 0 to 3% Et_2O in hexanes) to provide the title compound as a colorless oil (99.0 mg, 0.39 mmol, 78% yield). ^1H NMR (400 MHz, $CDCl_3$) δ: 8.08 (d, *J* = 7.0 Hz, 2H), 7.64 (t, *J* = 7.7 Hz, 1H), 7.55 (dd, *J* = 7.9, 1.0 Hz, 1H), 7.49 (t, *J* = 7.5 Hz, 2H), 7.42 (d, *J* = 7.3 Hz, 1H), 7.04 (dd, *J* = 7.6, 1.0 Hz, 1H), 2.85 (tt, *J* = 9.1, 5.4 Hz, 1H), 1.83 (dtd, *J* = 13.2, 9.4, 5.4 Hz, 2H), 1.68 (ddt, *J* = 13.3, 9.9, 5.8 Hz, 2H), 1.38 – 1.12 (m, 4H), 0.91 (t, *J* = 7.3 Hz, 6H); ^{13}C NMR (100 MHz, $CDCl_3$) δ: 165.52, 156.50, 140.15, 136.55, 128.72, 128.71, 127.04, 121.00, 117.56, 47.64, 38.09, 20.86, 14.40; IR υ_{max}/cm^{-1} (film): 3060, 2954, 2927, 2870, 2360, 2341, 1569, 1445, 1159, 1026, 814, 758, 691; m/z LRMS (ESI + APCI): $[M+H]^+$ calculated for $C_{18}H_{24}N^+$ = 254.2, found 254.2.

2-Cyclopropyl-6-phenylpyridine (3d)

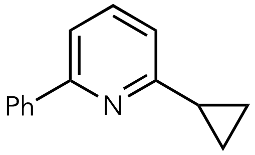

Prepared according to general procedure E using **1a** (283 mg, 0.50 mmol), *t*BuOOH (90% in H_2O; 138 µL, 1.25 mmol), bromocyclopropane (80.0 µL, 1.00 mmol), $(TMS)_3SiH$ (309 µL, 1.00 mmol), and MeCN:TFE (1:1; 10.0 mL, 0.05 M). The reaction mixture was placed in the white LED photoreactor setup with stirring for 19 hours. After the stated workup procedure, the crude material was purified by a flash chromatography column (silica gel: 0 to 5% Et_2O in hexanes) to provide the title compound as a colorless oil (30.0 mg, 0.15 mmol, 31% yield). ^1H NMR (400 MHz, $CDCl_3$) δ: 8.02 (d, *J* = 7.0 Hz, 2H), 7.59 (t, *J* = 7.7 Hz, 1H), 7.52 – 7.42 (m, 3H), 7.42 – 7.32 (m, 1H), 7.07 (dd, *J* = 7.6, 1.0 Hz, 1H), 2.10 (tt, *J* = 8.2, 4.8 Hz, 1H), 1.19 –

1.08 (m, 2H), 1.06 – 0.93 (m, 2H); ^{13}C NMR (100 MHz, $CDCl_3$) δ: 162.57, 156.42, 139.79, 136.59, 128.82, 128.69, 126.94, 119.79, 117.01, 17.36, 10.02; IR υ_{max}/cm^{-1} (film): 3060, 3004, 2924, 2852, 2360, 2342, 1570, 1452, 1161, 1026, 909, 758, 691; m/z LRMS (ESI + APCI): $[M+H]^+$ calculated for $C_{14}H_{14}N^+$ = 196.1, found 196.1.

The spectroscopic data matches the previously reported synthesis.[15]

2-Cyclobutyl-6-phenylpyridine (3e)

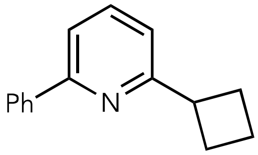

Prepared according to general procedure E using **1a** (283 mg, 0.50 mmol), *t*BuOOH (90% in H_2O; 138 µL, 1.25 mmol), bromocyclobutane (94.0 µL, 1.00 mmol), $(TMS)_3SiH$ (309 µL, 1.00 mmol), and MeCN:TFE (1:1; 10.0 mL, 0.05 M). The reaction mixture was placed in the white LED photoreactor setup with stirring for 19 hours. After the stated workup procedure, the crude material was purified by a flash chromatography column (silica gel: 0 to 5% Et_2O in hexanes) to provide the title compound as a colorless oil (69.0 mg, 0.33 mmol, 66% yield). ^{1}H NMR (400 MHz, $CDCl_3$) δ: 8.09 (dd, J = 8.3, 1.4 Hz, 2H), 7.66 (t, J = 7.8 Hz, 1H), 7.55 (dd, J = 7.9, 1.0 Hz, 1H), 7.53 – 7.47 (m, 2H), 7.47 – 7.37 (m, 1H), 7.11 (dd, J = 7.7, 1.0 Hz, 1H), 3.77 (p, J = 8.7 Hz, 1H), 2.55 – 2.35 (m, 4H), 2.18 – 2.04 (m, 1H), 2.03 – 1.90 (m, 1H); ^{13}C NMR (100 MHz, $CDCl_3$) δ: 164.63, 156.49, 139.94, 136.88, 128.80, 128.74, 127.07, 119.47, 117.65, 42.46, 28.61, 18.49; IR υ_{max}/cm^{-1} (film): 3059, 2976, 2935, 2861, 2360, 2342, 1568, 1444, 1158, 1028, 814, 756, 691; m/z LRMS (ESI + APCI): $[M+H]^+$ calculated for $C_{15}H_{16}N^+$ = 210.1, found 210.2.

The spectroscopic data matches the previously reported synthesis.[16]

2-Cyclopentyl-6-phenylpyridine (3f)

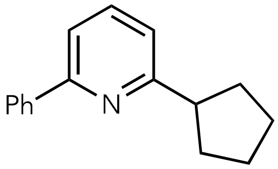

Prepared according to general procedure E using **1a** (283 mg, 0.50 mmol), *t*BuOOH (90% in H_2O; 138 µL, 1.25 mmol), bromocyclopentane (101 µL, 1.00 mmol), $(TMS)_3SiH$ (309 µL, 1.00 mmol), and MeCN:TFE (1:1; 10.0 mL, 0.05 M). The reaction mixture was placed in the white LED photoreactor setup with stirring for 17 hours. After the stated workup procedure, the crude material was purified by a flash chromatography column (silica gel: 0 to 5% Et_2O in hexanes) to provide the title compound as a colorless oil (97.0 mg, 0.43 mmol, 87% yield). ^{1}H NMR (400 MHz, $CDCl_3$) δ: 8.09 (d, J = 7.0 Hz, 2H), 7.65 (t, J = 7.7 Hz, 1H), 7.55

(dd, J = 7.8, 1.0 Hz, 1H), 7.52 – 7.46 (m, 2H), 7.46 – 7.37 (m, 1H), 7.13 (dd, J = 7.6, 1.0 Hz, 1H), 3.38 – 3.21 (m, 1H), 2.24 – 2.07 (m, 2H), 2.01 – 1.83 (m, 4H), 1.82 – 1.69 (m, 2H); ^{13}C NMR (100 MHz, $CDCl_3$) δ: 165.70, 156.37, 139.96, 136.82, 128.73, 128.70, 127.01, 120.08, 117.53, 48.12, 33.67, 26.00; IR $υ_{max}$/cm^{-1} (film): 3059, 2949, 2865, 2359, 2342, 1569, 1444, 1026, 758, 691; m/z LRMS (ESI + APCI): $[M+H]^+$ calculated for $C_{16}H_{18}N^+$ = 224.1, found 224.1.
The spectroscopic data matches the previously reported synthesis.[17]

4-(6-phenylpyridin-2-yl)cyclohexan-1-one (3g)

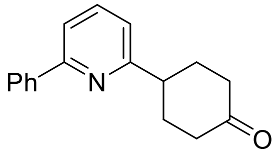

Prepared according to general procedure D using **1a** (283 mg, 0.50 mmol), *t*BuOOH (5.5 M in decane; 227 µL, 1.25 mmol), 4-bromocyclohexan-1-one (443 mg, 2.50 mmol), $(TMS)_3SiH$ (771 µL, 2.50 mmol), and MeCN:TFE (1:1; 10.0 mL, 0.05 M). The reaction mixture was placed in the white LED photoreactor setup with stirring for 17 hours. After the stated workup procedure, the crude material was purified by a flash chromatography column (silica gel: 10% EtOAc hexanes) to provide the title compound as a colorless oil (62.0 mg, 0.25 mmol, 50% yield). ^{1}H NMR (400 MHz, $CDCl_3$) δ: 8.08 – 7.99 (m, 2H), 7.68 (t, J = 7.7 Hz, 1H), 7.58 (dd, J = 7.9, 1.0 Hz, 1H), 7.51 – 7.43 (m, 2H), 7.43 – 7.35 (m, 1H), 7.12 (dd, J = 7.6, 1.0 Hz, 1H), 3.25 (tt, J = 11.1, 3.7 Hz, 1H), 2.68 – 2.43 (m, 4H), 2.41 – 2.28 (m, 2H), 2.25 – 2.10 (m, 2H); ^{13}C NMR (100 MHz, CDCl3) δ: 211.48, 163.37, 156.59, 139.48, 137.26, 128.94, 128.73, 126.90, 119.53, 118.20, 44.07, 40.94, 32.38; m/z LRMS (ESI + APCI): $[M+H]^+$ calculated for $C_{17}H_{18}NO^+$ = 252.1 found 252.1.

2-Phenyl-6-(tetrahydro-2*H*-pyran-4-yl)pyridine (3h)

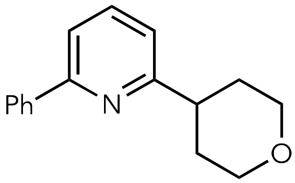

Prepared according to general procedure E using **1a** (283 mg, 0.50 mmol), *t*BuOOH (90% in H_2O; 138 µL, 1.25 mmol), 4-bromotetrahydro-2*H*-pyran (112 µL, 1.00 mmol), $(TMS)_3SiH$ (309 µL, 1.00 mmol), and MeCN:TFE (1:1; 10.0 mL, 0.05 M). The reaction mixture was placed in the white LED photoreactor setup with stirring for 21 hours. After the stated workup procedure, the crude material was purified by a flash chromatography column (silica gel: 5 to 12% Et_2O in hexanes) to provide the title compound as a colorless oil (87.0 mg, 0.36 mmol, 73% yield). ^{1}H NMR (400 MHz, $CDCl_3$) δ: 8.05 (d, J = 7.0 Hz, 2H), 7.68 (t, J =

7.8 Hz, 1H), 7.58 (dd, $J = 7.9, 0.9$ Hz, 1H), 7.51 – 7.44 (m, 2H), 7.44 – 7.36 (m, 1H), 7.10 (dd, $J = 7.7, 0.9$ Hz, 1H), 4.18 – 4.08 (m, 2H), 3.59 (td, $J = 11.6, 2.6$ Hz, 2H), 3.04 (tt, $J = 11.5, 4.3$ Hz, 1H), 2.11 – 1.89 (m, 4H); ^{13}C NMR (100 MHz, $CDCl_3$) δ: 164.25, 156.62, 139.70, 137.24, 128.90, 128.76, 127.01, 119.26, 118.08, 68.27, 43.53, 32.54; IR υ_{max}/cm^{-1} (film): 3061, 2951, 2843, 2360, 1569, 1446, 1385, 1237, 1126, 1084, 908, 761, 729, 693; m/z LRMS (ESI + APCI): $[M+H]^+$ calculated for $C_{16}H_{18}NO^+ = 240.1$ found 240.2.

Benzyl 4-(6-phenylpyridin-2-yl)piperidine-1-carboxylate (3i)

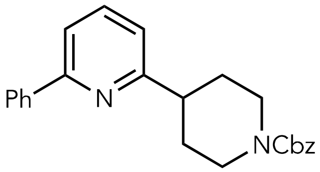

Prepared according to general procedure E using **1a** (283 mg, 0.50 mmol), *t*BuOOH (90% in H_2O; 138 μL, 1.25 mmol), benzyl 4-bromopiperidine-1-carboxylate (217 μL, 1.00 mmol), $(TMS)_3SiH$ (309 μL, 1.00 mmol), and MeCN:TFE (1:1; 10.0 mL, 0.05 M). The reaction mixture was placed in the white LED photoreactor setup with stirring for 17 hours. After the stated workup procedure, the crude material was purified by a flash chromatography column (silica gel: 0 to 40% Et_2O in hexanes) to provide the title compound as a colorless oil (119 mg, 0.32 mmol, 64% yield). ^{1}H NMR (400 MHz, $CDCl_3$) δ: 8.04 (d, J = 7.5 Hz, 2H), 7.68 (t, J = 7.7 Hz, 1H), 7.58 (d, J = 7.8 Hz, 1H), 7.48 (t, J = 7.5 Hz, 2H), 7.45 – 7.28 (m, 6H), 7.08 (d, J = 7.7 Hz, 1H), 5.19 (s, 2H), 4.37 (s, 2H), 2.97 (d, J = 12.7 Hz, 3H), 2.02 (d, J = 13.1 Hz, 2H), 1.87 (d, J = 12.9 Hz, 2H); ^{13}C NMR (100 MHz, $CDCl_3$) δ: 163.96, 156.64, 155.41, 139.60, 137.25, 137.04, 128.91, 128.74, 128.56, 128.01, 127.95, 126.98, 119.40, 118.17, 67.11, 44.41, 44.39, 31.68; IR υ_{max}/cm^{-1} (film): 2941, 2853, 2360, 2342, 1691, 1569, 1445, 1429, 1225, 1120, 1016, 761, 694; m/z LRMS (ESI + APCI): $[M+H]^+$ calculated for $C_{24}H_{25}N_2O_2^+ = 373.2$, found 373.2.

2-((1*S*,2*S*,5*R*)-2-Isopropyl-5-methylcyclohexyl)-6-phenylpyridine (3j)

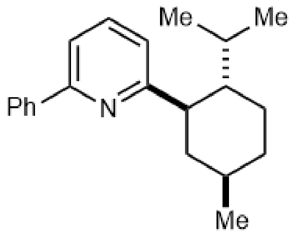

>20:1

Prepared according to general procedure E using **1a** (283 mg, 0.50 mmol), *t*BuOOH (90% in H_2O; 138 μL, 1.25 mmol), (*1S*,2*S*,4*R*)-2-bromo-1-isopropyl-4-methylcyclohexane (219 mg, 1.00 mmol), $(TMS)_3SiH$ (309 μL, 1.00 mmol), and MeCN:TFE (1:1; 10.0 mL, 0.05 M). The reaction mixture was placed in the white

LED photoreactor setup with stirring for 21 hours. After the stated workup procedure, the crude material was purified by a flash chromatography column (silica gel: 0 to 10% Et_2O in hexanes) followed by preparative thin layer chromatography (1% Et_2O in hexanes) to provide the title compound as a colorless oil (36.0 mg, 0.12 mmol, 25% yield). ^1H NMR (400 MHz, $CDCl_3$) δ: 8.03 (d, *J* = 7.0 Hz, 2H), 7.64 (t, *J* = 7.7 Hz, 1H), 7.52 (dd, *J* = 7.8, 1.0 Hz, 1H), 7.47 (t, *J* = 7.4 Hz, 2H), 7.39 (t, *J* = 7.3 Hz, 1H), 7.05 (dd, *J* = 7.6, 1.0 Hz, 1H), 2.77 (td, *J* = 11.4, 3.5 Hz, 1H), 1.92 – 1.72 (m, 4H), 1.61 – 1.47 (m, 1H), 1.46 – 1.29 (m, 2H), 1.21 (qd, *J* = 13.0, 3.1 Hz, 1H), 1.15 – 1.01 (m, 1H), 0.92 (d, *J* = 6.5 Hz, 3H), 0.84 (d, *J* = 7.0 Hz, 3H), 0.76 (d, *J* = 6.9 Hz, 3H); ^{13}C NMR (100 MHz, $CDCl_3$) δ: 165.82, 156.58, 140.18, 136.83, 128.75, 128.71, 127.08, 120.51, 117.65, 50.18, 46.79, 43.65, 35.34, 33.16, 28.14, 24.75, 22.71, 21.60, 15.89; IR υ_{max}/cm^{-1} (film): 2951, 2918, 2867, 2847, 2359, 2342, 1589, 1569, 1445, 759, 692, 668; m/z LRMS (ESI + APCI): $[M+H]^+$ calculated for $C_{21}H_{28}N^+$ = 294.2, found 294.3.

2-(*tert*-Butyl)-6-phenylpyridine (3k)

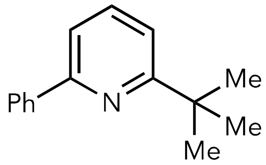

Prepared according to general procedure E using **1a** (283 mg, 0.50 mmol), *t*BuOOH (90% in H_2O; 138 µL, 1.25 mmol), 2-bromo-2-methylpropane (112 µL, 1.00 mmol), $(TMS)_3SiH$ (309 µL, 1.00 mmol), and MeCN:TFE (1:1; 10.0 mL, 0.05 M). The reaction mixture was placed in the white LED photoreactor setup with stirring for 21 hours. After the stated workup procedure, the crude material was purified by a flash chromatography column (silica gel: 0 to 3% Et_2O in hexanes) to provide the title compound as a colorless oil (94.0 mg, 0.45 mmol, 89% yield). ^1H NMR (400 MHz, $CDCl_3$) δ: 8.19 – 8.07 (m, 2H), 7.68 (t, *J* = 7.8 Hz, 1H), 7.58 (dd, *J* = 7.8, 0.9 Hz, 1H), 7.53 – 7.46 (m, 2H), 7.45 – 7.39 (m, 1H), 7.29 (dd, *J* = 7.8, 0.9 Hz, 1H), 1.47 (s, 9H); ^{13}C NMR (100 MHz, $CDCl_3$) δ: 169.08, 155.48, 140.04, 136.88, 128.76, 128.71, 126.97, 117.46, 116.98, 37.87, 30.39; IR υ_{max}/cm^{-1} (film): 3063, 2954, 2901, 2863, 1570, 1478, 1443, 1359, 1163, 1145, 816, 760, 692; m/z LRMS (ESI + APCI): $[M+H]^+$ calculated for $C_{15}H_{18}N^+$ = 212.1, found 212.2.
The spectroscopic data matches the previously reported synthesis.[18]

2-(Adamantan-1-yl)-6-phenylpyridine (3l)

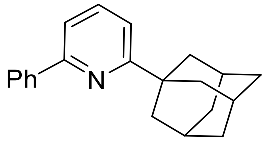

Prepared according to general procedure E using **1a** (283 mg, 0.50 mmol), *t*BuOOH (90% in H_2O; 138 µL, 1.25 mmol), 1-bromoadamantane (215 mg, 1.00 mmol), $(TMS)_3SiH$ (309 µL, 1.00 mmol), and MeCN:TFE (1:1; 10.0 mL, 0.05 M). The reaction mixture was placed in the white LED photoreactor setup with stirring for 16 hours. After the stated workup procedure, the crude material was purified by a flash chromatography column (silica gel: 0 to 5% Et_2O in hexanes) to provide the title compound as a colorless oil (139 mg, 0.48 mmol, 96% yield). ^1H NMR (400 MHz, $CDCl_3$) δ: 8.13 (d, *J* = 7.0 Hz, 2H), 7.68 (t, *J* = 7.8 Hz, 1H), 7.56 (d, *J* = 7.8 Hz, 1H), 7.48 (dd, *J* = 8.4, 6.8 Hz, 2H), 7.41 (d, *J* = 7.3 Hz, 1H), 7.21 (d, *J* = 7.8 Hz, 1H), 2.15 (p, *J* = 4.1 Hz, 3H), 2.11 (d, *J* = 2.9 Hz, 6H), 1.84 (t, *J* = 3.1 Hz, 6H); ^{13}C NMR (100 MHz, $CDCl_3$) δ: 168.86, 155.61, 140.13, 136.89, 128.71, 128.70, 126.98, 117.28, 117.09, 42.13, 39.41, 37.07, 29.02; IR υ_{max}/cm^{-1} (film): 2902, 2848, 2359, 2340, 1588, 1570, 1443, 904, 759, 728, 692; m/z LRMS (ESI + APCI): $[M+H]^+$ calculated for $C_{15}H_{18}N^+$ = 290.2, found 290.2.
The spectroscopic data matches the previously reported synthesis.[19]

2-Butyl-6-phenylpyridine (3m)

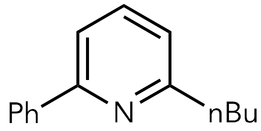

Prepared according to general procedure E using **1a** (283 mg, 0.50 mmol), *t*BuOOH (90% in H_2O; 138 µL, 1.25 mmol), 1-bromobutane (108 µL, 1.00 mmol), $(TMS)_3SiH$ (309 µL, 1.00 mmol), and MeCN:TFE (1:1; 10.0 mL, 0.05 M). The reaction mixture was placed in the white LED photoreactor setup with stirring for 19 hours. After the stated workup procedure, the crude material was purified by a flash chromatography column (silica gel: 0 to 5% Et_2O in hexanes) to provide the title compound as a colorless oil (65.0.mg, 0.31 mmol, 62%). ^1H NMR (400 MHz, $CDCl_3$) δ: 8.05 – 7.98 (m,2H), 7.65 (t, *J* = 7.7 Hz, 1H), 7.53 (dd, *J* = 7.9, 1.0 Hz, 1H), 7.51 – 7.44 (m, 2H), 7.44 – 7.36 (m, 1H), 7.09 (dd, *J* = 7.7, 1.0 Hz, 1H), 2.93 – 2.84 (m, 2H), 1.89 – 1.73 (m, 2H), 1.52 – 1.40 (m, 2H), 0.99 (t, *J* = 7.4 Hz, 3H); ^{13}C NMR (100 MHz, $CDCl_3$) δ: 162.50, 156.86, 139.92, 136.97, 128.79, 128.77, 127.14, 121.11, 117.82, 38.34, 32.07, 22.66, 14.14; IR υ_{max}/cm^{-1} (film): 3060, 2954, 2927, 2857, 2360, 2342, 1590, 1570, 1445, 1026, 757, 692; m/z LRMS (ESI + APCI): $[M+H]^+$ calculated for $C_{15}H_{18}N^+$ = 212.1, found 212.2.

2-Octyl-6-phenylpyridine (3n)

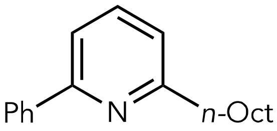

Prepared according to general procedure E using **1a** (283 mg, 0.50 mmol), *t*BuOOH (90% in H_2O; 138 μL, 1.25 mmol), 1-bromooctane (154 μL, 1.00 mmol), $(TMS)_3SiH$ (309 μL, 1.00 mmol), and MeCN:TFE (1:1; 10.0 mL, 0.05 M). The reaction mixture was placed in the white LED photoreactor setup with stirring for 19 hours. After the stated workup procedure, the crude material was purified by a flash chromatography column (silica gel: 0 to 5% Et_2O in hexanes) to provide the title compound as a colorless oil (85.0 mg, 0.32 mmol, 64% yield). ^1H NMR (400 MHz, $CDCl_3$) δ: 8.02 (d, *J* = 7.0 Hz, 2H), 7.64 (t, *J* = 7.7 Hz, 1H), 7.53 (dd, *J* = 7.8, 1.0 Hz, 1H), 7.51 – 7.44 (m, 2H), 7.43 – 7.37 (m, 1H), 7.09 (dd, *J* = 7.6, 1.0 Hz, 1H), 2.92 – 2.77 (m, 2H), 1.88 – 1.72 (m, 2H), 1.49 – 1.22 (m, 10H), 0.96 – 0.83 (m, 3H); ^{13}C NMR (100 MHz, $CDCl_3$) δ: 162.56, 156.89, 140.03, 136.89, 128.75, 128.68, 127.12, 121.08, 117.77, 38.70, 32.02, 29.93, 29.65, 29.59, 29.41, 22.81, 14.24; IR υ_{max}/cm^{-1} (film): 3060, 2923, 2853, 2360, 2341, 1590, 1570, 1457, 1445, 1026, 756, 691; m/z LRMS (ESI + APCI): $[M+H]^+$ calculated for $C_{19}H_{26}N^+$ = 268.2, found 268.2.

3-(6-Phenylpyridin-2-yl)propan-1-ol (3o)

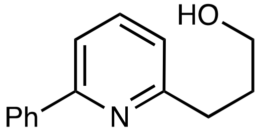

Prepared according to general procedure E using **1a** (283 mg, 0.50 mmol), *t*BuOOH (90% in H_2O; 138 μL, 1.25 mmol), 3-bromopropan-1-ol (90.0 μL, 1.00 mmol), $(TMS)_3SiH$ (309 μL, 1.00 mmol), and MeCN:TFE (1:1; 10.0 mL, 0.05 M). The reaction mixture was placed in the white LED photoreactor setup with stirring for 23 hours. After the stated workup procedure, the crude material was purified by a flash chromatography column (silica gel: 50 to 60% EtOAc in hexanes) to provide the title compound as a colorless oil (36.0 mg, 0.17 mmol, 34% yield). ^1H NMR (400 MHz, $CDCl_3$) δ: 7.93 (d, *J* = 7.0 Hz, 2H), 7.67 (t, *J* = 7.7 Hz, 1H), 7.54 (d, *J* = 7.9 Hz, 1H), 7.47 (t, *J* = 7.3 Hz, 2H), 7.41 (d, *J* = 7.2 Hz, 1H), 7.12 (d, *J* = 7.6 Hz, 1H), 4.38 (*br* s, 1H), 3.74 (t, *J* = 5.9 Hz, 2H), 3.04 (t, *J* = 6.7 Hz, 2H), 2.05 (p, *J* = 5.9 Hz, 2H); ^{13}C NMR (100 MHz, $CDCl_3$) δ: 161.40, 157.01, 139.41, 137.61, 129.05, 128.92, 127.12, 121.68, 118.47, 62.47, 35.55, 31.38; IR υ_{max}/cm^{-1} (film): 3325, 3060, 2924, 2863, 1590, 1569, 1445, 1154, 1057, 921, 811, 757, 693; m/z LRMS (ESI + APCI): $[M+H]^+$ calculated for $C_{14}H_{16}NO^+$ = 214.1, found 214.1.

Methyl 5-(6-phenylpyridin-2-yl)pentanoate (3p)

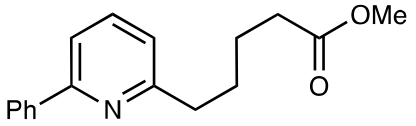

Prepared according to general procedure E using **1a** (283 mg, 0.50 mmol), *t*BuOOH (90% in H_2O; 138 μL, 1.25 mmol), methyl 5-bromopentanoate (143 μL, 1.00 mmol), $(TMS)_3SiH$ (309 μL, 1.00 mmol), and MeCN:TFE (1:1; 10.0 mL, 0.05 M). The reaction mixture was placed in the white LED photoreactor setup with stirring for 23 hours. After the stated workup procedure, the crude material was purified by a flash chromatography column (silica gel: 5 to 7.5% EtOAc in hexanes) to provide the title compound as a colorless oil (38.0 mg, 0.14 mmol, 29% yield). ^{1}H NMR (400 MHz, $CDCl_3$) δ: 8.00 (d, *J* = 7.1 Hz, 2H), 7.64 (t, *J* = 7.7 Hz, 1H), 7.53 (d, *J* = 7.8 Hz, 1H), 7.46 (t, *J* = 7.3 Hz, 2H), 7.40 (d, *J* = 7.2 Hz, 1H), 7.08 (d, *J* = 7.5 Hz, 1H), 3.67 (s, 3H), 2.88 (t, *J* = 7.6 Hz, 2H), 2.39 (t, *J* = 7.4 Hz, 2H), 1.86 (tt, *J* = 8.6, 7.0 Hz, 2H), 1.80 – 1.64 (m, 2H); ^{13}C NMR (100 MHz, $CDCl_3$) δ: 174.22, 161.71, 156.95, 139.89, 137.00, 128.81, 128.76, 127.08, 121.14, 117.92, 51.58, 38.13, 34.09, 29.17, 24.78; IR υ_{max}/cm^{-1} (film): 3060, 2947, 2859, 2359, 2341, 1734, 1590, 1570, 1445, 1197, 1170, 759, 693; m/z LRMS (ESI + APCI): $[M+H]^+$ calculated for $C_{17}H_{20}NO_2^+$ = 270.1, found 270.1.

5-(6-phenylpyridin-2-yl)pentanenitrile (3q)

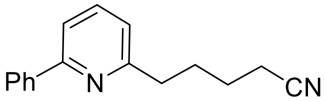

Prepared according to general procedure D using **1a** (283 mg, 0.50 mmol), *t*BuOOH (5.5 M in decane; 227 μL, 1.25 mmol), 5-bromopentanenitrile (292 μL, 2.50 mmol), $(TMS)_3SiH$ (771 μL, 2.50 mmol), and MeCN:TFE (1:1; 10.0 mL, 0.05 M). The reaction mixture was placed in the white LED photoreactor setup with stirring for 36 hours. After the stated workup procedure, the crude material was purified by a flash chromatography column (silica gel: 10% EtOAc in hexanes) to provide the title compound as a pale-yellow oil (20.0 mg, 0.085 mmol, 17% yield). ^{1}H NMR (400 MHz, CDCl3) δ: 8.09 – 7.96 (m, 2H), 7.67 (t, *J* = 7.7 Hz, 1H), 7.56 (dd, *J* = 7.9, 1.0 Hz, 1H), 7.51 – 7.43 (m, 2H), 7.43 – 7.35 (m, 1H), 7.09 (dd, *J* = 7.6, 1.0 Hz, 1H), 2.91 (t, *J* = 7.5 Hz, 2H), 2.41 (t, *J* = 7.2 Hz, 2H), 2.08 – 1.90 (m, 2H), 1.85 – 1.72 (m, 2H); ^{13}C NMR (100 MHz, $CDCl_3$) δ:f 160.87, 157.04, 139.66, 137.26, 128.99, 128.84, 127.08, 121.23, 119.85, 118.20, 37.34, 28.54, 25.08, 17.19; m/z LRMS (ESI + APCI): $[M+H]^+$ calculated for $C_{16}H_{17}N_2^+$ = 237.1 found 237.2.

2-(Pent-4-en-1-yl)-6-phenylpyridine (3r)

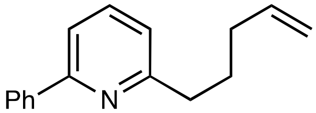

Prepared according to general procedure E using **1a** (283 mg, 0.50 mmol), *t*BuOOH (90% in H_2O; 138 μL, 1.25 mmol), 5-bromopent-1-ene (119 μL, 1.00 mmol), $(TMS)_3SiH$ (309 μL, 1.00 mmol), and MeCN:TFE (1:1; 10.0 mL, 0.05 M). The reaction mixture was placed in the white LED photoreactor setup with stirring for 23 hours. After the stated workup procedure, the crude material was purified by a flash chromatography column (silica gel: 1 to 3% Et_2O in hexanes) followed by preparative thin layer chromatography (silica gel: 100% hexanes) to provide the title compound as a colorless oil (22.0 mg, 0.10 mmol, 20% yield). ^{1}H NMR (400 MHz, $CDCl_3$) δ: 8.01 (d, J = 7.2 Hz, 2H), 7.65 (t, J = 7.7 Hz, 1H), 7.54 (d, J = 7.8 Hz, 1H), 7.47 (t, J = 7.5 Hz, 2H), 7.41 (d, J = 7.1 Hz, 1H), 7.08 (d, J = 7.5 Hz, 1H), 5.89 (ddt, J = 16.9, 10.2, 6.7 Hz, 1H), 5.06 (dd, J = 17.1, 1.9 Hz, 1H), 5.00 (dd, J = 10.1, 1.9 Hz, 1H), 2.93 – 2.81 (m, 2H), 2.18 (q, J = 7.2 Hz, 2H), 1.93 (p, J = 7.7 Hz, 2H); ^{13}C NMR (100 MHz, $CDCl_3$) δ: 162.11, 156.97, 139.97, 138.78, 136.97, 128.82, 128.79, 127.12, 121.21, 117.89, 114.89, 38.00, 33.59, 29.04; IR $υ_{max}/cm^{-1}$ (film): 3062, 2925, 2855, 2360, 2341, 1590, 1570, 1457, 1445, 990, 909, 756, 692; m/z LRMS (ESI + APCI): $[M+H]^+$ calculated for $C_{16}H_{18}N^+$ = 224.1, found 224.1.

diethyl (4-((6-phenylpyridin-2-yl)methyl)benzyl)phosphonate (3s)

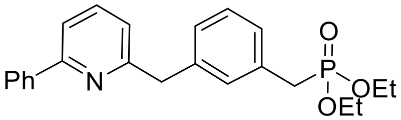

Prepared according to general procedure D using **1a** (283 mg, 0.50 mmol), *t*BuOOH (5.5 M in decane; 227 μL, 1.25 mmol), diethyl (4-(bromomethyl)benzyl)phosphonate (321 mg, 1.00 mmol), $(TMS)_3SiH$ (309 μL, 1.00 mmol), and MeCN:TFE (1:1; 10.0 mL, 0.05 M). The reaction mixture was placed in the white LED photoreactor setup with stirring for 17 hours. After the stated workup procedure, the crude material was purified by a flash chromatography column (silica gel: 97% DCM in EtOH) followed by addition of 1.0 equivalent of 2.0 M HCl in Et_2O. The crude material was diluted with CH_2Cl_2 and washed with $NaHCO_3$ x2. The aqueous layers were extracted with CH_2Cl_2 x2 and combined to do a brine wash x1. The resulting crude material was subjected to a preparatory plate chromatography with (1:1:8 EtOAc:EtOH:hexanes) to provide the title compound as a white solid oil (41.0 mg, 0.11 mmol, 21% yield). ^{1}H NMR (400 MHz, $CDCl_3$) δ: 8.01 (d, J = 7.1 Hz, 2H), 7.62 (t, J = 7.7 Hz, 1H), 7.54 (d, J = 7.8 Hz, 1H), 7.46 (t, J = 7.3 Hz, 2H), 7.40 (t, J = 7.3 Hz, 1H), 7.30 – 7.21 (m, 3H), 7.18 (dd, J = 7.1, 2.0 Hz, 1H), 7.01 (d, J = 7.6 Hz, 1H), 4.21 (s, 2H), 4.02 – 3.90 (m, 4H), 3.13 (d, J = 21.6 Hz, 2H), 1.18 (t, J = 7.1 Hz, 6H); ^{13}C NMR (100 MHz,

$CDCl_3$) δ: 160.87, 156.97, 140.09 (d, *J* = 3.0 Hz), 139.70, 137.23, 131.94 (d, *J* = 9.1 Hz), 130.79 (d, *J* = 6.7 Hz), 128.92, 128.83 (d, *J* = 3.4 Hz), 128.79, 127.96 (d, *J* = 1.5 Hz), 127.91 (d, *J* = 3.9 Hz), 127.10, 121.45, 118.07, 62.20 (d, *J* = 6.8 Hz), 44.93, 33.86 (d, *J* = 138.0 Hz), 16.45 (d, *J* = 5.9 Hz); ^{31}P NMR (162 MHz, $CDCl_3$) δ: 26.38; m/z LRMS (ESI + APCI): $[M+H]^+$ calculated for $C_{23}H_{27}NO_3P^+$ = 396.2 found 396.3.

2-Cyclohexyl-6-isopropylpyridine (3t)

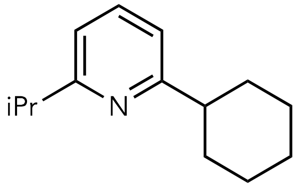

Prepared according to general procedure E using **1t** (2.66 g, 5.00 mmol), *t*BuOOH (90% in H_2O; 1.38 mL, 12.5 mmol), bromocyclohexane (1.24 mL, 10.0 mmol), $(TMS)_3SiH$ (3.09 mL, 10.0 mmol), and MeCN:TFE (1:1; 100 mL, 0.05 M). The reaction mixture was placed in the white LED photoreactor setup with stirring for 21 hours. After the stated workup procedure, the crude material was purified by a flash chromatography column (silica gel: 0 to 5% Et_2O in hexanes). After collecting all fractions that contained the product, HCl (4.00M in dioxane; 3.00 mL) was added. The collected fractions were concentrated *in vacuo* and the residue heated to 40 °C under vacuum to provide the HCl salt of the title compound as a yellow solid (1.06 g, 4.40 mmol, 88% yield). Spectroscopic data was collected of the free base of the title compound. ^{1}H NMR (400 MHz, $CDCl_3$) δ: 7.51 (t, *J* = 7.7 Hz, 1H), 7.05 – 6.72 (m, 2H), 3.04 (hept, *J* = 6.9 Hz, 1H), 2.74 – 2.63 (m, 1H), 1.99 – 1.92 (m, 2H), 1.88 – 1.81 (m, 2H), 1.75 (ddtd, *J* = 12.5, 4.8, 3.1, 1.6 Hz, 1H), 1.56 – 1.36 (m, 5H), 1.29 (d, *J* = 7.0 Hz, 6H); ^{13}C NMR (100 MHz, $CDCl_3$) δ: 166.56, 165.76, 136.69, 117.74, 117.28, 46.71, 36.55, 33.21, 26.73, 26.32, 22.86; IR υ_{max}/cm^{-1} (film): 2960, 2924, 2851, 2360, 2342, 1587, 1575, 1449, 1158, 806, 748; m/z LRMS (ESI + APCI): $[M+H]^+$ calculated for $C_{14}H_{22}N^+$ = 204.2, found 204.2.

2-Cyclohexyl-6-methoxypyridine (3u)

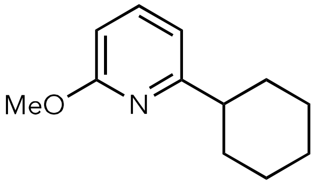

Prepared according to general procedure E using **1u** (260 mg, 0.50 mmol), *t*BuOOH (90% in H_2O; 138 µL, 1.25 mmol), bromocyclohexane (124 µL, 1.00 mmol), $(TMS)_3SiH$ (309 µL, 1.00 mmol), and MeCN:TFE (1:1; 10.0 mL, 0.05). The reaction mixture was placed in the white LED photoreactor setup with stirring for 19 hours. After the stated workup procedure, the crude material was purified by a flash chromatography

column (silica gel: 0 to 5% Et_2O in hexanes) to provide the title compound as a colorless oil (78.0 mg, 0.41 mmol, 82% yield). ^{1}H NMR (400 MHz, $CDCl_3$) δ: 7.46 (dd, J = 8.2, 7.3 Hz, 1H), 6.70 (d, J = 7.3 Hz, 1H), 6.53 (d, J = 8.2 Hz, 1H), 3.92 (s, 3H), 2.59 (tt, J = 11.7, 3.5 Hz, 1H), 1.94 (ddd, J = 12.3, 3.6, 1.7 Hz, 2H), 1.84 (dt, J = 12.5, 3.2 Hz, 2H), 1.74 (dtt, J = 11.2, 3.3, 1.6 Hz, 1H), 1.53 (qd, J = 12.4, 3.0 Hz, 2H), 1.40 (qt, J = 12.2, 3.0 Hz, 2H), 1.34 – 1.21 (m, 1H); ^{13}C NMR (100 MHz, $CDCl_3$) δ: 164.66, 163.57, 138.79, 113.35, 107.28, 53.17, 46.11, 32.81, 26.68, 26.32; IR υ_{max}/cm^{-1} (film): 2924, 2851, 2360, 2341, 1577, 1461, 1412, 1311, 1284, 1256, 1046, 798, 736, 702; m/z LRMS (ESI + APCI): $[M+H]^+$ calculated for $C_{12}H_{18}NO^+$= 192.1, found 192.2.
The spectroscopic data matches the previously reported synthesis.[20]

2-Butyl-6-cyclohexyl-3-fluoropyridine (3v)

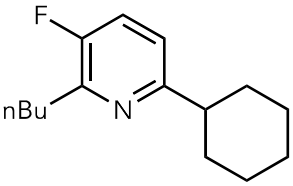

Prepared according to general procedure E using **1v** (282 mg, 0.50 mmol), *t*BuOOH (90% in H_2O; 138 µL, 1.25 mmol), bromocyclohexane (124 µL, 1.00 mmol), $(TMS)_3SiH$ (309 µL, 1.00 mmol), and MeCN:TFE (1:1; 10.0 mL, 0.05 M). The reaction mixture was placed in the white LED photoreactor setup with stirring for 21 hours. After the stated workup procedure, the crude material was purified by a flash chromatography column (silica gel: 0 to 3% Et_2O in hexanes) to provide the title compound as a yellow oil (80.0 mg, 0.34 mmol, 68% yield). ^{1}H NMR (400 MHz, $CDCl_3$) δ: 7.19 (dd, J = 9.5, 8.4 Hz, 1H), 6.93 (dd, J = 8.5, 3.7 Hz, 1H), 2.80 (td, J = 7.9, 2.5 Hz, 2H), 2.67 (hd, J = 9.1, 4.7 Hz, 1H), 1.99 – 1.88 (m, 2H), 1.87 – 1.78 (m, 2H), 1.78 – 1.60 (m, 3H), 1.47 – 1.33 (m, 6H), 1.32 – 1.14 (m, 1H), 0.92 (t, J = 7.4 Hz, 3H); ^{13}C NMR (100 MHz, $CDCl_3$) δ: 161.67 (d, J = 5.0 Hz), 156.17 (d, J = 252.2 Hz), 149.35 (d, J = 15.2 Hz), 122.61 (d, J = 19.6 Hz), 118.81 (d, J = 3.4 Hz), 46.02 (d, J = 1.4 Hz), 33.35, 31.73 (d, J = 2.3 Hz), 31.09 (d, J = 1.4 Hz), 26.68, 26.21, 22.70, 14.05; ^{19}F NMR (376 MHz, $CDCl_3$) δ: -131.43 (d, J = 9.24 Hz); IR υ_{max}/cm^{-1} (film): 2925, 2852, 2359, 2342, 1597, 1462, 1239, 1121, 824; m/z LRMS (ESI + APCI): $[M+H]^+$ calculated for $C_{15}H_{23}FN^+$ = 236.2, found 236.2.

6-Butyl-2-cyclohexyl-3-(methoxymethyl)pyridine (3w)

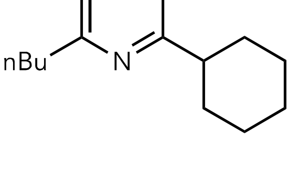

Prepared according to general procedure E using **1w** (295 mg, 0.50 mmol), *t*BuOOH (90% in H_2O; 138 µL, 1.25 mmol), bromocyclohexane (124 µL, 1.00 mmol), $(TMS)_3SiH$ (309 µL, 1.00 mmol), and MeCN:TFE (1:1; 10.0 mL, 0.05 M). The reaction mixture was placed in the white LED photoreactor setup with stirring for 17 hours. After the stated workup procedure, the crude material was purified by a flash chromatography column (silica gel: 0 to 5% Et_2O in hexanes) to provide the title compound as a colorless oil (56.0 mg, 0.21 mmol, 43% yield). ^{1}H NMR (400 MHz, $CDCl_3$) δ: 7.47 (d, J = 7.8 Hz, 1H), 6.90 (d, J = 7.8 Hz, 1H), 4.45 (s, 2H), 3.40 (s, 3H), 2.86 – 2.65 (m, 3H), 1.91 – 1.80 (m, 2H), 1.72 (m, 7H), 1.38 (p, J = 7.7 Hz, 5H), 0.93 (t, J = 7.4 Hz, 3H); ^{13}C NMR (100 MHz, $CDCl_3$) δ: 163.74, 161.18, 136.66, 126.81, 119.39, 71.81, 58.35, 41.93, 38.03, 32.30, 31.96, 26.90, 26.19, 22.55, 14.15; IR υ_{max}/cm^{-1} (film): 2923, 2851, 2359, 2342, 1591, 1571, 1449, 1191, 1098, 832; m/z LRMS (ESI + APCI): $[M+H]^+$ calculated for $C_{17}H_{28}NO^+$ = 262.2, found 262.2.

2-Cyclohexyl-3-phenylpyridine and 2-cyclohexyl-5-phenylpyridine (3x)

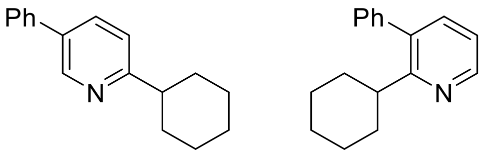

Major

Minor

6.3:1.0 (major:minor)

Prepared according to general procedure E using **1x** (282 mg, 0.50 mmol), *t*BuOOH (90% in H_2O; 138 µL, 1.25 mmol), bromocyclohexane (124 µL, 1.00 mmol), $(TMS)_3SiH$ (309 µL, 1.00 mmol) MeCN:TFE (1:1; 10.0 mL, 0.05 M). The reaction mixture was placed in the white LED photoreactor setup with stirring for 21 hours. After the stated workup procedure, mesitylene (70.0 µL, 0.50 mmol) was added as an internal standard for ^{1}H NMR analysis which displayed a 6.3:1.0:2.0 mixture of isomeric products **3x** (**major**):**3x** (**minor**):**3x'.** The crude material was purified by a flash chromatography column (silica gel: 5 to 50% Et_2O in hexanes) to provide a 5.6:1 as an inseparable mixture of **3x** (**major**)**: 3x** (**minor**)**.** The mixture was isolated as a white solid (69.0 mg, 0.29 mmol, 58% combined yield). mp: 47–54 °C; ^{1}H NMR (Major; 400 MHz, $CDCl_3$) δ: 8.76 (d, J = 2.4 Hz, 1H), 7.79 (dd, J = 8.1, 2.5 Hz, 1H), 7.56 (d, J = 7.0 Hz, 2H), 7.45 (dd, J = 8.4, 6.7 Hz, 2H), 7.40 – 7.34 (m, 1H), 7.22 (d, J = 8.1 Hz, 1H), 2.76 (tt, J = 11.8, 3.4 Hz, 1H), 2.07 – 1.97 (m, 2H), 1.88 (dt, J = 12.5, 3.3 Hz, 2H), 1.82 – 1.68 (m, 1H), 1.57 (qd, J = 12.3, 3.0 Hz, 2H), 1.44 (qt, J = 12.5, 3.1 Hz, 2H), 1.31 (ddt, J = 25.0, 12.7, 3.4 Hz, 1H); ^{13}C NMR (Major; 100 MHz, $CDCl_3$) δ: 165.47, 147.53, 138.15, 134.95, 134.09, 129.10, 127.82, 127.09, 120.98, 46.33, 33.07, 26.71, 26.19; IR υ_{max}/cm^{-1} (film): 2922, 2850, 2360, 2342, 1595, 1473, 1447, 1005, 773, 752, 695; m/z LRMS (ESI + APCI): $[M+H]^+$ calculated for $C_{17}H_{20}N^+$ = 238.2, found 238.1.

2,6-Dicyclohexyl-3-phenylpyridine (3x')

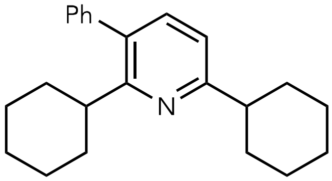

The title compound was prepared according to the synthesis of **3x** (see above). The crude material was purified by a flash chromatography column (silica gel: 0 to 5% Et_2O in hexanes) to provide the title compound as a white solid (25.0 mg, 0.08 mmol, 16% yield). mp: 101–103 °C; ^{1}H NMR (400 MHz, $CDCl_3$) δ: 7.42 (t, *J* = 7.3 Hz, 2H), 7.37 (d, *J* = 7.5 Hz, 2H), 7.29 (d, *J* = 7.4 Hz, 2H), 6.96 (d, *J* = 7.9 Hz, 1H), 2.74 (dd, *J* = 13.4, 9.6 Hz, 2H), 2.00 (d, *J* = 13.0 Hz, 2H), 1.87 (d, *J* = 13.4 Hz, 2H), 1.76 (q, *J* = 11.3 Hz, 5H), 1.70 – 1.52 (m, 5H), 1.51 – 1.39 (m, 2H), 1.37 – 1.24 (m, 2H), 1.18 (q, *J* = 13.1 Hz, 2H); ^{13}C NMR (100 MHz, $CDCl_3$) δ: 164.75, 162.22, 140.83, 137.63, 133.09, 129.40, 128.29, 127.02, 117.36, 46.31, 42.09, 33.05, 32.76, 26.77, 26.60, 26.42, 26.15; IR υ_{max}/cm^{-1} (film): 2923, 2850, 2360, 2342, 1587, 1558, 1449, 700; m/z LRMS (ESI + APCI): $[M+H]^+$ calculated for $C_{23}H_{30}N^+$ = 320.2, found 320.3.

5-(((6-Cyclohexylpyridin-2-yl)oxy)methyl)-3-phenylisoxazole (3y)

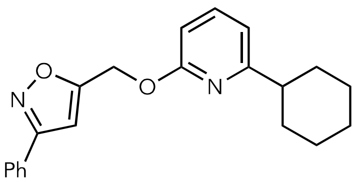

Prepared according to general procedure E using **1y** (331 mg, 0.50 mmol), *t*BuOOH (90% in H_2O; 138 µL, 1.25 mmol), bromocyclohexane (124 µL, 1.00 mmol), $(TMS)_3SiH$ (309 µL, 1.00 mmol), and MeCN:TFE (1:1; 10.0 mL, 0.05 M). The reaction mixture was placed in the white LED photoreactor setup with stirring for 17 hours. After the stated workup procedure, the crude material was purified by a flash chromatography column (silica gel: 0 to 5% Et_2O in hexanes) to provide the title compound as a colorless oil (49.0 mg, 0.15 mmol, 29% yield). ^{1}H NMR (400 MHz, $CDCl_3$) δ: 7.80 (tq, *J* = 5.5, 2.3 Hz, 2H), 7.52 (dd, *J* = 8.2, 7.3 Hz, 1H), 7.44 (dd, *J* = 5.1, 2.0 Hz, 3H), 6.76 (d, *J* = 7.3 Hz, 1H), 6.65 – 6.58 (m, 2H), 5.55 (s, 2H), 2.60 (tt, *J* = 11.7, 3.5 Hz, 1H), 1.93 (dqd, *J* = 11.8, 3.3, 1.9 Hz, 2H), 1.85 (dt, *J* = 12.4, 3.2 Hz, 2H), 1.75 (dddd, *J* = 12.5, 4.9, 3.1, 1.5 Hz, 1H), 1.53 (qd, *J* = 12.3, 2.9 Hz, 2H), 1.40 (qt, *J* = 12.4, 3.0 Hz, 2H), 1.34 – 1.20 (m, 1H); ^{13}C NMR (100 MHz, $CDCl_3$) δ: 169.76, 164.46, 162.52, 161.79, 139.33, 130.08, 129.21, 129.02, 126.95, 114.54, 108.00, 101.44, 58.03, 45.98, 32.79, 26.62, 26.29; IR υ_{max}/cm^{-1} (film): 2924, 2850, 2359, 2342, 1595, 1574, 1440, 1405, 1278, 1252, 1029, 9907, 798, 766, 691; m/z LRMS (ESI + APCI): $[M+H]^+$ calculated for $C_{21}H_{23}N_2O_2^+$ = 335.2, found 335.2.

2-(3-Chloro-4-((2-fluorobenzyl)oxy)phenyl)-6-cyclohexylpyridine (3z)

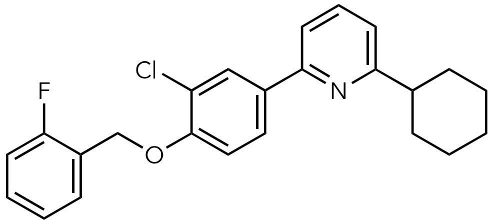

Prepared according to general procedure E using **1z** (362 mg, 0.50 mmol), *t*BuOOH (90% in H_2O; 138 μL, 1.25 mmol), bromocyclohexane(124 μL, 1.00 mmol), $(TMS)_3SiH$ (309 μL, 1.00 mmol), and MeCN:TFE (1:1; 10.0 mL, 0.05 M). The reaction mixture was placed in the white LED photoreactor setup with stirring for 19 hours. After the stated workup procedure, the crude material was purified by a flash chromatography column (silica gel: 0 to 7% Et_2O in hexanes) to provide the title compound as a colorless oil (147 mg, 0.37 mmol, 74% yield). ^{1}H NMR (400 MHz, $CDCl_3$) δ: 8.13 (d, J = 2.2 Hz, 1H), 7.88 (dd, J = 8.6, 2.2 Hz, 1H), 7.62 (t, J = 7.7 Hz, 2H), 7.44 (dd, J = 7.8, 1.0 Hz, 1H), 7.32 (tdd, J = 7.5, 5.3, 1.8 Hz, 1H), 7.19 (td, J = 7.5, 1.2 Hz, 1H), 7.13 – 7.09 (m, 1H), 7.09 – 7.02 (m, 2H), 5.29 (s, 2H), 2.76 (tt, J = 11.8, 3.5 Hz, 1H), 2.07 – 1.98 (m, 2H), 1.88 (dt, J = 12.9, 3.4 Hz, 2H), 1.78 (dtd, J = 11.2, 3.2, 1.6 Hz, 1H), 1.59 (qd, J = 12.4, 3.1 Hz, 2H), 1.45 (qt, J = 12.4, 3.2 Hz, 2H), 1.32 (ddt, J = 25.2, 12.7, 3.7 Hz, 1H); ^{13}C NMR (100 MHz, $CDCl_3$) δ: 166.54, 160.29 (d, J = 246.5 Hz), 154.70, 154.39, 137.08, 134.18, 129.78 (d, J = 8.2 Hz), 129.38 (d, J = 3.9 Hz), 129.06, 126.30, 124.52 (d, J = 3.6 Hz), 123.87, 123.73, 119.33, 117.11, 115.35 (d, J = 21.0 Hz), 113.90, 64.64 (d, J = 4.7 Hz), 46.72, 33.06, 26.72, 26.28; ^{19}F NMR (376 MHz, $CDCl_3$) δ: -118.81 (dt, J = 12.6, 6.3 Hz); IR υ_{max}/cm^{-1} (film): 2924, 2850, 2360, 2342, 1536, 1502, 1448, 1382, 1267, 1232, 1058, 1005, 798, 754; m/z LRMS (ESI + APCI): $[M+H]^+$ calculated for $C_{24}H_{24}ClFNO^+$ = 396.2, found 396.2.

Ethyl 2-cyclohexyl-6-(4-(4,4,5,5-tetramethyl-1,3,2-dioxaborolan-2-yl)phenyl)nicotinate (3aa)

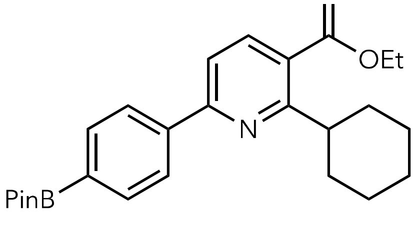

Prepared according to general procedure E using **1aa** (382 mg, 0.50 mmol), *t*BuOOH (90% in H_2O; 138 μL, 1.25 mmol), bromocyclohexane (124 μL, 1.00 mmol), $(TMS)_3SiH$ (309 μL, 1.00 mmol), and MeCN:TFE (1:1; 10.0 mL, 0.05 M). The reaction mixture was placed in the white LED photoreactor setup with stirring for 17 hours. After the stated workup procedure, the crude material was purified by a flash chromatography column (silica gel: 0 to 15% Et_2O in hexanes) to provide the title compound as a white

solid (62.0 mg, 0.14 mmol, 29% yield). mp: 48–54 °C; ^{1}H NMR (400 MHz, $CDCl_3$) δ: 8.12 (dd, J = 11.0, 8.2 Hz, 3H), 7.92 (d, J = 8.0 Hz, 2H), 7.62 (d, J = 8.2 Hz, 1H), 4.40 (q, J = 7.1 Hz, 2H), 3.56 (tt, J = 10.8, 3.4 Hz, 1H), 1.91 – 1.68 (m, 7H), 1.51 – 1.41 (m, 6H), 1.37 (m, 12H); ^{13}C NMR (100 MHz, $CDCl_3$) δ: 167.39, 166.55, 158.25, 141.39, 139.07, 135.27, 126.55, 123.80, 116.92, 116.81, 84.02, 61.30, 43.26, 32.64, 26.84, 26.35, 25.01, 14.42; IR υ_{max}/cm^{-1} (film): 2978, 2926, 2851, 2360, 2342, 1718, 1583, 1448, 1358, 1257, 1143, 1098, 858; m/z LRMS (ESI + APCI): $[M+H]^+$ calculated for $C_{26}H_{35}BNO_4^+$ = 436.3, found 436.3.

(*S*)-2-cyclohexyl-5-(1-methylpyrrolidin-2-yl)pyridine (3ab)

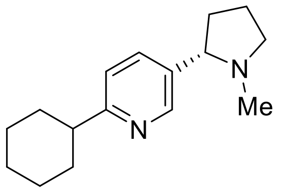

Prepared according to a modified procedure D using **1ab** (286 mg, 0.25 mmol), *t*BuOOH (5.5 M in decane; 114 µL, 0.625 mmol), bromocyclohexane (62.0 µL, 0.50 mmol), $(TMS)_3SiH$ (155 µL, 0.50 mmol), TfOH (22.0 µL, 0.25 mmol), and MeCN:TFE (1:1; 5.0 mL, 0.05 M). The reaction mixture was placed in the white LED photoreactor setup with stirring for 14 hours. An aliquot of the reaction was assayed via ^{1}H NMR and showed to be an 8:1 mixture of regioisomers. The reaction was washed with hexanes (x6), concentrated *in vacuo*, and redissolved in MeOH: H_2O (9:1; 1.5 mL, 0.30 M). K_2CO_3 was added (346 mg, 2.5 mmol) and the reaction was heated at 40 ºC for 1 hour. The reaction was diluted with CH_2Cl_2 (5.00 mL, 0.05 M) and dried over magnesium sulfate. The resulting crude material was purified by a flash chromatography column (silica gel: 76:19:5 hexanes:acetone:AcOH to 80:20 hexanes:acetone) to provide the title compound as a colorless oil (48.0 mg, 0.198 mmol, 79% yield). ^{1}H NMR (400 MHz, $CDCl_3$) δ: 8.39 (d, J = 2.2 Hz, 1H), 7.62 (dd, J = 8.1, 2.3 Hz, 1H), 7.11 (d, J = 8.1 Hz, 1H), 3.24 (td, J = 8.6, 2.1 Hz, 1H), 3.06 (t, J = 8.2 Hz, 1H), 2.67 (tt, J = 11.8, 3.4 Hz, 1H), 2.28 (q, J = 9.0 Hz, 1H), 2.15 (s, 4H), 1.94 (tdd, J = 13.0, 4.6, 2.2 Hz, 3H), 1.82 (ddt, J = 11.2, 6.9, 4.4 Hz, 3H), 1.77 (s, 2H), 1.56 – 1.27 (m, 4H), 1.28 – 1.20 (m, 1H); ^{13}C NMR (100 MHz, $CDCl_3$) δ: 165.66, 148.81, 135.44, 121.05, 68.84, 57.03, 46.32, 40.38, 34.95, 33.08, 33.07, 26.71, 26.19, 22.57; m/z LRMS (ESI + APCI): $[M+H]^+$ calculated for $C_{16}H_{25}N_2^+$ = 245.2 found 245.2.

2-Cyclohexyl-6-((1-(4-phenoxyphenoxy)propan-2-yl)oxy)pyridine (3ac)

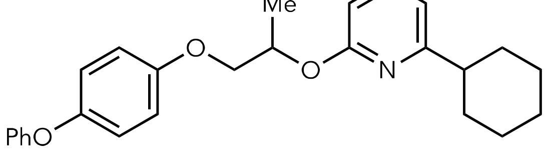

Prepared according to general procedure E using **1ac** (366 mg, 0.50 mmol), *t*BuOOH (90% in H_2O; 138 μL, 1.25 mmol), bromocyclohexane (124 μL, 1.00 mmol), $(TMS)_3SiH$ (309 μL, 1.00 mmol), and MeCN:TFE (1:1; 10.0 mL, 0.05 M). The reaction mixture was placed in the white LED photoreactor setup with stirring for 21 hours. After the stated workup procedure, the crude material was purified by a flash chromatography column (silica gel: 0 to 5% Et_2O in hexanes) to provide the title compound as a colorless oil (130 mg, 0.32 mmol, 64% yield). ^{1}H NMR (400 MHz, $CDCl_3$) δ: 7.49 (t, *J* = 7.8 Hz, 1H), 7.32 (t, *J* = 7.7 Hz, 2H), 7.06 (t, *J* = 7.4 Hz, 1H), 7.03 – 6.89 (m, 6H), 6.72 (d, *J* = 7.3 Hz, 1H), 6.56 (d, *J* = 8.3 Hz, 1H), 5.65 (h, *J* = 6.0 Hz, 1H), 4.27 (dd, *J* = 10.0, 5.1 Hz, 1H), 4.08 (dd, *J* = 9.9, 5.4 Hz, 1H), 2.60 (tt, *J* = 11.9, 3.4 Hz, 1H), 1.96 (d, *J* = 12.7 Hz, 2H), 1.86 (d, *J* = 12.8 Hz, 2H), 1.76 (d, *J* = 12.7 Hz, 1H), 1.61 – 1.50 (m, 4H), 1.48 – 1.17 (m, 4H); ^{13}C NMR (100 MHz, $CDCl_3$) δ: 164.41, 162.52, 158.58, 155.38, 150.41, 139.01, 129.72, 122.56, 120.80, 117.80, 115.94, 113.54, 108.34, 71.21, 68.84, 46.07, 32.89, 32.78, 26.68, 26.67, 26.30, 17.29; IR υ_{max}/cm^{-1} (film): 2924, 2851, 2360, 2342, 1589, 1575, 1503, 1487, 1445, 1282, 1217, 1043, 977, 798, 733, 690; m/z LRMS (ESI + APCI): $[M+H]^+$ calculated for $C_{26}H_{30}NO_3^+$ = 404.2, found 404.3.

2-cyclohexylquinoline (3ad)

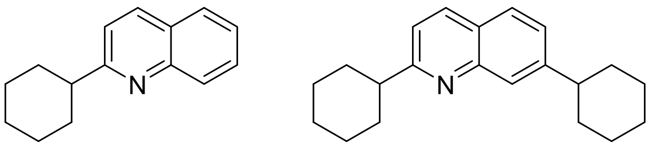

Major   Minor

Prepared according to general procedure D using **1ad** (270 mg, 0.50 mmol), *t*BuOOH (5.5 M in decane; 227 μL, 1.25 mmol), bromocyclohexane (124 μL, 1.00 mmol), $(TMS)_3SiH$ (309 μL, 1.00 mmol), and MeCN:TFE (1:1; 10.0 mL, 0.05 M). The reaction mixture was placed in the white LED photoreactor setup with stirring for 17 hours. After the stated workup procedure, mesitylene (70.0 μL 0.50 mmol) was added as an internal standard for ^{1}H NMR analysis which displayed an overlapping mixture of isomeric products **3ad (major)** and **3ad (minor)**. The crude material was purified by a flash chromatography column (silica gel: 15% Et_2O in hexanes) to provide the title compounds as an inseparable mixture of alkylated products **3ad (major): 3ad (minor)** 1.0:0.11. The mixture was isolated as a pale-yellow oil (51.2 mg, 0.24 mmol, 48% yield). ^{1}H NMR (Major; 400 MHz, $CDCl_3$) δ: 8.11 – 7.99 (m, 2H), 7.74 (dd, *J* = 8.1, 1.5 Hz, 1H), 7.65 (ddd, *J* = 8.4, 6.9, 1.5 Hz, 1H), 7.45 (ddd, *J* = 8.1, 6.9, 1.2 Hz, 1H), 7.30 (d, *J* = 8.6 Hz, 1H), 2.92 (tt, *J* = 12.0, 3.5 Hz, 1H), 2.02 (ddq, *J* = 12.6, 5.4, 2.0 Hz, 2H), 1.88 (dt, *J* = 12.8, 3.3 Hz, 2H), 1.78 (dtt, *J* = 12.8, 3.2, 1.5 Hz, 1H), 1.62 (qd, *J* = 12.4, 2.8 Hz, 2H), 1.53 – 1.39 (m, 2H), 1.39 – 1.29 (m, 1H); ^{13}C NMR (Major; 100 MHz, $CDCl_3$) δ: 166.85, 129.64, 128.74, 127.58, 127.11, 125.95, 124.59, 124.10, 119.69, 32.95, 31.56, 26.62, 26.19; m/z LRMS (ESI + APCI): $[M+H]^+$ calculated for $C_{15}H_{18}N^+$ (title compound) =

212.1 found 212.2; m/z LRMS (ESI + APCI): $[M+H]^+$ calculated for $C_{21}H_{28}N^+$ (doubly alkylated product) = 294.2 found 294.2.

2-Cyclohexyl-6-methoxyquinoline (3ae)

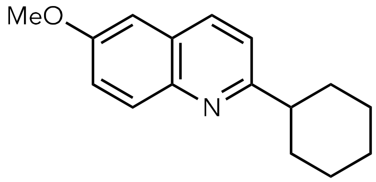

Prepared according to general procedure E using **1ae** (285 mg, 0.50 mmol), *t*BuOOH (90% in H_2O; 138 μL, 1.25 mmol), bromocyclohexane (124 μL, 1.00 mmol), $(TMS)_3SiH$ (309 μL, 1.00 mmol), and MeCN:TFE (1:1; 10.0 mL, 0.05 M). The reaction mixture was placed in the white LED photoreactor setup with stirring for 17 hours. After the stated workup procedure, mesitylene (70.0 μL 0.50 mmol) was added as an internal standard for ^{1}H NMR analysis which displayed a 1.5:1 mixture of isomeric products **3ae** and **3ae'** (see below). The crude material was purified by a flash chromatography column (silica gel: 0 to 10% Et_2O in hexanes) to provide the title compound as a yellow oil (58.0 mg, 0.24 mmol, 48% yield). ^{1}H NMR (400 MHz, $CDCl_3$) δ: 7.95 (t, *J* = 8.4 Hz, 2H), 7.32 (dd, *J* = 9.2, 2.8 Hz, 1H), 7.27 (d, *J* = 8.6 Hz, 1H), 7.03 (d, *J* = 2.9 Hz, 1H), 3.90 (s, 3H), 2.88 (tt, *J* = 12.0, 3.5 Hz, 1H), 2.04 – 1.96 (m, 2H), 1.88 (dt, *J* = 12.7, 3.2 Hz, 2H), 1.81 – 1.73 (m, 1H), 1.60 (qd, *J* = 12.4, 3.1 Hz, 2H), 1.54 – 1.39 (m, 2H), 1.34 (tt, *J* = 12.7, 3.4 Hz, 1H); ^{13}C NMR (100 MHz, $CDCl_3$) δ: 164.49, 157.25, 143.91, 135.28, 130.47, 127.87, 121.81, 119.86, 105.26, 55.59, 47.47, 33.06, 26.70, 26.24; IR υ_{max}/cm^{-1} (film): 2923, 2849, 2360, 2342, 1623, 1600, 1559, 1498, 1448, 1237, 1225, 1159, 1031, 849, 832; m/z LRMS (ESI + APCI): $[M+H]^+$ calculated for $C_{16}H_{20}NO^+$ = 242.2, found 242.2.
The spectroscopic data matches the previously reported synthesis.[21]

2,7-Dicyclohexyl-6-methoxyquinoline (3ae')

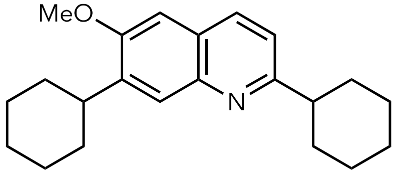

The title compound was prepared according to the synthesis of **3ae** (see above). The crude material was purified by a flash chromatography column (silica gel: 0 to 5% Et_2O in hexanes) to provide the title compound as a yellow oil (41.0 mg, 0.13 mmol, 25% yield). ^{1}H NMR (400 MHz, $CDCl_3$) δ: 7.94 (d, *J* = 8.5 Hz, 1H), 7.84 (s, 1H), 7.22 (d, *J* = 8.4 Hz, 1H), 6.98 (s, 1H), 3.93 (s, 3H), 3.06 (tt, *J* = 10.9, 2.5 Hz, 1H), 2.93 – 2.81 (m, 1H), 2.06 – 1.98 (m, 2H), 1.95 (d, *J* = 8.3 Hz, 2H), 1.91 – 1.86 (m, 4H), 1.83 – 1.71 (m, 2H), 1.60 (qd, *J* = 12.4, 3.0 Hz, 2H), 1.54 – 1.39 (m, 6H), 1.39 – 1.18 (m, 2H); ^{13}C NMR (100 MHz, $CDCl_3$)

δ: 163.97, 155.34, 143.70, 141.45, 134.46, 126.17, 125.77, 118.63, 103.72, 55.30, 47.30, 37.12, 33.14, 32.84, 26.93, 26.44, 26.29, 25.95; IR υ_{max}/cm^{-1} (film): 2923, 2850 2359, 2342, 15999, 1495, 1473, 1448, 1242, 1224; m/z LRMS (ESI + APCI): $[M+H]^+$ calculated for $C_{22}H_{30}NO^+$ = 324.2, found 324.3.

6-Cyclohexyl-2-phenylfuro[2,3-*b*]pyridine (3af)

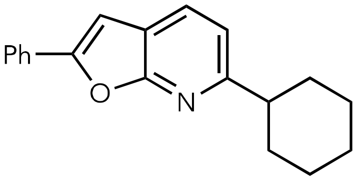

Prepared according to general procedure E using **1af** (303 mg, 0.50 mmol), *t*BuOOH (90% in H_2O; 138 μL, 1.25 mmol), bromocyclohexane (124 μL, 1.00 mmol), $(TMS)_3SiH$ (309 μL, 1.00 mmol), and MeCN:TFE (1:1; 10.0 mL, 0.05 M). The reaction mixture was placed in the white LED photoreactor setup with stirring for 16 hours. After the modified workup procedure of concentrating the crude reaction mixture in CH_2Cl_2 with 20.0 mL 1.00 M aqueous NaOH, the crude material was purified by a flash chromatography column (silica gel: 0 to 3% Et_2O in hexanes) to provide the title compound as a white solid (31.0 mg, 0.11 mmol, 22% yield). mp: 99–102 °C; ^{1}H NMR (400 MHz, $CDCl_3$) δ: 7.90 (d, *J* = 7.7 Hz, 2H), 7.80 (d, *J* = 7.8 Hz, 1H), 7.44 (t, *J* = 7.6 Hz, 2H), 7.37 (d, *J* = 7.3 Hz, 1H), 7.09 (d, *J* = 7.8 Hz, 1H), 6.96 (s, 1H), 2.78 (tt, *J* = 12.0, 3.6 Hz, 1H), 2.04 – 1.97 (m, 2H), 1.93 – 1.84 (m, 2H), 1.76 (d, *J* = 12.4 Hz, 1H), 1.65 (qd, *J* = 12.4, 3.2 Hz, 2H), 1.50 – 1.38 (m, 3H); ^{13}C NMR (100 MHz, $CDCl_3$) δ: 162.12, 161.88, 154.96, 130.08, 129.87, 129.02, 128.95, 125.10, 118.99, 117.44, 100.11, 46.53, 33.27, 26.76, 26.18; IR υ_{max}/cm^{-1} (film): 2924, 2850, 2360, 2342, 1601, 1562, 1490, 1393, 1260, 827, 755; m/z LRMS (ESI + APCI): $[M+H]^+$ calculated for $C_{19}H_{20}NO^+$ = 278.2, found 278.2.

2-(2-Cyclopentyl-6-methylpyridin-3-yl)-4-fluoroisoindoline-1,3-dione (3ag)

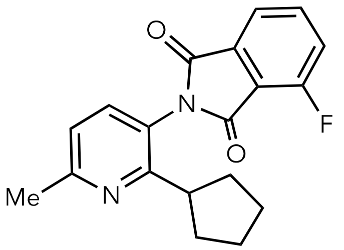

Prepared according to general procedure E using **1ag** (333 mg, 0.50 mmol), *t*BuOOH (90% in H_2O; 138 μL, 1.25 mmol), bromocyclopentane (101 μL, 1.00 mmol), $(TMS)_3SiH$ (309 μL, 1.00 mmol), and

MeCN:TFE (1:1; 10.0 mL, 0.05 M). The reaction mixture was placed in the white LED photoreactor setup with stirring for 17 hours. After the stated workup procedure, the crude material was purified by a flash chromatography column (silica gel: 0 to 50% Et_2O in hexanes) to provide the title compound as a colorless oil (44.0 mg, 0.14 mmol, 27% yield). ^{1}H NMR (400 MHz, $CDCl_3$) δ: 7.85 – 7.71 (m, 2H), 7.47 (t, J = 8.1 Hz, 1H), 7.31 (d, J = 8.0 Hz, 1H), 7.08 (d, J = 8.0 Hz, 1H), 2.96 (p, J = 8.4 Hz, 1H), 2.58 (s, 3H), 1.98 – 1.72 (m, 6H), 1.63 – 1.47 (m, 2H); ^{13}C NMR (100 MHz, $CDCl_3$) δ: 166.55 (d, J = 3.0 Hz), 164.27 (d, J = 1.5 Hz), 162.79, 159.75, 158.00 (d, J = 266.8 Hz), 137.18 (d, J = 7.6 Hz), 136.59, 134.18 (d, J = 1.4 Hz), 123.20, 122.97 (d, J = 19.7 Hz), 121.23, 120.24 (d, J = 3.7 Hz), 117.94 (d, J = 12.3 Hz), 42.49, 33.37, 33.34, 26.10, 26.09, 24.77; ^{19}F NMR (377 MHz, $CDCl_3$) δ: -111.66 (dd, J = 8.7, 4.0 Hz); IR υ_{max}/cm^{-1} (film): 2953, 2868, 2360, 2342, 1723, 1611, 1480, 1382, 1260, 1107, 972, 746; m/z LRMS (ESI + APCI): $[M+H]^+$ calculated for $C_{19}H_{18}FN_2O_2^+$ = 325.1, found 325.2.

2-Chloro-*N*-(4-chloro-3-(6-isopropylpyridin-2-yl)phenyl)-4-(methylsulfonyl)benzamide (3ah)

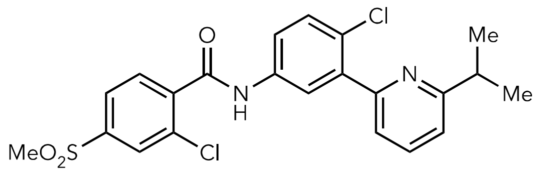

Prepared according to general procedure E using **1ah** (416 mg, 0.50 mmol), *t*BuOOH (90% in H_2O; 138 μL, 1.25 mmol), 2-bromopropane (94.0 μL, 1.00 mmol), $(TMS)_3SiH$ (309 μL, 1.00 mmol), and MeCN:TFE (1:1; 10.0 mL, 0.05 M). The reaction mixture was placed in the white LED photoreactor setup with stirring for 23 hours. After the stated workup procedure, the crude material was purified by a flash chromatography column (silica gel: 10 to 40% EtOAc in hexanes) to provide the title compound as a pale–yellow solid (207 mg, 0.45 mmol, 90% yield). mp: 170–171 °C; ^{1}H NMR (400 MHz, $CDCl_3$) δ: 8.92 (s, 1H), 7.88 – 7.77 (m, 3H), 7.73 – 7.64 (m, 2H), 7.60 (d, J = 8.0 Hz, 1H), 7.51 (dd, J = 7.8, 1.0 Hz, 1H), 7.45 (d, J = 8.6 Hz, 1H), 7.13 (dd, J = 7.9, 1.0 Hz, 1H), 3.23 – 2.77 (m, 4H), 1.25 (d, J = 6.9 Hz, 6H); ^{13}C NMR (100 MHz, $CDCl_3$) δ: 167.39, 163.58, 155.08, 142.65, 140.52, 139.69, 136.70, 136.64, 132.50, 131.01, 130.36, 129.02, 127.99, 125.81, 123.38, 122.44, 121.48, 119.22, 44.51, 36.41, 22.75; IR υ_{max}/cm^{-1} (film): 3306, 3062, 2962, 2926, 2360, 2342, 1662, 1571, 1539, 1447, 1450, 1313, 1153, 1097, 960, 814, 750; m/z LRMS (ESI + APCI): $[M+H]^+$ calculated for $C_{22}H_{21}Cl_2N_2O_3S^+$ = 463.1, found 463.1.

Ethyl 4-(8-chloro-2-cyclobutyl-5,6-dihydro-11*H*-benzo [5,6] cyclohepta[1,2-*b*]pyridin-11-ylidene) piperidine-1-carboxylate (3ai)

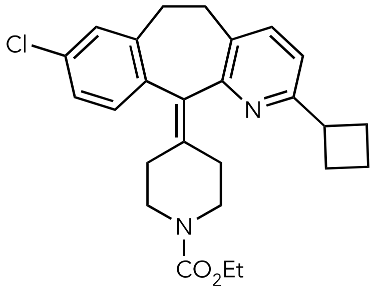

Prepared according to general procedure E using **1ai** (397 mg, 0.50 mmol), *t*BuOOH (90% in H_2O; 138 µL, 1.25 mmol), bromocyclobutane (94.0 µL, 1.00 mmol), $(TMS)_3SiH$ (309 µL, 1.00 mmol), and MeCN:TFE (1:1; 10.0 mL, 0.05 M). The reaction mixture was placed in the white LED photoreactor setup with stirring for 16 hours. After the stated workup procedure, the crude material was purified by a flash chromatography column (silica gel: 10 to 25% EtOAc in hexanes) to provide the title compound as a white solid (131 mg, 0.30 mmol, 60% yield). mp: 49–52 °C; ^{1}H NMR (400 MHz, $CDCl_3$) δ: 7.32 (d, *J* = 7.9 Hz, 1H), 7.18 – 7.08 (m, 3H), 6.99 (d, *J* = 7.8 Hz, 1H), 4.14 (q, *J* = 7.1 Hz, 2H), 3.77 (s, 2H), 3.62 (p, *J* = 8.7 Hz, 1H), 3.44 – 3.14 (m, 4H), 2.78 (ddt, *J* = 11.6, 7.3, 4.2 Hz, 2H), 2.54 (ddd, *J* = 14.0, 9.1, 4.5 Hz, 1H), 2.42 – 2.15 (m, 7H), 2.02 (dq, *J* = 10.7, 8.8 Hz, 1H), 1.86 (dtt, *J* = 11.8, 8.6, 3.2 Hz, 1H), 1.25 (t, *J* = 7.1 Hz, 3H); ^{13}C NMR (100 MHz, $CDCl_3$) δ: 161.86, 155.87, 155.66, 140.11, 138.34, 137.95, 137.39, 134.62, 132.82, 130.66, 130.19, 128.83, 126.10, 119.13, 61.41, 45.11, 45.09, 42.07, 31.88, 31.59, 31.07, 30.79, 28.95, 28.64, 18.40, 14.83; IR υ_{max}/cm^{-1} (film): 2927, 2854, 2359, 2342, 1693, 1585, 1428, 1385, 1277, 1220, 1115, 995, 837, 750, 667; m/z LRMS (ESI + APCI): $[M+H]^+$ calculated for $C_{26}H_{30}ClN_2O_2^+$ = 437.2, found 437.3.

Ethyl (*S*)-4-((4-chlorophenyl) (6-(tetrahydro-2*H*-pyran-4-yl)pyridin-2-yl)methoxy) piperidine-1-carboxylate (3aj)

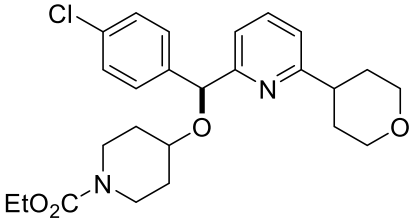

Prepared according to general procedure E using **1aj** (393 mg, 0.50 mmol), *t*BuOOH (90% in H_2O; 138 µL, 1.25 mmol), 4-bromotetrahydro-2*H*-pyran (113 µL, 1.00 mmol), $(TMS)_3SiH$ (309 µL, 1.00 mmol), and MeCN:TFE (1:1; 10.0 mL, 0.05 M). The reaction mixture was placed in the white LED photoreactor setup with stirring for 17 hours. After the stated workup procedure, the crude material was purified by a flash chromatography column (silica gel: 50 to 70% Et_2O in hexanes) to provide the title compound as a colorless

oil (140 mg, 0.31 mmol, 61% yield). ^{1}H NMR (400 MHz, $CDCl_3$) δ: 7.50 (t, *J* = 7.8 Hz, 1H), 7.29 (d, *J* = 8.5 Hz, 2H), 7.21 (d, *J* = 7.0 Hz, 1H), 7.18 – 7.14 (m, 2H), 6.93 (d, *J* = 7.7 Hz, 1H), 5.50 (s, 1H), 4.07 – 3.94 (m, 4H), 3.72 – 3.60 (m, 2H), 3.55 (tt, *J* = 7.5, 3.6 Hz, 1H), 3.44 (ddt, *J* = 14.0, 11.7, 2.1 Hz, 2H), 3.11 (dtd, *J* = 12.3, 8.2, 3.7 Hz, 2H), 2.81 (tt, *J* = 10.8, 4.9 Hz, 1H), 1.85 – 1.66 (m, 6H), 1.57 (ddtd, *J* = 16.3, 12.2, 7.9, 3.7 Hz, 2H), 1.15 (t, *J* = 7.1 Hz, 3H); ^{13}C NMR (100 MHz, $CDCl_3$) δ: 163.67, 161.36, 155.64, 140.53, 137.40, 133.20, 128.47, 128.06, 119.52, 118.12, 81.21, 72.73, 68.20, 68.16, 61.36, 43.25, 41.15, 41.12, 32.58, 32.37, 31.29, 31.11, 14.81; IR υ_{max}/cm^{-1} (film): 2947, 2846, 2360, 2342, 1693, 1589, 1573, 1451, 1431, 1272, 11085, 1029, 1014, 751; m/z LRMS (ESI + APCI): $[M+H]^+$ calculated for $C_{25}H_{32}ClN_2O_4^+$ = 459.2, found 459.3.

6-Cyclohexyl-2-methyl-3-((3-methylpyridin-2-yl)oxy)pyridine (3ak)

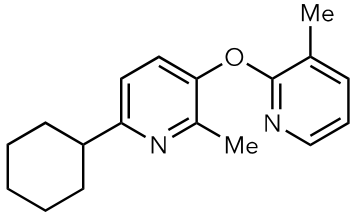

>20:1

Prepared according to general procedure E using **1ak** (305 mg, 0.50 mmol), *t*BuOOH (5.50 M in decane; 227 μL, 1.25 mmol), bromocyclohexane (124 μL, 1.00 mmol), $(TMS)_3SiH$ (309 μL, 1.00 mmol), and MeCN:TFE (1:1; 10.0 mL, 0.05 M). The reaction mixture was placed in the white LED photoreactor setup with stirring for 21 hours. After the stated workup procedure, the crude material was purified by a flash chromatography column (silica gel: 0 to 20% Et_2O in hexanes) to provide the title compound as a colorless oil (124 mg, 0.44 mmol, 88% yield). ^{1}H NMR (400 MHz, $CDCl_3$) δ: 7.92 (d, *J* = 4.8 Hz, 1H), 7.50 (d, *J* = 7.3 Hz, 1H), 7.30 (d, *J* = 1.5 Hz, 1H), 7.01 (d, *J* = 8.2 Hz, 1H), 6.87 (t, *J* = 6.9 Hz, 1H), 2.69 (td, *J* = 11.8, 3.5 Hz, 1H), 2.37 (s, 3H), 2.35 (s, 3H), 1.99 (d, *J* = 10.9 Hz, 2H), 1.87 – 1.79 (m, 2H), 1.73 (d, *J* = 12.9 Hz, 1H), 1.43 (q, *J* = 11.9 Hz, 4H), 1.32 – 1.20 (m, 1H); ^{13}C NMR (100 MHz, $CDCl_3$) δ: 162.03, 161.38, 150.38, 146.66, 144.60, 139.78, 129.50, 121.36, 118.58, 118.52, 46.24, 33.31, 26.71, 26.20, 19.61, 16.02; IR υ_{max}/cm^{-1} (film): 2923, 2850, 2360, 2341, 1587, 1447, 1413, 1276, 1234, 1181, 1158, 1119, 878, 784; m/z LRMS (ESI + APCI): $[M+H]^+$ calculated for $C_{18}H_{23}N_2O^+$ = 283.2, found 283.2.

2-Cyclohexyl-6-(2-((6-(trifluoromethyl)pyridin-2-yl)oxy)ethyl)pyridine (3al)

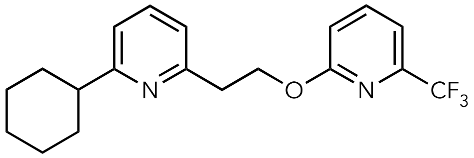

>20:1

Prepared according to general procedure E using **1al** (339 mg, 0.50 mmol), *t*BuOOH (5.50 M in decane; 227 µL, 1.25 mmol), bromocyclohexane (124 µL, 1.00 mmol), $(TMS)_3SiH$ (309 µL, 1.00 mmol), and MeCN:TFE (1:1; 10.0 mL, 0.05 M). The reaction mixture was placed in the white LED photoreactor setup with stirring for 16 hours. After the stated workup procedure, the crude material was purified by a flash chromatography column (silica gel: 0 to 10% Et_2O in hexanes) to provide the title compound as a colorless oil (107 mg, 0.30 mmol, 61% yield). ^{1}H NMR (400 MHz, $CDCl_3$) δ: 7.65 (t, *J* = 7.8 Hz, 1H), 7.52 (t, *J* = 7.8 Hz, 1H), 7.21 (d, *J* = 7.3 Hz, 1H), 7.04 (d, *J* = 7.5 Hz, 1H), 6.99 (d, *J* = 7.8 Hz, 1H), 6.86 (d, *J* = 8.4 Hz, 1H), 4.73 (t, *J* = 6.9 Hz, 2H), 3.24 (t, *J* = 6.8 Hz, 2H), 2.68 (tt, *J* = 11.7, 3.5 Hz, 1H), 2.00 – 1.92 (m, 2H), 1.89 – 1.79 (m, 2H), 1.77 – 1.66 (m, 1H), 1.56 – 1.35 (m, 4H), 1.27 (dtt, *J* = 12.9, 8.8, 4.4 Hz, 1H); ^{13}C NMR (100 MHz, $CDCl_3$) δ: 166.42, 163.91, 157.54, 145.63 (q, *J* = 34.6 Hz), 139.34, 136.70, 121.52 (q, *J* = 273.8 Hz), 120.75, 118.42, 114.77, 113.20 (q, *J* = 3.2 Hz), 66.05, 46.73, 37.91, 33.16, 26.71, 26.26; ^{19}F NMR (376 MHz, $CDCl_3$) δ: -68.45; IR υ_{max}/cm^{-1} (film): 2927, 2853, 2360, 2341, 1606, 1576, 1470, 1455, 1349, 1285, 1184, 1139, 1122, 1075, 988, 907, 810, 730; m/z LRMS (ESI + APCI): $[M+H]^+$ calculated for $C_{19}H_{22}F_3N_2O^+$ = 351.2, found 351.2.

2-Chloro-5-(((4-(6-cyclohexylpyridin-2-yl)benzyl)oxy)methyl)pyridine (3am)

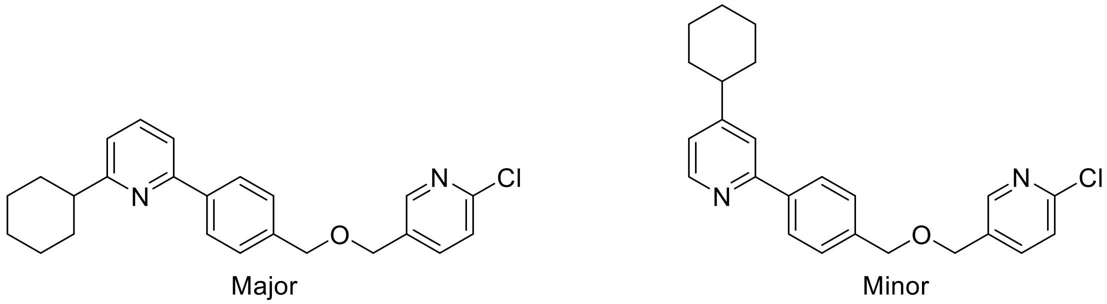

6.9:1.0 (major:minor)

Prepared according to general procedure E using **1am** (361 mg, 0.50 mmol), *t*BuOOH (90% in H_2O; 138 µL, 1.25 mmol), bromocyclohexane (124 µL, 1.00 mmol), $(TMS)_3SiH$ (309 µL, 1.00 mmol), and MeCN:TFE (1:1; 10.0 mL, 0.05 M). The reaction mixture was placed in the white LED photoreactor setup with stirring for 20 hours. After the stated workup procedure, mesitylene (70.0 µL, 0.50 mmol) was added as an internal standard for ^{1}H NMR analysis which displayed a 6.9:1.0 mixture of **3am** (**major**):**3am**

(**minor**). The crude material was purified by a flash chromatography column (silica gel: 20 to 40% Et_2O in hexanes) to provide a 10:1.0 inseparable mixture of isomers of the title compound and a doubly alkylated product as a colorless oil (89.0 mg, 0.23 mmol, 45% combined yield). ^{1}H NMR (Major; 400 MHz, $CDCl_3$) δ: 8.36 (d, *J* = 1.6 Hz, 1H), 8.04 (d, *J* = 8.3 Hz, 2H), 7.71 – 7.62 (m, 2H), 7.52 (dd, *J* = 7.8, 1.0 Hz, 1H), 7.44 (d, *J* = 8.2 Hz, 2H), 7.32 (d, *J* = 8.2 Hz, 1H), 7.09 (dd, *J* = 7.7, 1.0 Hz, 1H), 4.63 (s, 2H), 4.53 (s, 2H), 2.78 (tt, *J* = 11.9, 3.5 Hz, 1H), 2.09 – 1.98 (m, 2H), 1.87 (dt, *J* = 12.8, 3.4 Hz, 2H), 1.77 (dddd, *J* = 12.1, 4.7, 3.0, 1.4 Hz, 1H), 1.60 (qd, *J* = 12.5, 3.2 Hz, 2H), 1.44 (qt, *J* = 12.5, 3.2 Hz, 2H), 1.32 (tt, *J* = 12.6, 3.4 Hz, 1H); ^{13}C NMR (Major; 100 MHz, $CDCl_3$) δ: 166.45, 155.93, 150.79, 148.93, 139.76, 138.32, 137.87, 136.95, 132.77, 128.14, 127.18, 124.14, 119.41, 117.66, 72.41, 68.48, 46.63, 32.98, 26.62, 26.19; IR υ_{max}/cm^{-1} (film): 2923, 2850, 2359, 1588, 1567, 1449, 1349, 1093, 1017, 799, 730; m/z LRMS (ESI + APCI): $[M+H]^+$ calculated for $C_{24}H_{26}ClN_2O^+$ (title compound) = 393.2, found 393.2; $[M+H]^+$ calculated for $C_{30}H_{36}ClN_2O^+$ (doubly alkylated product) = 475.3, found 475.3.

3-((6-Chloropyridin-3-yl)methoxy)-2-cyclohexyl-6-methylpyridine (3an)

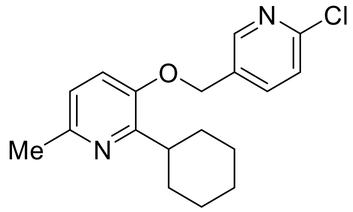

>20:1

Prepared according to general procedure E using **1an** (323 mg, 0.50 mmol), *t*BuOOH (5.50 M in decane; 227 μL, 1.25 mmol), bromocyclohexane (124 μL, 1.00 mmol), $(TMS)_3SiH$ (309 μL, 1.00 mmol), and MeCN:TFE (1:1; 10.0 mL, 0.05 M). The reaction mixture was placed in the white LED photoreactor setup with stirring for 21 hours. After the stated workup procedure, mesitylene (70.0 μL, 0.50 mmol) was added as an internal standard for ^{1}H NMR analysis which displayed a >20:1 ratio of alkylated products. The crude material was purified by a flash chromatography column (silica gel: 5 to 20% Et_2O in hexanes) to provide the title compound as a pale–yellow solid (62.0 mg, 0.20 mmol, 39% yield). mp: 92–93 °C; ^{1}H NMR (400 MHz, $CDCl_3$) δ: 8.47 (d, *J* = 2.4 Hz, 1H), 7.72 (d, *J* = 8.1 Hz, 1H), 7.37 (dd, *J* = 8.3, 1.6 Hz, 1H), 7.02 (dd, *J* = 8.3, 1.6 Hz, 1H), 6.89 (d, *J* = 8.4 Hz, 1H), 5.03 (s, 2H), 3.08 (t, *J* = 11.6 Hz, 1H), 2.46 (d, *J* = 1.7 Hz, 3H), 1.89 – 1.59 (m, 7H), 1.48 – 1.18 (m, 3H); ^{13}C NMR (100 MHz, $CDCl_3$) δ: 155.52, 151.31, 150.06, 149.32, 148.59, 137.80, 131.64, 124.45, 120.54, 119.25, 67.26, 40.09, 31.32, 26.86, 26.21, 23.82. IR υ_{max}/cm^{-1} (film): 2926, 2852, 2359, 2341, 1568, 1456, 1349, 1254, 1213, 1100, 1020, 822, 749; m/z LRMS (ESI + APCI): $[M+H]^+$ calculated for $C_{18}H_{22}ClN_2O^+$ = 317.1, found 317.2.

2-((6-Cyclohexyl-3-fluoropyridin-2-yl)methoxy)-6-(trifluoromethyl)nicotinonitrile (3ao)

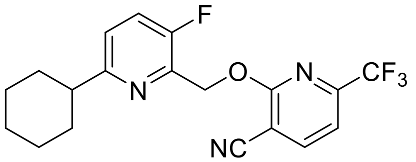

Major

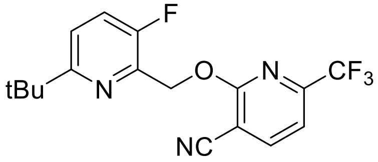

Minor

>20:1

Minor amounts of *t*Bu side pdt

Prepared according to general procedure E using **1ao** (354 mg, 0.50 mmol), *t*BuOOH (5.50 M in decane; 227 µL, 1.25 mmol), bromocyclohexane (124 µL, 1.00 mmol), $(TMS)_3SiH$ (309 µL, 1.00 mmol), and MeCN:TFE (1:1; 10.0 mL, 0.05 M). The reaction mixture was placed in the white LED photoreactor setup with stirring for 16 hours. After the stated workup procedure, mesitylene (70.0 µL, 0.50 mmol) was added as an internal standard for ^{1}H NMR analysis which displayed a 7.1:1.0 mixture of products **3ao** and **3ao'** (*tert*-butylated side product). The crude material was purified by a flash chromatography column (silica gel: 1 to 10% Et_2O in hexanes) followed by preparative thin layer chromatography (silica gel: 7 % Et_2O in hexanes) to provide the title compound as a pale–yellow oil (93.0 mg, 0.25 mmol, 49% yield). ^{1}H NMR (400 MHz, $CDCl_3$) δ: 8.00 (d, *J* = 7.7 Hz, 1H), 7.31 – 7.23 (m, 2H), 7.06 (dd, *J* = 8.6, 3.8 Hz, 1H), 5.61 (d, *J* = 2.0 Hz, 2H), 2.58 (tt, *J* = 11.5, 3.3 Hz, 1H), 1.82 – 1.76 (m, 2H), 1.73 (dp, *J* = 10.1, 2.6 Hz, 2H), 1.64 (ddd, *J* = 12.7, 3.4, 1.6 Hz, 1H), 1.39 – 1.23 (m, 4H), 1.20 – 0.98 (m, 1H); ^{13}C NMR (100 MHz, $CDCl_3$) δ: 163.59, 162.27 (d, *J* = 4.8 Hz), 156.73 (d, *J* = 257.1 Hz), 149.07 (q, *J* = 36.1 Hz), 144.73, 141.09 (d, *J* = 14.0 Hz), 123.76 (d, *J* = 18.6 Hz), 122.41 (d, *J* = 3.6 Hz), 120.48 (q, *J* = 274.7 Hz), 113.93, 113.26 (q, *J* = 3.1 Hz), 100.65, 65.85 (d, *J* = 1.9 Hz), 45.72 (d, *J* = 1.4 Hz), 33.02, 26.51, 26.10; ^{19}F NMR (376 MHz, $CDCl_3$) δ: -69.06, -130.14 (d, *J* = 9.0 Hz); IR υ_{max}/cm^{-1} (film): 2927, 2853, 2359, 2341, 1586, 1469, 1453, 1409, 1344, 1263, 1184, 1147, 1117, 1102, 990, 834; m/z LRMS (ESI + APCI): $[M+H]^+$ calculated for $C_{19}H_{18}F_4N_3O^+$ = 380.1, found 380.2.

2-((6-(*tert*-Butyl)-3-fluoropyridin-2-yl)methoxy)-6-(trifluoromethyl)nicotinonitrile (3ao')

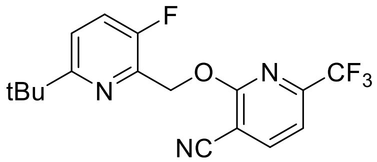

The title compound was prepared according to the synthesis of **3ao**. The crude material was purified by a flash chromatography column (silica gel: 1 to 10% Et_2O in hexanes) followed by preparative thin layer chromatography (silica gel: 7 % Et_2O in hexanes) to provide the title compound as a pale–yellow solid

(31.0 mg, 0.09 mmol, 18% yield). mp: 47–49 °C; ^{1}H NMR (400 MHz, $CDCl_3$) δ: 8.08 (dd, *J* = 7.6, 0.7 Hz, 1H), 7.35 (d, *J* = 7.7 Hz, 1H), 7.32 – 7.27 (m, 2H), 5.75 (d, *J* = 1.9 Hz, 2H), 1.21 (s, 9H); ^{13}C NMR (100 MHz, $CDCl_3$) δ: 164.72 (d, *J* = 4.6 Hz), 163.91, 156.04 (d, *J* = 256.5 Hz), 149.11 (q, *J* = 36.0 Hz), 144.67, 140.61 (d, *J* = 14.1 Hz), 123.21 (d, *J* = 18.2 Hz), 120.50 (q, *J* = 275.7 Hz), 120.17 (d, *J* = 3.5 Hz), 114.04, 113.17 (q, *J* = 3.1 Hz), 100.62, 65.44 (d, *J* = 1.9 Hz), 37.26, 30.19; ^{19}F NMR (376 MHz, $CDCl_3$) δ: -69.09, -131.80 – -131.90 (m); IR υ_{max}/cm^{-1} (film): 2960, 2928, 2855, 2360, 2341, 1586, 1466, 1430, 1344, 1263, 1184, 1149, 1118, 1102, 836; m/z LRMS (ESI + APCI): $[M+H]^+$ calculated for $C_{17}H_{16}F_4N_3O^+$ = 354.1, found 354.2.

2-Chloro-6-(1-(6-cyclohexylpyridin-2-yl)ethoxy)-5-fluoronicotinonitrile (3ap)

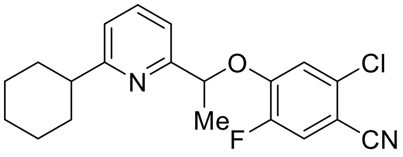

>20:1

Prepared according to general procedure E using **1ap** (344 mg, 0.50 mmol), *t*BuOOH (5.50 M in decane; 227 µL, 1.25 mmol), bromocyclohexane (124 µL, 1.00 mmol), $(TMS)_3SiH$ (309 µL, 1.00 mmol), and MeCN:TFE (1:1; 10.0 mL, 0.05 M). The reaction mixture was placed in the white LED photoreactor setup with stirring for 17 hours. After the stated workup procedure, mesitylene (70.0 µL, 0.50 mmol) was added as an internal standard for ^{1}H NMR analysis which showed a >20:1 product ratio for the title compound. The crude material was purified by a flash chromatography column (silica gel: 3 to 5% Et_2O in hexanes) and heating the isolated material on high vacuum at 50 °C to provide the title compound as a colorless oil (95.0 mg, 0.26 mmol, 53% yield). ^{1}H NMR (400 MHz, $CDCl_3$) δ: 7.64 – 7.54 (m, 2H), 7.23 (dd, *J* = 7.7, 1.0 Hz, 1H), 7.06 (dd, *J* = 7.8, 1.0 Hz, 1H), 6.31 (q, *J* = 6.6 Hz, 1H), 2.69 (tt, *J* = 11.6, 3.5 Hz, 1H), 1.93 (d, *J* = 11.0 Hz, 2H), 1.84 (d, *J* = 12.5 Hz, 2H), 1.79 – 1.69 (m, 4H), 1.55 – 1.35 (m, 4H), 1.31 – 1.19 (m, 1H); ^{13}C NMR (100 MHz, $CDCl_3$) δ: 166.33, 158.68, 154.47 (d, *J* = 12.6 Hz), 145.73 (d, *J* = 4.0 Hz), 145.64 (d, *J* = 263.0 Hz), 137.18, 127.93 (d, *J* = 20.0 Hz), 120.02, 117.33, 114.41, 102.06 (d, *J* = 3.2 Hz), 46.51, 33.03, 33.01, 26.65, 26.63, 26.24; ^{19}F NMR (376 MHz, $CDCl_3$) δ: -138.64 (d, *J* = 8.4 Hz); IR υ_{max}/cm^{-1} (film): 2925, 2851, 2360, 2341, 2235, 1608, 1574, 1562, 1456, 1325, 1268, 1221, 1164, 1046, 899, 808, 745; m/z LRMS (ESI + APCI): $[M+H]^+$ calculated for $C_{19}H_{20}ClFN_3O^+$ = 360.1, found 360.2.

4-((2-Cyclohexyl-6-methylpyridin-3-yl)oxy)-6-methylpyrimidine (3aq)

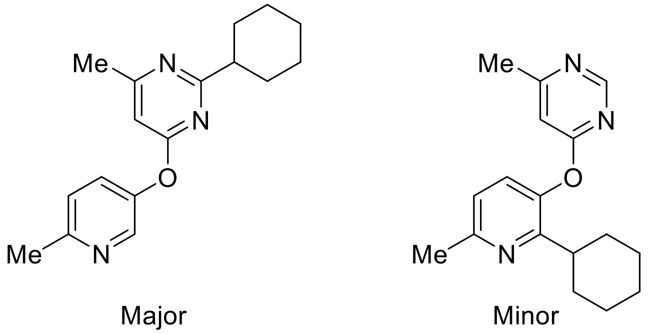

2:1 (major:minor)

Prepared according to general procedure E using **1aq** (306 mg, 0.50 mmol), *t*BuOOH (5.50 M in decane; 227 µL, 1.25 mmol), bromocyclohexane (124 µL, 1.00 mmol), $(TMS)_3SiH$ (309 µL, 1.00 mmol), and MeCN:TFE (1:1; 10.0 mL, 0.05 M). The reaction mixture was placed in the white LED photoreactor setup with stirring for 21 hours. The crude material was purified by a flash chromatography column (silica gel: 2% NEt_3 in hexanes) to provide a 1:2 inseparable mixture of isomers **3aq** (**minor**) and **3aq** (**major**) as a colorless oil (65.6 mg, 0.23 mmol, 46% combined yield). ^{1}H NMR (Mixture of isomers; 400 MHz, $CDCl_3$) δ: 8.57 (minor; s, 1H), 8.34 (major; d, *J* = 2.9 Hz, 2H), 7.36 (dd, *J* = 8.5, 2.7 Hz, 2H), 7.19 (d, *J* = 13.1 Hz, 1H), 7.14 (d, *J* = 8.1 Hz, 3H), 6.95 (d, *J* = 8.2 Hz, 1H), 6.70 (s, 1H), 6.43 (s, 2H), 2.82 – 2.57 (m, 3H), 2.58 – 2.36 (m, 20H), 1.86 – 1.77 (m, 5H), 1.75 – 1.55 (m, 15H), 1.49 – 1.34 (m, 5H), 1.34 – 1.10 (m, 11H); ^{13}C NMR (Mixture of isomers; 100 MHz, $CDCl_3$) δ: 174.64, 169.72, 169.31, 169.18, 169.08, 158.30, 157.70, 155.43, 154.99, 147.26, 143.90, 142.60, 130.05, 129.62, 123.60, 121.33, 106.83, 103.61, 47.20, 40.11, 31.65, 31.36, 26.57, 26.13, 25.91, 24.23, 24.15, 23.86; m/z LRMS (ESI + APCI): $[M+H]^+$ calculated for $C_{17}H_{22}N_3O^+$ =284.2, found 284.2.

5-(4-(2-(6-Cyclohexyl-5-ethylpyridin-2-yl)ethoxy)benzyl)-3-(pyridin-2-ylmethyl)thiazolidine-2,4-dione (3ar)

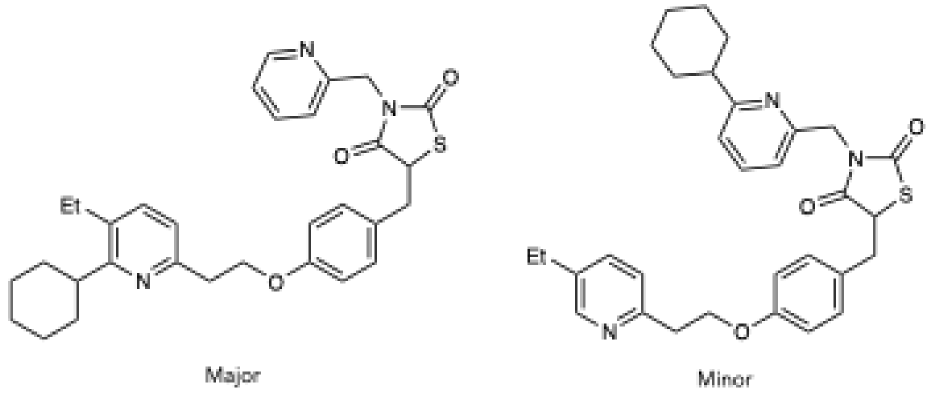

10.8:1.0 (major:minor)

Prepared according to general procedure F using **1ar** (429 mg, 0.50 mmol), TfOH (44.0 μL, 0.50 mmol), *t*BuOOH (5.50 M in decane; 227 μL, 1.25 mmol), bromocyclohexane (124 μL, 1.00 mmol), $(TMS)_3SiH$ (309 μL, 1.00 mmol), and MeCN:TFE (1:20; 25.0 mL, 0.02 M). The reaction mixture was placed in the white LED photoreactor setup with stirring for 18 hours. After the stated workup procedure, the crude material was concentrated *in vacuo*. To this residue was added CH_2Cl_2 (10.0 mL) and DBU (75.0 μL, 0.50 mmol). The reaction was allowed to stir for 1 hour at room temperature. The reaction was then quenched with a saturated solution of NH_4Cl, the organic layer washed with water (1x), dried (Na_2SO_4), filtered, and concentrated *in vacuo*. 1,3,5-Trimethoxybenzene (82.5 mg, 0.50 mmol) was added as an internal standard to the crude material for ^{1}H NMR analysis which displayed a 10.8:1.0 mixture of products **3ar** (**major**) and **3ar** (**minor**). The crude material was purified by a flash chromatography column (silica gel: 25 to 30% EtOAc in hexanes) to provide the title compound as a white solid (92.0 mg, 0.17 mmol, 35% yield). mp: 121–123 °C; ^{1}H NMR (400 MHz, $CDCl_3$) δ: 8.50 (dd, *J* = 4.9, 0.9 Hz, 1H), 7.53 (td, *J* = 7.7, 1.8 Hz, 1H), 7.33 (d, *J* = 7.7 Hz, 1H), 7.12 (td, *J* = 6.1, 1.5 Hz, 3H), 6.96 (d, *J* = 7.8 Hz, 1H), 6.93 (d, *J* = 7.9 Hz, 1H), 6.84 (d, *J* = 8.6 Hz, 2H), 4.86 (d, *J* = 2.1 Hz, 2H), 4.53 (dd, *J* = 9.0, 3.9 Hz, 1H), 4.35 (t, *J* = 6.9 Hz, 2H), 3.45 (dd, *J* = 14.2, 3.8 Hz, 1H), 3.28 – 2.97 (m, 3H), 2.84 (tt, *J* = 10.9, 4.1 Hz, 1H), 2.64 (q, *J* = 7.6 Hz, 2H), 1.93 – 1.81 (m, 2H), 1.81 – 1.60 (m, 5H), 1.50 – 1.30 (m, 3H), 1.20 (t, *J* = 7.5 Hz, 3H); ^{13}C NMR (100 MHz, $CDCl_3$) δ: 173.81, 171.13, 163.36, 158.56, 154.72, 154.02, 149.68, 136.73, 136.31, 133.41, 130.58, 127.52, 122.65, 121.37, 120.53, 114.93, 67.65, 51.97, 46.23, 41.54, 37.82, 37.75, 32.49, 26.87, 26.21, 24.90, 15.52; IR υ_{max}/cm^{-1} (film): 2962, 2924, 2850, 2359, 2341, 1751, 1681, 1591, 1571, 1511, 1379, 1244, 1154, 1035, 981, 827, 751, 667; m/z LRMS (ESI + APCI): $[M+H]^+$ calculated for $C_{31}H_{36}N_3O_3S^+$ = 530.2, found 530.3.

Ethyl 4-(2-cyclohexyl-8-(pyridin-2-yl)-5,6-dihydro-11*H*-benzo [5,6] cyclohepta [1,2-*b*] pyridin-11-ylidene)piperidine-1-carboxylate (3as)

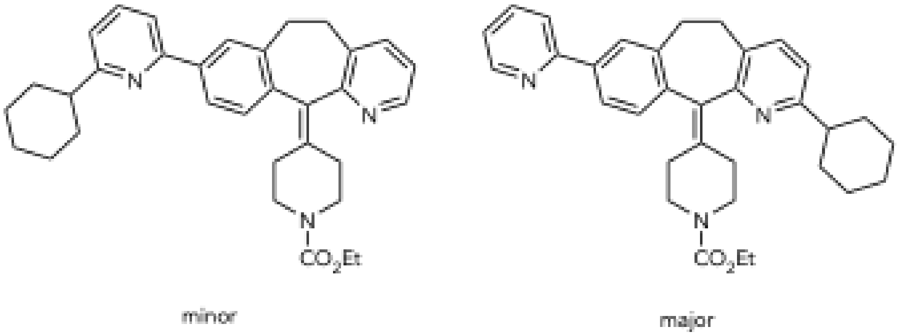

12.0:1.0 (major:minor)

Prepared according to general procedure F using **1as** (418 mg, 0.50 mmol), TfOH (44.0 μL, 0.50 mmol), *t*BuOOH (5.50 M in decane; 227 μL, 1.25 mmol), bromocyclohexane (124 μL, 1.00 mmol), $(TMS)_3SiH$

(309 μL, 1.00 mmol), and MeCN:TFE (1:20; 25.0 mL, 0.02 M). The reaction mixture was placed in the white LED photoreactor setup with stirring for 17 hours. After the modified workup procedure of concentrating the crude reaction mixture in CH_2Cl_2 with 20.0 mL 1.00 M aqueous NaOH, 1,3,5-trimethoxybenzene (82.5 mg, 0.50 mmol) was added as an internal standard to the crude material for ^{1}H NMR analysis which displayed a 12.0:1.0 mixture of products **3as** (**major**) and **3as** (**minor**). The crude material was purified by a flash chromatography column (silica gel: 15 to 35% EtOAc in hexanes) to provide the title compound as a white solid (128 mg, 0.25 mmol, 50% yield). mp: 137–141 °C; ^{1}H NMR (400 MHz, $CDCl_3$) δ: 8.64 (d, *J* = 4.8 Hz, 1H), 7.86 (d, *J* = 1.9 Hz, 1H), 7.78 – 7.52 (m, 3H), 7.33 (dd, *J* = 8.0, 5.7 Hz, 2H), 7.16 (ddd, *J* = 5.9, 4.8, 2.6 Hz, 1H), 6.93 (d, *J* = 7.9 Hz, 1H), 4.13 (q, *J* = 7.1 Hz, 2H), 3.85 (s, 2H), 3.46 (ddd, *J* = 16.2, 10.8, 4.3 Hz, 1H), 3.30 (ddd, *J* = 15.9, 8.2, 4.4 Hz, 1H), 3.11 (dp, *J* = 13.4, 4.0 Hz, 2H), 2.97 – 2.78 (m, 2H), 2.69 (ddd, *J* = 11.6, 8.2, 3.3 Hz, 1H), 2.52 (ddd, *J* = 14.3, 9.3, 4.4 Hz, 1H), 2.37 (dq, *J* = 8.4, 4.2 Hz, 3H), 1.99 – 1.62 (m, 5H), 1.54 – 1.29 (m, 5H), 1.24 (t, *J* = 7.1 Hz, 3H); ^{13}C NMR (100 MHz, $CDCl_3$) δ: 163.47, 157.21, 155.57, 149.65, 140.65, 138.71, 138.38, 138.04, 136.96, 136.73, 135.46, 130.57, 129.68, 127.42, 124.42, 122.04, 120.41, 118.69, 61.30, 46.08, 45.03, 33.92, 32.39, 32.10, 31.73, 31.09, 30.72, 26.68, 26.49, 26.17, 14.77; IR υ_{max}/cm^{-1} (film): 3007, 2978, 2924, 2852, 2360, 2341, 1684, 1586, 1464, 1432, 1277, 1226, 1115, 996, 748, 667; m/z LRMS (ESI + APCI): $[M+H]^+$ calculated for $C_{33}H_{38}N_3O_2^+$ = 508.3, found 508.4.

Ethyl 4-(8-(6-cyclohexylpyridin-2-yl)-5,6-dihydro-11*H*-benzo [5,6] cyclohepta[1,2-*b*]pyridin-11-ylidene)piperidine-1-carboxylate (3at)

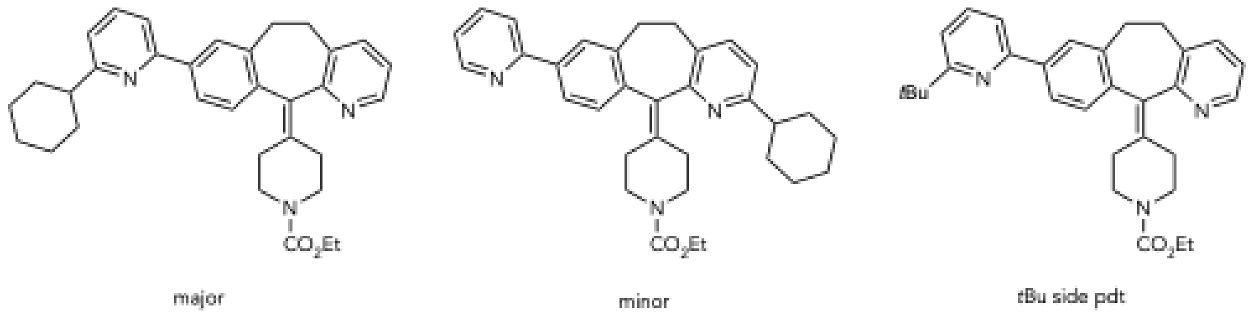

4.9:1.0 (major:minor)

Small amounts of *t*Bu side pdt

Prepared according to general procedure F using **1at** (418 mg, 0.50 mmol), TfOH (44.0 μL, 0.50 mmol), *t*BuOOH (5.50 M in decane; 227 μL, 1.25 mmol), bromocyclohexane (124 μL, 1.00 mmol), $(TMS)_3SiH$ (309 μL, 1.00 mmol), and MeCN:TFE (1:20; 25.0 mL, 0.02 M). The reaction mixture was placed in the white LED photoreactor setup with stirring for 16 hours. After the modified workup procedure of concentrating the crude reaction mixture in CH_2Cl_2 with 20.0 mL 1.00 M aqueous NaOH, mesitylene (70.0

μL, 0.50 mmol) was added as an internal standard for ^{1}H NMR analysis which displayed a 4.9:1.0 mixture of products **3at** (**major**) and **3at** (**minor**). The crude material was purified by a flash chromatography column (silica gel: 30 to 40% EtOAc in hexanes with 1% Et_3N) to provide a 12.5:1.0 inseparable mixture of isomers **3at** (**major**) and **3at'** (***t*Bu side product**) as an off–white solid (140 mg, 0.28 mmol, 55% combined yield). mp: 65–72 °C; ^{1}H NMR (Major; 400 MHz, $CDCl_3$) δ: 8.33 (dd, *J* = 4.8, 1.7 Hz, 1H), 7.78 (d, *J* = 1.8 Hz, 1H), 7.69 (dd, *J* = 7.9, 1.9 Hz, 1H), 7.55 (t, *J* = 7.8 Hz, 1H), 7.42 – 7.30 (m, 2H), 7.21 (d, *J* = 8.0 Hz, 1H), 7.04 – 6.92 (m, 2H), 4.07 (q, *J* = 7.1 Hz, 2H), 3.76 (s, 2H), 3.43 (ddd, *J* = 15.9, 10.2, 4.6 Hz, 1H), 3.32 (ddd, *J* = 15.8, 8.1, 4.6 Hz, 1H), 3.13 – 2.99 (m, 2H), 2.93 – 2.79 (m, 2H), 2.69 (tt, *J* = 11.8, 3.4 Hz, 1H), 2.44 (ddd, *J* = 14.1, 9.5, 4.6 Hz, 1H), 2.38 – 2.16 (m, 3H), 1.94 (ddd, *J* = 13.5, 3.7, 1.7 Hz, 2H), 1.79 (dt, *J* = 12.5, 3.3 Hz, 2H), 1.69 (dtd, *J* = 11.8, 3.2, 1.6 Hz, 1H), 1.50 (qd, *J* = 12.4, 3.1 Hz, 2H), 1.44 – 1.29 (m, 2H), 1.30 – 1.13 (m, 4H); ^{13}C NMR (Major; 100 MHz, $CDCl_3$) δ: 166.52, 157.41, 156.31, 155.65, 146.64, 139.88, 139.38, 138.16, 137.68, 137.13, 136.98, 135.24, 133.85, 129.70, 127.79, 124.86, 122.24, 119.29, 117.77, 61.41, 46.78, 45.02, 33.09, 32.11, 31.97, 30.96, 30.71, 30.36, 26.72, 26.28, 14.83; IR υ_{max}/cm^{-1} (film): 2924, 2851, 2360, 2341, 1688, 1572, 1436, 1385, 1324, 1227, 1113, 995, 805, 747, 667; m/z LRMS (ESI + APCI): $[M+H]^+$ calculated for $C_{33}H_{38}N_3O_2^+$ (title compound **3at**) = 508.3, found 508.4; $[M+H]^+$ calculated for $C_{31}H_{36}N_3O_2^+$ (*t*Bu side product **3at'**) = 482.3, found 482.3.

4-((2-Bromophenyl)thio)-2-cyclohexyl-6-phenylpyridine (7)

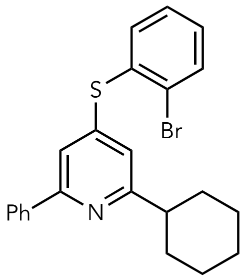

Prepared according to general procedure E using triphenyl(2-phenylpyridin-4-yl)phosphonium trifluoromethanesulfonate **(2a)** (283 mg, 0.50 mmol), *t*BuOOH (90% in H_2O; 138 μL, 1.25 mmol), 4-bromocyclohexane (124 μL, 1.00 mmol), $(TMS)_3SiH$ (309 μL, 1.00 mmol), and MeCN:TFE (1:1; 10.0 mL, 0.05 M). The reaction mixture was placed in the white LED photoreactor setup with stirring for 18 hours. The reaction mixture was then washed with hexanes (6x) and concentrated *in vacuo*. This crude mixture was dried on high vacuum for 2 hours before being dissolved in THF (anhydrous; 1.00 mL). To a separate 8 mL vial purged with N_2, sodium hydride (30.0 mg, 0.75 mmol) dissolved in THF (anhydrous; 1.00 mL) at 0 °C was added 2-bromobenzenethiol (90.0 μL, 0.75 mmol) and the reaction stirred at 0 °C for 30 minutes. Then 15-crown-5 (150 μL, 0.75 mmol) was added and the reaction stirred at 0 °C for 30 minutes.

To this was added the THF solution containing the phosphonium mixture dropwise and the reaction stirred at 0 °C for 30 minutes before being heated to 60 °C for 2 hours. After cooling to room temperature, the reaction was diluted in CH_2Cl_2 and washed once with H_2O, then the organic layer was dried (Na_2SO_4) and concentrated *in vacuo*. The crude material was purified by a flash chromatography column (silica gel: 0 to 3% Et_2O in hexanes) followed by preparative thin layer chromatography (silica gel: 2% Et_2O in hexanes) to provide the title compound as a colorless oil (104 mg, 0.25 mmol, 49% yield). ^{1}H NMR (400 MHz, $CDCl_3$) δ: 7.92 (d, *J* = 6.8 Hz, 2H), 7.74 (dd, *J* = 8.0, 1.5 Hz, 1H), 7.55 (dd, *J* = 7.7, 1.7 Hz, 1H), 7.48 – 7.32 (m, 4H), 7.32 – 7.18 (m, 2H), 6.86 (d, *J* = 1.6 Hz, 1H), 2.71 (tt, *J* = 11.8, 3.4 Hz, 1H), 2.03 – 1.94 (m, 2H), 1.85 (dt, *J* = 12.6, 3.3 Hz, 2H), 1.80 – 1.70 (m, 1H), 1.54 (qd, *J* = 12.3, 3.1 Hz, 2H), 1.41 (qt, *J* = 12.5, 3.1 Hz, 2H), 1.28 (qt, *J* = 12.5, 3.3 Hz, 1H); ^{13}C NMR (100 MHz, $CDCl_3$) δ: 166.84, 157.02, 147.58, 139.56, 135.64, 134.01, 132.89, 130.47, 129.01, 128.78, 128.75, 128.53, 127.17, 118.12, 116.59, 46.65, 32.90, 26.64, 26.22; IR υ_{max}/cm^{-1} (film): 3059, 2922, 2849, 2360, 2342, 1565, 1543, 1446, 1397, 1105, 1020, 772, 750, 691; m/z LRMS (ESI + APCI): $[M+H]^+$ calculated for $C_{23}H_{23}BrNS^+$ = 424.1, found 424.1.

2-cyclohexyl-6-phenyl-4-(phenylethynyl)pyridine (8)

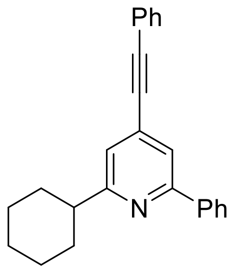

Prepared according to previously reported method[16] using **2a** (324 mg, 0.50 mmol), phenylacetylene (102.1 mg, 1 mmol), $Pd(PPh_3)_2Cl_2$ (35.1 mg, 10 mol%), CuI (9.5 mg, 10 mol%), DIPEA (96.9 mg, 0.75 mmol) and anhydrous NMP (2 mL, 0.25 M). The reaction was heated to 100 ºC for 12 hours. After the reported workup procedure, the crude material was purified via flash chromatography column (silica gel: 25% CH_2Cl_2 in hexanes) to provide the title compound as a colorless oil (59.0 mg, 0.175 mmol, 35% yield). ^{1}H NMR (400 MHz, $CDCl_3$) δ: 8.01 – 7.93 (m, 2H), 7.58 (d, *J* = 1.3 Hz, 1H), 7.54 – 7.44 (m, 2H), 7.39 (dd, *J* = 8.2, 6.4 Hz, 2H), 7.36 – 7.27 (m, 4H), 7.17 – 7.07 (m, 1H), 2.72 (tt, *J* = 11.9, 3.5 Hz, 1H), 2.02 – 1.91 (m, 2H), 1.81 (dt, *J* = 12.9, 3.3 Hz, 2H), 1.75 – 1.65 (m, 1H), 1.54 (qd, *J* = 12.5, 3.2 Hz, 2H), 1.38 (qt, *J* = 12.6, 3.2 Hz, 2H), 1.31 – 1.20 (m, 1H); ^{13}C NMR (100 MHz, $CDCl_3$) δ: 166.69, 156.72, 139.47, 132.14, 131.99, 129.11, 129.05, 128.80, 128.61, 127.12, 122.57, 121.27, 119.80, 92.73, 87.84, 46.67, 32.99, 26.70, 26.29; m/z LRMS (ESI + APCI): $[M+H]^+$ calculated for $C_{25}H_{24}N^+$ = 338.2, found 338.3.

2-cyclohexyl-6-phenylpyridin-4-amine (9)

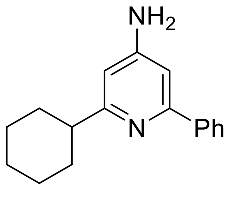

Prepared according to previously reported method[17] using **2a** (162 mg, 0.25 mmol), sodium azide (20.3 mg, 0.31 mmol) and DMSO (0.18 mL). The reaction was stirred at 120 °C for 20 hours before it was cooled to room temperature and H_2O (20 uL) was added. The reaction was heated to 100 °C for 40 hours before cooling to room temperature. The crude reaction mixture was directly adsorbed to SiO_2 and purified via flash column chromatography (silica gel: 35% ethyl acetate in hexanes with 5% triethylamine) to provide the title compound as a colorless amorphous solid (49.6 mg, 0.20 mmol, 79% yield). ^{1}H NMR (600 MHz, $CDCl_3$) δ 7.94 – 7.88 (m, 1H), 7.41 (td, *J* = 7.2, 1.3 Hz, 2H), 7.37 – 7.33 (m, 1H), 6.78 (d, *J* = 2.0 Hz, 1H), 6.37 (d, *J* = 2.0 Hz, 1H), 4.30 (s, 2H), 2.71 (tt, *J* = 11.9, 3.4 Hz, 1H), 2.05 – 1.94 (m, 2H), 1.84 (dt, *J* = 13.1, 3.3 Hz, 2H), 1.79 – 1.71 (m, 1H), 1.51 (qd, *J* = 12.5, 3.2 Hz, 2H), 1.46 – 1.39 (m, 2H), 1.33 – 1.23 (m, 2H). ^{13}C NMR (151 MHz, $CDCl_3$) δ 166.98, 157.20, 154.18, 139.85, 128.70, 128.59, 127.20, 105.27, 104.72, 46.27, 33.06, 26.71, 26.32; m/z LRMS (ESI + APCI): $[M+H]^+$ calculated for $C_{17}H_{21}N_2^+$ = 253.2, found 253.2.

4-((1-benzhydrylazetidin-3-yl)oxy)-2-cyclohexyl-6-phenylpyridine (10)

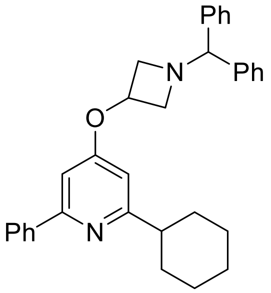

Prepared according to previously reported method[13] using **2a** (324 mg, 0.50 mmol), NaH (60% dispersion in mineral oil; 30.0 mg, 0.75 mmol), 1-benzhydrylazetidin-3-ol (180 mg, 0.75 mmol), and THF (0.50 mL, 5.0 M). The reaction mixture was stirred at room temperature for 12 hours. After the reported workup procedure, the crude material was purified via flash chromatography column (silica gel: 5 to 10% EtOAc in hexanes) to provide the title compound as a colorless solid (105.2 mg, 0.22 mmol, 44% yield). ^{1}H NMR (400 MHz, $CDCl_3$) δ: 7.86 (d, *J* = 7.3 Hz, 2H), 7.35 (d, *J* = 7.8 Hz, 6H), 7.29 (d, *J* = 7.0 Hz, 1H), 7.20 (t, *J*

= 7.5 Hz, 4H), 7.12 (t, J = 7.4 Hz, 2H), 6.82 (d, J = 2.2 Hz, 1H), 6.40 (d, J = 2.1 Hz, 1H), 4.82 (p, J = 5.7 Hz, 1H), 4.36 (s, 1H), 3.70 – 3.62 (m, 2H), 3.13 – 3.05 (m, 2H), 2.62 (tt, J = 11.8, 3.4 Hz, 1H), 1.95 – 1.87 (m, 2H), 1.77 (dt, J = 12.6, 3.3 Hz, 2H), 1.71 – 1.62 (m, 1H), 1.45 (qd, J = 12.2, 2.9 Hz, 2H), 1.39 – 1.26 (m, 2H), 1.20 (dd, J = 9.8, 3.5 Hz, 1H); ^{13}C NMR (100 MHz, $CDCl_3$) δ: 168.42, 164.48, 158.42, 141.90, 139.97, 128.85, 128.68, 128.67, 127.53, 127.43, 127.13, 105.75, 104.67, 78.44, 66.23, 60.41, 46.82, 33.03, 26.67, 26.26; m/z LRMS (ESI + APCI): $[M+H]^+$ calculated for $C_{33}H_{35}N_2O^+$ = 475.3 found 475.4.

2-Isopropyl-2',6'-dimethyl-6-phenyl-4,4'-bipyridine (11)

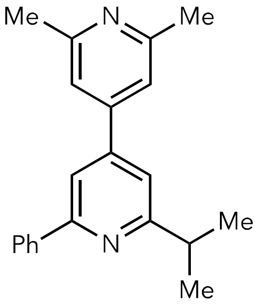

Prepared according to general procedure E using (2,6-dimethylpyridin-4-yl)diphenyl(2-phenylpyridin-4-yl)phosphonium trifluoromethanesulfonate **(2b')** (297 mg, 0.50 mmol), *t*BuOOH (90% in H_2O; 138 μL, 1.25 mmol), 2-bromopropane (94.0 μL, 1.00 mmol), $(TMS)_3SiH$ (309 μL, 1.00 mmol), and MeCN:TFE (1:1; 10.0 mL, 0.05 M). The reaction mixture was placed in the white LED photoreactor setup with stirring for 16 hours. After washing with hexanes (6x), *t*BuOOH (90% in H_2O; 138 μL, 1.25 mmol), 2-bromopropane (94.0 μL, 1.00 mmol), $(TMS)_3SiH$ (309 μL, 1.00 mmol) were added to the reaction mixture and the reaction vessel was placed in the white LED photoreactor setup with stirring for an additional 17 hours. After washing with hexanes (6x), *t*BuOOH (90% in H_2O; 138 μL, 1.25 mmol), 2-bromopropane (94.0 μL, 1.00 mmol), $(TMS)_3SiH$ (309 μL, 1.00 mmol) were added to the reaction mixture and the reaction vessel was placed in the white LED photoreactor setup with stirring for an additional 19 hours. After washing with hexanes (6x), the rection mixture was concentrated *in vacuo*, and then HCl (4.0 M in dioxane; 250 μL, 1.00 mmol) and EtOH (1.25 mL) were added, and the reaction mixture was heated to 80 °C for 22 hours. After cooling to room temperature, the reaction mixture was diluted in CH_2Cl_2 and washed with saturated aqueous $NaHCO_3$ (1x). The aqueous layer was extracted with CH_2Cl_2 (3x) and the combined organic extracts were dried (Na_2SO_4) and concentrated *in vacuo*. The crude material was purified by a flash chromatography column (silica gel: 20 to 40% Et_2O in hexanes) to provide the title compound as a colorless oil (107 mg, 0.35 mmol, 71% yield). ^{1}H NMR (400 MHz, $CDCl_3$) δ: 8.10 (d, J = 7.0 Hz, 2H), 7.72 (d, J = 1.5 Hz, 1H), 7.49 (t, J = 7.4 Hz, 2H), 7.43 (d, J = 7.2 Hz, 1H), 7.33 – 7.16 (m, 3H), 3.22 (hept, J = 6.9 Hz, 1H), 2.63 (s, 6H), 1.42 (d, J = 6.9 Hz, 6H); ^{13}C NMR (100 MHz, $CDCl_3$) δ: 168.19, 158.74, 157.44, 147.50, 147.46, 139.69, 129.08, 128.82, 127.19, 118.42, 117.09, 115.93, 36.76, 24.73, 22.81; IR υ_{max}/cm^{-1} (film):

2959, 2925, 2867, 2359, 2342, 1593, 1580, 1543, 1428, 1386; m/z LRMS (ESI + APCI): $[M+H]^+$ calculated for $C_{21}H_{23}N_2^+$ = 303.2, found 303.2.

2-(*tert*-Butyl)-6-phenylpyridine-4-*d* (12)

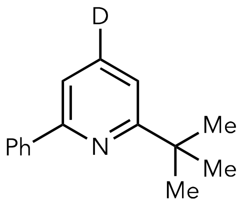

Prepared according to general procedure E using triphenyl(2-phenylpyridin-4-yl)phosphonium trifluoromethanesulfonate **(2a)** (283 mg, 0.50 mmol), *t*BuOOH (5-6 M in decane; 227 µL, 1.25 mmol), 2-bromo-2-methylpropane (112 µL, 1.00 mmol), $(TMS)_3SiH$ (309 µL, 1.00 mmol), and MeCN:TFE (1:1; 10.0 mL, 0.05 M). The reaction mixture was placed in the white LED photoreactor setup with stirring for 17 hours. The reaction mixture was then washed with hexanes (6x), transferred to a 25 mL round bottom flask, and concentrated *in vacuo*. This residue was dried on high vacuum for 15 minutes before adding K_2CO_3 (104 mg, 0.75 mmol) and MeOD:D_2O (9:1; 1.70 mL). This mixture was stirred at room temperature for 5 hours. The reaction mixture was then transferred to a separatory funnel, diluted in CH_2Cl_2, and vigorously shaken with K_2CO_3 (691 mg, 5.00 mmol) dissolved in D_2O (2.00 mL). The organic layer was then dried (Na_2SO_4) and concentrated *in vacuo*. The crude material was purified by a flash chromatography column (silica gel: 0 to 3% Et_2O in hexanes) to provide the title compound as a colorless oil (86.0 mg, 0.41 mmol, 81% yield). ^1H NMR (400 MHz, $CDCl_3$) δ: 8.16 (d, J = 7.6 Hz, 2H), 7.59 (s, 1H), 7.51 (t, J = 7.6 Hz, 2H), 7.43 (t, J = 7.2 Hz, 1H), 7.30 (s, 1H), 1.49 (s, 9H); ^{13}C NMR (100 MHz, $CDCl_3$) δ: 169.38, 155.79, 140.36, 137.15 – 136.52 (t, J = 24.2 Hz), 129.07, 129.02, 127.28, 117.66, 117.17, 38.17, 30.70; IR υ_{max}/cm^{-1} (film): 3063, 2953, 2864, 2360, 2341, 1559, 1410, 1359, 1156, 1068, 898, 755, 695, 661; m/z LRMS (ESI + APCI): $[M+H]^+$ calculated for $C_{15}H_{17}DN^+$ = 213.1, found 213.2.

1.8 Control Experiments ^{1}H Spectra

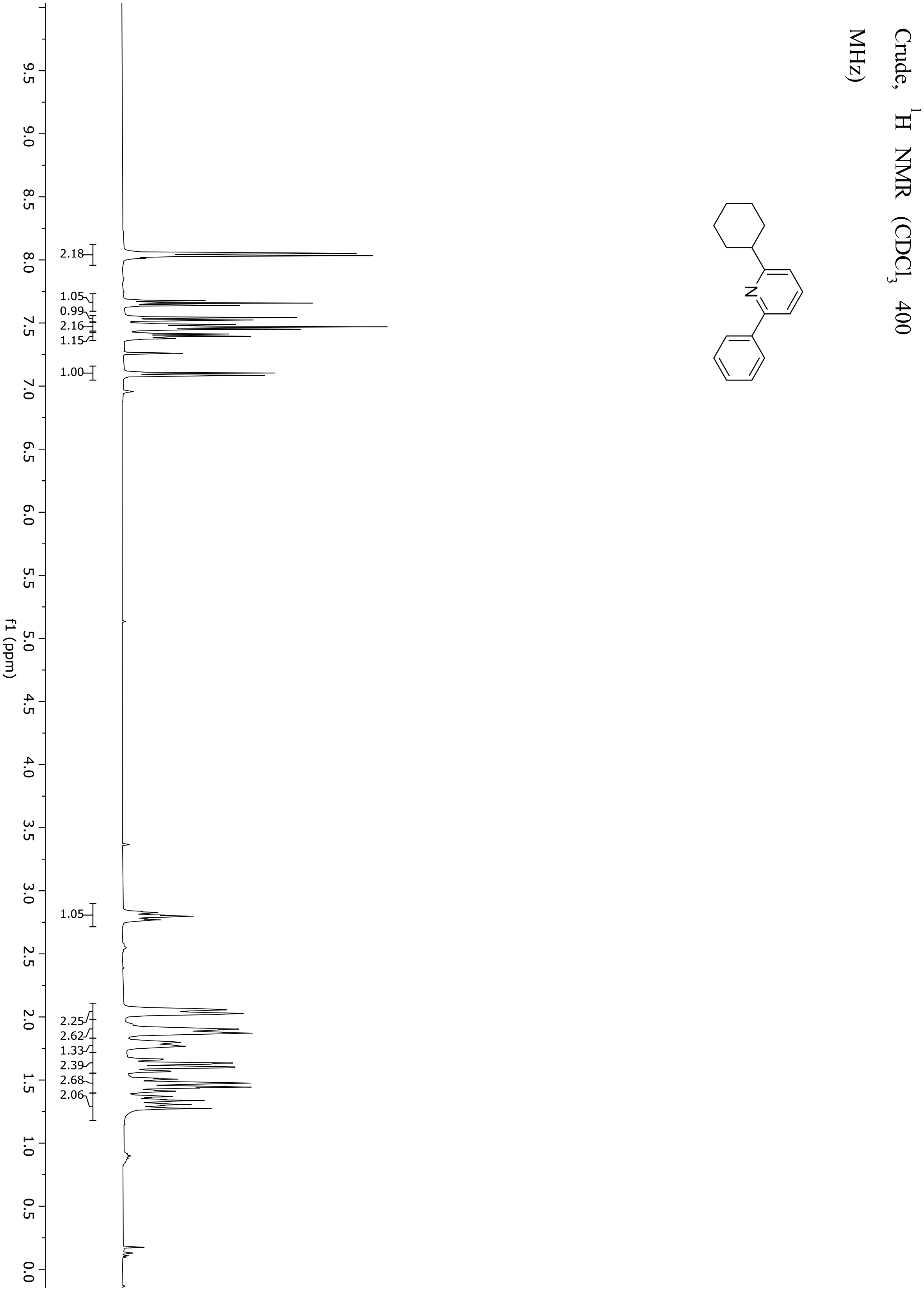

Crude, ^{1}H NMR ($CDCl_3$ 400 MHz)

2 eq TFA

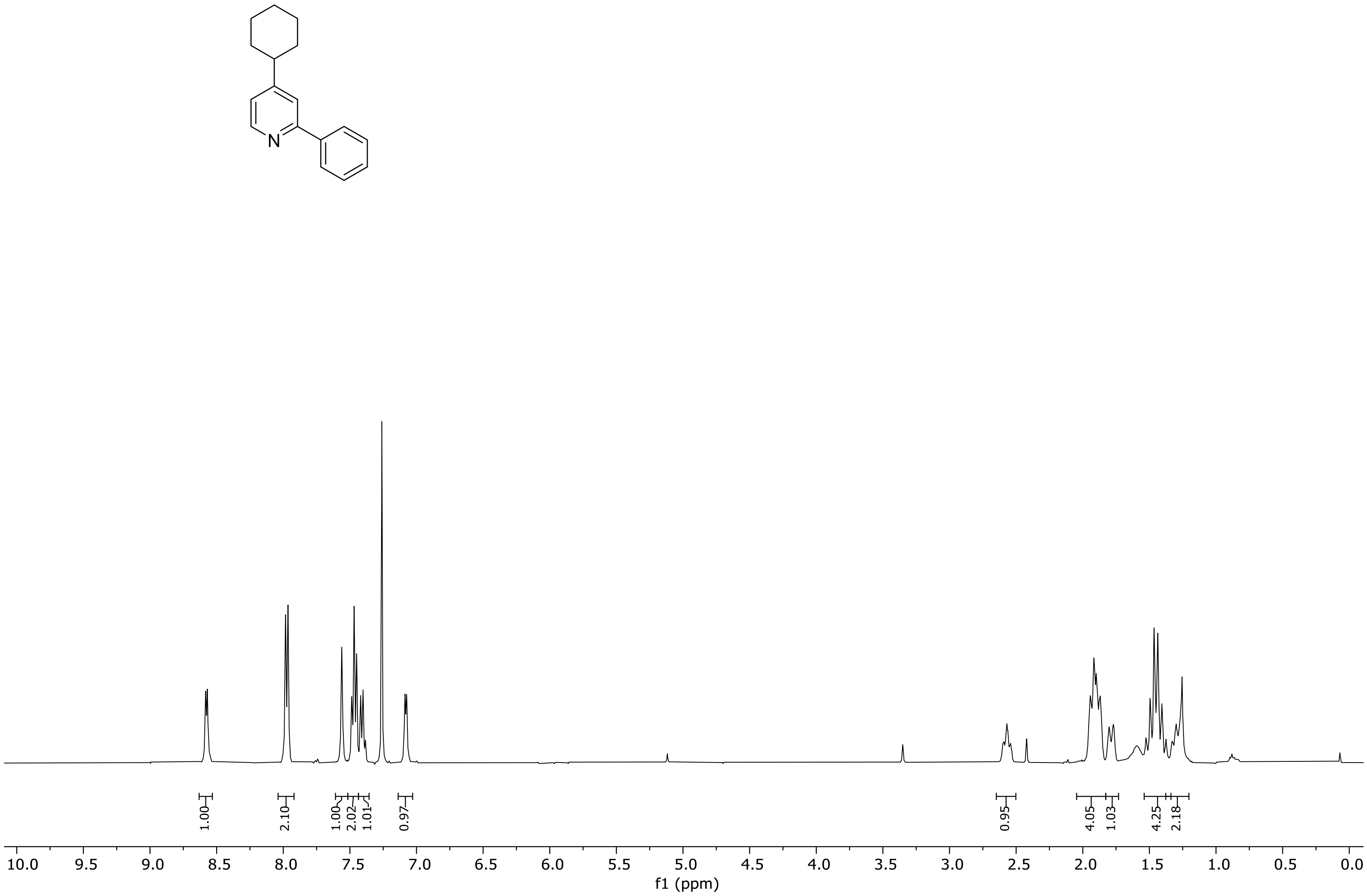

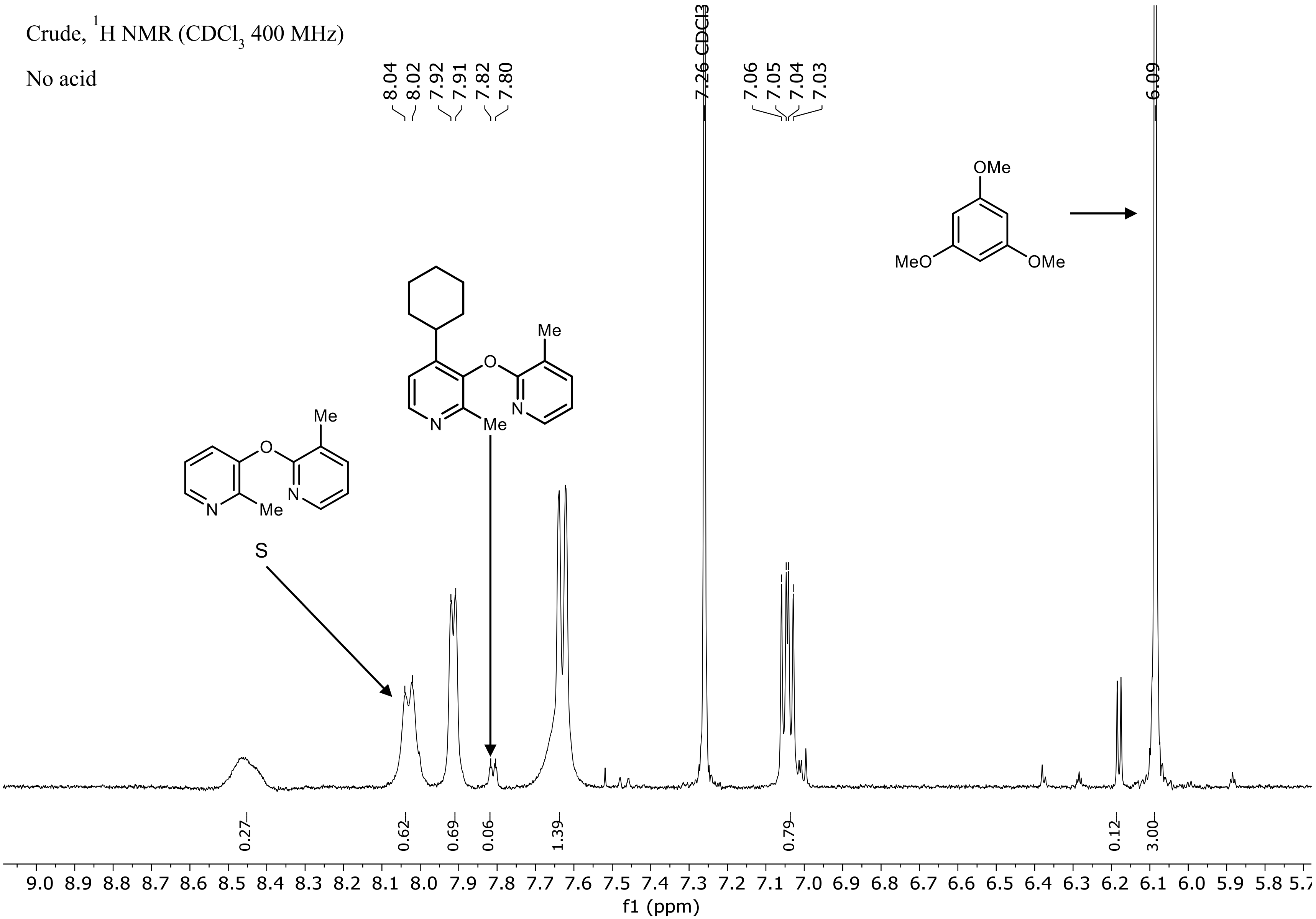
Crude, 1H NMR (CDCl3 400 MHz)
No acid
8.04
8.02
7.92
7.91
7.82
7.80
7.26 CDCl3
7.06
7.05
7.04
7.03
6.09
S
Me
O
N
Me
OMe
MeO
0.27
0.62
0.69
0.06
1.39
0.79
0.12
3.00
9.0 8.9 8.8 8.7 8.6 8.5 8.4 8.3 8.2 8.1 8.0 7.9 7.8 7.7 7.6 7.5 7.4 7.3 7.2 7.1 7.0 6.9 6.8 6.7 6.6 6.5 6.4 6.3 6.2 6.1 6.0 5.9 5.8
f1 (ppm)

Crude, ^{1}H NMR ($CDCl_3$ 400 MHz)

1 eq TFA

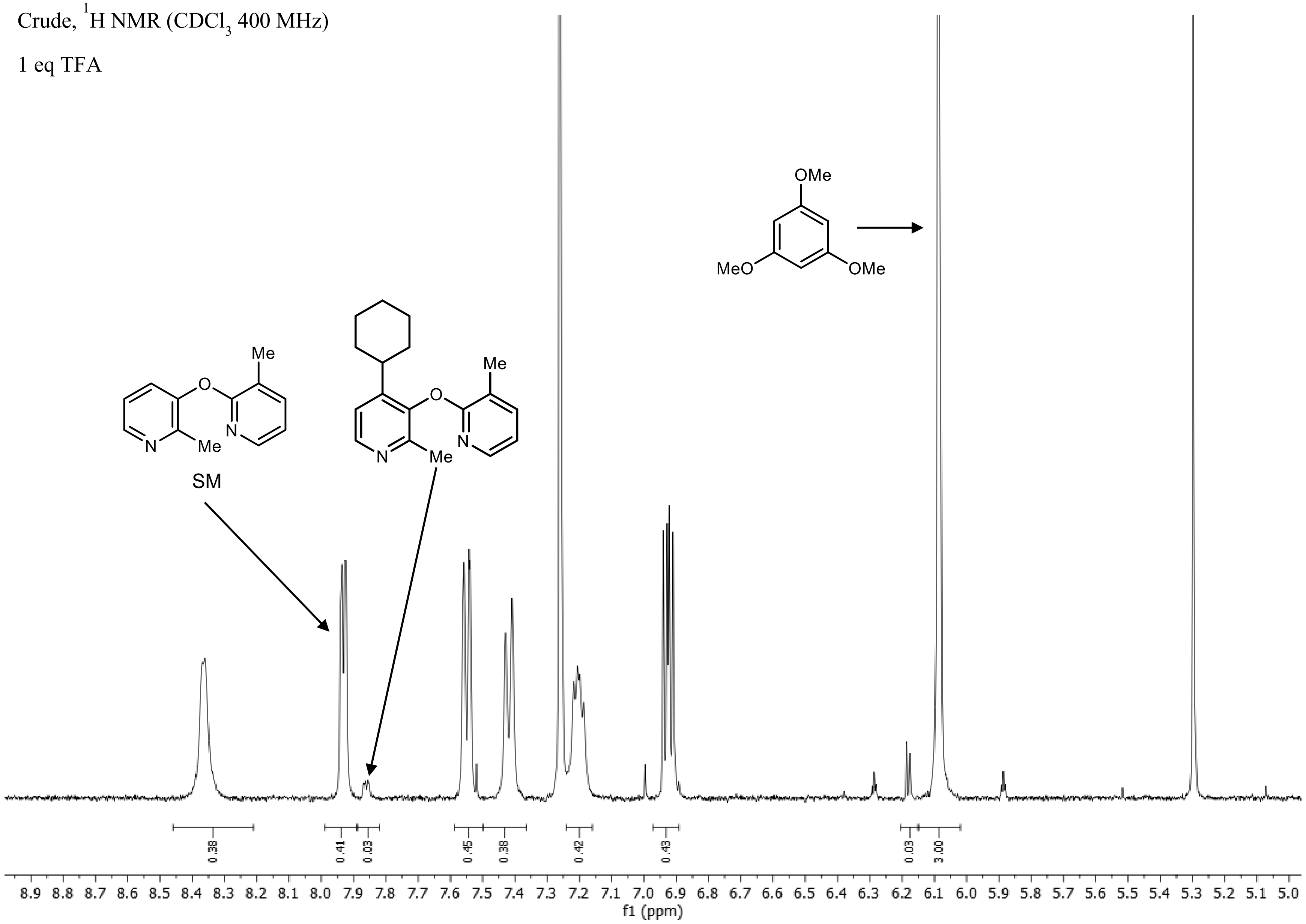

Crude, ^{1}H NMR ($CDCl_3$ 400 MHz)

2 eq TFA

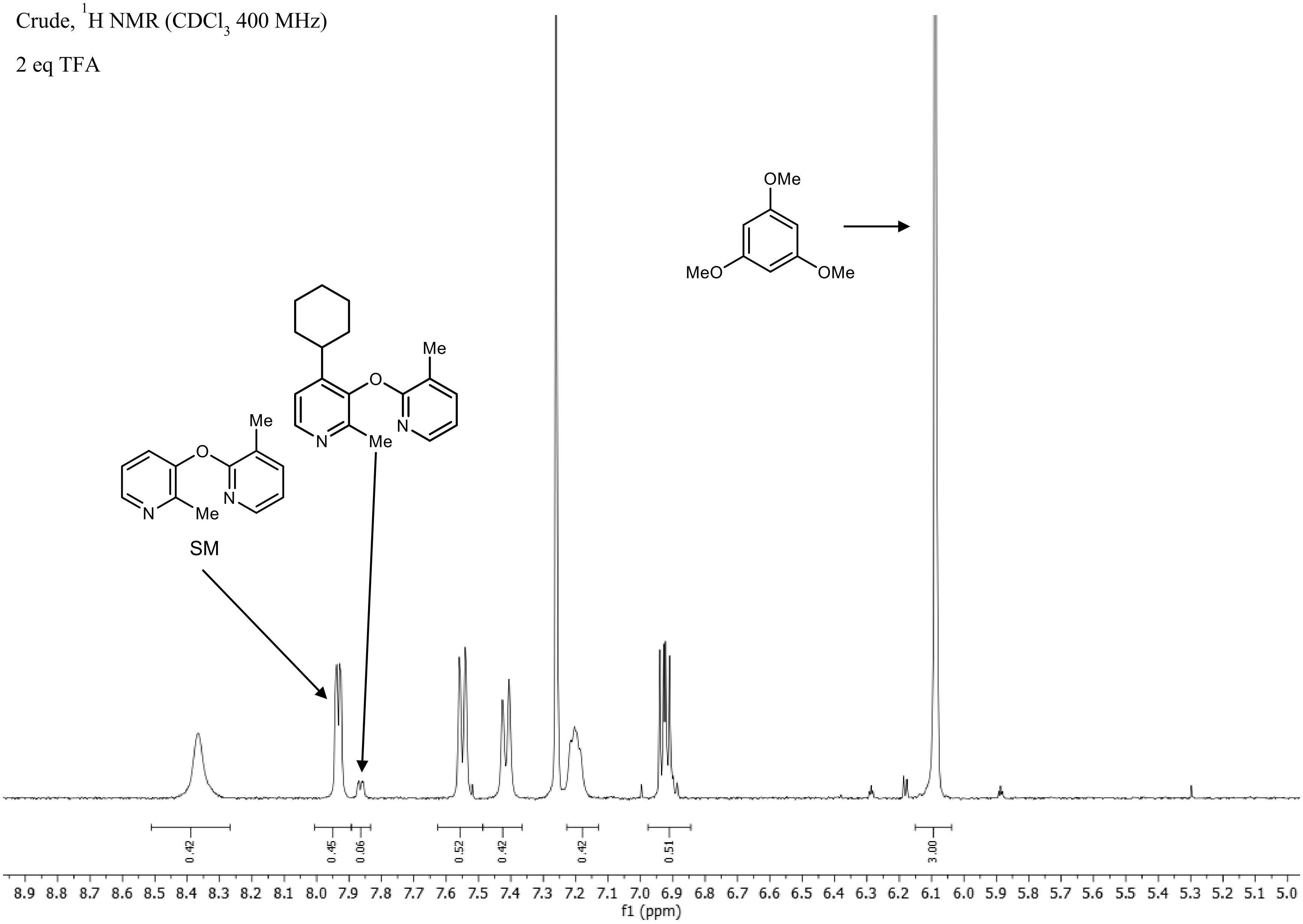

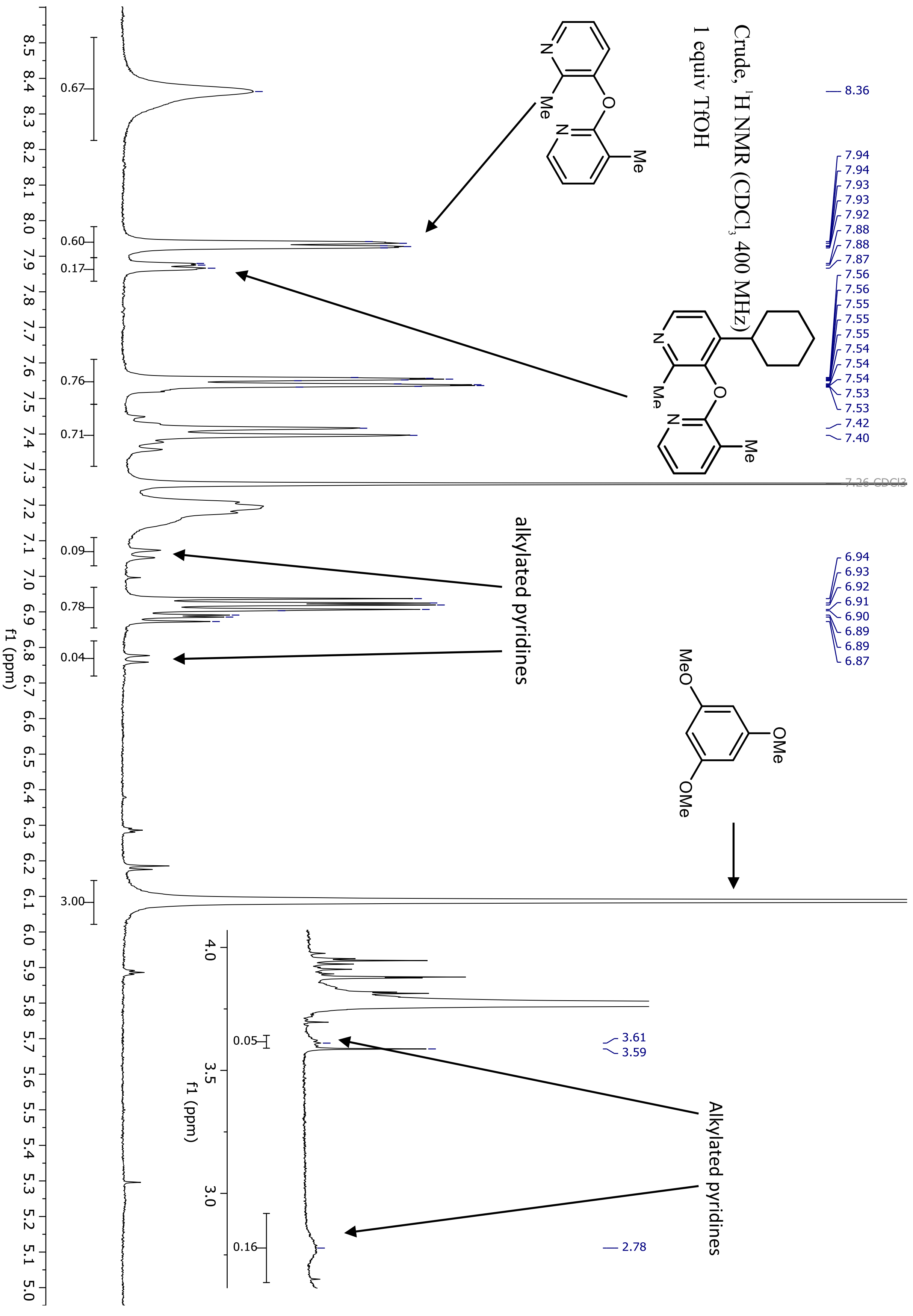
Crude, ^{1}H NMR ($CDCl_3$, 400 MHz)
1 equiv TfOH
alkylated pyridines
Alkylated pyridines
MeO
OMe
OMe
Me
N
O
7.26 CDCl3
f1 (ppm)

Crude, ^{1}H NMR ($CDCl_3$, 400 MHz)
2 equiv TfOH

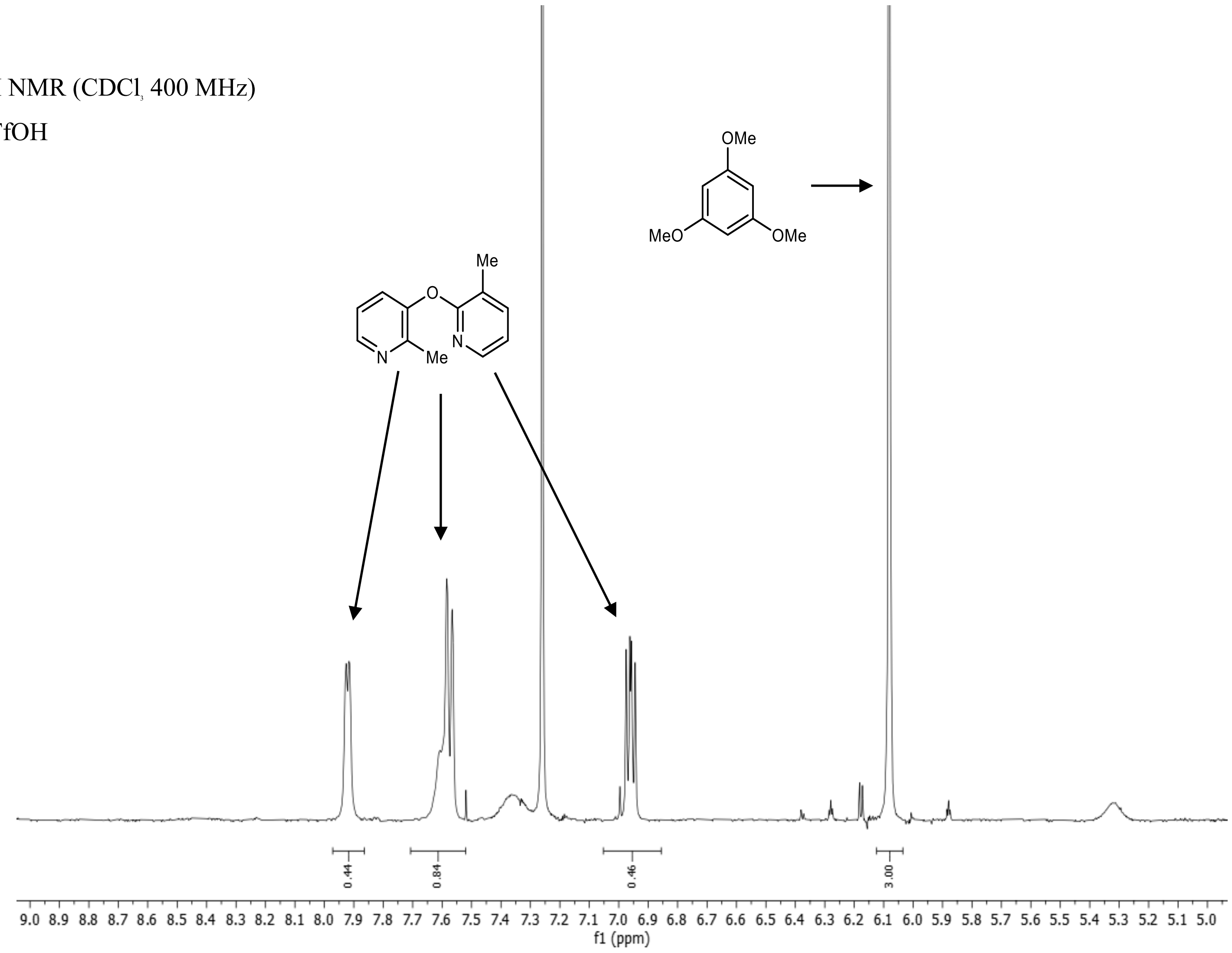

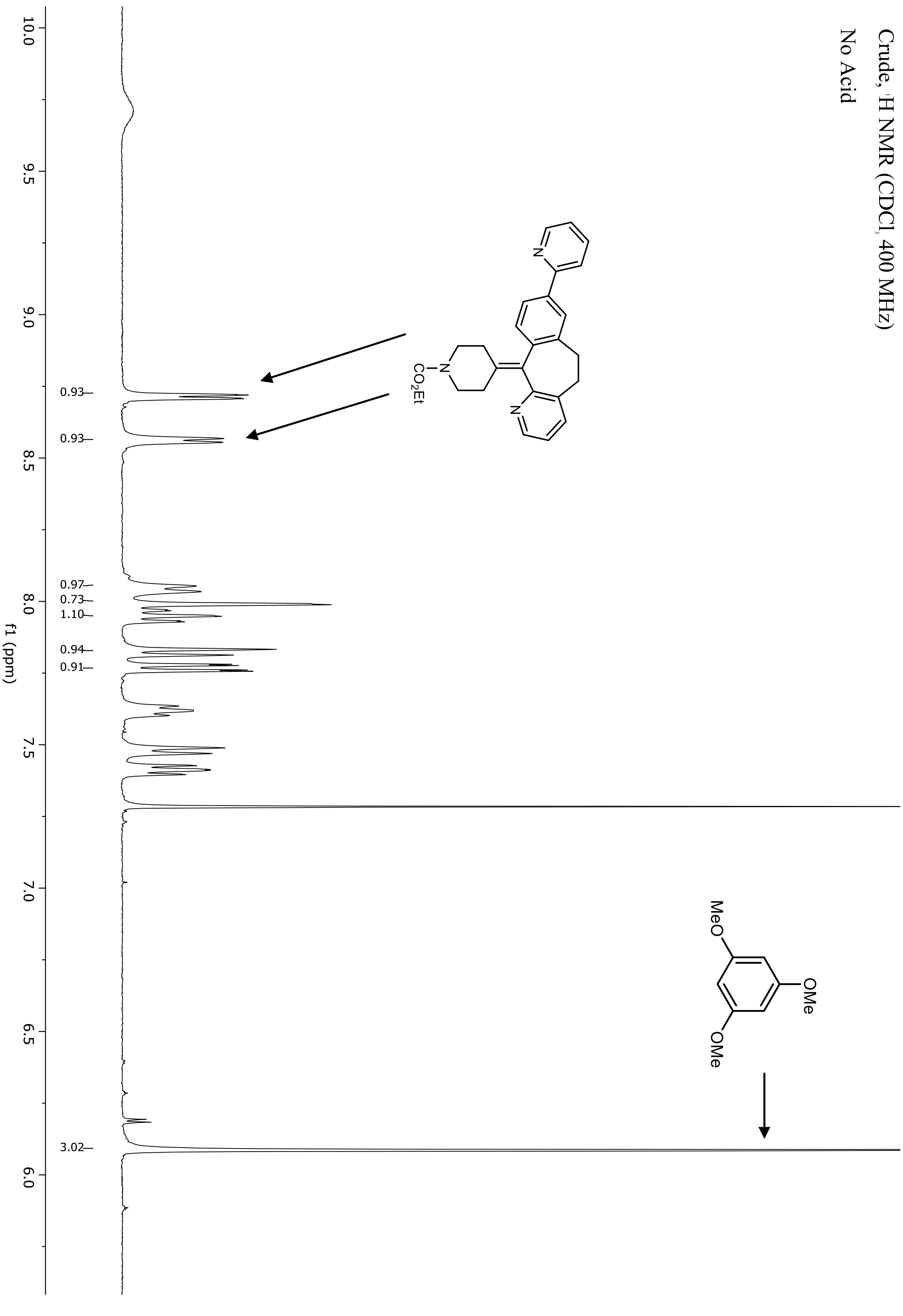
Crude, ^{1}H NMR ($CDCl_3$, 400 MHz)
No Acid
N
N
N
CO_2Et
MeO
OMe
OMe
0.93
0.93
0.97
0.73
1.10
0.94
0.91
3.02
10.0
9.5
9.0
8.5
8.0
7.5
7.0
6.5
6.0
f1 (ppm)

Crude, ^1H NMR ($CDCl_3$ 400 MHz)

1 eq TFA

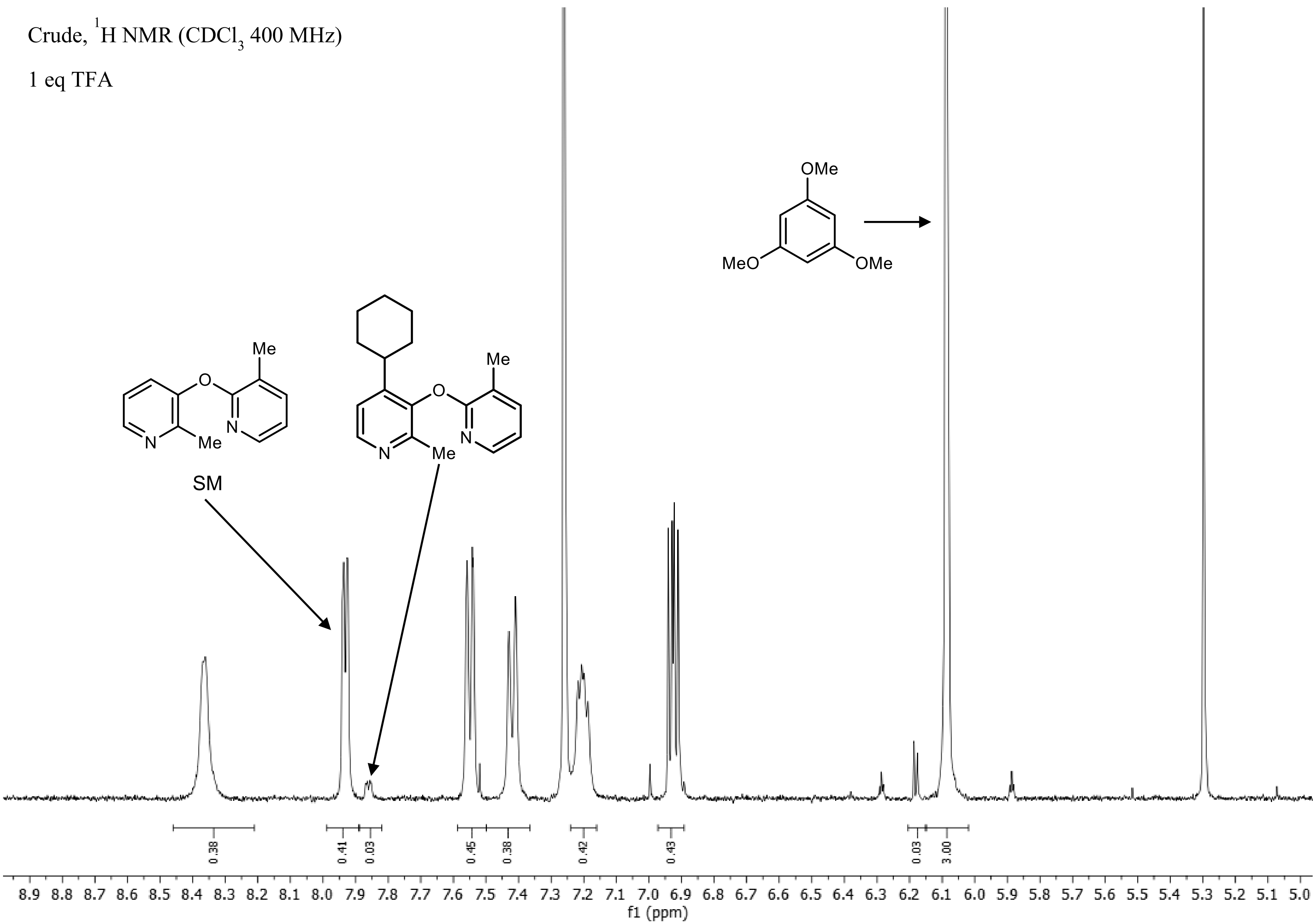

Crude, ^{1}H NMR ($CDCl_3$ 400 MHz)

2 eq TFA

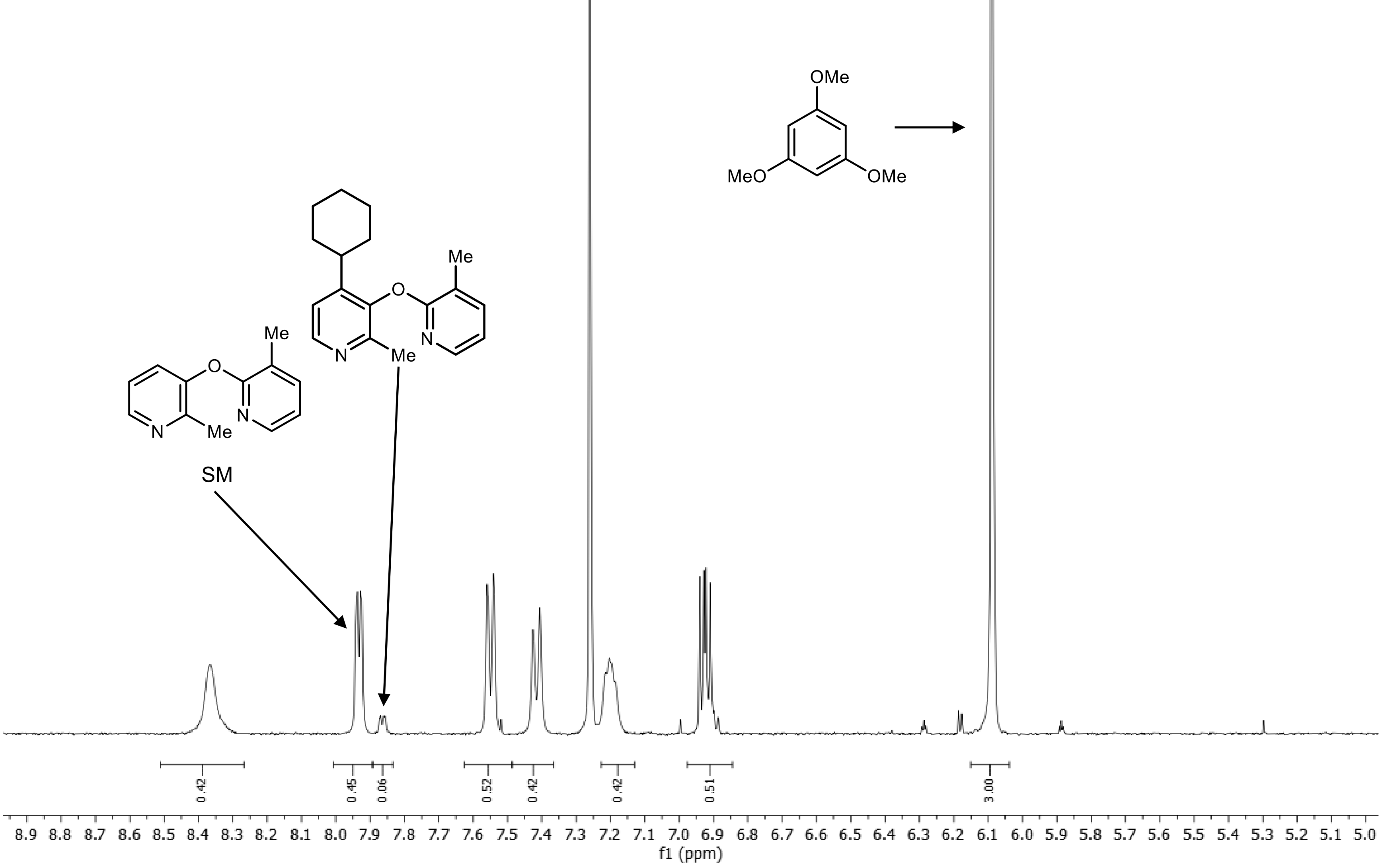

Crude, ^{1}H NMR ($CDCl_3$, 400 MHz)

1 eq TfOH

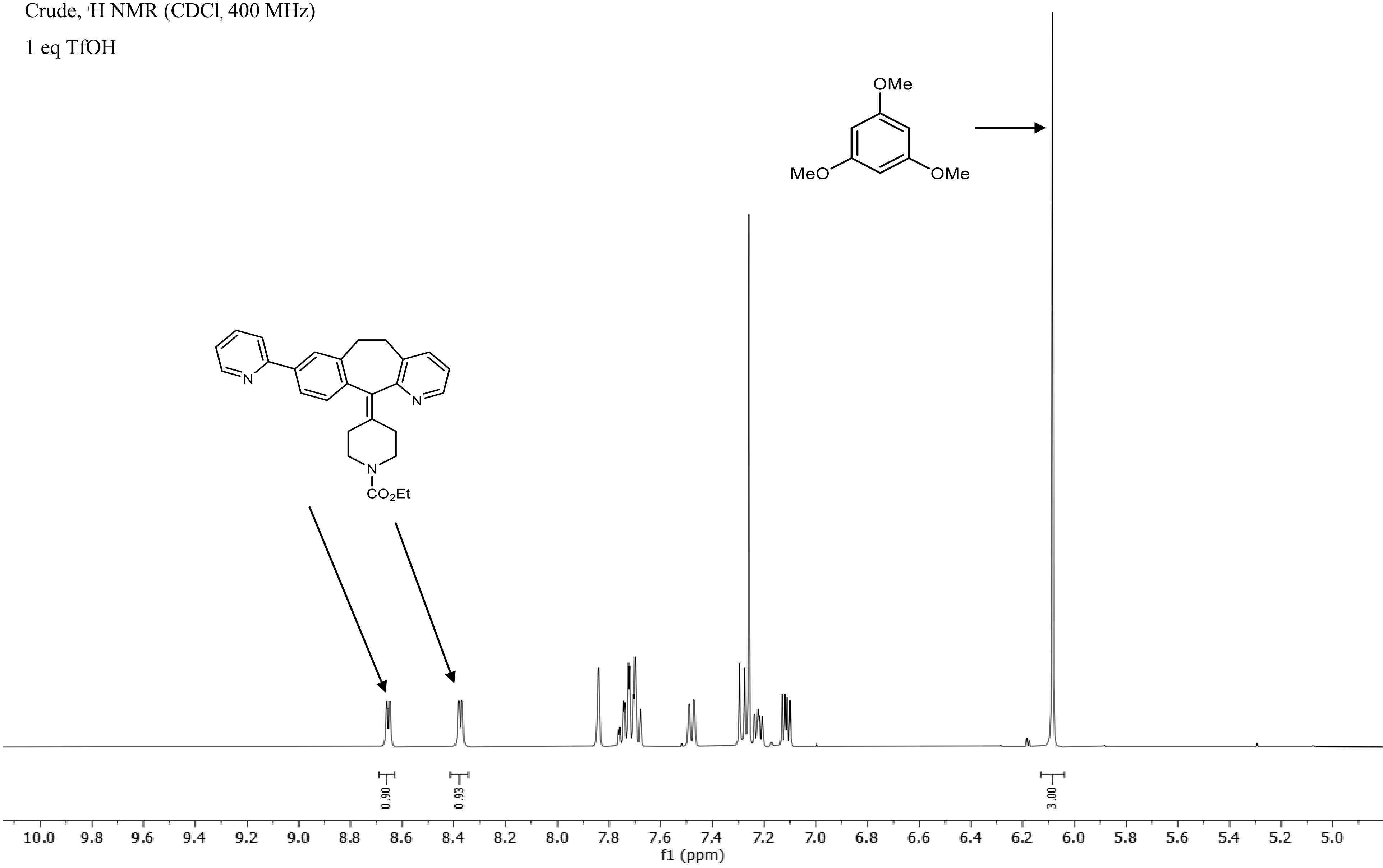

Crude, ^{1}H NMR ($CDCl_3$, 400 MHz)

2 eq TfOH

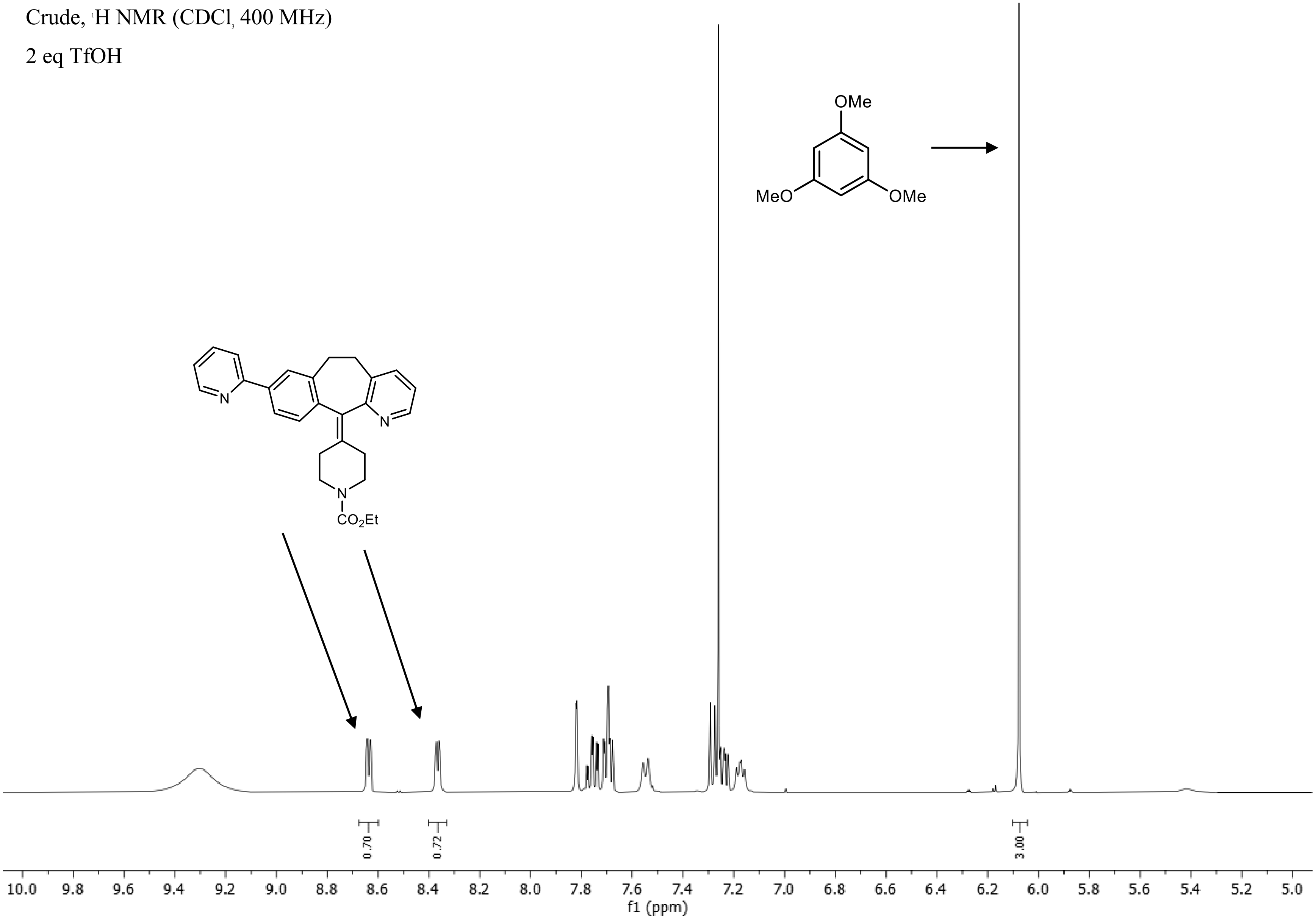

Crude, ^{1}H NMR ($CDCl_3$, 400 MHz); Tentative assignment of compounds

No TfOH

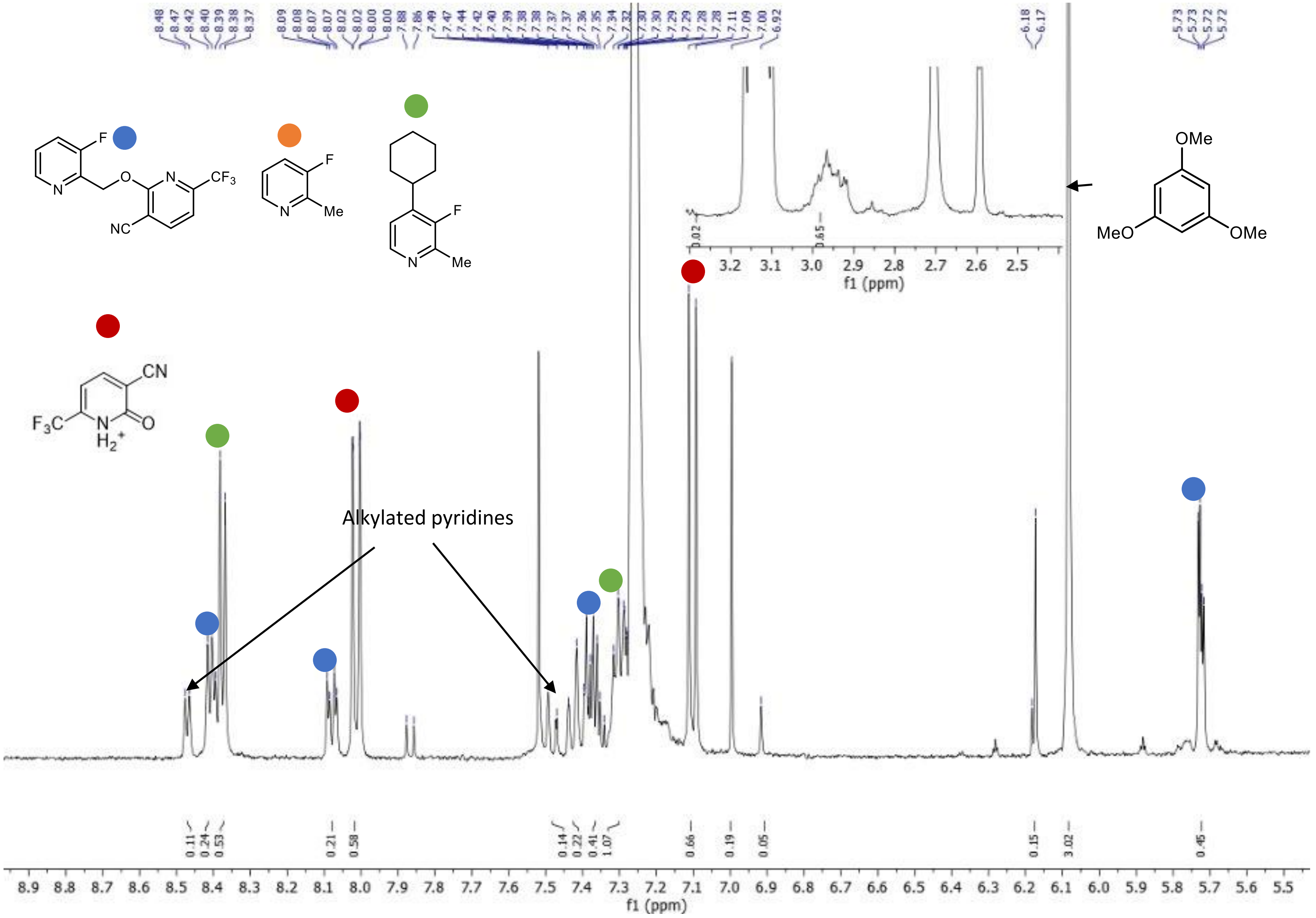

Crude, ^{1}H NMR ($CDCl_3$, 400 MHz); Tentative assignment of compounds

1eq TfOH

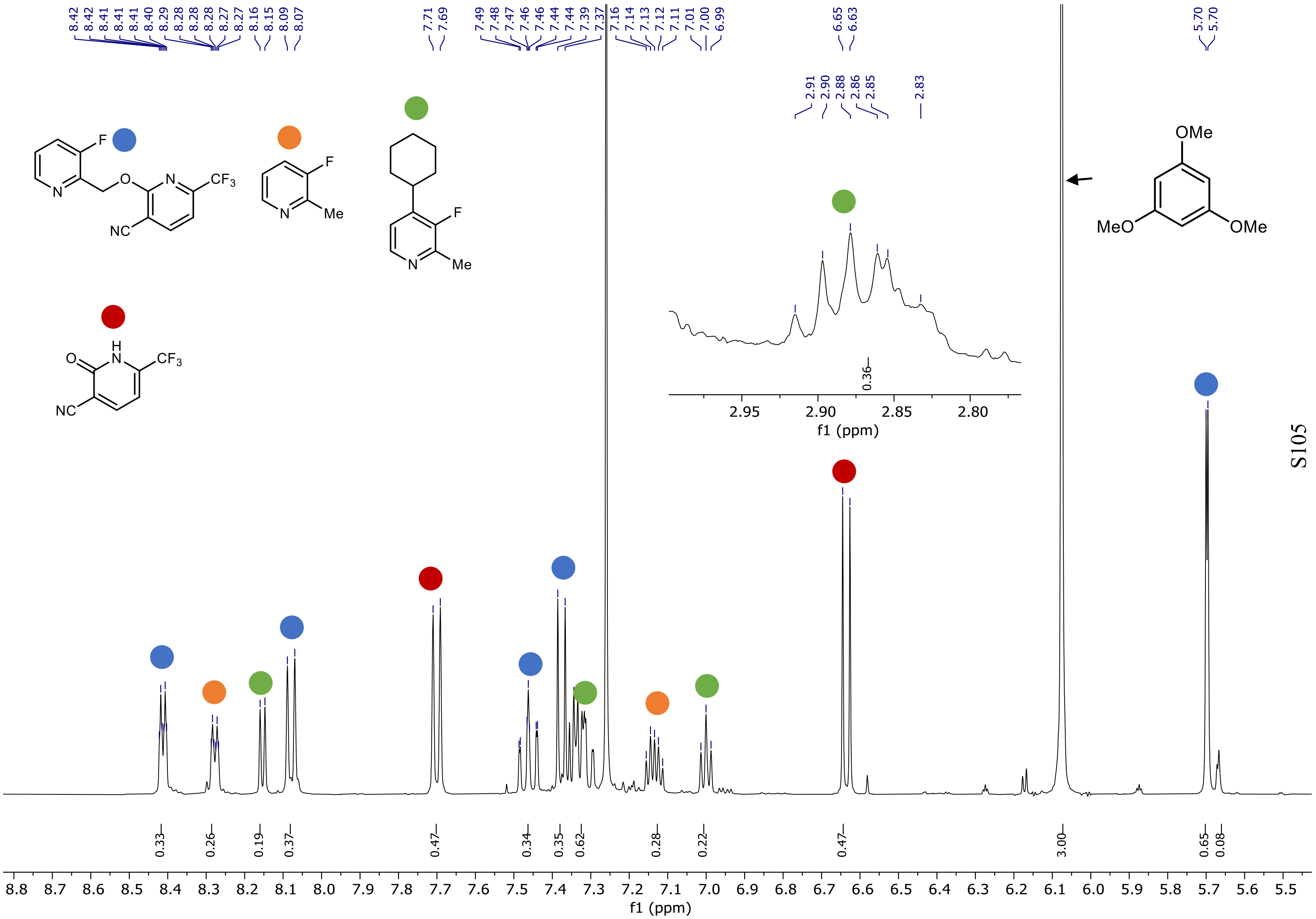

Crude, H NMR (CDCl, 400 MHz); Tentative assignment of compounds

2 eq TfOH

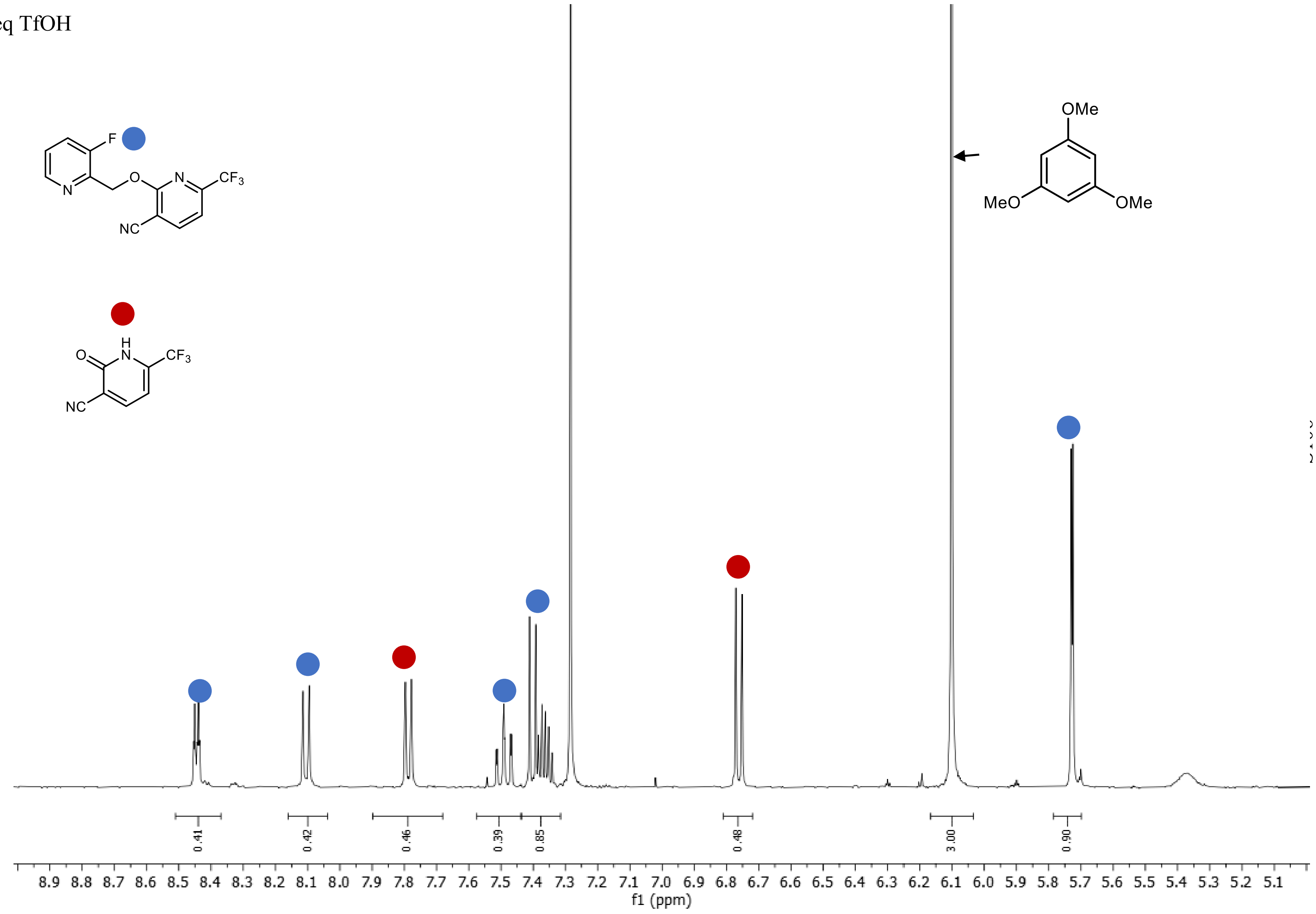

Crude, 1H NMR (CDCl3 400 MHz)

1 eq TFA

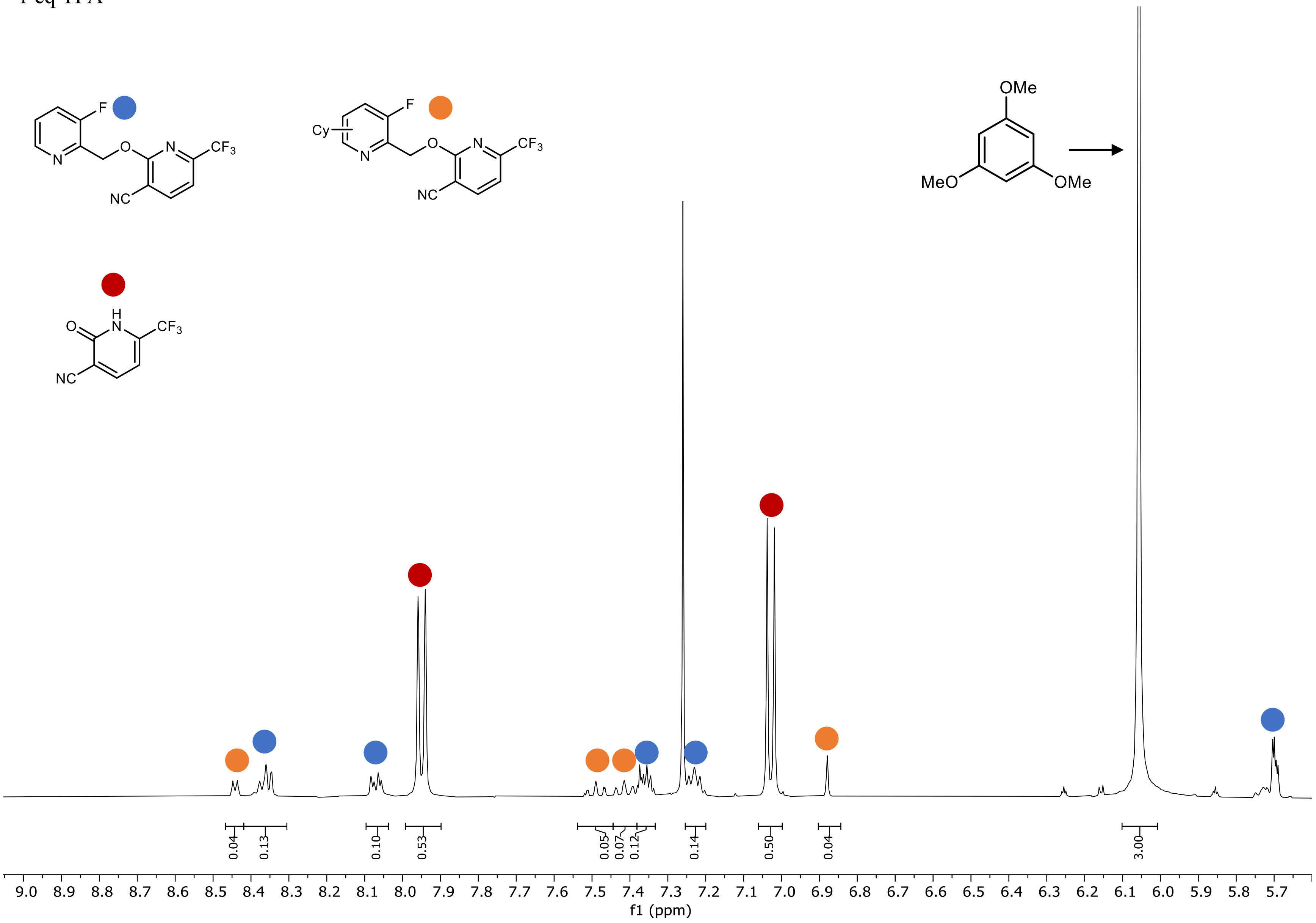

Crude, ^{1}H NMR (CDCl$_3$, 400 MHz)

2 eq TFA

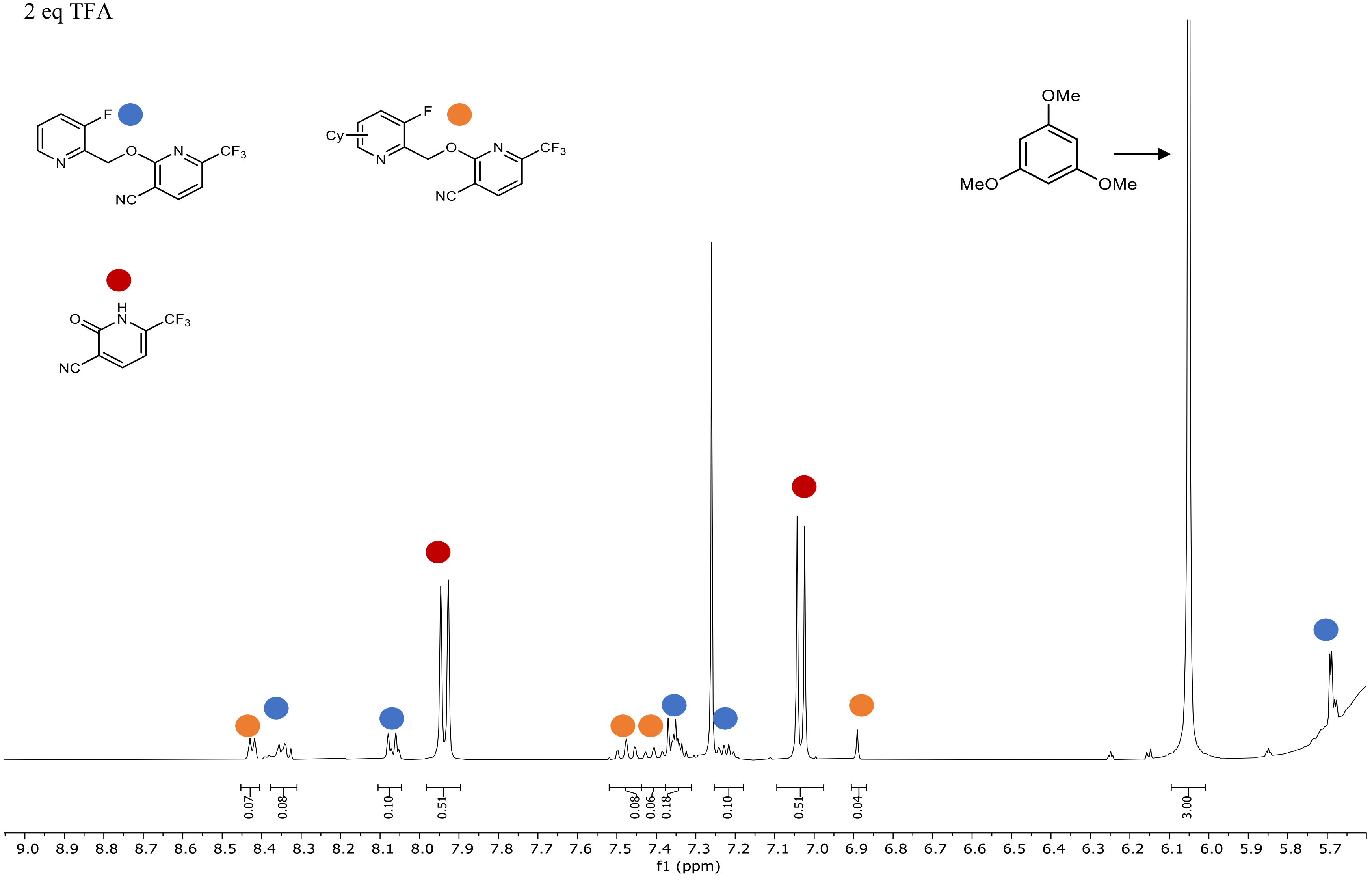

Crude, 1H NMR (CDCl3 400 MHz)

No acid

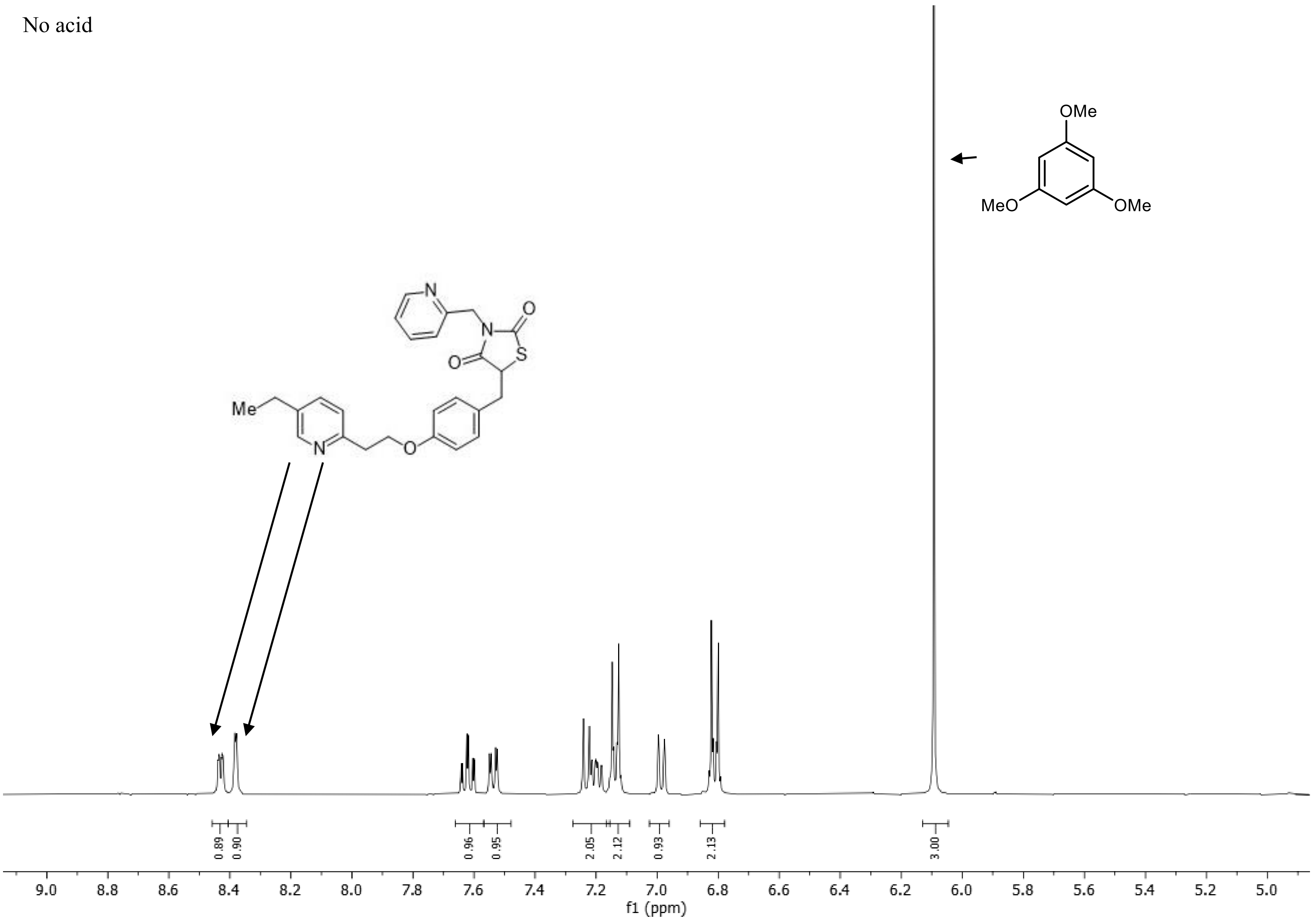

Crude, 1H NMR (CDCl3 400 MHz)

1 eq TfOH

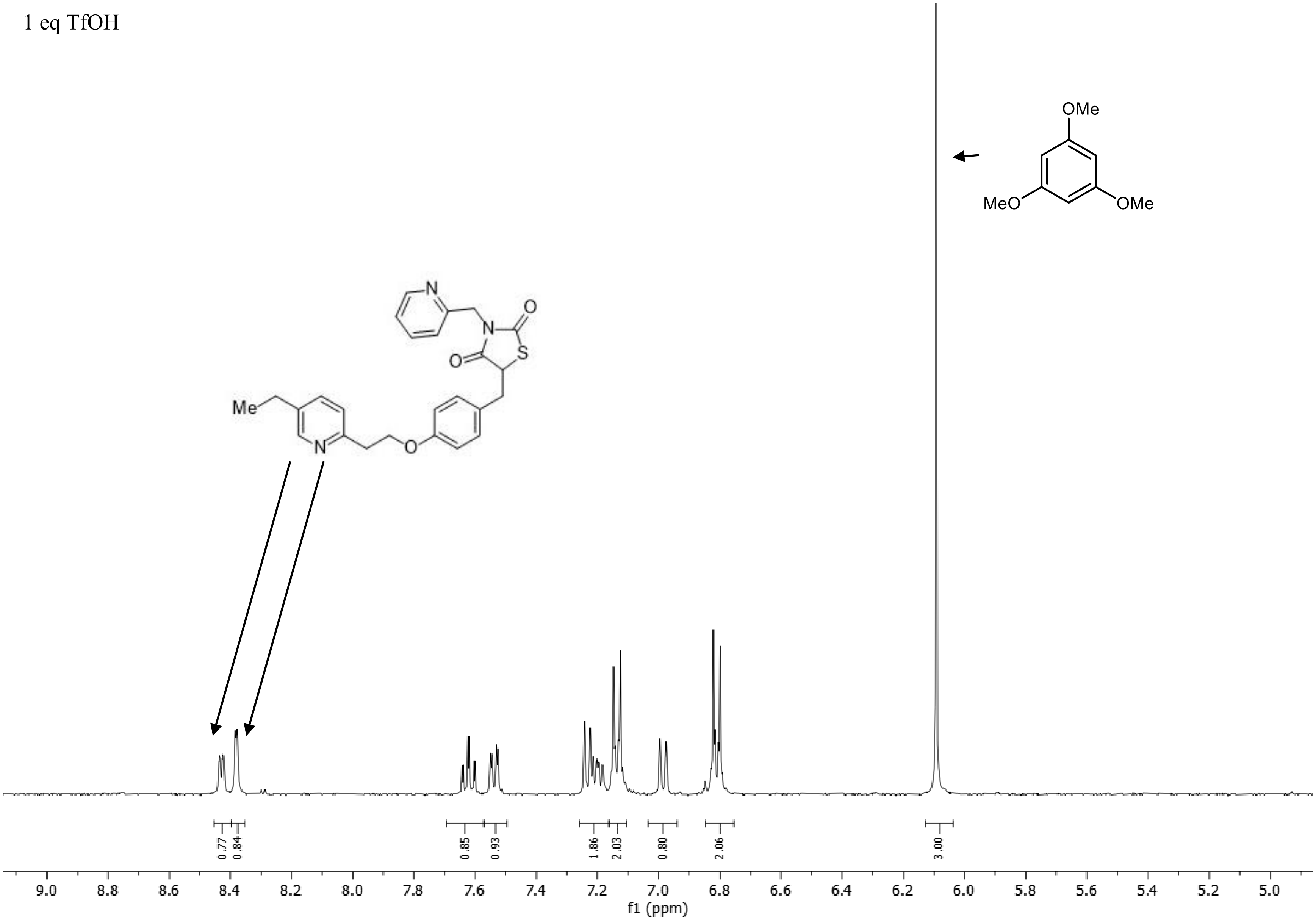

Crude, ^{1}H NMR (CDCl$_3$, 400 MHz)

2 eq TfOH

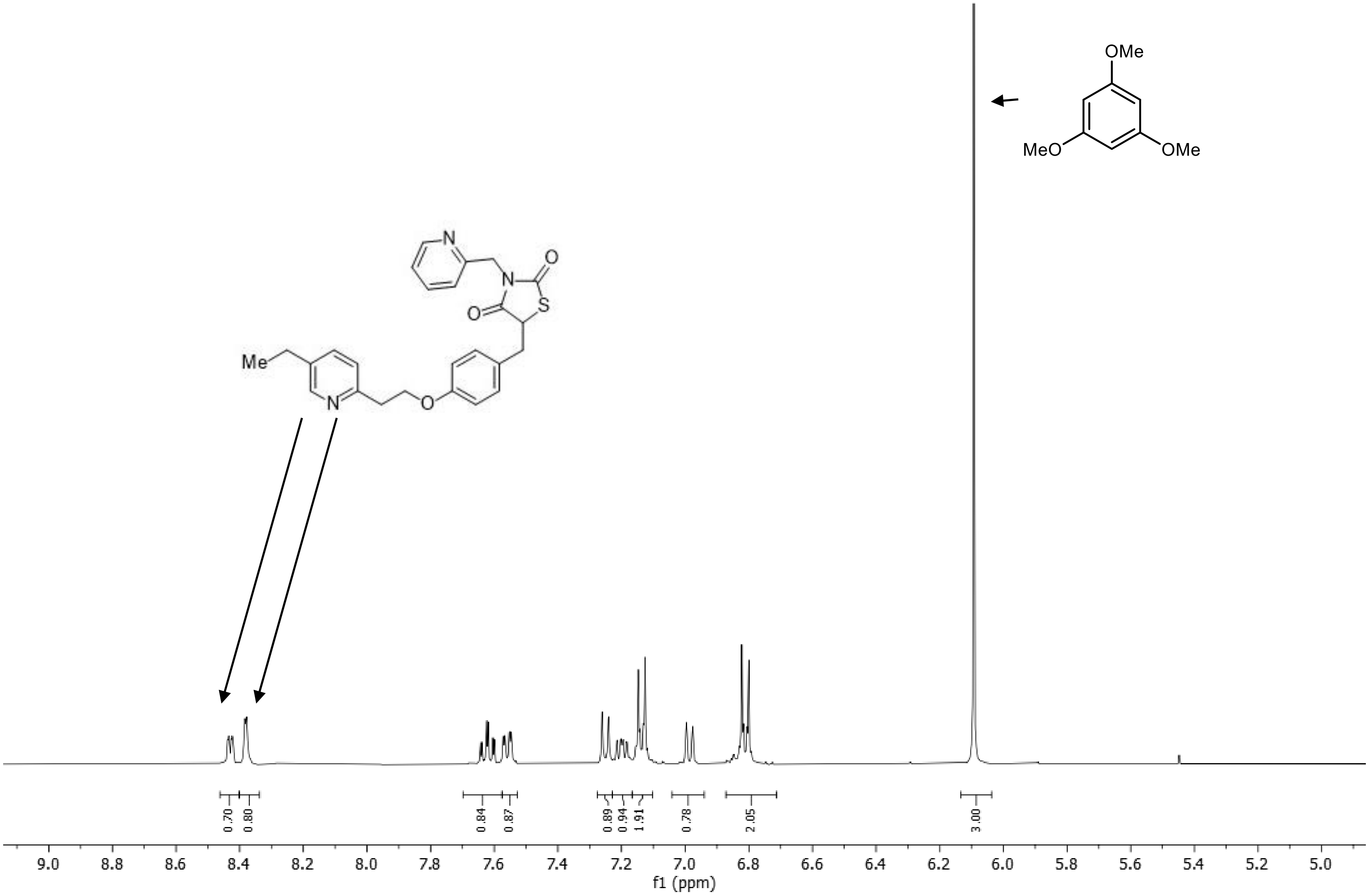

Crude, H NMR (CDCl 400 MHz)

1 eq TFA

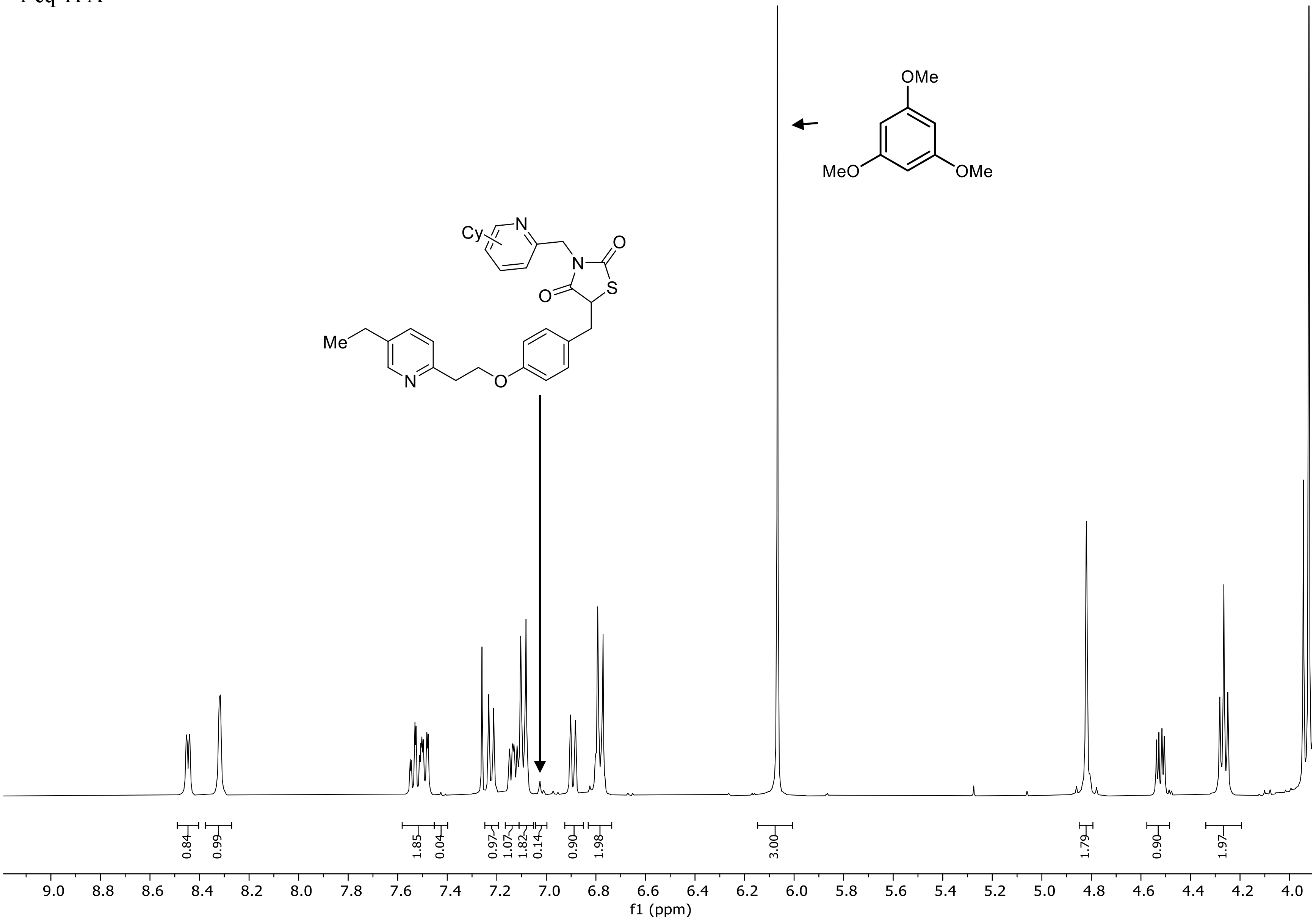

^{1}H NMR (CDCl, 400 MHz)

2 eq TFA

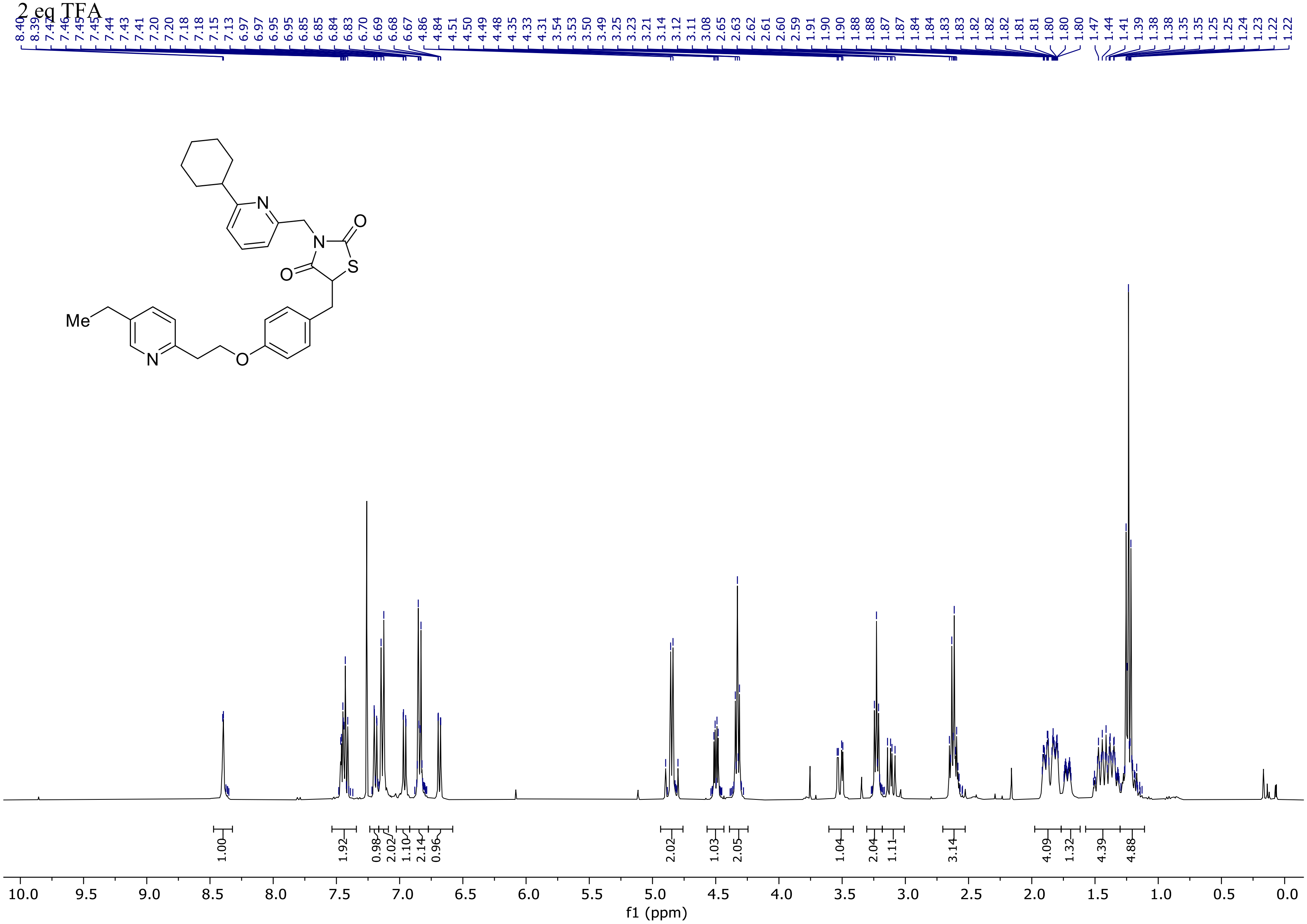

1.9 ^{1}H, ^{13}C, ^{19}F, and ^{31}P Spectra

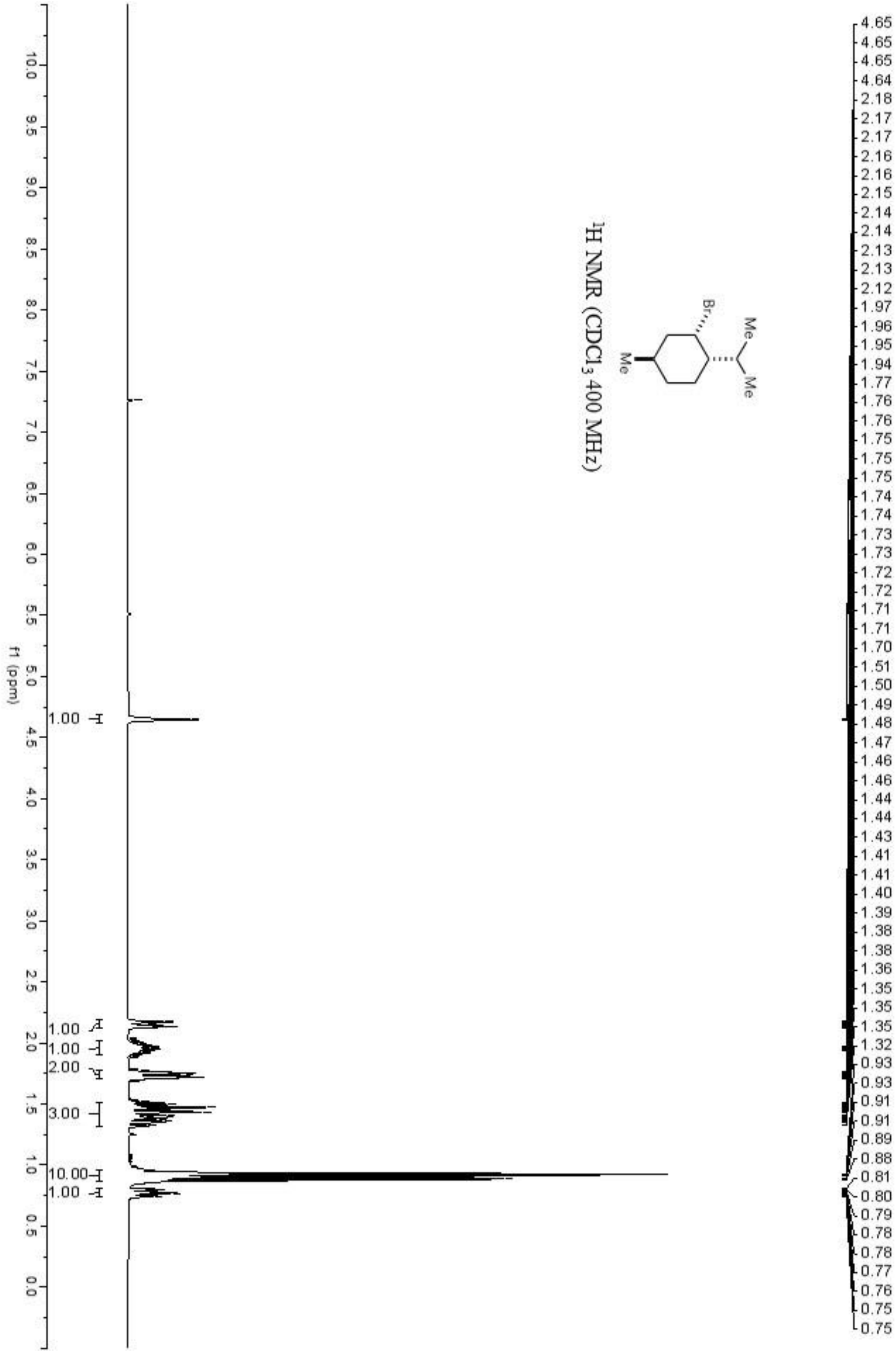

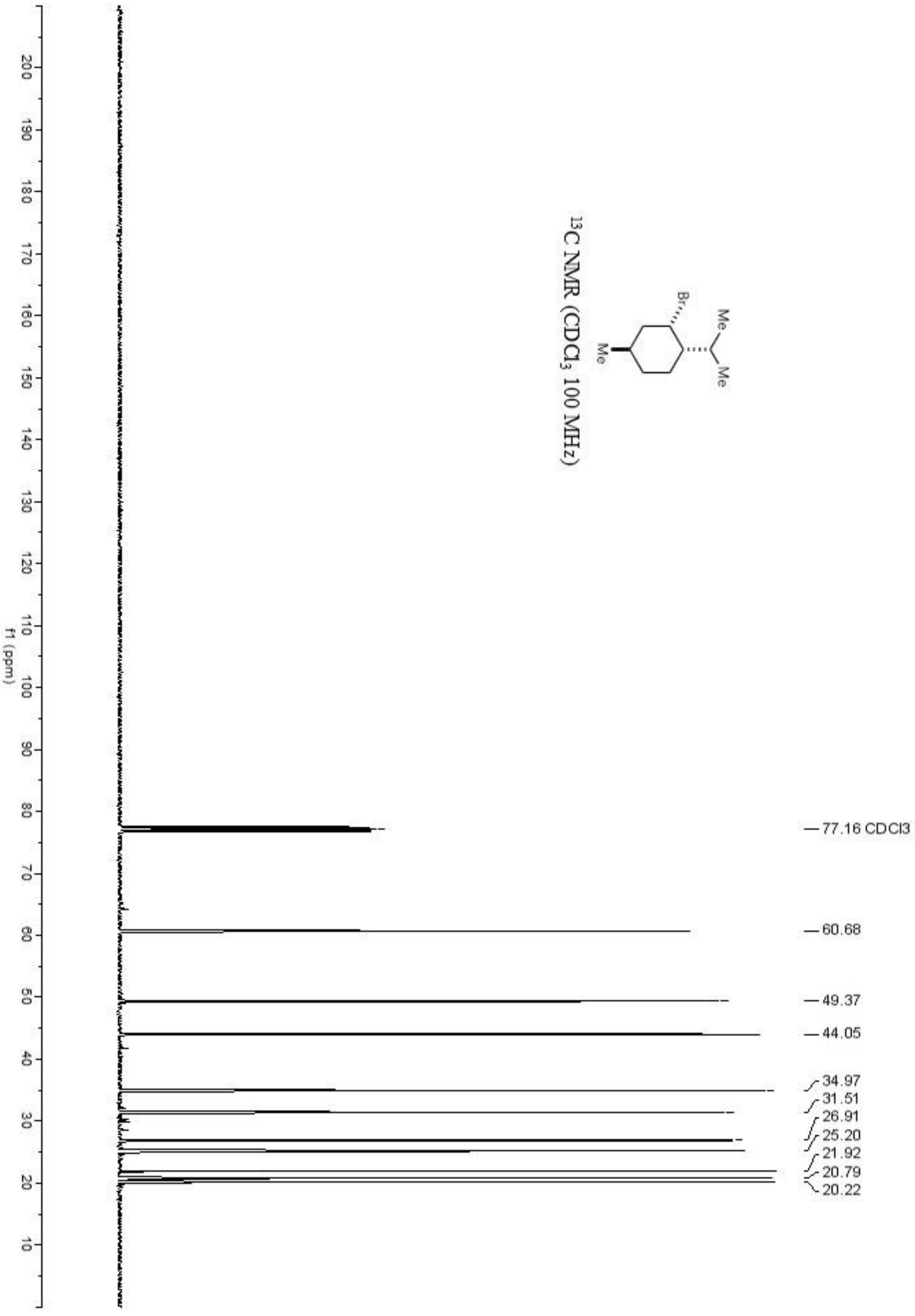

13C NMR (CDCl3 100 MHz)
Br
Me
Me
Me
77.16 CDCl3
60.68
49.37
44.05
34.97
31.51
26.91
25.20
21.92
20.79
20.22
f1 (ppm)

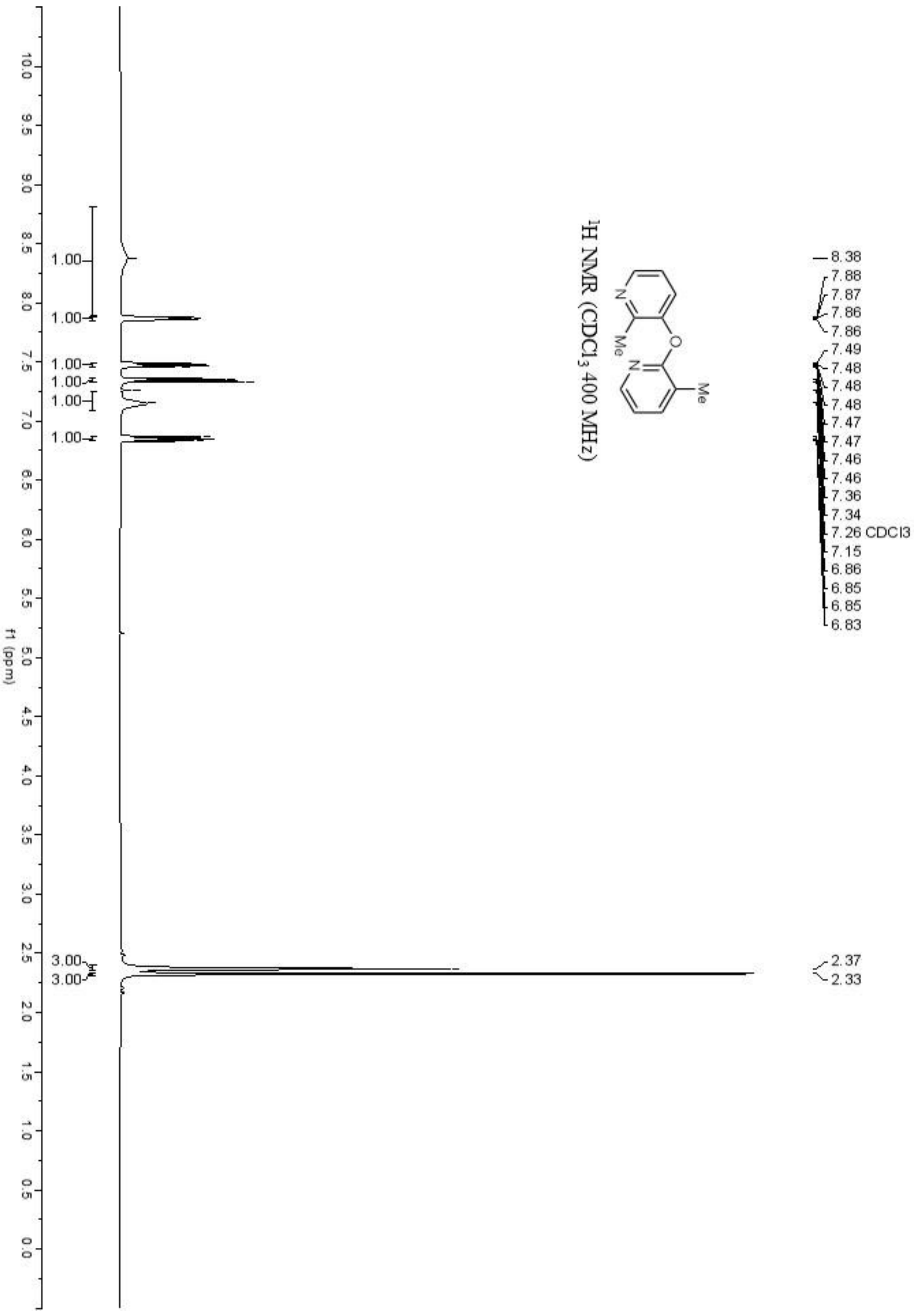

¹H NMR (CDCl3 400 MHz)
Me
N
O
N
Me
8.38
7.88
7.87
7.86
7.86
7.49
7.48
7.48
7.48
7.47
7.47
7.46
7.46
7.36
7.34
7.26 CDCl3
7.15
6.86
6.85
6.85
6.83
2.37
2.33
1.00
1.00
1.00
1.00
1.00
1.00
3.00
3.00
f1 (ppm)
10.0
9.5
9.0
8.5
8.0
7.5
7.0
6.5
6.0
5.5
5.0
4.5
4.0
3.5
3.0
2.5
2.0
1.5
1.0
0.5
0.0

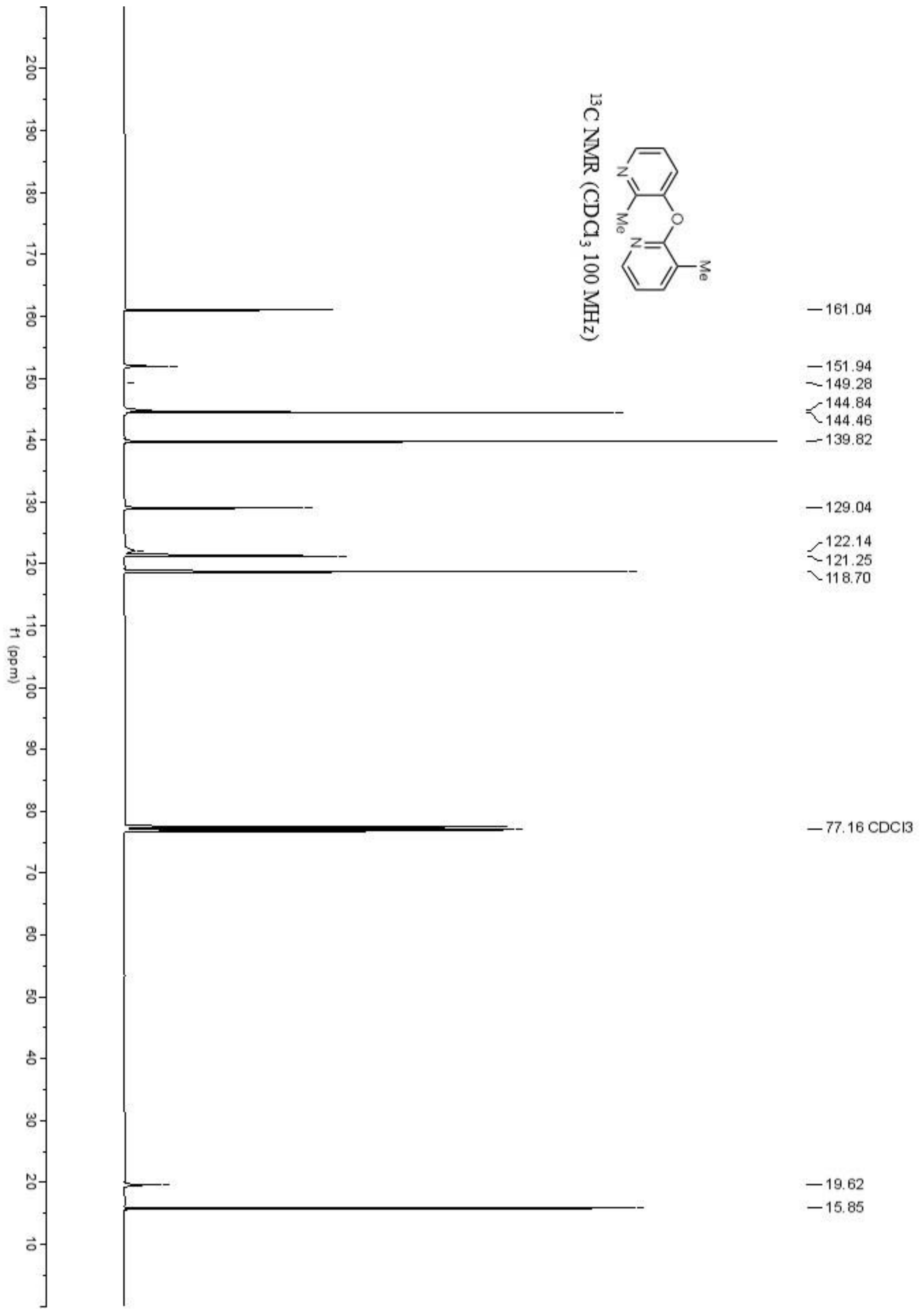

13C NMR (CDCl3 100 MHz)
Me
N
O
N
Me
161.04
151.94
149.28
144.84
144.46
139.82
129.04
122.14
121.25
118.70
77.16 CDCl3
19.62
15.85
f1 (ppm)

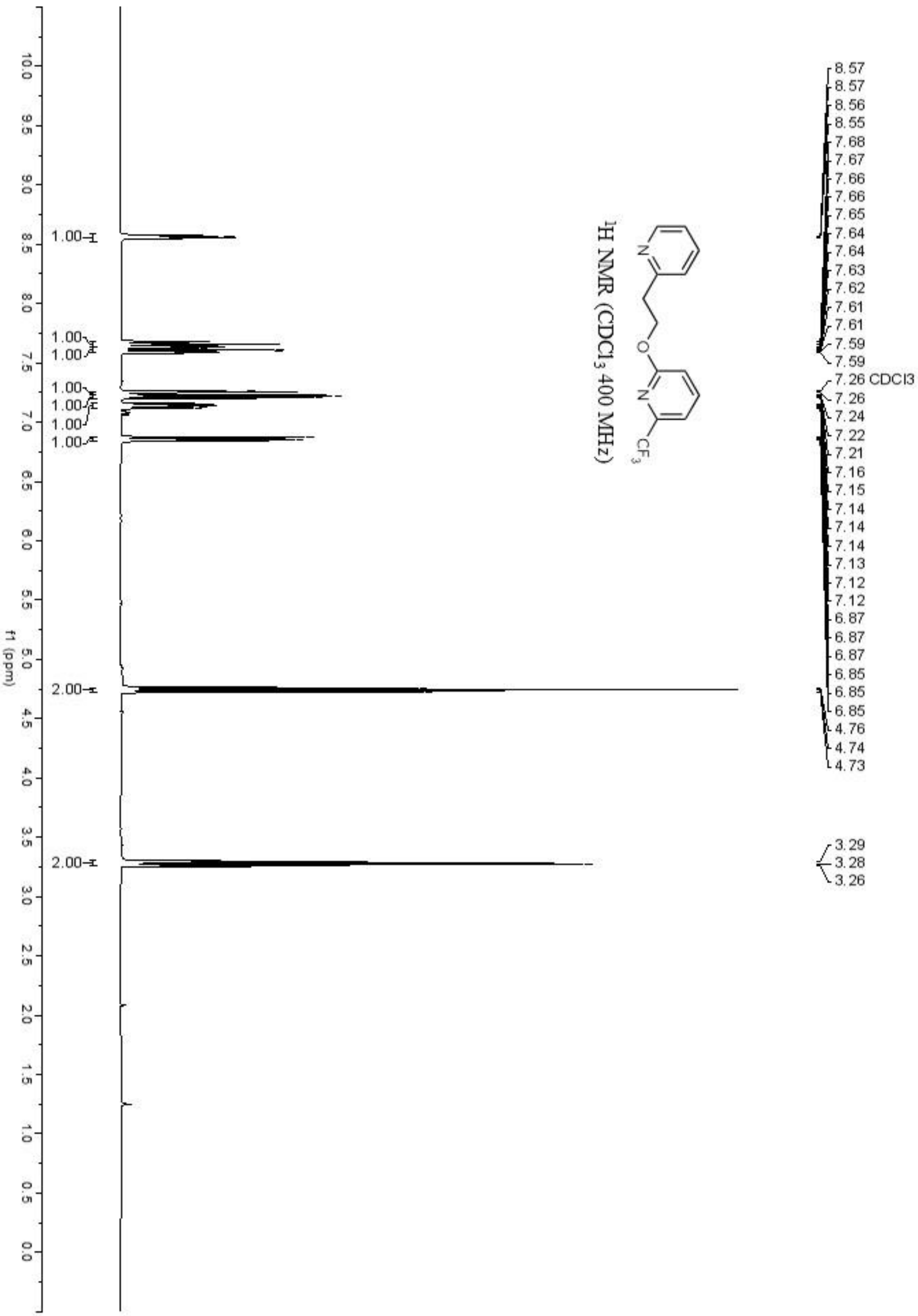

¹H NMR (CDCl3 400 MHz)
CF3
N
O
8.57
8.57
8.56
8.55
7.68
7.67
7.66
7.66
7.65
7.64
7.64
7.63
7.62
7.61
7.61
7.59
7.59
7.26 CDCl3
7.26
7.24
7.22
7.21
7.16
7.15
7.14
7.14
7.14
7.13
7.12
7.12
6.87
6.87
6.87
6.85
6.85
6.85
4.76
4.74
4.73
3.29
3.28
3.26
1.00
1.00
1.00
1.00
1.00
1.00
1.00
2.00
2.00
f1 (ppm)
10.0
9.5
9.0
8.5
8.0
7.5
7.0
6.5
6.0
5.5
5.0
4.5
4.0
3.5
3.0
2.5
2.0
1.5
1.0
0.5
0.0

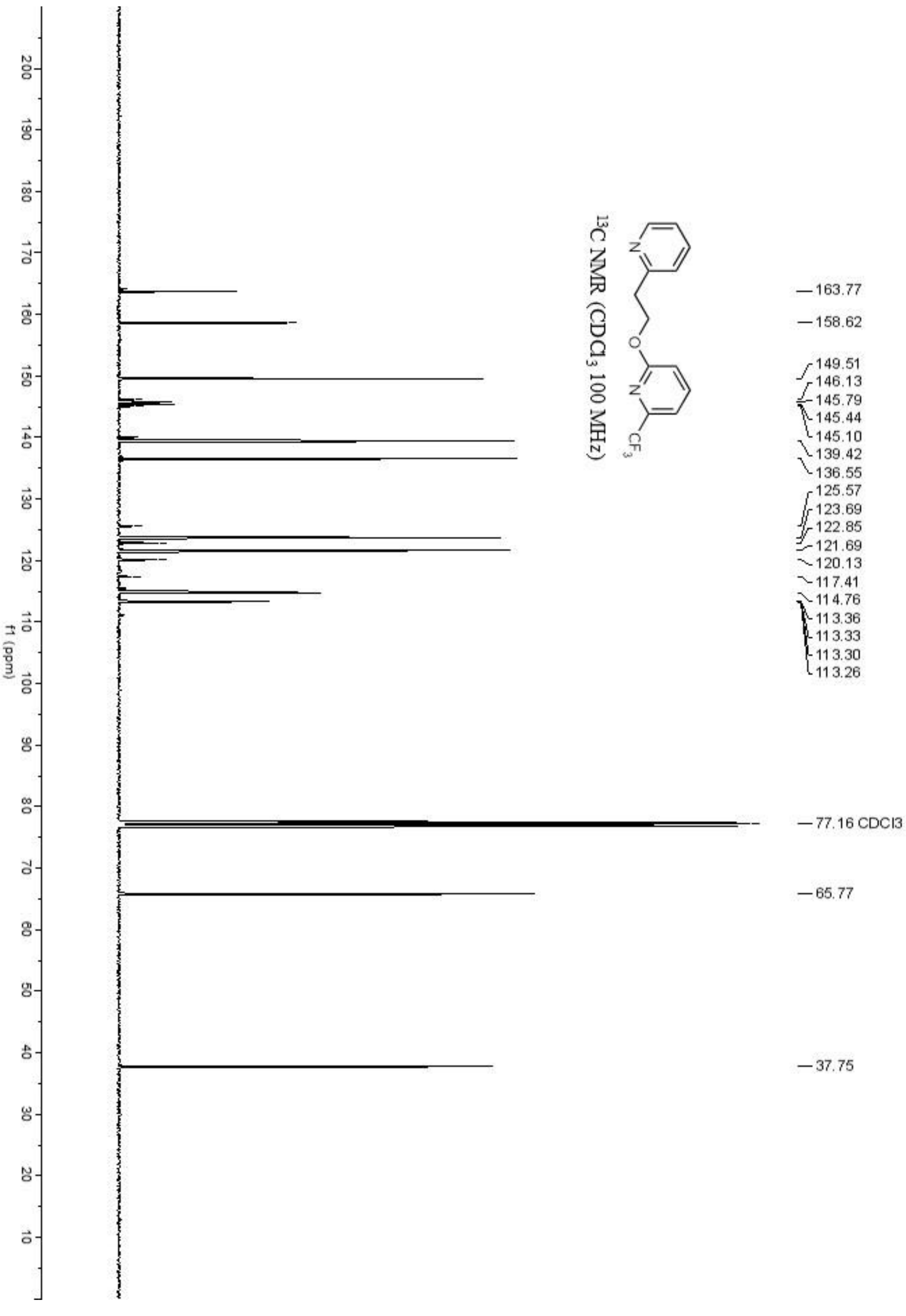

13C NMR (CDCl3 100 MHz)
163.77
158.62
149.51
146.13
145.79
145.44
145.10
139.42
136.55
125.57
123.69
122.85
121.69
120.13
117.41
114.76
113.36
113.33
113.30
113.26
77.16 CDCl3
65.77
37.75
f1 (ppm)

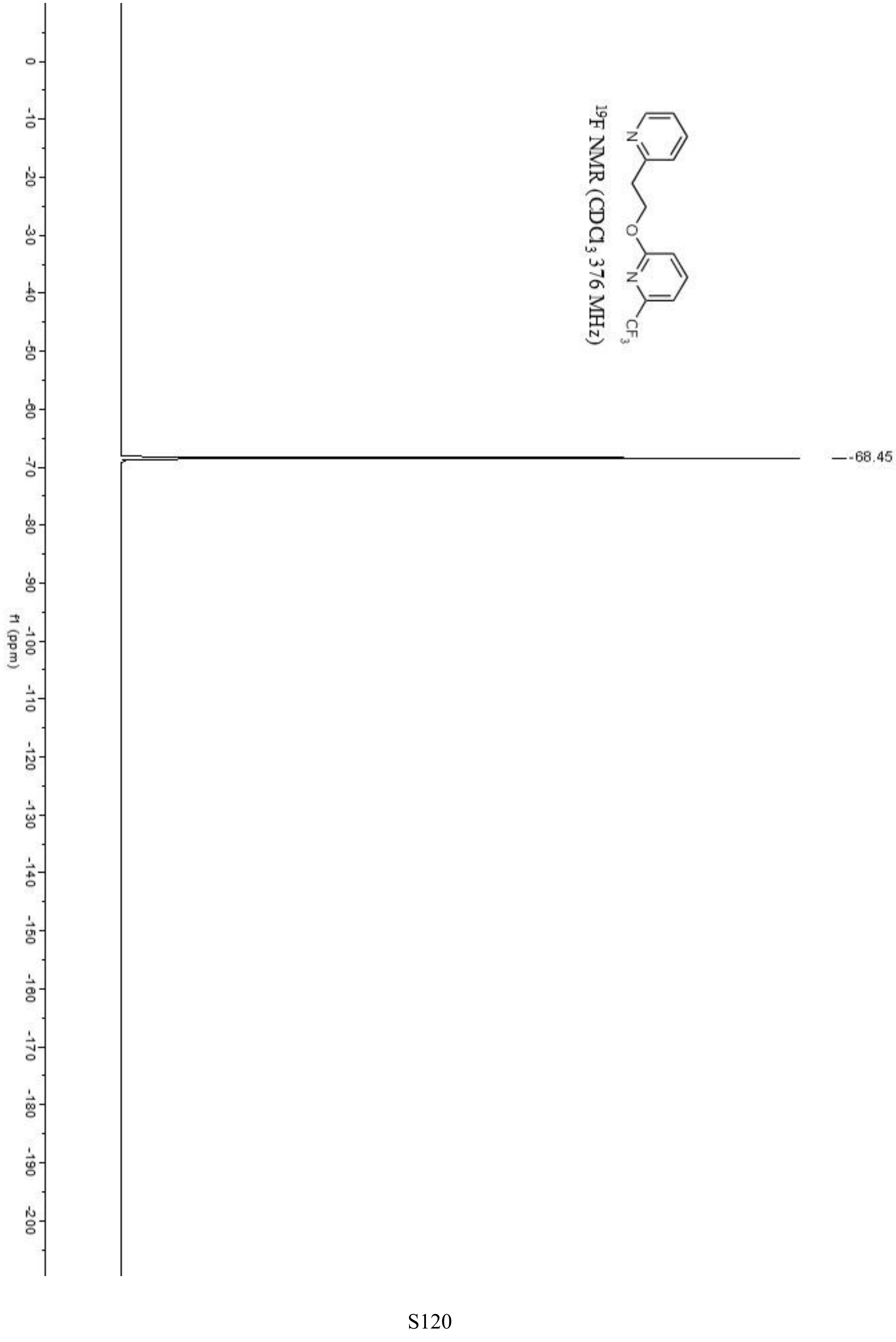

19F NMR (CDCl3 376 MHz)
N
O
N
CF3
--68.45
0
-10
-20
-30
-40
-50
-60
-70
-80
-90
-100
-110
-120
-130
-140
-150
-160
-170
-180
-190
-200
f1 (ppm)

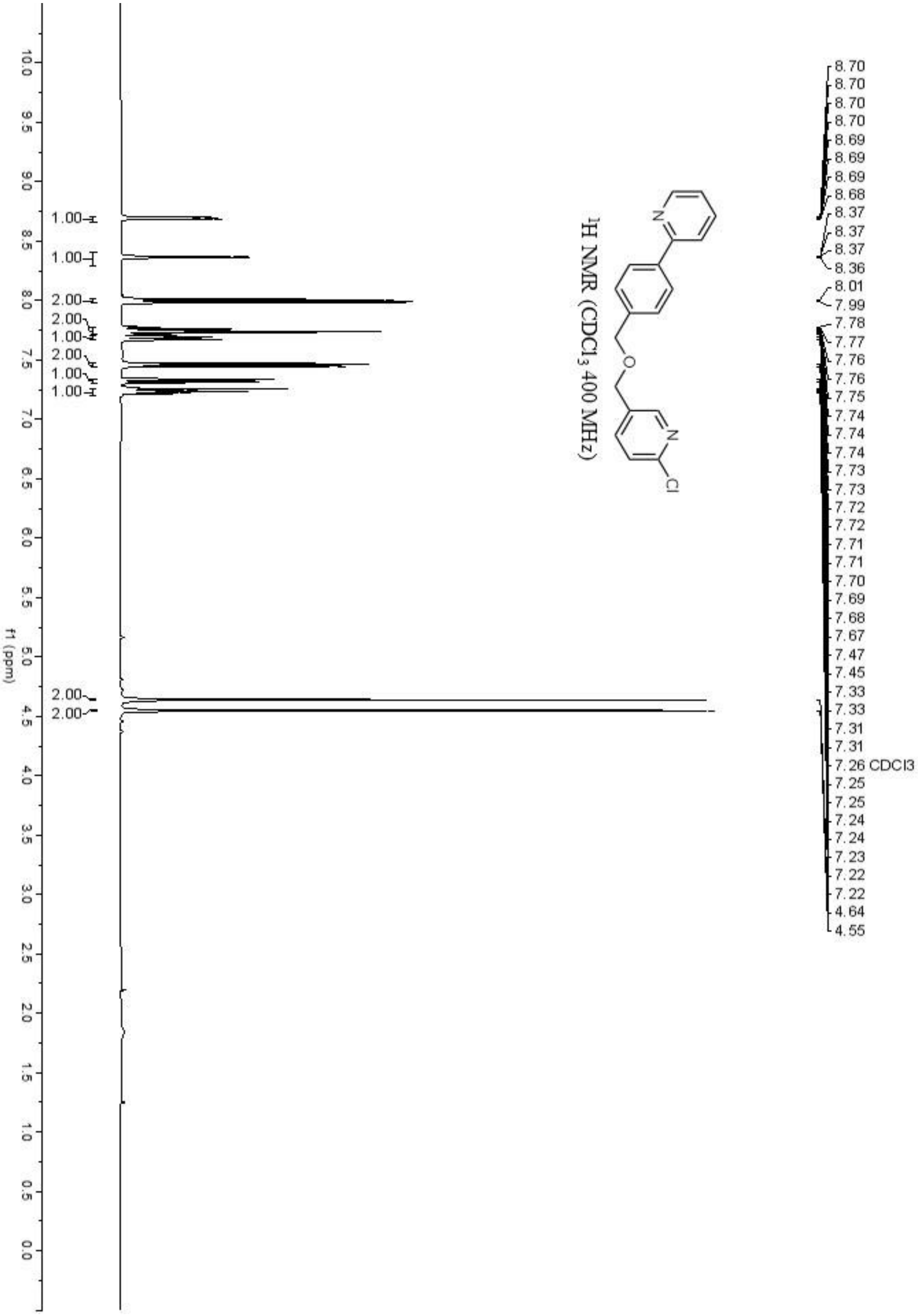

1H NMR (CDCl3 400 MHz)
f1 (ppm)
8.70
8.70
8.70
8.70
8.69
8.69
8.69
8.68
8.37
8.37
8.37
8.36
8.01
7.99
7.78
7.77
7.76
7.76
7.75
7.74
7.74
7.74
7.73
7.73
7.72
7.72
7.71
7.71
7.70
7.69
7.68
7.67
7.47
7.45
7.33
7.33
7.31
7.31
7.26 CDCl3
7.25
7.25
7.24
7.24
7.23
7.22
7.22
4.64
4.55

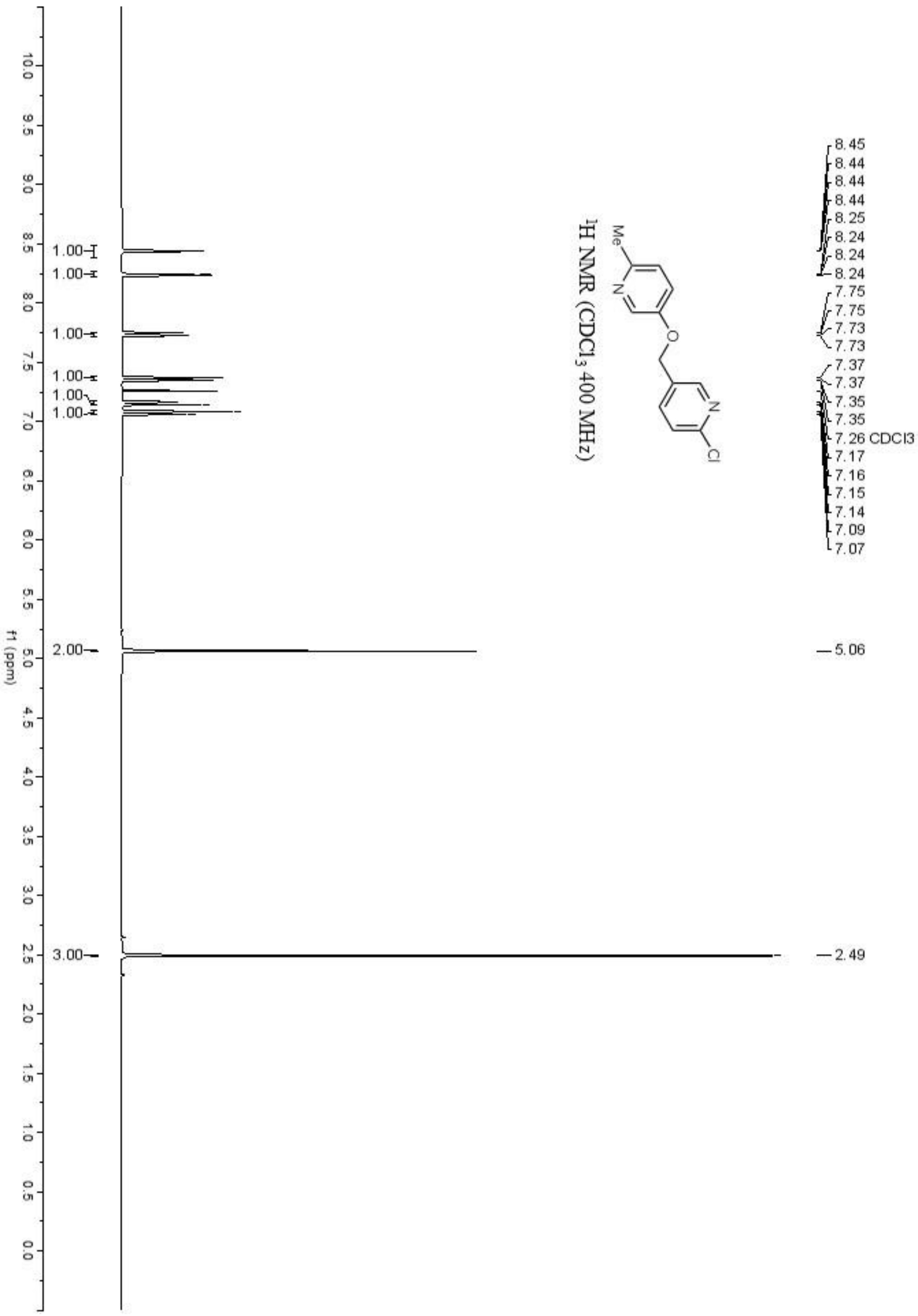

1H NMR (CDCl3 400 MHz)
Me
N
O
N
Cl
8.45
8.44
8.44
8.44
8.25
8.24
8.24
8.24
7.75
7.75
7.73
7.73
7.37
7.37
7.35
7.35
7.26 CDCl3
7.17
7.16
7.15
7.14
7.09
7.07
5.06
2.49
1.00
1.00
1.00
1.00
1.00
1.00
2.00
3.00
f1 (ppm)

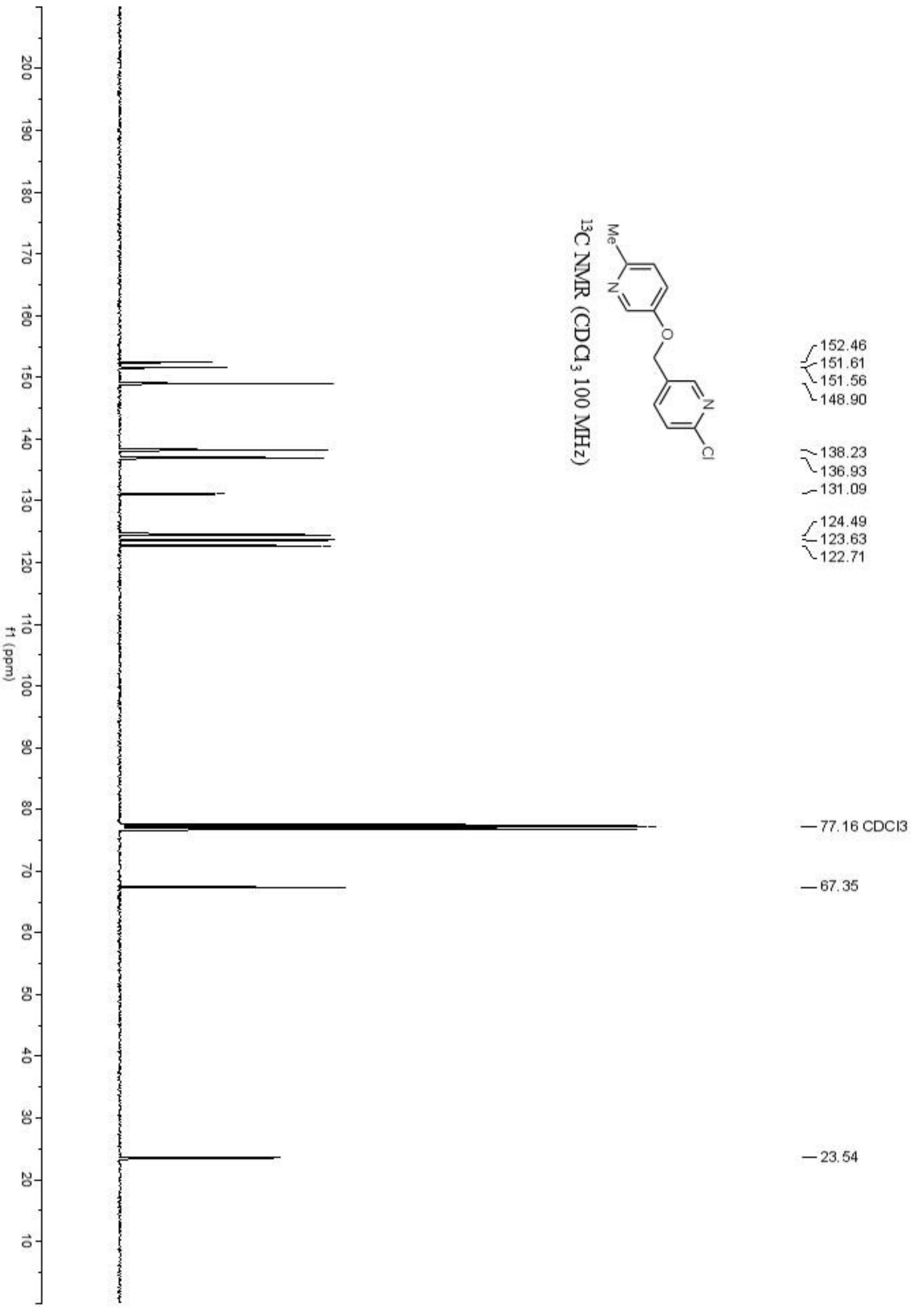

Me
N
O
N
Cl
13C NMR (CDCl3 100 MHz)
152.46
151.61
151.56
148.90
138.23
136.93
131.09
124.49
123.63
122.71
77.16 CDCl3
67.35
23.54
200
190
180
170
160
150
140
130
120
110
100
90
80
70
60
50
40
30
20
10
f1 (ppm)

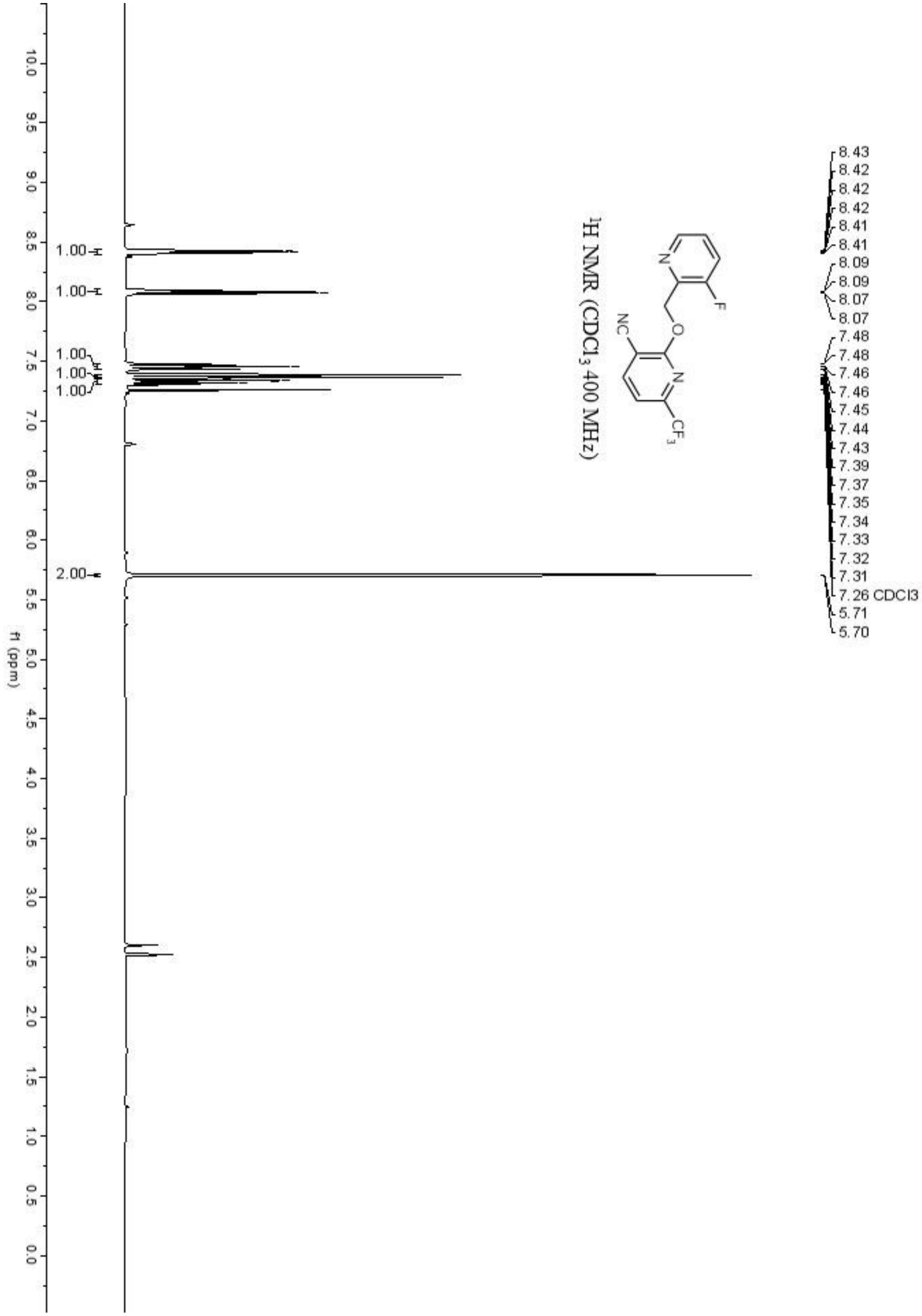
1H NMR (CDCl3 400 MHz)
NC
O
N
F
N
CF3
8.43
8.42
8.42
8.42
8.41
8.41
8.09
8.09
8.07
8.07
7.48
7.48
7.46
7.46
7.45
7.44
7.43
7.39
7.37
7.35
7.34
7.33
7.32
7.31
7.26 CDCl3
5.71
5.70
1.00
1.00
1.00
1.00
1.00
2.00
f1 (ppm)

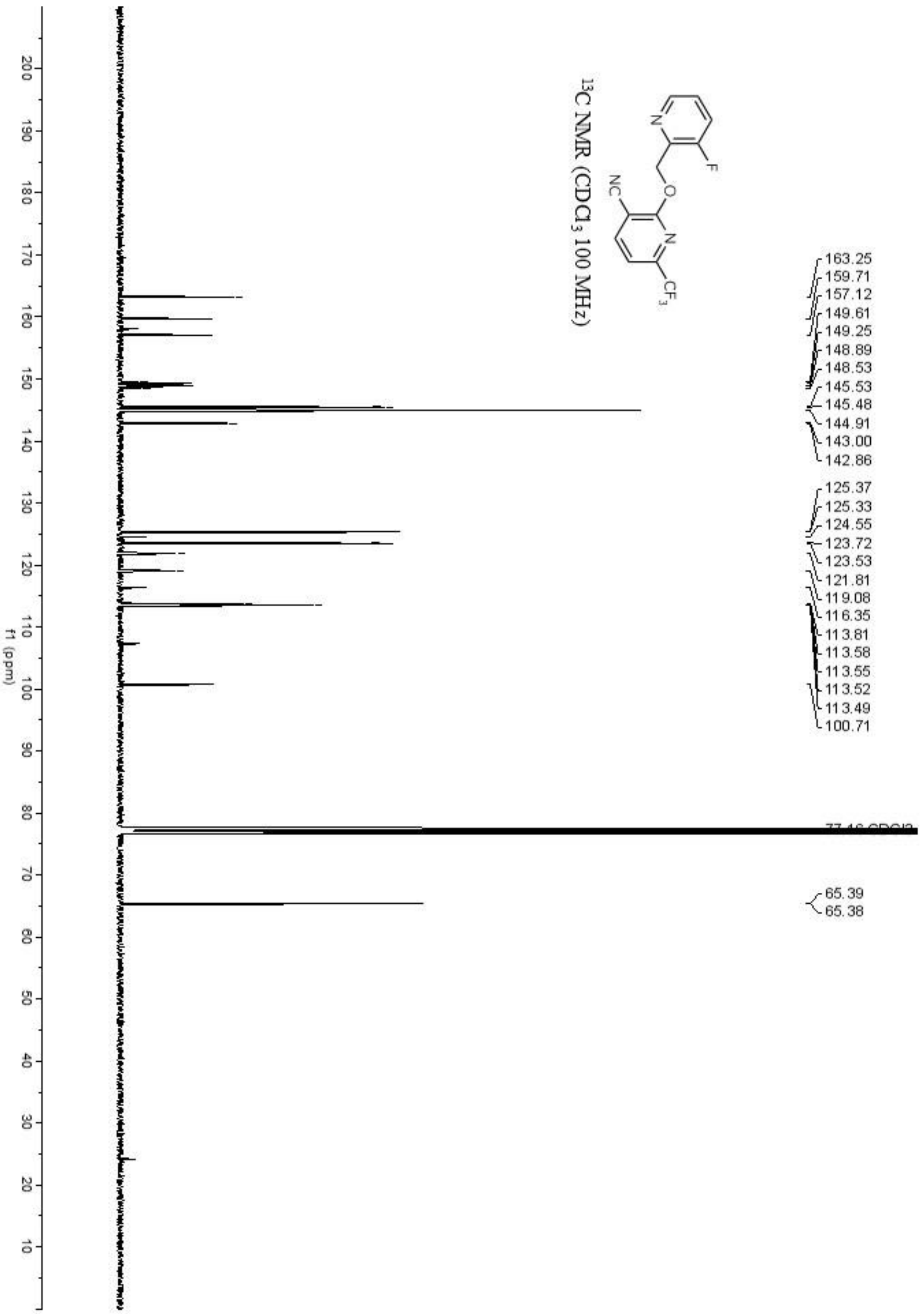

^{13}C NMR (CDCl$_3$ 100 MHz)
NC
O
N
F
N
CF$_3$
163.25
159.71
157.12
149.61
149.25
148.89
148.53
145.53
145.48
144.91
143.00
142.86
125.37
125.33
124.55
123.72
123.53
121.81
119.08
116.35
113.81
113.58
113.55
113.52
113.49
100.71
77.16 CDCl3
65.39
65.38
200
190
180
170
160
150
140
130
120
110
100
90
80
70
60
50
40
30
20
10
f1 (ppm)

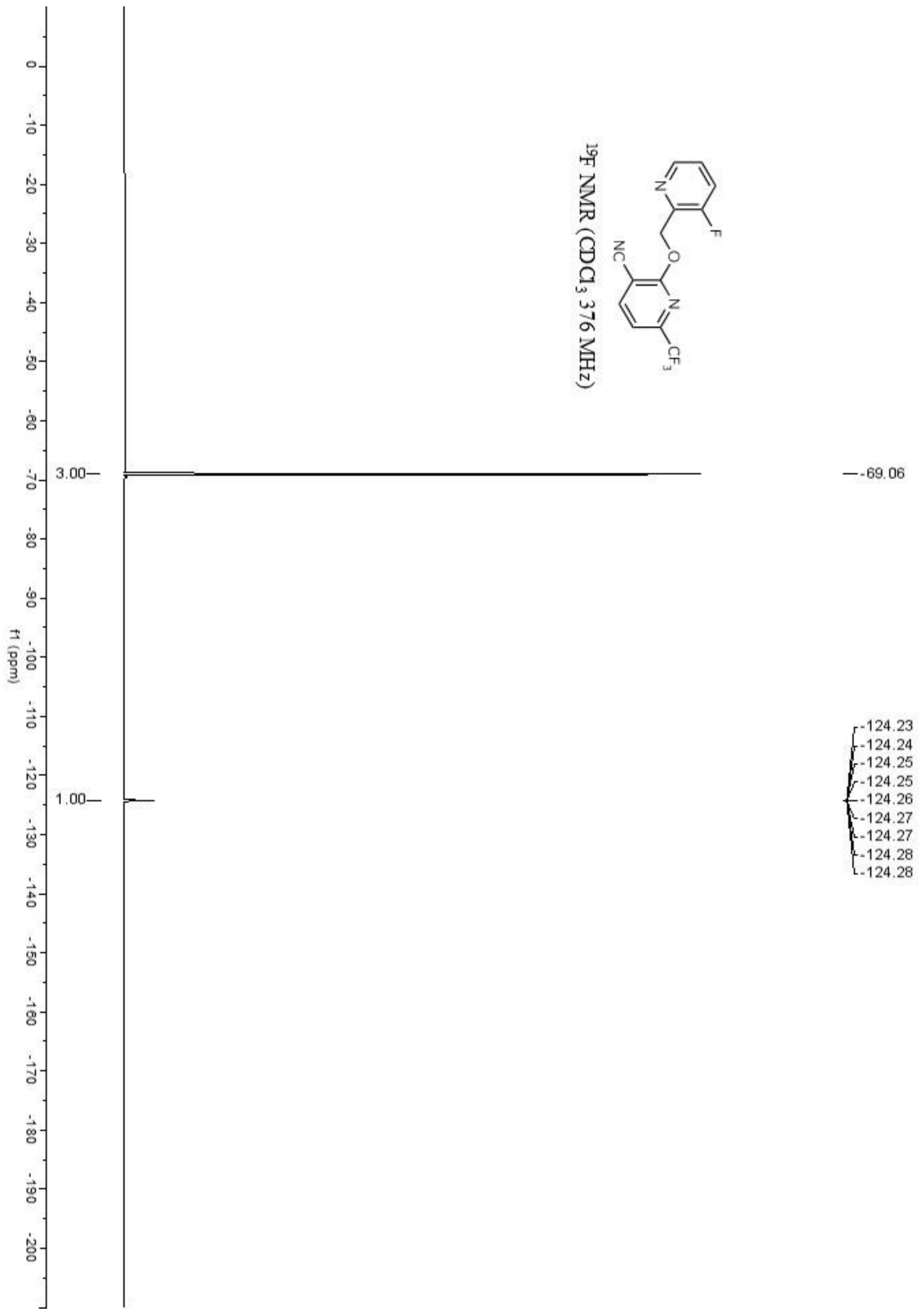

19F NMR (CDCl3 376 MHz)
NC
O
N
F
CF3
3.00
1.00
-69.06
-124.23
-124.24
-124.25
-124.25
-124.26
-124.27
-124.27
-124.28
-124.28
f1 (ppm)

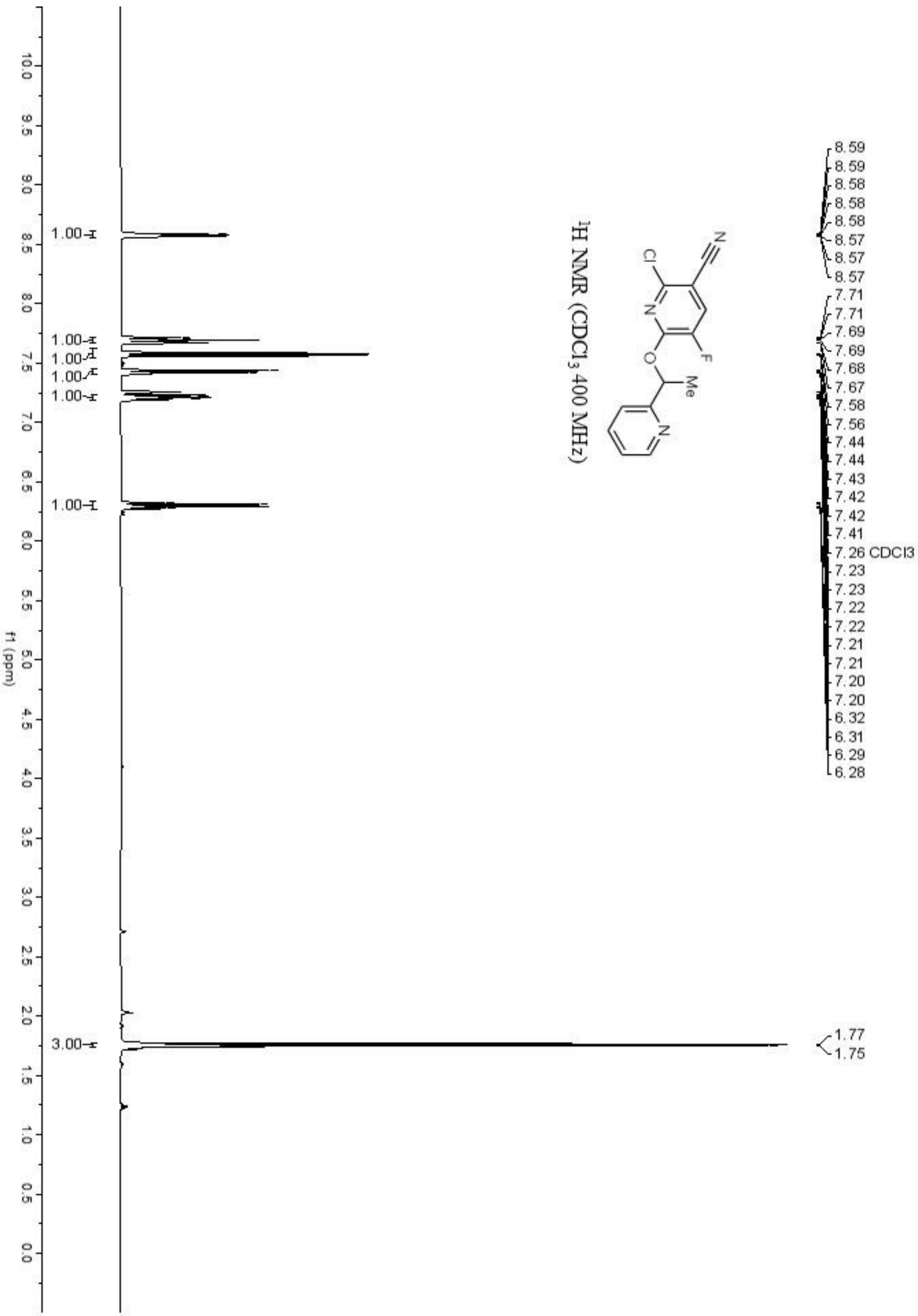

1H NMR (CDCl3 400 MHz)
N
Cl
N
F
O
Me
N
8.59
8.59
8.58
8.58
8.58
8.57
8.57
8.57
7.71
7.71
7.69
7.69
7.68
7.67
7.58
7.56
7.44
7.44
7.43
7.42
7.42
7.41
7.26 CDCl3
7.23
7.23
7.22
7.22
7.21
7.21
7.20
7.20
6.32
6.31
6.29
6.28
1.77
1.75
1.00
1.00
1.00
1.00
1.00
1.00
3.00
f1 (ppm)
10.0
9.5
9.0
8.5
8.0
7.5
7.0
6.5
6.0
5.5
5.0
4.5
4.0
3.5
3.0
2.5
2.0
1.5
1.0
0.5
0.0

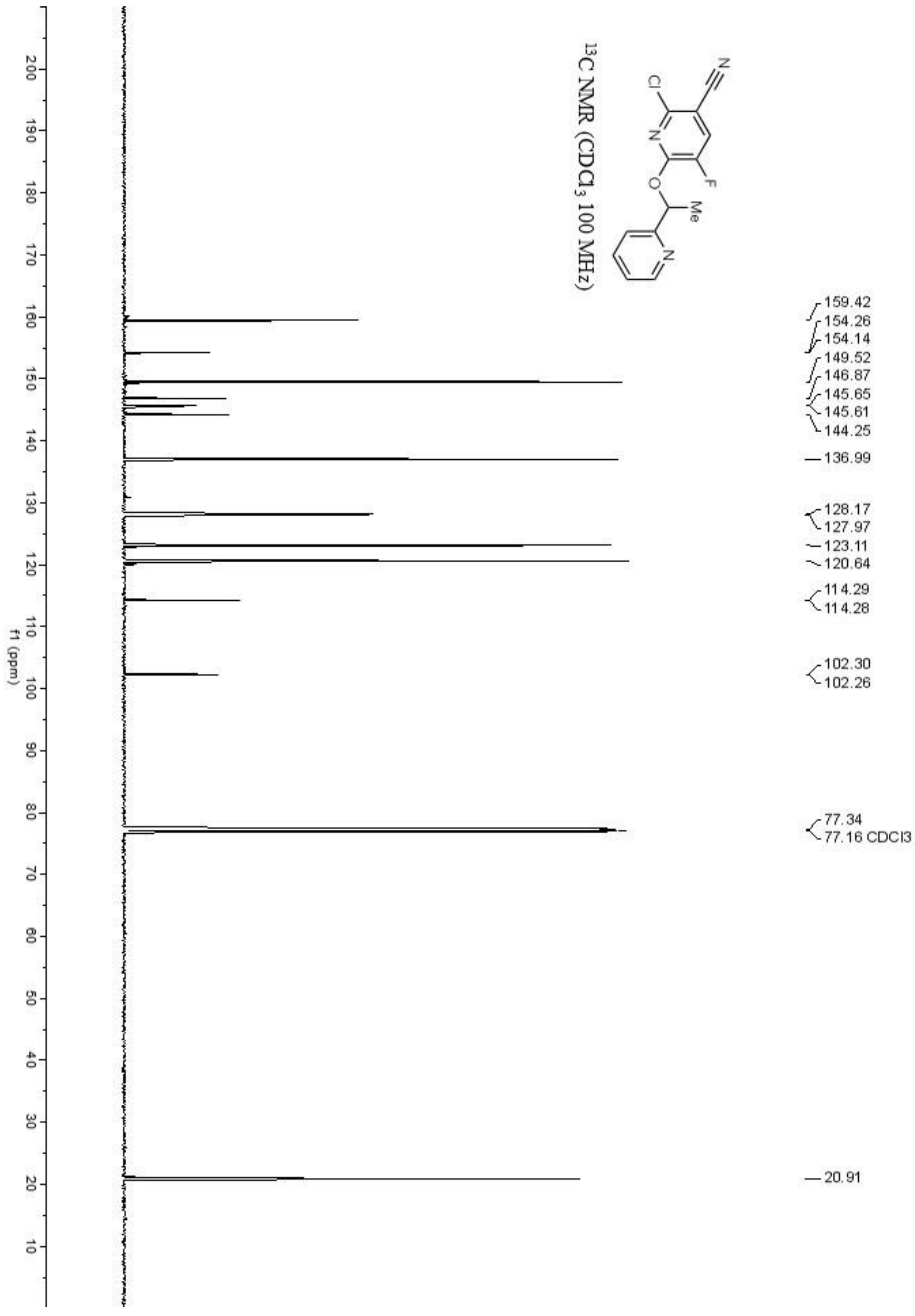

13C NMR (CDCl3 100 MHz)
Cl
N
O
F
Me
N
N
159.42
154.26
154.14
149.52
146.87
145.65
145.61
144.25
136.99
128.17
127.97
123.11
120.64
114.29
114.28
102.30
102.26
77.34
77.16 CDCl3
20.91
f1 (ppm)

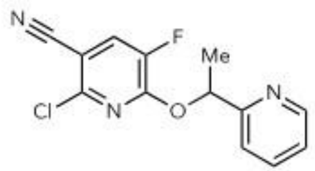

^{19}F NMR ($CDCl_3$ 376 MHz)

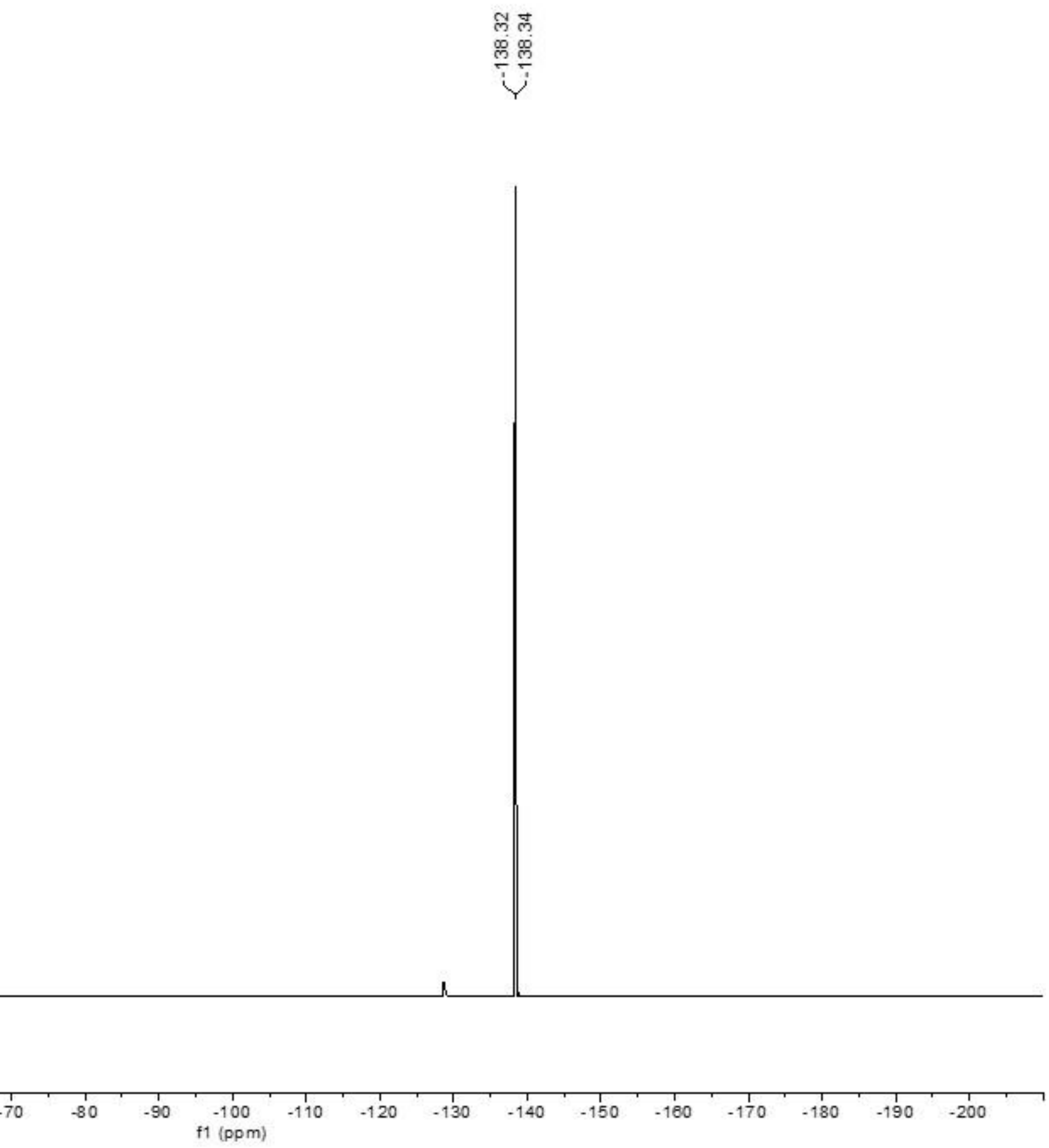

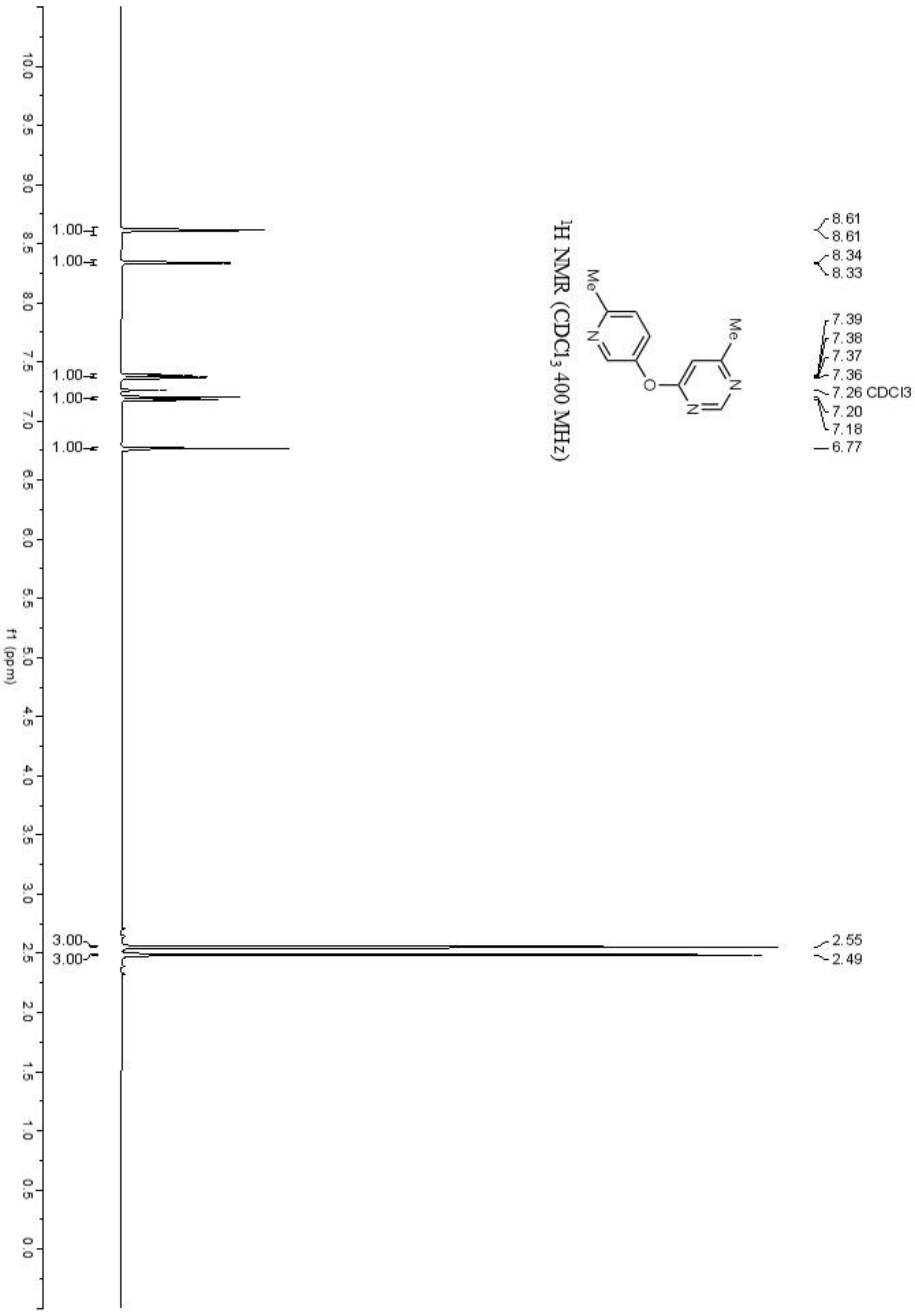

¹H NMR (CDCl3 400 MHz)
Me
N
O
Me
N
N
8.61
8.61
8.34
8.33
7.39
7.38
7.37
7.36
7.26 CDCl3
7.20
7.18
6.77
2.55
2.49
1.00
1.00
1.00
1.00
1.00
3.00
3.00
f1 (ppm)
10.0
9.5
9.0
8.5
8.0
7.5
7.0
6.5
6.0
5.5
5.0
4.5
4.0
3.5
3.0
2.5
2.0
1.5
1.0
0.5
0.0

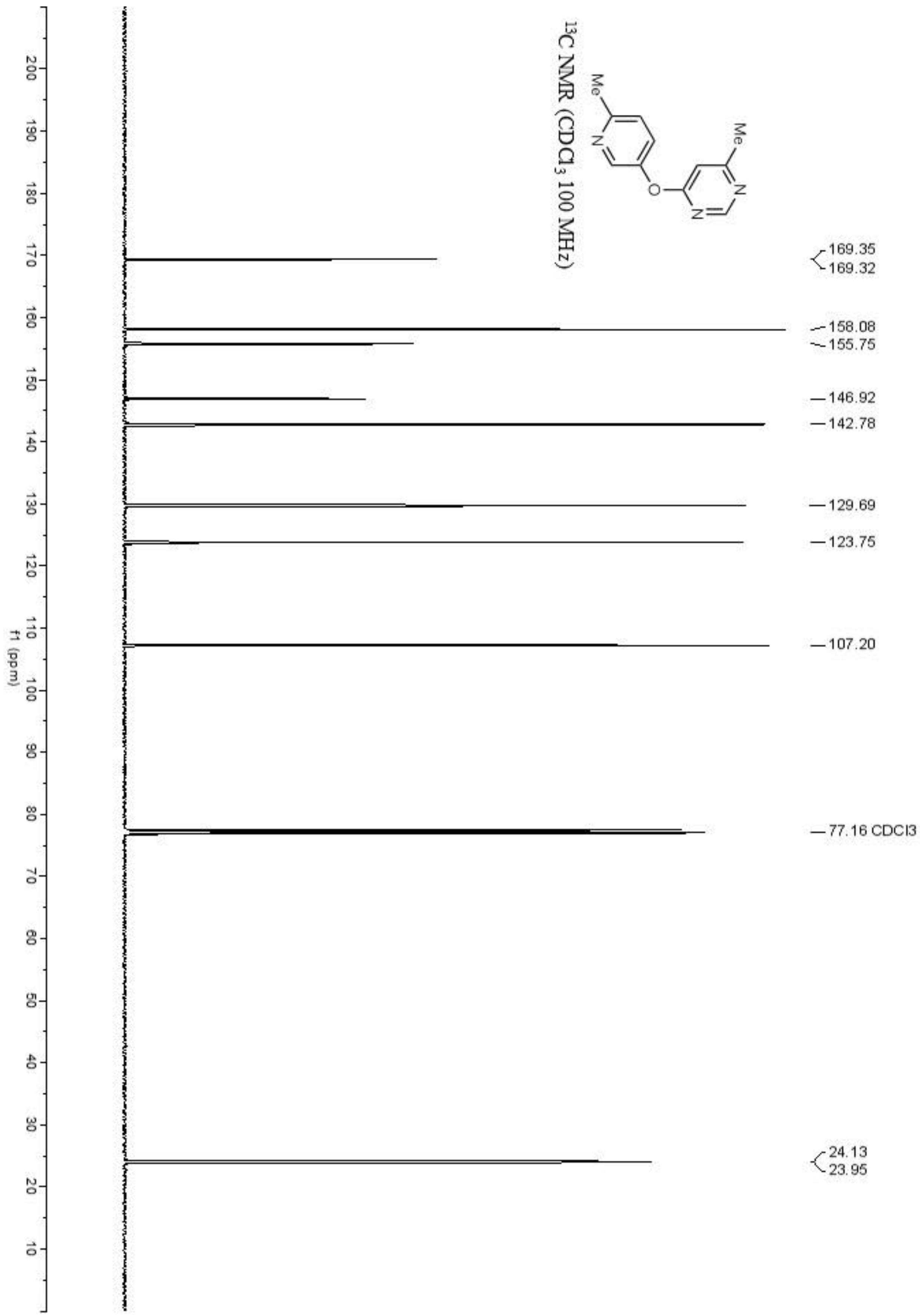
13C NMR (CDCl3 100 MHz)
Me
N
O
Me
N
N
169.35
169.32
158.08
155.75
146.92
142.78
129.69
123.75
107.20
77.16 CDCl3
24.13
23.95
f1 (ppm)
200
190
180
170
160
150
140
130
120
110
100
90
80
70
60
50
40
30
20
10

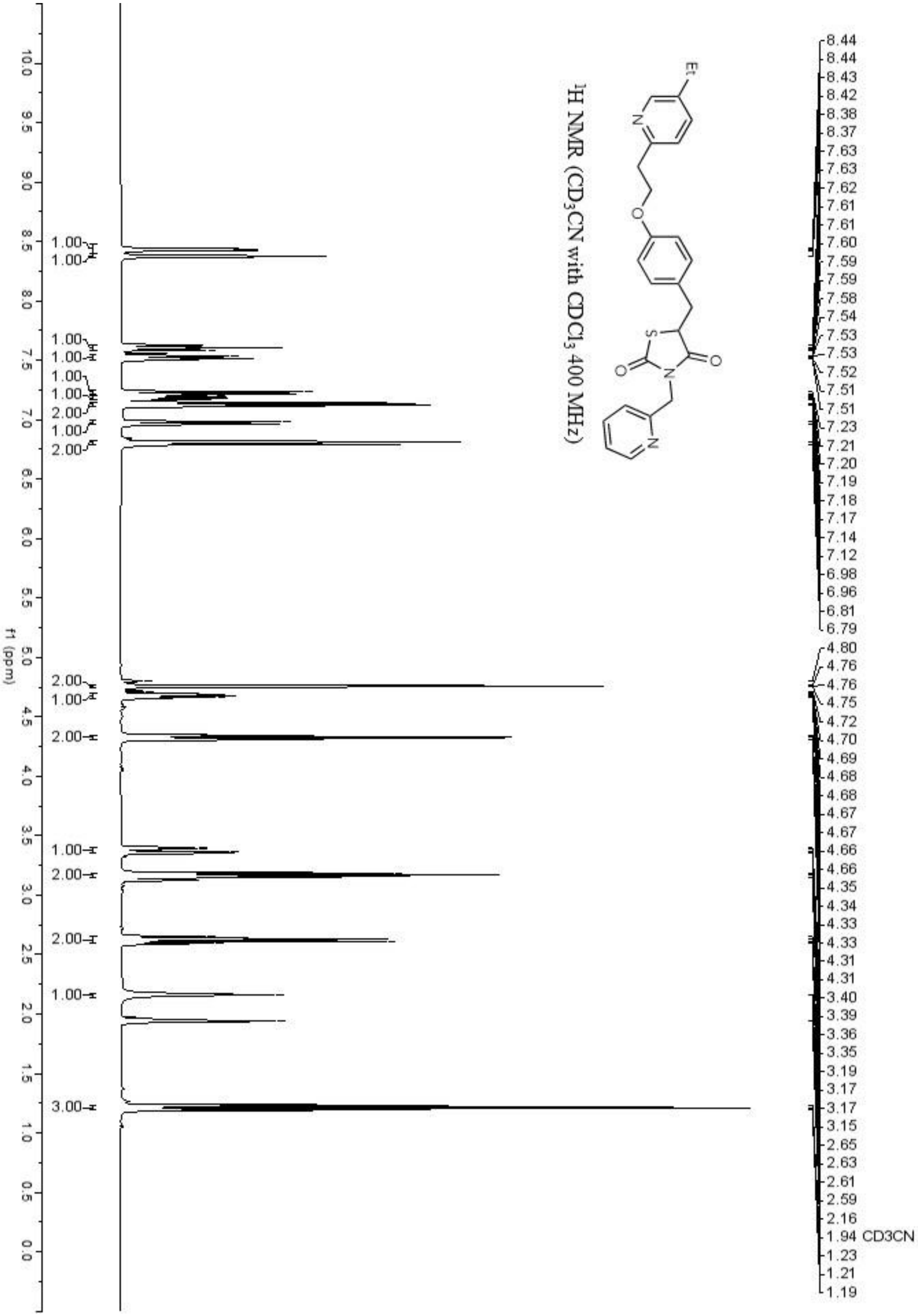
^{1}H NMR (CD$_3$CN with CDCl$_3$ 400 MHz)

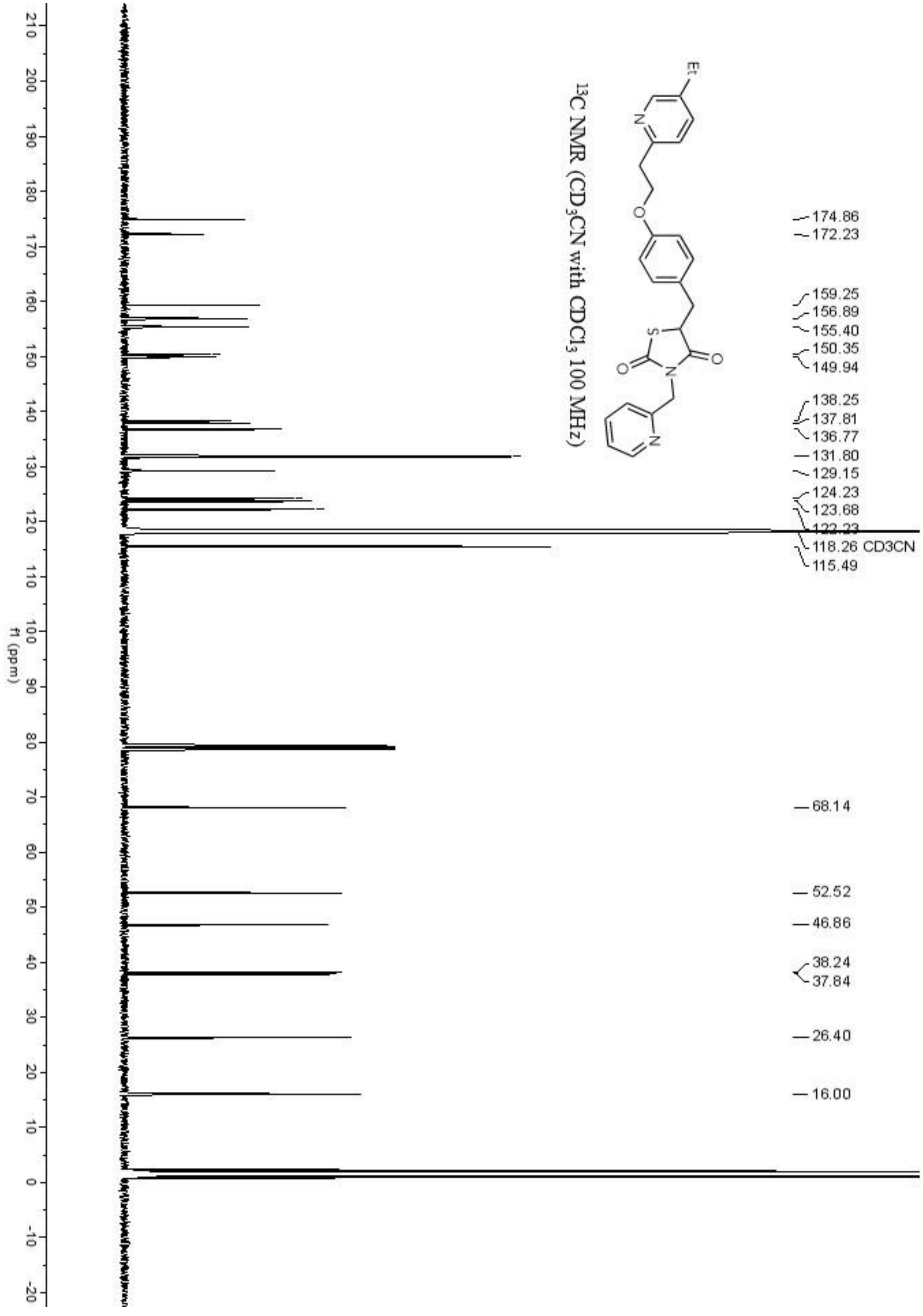
13C NMR (CD3CN with CDCl3 100 MHz)
Et
N
O
S
N
174.86
172.23
159.25
156.89
155.40
150.35
149.94
138.25
137.81
136.77
131.80
129.15
124.23
123.68
122.23
118.26 CD3CN
115.49
68.14
52.52
46.86
38.24
37.84
26.40
16.00
f1 (ppm)

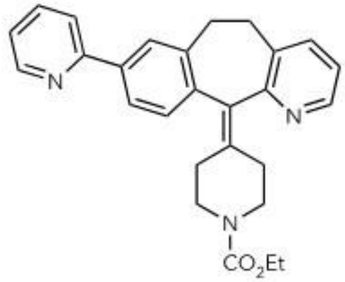

^1H NMR ($CDCl_3$ 400 MHz)
6

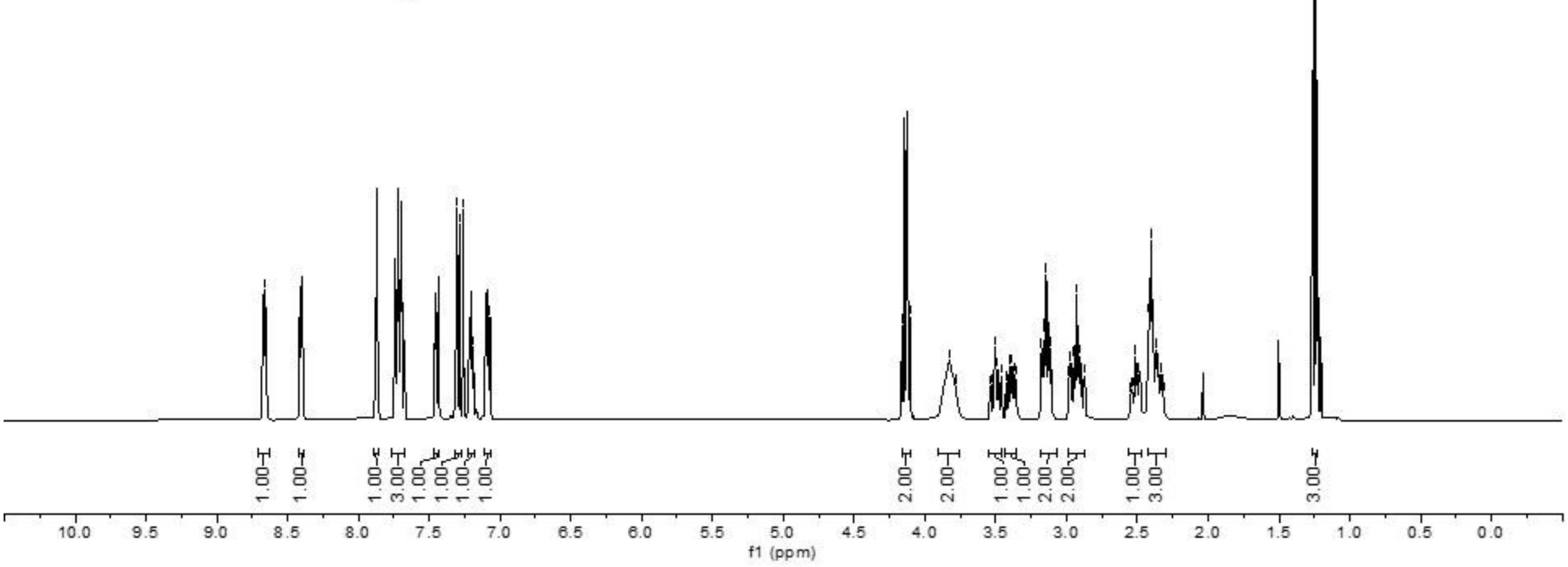

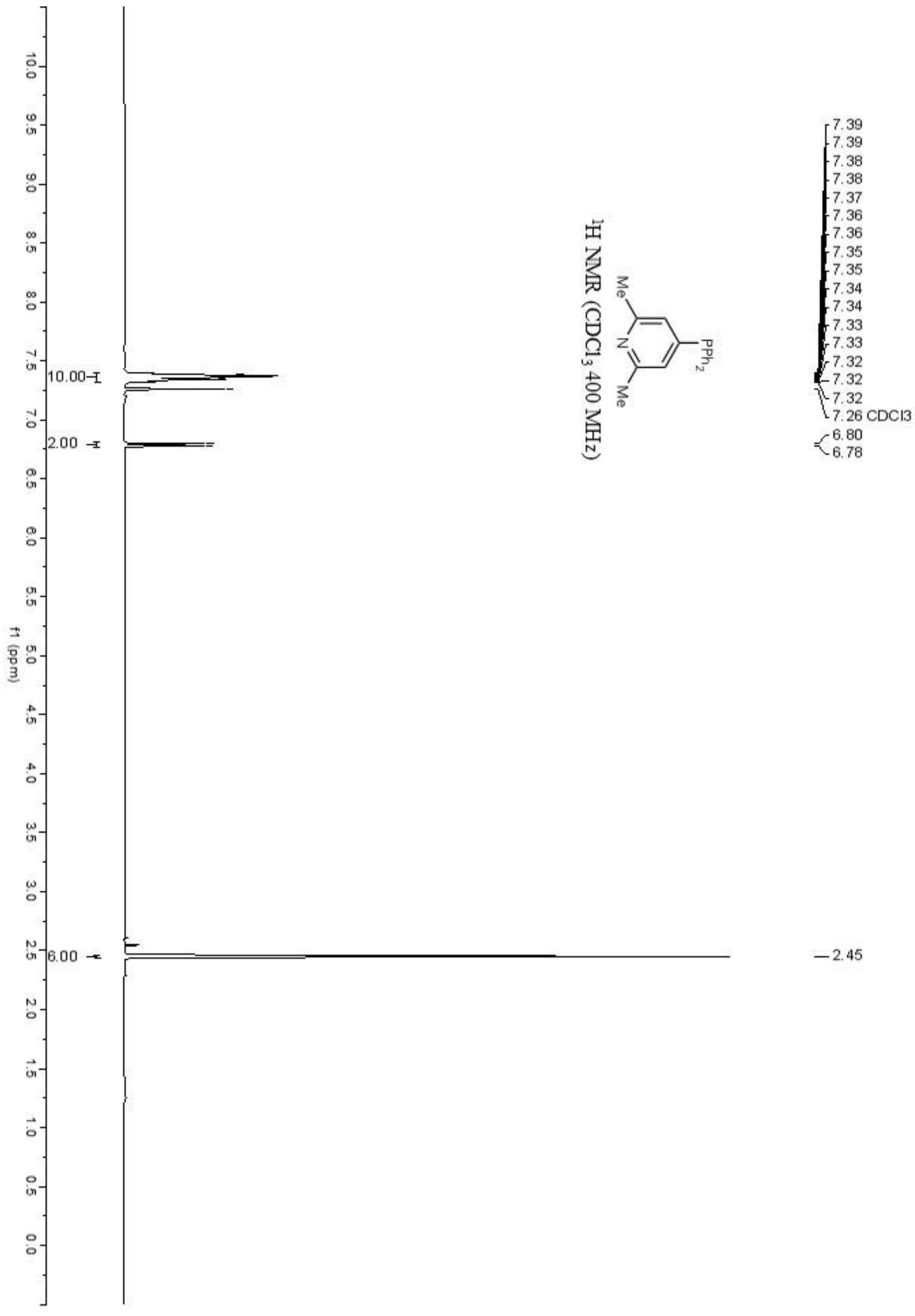

¹H NMR (CDCl₃ 400 MHz)
PPh₂
Me
N
Me
7.39
7.39
7.38
7.38
7.37
7.36
7.36
7.35
7.35
7.34
7.34
7.33
7.33
7.32
7.32
7.32
7.26 CDCl3
6.80
6.78
2.45
10.00
2.00
6.00
f1 (ppm)

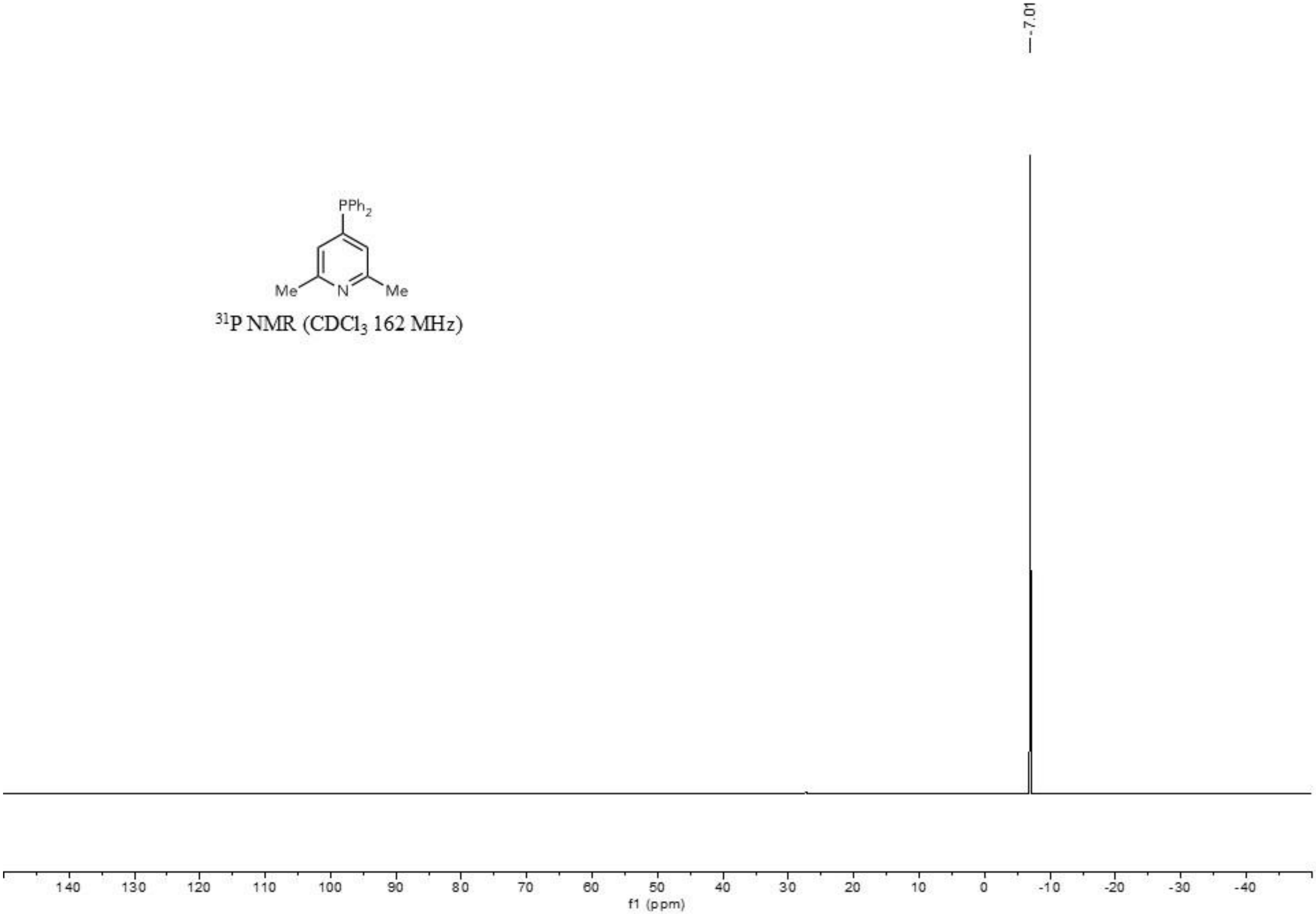

^{31}P NMR ($CDCl_3$ 162 MHz)

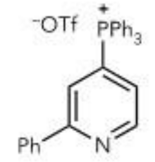

^{1}H NMR ($CDCl_3$ 400 MHz)

1a

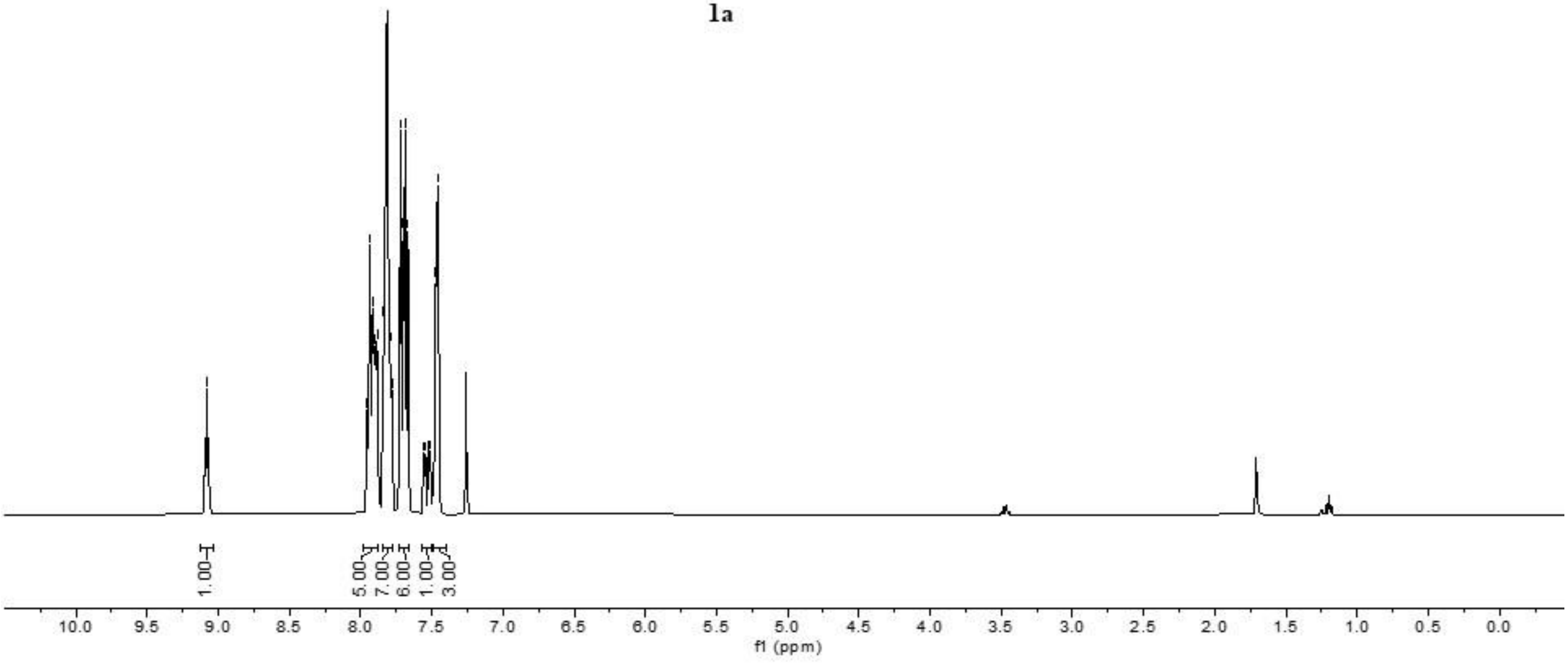

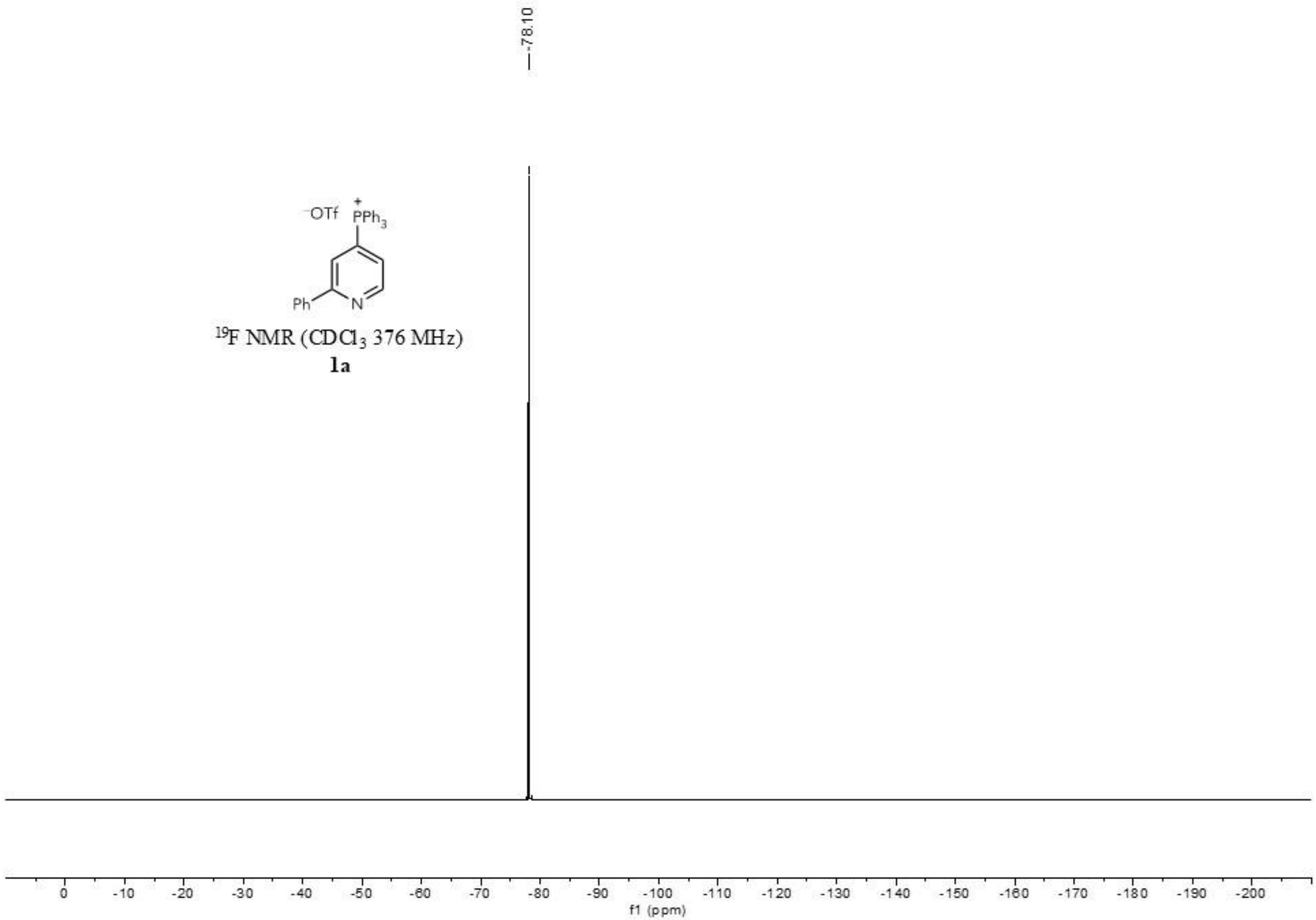
-78.10
⁻OTf
$\overset{+}{P}Ph_3$
Ph
N
^{19}F NMR ($CDCl_3$ 376 MHz)
1a
0 -10 -20 -30 -40 -50 -60 -70 -80 -90 -100 -110 -120 -130 -140 -150 -160 -170 -180 -190 -200
f1 (ppm)

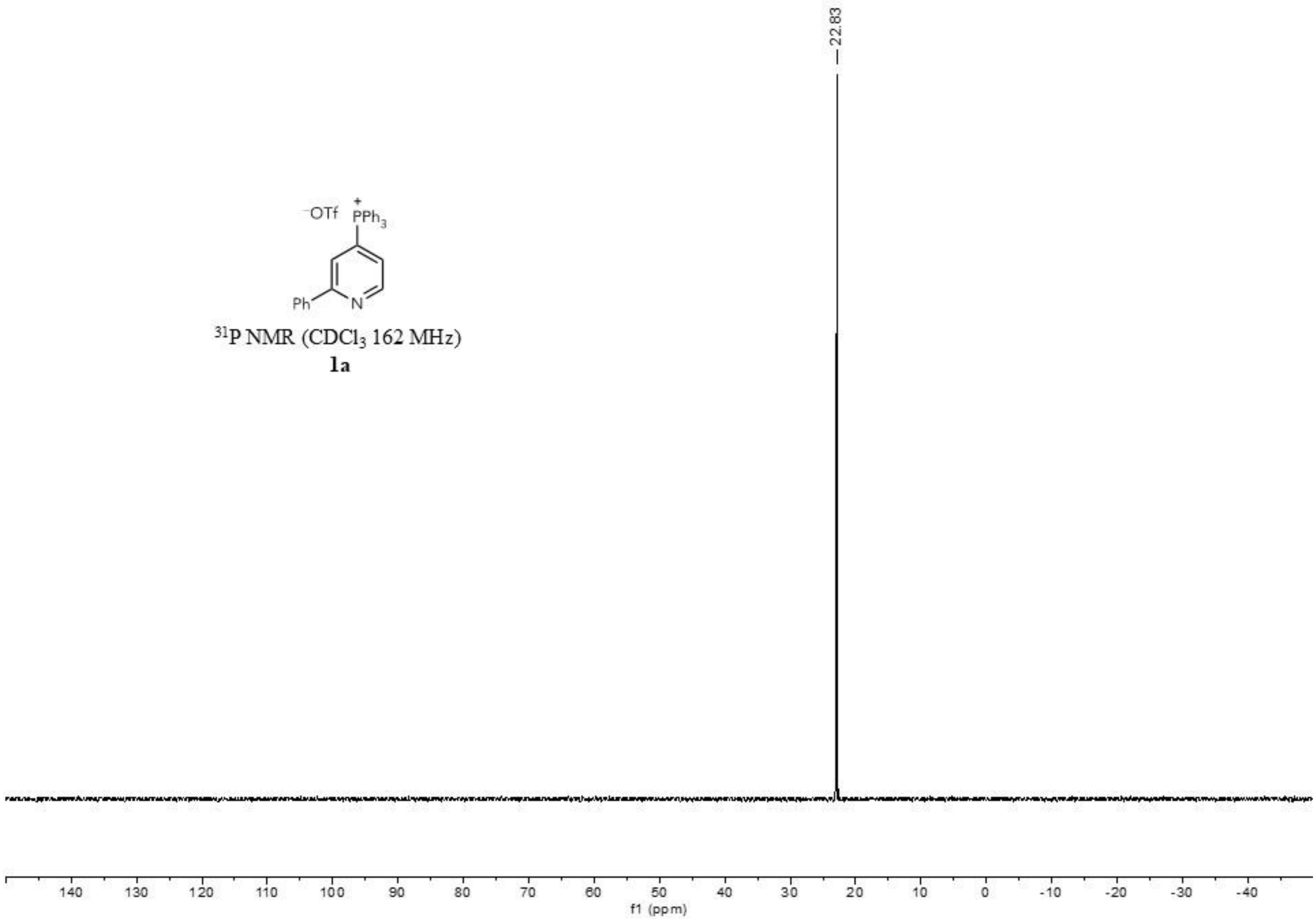

-OTf
+PPh3
Ph
N
31P NMR (CDCl3 162 MHz)
1a
22.83
140 130 120 110 100 90 80 70 60 50 40 30 20 10 0 -10 -20 -30 -40
f1 (ppm)

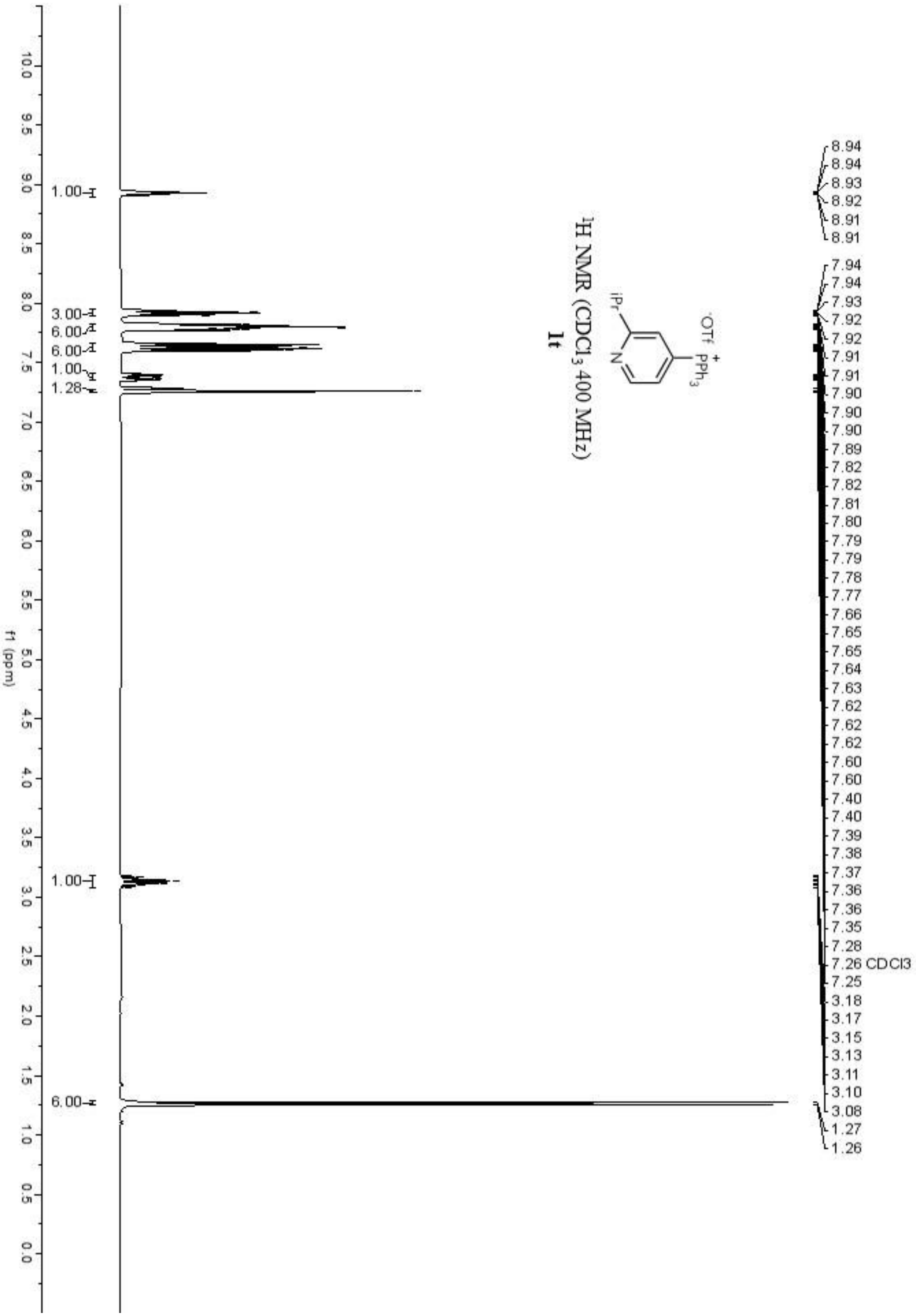
1H NMR (CDCl3 400 MHz)
1t
-OTf
+PPh3
iPr
N
8.94
8.94
8.93
8.92
8.91
8.91
7.94
7.94
7.93
7.92
7.92
7.91
7.91
7.90
7.90
7.90
7.89
7.82
7.82
7.81
7.80
7.79
7.79
7.78
7.77
7.66
7.65
7.65
7.64
7.63
7.62
7.62
7.62
7.60
7.60
7.40
7.40
7.39
7.38
7.37
7.36
7.36
7.35
7.28
7.26 CDCl3
7.25
3.18
3.17
3.15
3.13
3.11
3.10
3.08
1.27
1.26
1.00
3.00
6.00
6.00
1.00
1.28
1.00
6.00
f1 (ppm)

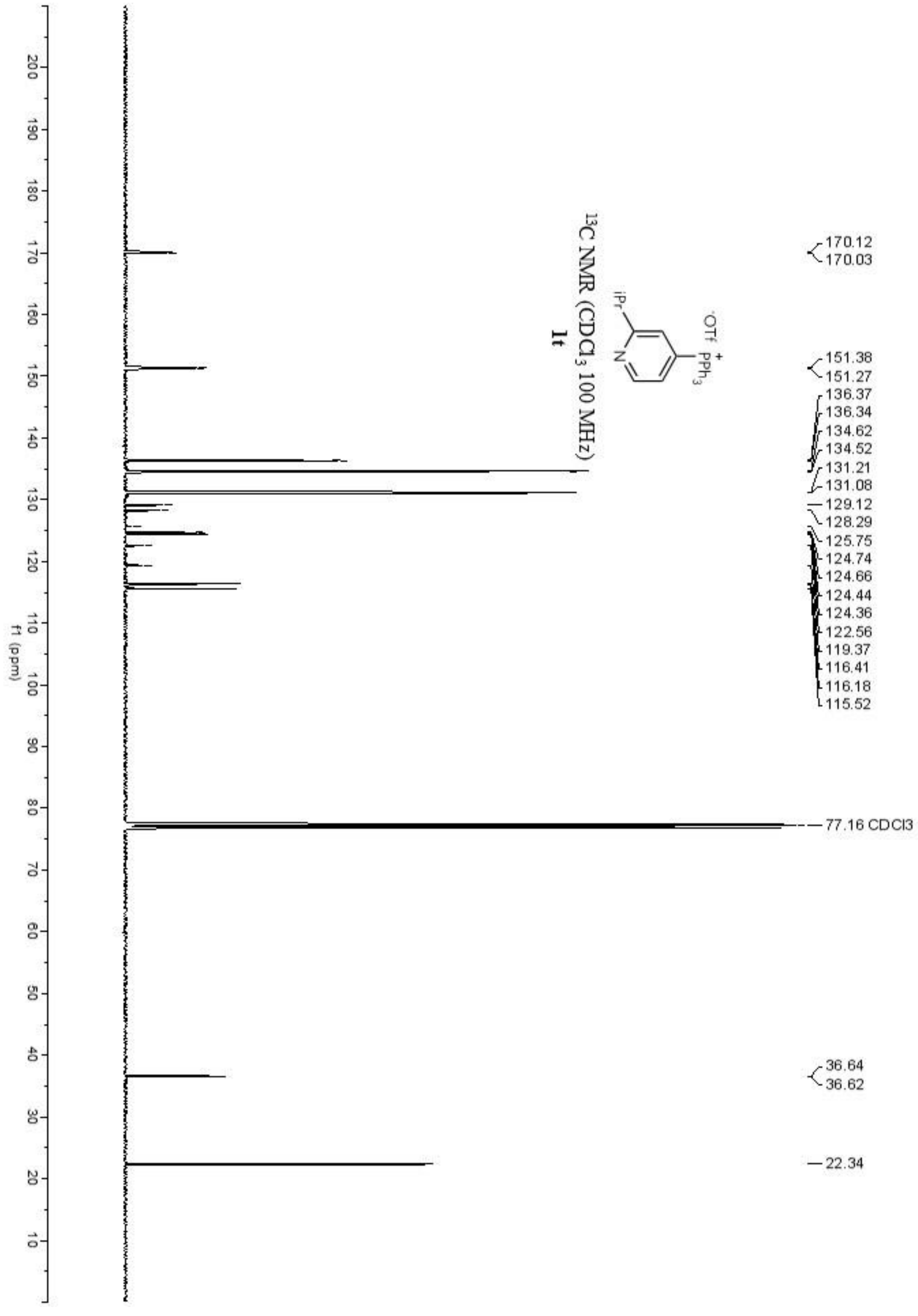

13C NMR (CDCl3 100 MHz)
1t
-OTf
+
PPh3
iPr
N
170.12
170.03
151.38
151.27
136.37
136.34
134.62
134.52
131.21
131.08
129.12
128.29
125.75
124.74
124.66
124.44
124.36
122.56
119.37
116.41
116.18
115.52
77.16 CDCl3
36.64
36.62
22.34
f1 (ppm)
200
190
180
170
160
150
140
130
120
110
100
90
80
70
60
50
40
30
20
10

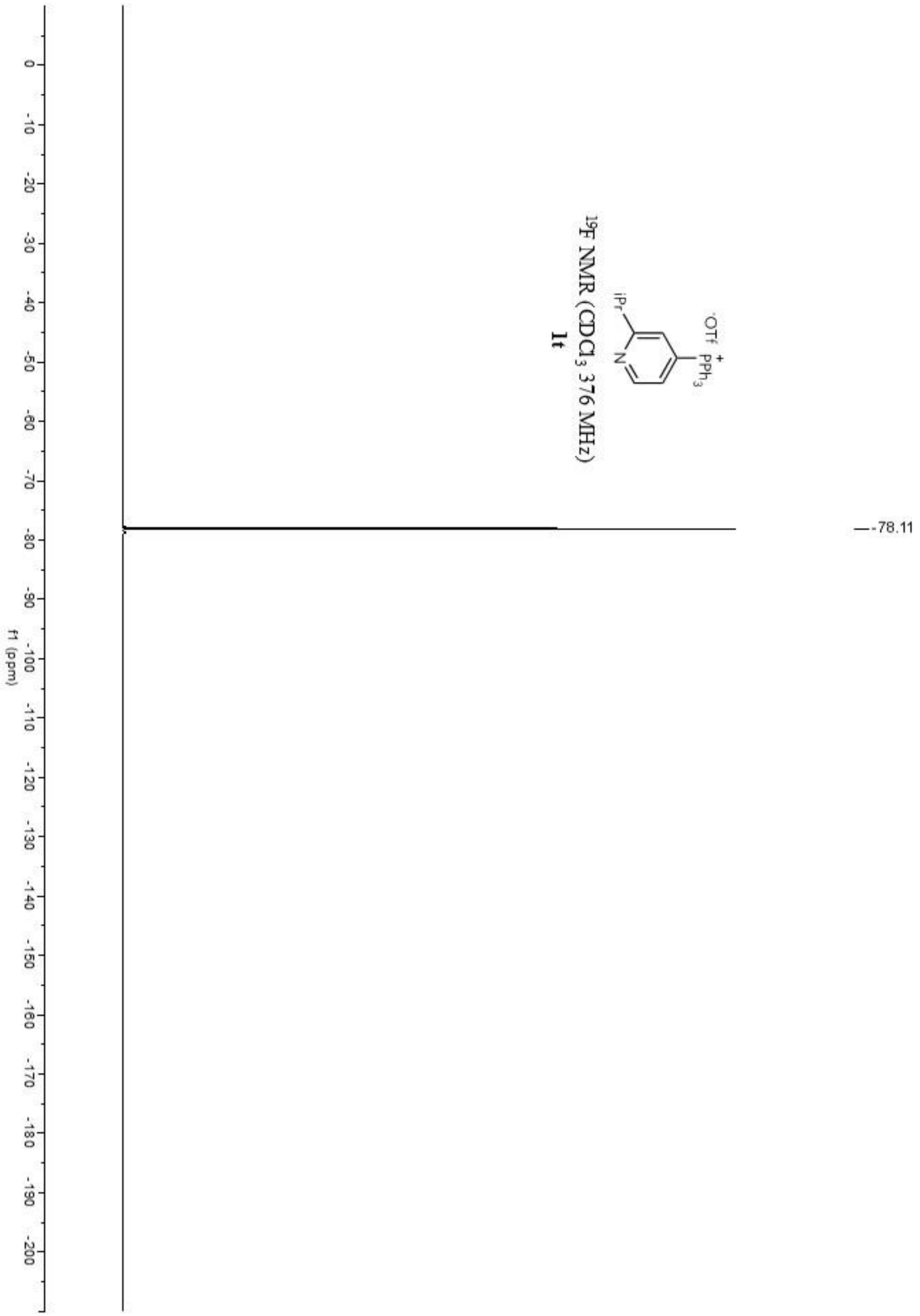
19F NMR (CDCl3 376 MHz)
1t
-78.11
f1 (ppm)

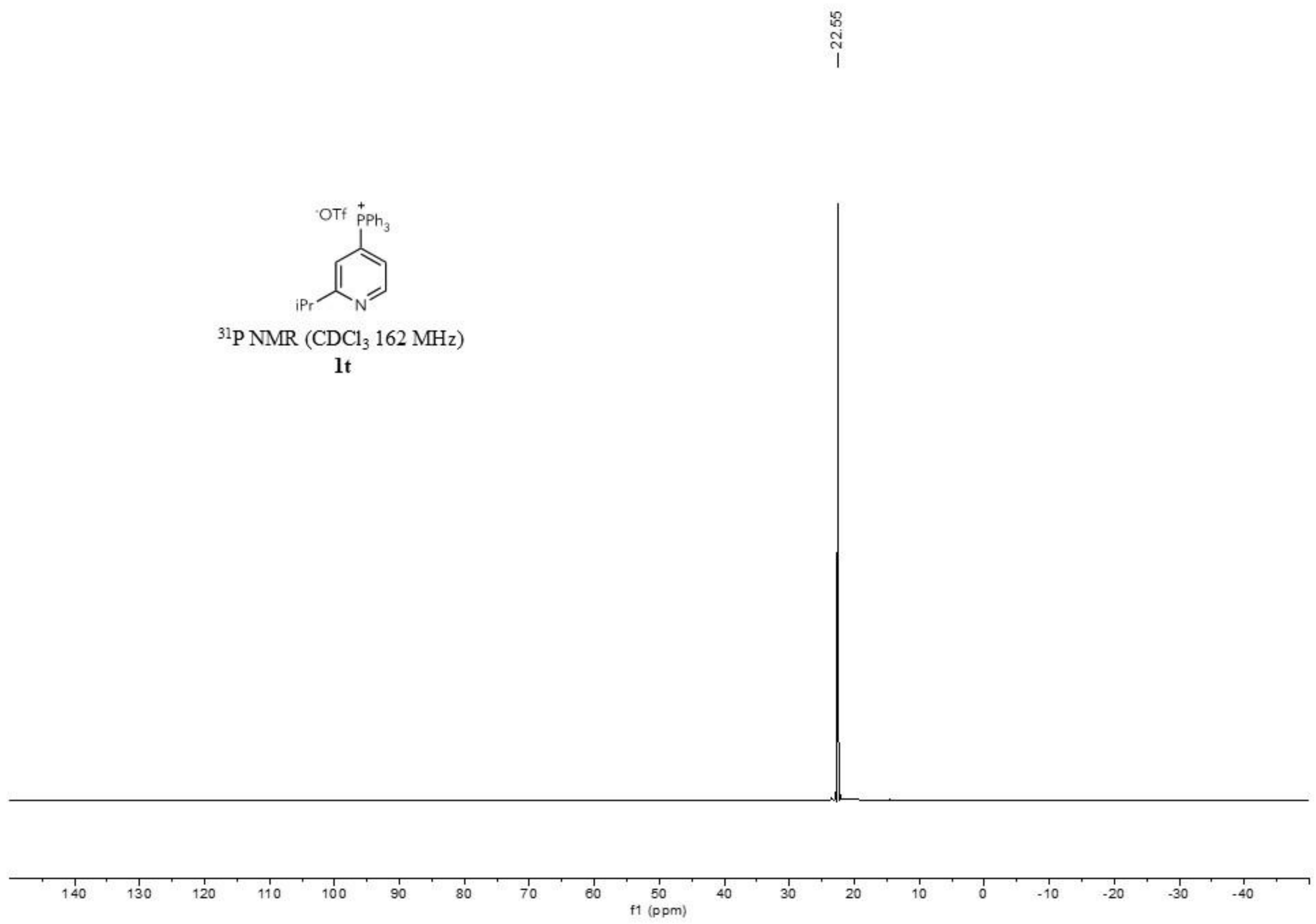

^{31}P NMR (CDCl$_3$ 162 MHz)

1t

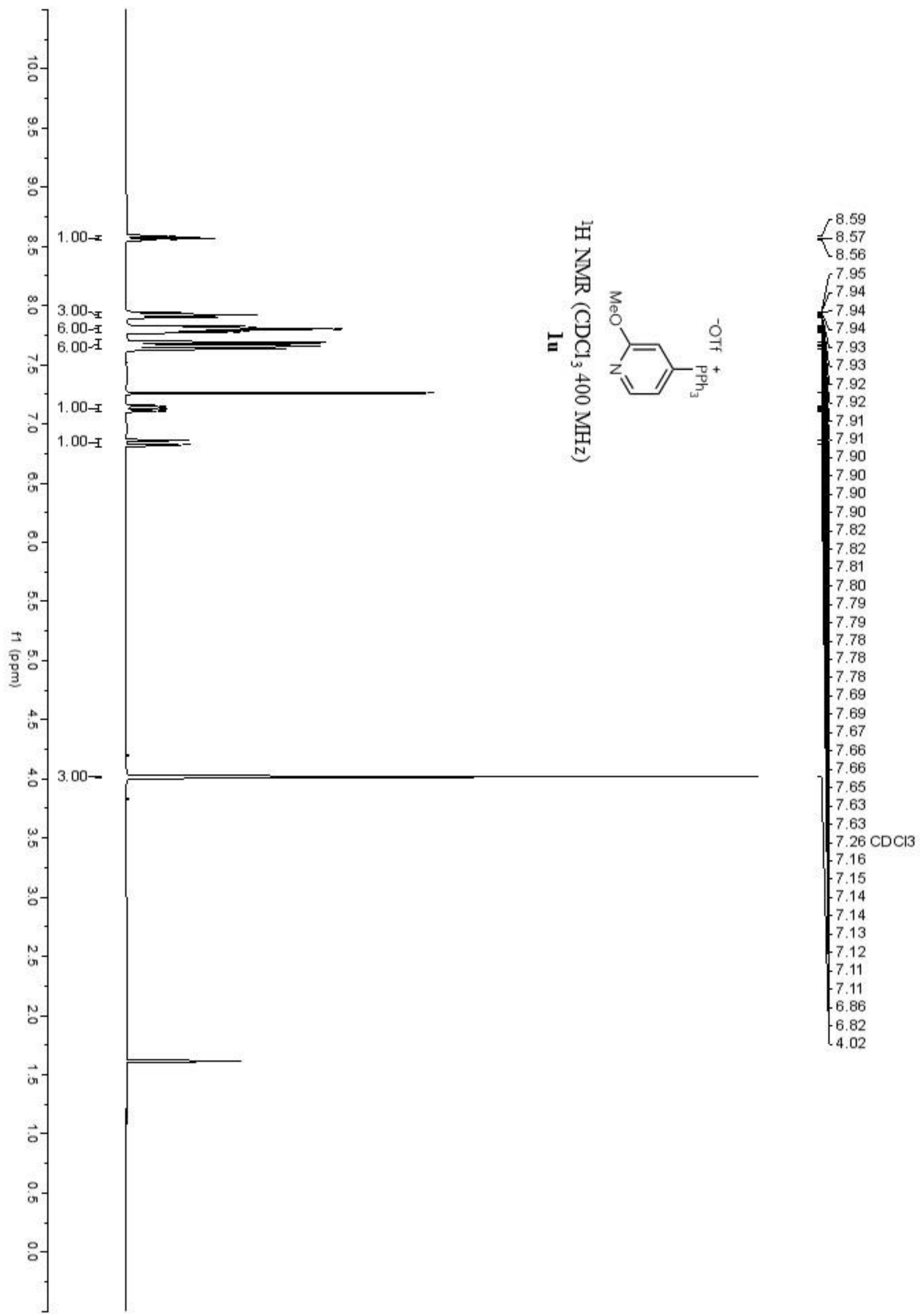
⁻OTf
+
PPh3
MeO
N
¹H NMR (CDCl3 400 MHz)
1u
8.59
8.57
8.56
7.95
7.94
7.94
7.94
7.93
7.93
7.92
7.92
7.91
7.91
7.90
7.90
7.90
7.90
7.82
7.82
7.81
7.80
7.79
7.79
7.78
7.78
7.78
7.69
7.69
7.67
7.66
7.66
7.65
7.63
7.63
7.26 CDCl3
7.16
7.15
7.14
7.14
7.13
7.12
7.11
7.11
6.86
6.82
4.02
1.00
3.00
6.00
6.00
1.00
1.00
3.00
10.0
9.5
9.0
8.5
8.0
7.5
7.0
6.5
6.0
5.5
5.0
4.5
4.0
3.5
3.0
2.5
2.0
1.5
1.0
0.5
0.0
f1 (ppm)

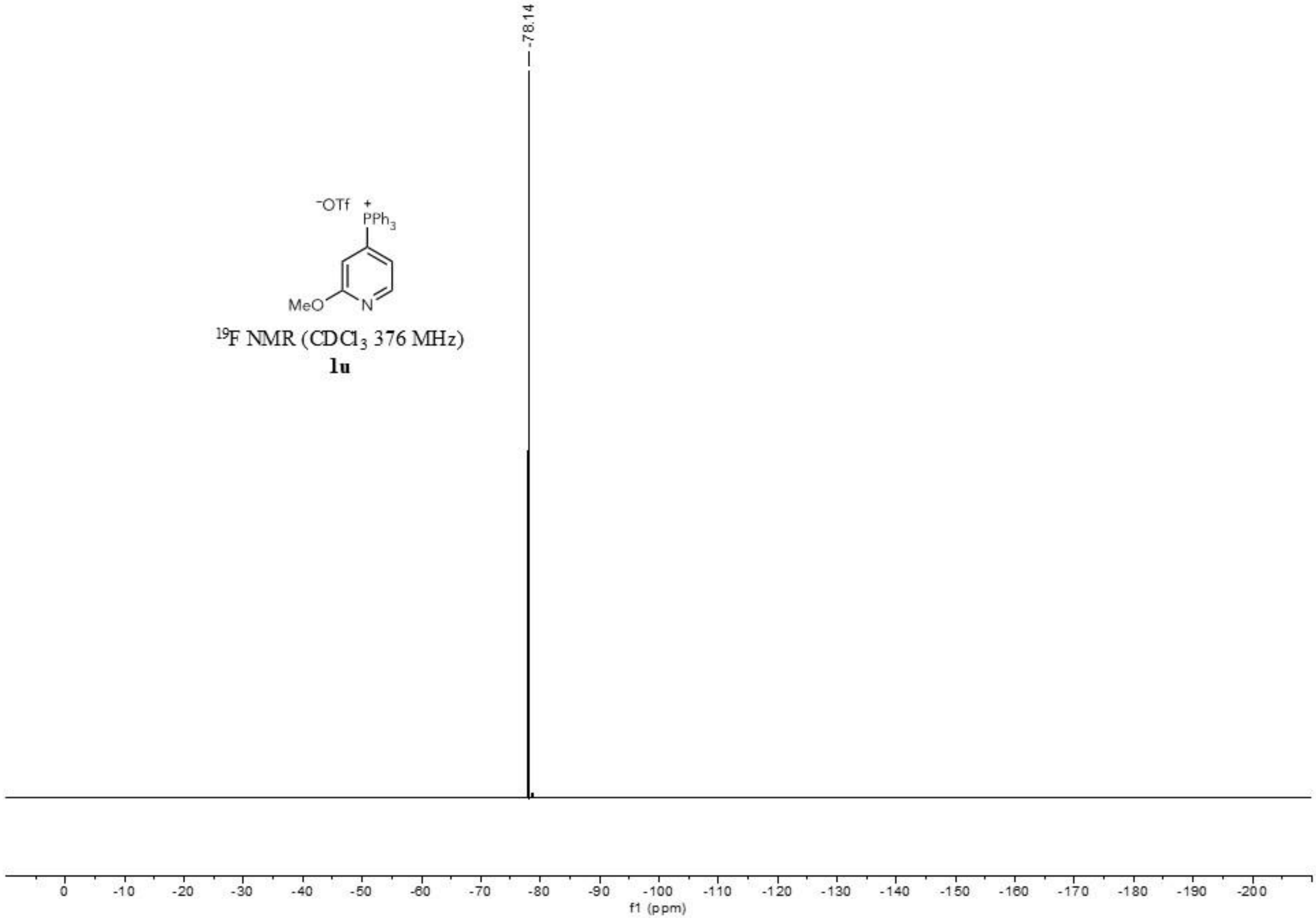
-78.14
-OTf
+
PPh3
MeO
N
19F NMR (CDCl3 376 MHz)
1u
0
-10
-20
-30
-40
-50
-60
-70
-80
-90
-100
-110
-120
-130
-140
-150
-160
-170
-180
-190
-200
f1 (ppm)

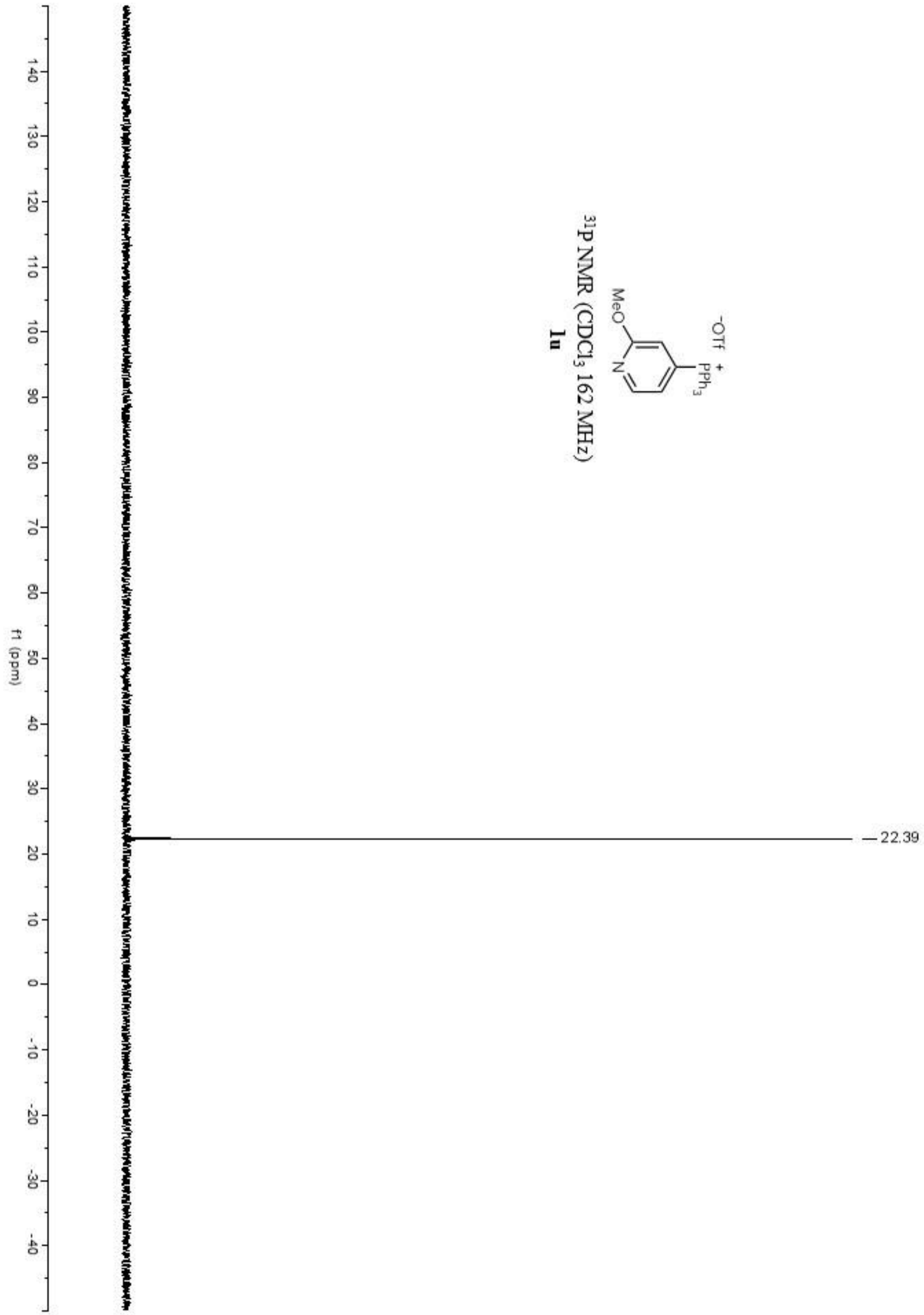

-OTf +
PPh3
MeO
N
31P NMR (CDCl3 162 MHz)
1u
22.39
f1 (ppm)

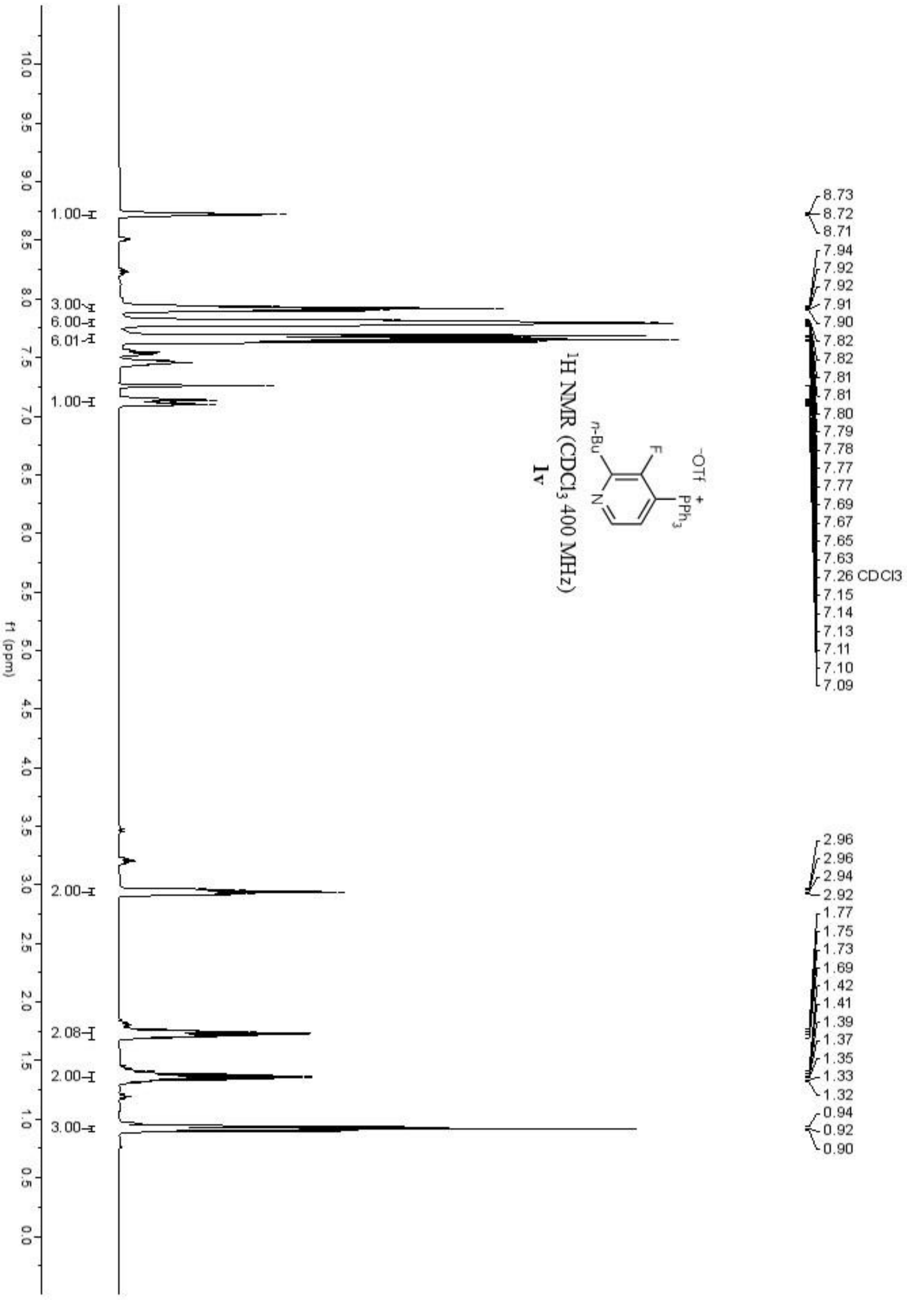

^{1}H NMR (CDCl$_3$ 400 MHz)
Iv
n-Bu
F
N
-OTf
+
PPh3
8.73
8.72
8.71
7.94
7.92
7.92
7.91
7.90
7.82
7.82
7.81
7.81
7.80
7.79
7.78
7.77
7.77
7.69
7.67
7.65
7.63
7.26 CDCl3
7.15
7.14
7.13
7.11
7.10
7.09
2.96
2.96
2.94
2.92
1.77
1.75
1.73
1.69
1.42
1.41
1.39
1.37
1.35
1.33
1.32
0.94
0.92
0.90
1.00
3.00
6.00
6.01
1.00
2.00
2.08
2.00
3.00
f1 (ppm)

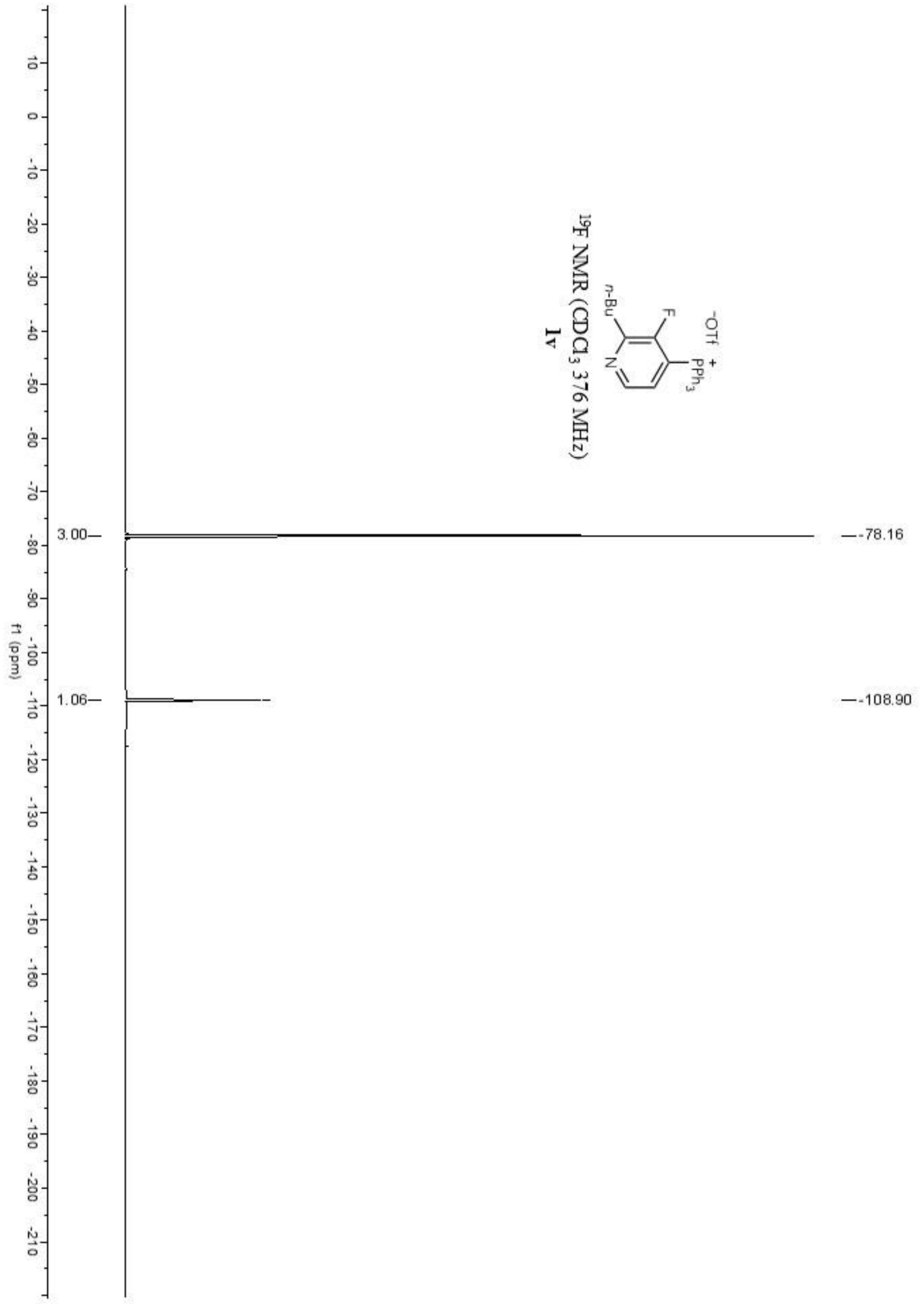

19F NMR (CDCl3 376 MHz)
1v
-OTf
+
PPh3
F
n-Bu
N
-78.16
-108.90
3.00
1.06
f1 (ppm)

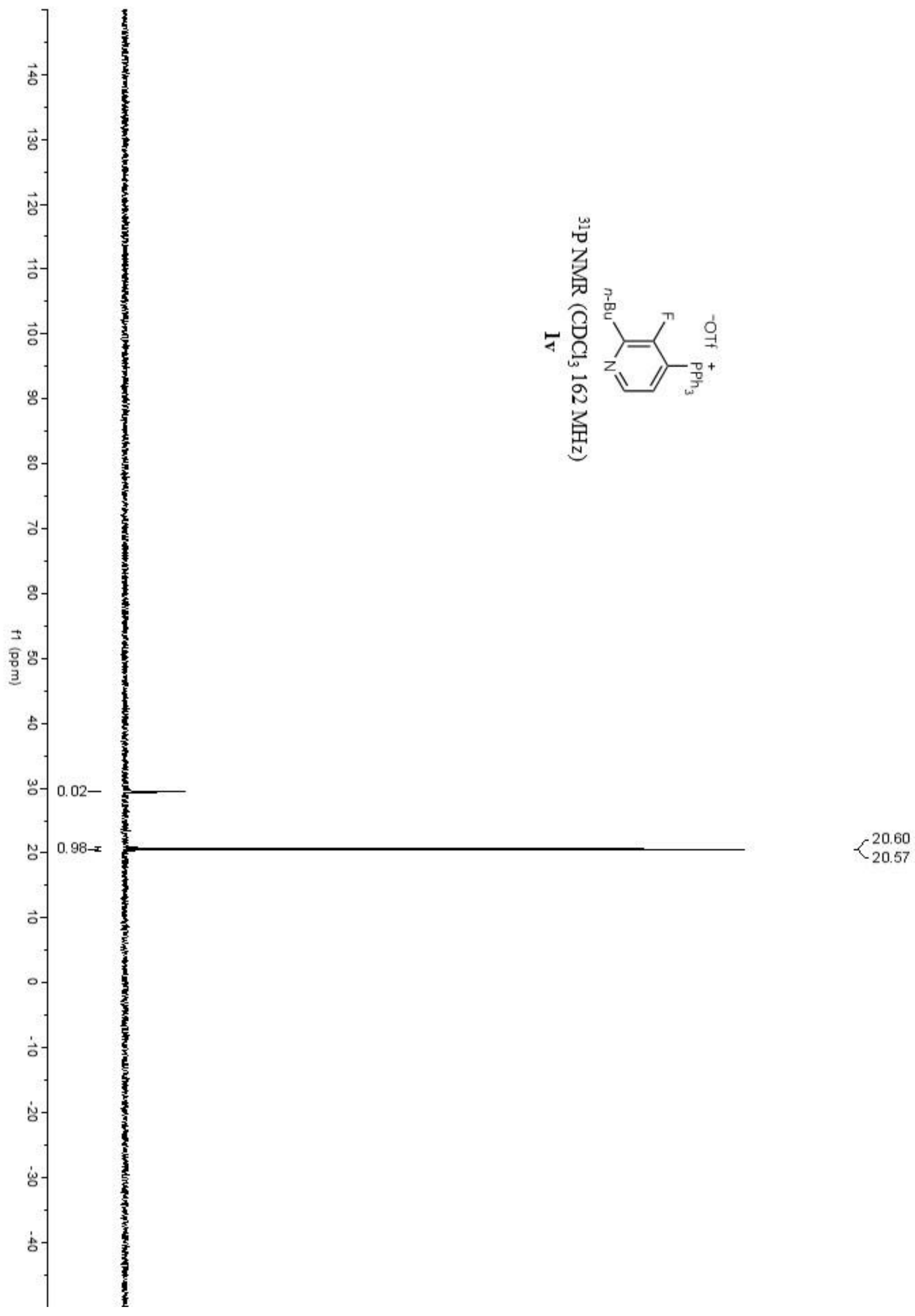

-OTf
+
PPh3
F
n-Bu
N
31P NMR (CDCl3 162 MHz)
1v
20.60
20.57
0.02
0.98
f1 (ppm)
140
130
120
110
100
90
80
70
60
50
40
30
20
10
0
-10
-20
-30
-40

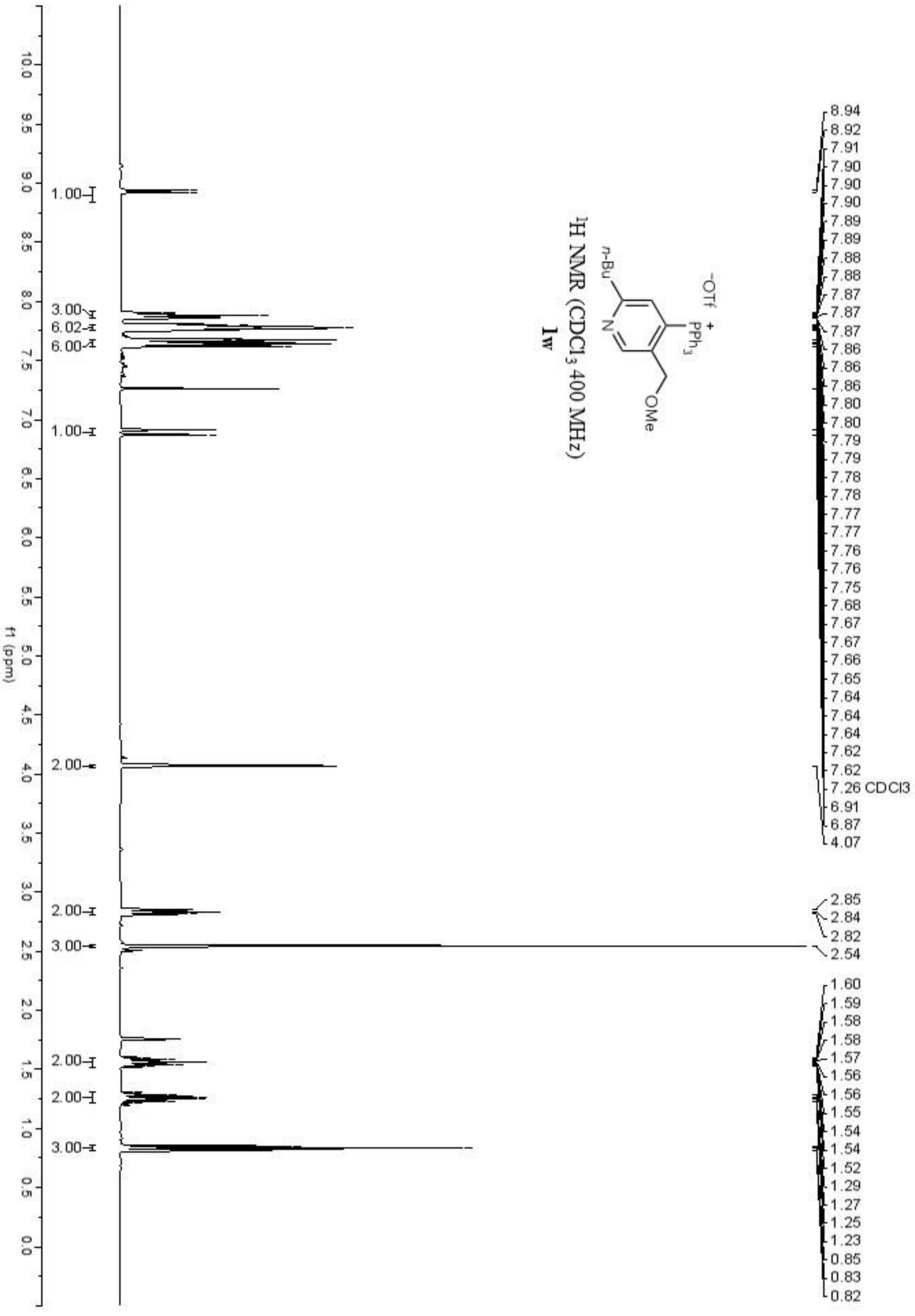

-OTf
+
PPh3
n-Bu
N
OMe
1H NMR (CDCl3 400 MHz)
1w
f1 (ppm)
10.0
9.5
9.0
8.5
8.0
7.5
7.0
6.5
6.0
5.5
5.0
4.5
4.0
3.5
3.0
2.5
2.0
1.5
1.0
0.5
0.0
1.00
3.00
6.02
6.00
1.00
2.00
2.00
3.00
2.00
2.00
3.00
8.94
8.92
7.91
7.90
7.90
7.90
7.89
7.89
7.88
7.88
7.87
7.87
7.87
7.86
7.86
7.86
7.80
7.80
7.79
7.79
7.78
7.78
7.77
7.77
7.76
7.76
7.75
7.68
7.67
7.67
7.66
7.65
7.64
7.64
7.64
7.62
7.62
7.26 CDCl3
6.91
6.87
4.07
2.85
2.84
2.82
2.54
1.60
1.59
1.58
1.58
1.57
1.56
1.56
1.55
1.54
1.54
1.52
1.29
1.27
1.25
1.23
0.85
0.83
0.82

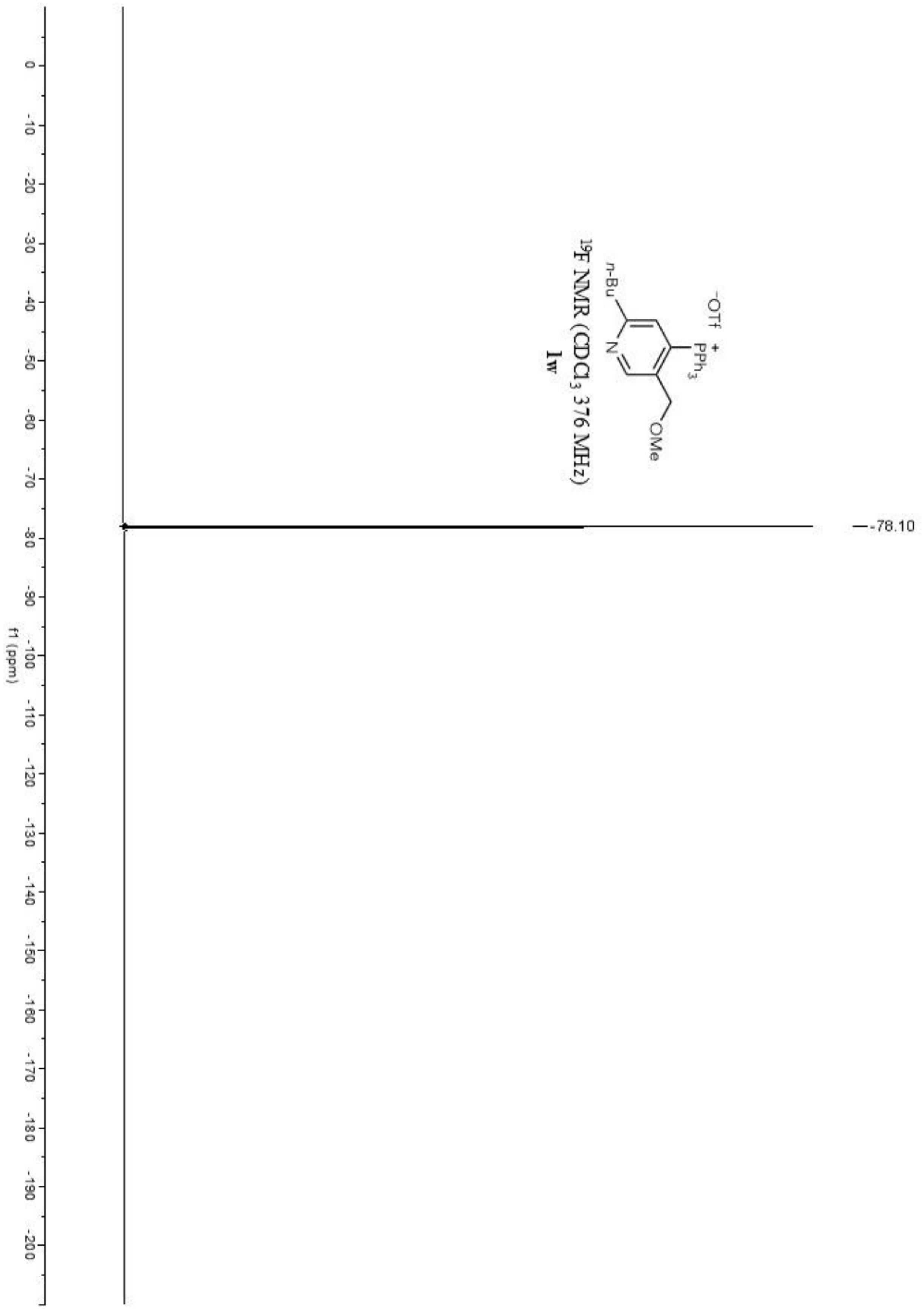
-OTf
+PPh3
n-Bu
N
OMe
19F NMR (CDCl3 376 MHz)
1w
-78.10
0
-10
-20
-30
-40
-50
-60
-70
-80
-90
-100
-110
-120
-130
-140
-150
-160
-170
-180
-190
-200
f1 (ppm)

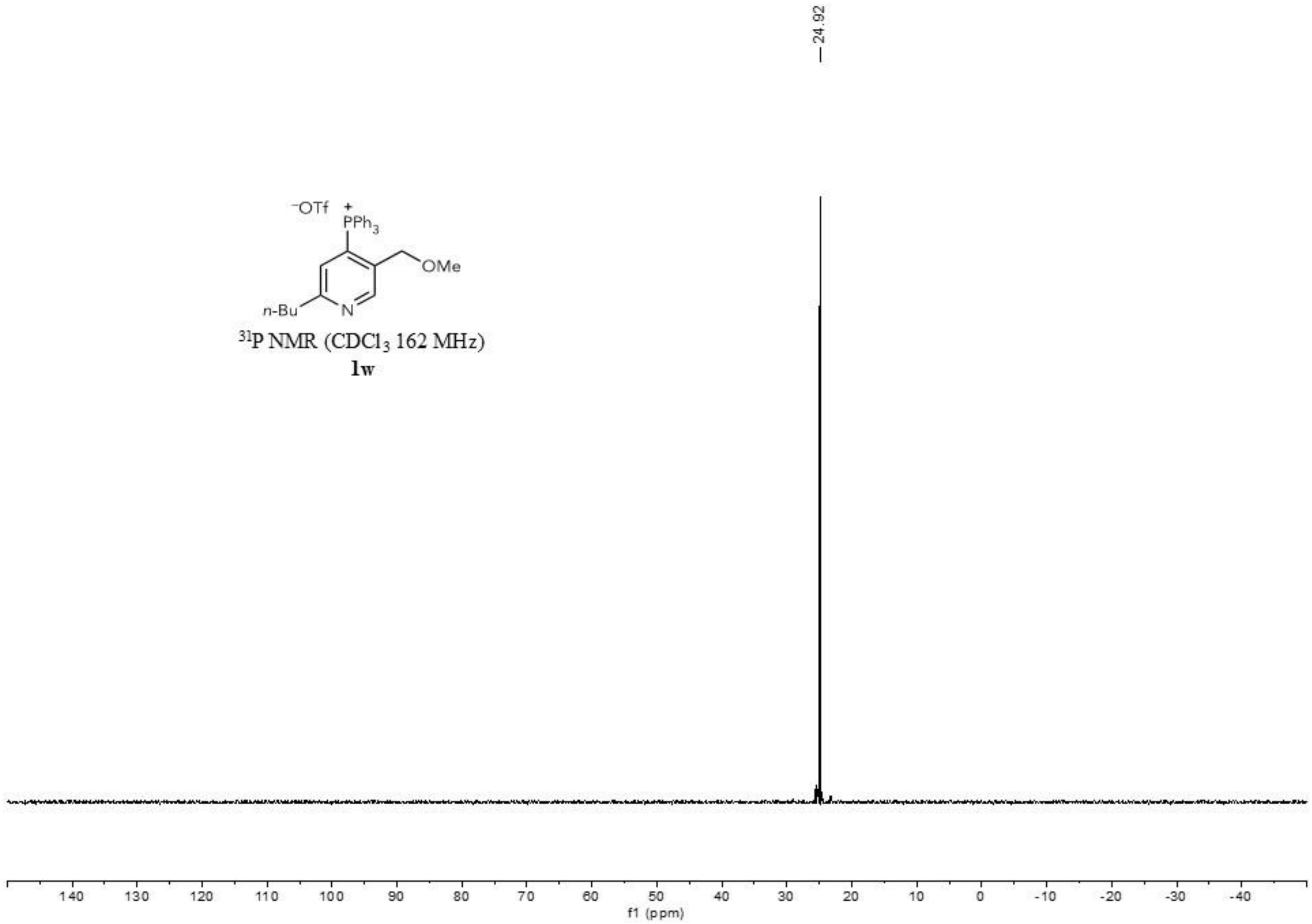

^{31}P NMR ($CDCl_3$ 162 MHz)

1w

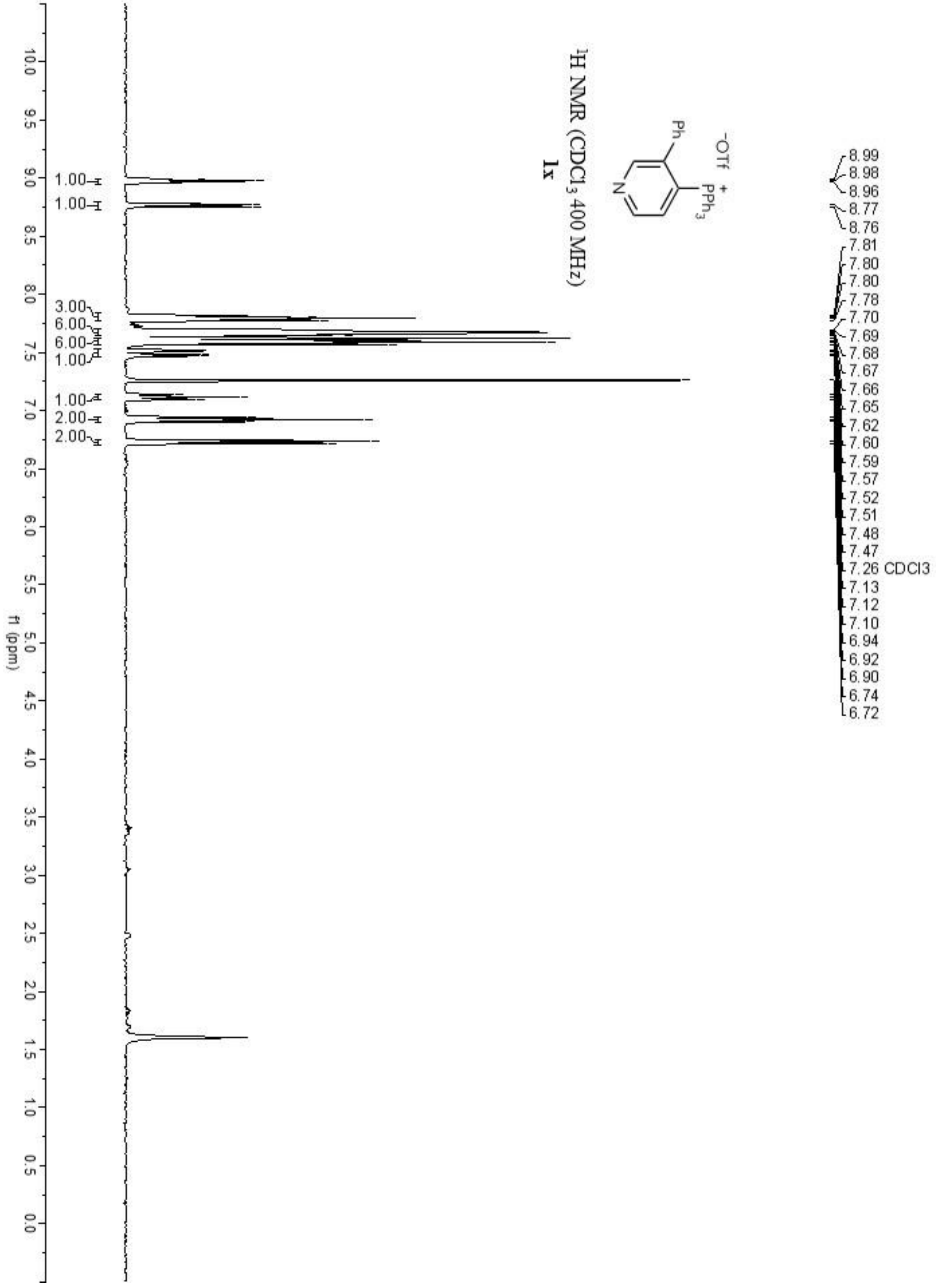

¹H NMR (CDCl3 400 MHz)
1x
-OTf
PPh3
Ph
N
8.99
8.98
8.96
8.77
8.76
7.81
7.80
7.80
7.78
7.70
7.69
7.68
7.67
7.66
7.65
7.62
7.60
7.59
7.57
7.52
7.51
7.48
7.47
7.26 CDCl3
7.13
7.12
7.10
6.94
6.92
6.90
6.74
6.72
1.00
1.00
3.00
6.00
6.00
1.00
1.00
2.00
2.00
f1 (ppm)

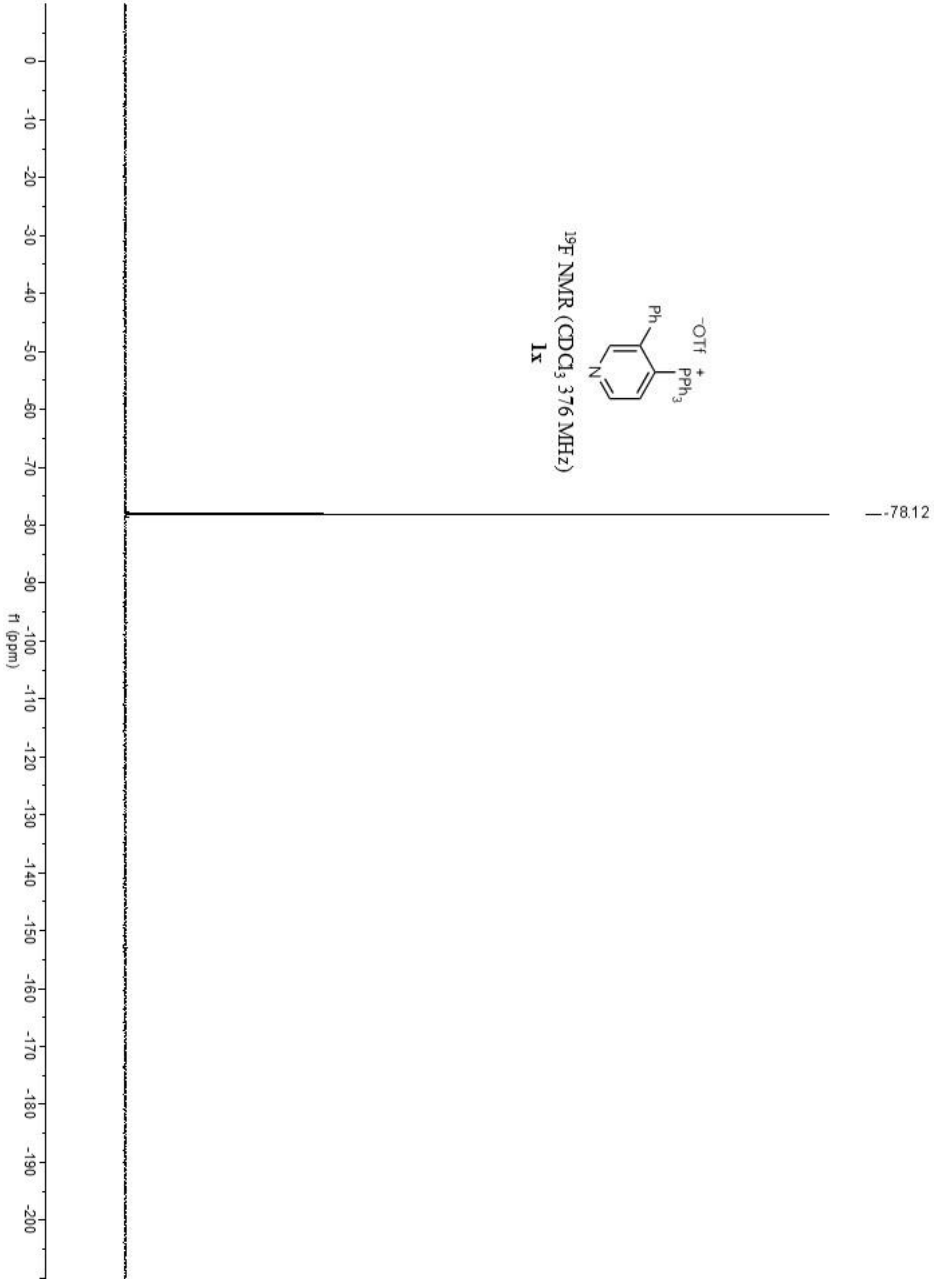

^{19}F NMR (CDCl$_3$ 376 MHz)
1x
-OTf
+
PPh3
Ph
N
-78.12
f1 (ppm)

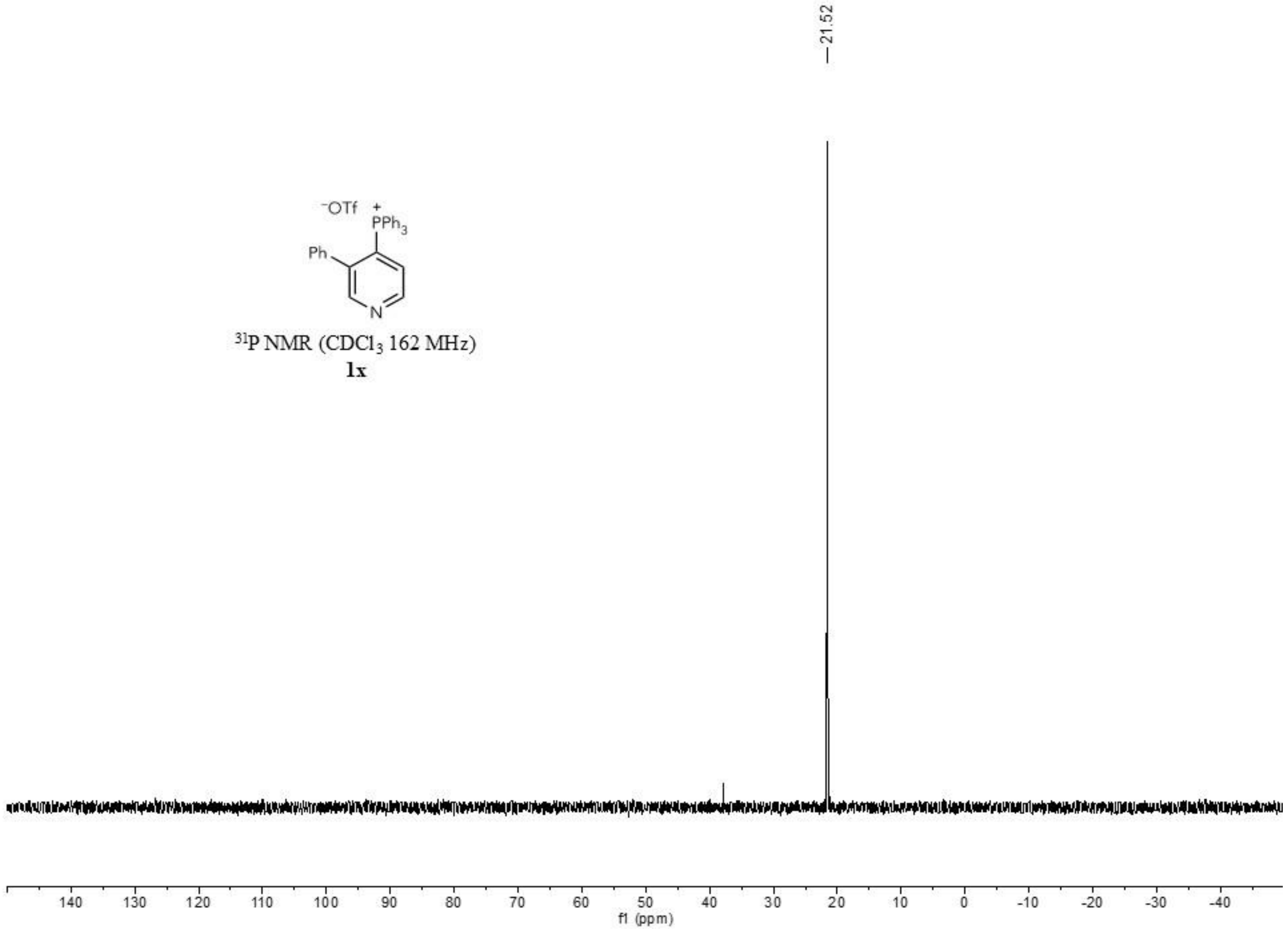
−21.52
⁻OTf +
PPh3
Ph
N
³¹P NMR (CDCl3 162 MHz)
1x
140 130 120 110 100 90 80 70 60 50 40 30 20 10 0 -10 -20 -30 -40
f1 (ppm)

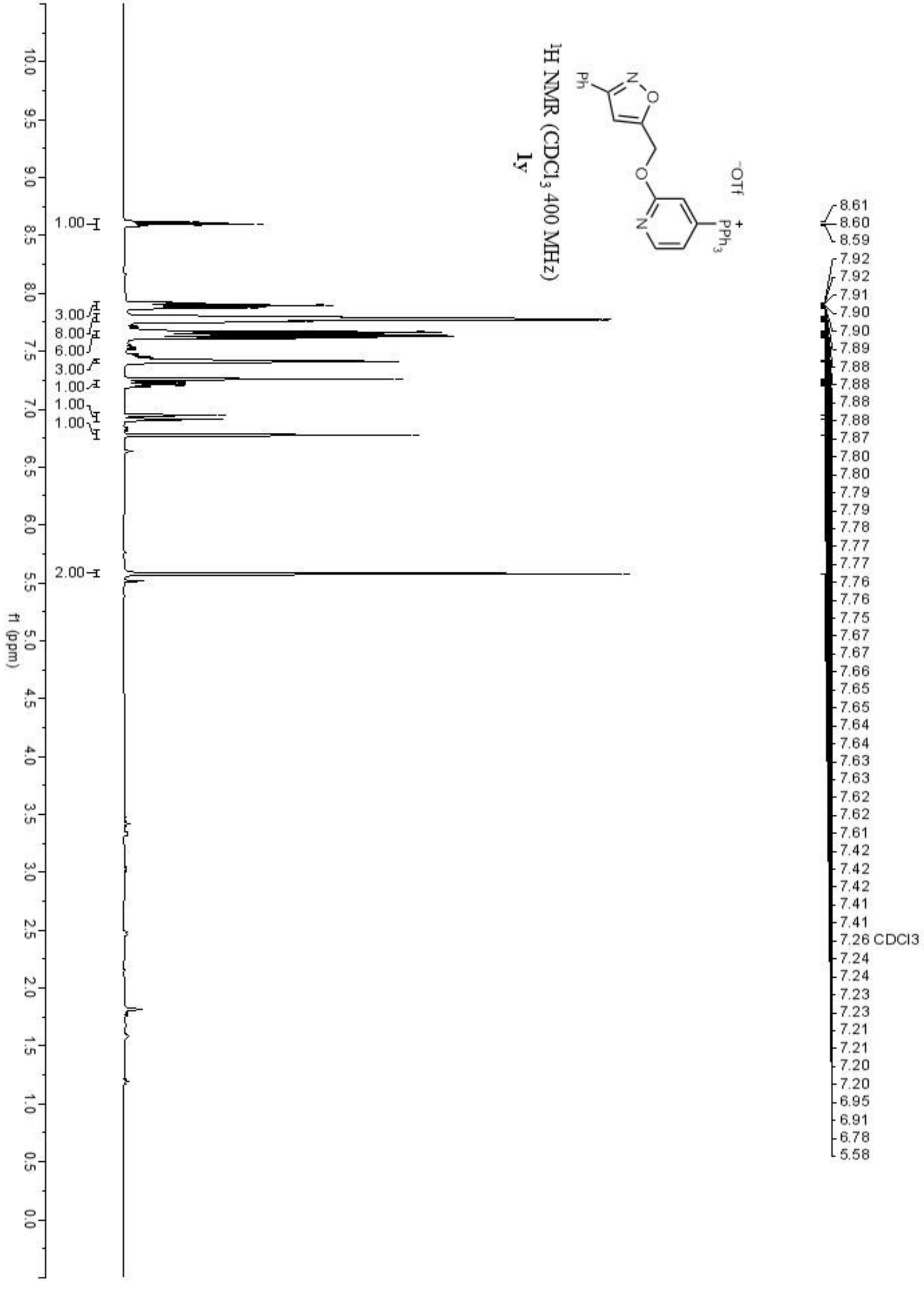

1H NMR (CDCl3 400 MHz)
1y
-OTf
+PPh3
Ph
8.61
8.60
8.59
7.92
7.92
7.91
7.90
7.90
7.89
7.88
7.88
7.88
7.88
7.87
7.80
7.80
7.79
7.79
7.78
7.77
7.77
7.76
7.76
7.75
7.67
7.67
7.66
7.65
7.65
7.64
7.64
7.63
7.63
7.62
7.62
7.61
7.42
7.42
7.42
7.41
7.41
7.26 CDCl3
7.24
7.24
7.23
7.23
7.21
7.21
7.20
7.20
6.95
6.91
6.78
5.58
1.00
3.00
8.00
6.00
3.00
1.00
1.00
1.00
2.00
f1 (ppm)

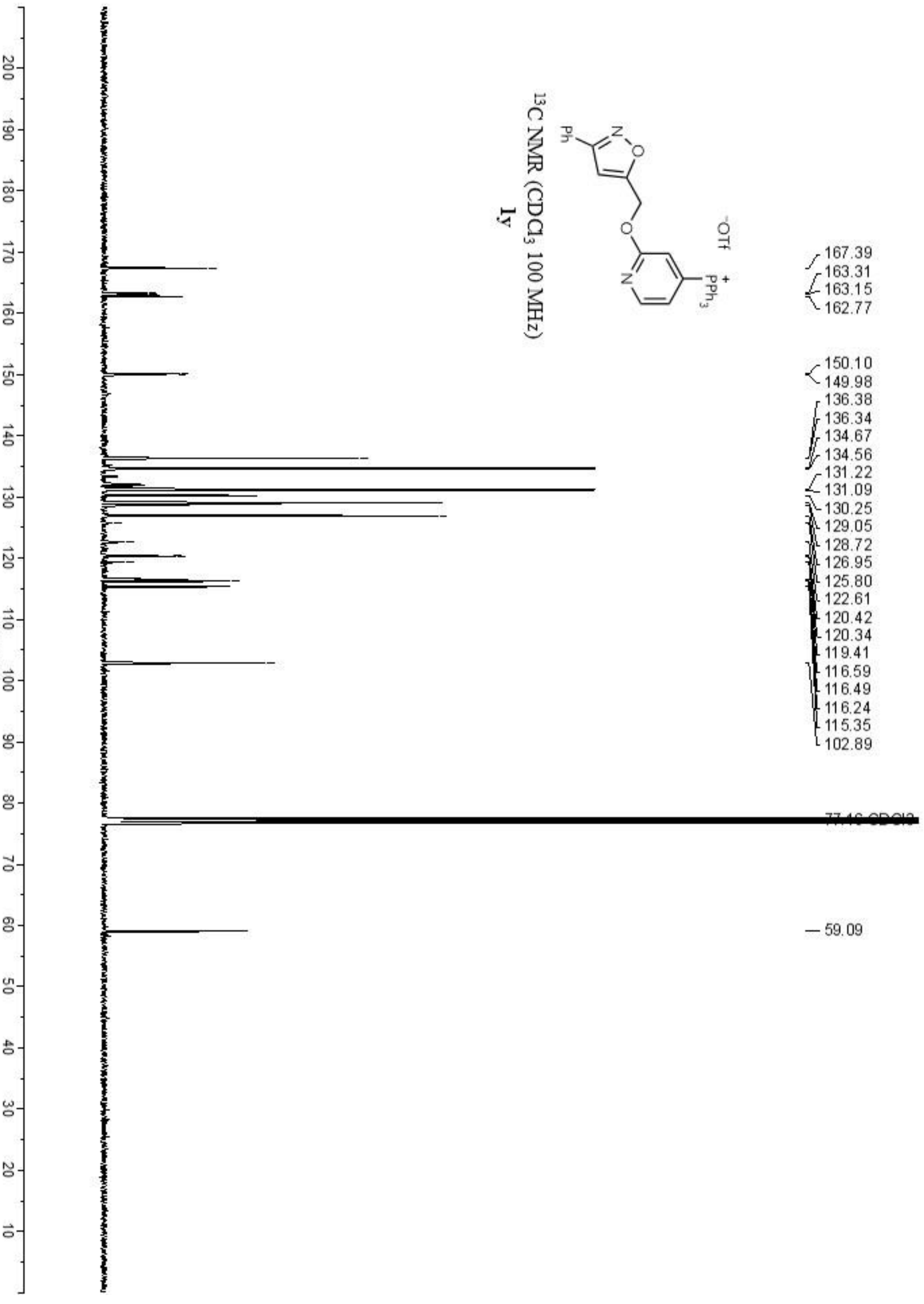
-OTf
+
PPh3
N
O
O
N
Ph
13C NMR (CDCl3 100 MHz)
1y
167.39
163.31
163.15
162.77
150.10
149.98
136.38
136.34
134.67
134.56
131.22
131.09
130.25
129.05
128.72
126.95
125.80
122.61
120.42
120.34
119.41
116.59
116.49
116.24
115.35
102.89
59.09
200
190
180
170
160
150
140
130
120
110
100
90
80
70
60
50
40
30
20
10
f1 (ppm)

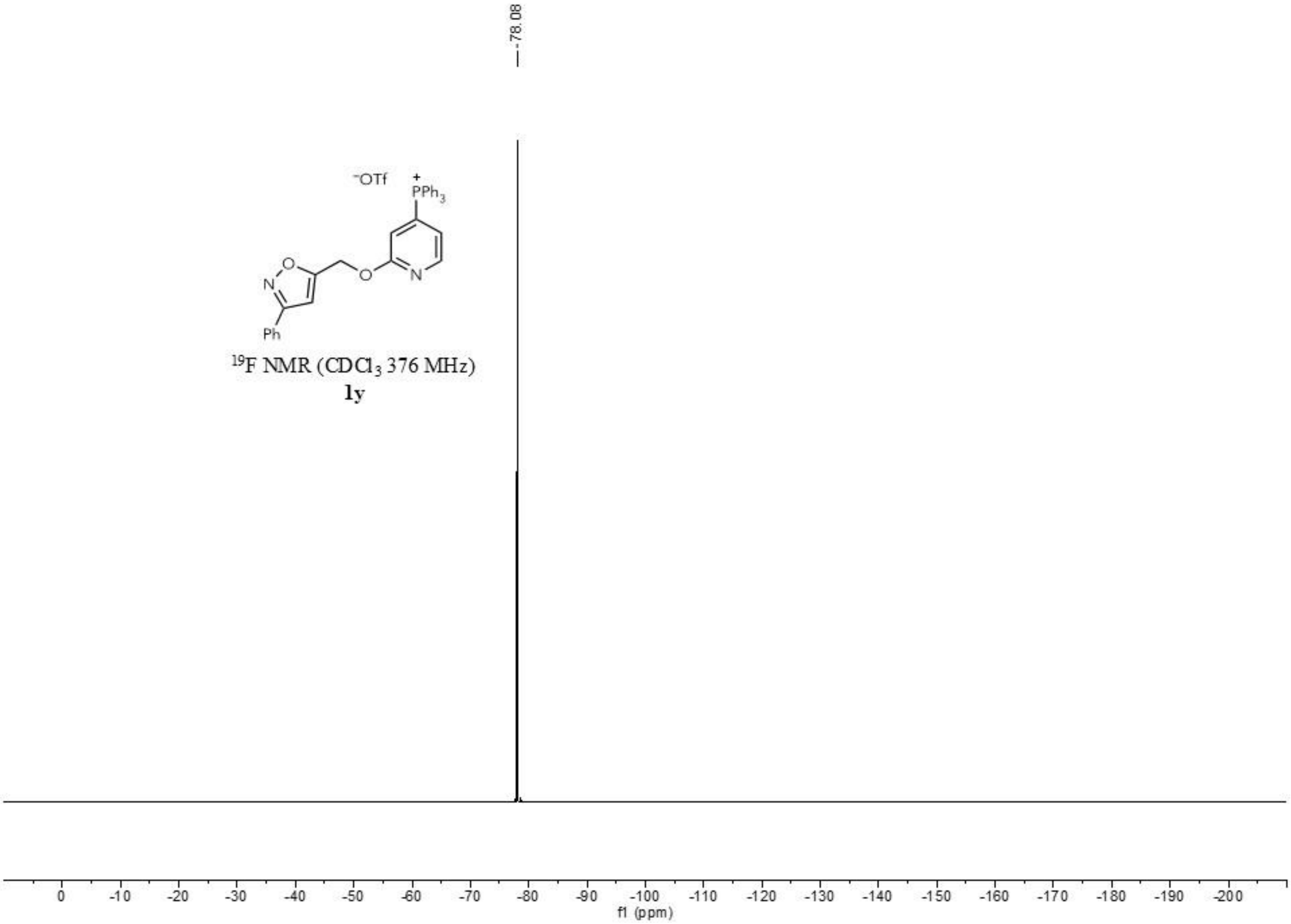
-78.08
⁻OTf
+
PPh3
O
N
N
O
Ph
19F NMR (CDCl3 376 MHz)
1y
0
-10
-20
-30
-40
-50
-60
-70
-80
-90
-100
-110
-120
-130
-140
-150
-160
-170
-180
-190
-200
f1 (ppm)

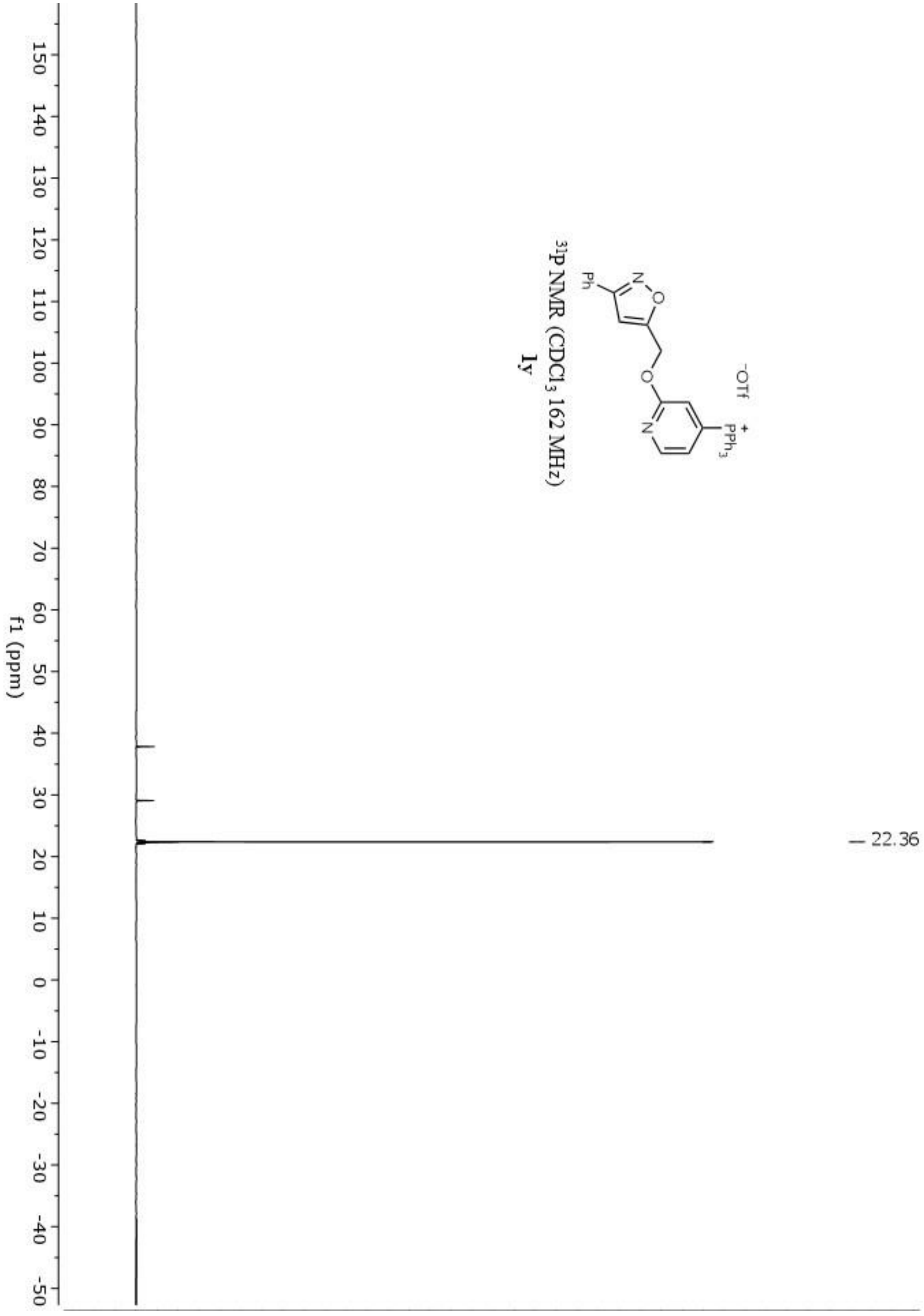

-OTf
+
PPh3
Ph
N
O
O
N
31P NMR (CDCl3 162 MHz)
1y
22.36
f1 (ppm)
150
140
130
120
110
100
90
80
70
60
50
40
30
20
10
0
-10
-20
-30
-40
-50

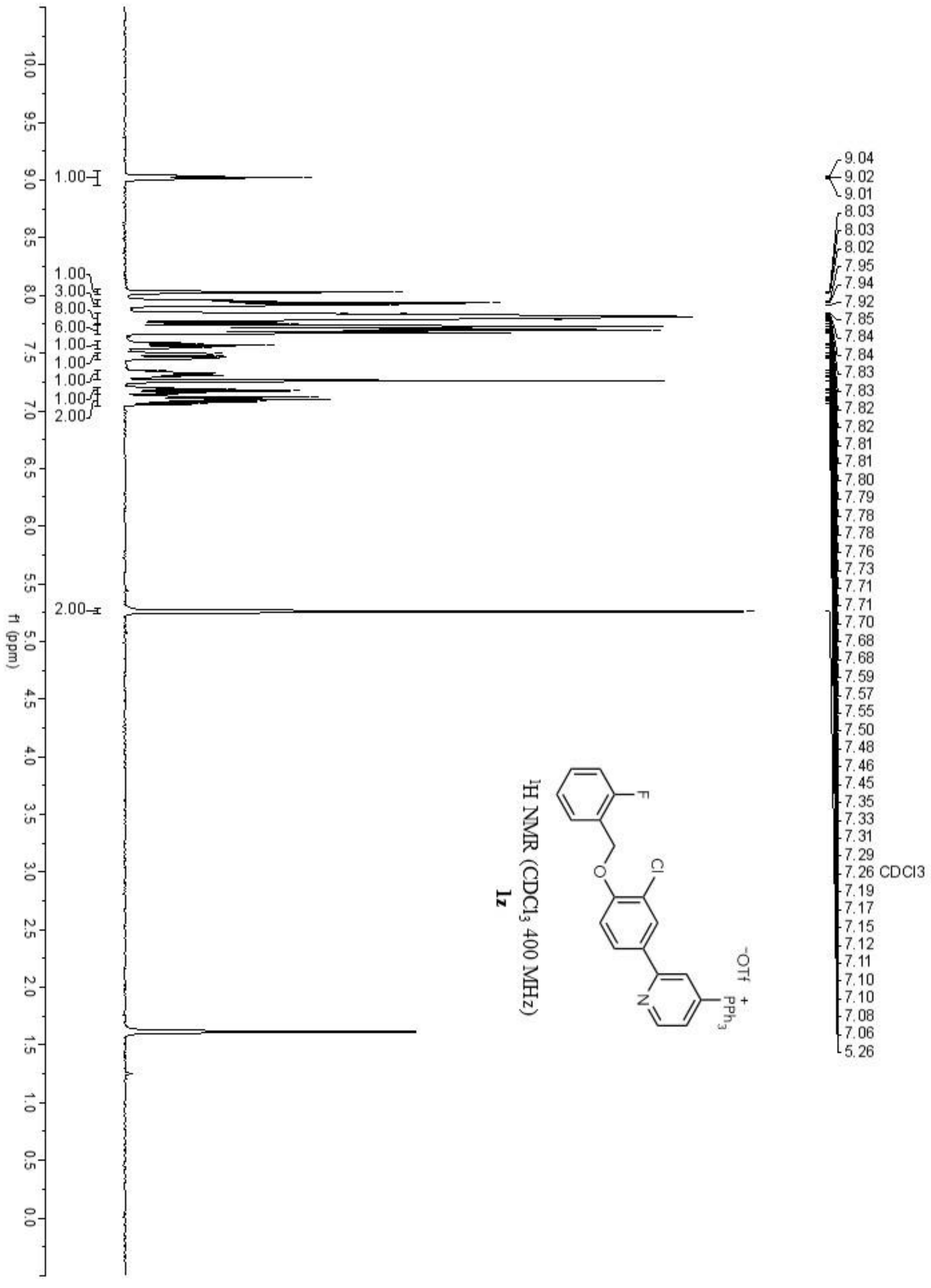

1H NMR (CDCl3 400 MHz)
1z
-OTf
+
PPh3
Cl
F
O
N
9.04
9.02
9.01
8.03
8.03
8.02
7.95
7.94
7.92
7.85
7.84
7.84
7.83
7.83
7.82
7.82
7.81
7.81
7.80
7.79
7.78
7.78
7.76
7.73
7.71
7.71
7.70
7.68
7.68
7.59
7.57
7.55
7.50
7.48
7.46
7.45
7.35
7.33
7.31
7.29
7.26 CDCl3
7.19
7.17
7.15
7.12
7.11
7.10
7.10
7.08
7.06
5.26
1.00
1.00
3.00
8.00
6.00
1.00
1.00
1.00
1.00
2.00
2.00
f1 (ppm)
10.0
9.5
9.0
8.5
8.0
7.5
7.0
6.5
6.0
5.5
5.0
4.5
4.0
3.5
3.0
2.5
2.0
1.5
1.0
0.5
0.0

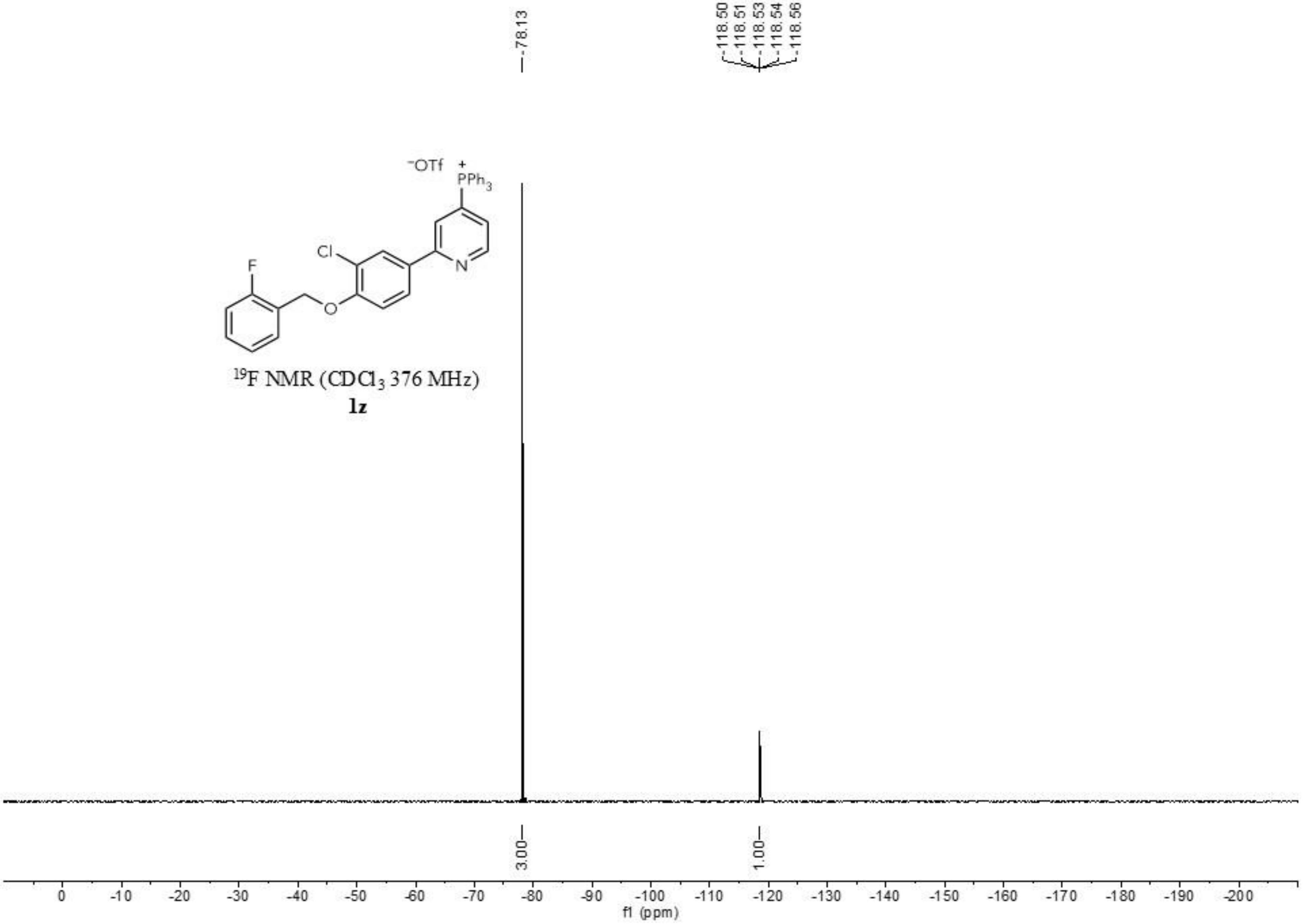
-78.13
-118.50
-118.51
-118.53
-118.54
-118.56
⁻OTf
+
PPh3
Cl
F
O
N
19F NMR (CDCl3 376 MHz)
1z
3.00
1.00
0
-10
-20
-30
-40
-50
-60
-70
-80
-90
-100
-110
-120
-130
-140
-150
-160
-170
-180
-190
-200
f1 (ppm)

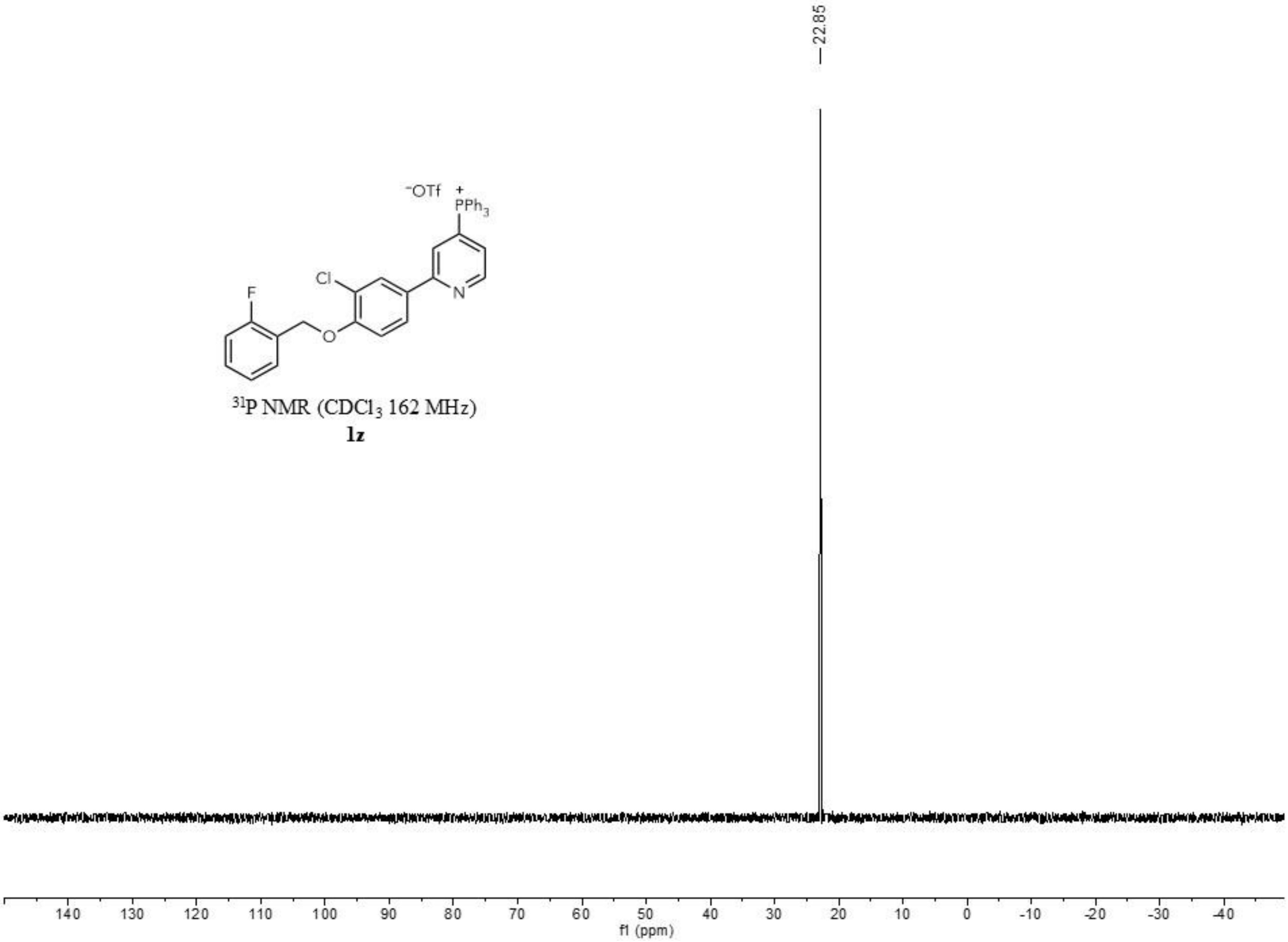

—22.85
⁻OTf
+
PPh3
Cl
F
O
N
31P NMR (CDCl3 162 MHz)
1z
140 130 120 110 100 90 80 70 60 50 40 30 20 10 0 -10 -20 -30 -40
f1 (ppm)

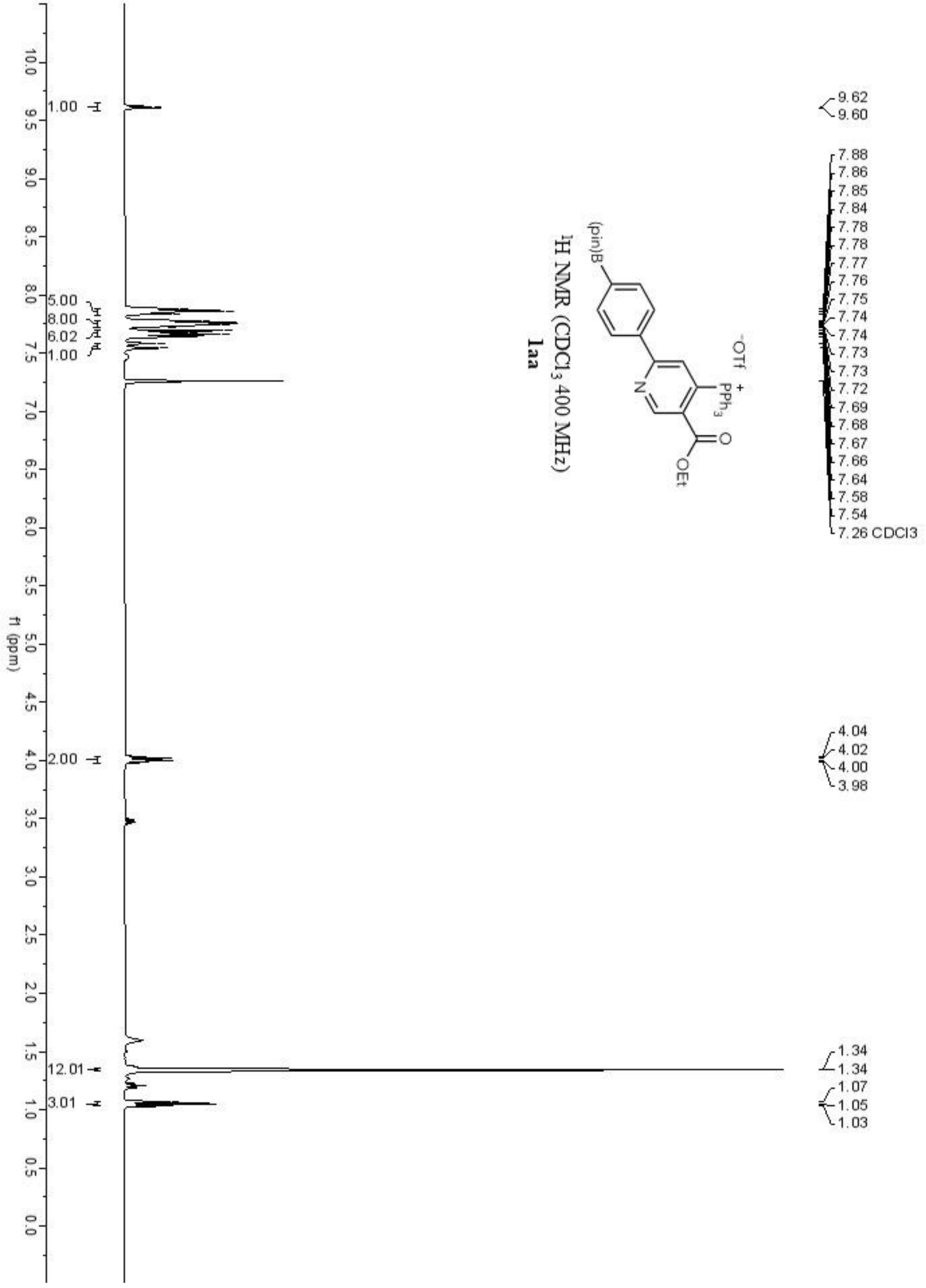

¹H NMR (CDCl₃ 400 MHz)
1aa
(pin)B
⁻OTf
⁺PPh₃
N
O
OEt
9.62
9.60
7.88
7.86
7.85
7.84
7.78
7.78
7.77
7.76
7.75
7.74
7.74
7.73
7.73
7.72
7.69
7.68
7.67
7.66
7.64
7.58
7.54
7.26 CDCl3
4.04
4.02
4.00
3.98
1.34
1.34
1.07
1.05
1.03
1.00
5.00
8.00
6.02
1.00
2.00
12.01
3.01
f1 (ppm)

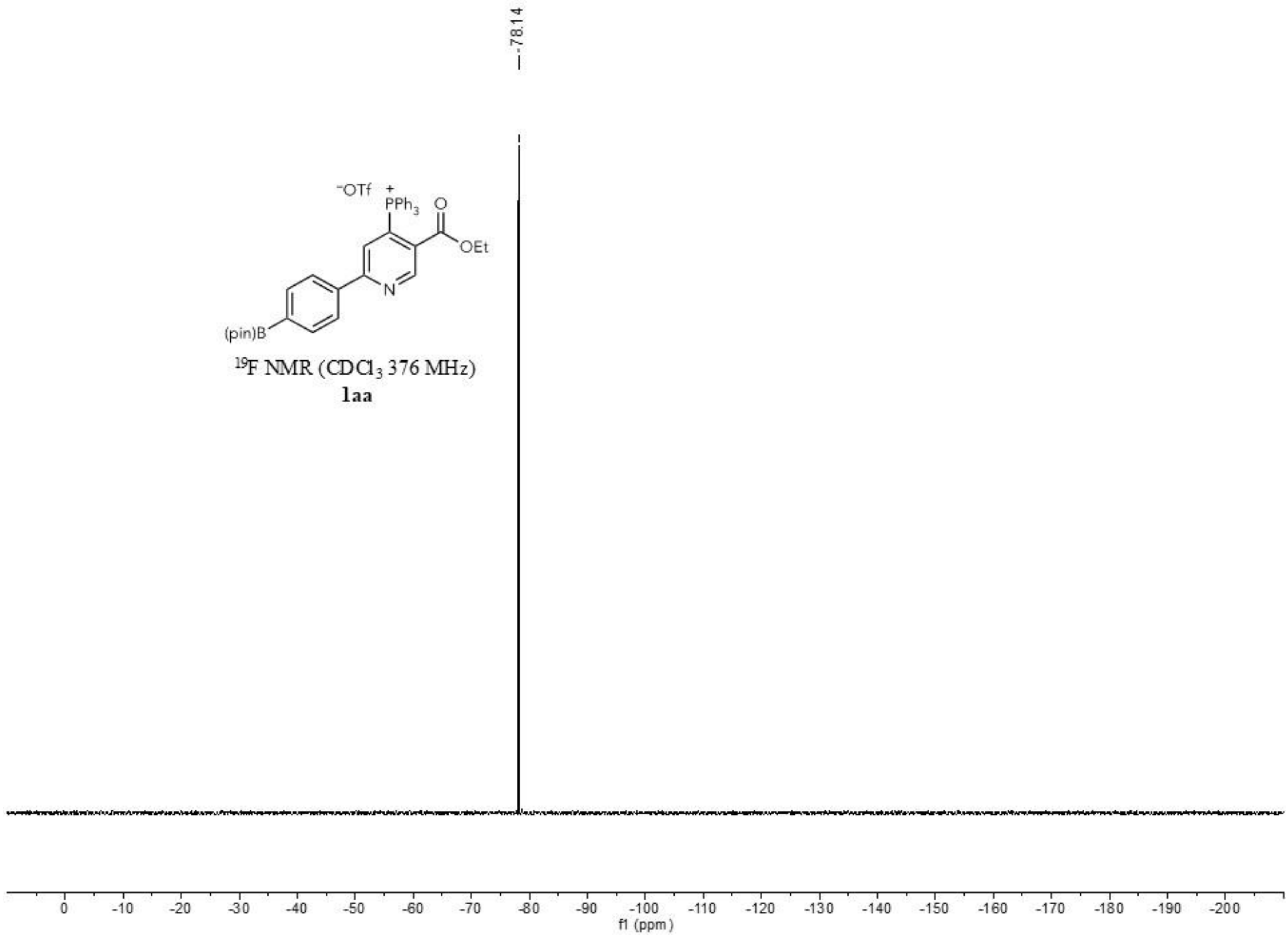
-78.14
-OTf
+
PPh3
O
OEt
N
(pin)B
19F NMR (CDCl3 376 MHz)
1aa
0
-10
-20
-30
-40
-50
-60
-70
-80
-90
-100
-110
-120
-130
-140
-150
-160
-170
-180
-190
-200
f1 (ppm)

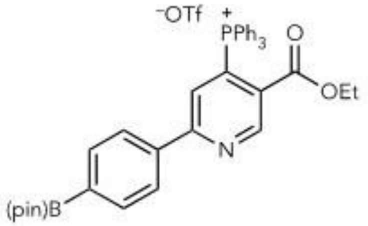

^{31}P NMR ($CDCl_3$ 162 MHz)

1aa

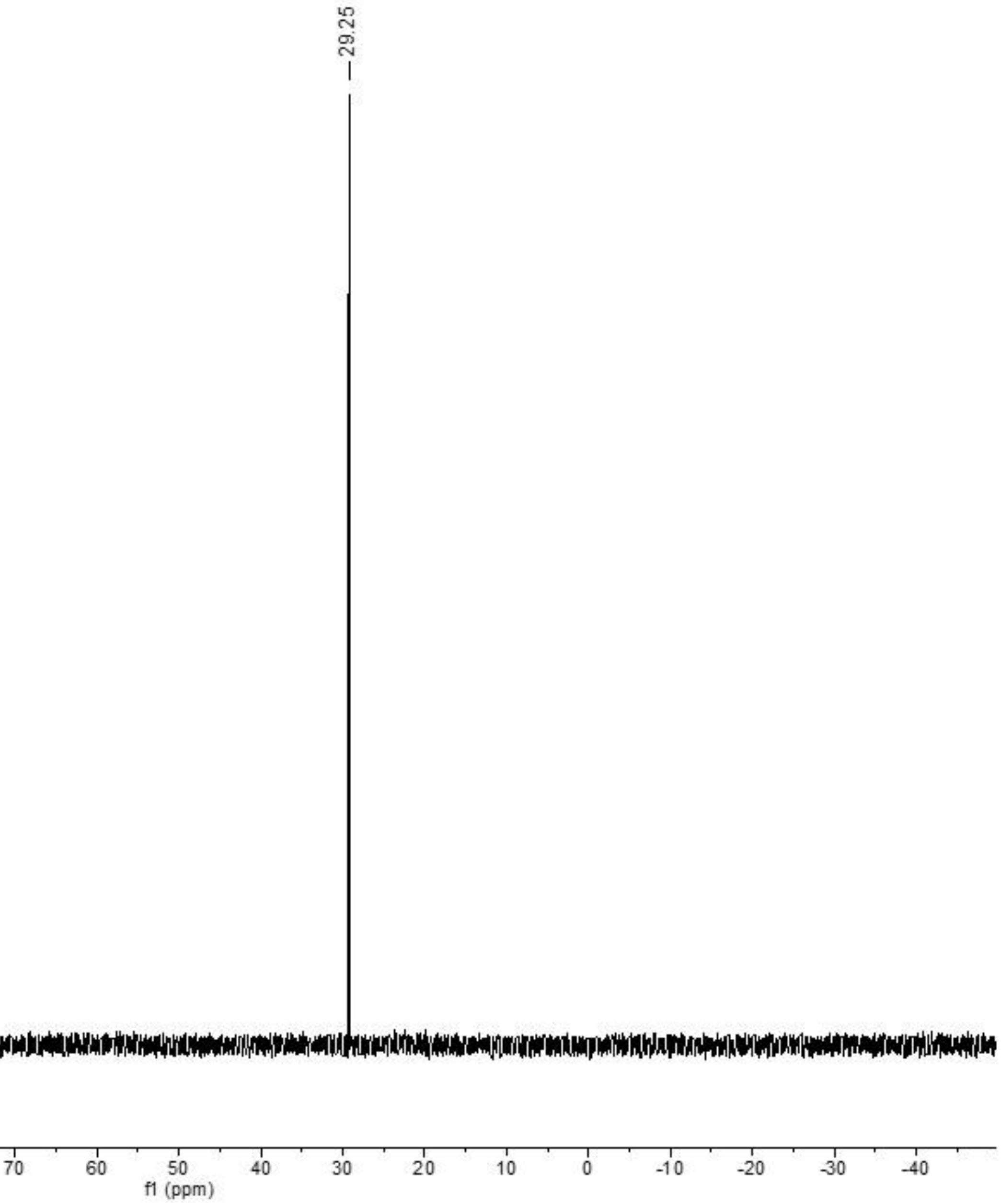

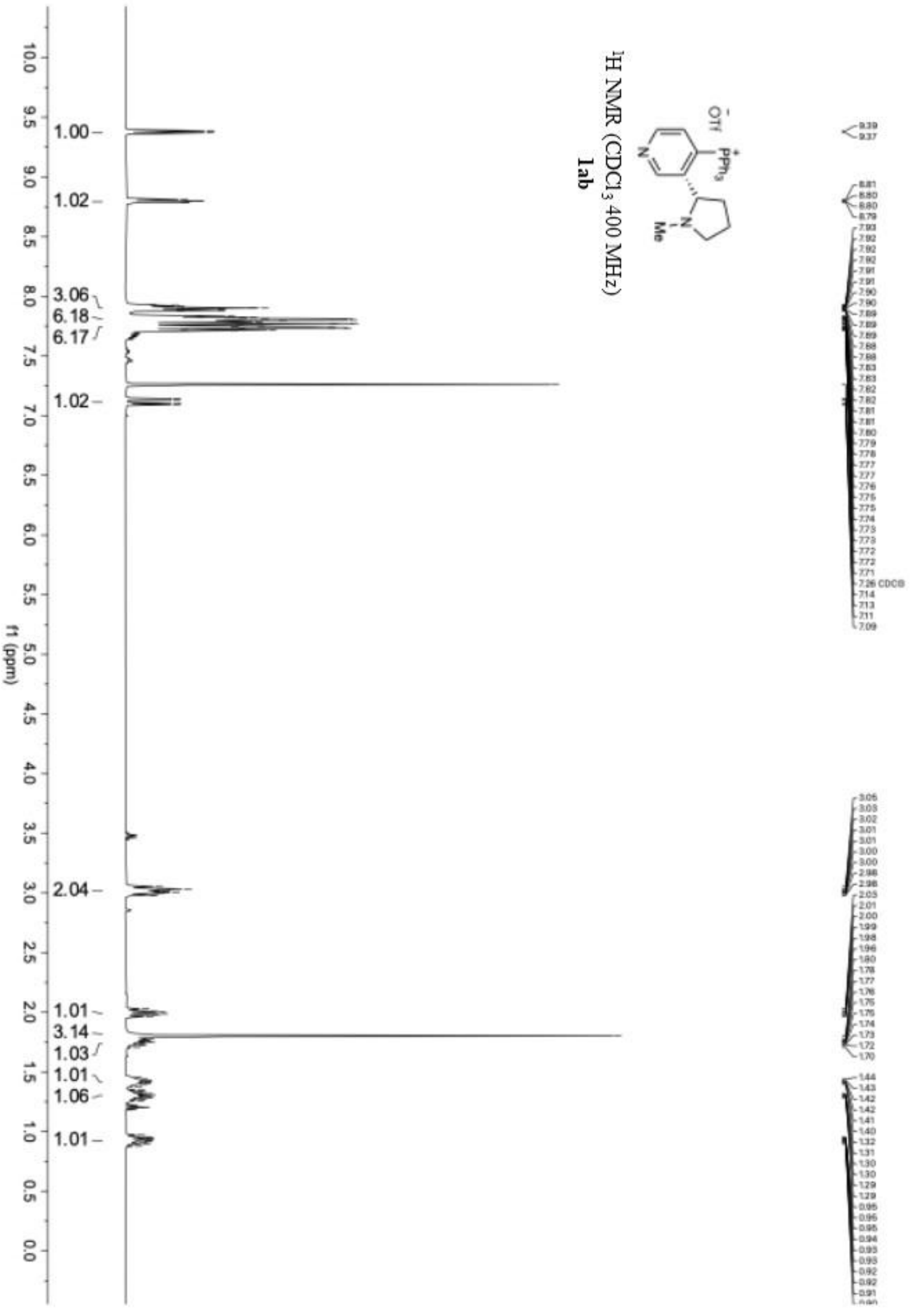

¹H NMR (CDCl₃ 400 MHz)
1ab
f1 (ppm)

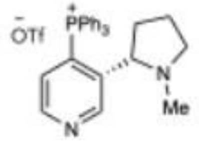

^{19}F NMR ($CDCl_3$ 376 MHz)

1ab

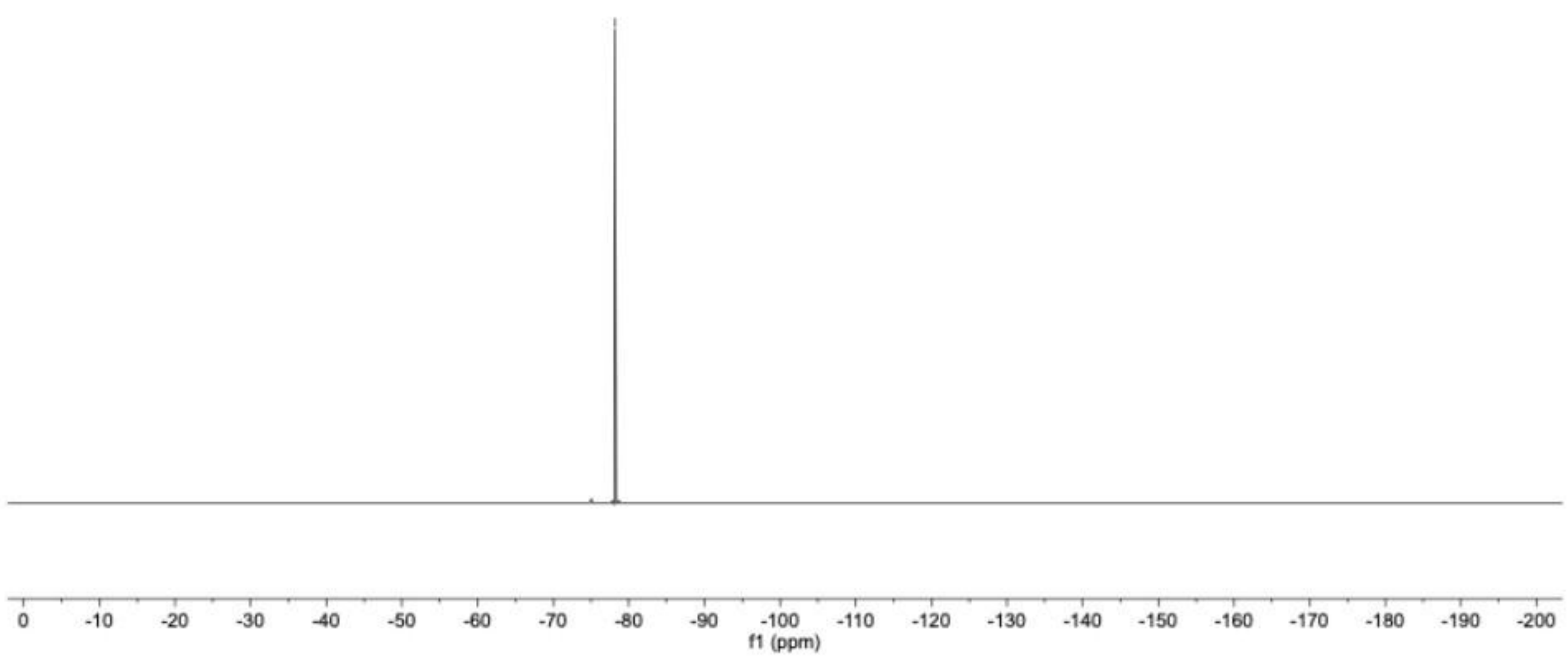

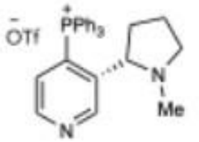

^{31}P NMR ($CDCl_3$ 162 MHz)
1ab

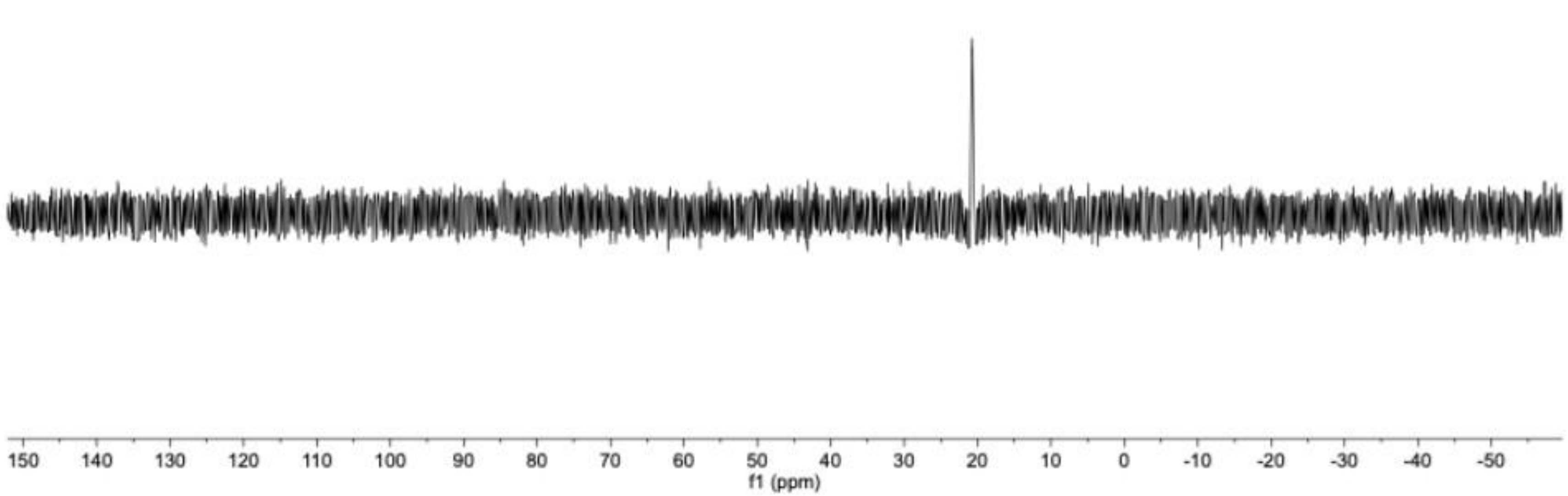

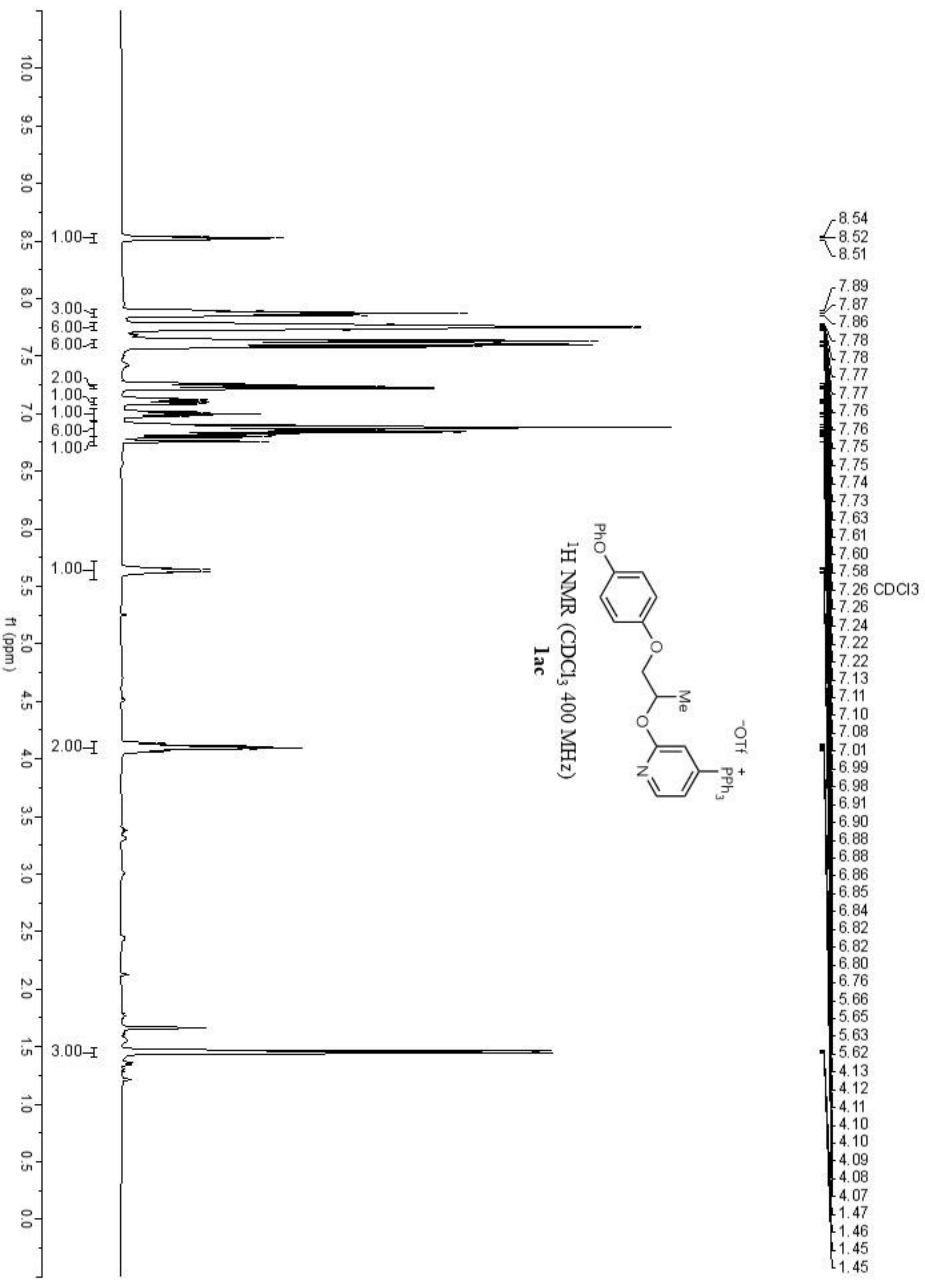

1H NMR (CDCl3 400 MHz)
1ac
PhO
O
O
Me
N
-OTf
+
PPh3
8.54
8.52
8.51
7.89
7.87
7.86
7.78
7.78
7.77
7.77
7.76
7.76
7.75
7.75
7.74
7.73
7.63
7.61
7.60
7.58
7.26 CDCl3
7.26
7.24
7.22
7.22
7.13
7.11
7.10
7.08
7.01
6.99
6.98
6.91
6.90
6.88
6.88
6.86
6.85
6.84
6.82
6.82
6.80
6.76
5.66
5.65
5.63
5.62
4.13
4.12
4.11
4.10
4.10
4.09
4.08
4.07
1.47
1.46
1.45
1.45
1.00
3.00
6.00
6.00
2.00
1.00
1.00
6.00
1.00
1.00
2.00
3.00
f1 (ppm)

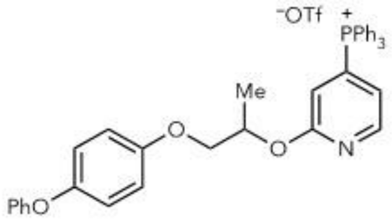

^{19}F NMR ($CDCl_3$ 376 MHz)
1ac

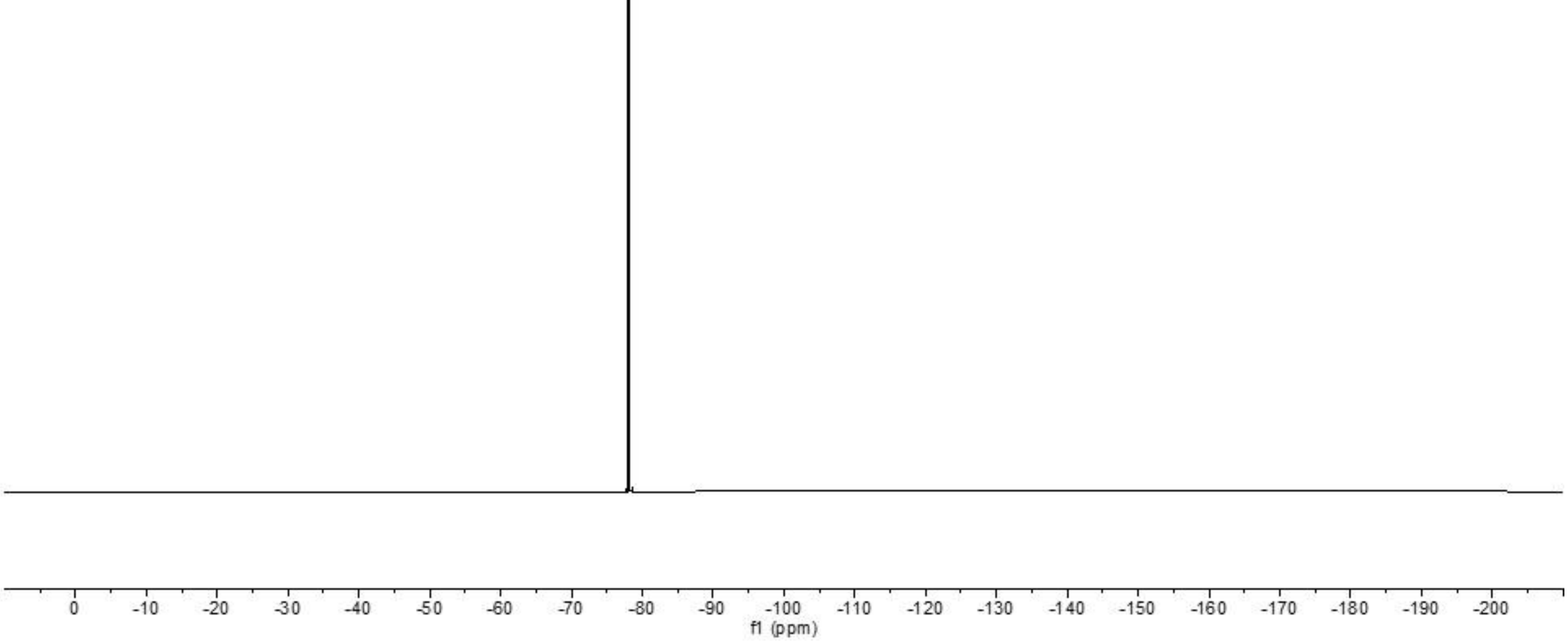

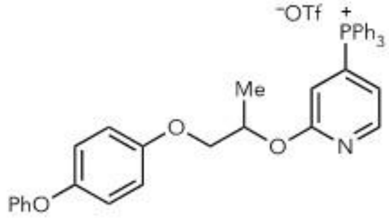

^{31}P NMR ($CDCl_3$ 162 MHz)
1ac

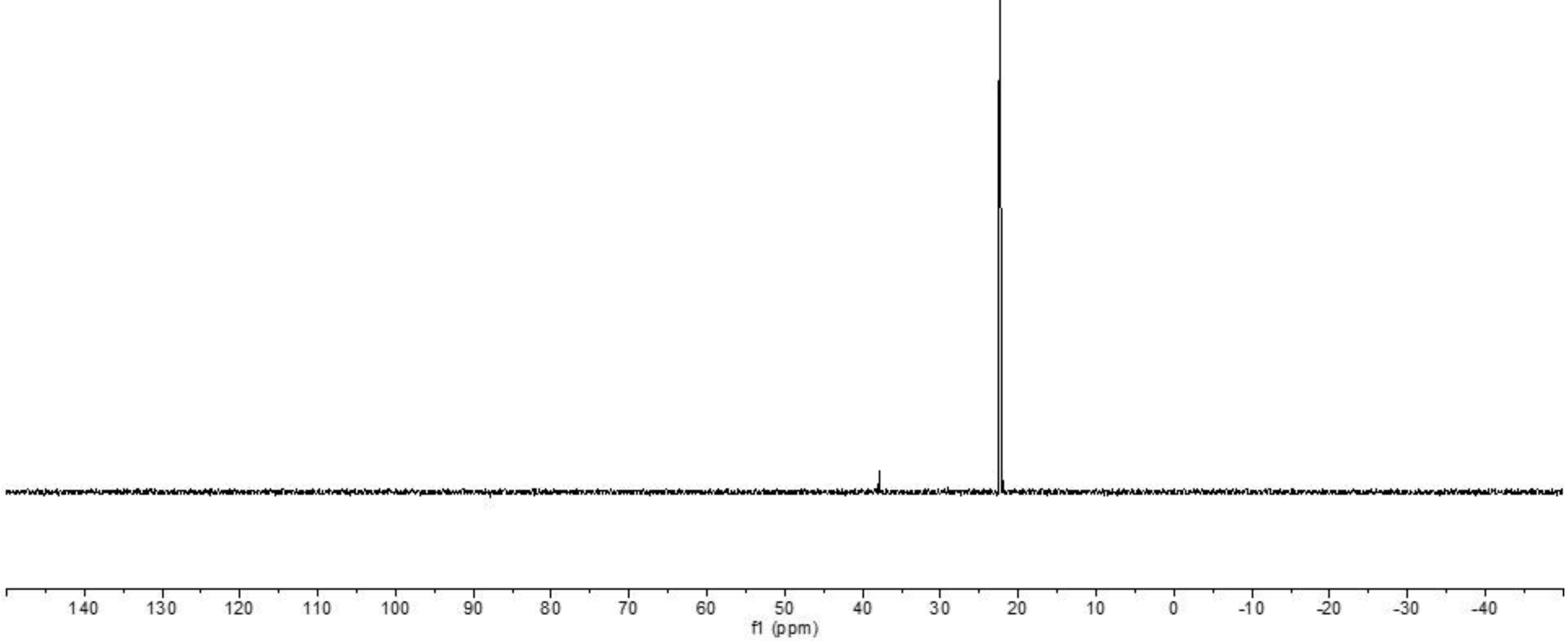

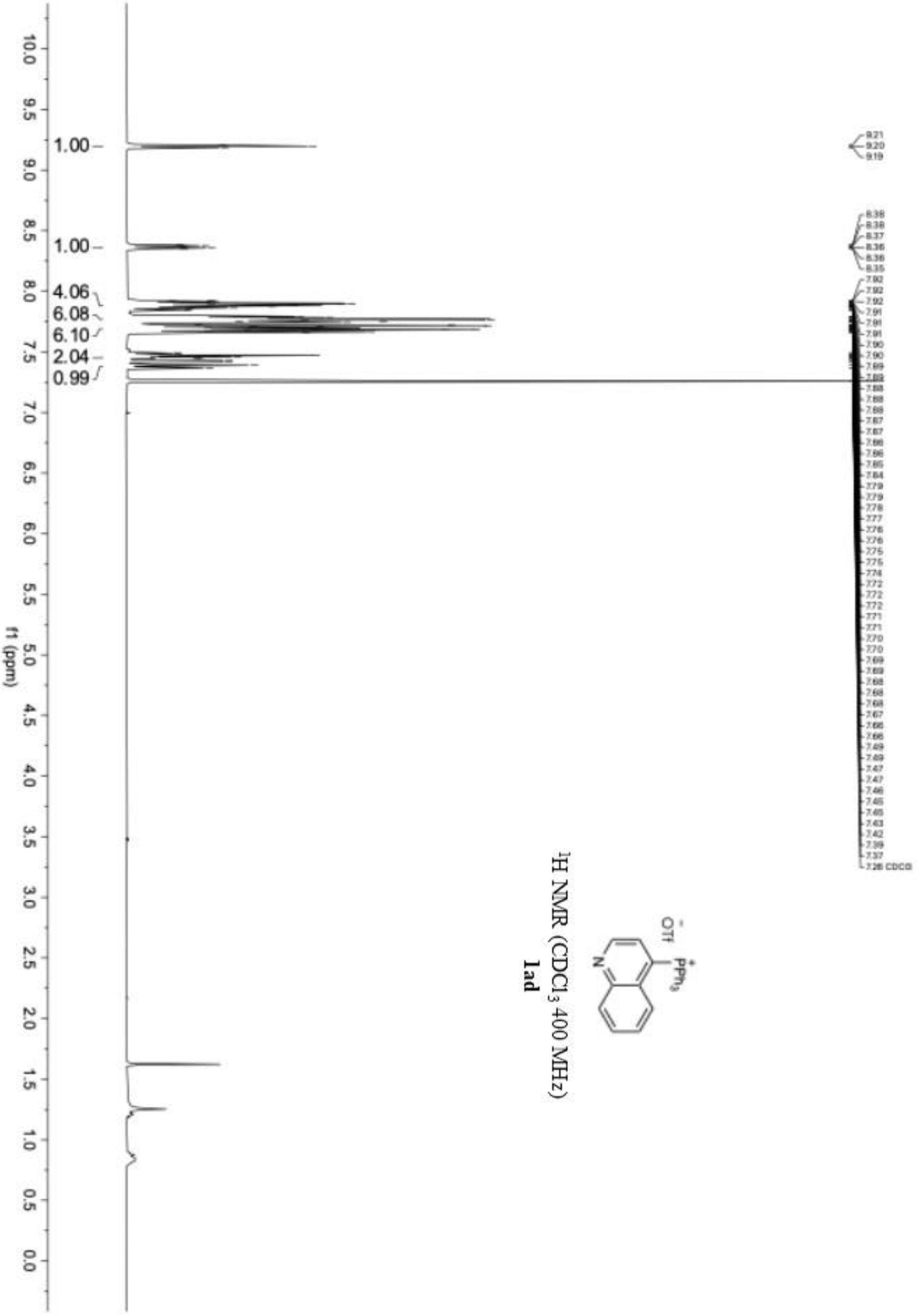

^1H NMR ($CDCl_3$ 400 MHz)
1ad

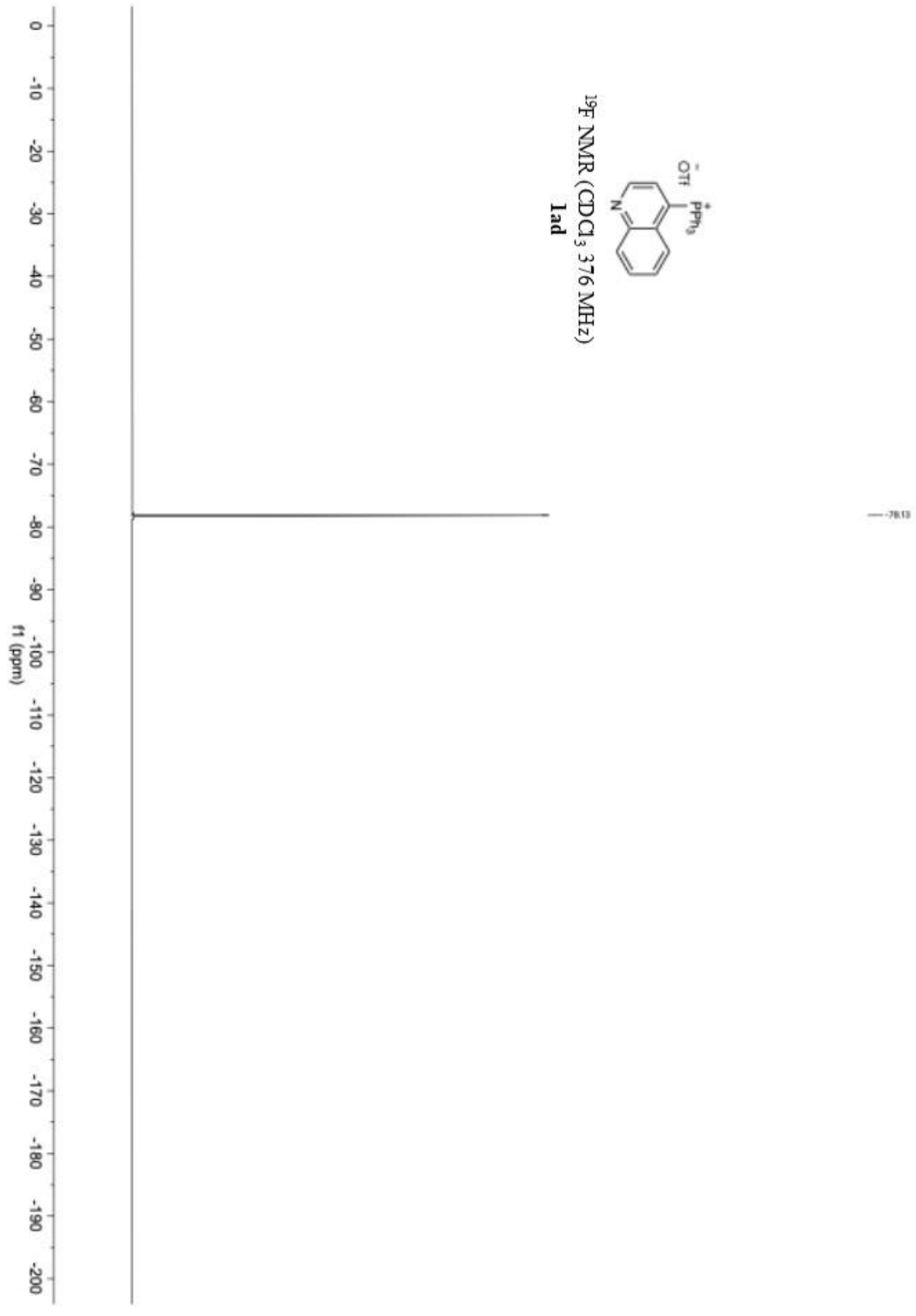

19F NMR (CDCl3 376 MHz)
1ad
OTf
PPh3
N
-78.13
0
-10
-20
-30
-40
-50
-60
-70
-80
-90
-100
-110
-120
-130
-140
-150
-160
-170
-180
-190
-200
f1 (ppm)

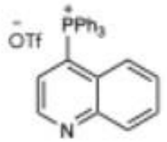

^{31}P NMR ($CDCl_3$ 162 MHz)

1ad

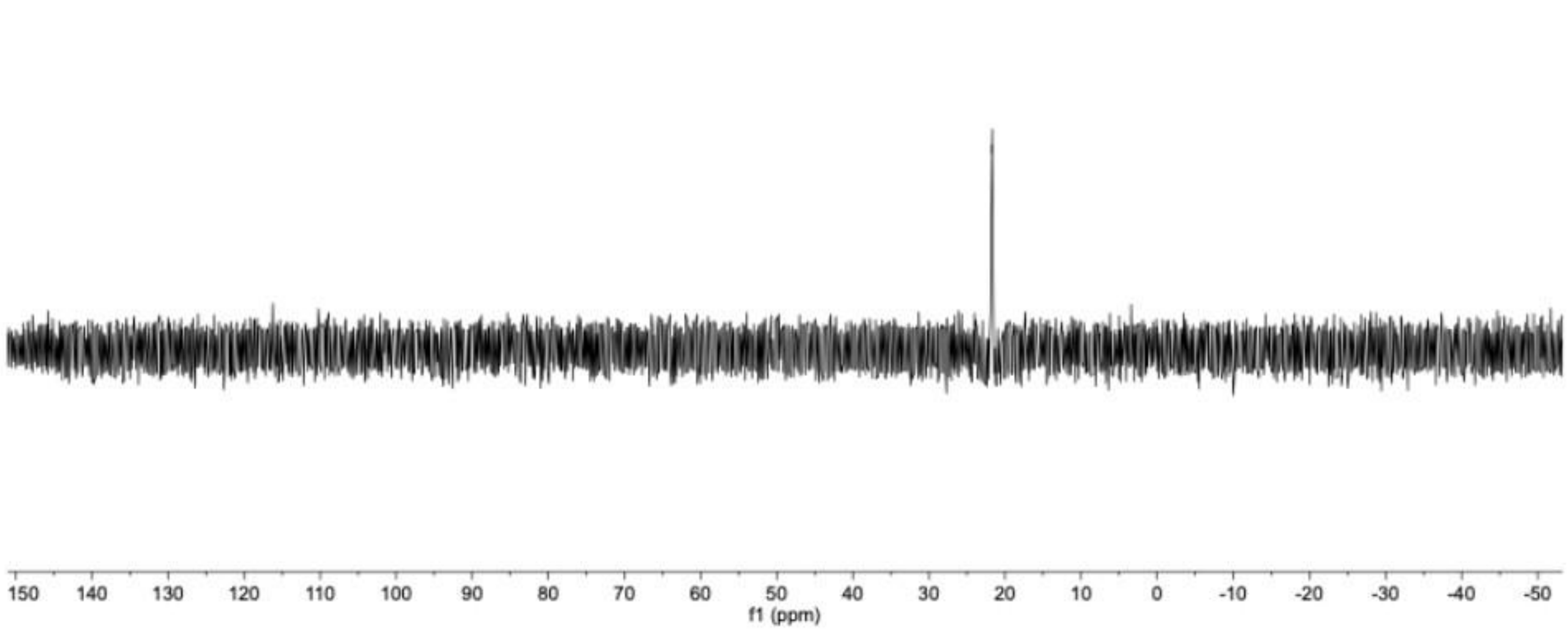

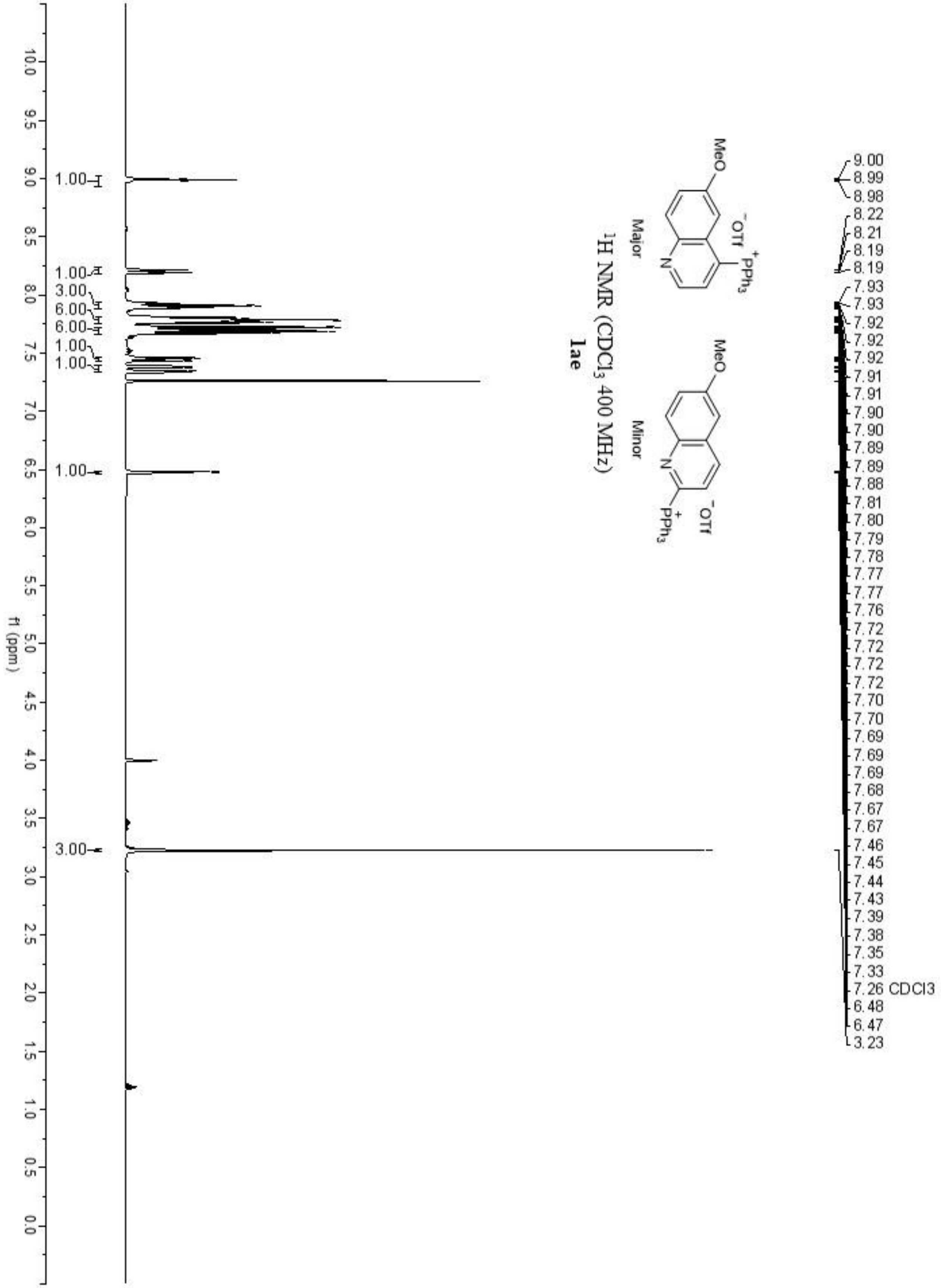

1H NMR (CDCl3 400 MHz)
1ae
Major
Minor
MeO
OTf
PPh3
N
9.00
8.99
8.98
8.22
8.21
8.19
8.19
7.93
7.93
7.92
7.92
7.92
7.91
7.91
7.90
7.90
7.89
7.89
7.88
7.81
7.80
7.79
7.78
7.77
7.77
7.76
7.72
7.72
7.72
7.72
7.70
7.70
7.69
7.69
7.69
7.68
7.67
7.67
7.46
7.45
7.44
7.43
7.39
7.38
7.35
7.33
7.26 CDCl3
6.48
6.47
3.23
1.00
1.00
3.00
6.00
6.00
1.00
1.00
1.00
3.00
f1 (ppm)

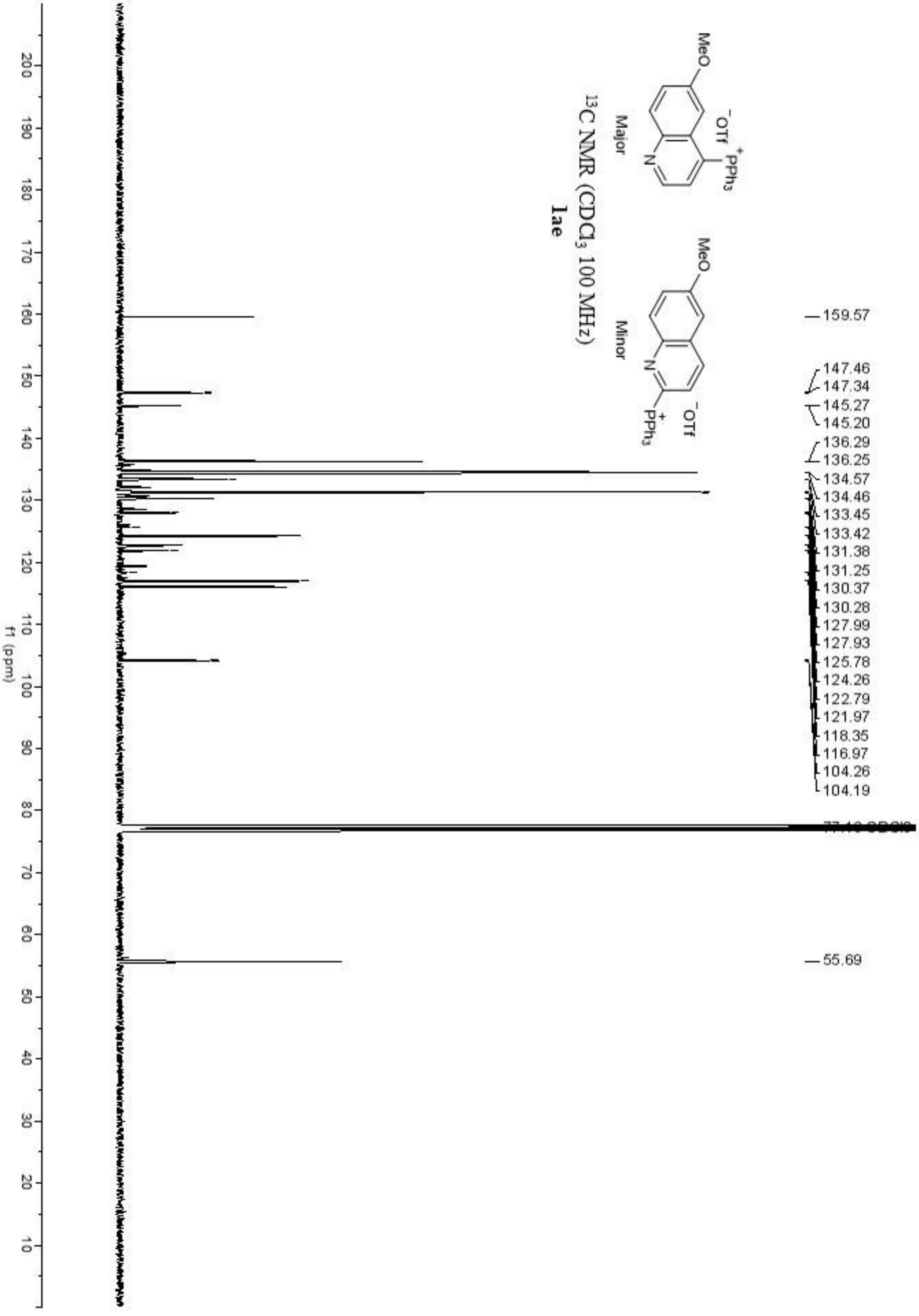
MeO
$^{-}$OTf
$^{+}$PPh$_3$
N
Major
MeO
$^{-}$OTf
$^{+}$PPh$_3$
N
Minor
^{13}C NMR (CDCl$_3$ 100 MHz)
1ae
159.57
147.46
147.34
145.27
145.20
136.29
136.25
134.57
134.46
133.45
133.42
131.38
131.25
130.37
130.28
127.99
127.93
125.78
124.26
122.79
121.97
118.35
116.97
104.26
104.19
55.69
200
190
180
170
160
150
140
130
120
110
100
90
80
70
60
50
40
30
20
10
f1 (ppm)

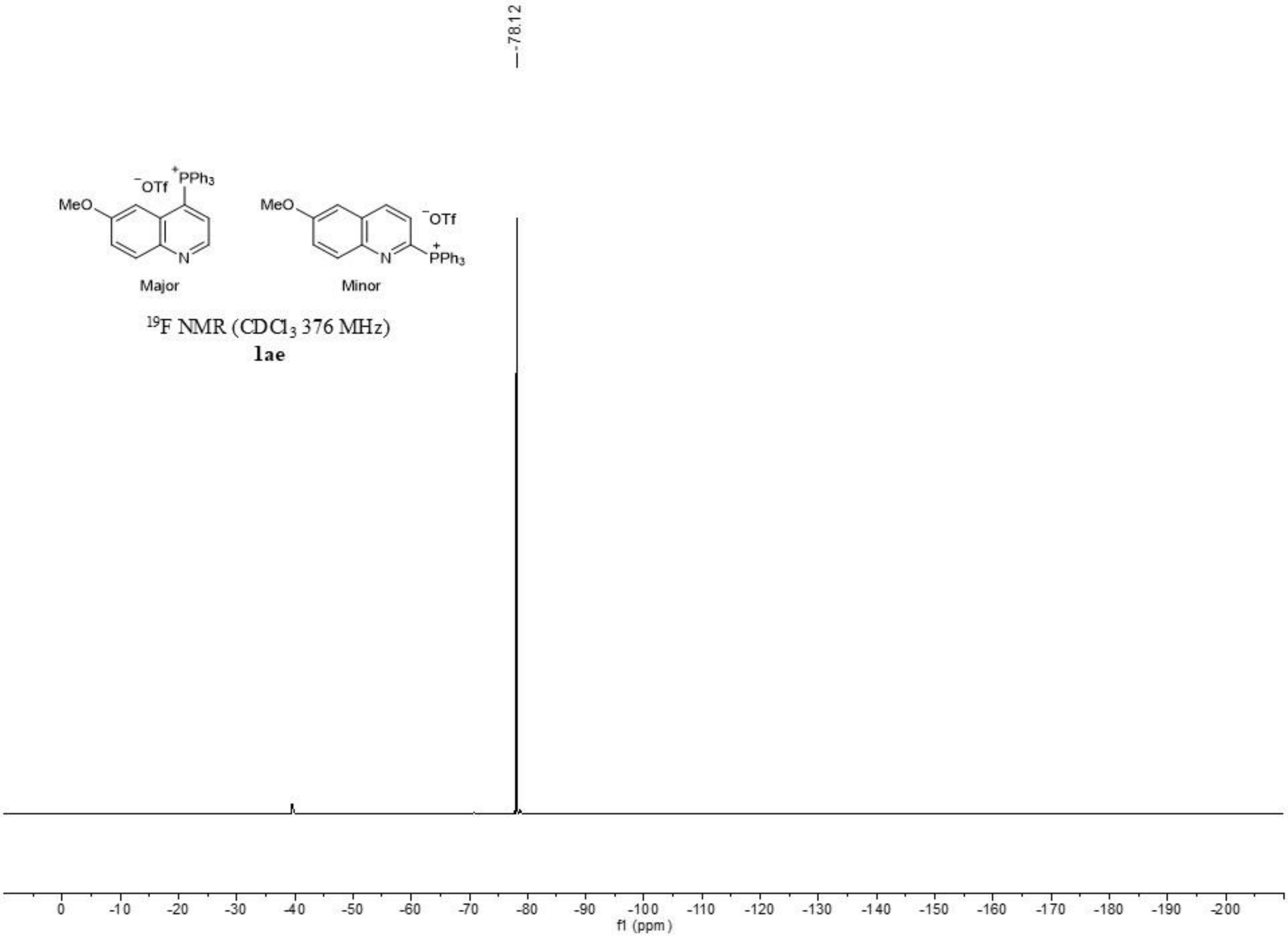
-78.12
MeO
⁻OTf
+PPh3
N
Major
Minor
19F NMR (CDCl3 376 MHz)
1ae
0
-10
-20
-30
-40
-50
-60
-70
-80
-90
-100
-110
-120
-130
-140
-150
-160
-170
-180
-190
-200
f1 (ppm)

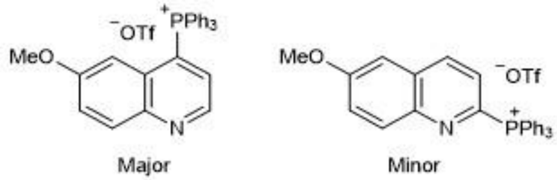

^{31}P NMR ($CDCl_3$ 162 MHz)

1ae

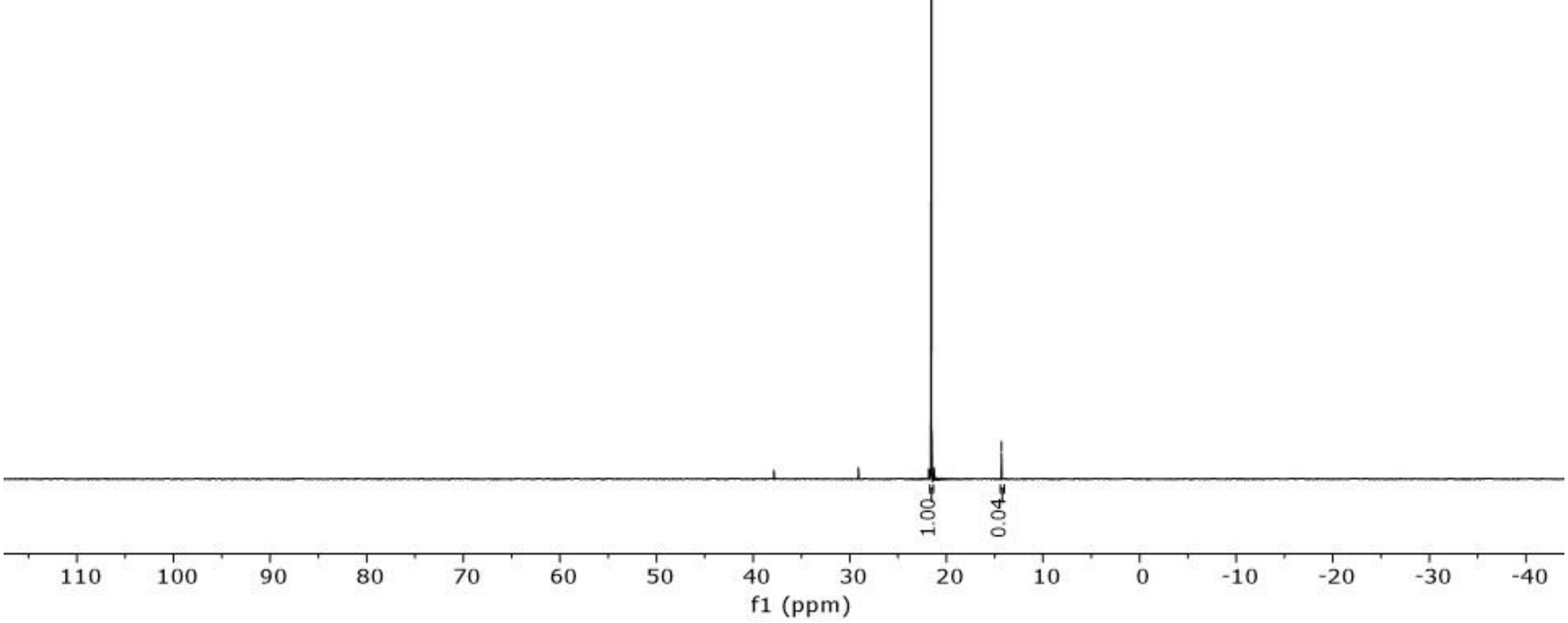

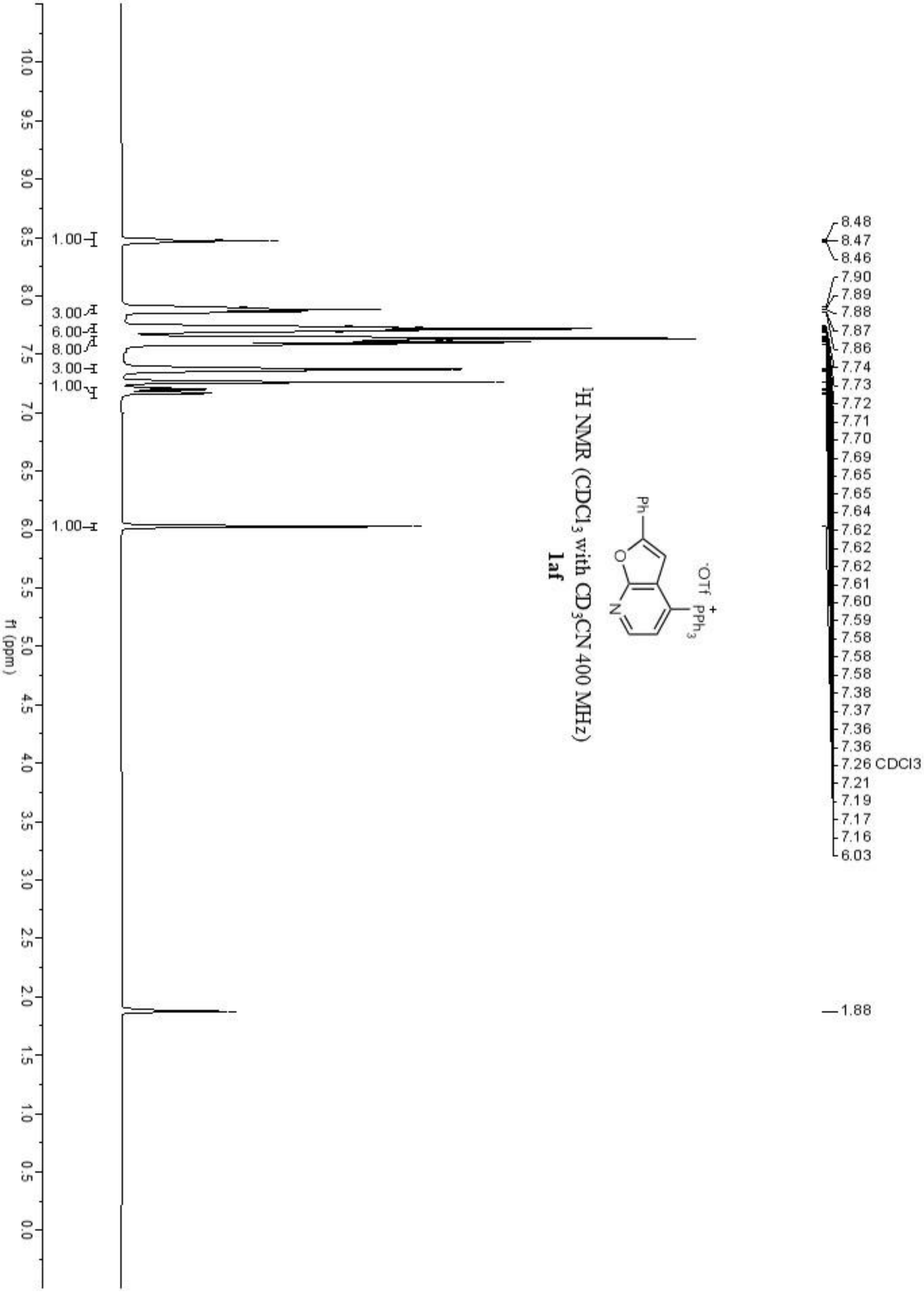

^{1}H NMR (CDCl$_3$ with CD$_3$CN 400 MHz)
1af
Ph
O
N
$^-$OTf
$^+$PPh$_3$
f1 (ppm)
8.48
8.47
8.46
7.90
7.89
7.88
7.87
7.86
7.74
7.73
7.72
7.71
7.70
7.69
7.65
7.65
7.64
7.62
7.62
7.62
7.61
7.60
7.59
7.58
7.58
7.58
7.38
7.37
7.36
7.36
7.26 CDCl3
7.21
7.19
7.17
7.16
6.03
1.88
1.00
3.00
6.00
8.00
3.00
1.00
1.00

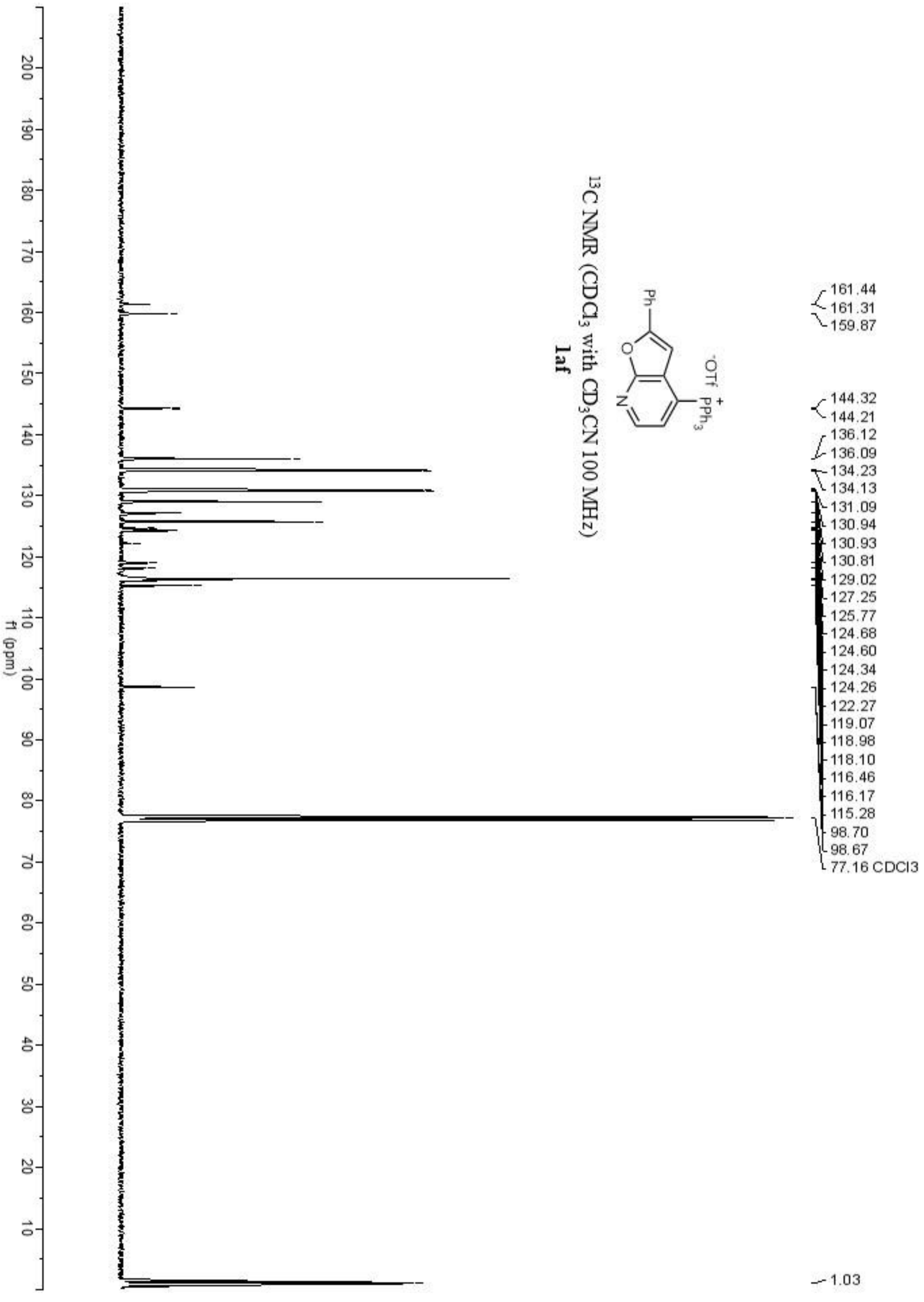

⁻OTf
PPh3
Ph
O
N
13C NMR (CDCl3 with CD3CN 100 MHz)
1af
161.44
161.31
159.87
144.32
144.21
136.12
136.09
134.23
134.13
131.09
130.94
130.93
130.81
129.02
127.25
125.77
124.68
124.60
124.34
124.26
122.27
119.07
118.98
118.10
116.46
116.17
115.28
98.70
98.67
77.16 CDCl3
1.03
200
190
180
170
160
150
140
130
120
110
100
90
80
70
60
50
40
30
20
10
f1 (ppm)

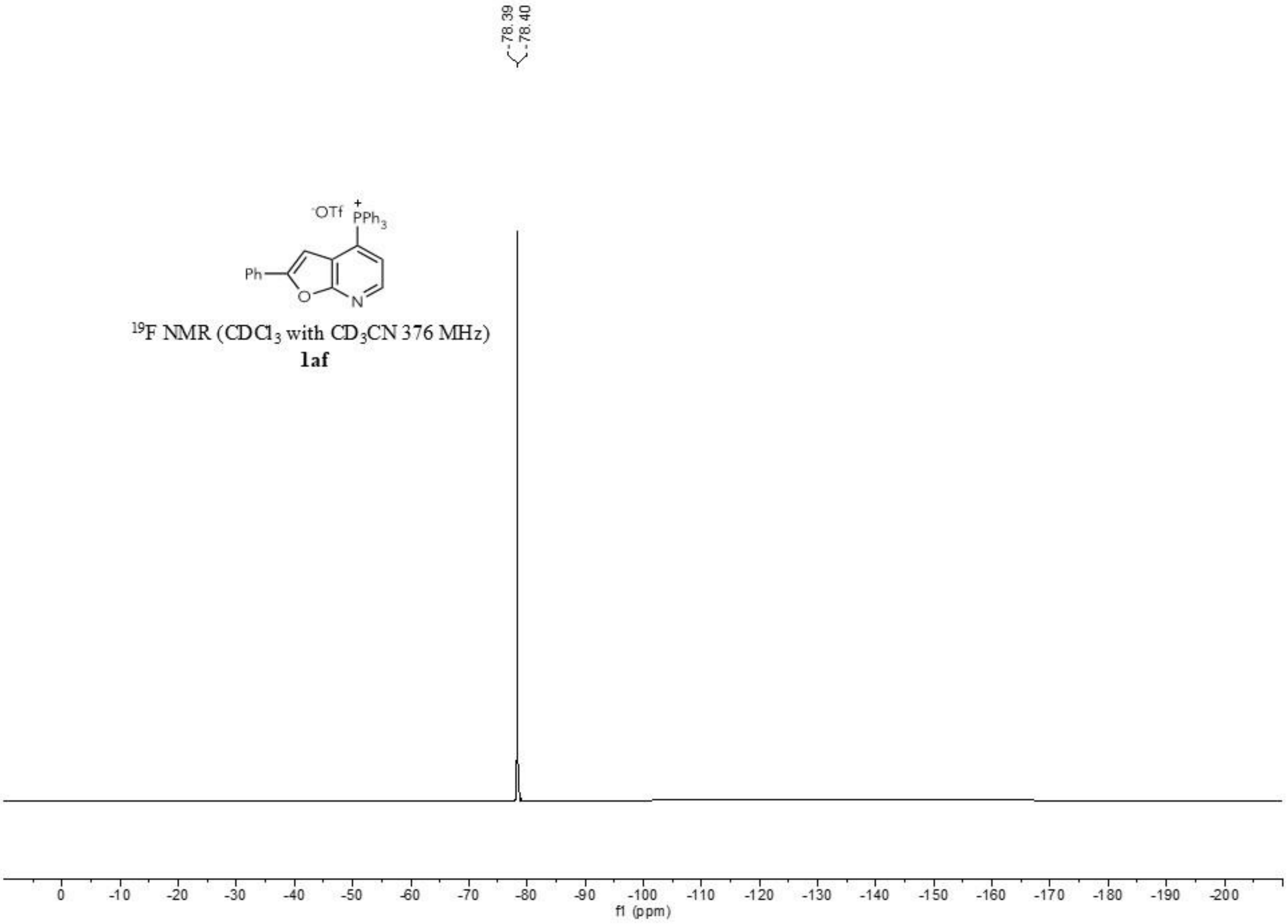

^{19}F NMR ($CDCl_3$ with CD_3CN 376 MHz)
1af

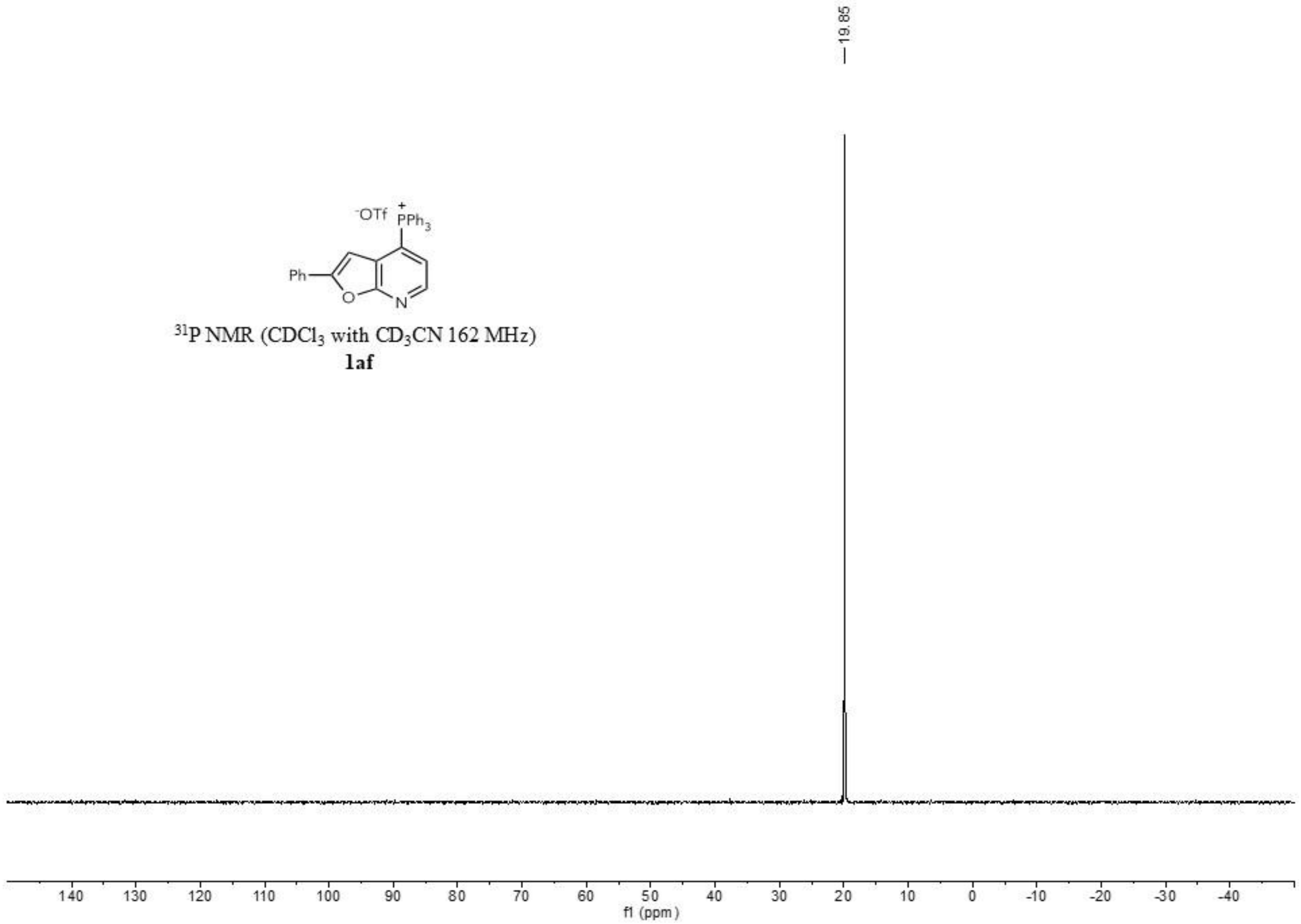

^{31}P NMR ($CDCl_3$ with CD_3CN 162 MHz)
1af

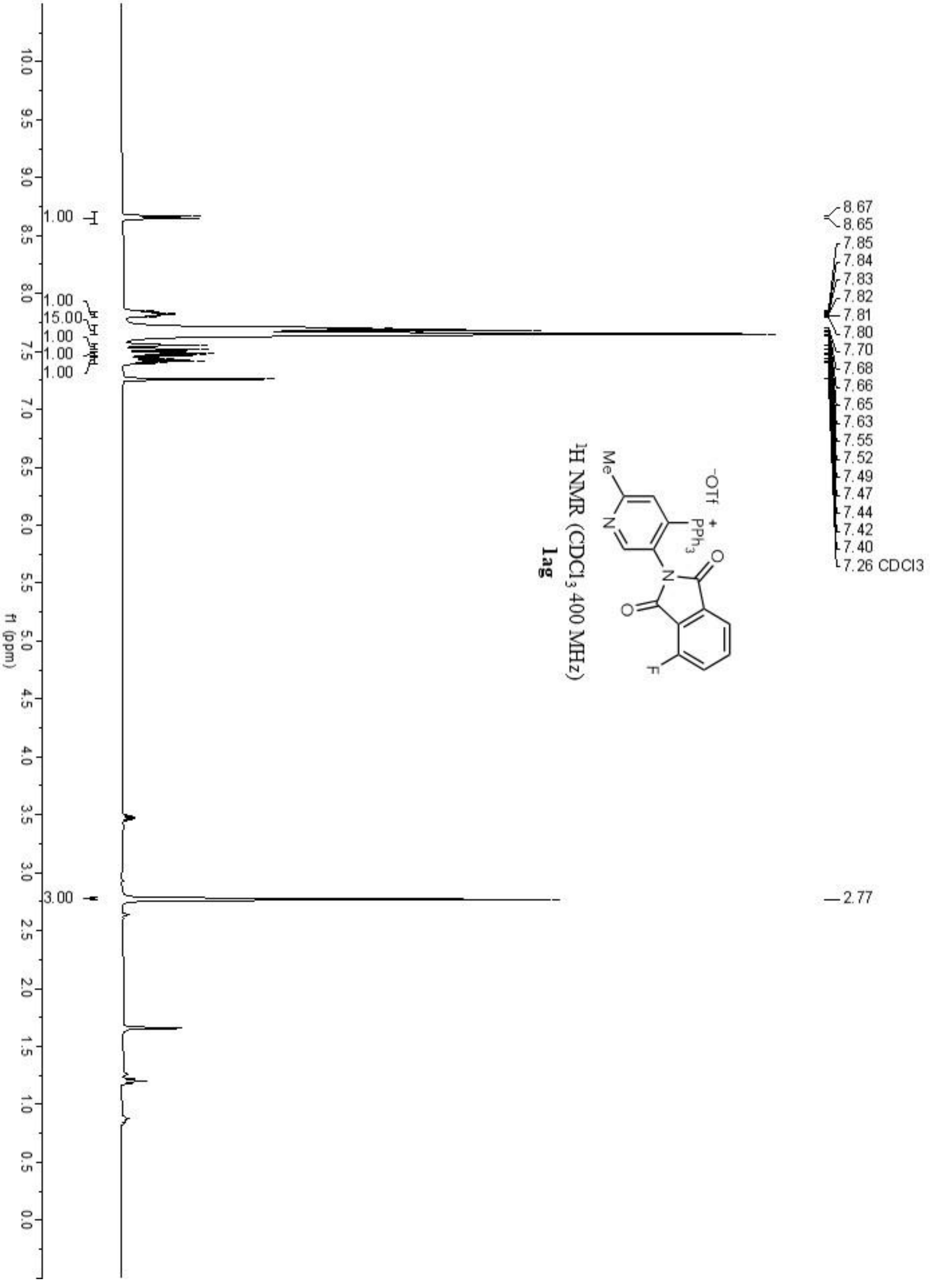

1H NMR (CDCl3 400 MHz)
1ag
-OTf
+
PPh3
Me
N
O
F
8.67
8.65
7.85
7.84
7.83
7.82
7.81
7.80
7.70
7.68
7.66
7.65
7.63
7.55
7.52
7.49
7.47
7.44
7.42
7.40
7.26 CDCl3
2.77
1.00
15.00
3.00
f1 (ppm)

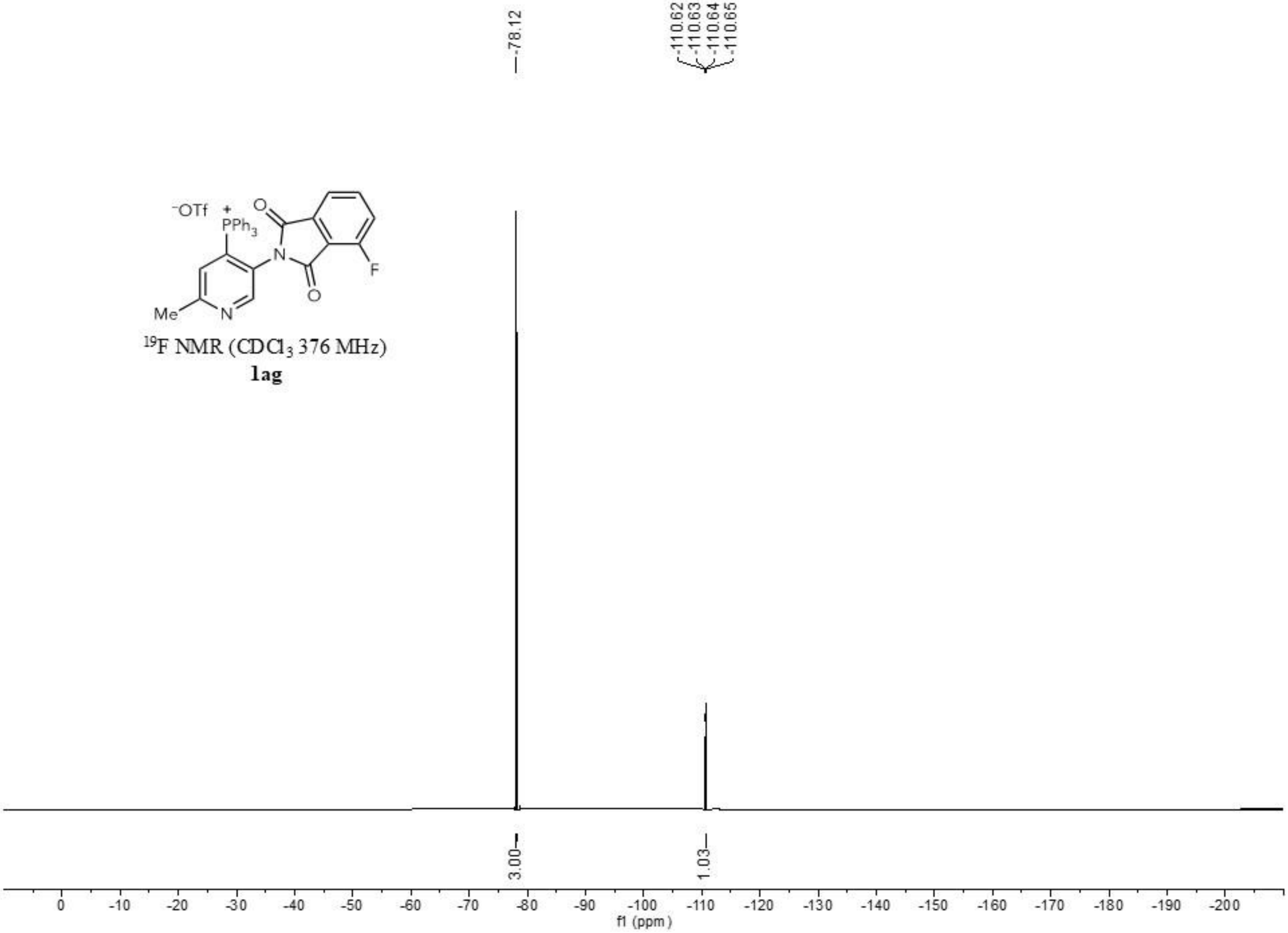

^{19}F NMR (CDCl$_3$ 376 MHz)
1ag

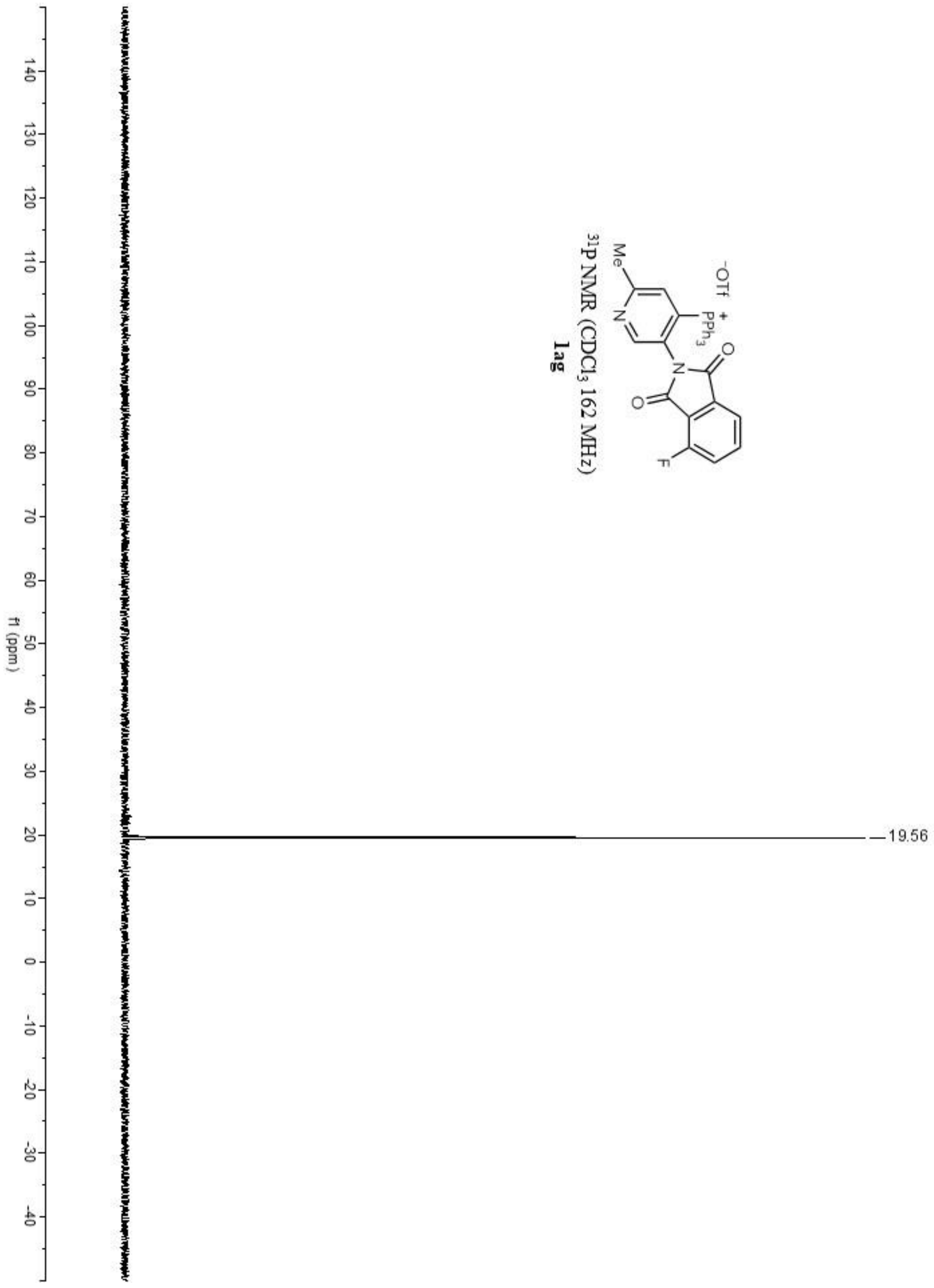

-OTf
+
PPh3
Me
N
O
F
31P NMR (CDCl3 162 MHz)
1ag
19.56
f1 (ppm)

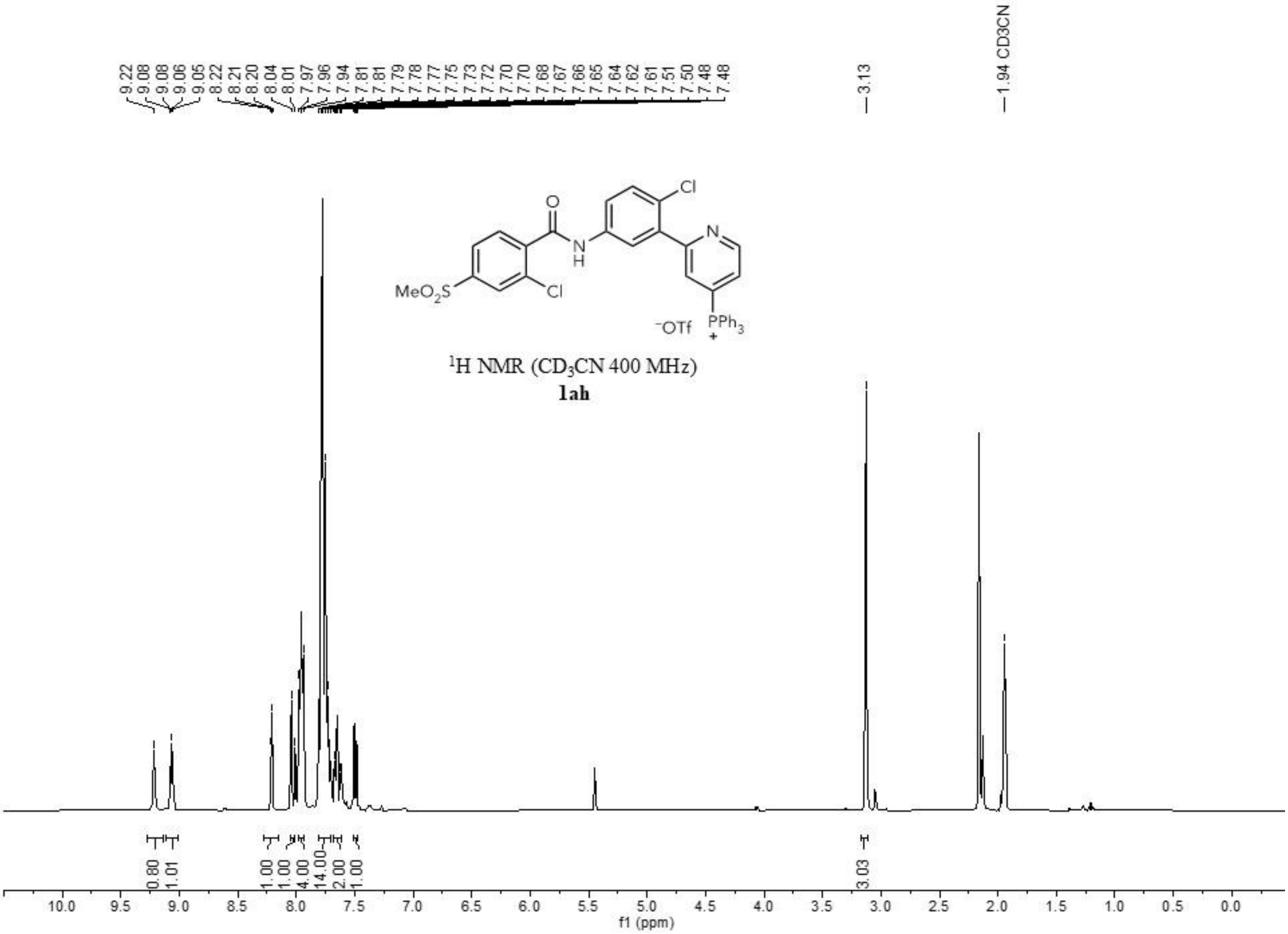

^{1}H NMR (CD_3CN 400 MHz)
1ah

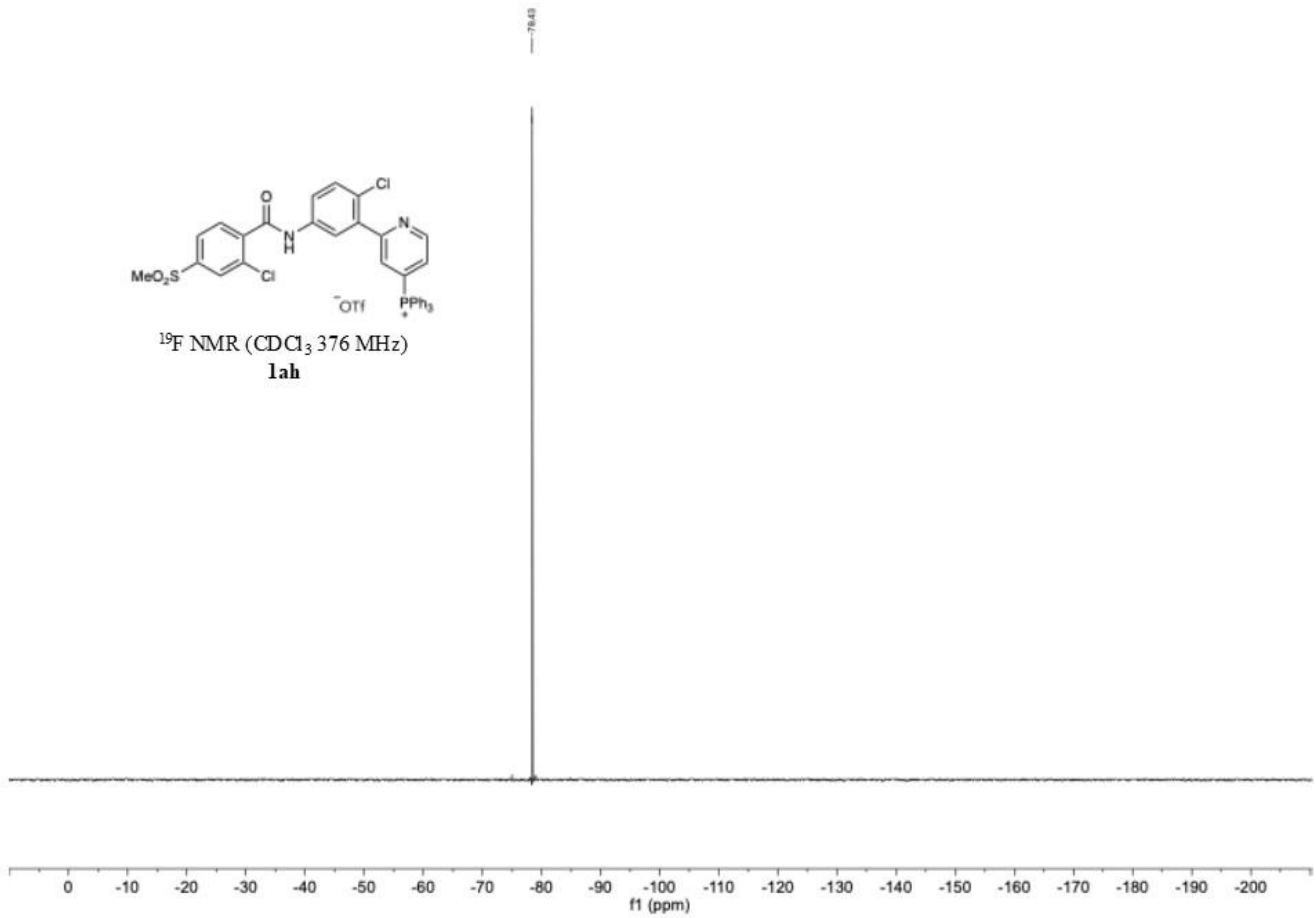

^{19}F NMR ($CDCl_3$ 376 MHz)

1ah

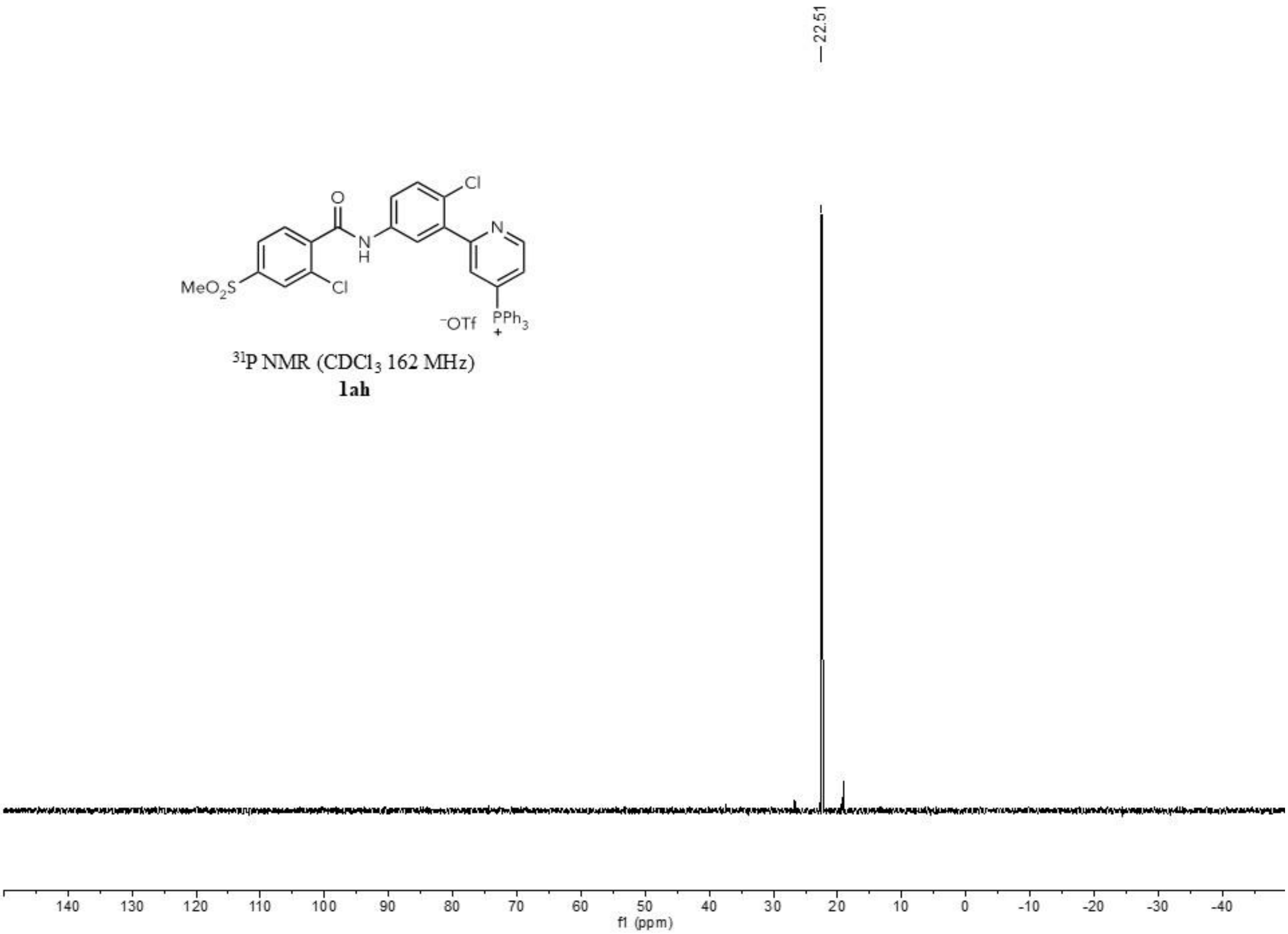

^{31}P NMR ($CDCl_3$ 162 MHz)
1ah

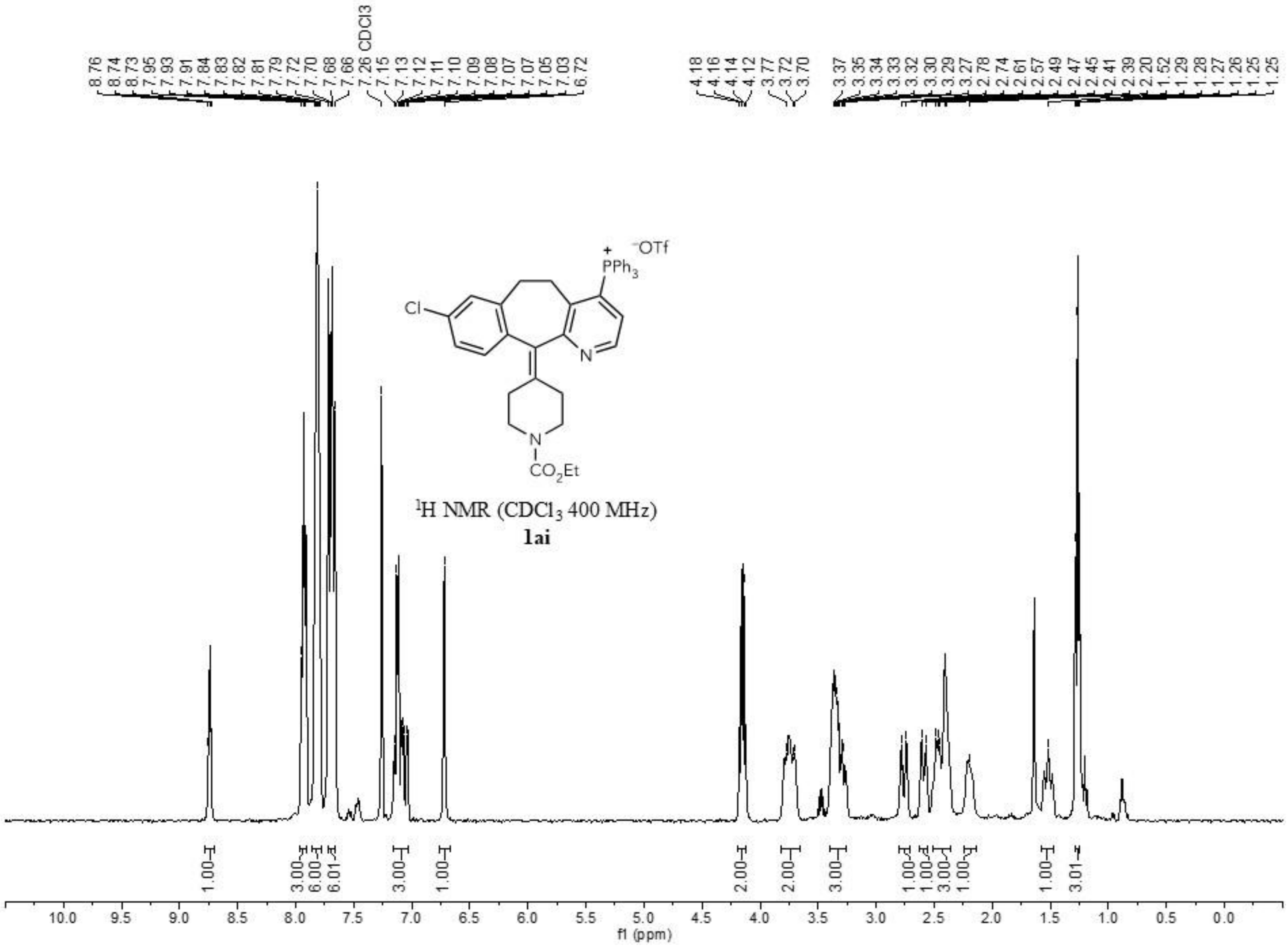

^{1}H NMR ($CDCl_3$ 400 MHz)

1ai

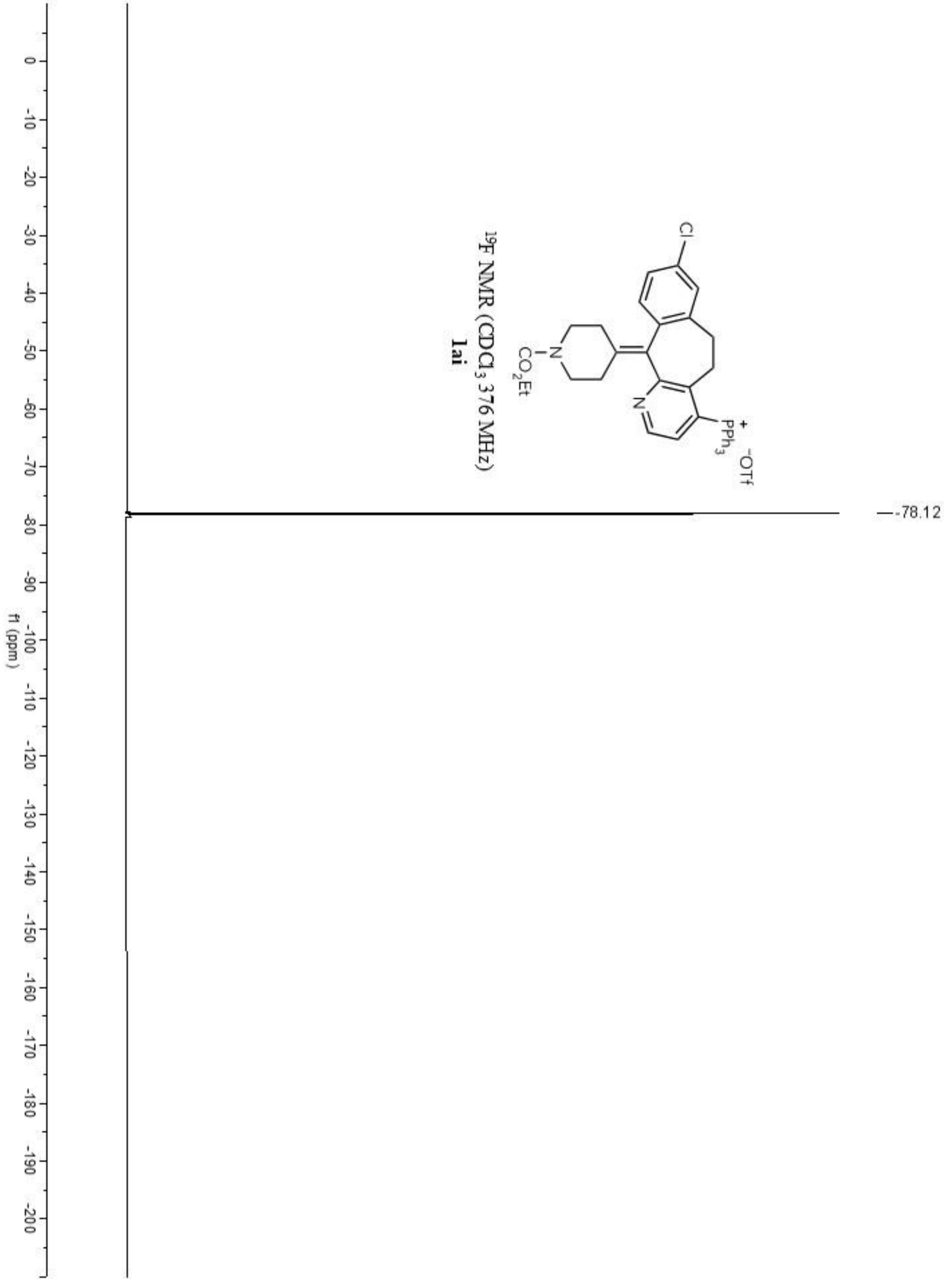
19F NMR (CDCl3 376 MHz)
1ai
Cl
N
CO2Et
PPh3
-OTf
-78.12
fl (ppm)

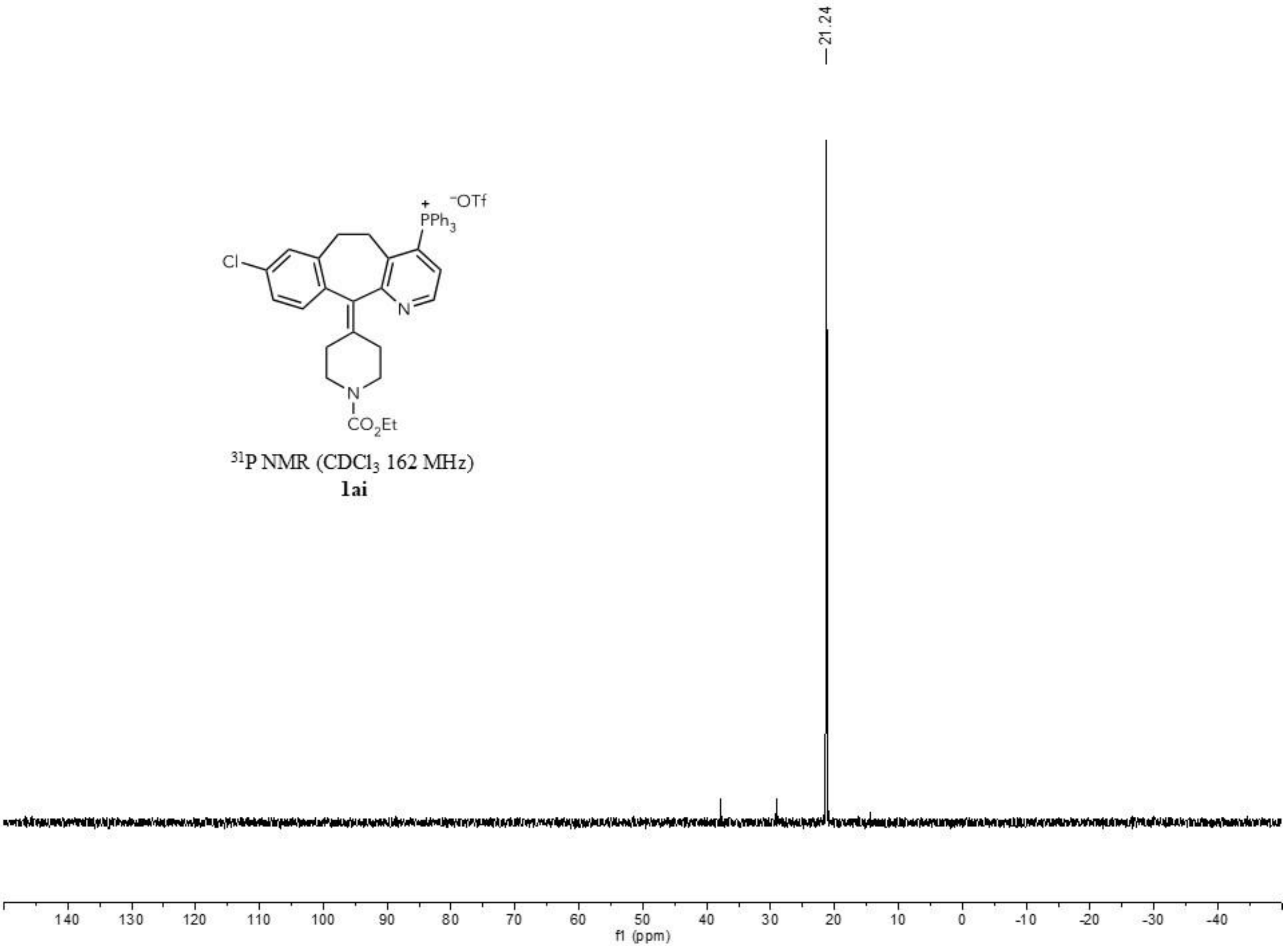

^{31}P NMR ($CDCl_3$ 162 MHz)
1ai

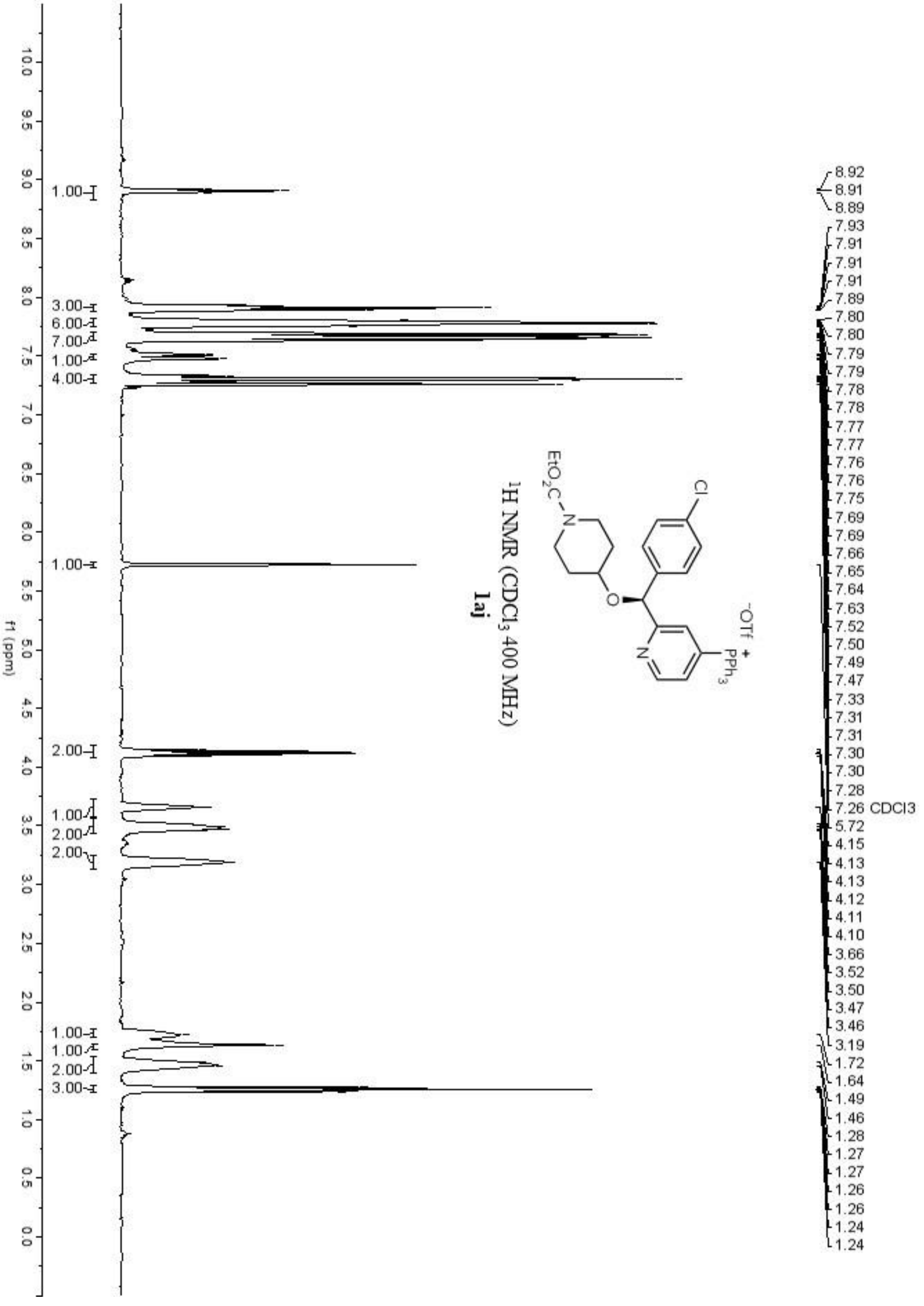

^{1}H NMR (CDCl$_3$ 400 MHz)
1aj
$^-$OTf
$^+$PPh$_3$
Cl
EtO$_2$C
f1 (ppm)
7.26 CDCl3

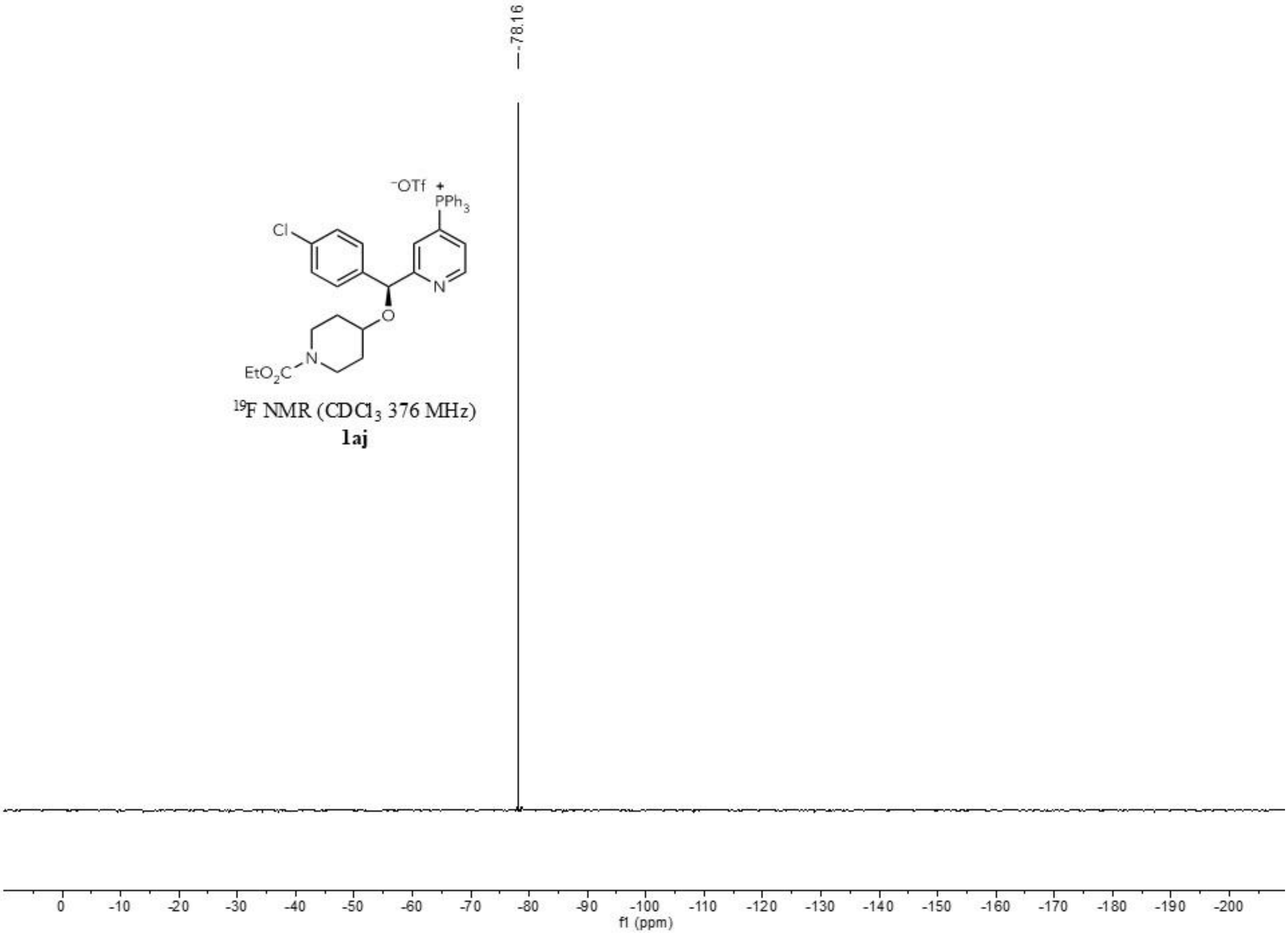
-78.16
-OTf +
PPh3
Cl
N
O
EtO2C
N
19F NMR (CDCl3 376 MHz)
1aj
0 -10 -20 -30 -40 -50 -60 -70 -80 -90 -100 -110 -120 -130 -140 -150 -160 -170 -180 -190 -200
f1 (ppm)

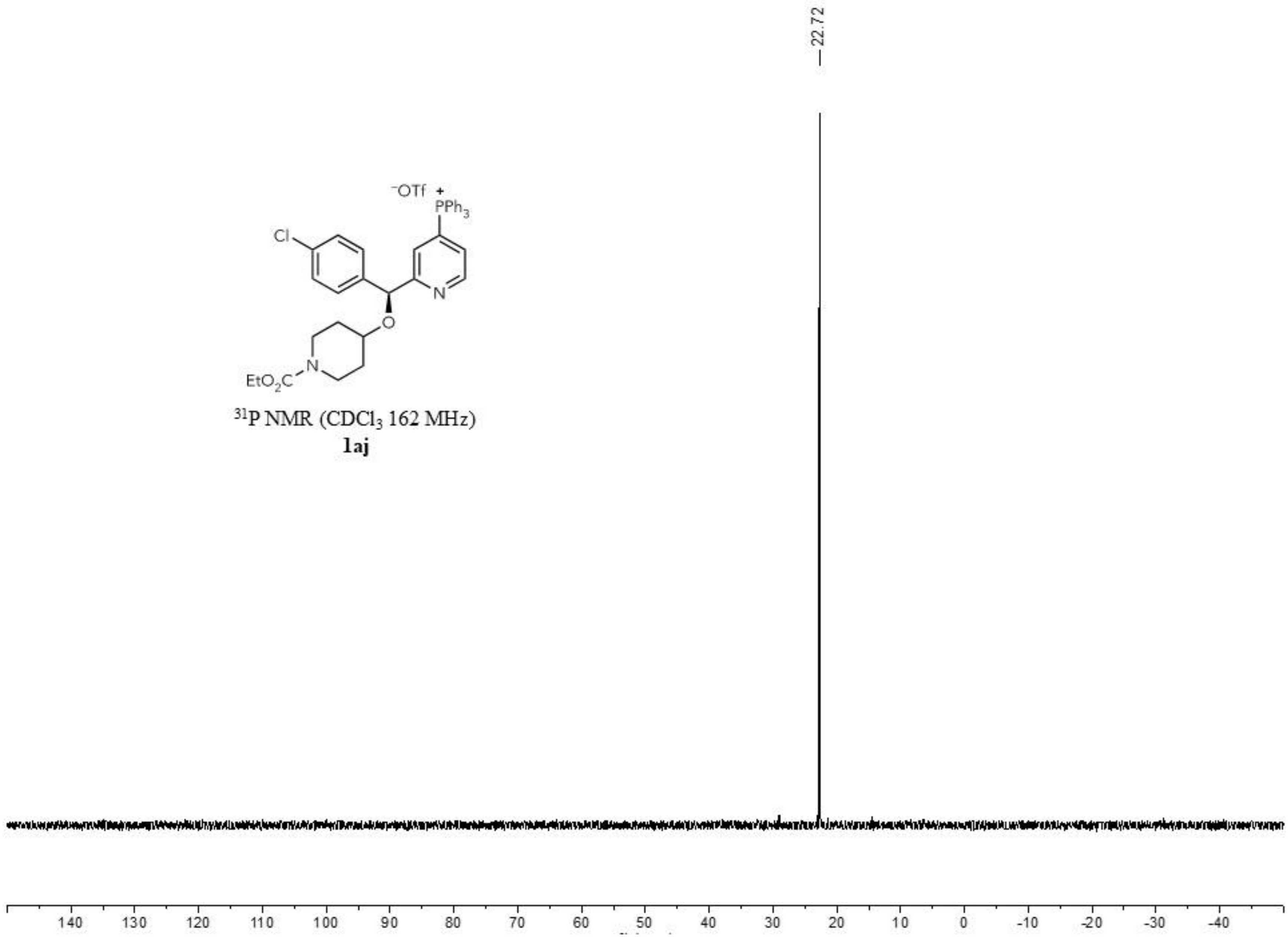

^{31}P NMR ($CDCl_3$ 162 MHz)
1aj

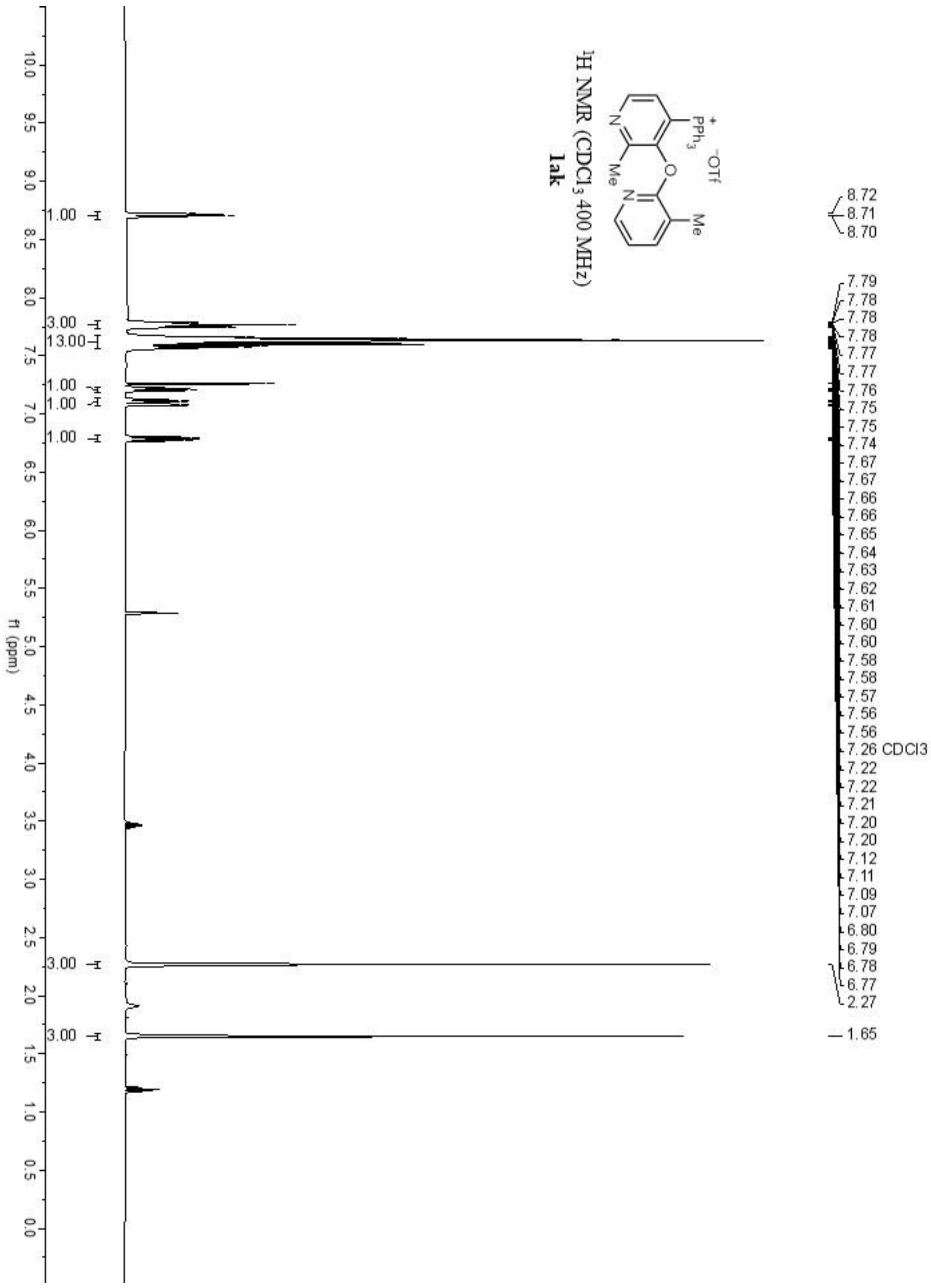
1H NMR (CDCl3 400 MHz)
1ak
PPh3
OTf
Me
N
O
8.72
8.71
8.70
7.79
7.78
7.78
7.78
7.77
7.77
7.76
7.75
7.75
7.74
7.67
7.67
7.66
7.66
7.65
7.64
7.63
7.62
7.61
7.60
7.60
7.58
7.58
7.57
7.56
7.56
7.26 CDCl3
7.22
7.22
7.21
7.20
7.20
7.12
7.11
7.09
7.07
6.80
6.79
6.78
6.77
2.27
1.65
1.00
3.00
13.00
1.00
1.00
1.00
3.00
3.00
f1 (ppm)

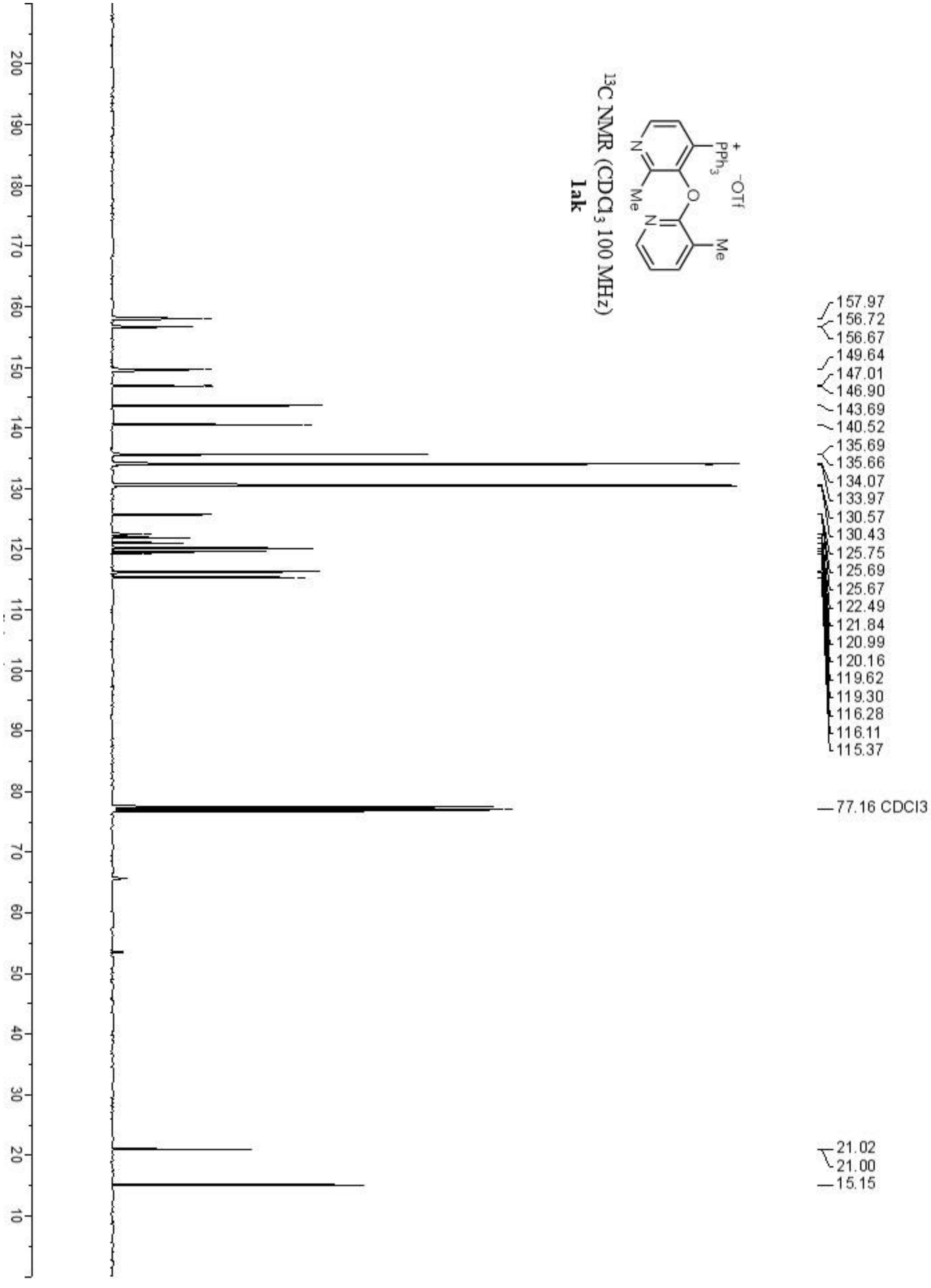
13C NMR (CDCl3 100 MHz)
1ak
PPh3
OTf
Me
N
O
157.97
156.72
156.67
149.64
147.01
146.90
143.69
140.52
135.69
135.66
134.07
133.97
130.57
130.43
125.75
125.69
125.67
122.49
121.84
120.99
120.16
119.62
119.30
116.28
116.11
115.37
77.16 CDCl3
21.02
21.00
15.15
200
190
180
170
160
150
140
130
120
110
100
90
80
70
60
50
40
30
20
10

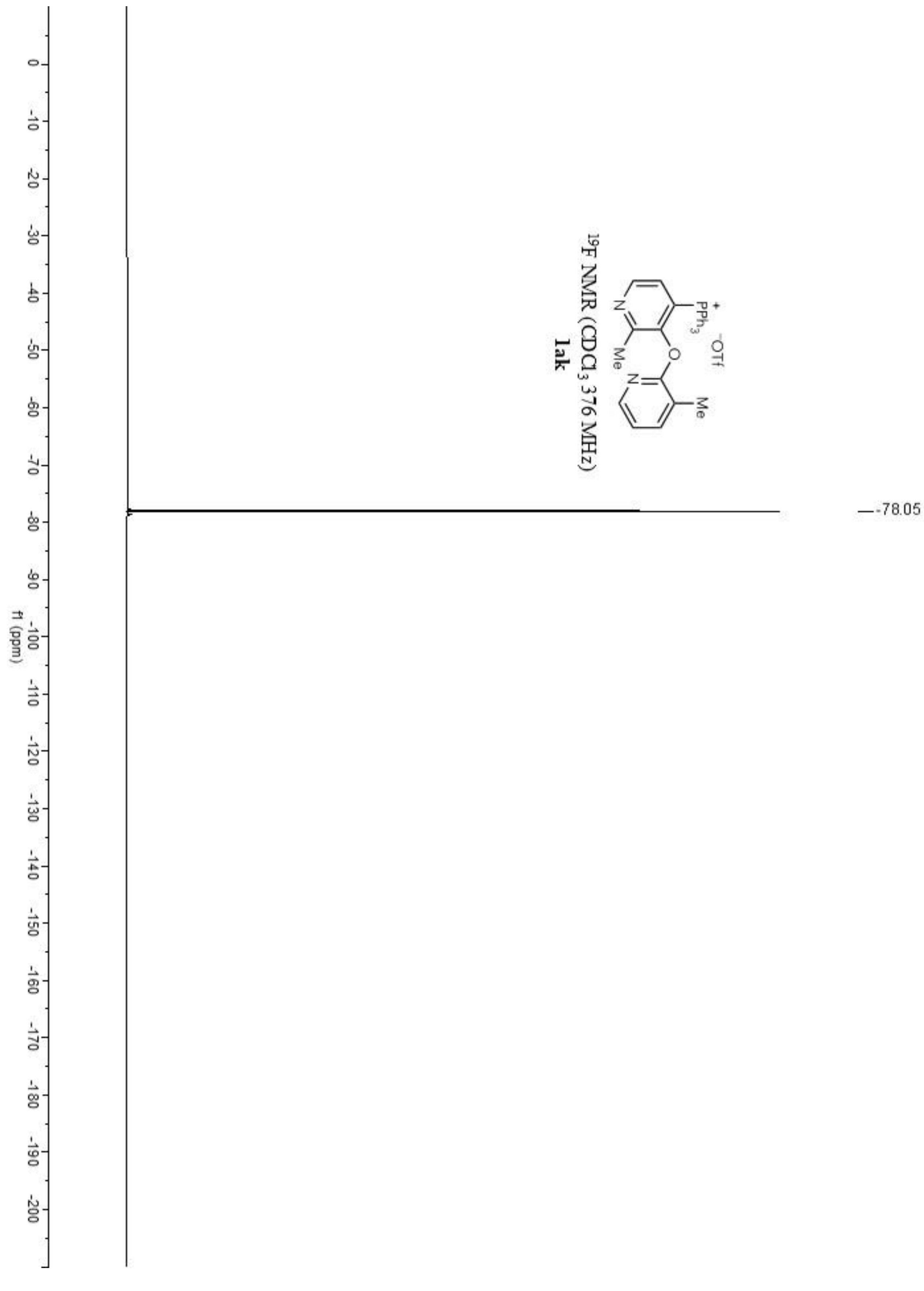

^{19}F NMR (CDCl$_3$ 376 MHz)
1ak
PPh$_3$
$^{-}$OTf
Me
Me
N
N
O
-78.05
fl (ppm)

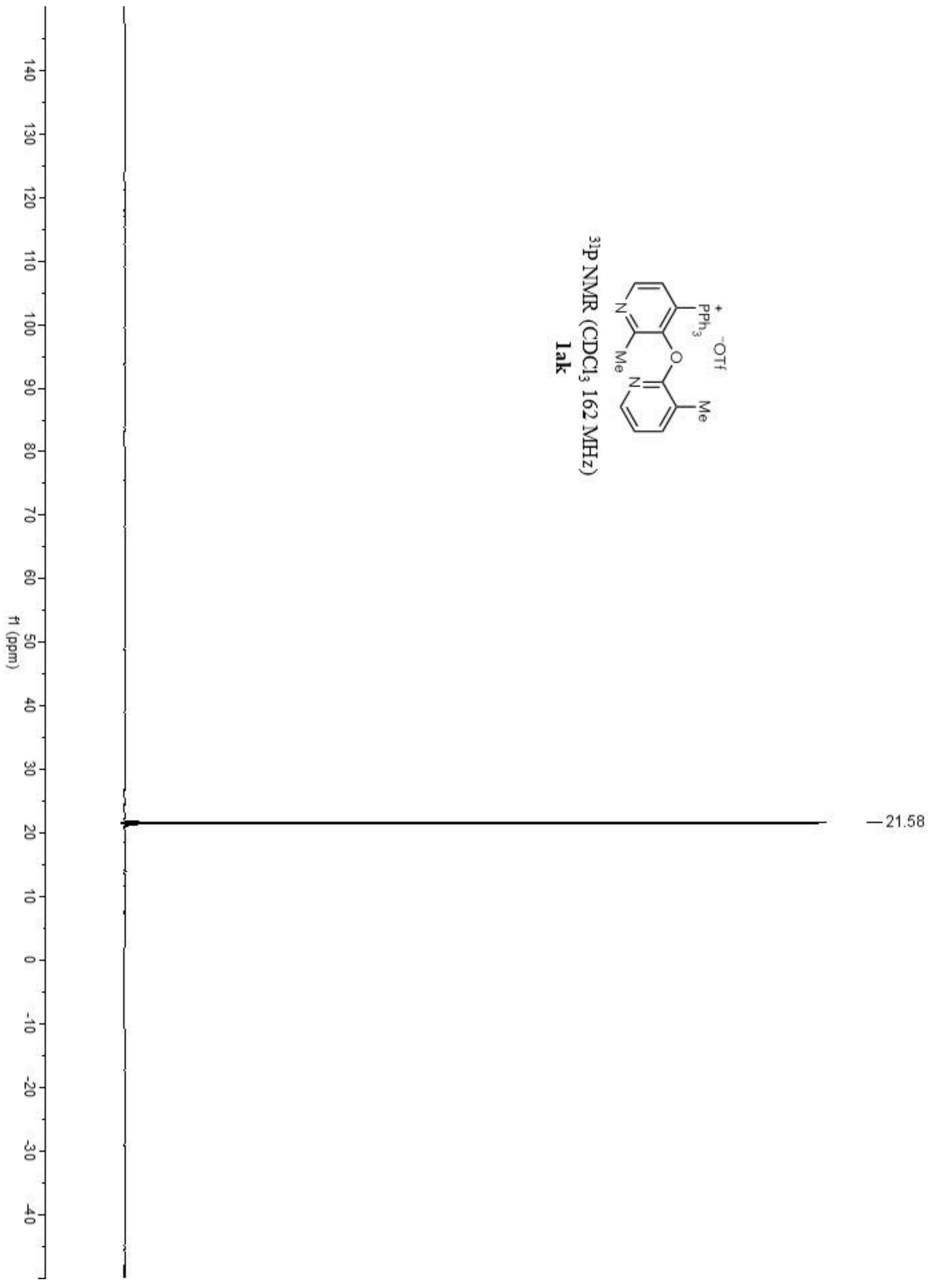
³¹P NMR (CDCl₃ 162 MHz)
1ak
PPh₃
⁻OTf
Me
N
O
—21.58
fl (ppm)
140
130
120
110
100
90
80
70
60
50
40
30
20
10
0
-10
-20
-30
-40

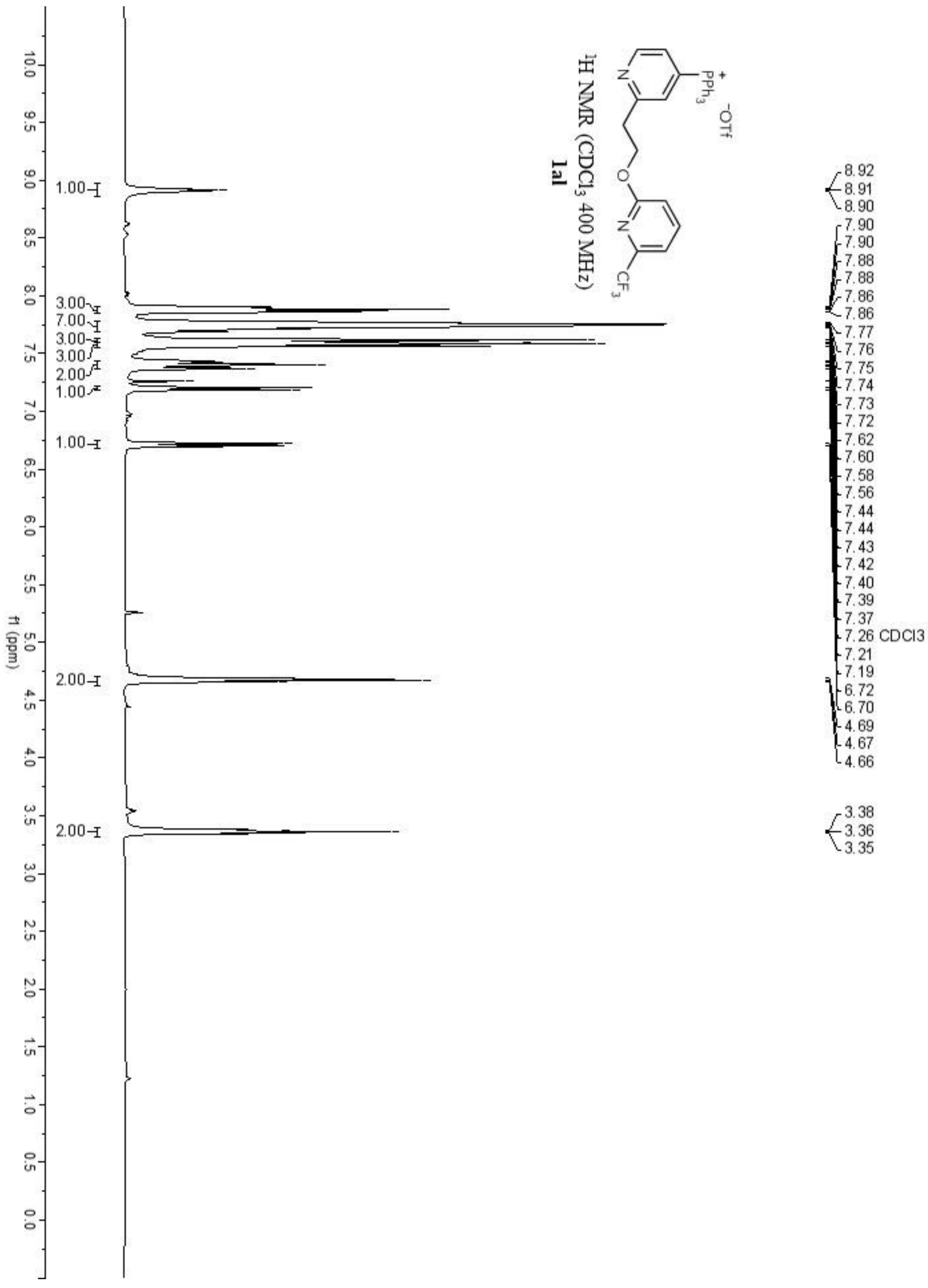

¹H NMR (CDCl3 400 MHz)
1al
PPh3
OTf
CF3
8.92
8.91
8.90
7.90
7.90
7.88
7.88
7.86
7.86
7.77
7.76
7.75
7.74
7.73
7.72
7.62
7.60
7.58
7.56
7.44
7.44
7.43
7.42
7.40
7.39
7.37
7.26 CDCl3
7.21
7.19
6.72
6.70
4.69
4.67
4.66
3.38
3.36
3.35
f1 (ppm)

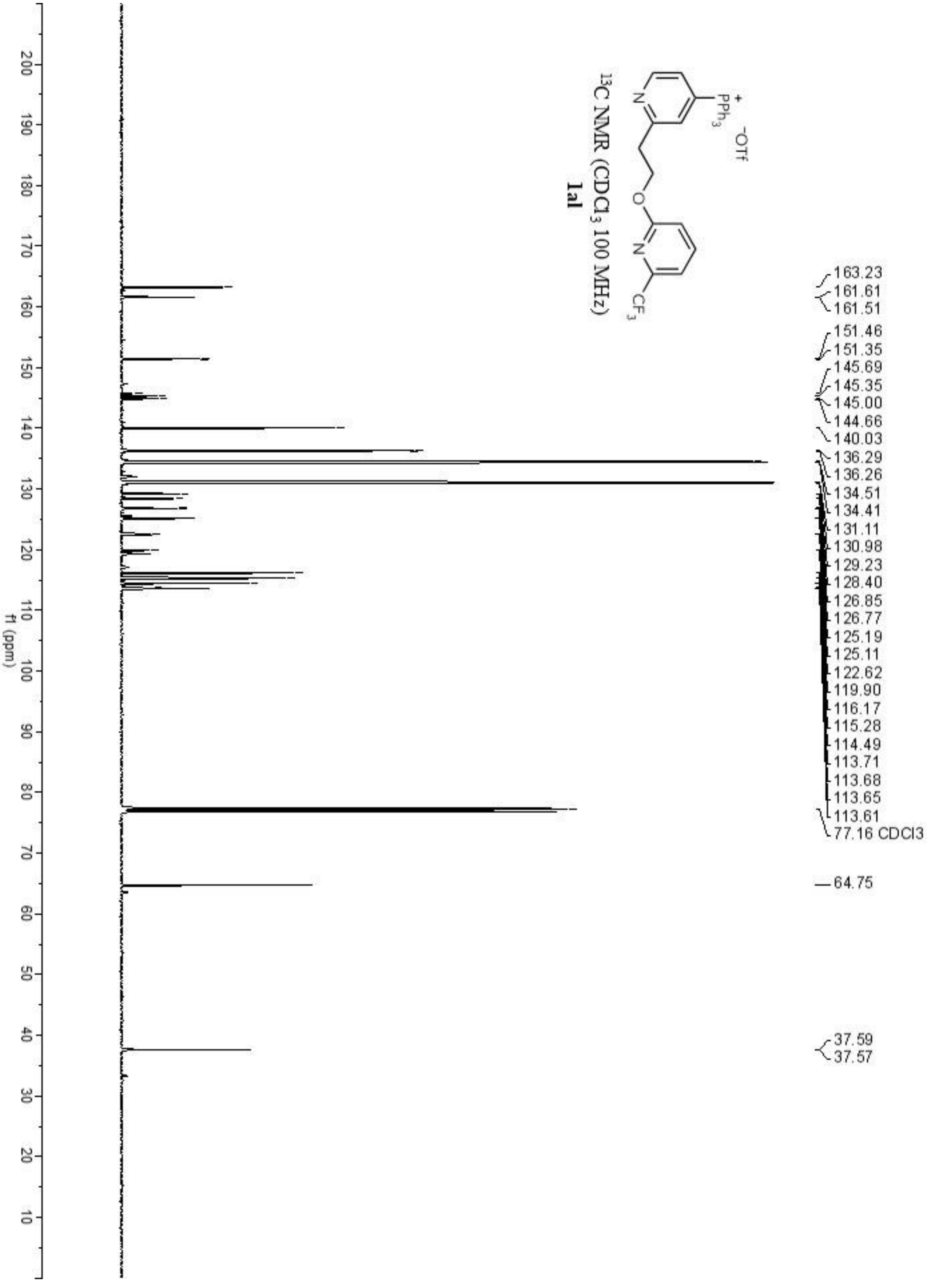

13C NMR (CDCl3 100 MHz)
1al
PPh3
-OTf
N
O
CF3
163.23
161.61
161.51
151.46
151.35
145.69
145.35
145.00
144.66
140.03
136.29
136.26
134.51
134.41
131.11
130.98
129.23
128.40
126.85
126.77
125.19
125.11
122.62
119.90
116.17
115.28
114.49
113.71
113.68
113.65
113.61
77.16 CDCl3
64.75
37.59
37.57
f1 (ppm)

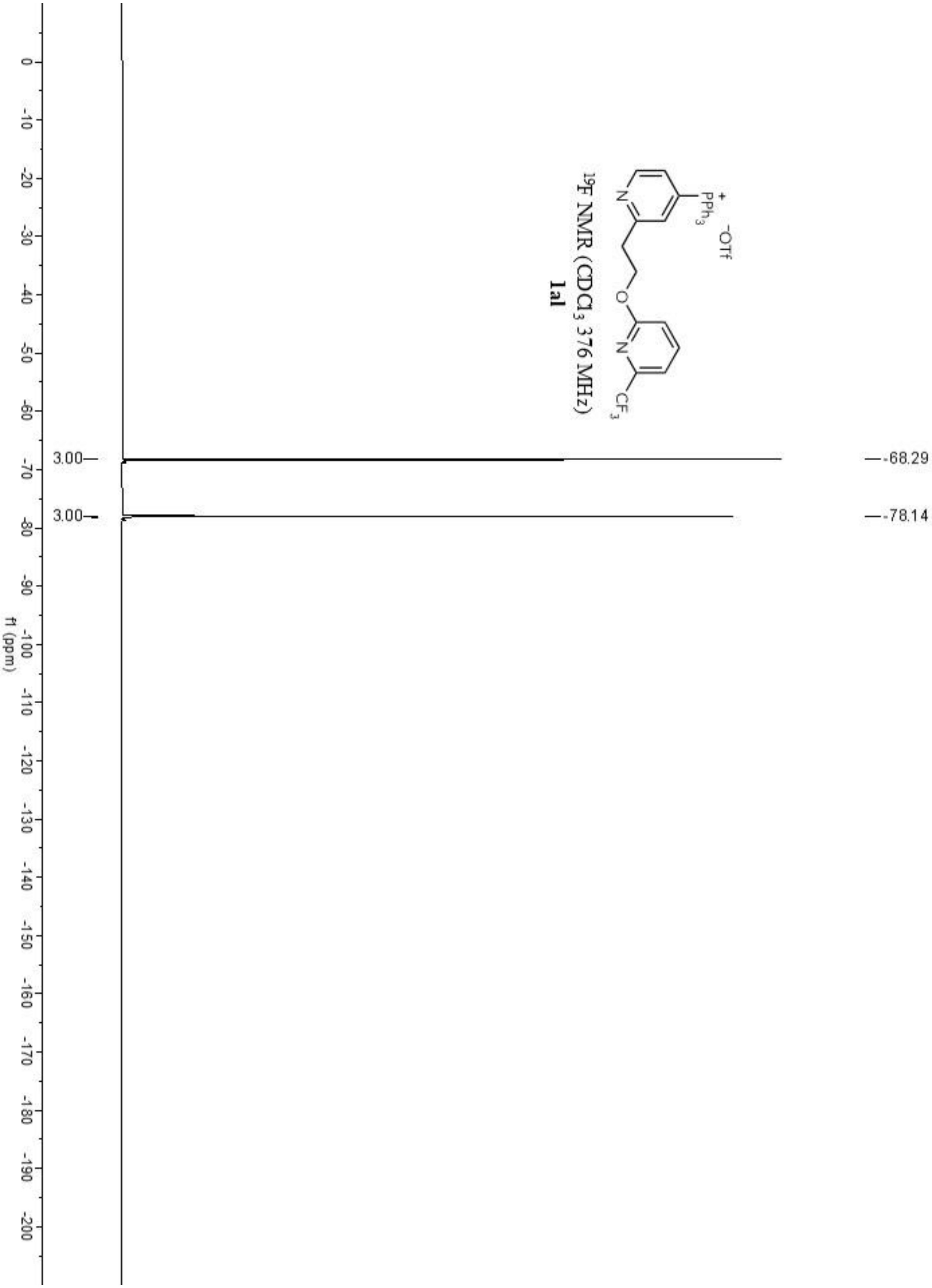

^{19}F NMR (CDCl$_3$ 376 MHz)
1al
+PPh3
-OTf
CF3
-68.29
-78.14
3.00
3.00
f1 (ppm)

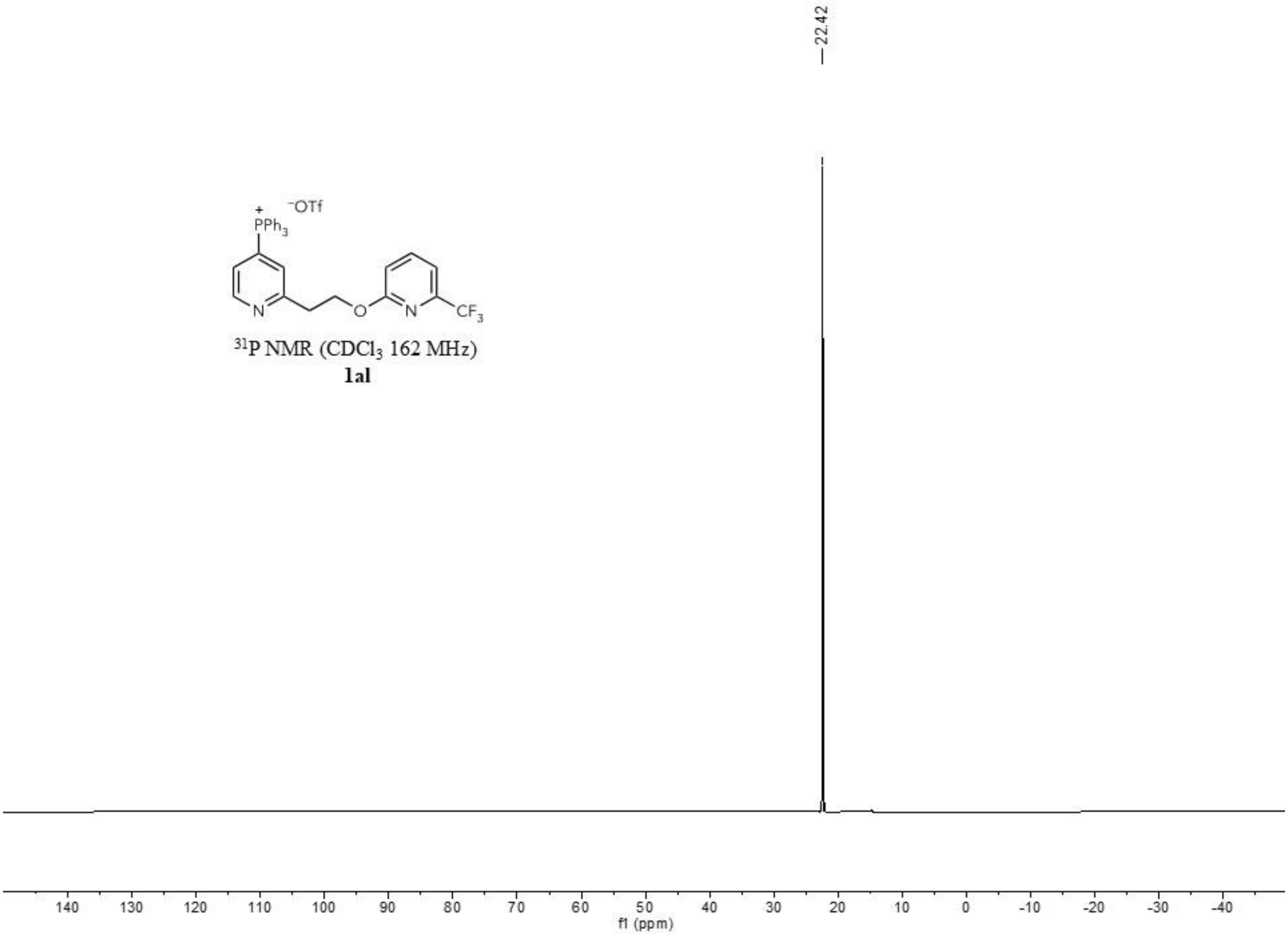

^{31}P NMR ($CDCl_3$ 162 MHz)
1al

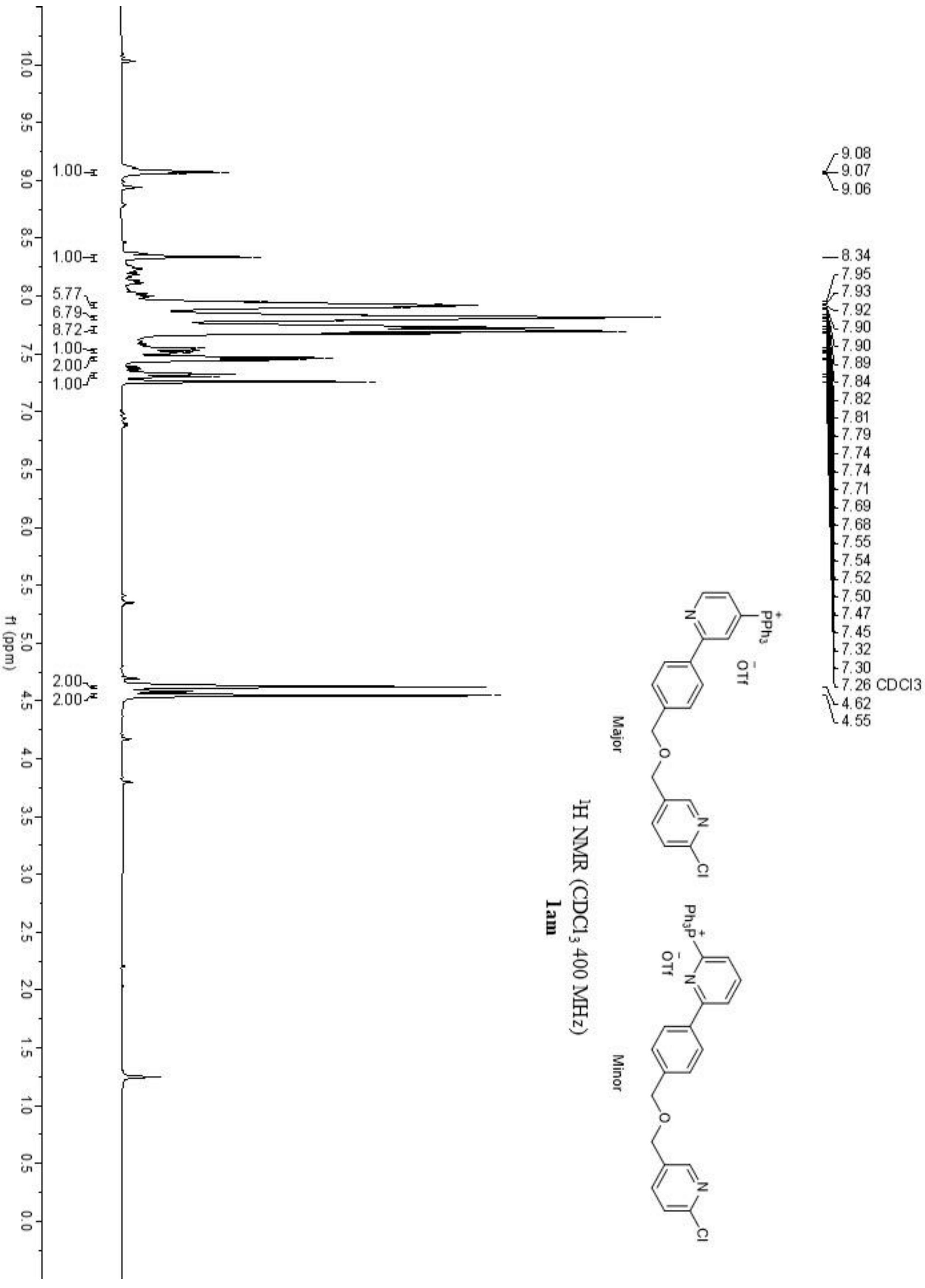

^{1}H NMR ($CDCl_3$ 400 MHz)

1am

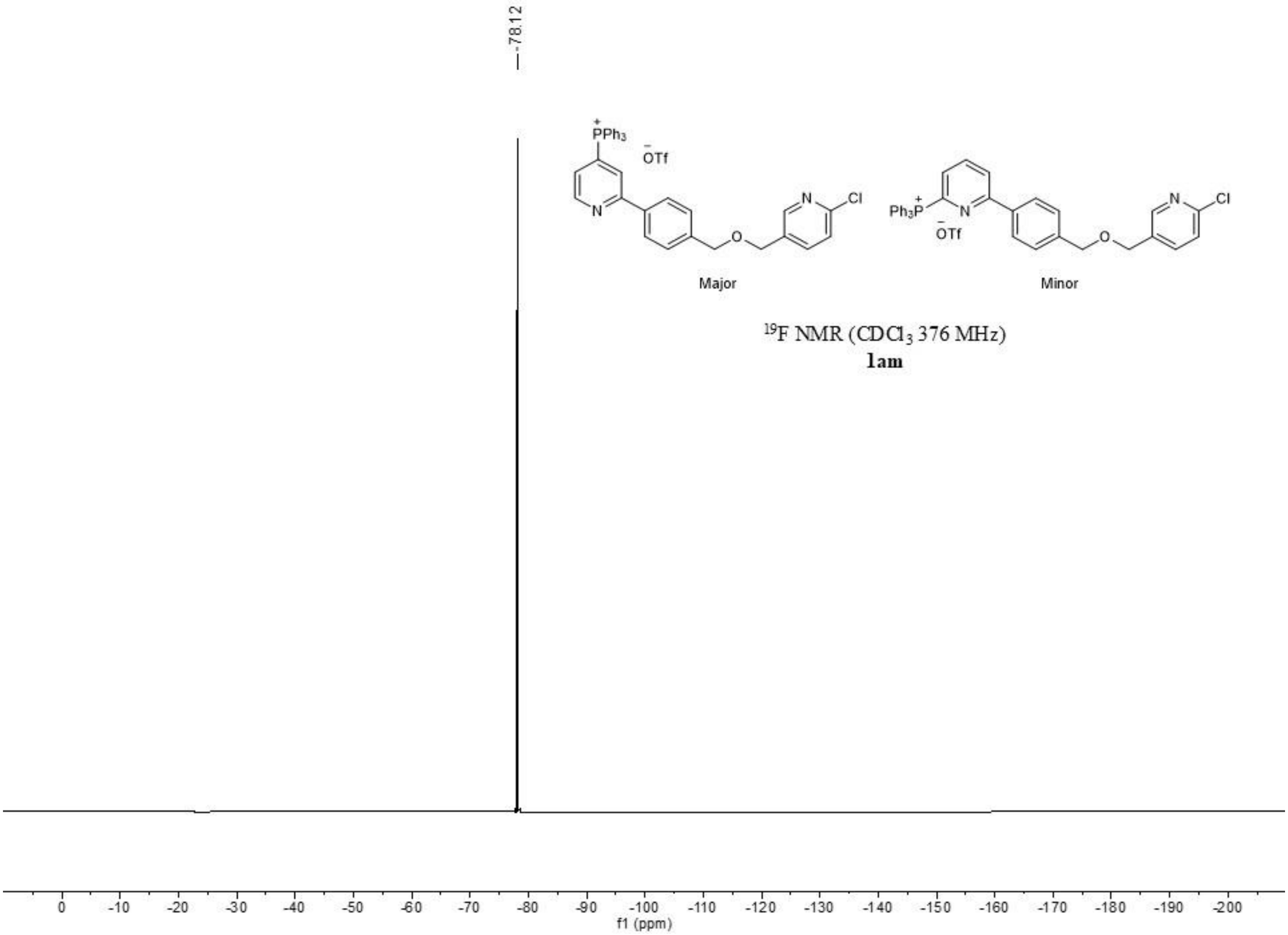

-78.12
PPh3
OTf
N
N
Cl
Major
Ph3P
OTf
N
N
Cl
Minor
19F NMR (CDCl3 376 MHz)
1am
0 -10 -20 -30 -40 -50 -60 -70 -80 -90 -100 -110 -120 -130 -140 -150 -160 -170 -180 -190 -200
f1 (ppm)

^{31}P NMR ($CDCl_3$ 162 MHz)

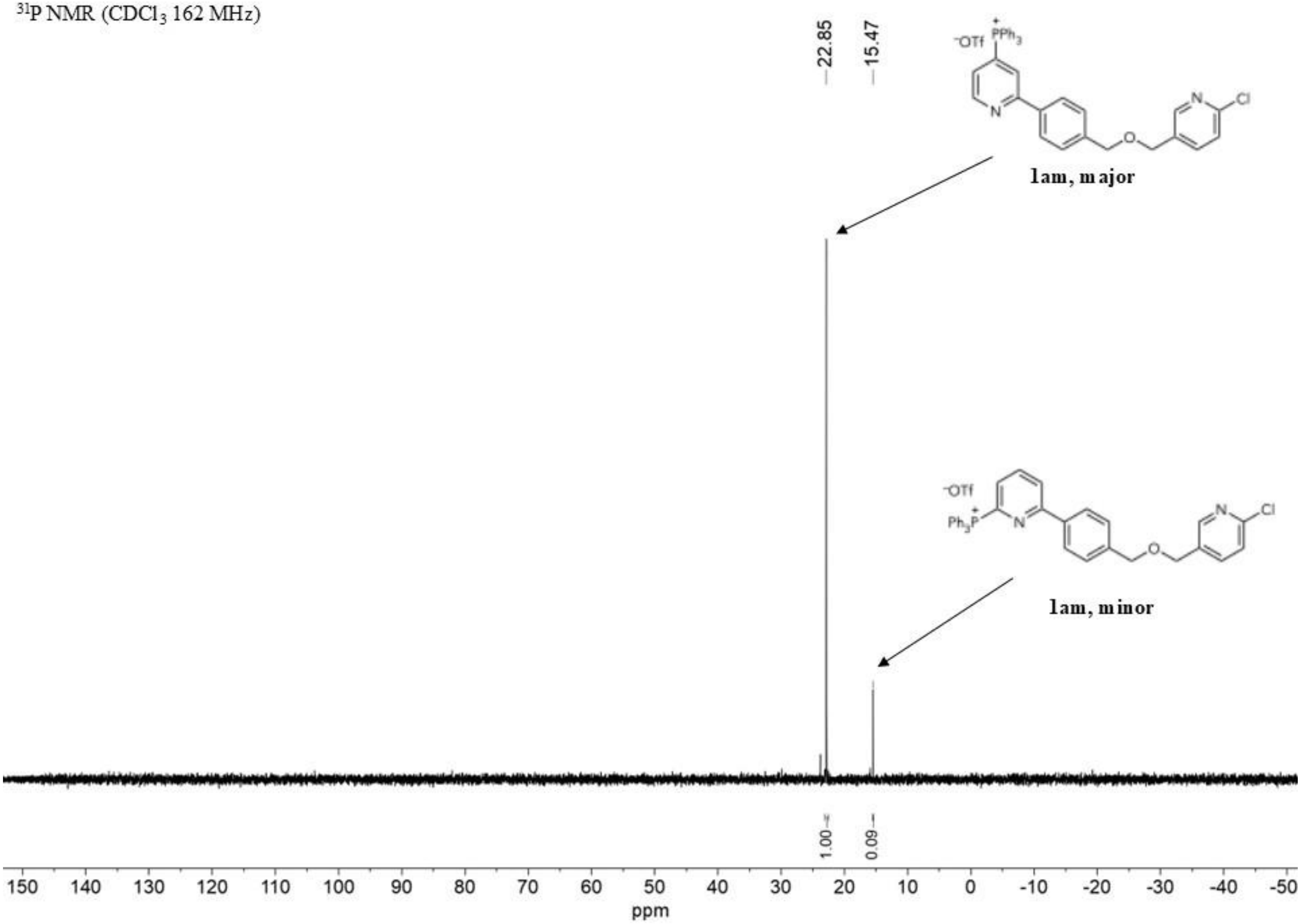

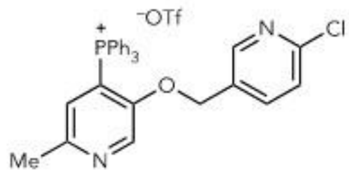

^{1}H NMR ($CDCl_3$ 400 MHz)

1an

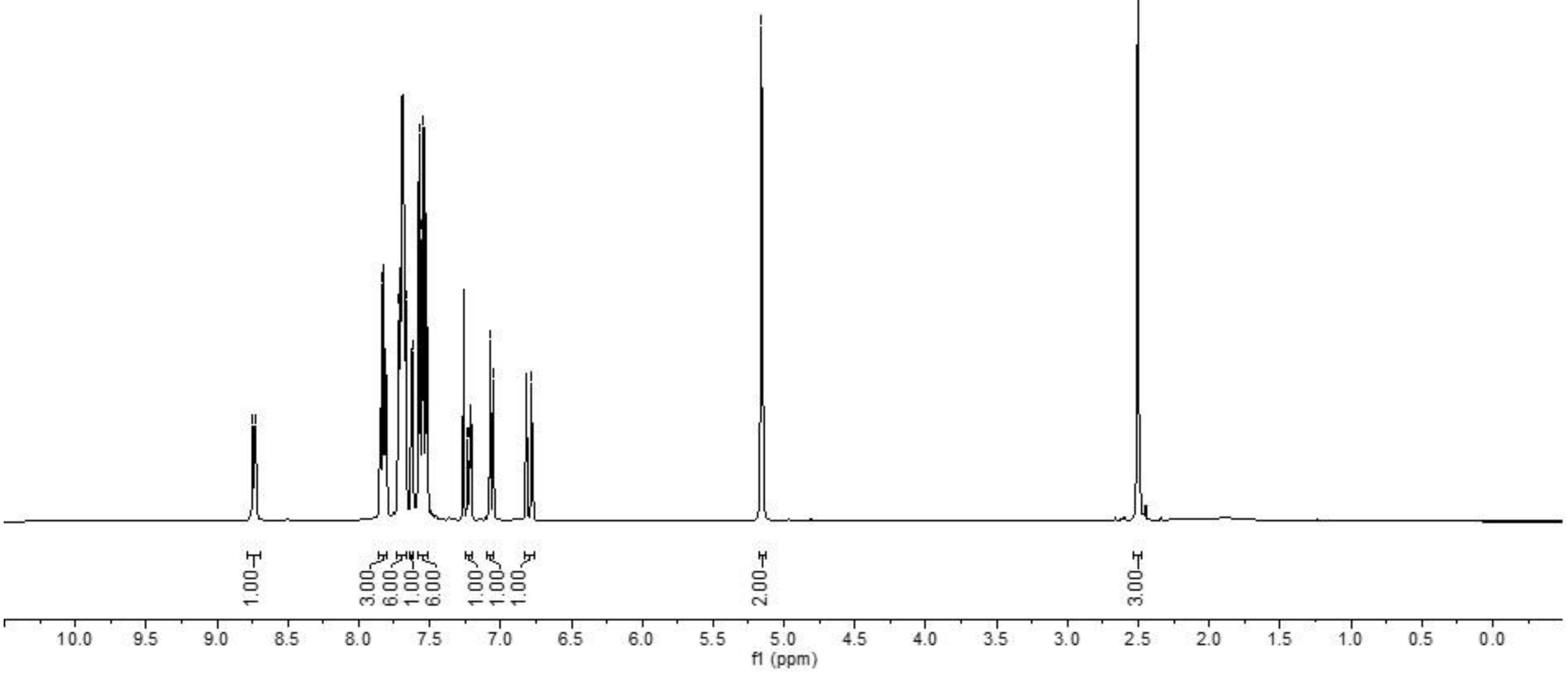

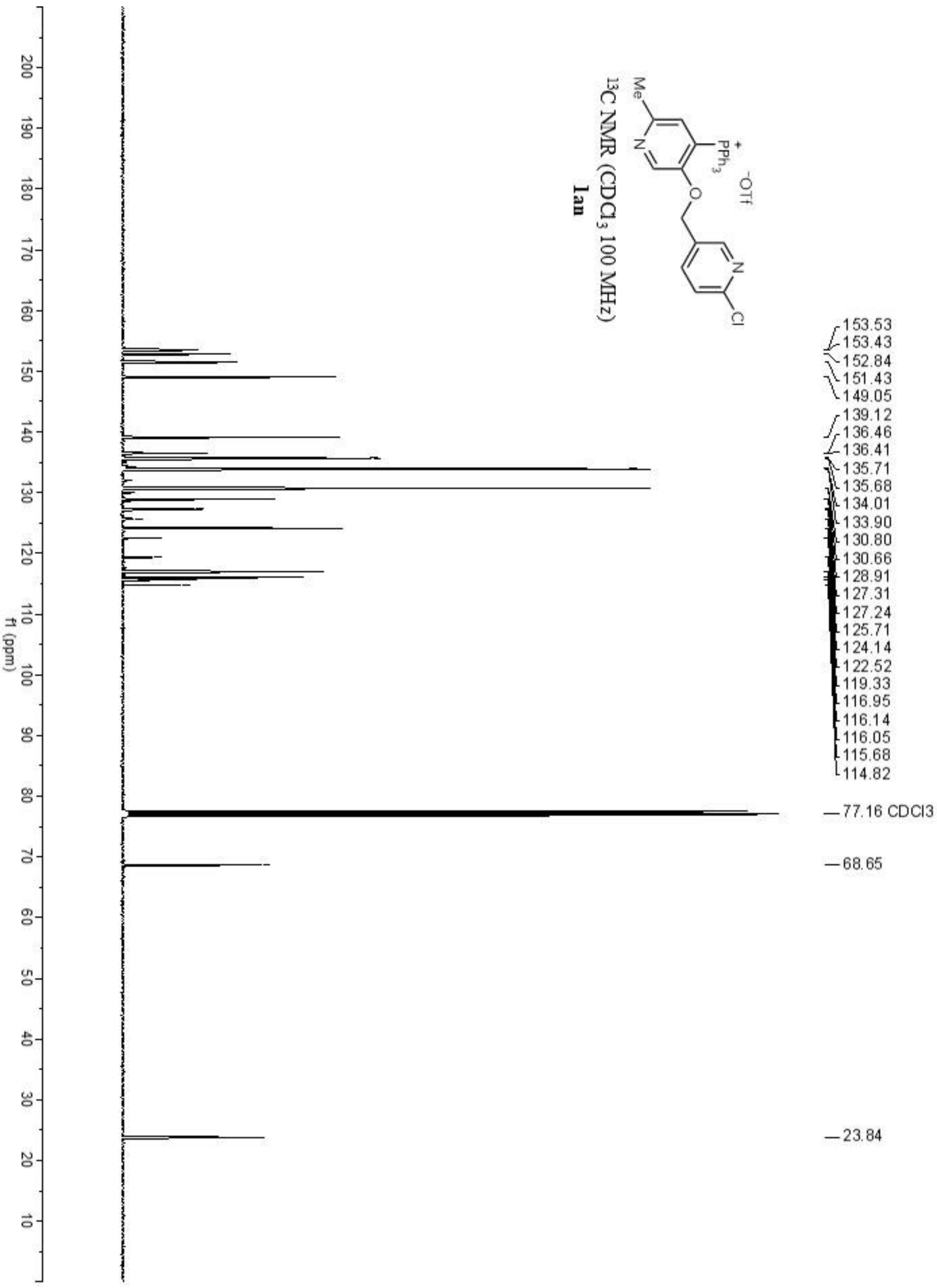

-OTf
PPh3
Me
N
O
N
Cl
13C NMR (CDCl3 100 MHz)
1an
153.53
153.43
152.84
151.43
149.05
139.12
136.46
136.41
135.71
135.68
134.01
133.90
130.80
130.66
128.91
127.31
127.24
125.71
124.14
122.52
119.33
116.95
116.14
116.05
115.68
114.82
77.16 CDCl3
68.65
23.84
200
190
180
170
160
150
140
130
120
110
100
90
80
70
60
50
40
30
20
10
f1 (ppm)

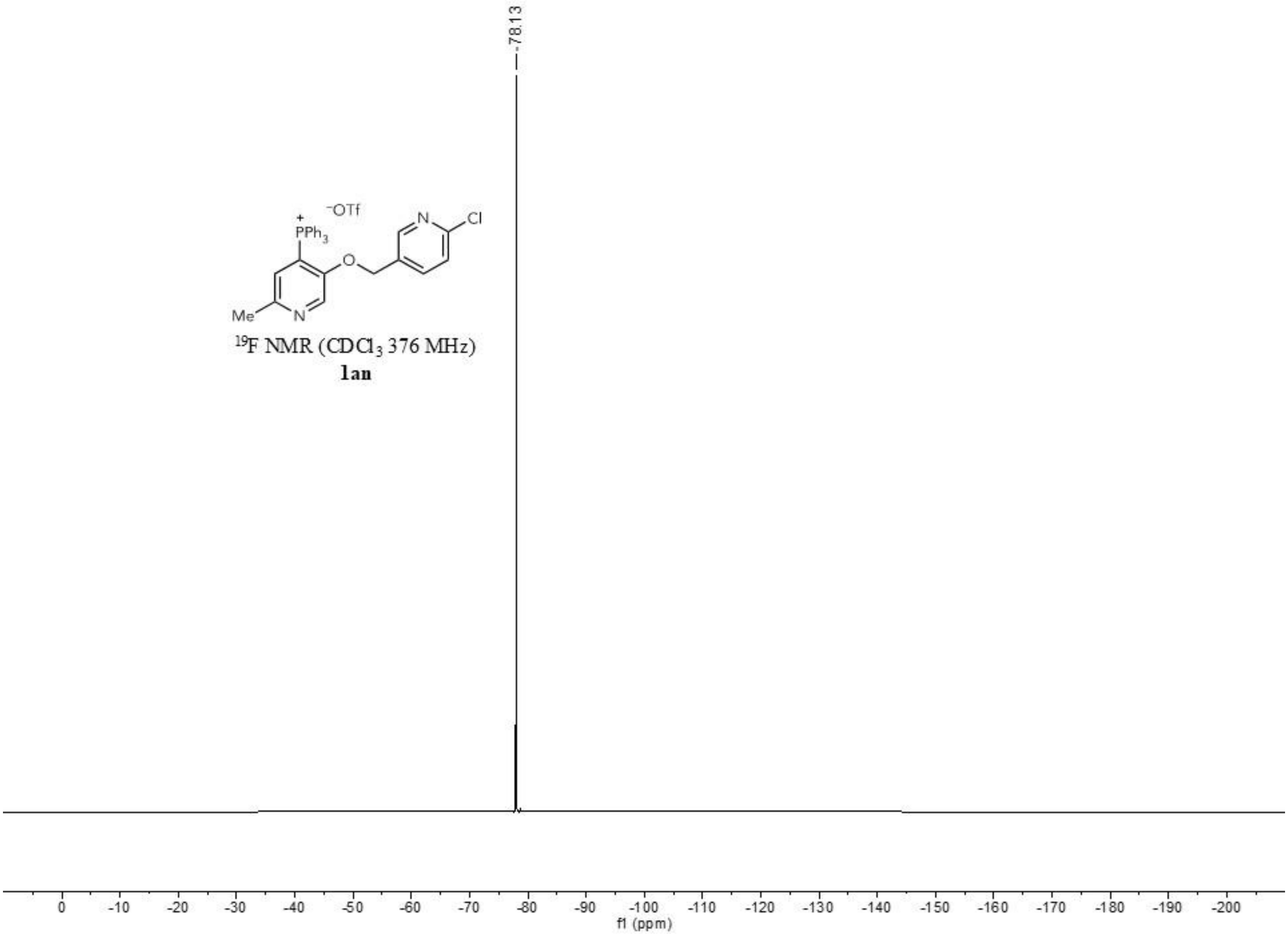

$^{-}$OTf
PPh$_3$
N
Cl
O
Me
N
^{19}F NMR (CDCl$_3$ 376 MHz)
1an
-78.13
0 -10 -20 -30 -40 -50 -60 -70 -80 -90 -100 -110 -120 -130 -140 -150 -160 -170 -180 -190 -200
f1 (ppm)

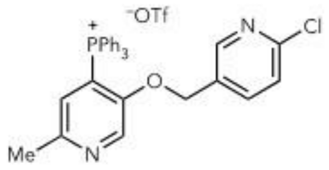

^{31}P NMR (CDCl$_3$ 162 MHz)
1an

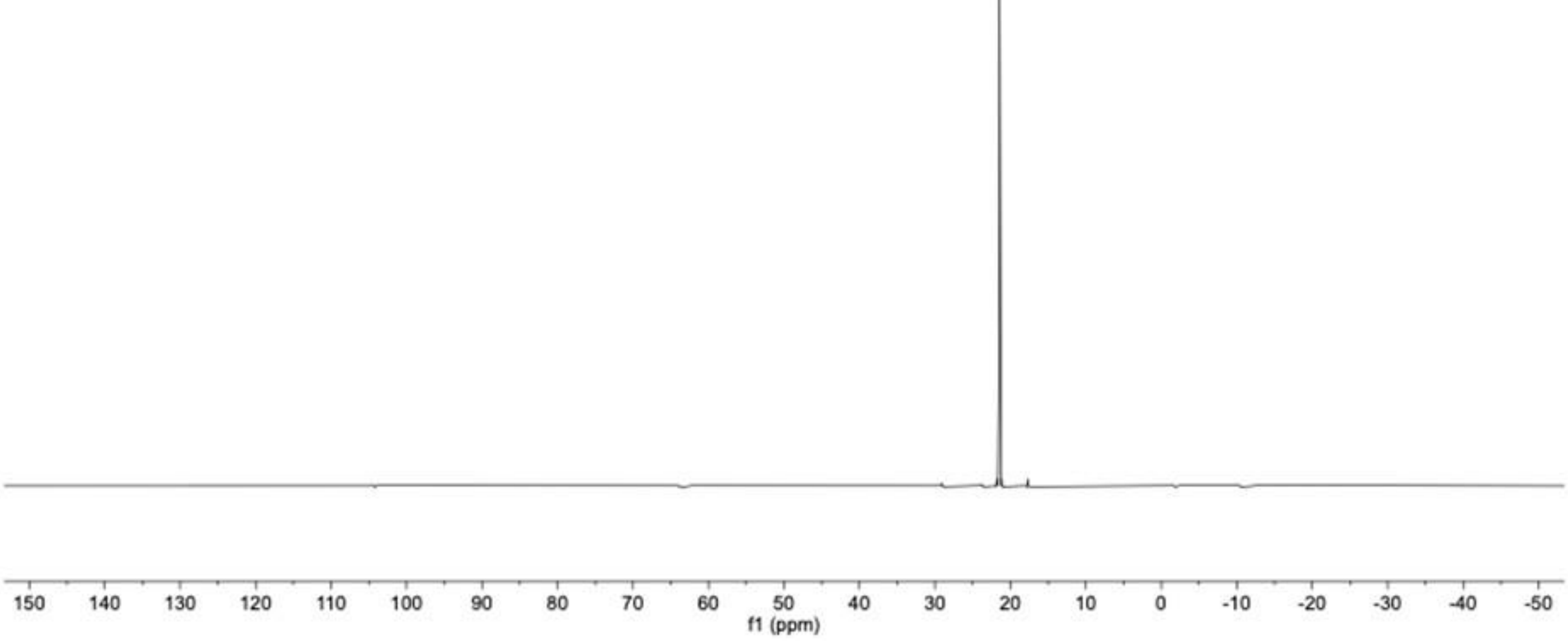

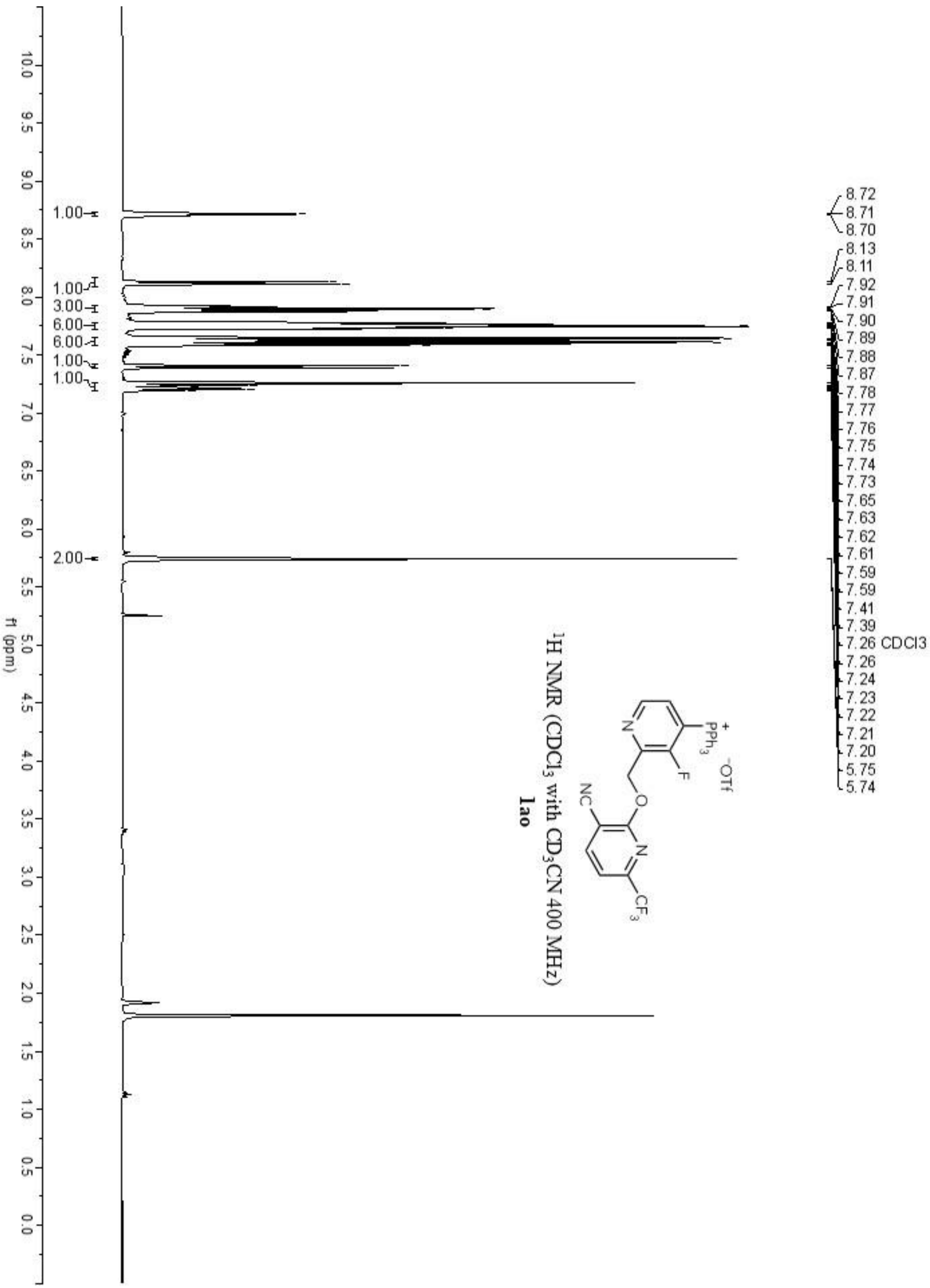

¹H NMR (CDCl₃ with CD₃CN 400 MHz)
1ao
8.72
8.71
8.70
8.13
8.11
7.92
7.91
7.90
7.89
7.88
7.87
7.78
7.77
7.76
7.75
7.74
7.73
7.65
7.63
7.62
7.61
7.59
7.59
7.41
7.39
7.26 CDCl3
7.26
7.24
7.23
7.22
7.21
7.20
5.75
5.74
f1 (ppm)

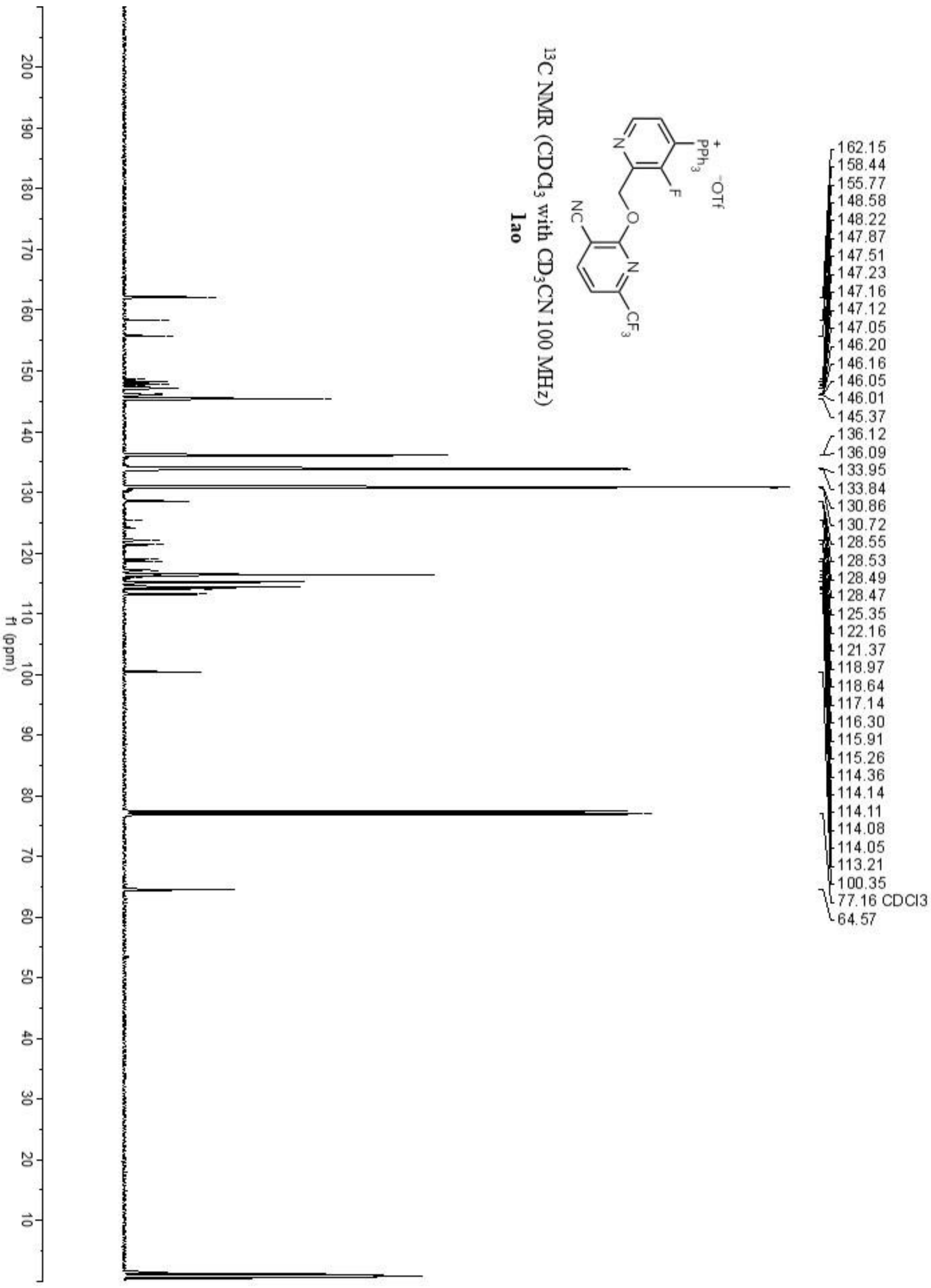

PPh3
-OTf
F
N
O
NC
N
CF3
13C NMR (CDCl3 with CD3CN 100 MHz)
1ao
162.15
158.44
155.77
148.58
148.22
147.87
147.51
147.23
147.16
147.12
147.05
146.20
146.16
146.05
146.01
145.37
136.12
136.09
133.95
133.84
130.86
130.72
128.55
128.53
128.49
128.47
125.35
122.16
121.37
118.97
118.64
117.14
116.30
115.91
115.26
114.36
114.14
114.11
114.08
114.05
113.21
100.35
77.16 CDCl3
64.57
f1 (ppm)

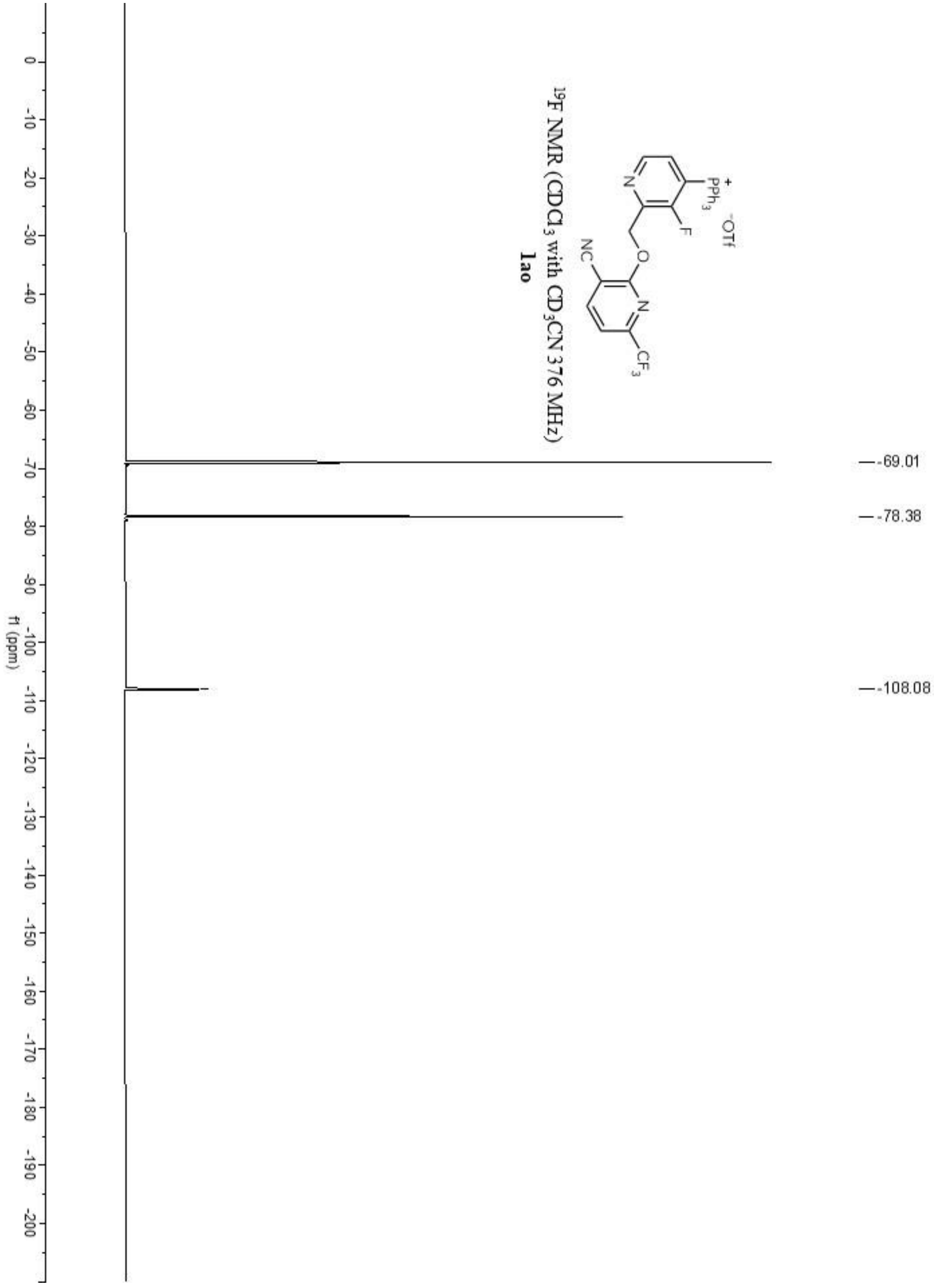
19F NMR (CDCl3 with CD3CN 376 MHz)
1ao
-69.01
-78.38
-108.08
fl (ppm)

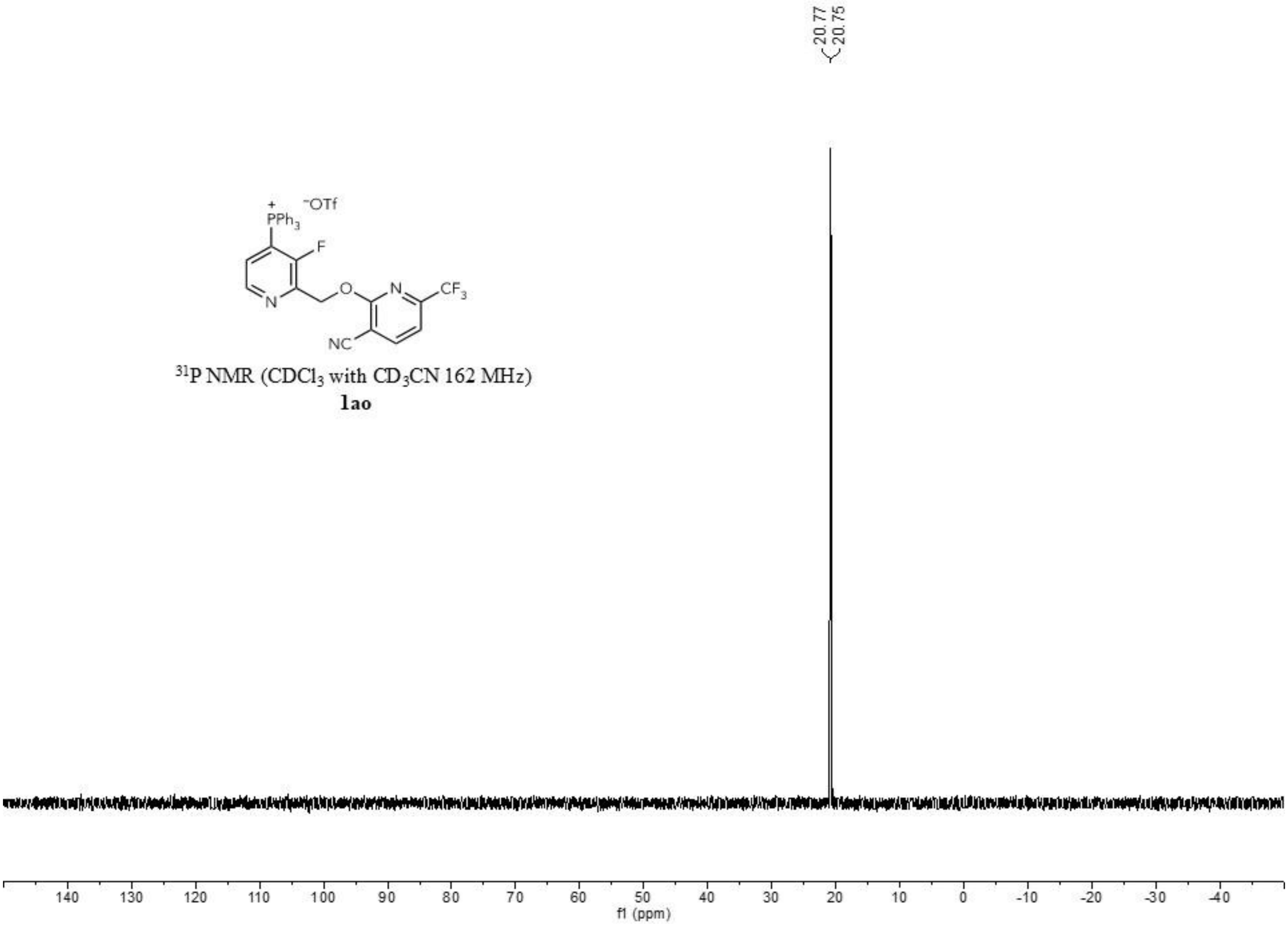

^{31}P NMR ($CDCl_3$ with CD_3CN 162 MHz)
1ao

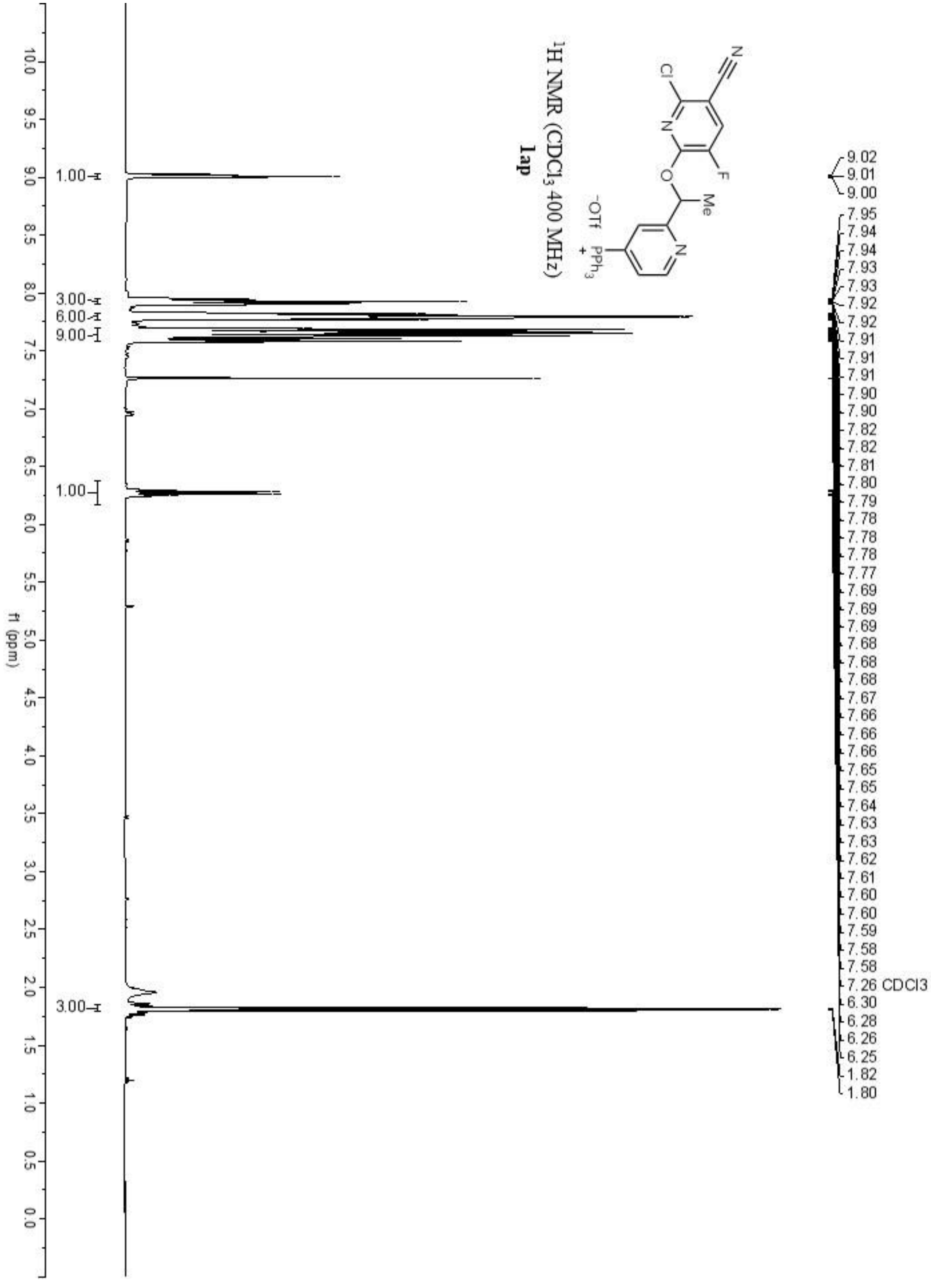
1H NMR (CDCl3 400 MHz)
1ap
-OTf
PPh3
Me
F
Cl
N
9.02
9.01
9.00
7.95
7.94
7.94
7.93
7.93
7.92
7.92
7.91
7.91
7.91
7.90
7.90
7.82
7.82
7.81
7.80
7.79
7.78
7.78
7.78
7.77
7.69
7.69
7.69
7.68
7.68
7.68
7.67
7.66
7.66
7.66
7.65
7.65
7.64
7.63
7.63
7.62
7.61
7.60
7.60
7.59
7.58
7.58
7.26 CDCl3
6.30
6.28
6.26
6.25
1.82
1.80
1.00
3.00
6.00
9.00
1.00
3.00
f1 (ppm)

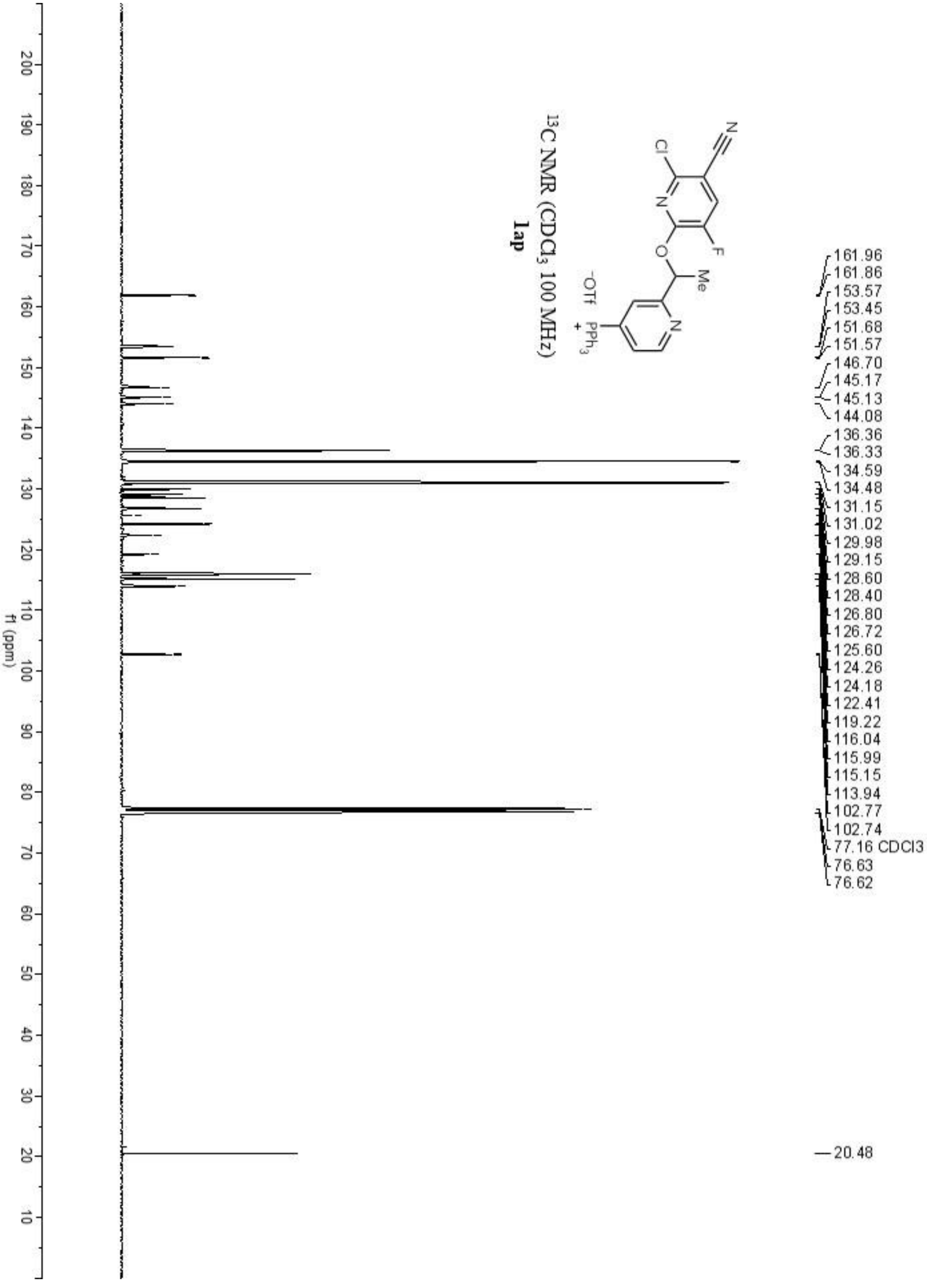
13C NMR (CDCl3 100 MHz)
1ap
N
Cl
N
O
F
Me
N
PPh3
+
-OTf
161.96
161.86
153.57
153.45
151.68
151.57
146.70
145.17
145.13
144.08
136.36
136.33
134.59
134.48
131.15
131.02
129.98
129.15
128.60
128.40
126.80
126.72
125.60
124.26
124.18
122.41
119.22
116.04
115.99
115.15
113.94
102.77
102.74
77.16 CDCl3
76.63
76.62
20.48
f1 (ppm)

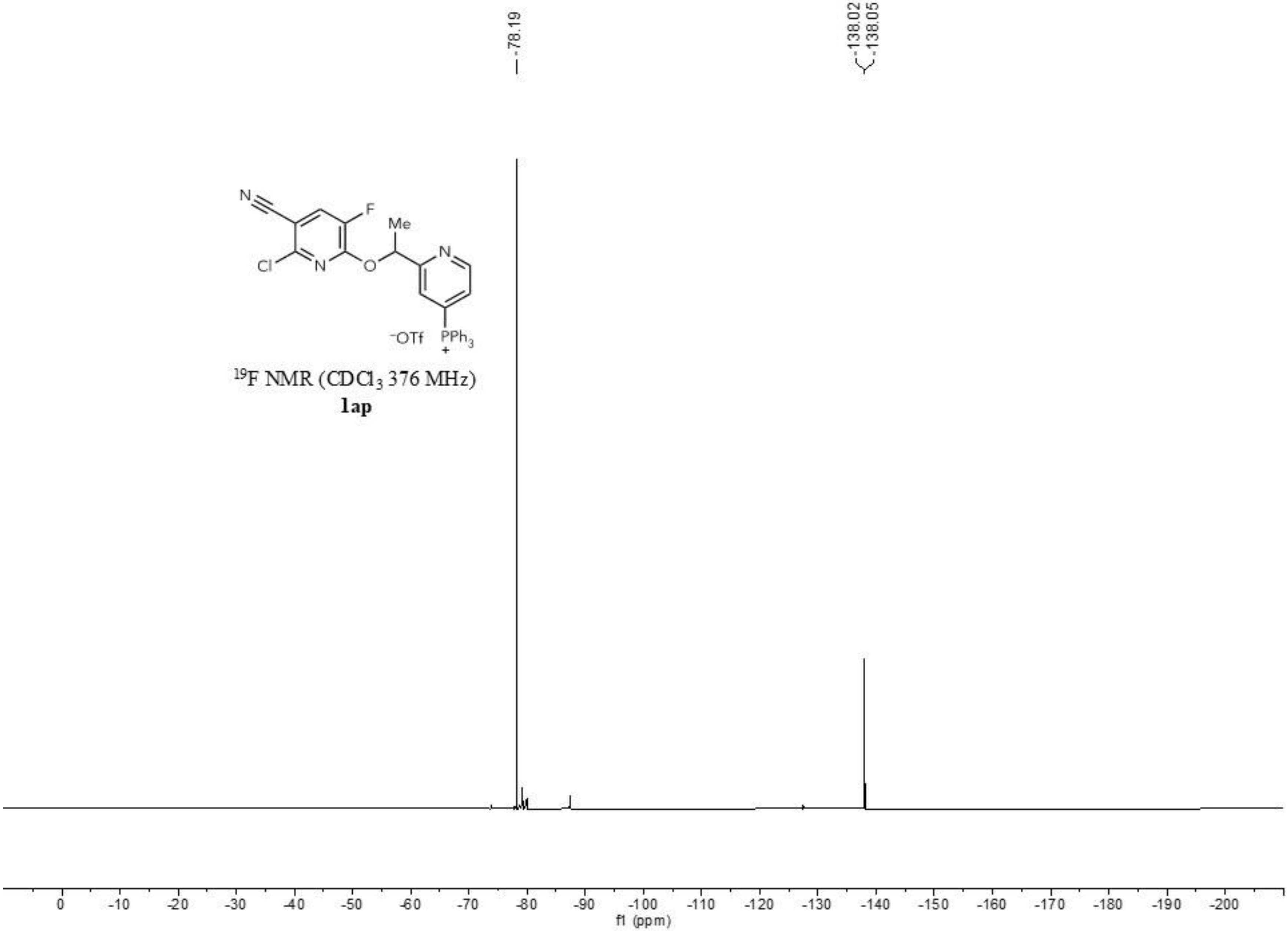

^{19}F NMR ($CDCl_3$ 376 MHz)

1ap

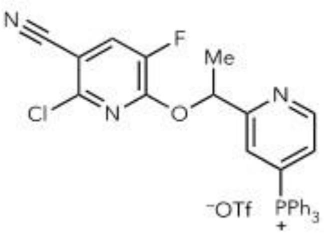

^{31}P NMR ($CDCl_3$ 162 MHz)

1ap

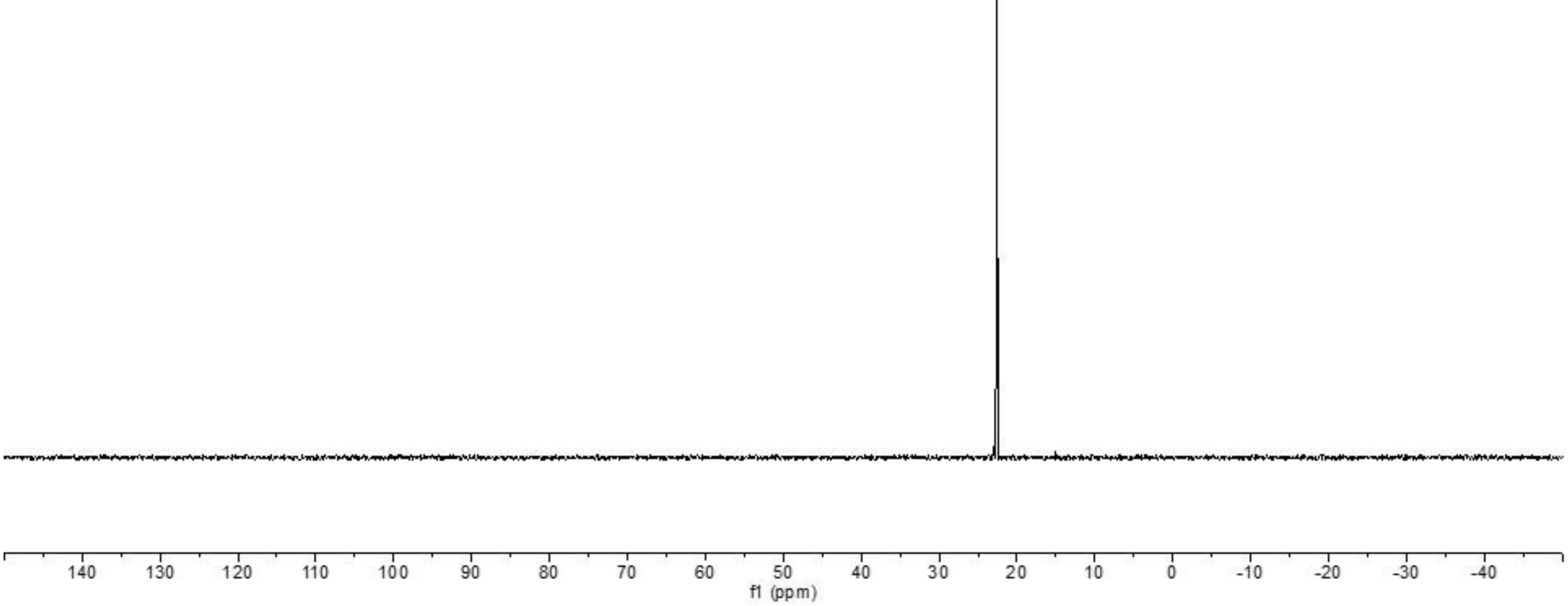

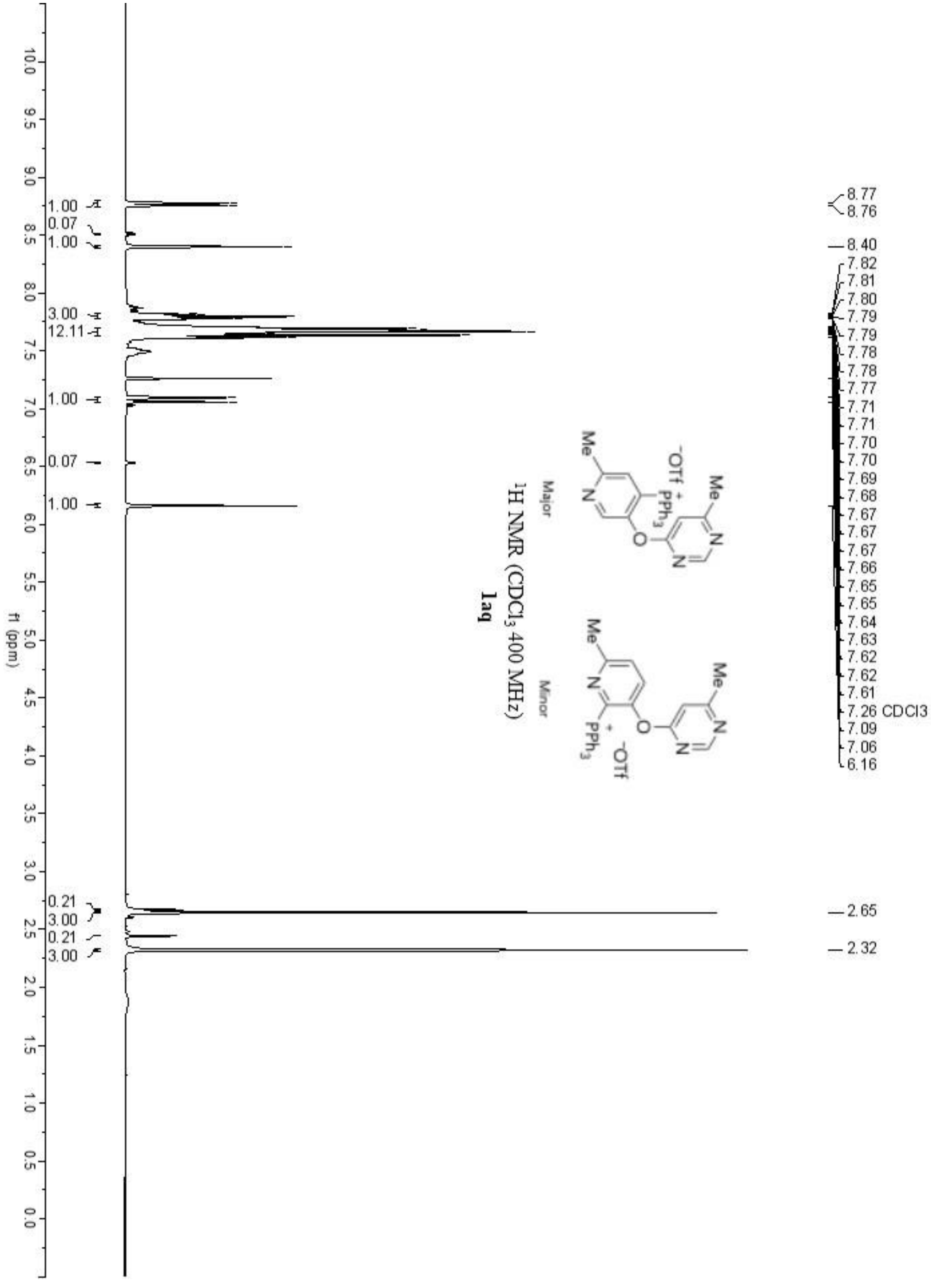

1H NMR (CDCl3 400 MHz)
1aq
Major
Minor
Me
N
O
PPh3
-OTf
8.77
8.76
8.40
7.82
7.81
7.80
7.79
7.79
7.78
7.78
7.77
7.71
7.71
7.70
7.70
7.69
7.68
7.67
7.67
7.67
7.66
7.65
7.65
7.64
7.63
7.62
7.62
7.61
7.26 CDCl3
7.09
7.06
6.16
2.65
2.32
1.00
0.07
1.00
3.00
12.11
1.00
0.07
1.00
0.21
3.00
0.21
3.00
f1 (ppm)

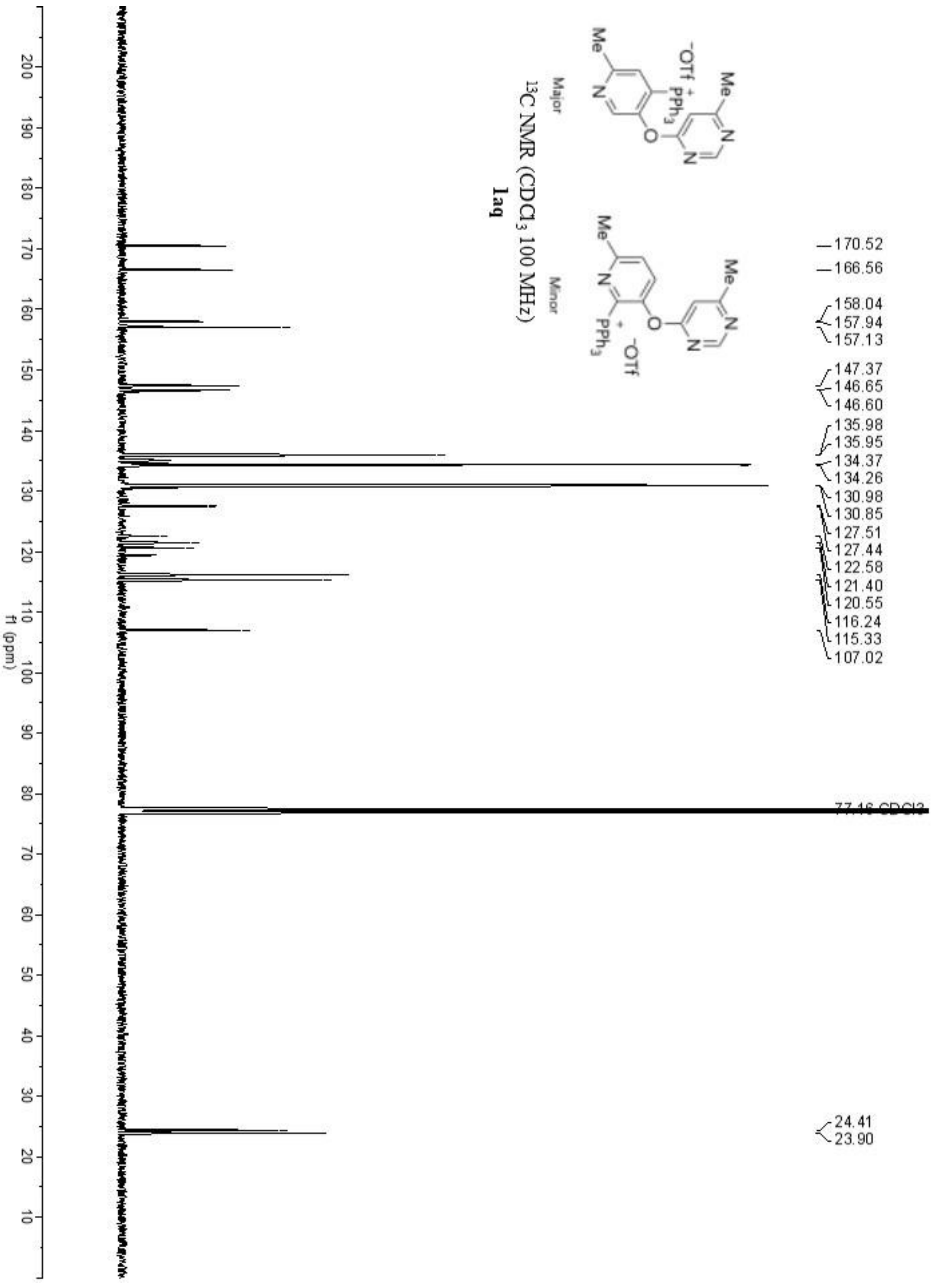
Major
Minor
Me
N
OTf
PPh3
O
13C NMR (CDCl3 100 MHz)
1aq
170.52
166.56
158.04
157.94
157.13
147.37
146.65
146.60
135.98
135.95
134.37
134.26
130.98
130.85
127.51
127.44
122.58
121.40
120.55
116.24
115.33
107.02
77.16 CDCl3
24.41
23.90
200
190
180
170
160
150
140
130
120
110
100
90
80
70
60
50
40
30
20
10
f1 (ppm)

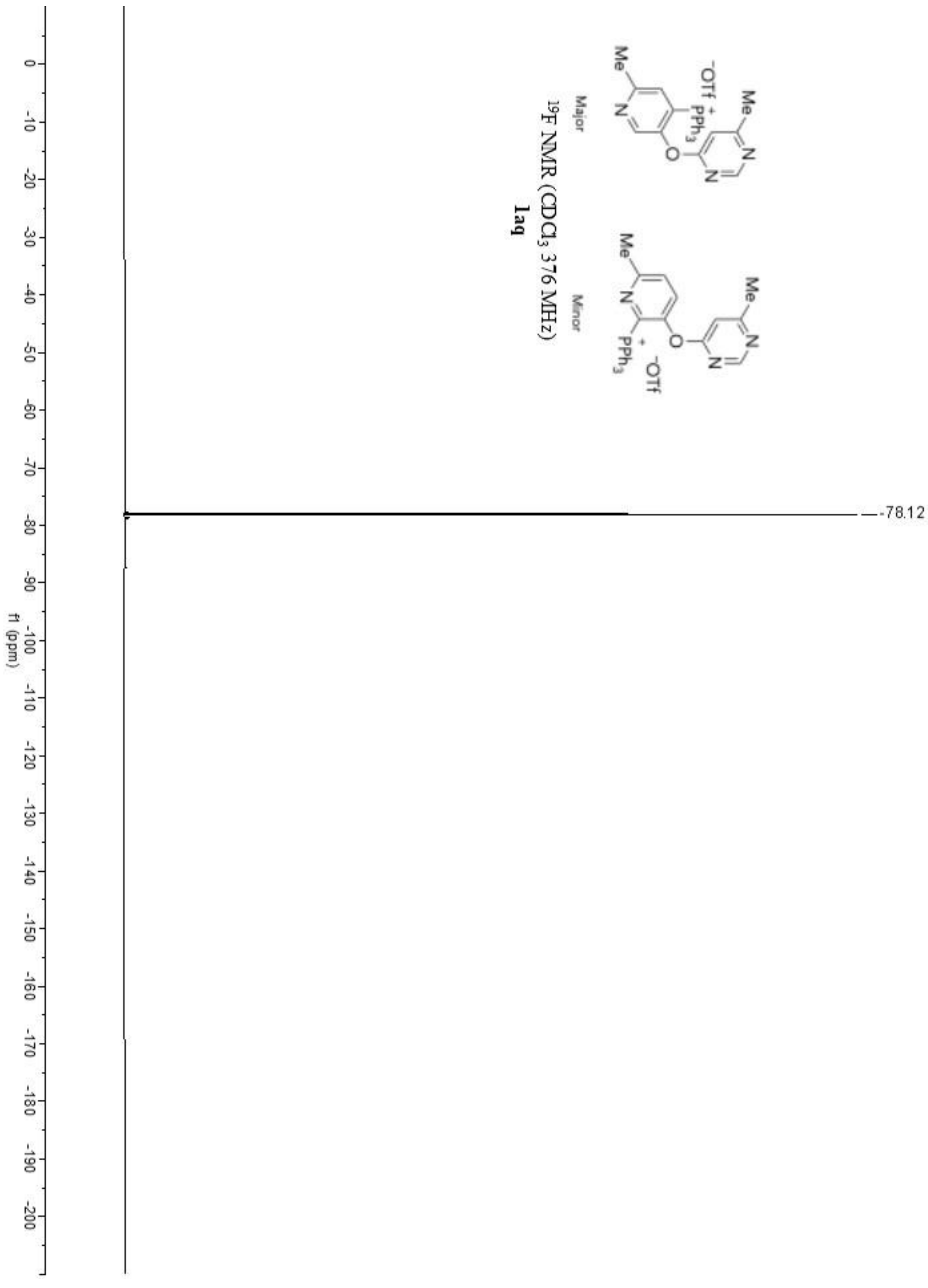

Major
Minor
^{19}F NMR (CDCl$_3$ 376 MHz)
1aq
-78.12
f1 (ppm)

^{31}P NMR ($CDCl_3$ 162 MHz)

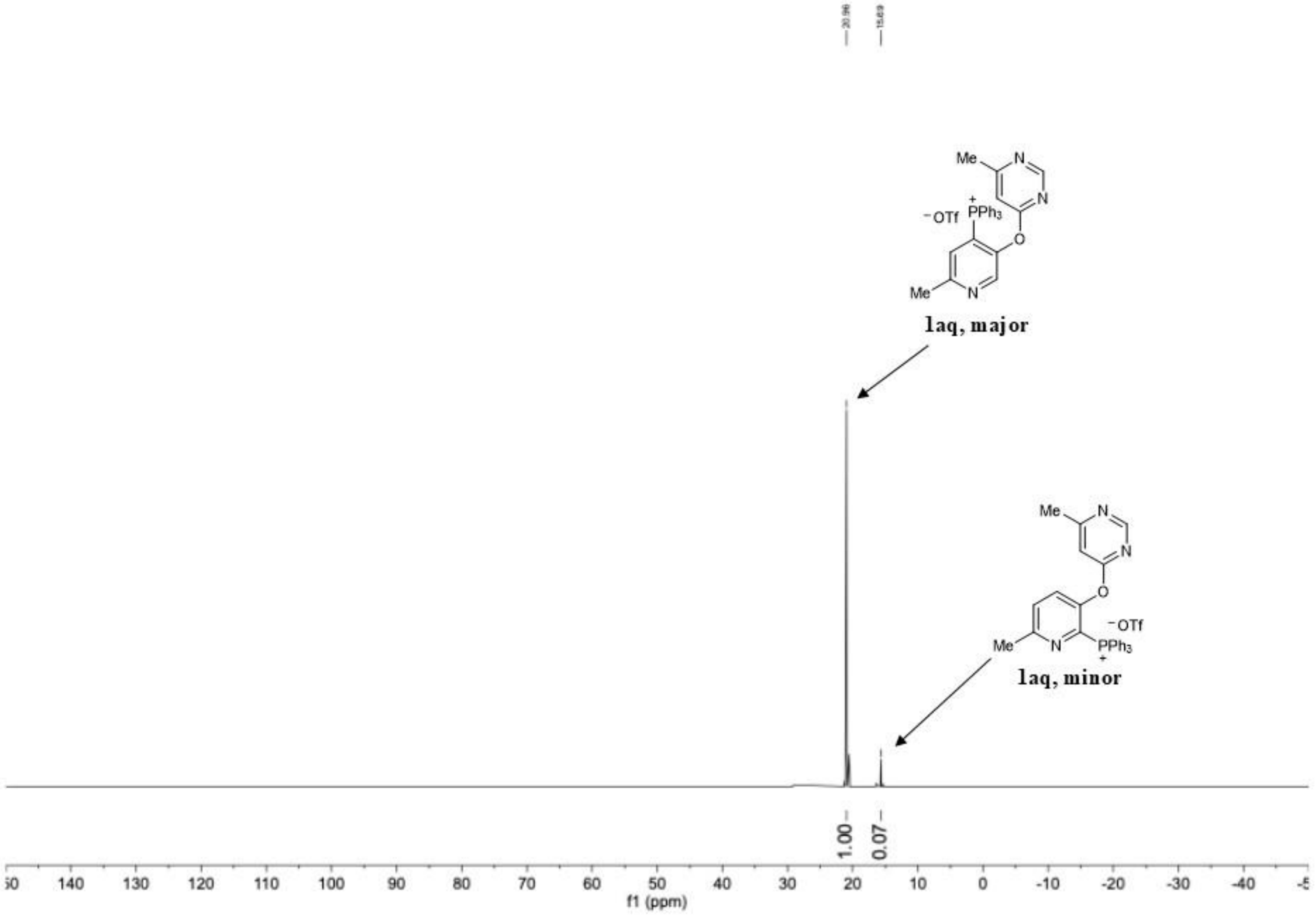

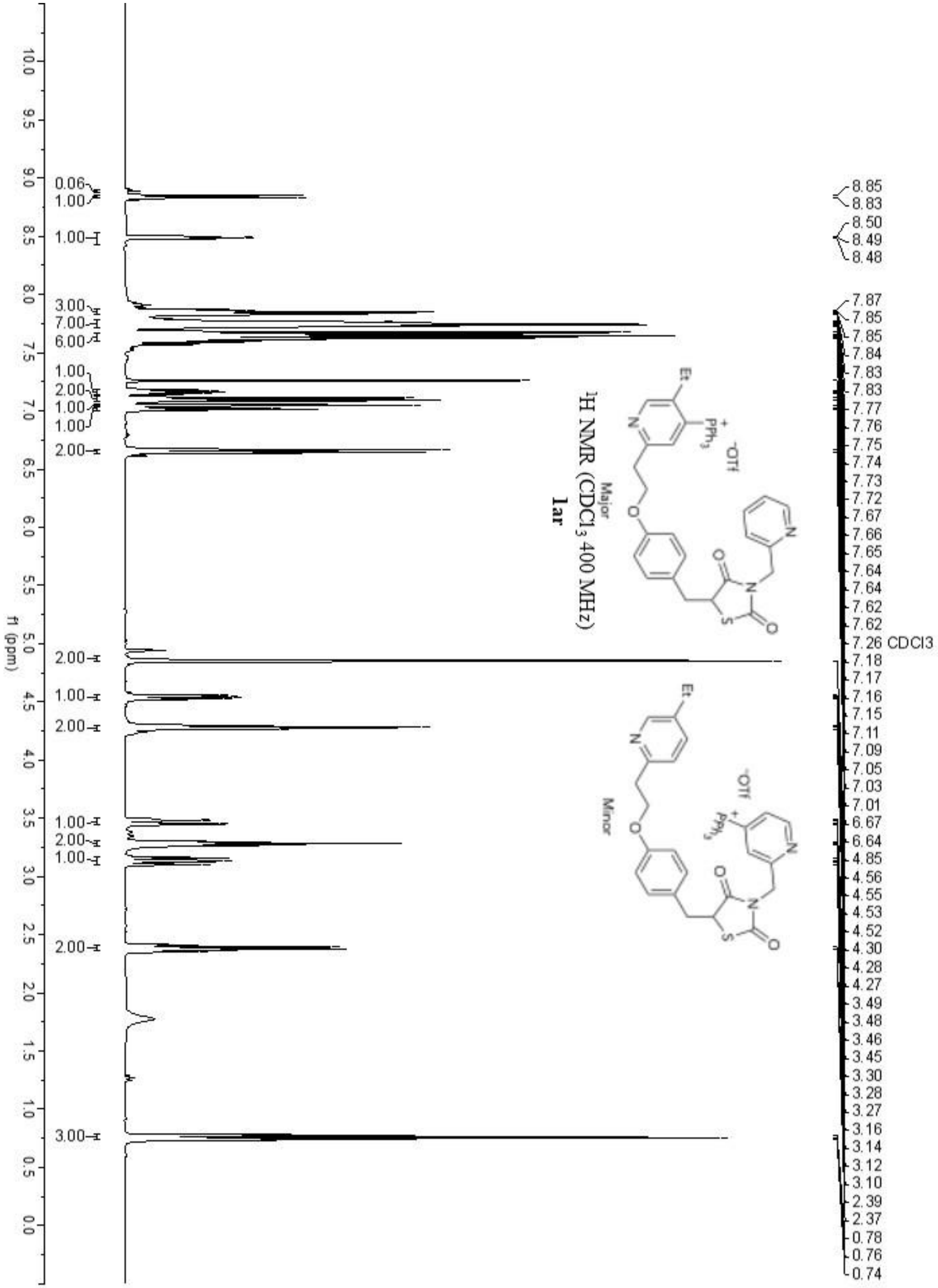
1H NMR (CDCl3 400 MHz)
1ar
Major
Minor
-OTf
PPh3
Et
8.85
8.83
8.50
8.49
8.48
7.87
7.85
7.85
7.84
7.83
7.83
7.77
7.76
7.75
7.74
7.73
7.72
7.67
7.66
7.65
7.64
7.64
7.62
7.62
7.26 CDCl3
7.18
7.17
7.16
7.15
7.11
7.09
7.05
7.03
7.01
6.67
6.64
4.85
4.56
4.55
4.53
4.52
4.30
4.28
4.27
3.49
3.48
3.46
3.45
3.30
3.28
3.27
3.16
3.14
3.12
3.10
2.39
2.37
0.78
0.76
0.74
f1 (ppm)

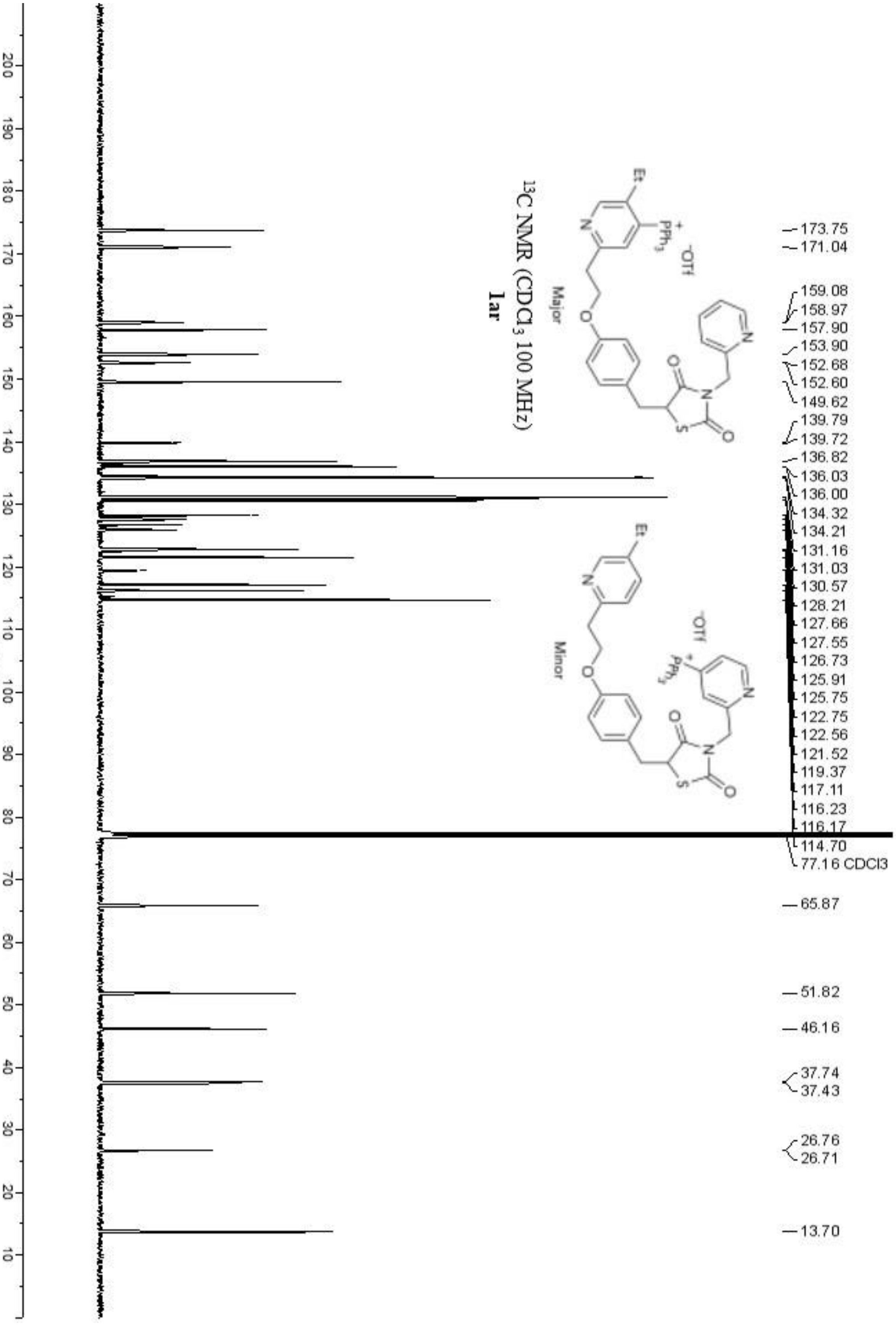

^{13}C NMR (CDCl$_3$ 100 MHz)
1ar
Major
Minor
173.75
171.04
159.08
158.97
157.90
153.90
152.68
152.60
149.62
139.79
139.72
136.82
136.03
136.00
134.32
134.21
131.16
131.03
130.57
128.21
127.66
127.55
126.73
125.91
125.75
122.75
122.56
121.52
119.37
117.11
116.23
116.17
114.70
77.16 CDCl3
65.87
51.82
46.16
37.74
37.43
26.76
26.71
13.70
f1 (ppm)

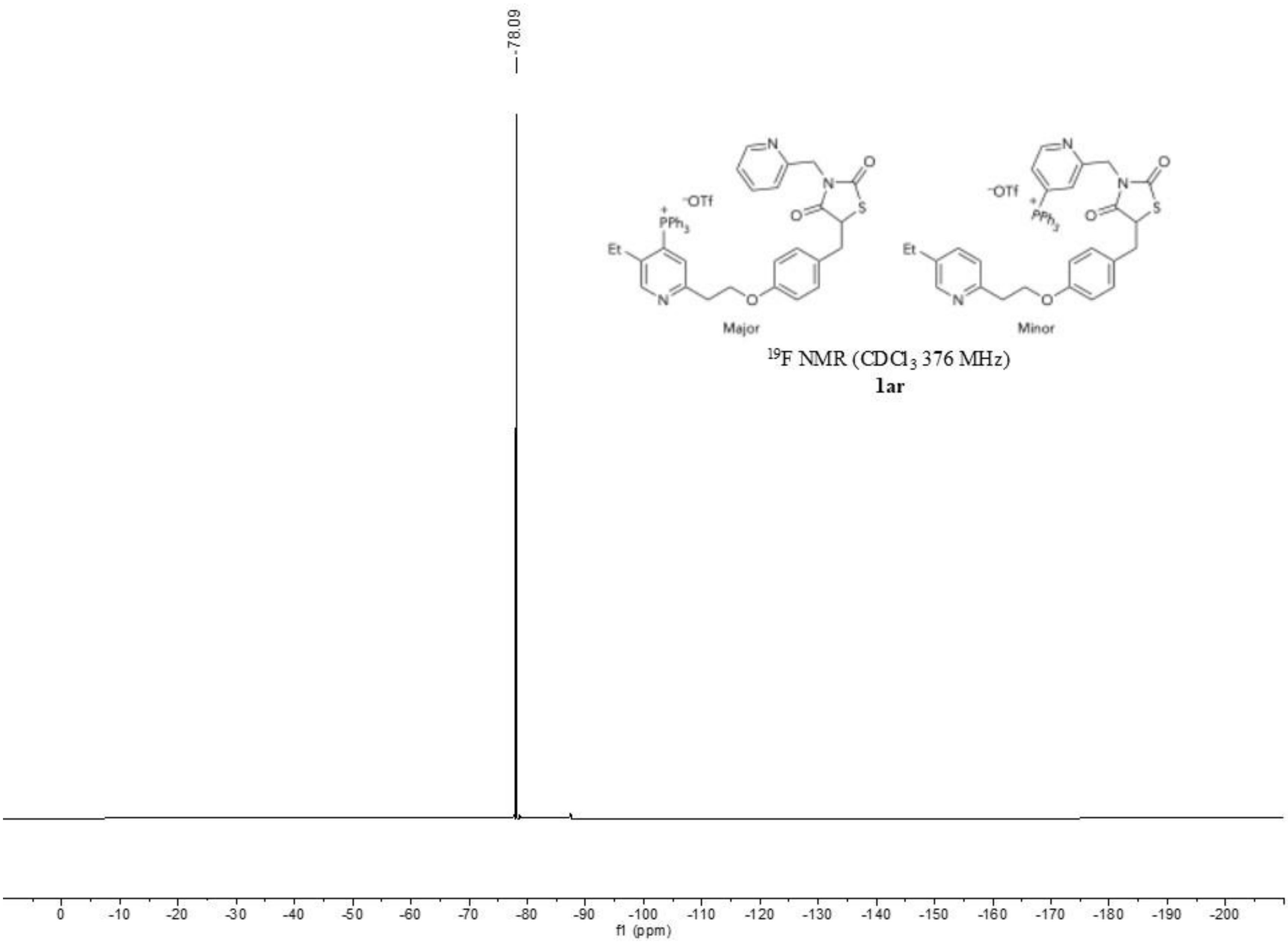

^{19}F NMR ($CDCl_3$ 376 MHz)

1ar

^{31}P NMR (CDCl$_3$ 162 MHz)

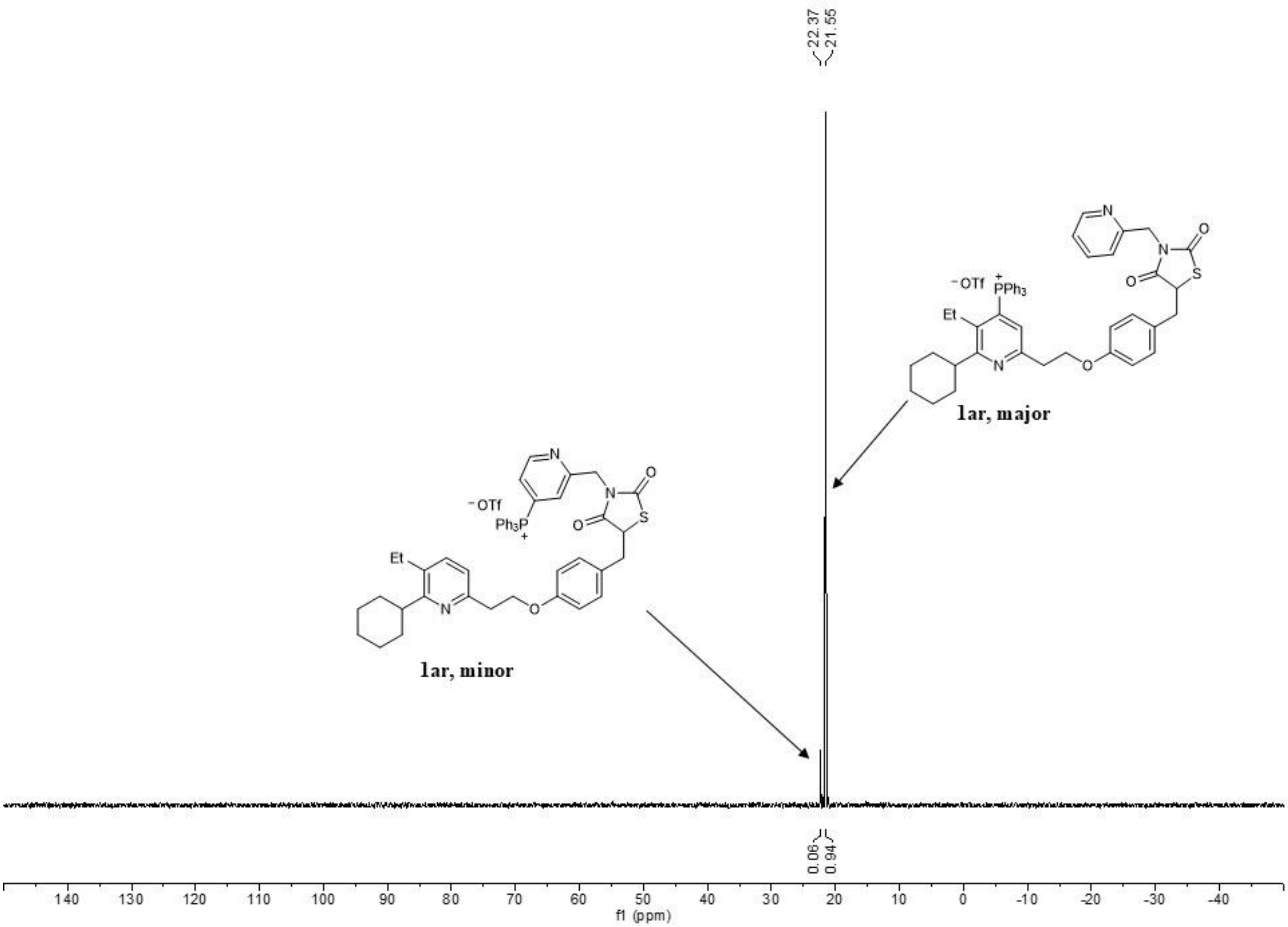

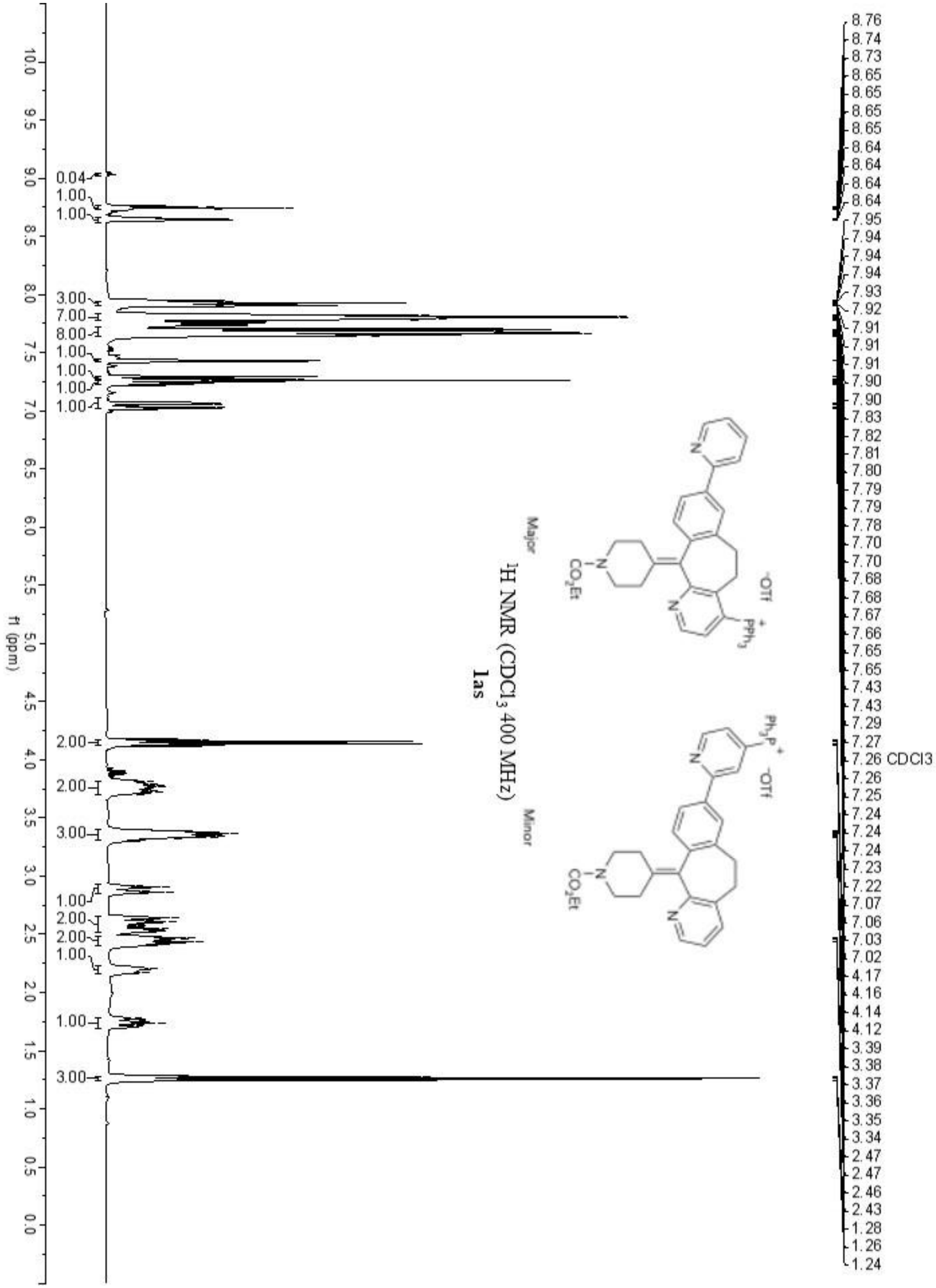
Major
Minor
-OTf
PPh3
Ph3P
CO2Et
1H NMR (CDCl3 400 MHz)
1as
CDCl3
f1 (ppm)

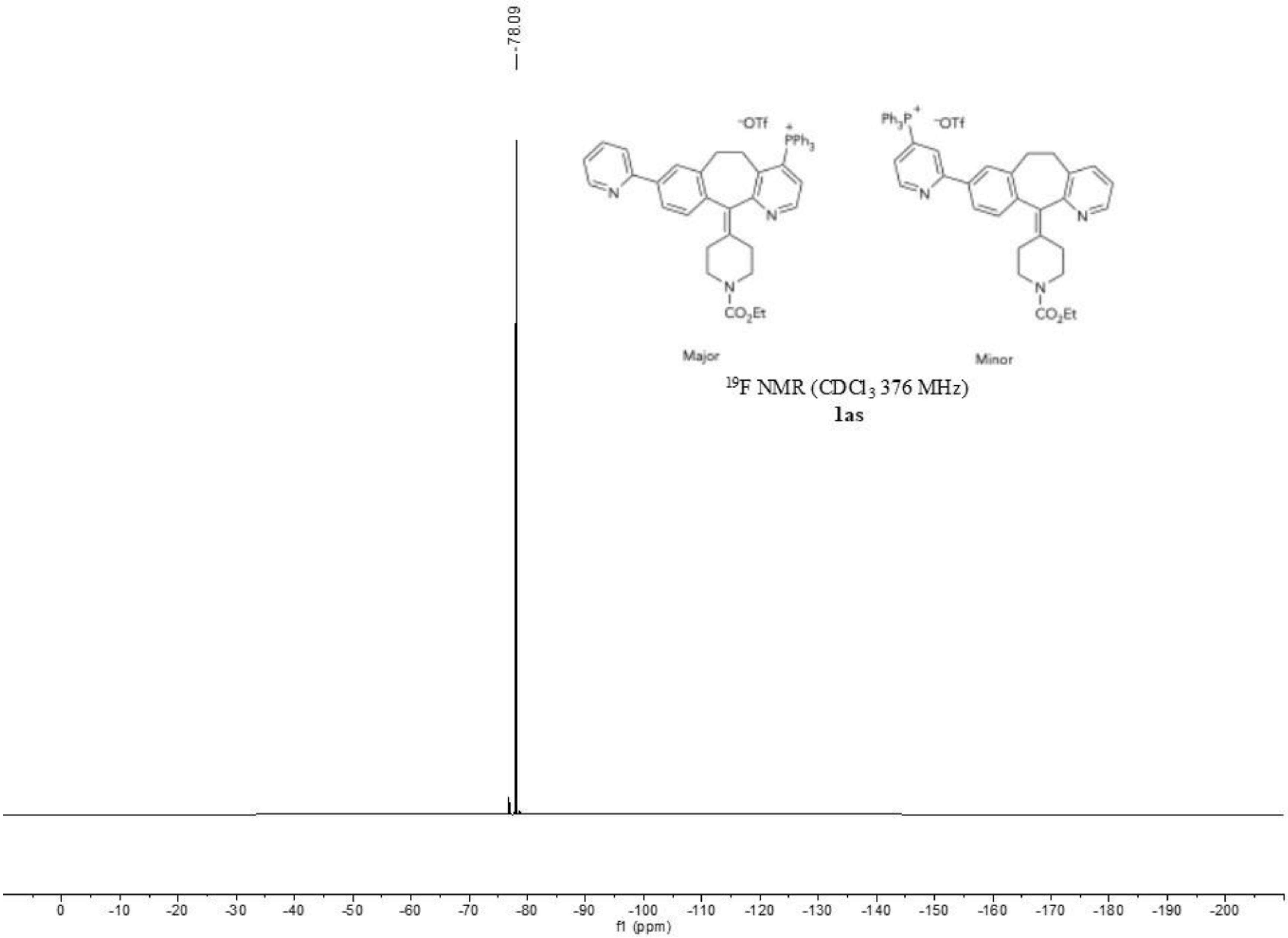

-78.09
-OTf
PPh3
Ph3P
N
CO2Et
Major
Minor
19F NMR (CDCl3 376 MHz)
1as
0
-10
-20
-30
-40
-50
-60
-70
-80
-90
-100
-110
-120
-130
-140
-150
-160
-170
-180
-190
-200
f1 (ppm)

^{31}P NMR ($CDCl_3$ 162 MHz)

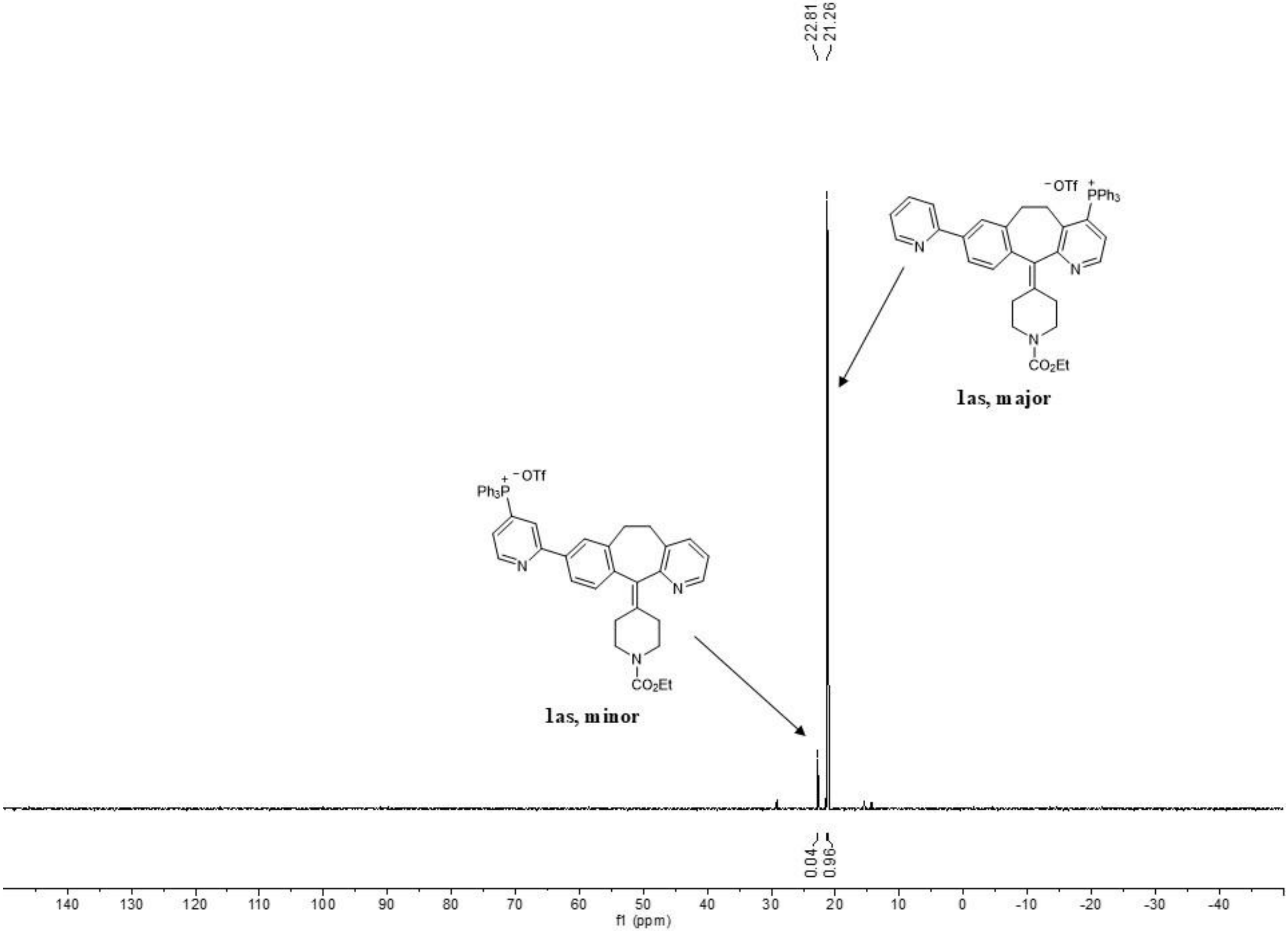

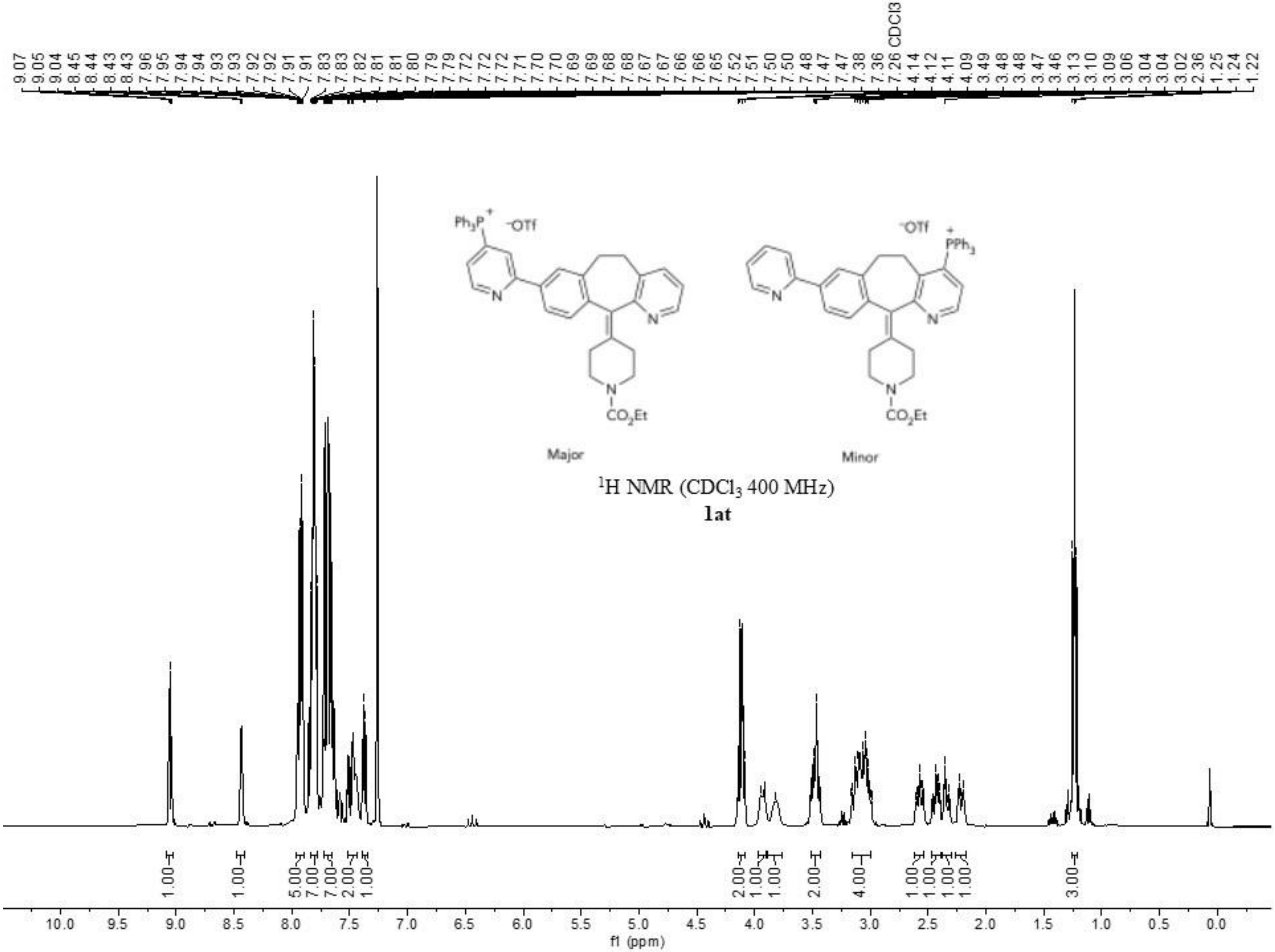

^{1}H NMR ($CDCl_3$ 400 MHz)

1at

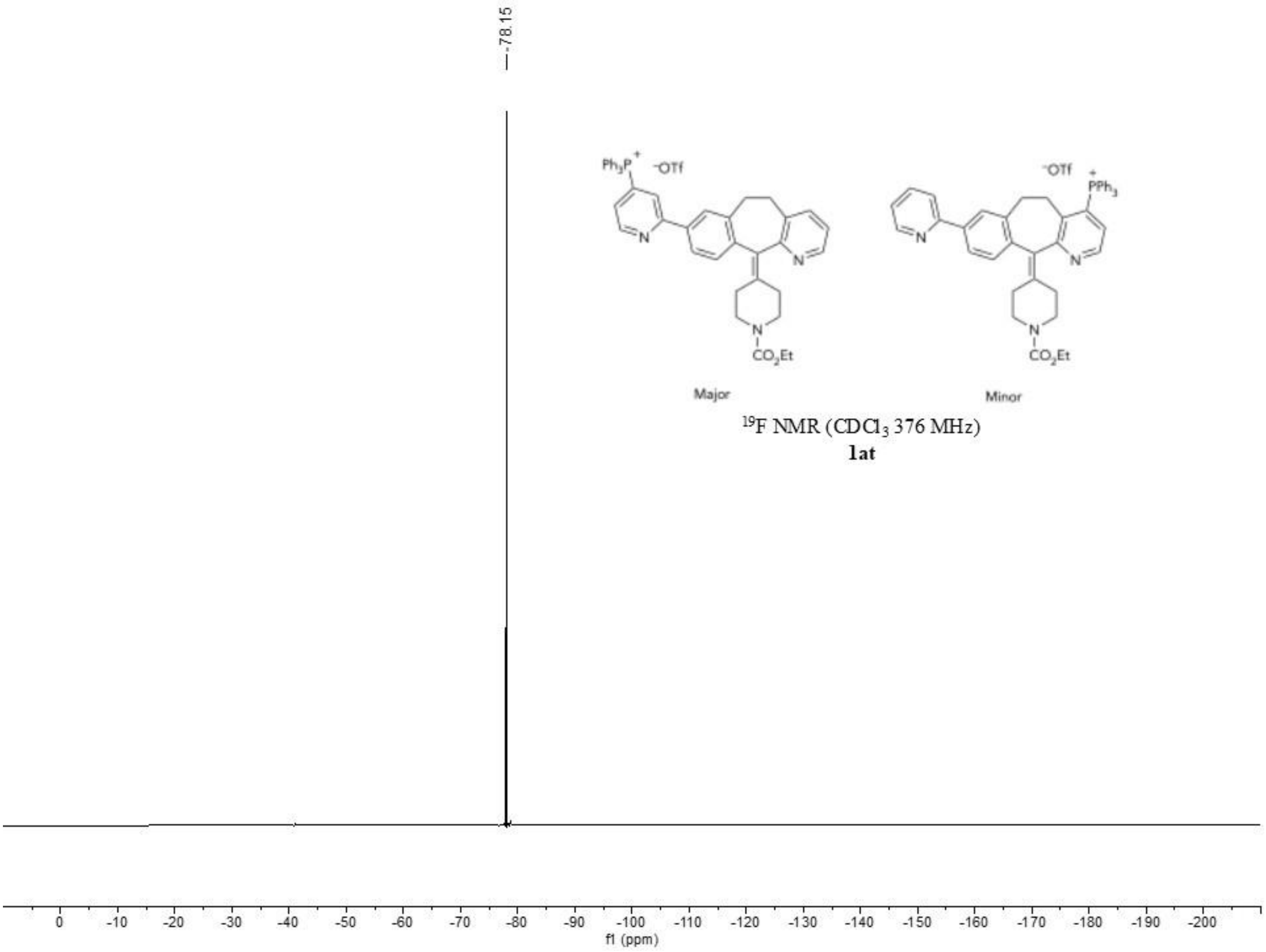

^{19}F NMR ($CDCl_3$ 376 MHz)

1at

^{31}P NMR ($CDCl_3$ 162 MHz)

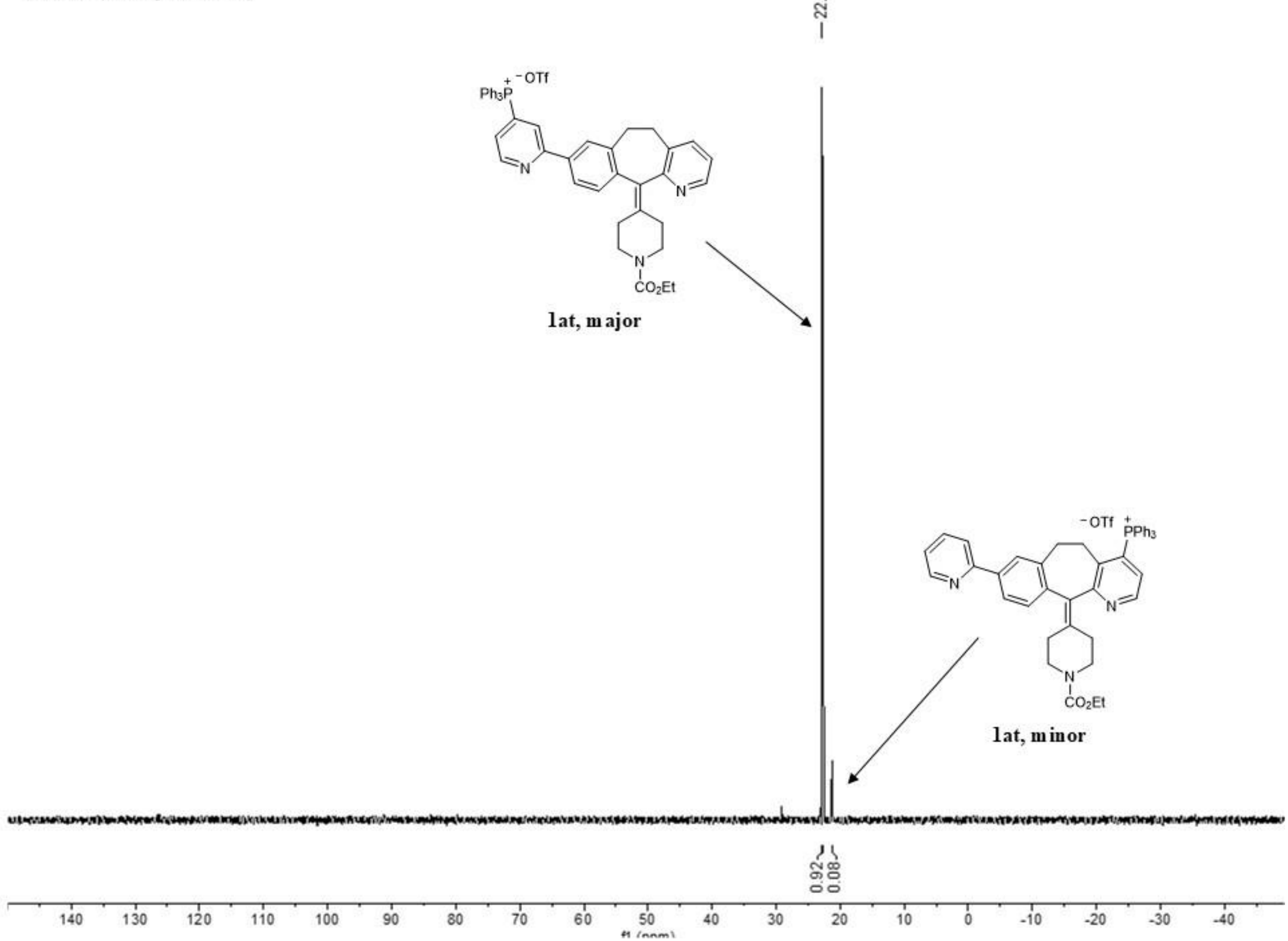

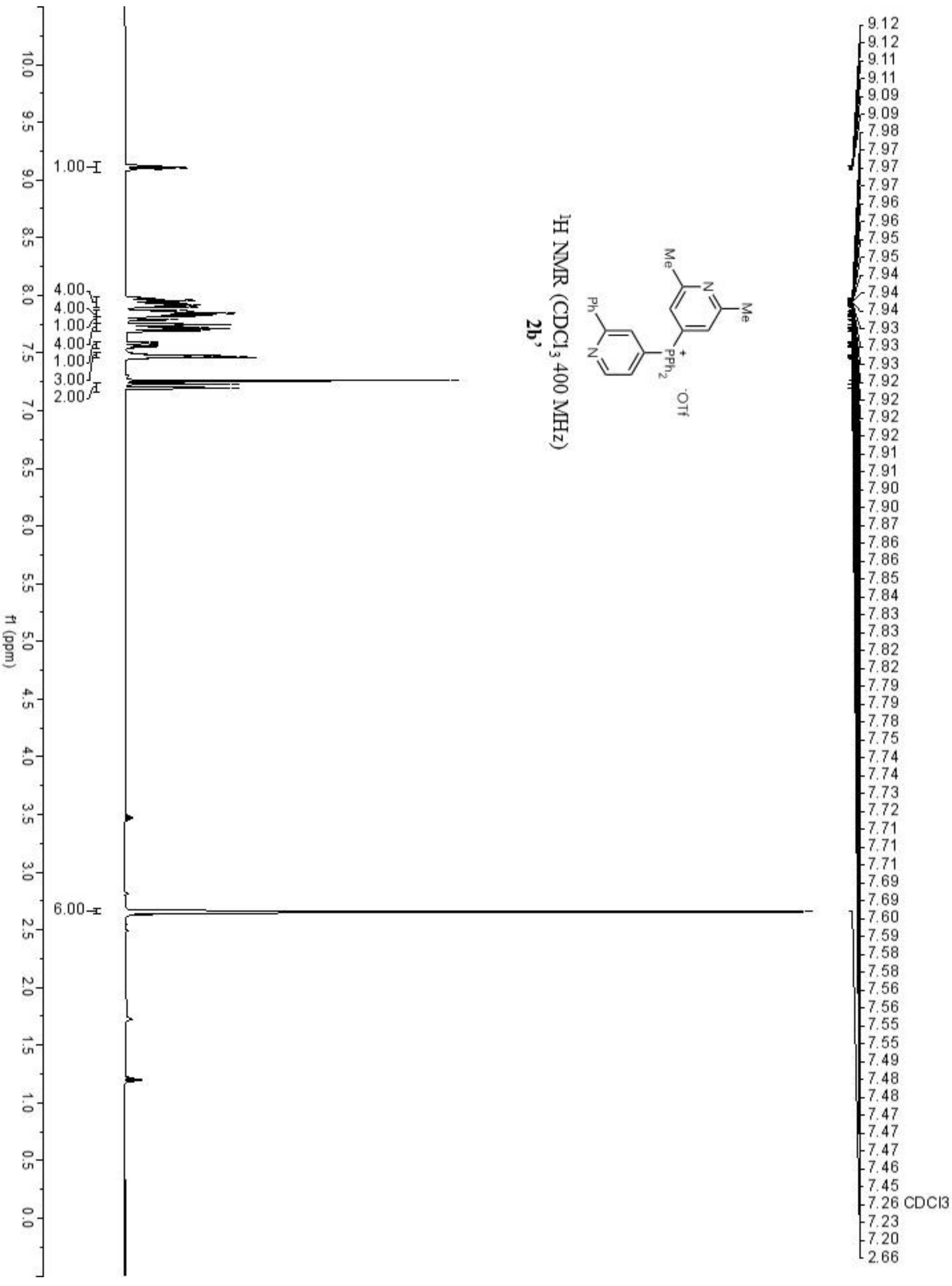
1H NMR (CDCl3 400 MHz)
2b'
Me
N
Me
PPh2
OTf
Ph
N
9.12
9.12
9.11
9.11
9.09
9.09
7.98
7.97
7.97
7.97
7.96
7.96
7.95
7.95
7.94
7.94
7.94
7.93
7.93
7.93
7.92
7.92
7.92
7.92
7.91
7.91
7.90
7.90
7.87
7.86
7.86
7.85
7.84
7.83
7.83
7.82
7.82
7.79
7.79
7.78
7.75
7.74
7.74
7.73
7.72
7.71
7.71
7.71
7.69
7.69
7.60
7.59
7.58
7.58
7.56
7.56
7.55
7.55
7.49
7.48
7.48
7.47
7.47
7.47
7.46
7.45
7.26 CDCl3
7.23
7.20
2.66
1.00
4.00
4.00
1.00
4.00
1.00
3.00
2.00
6.00
10.0
9.5
9.0
8.5
8.0
7.5
7.0
6.5
6.0
5.5
5.0
4.5
4.0
3.5
3.0
2.5
2.0
1.5
1.0
0.5
0.0
f1 (ppm)

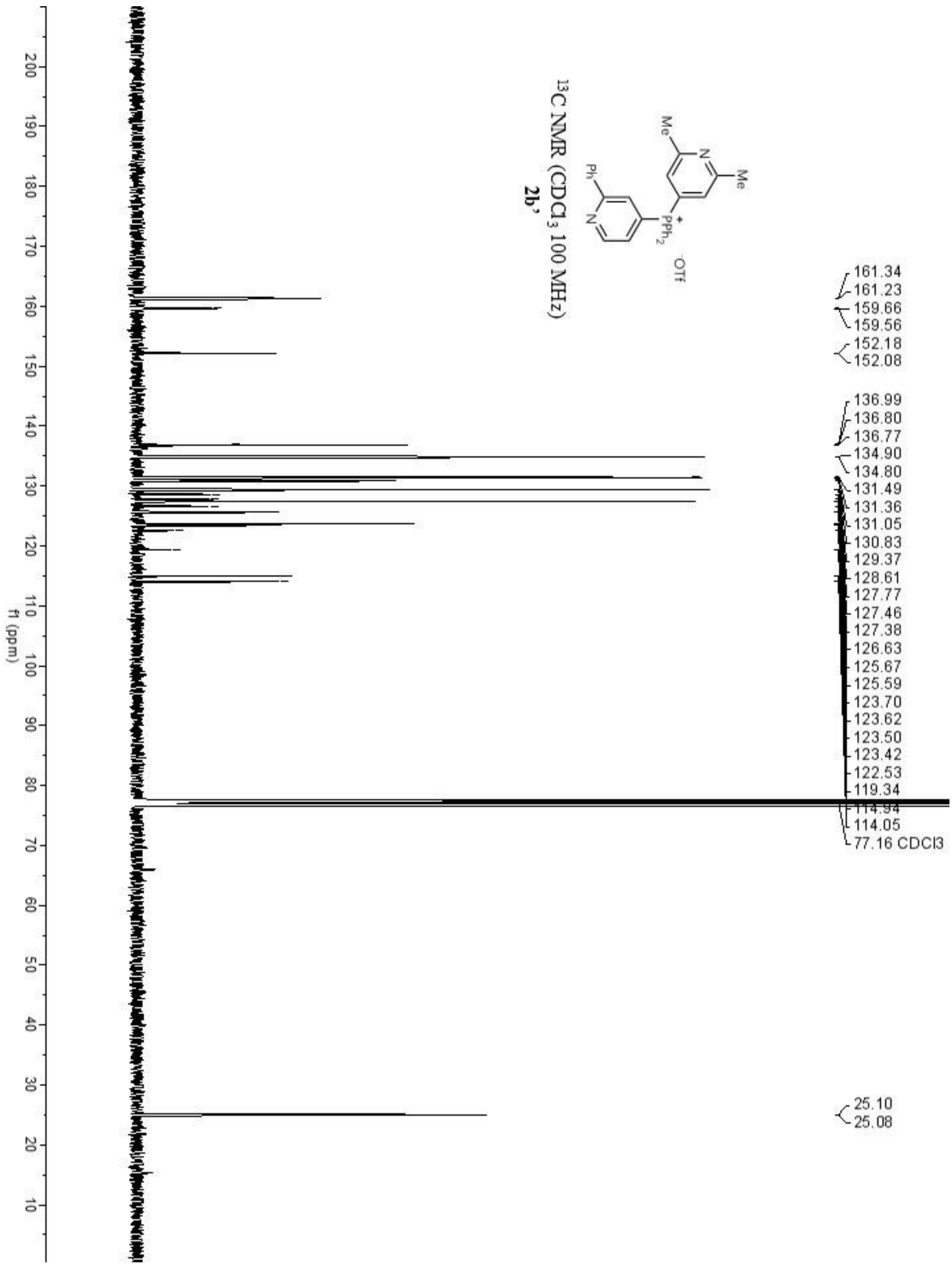
13C NMR (CDCl3 100 MHz)
2b'
Me
N
Me
PPh2
-OTf
Ph
N
161.34
161.23
159.66
159.56
152.18
152.08
136.99
136.80
136.77
134.90
134.80
131.49
131.36
131.05
130.83
129.37
128.61
127.77
127.46
127.38
126.63
125.67
125.59
123.70
123.62
123.50
123.42
122.53
119.34
114.94
114.05
77.16 CDCl3
25.10
25.08
f1 (ppm)

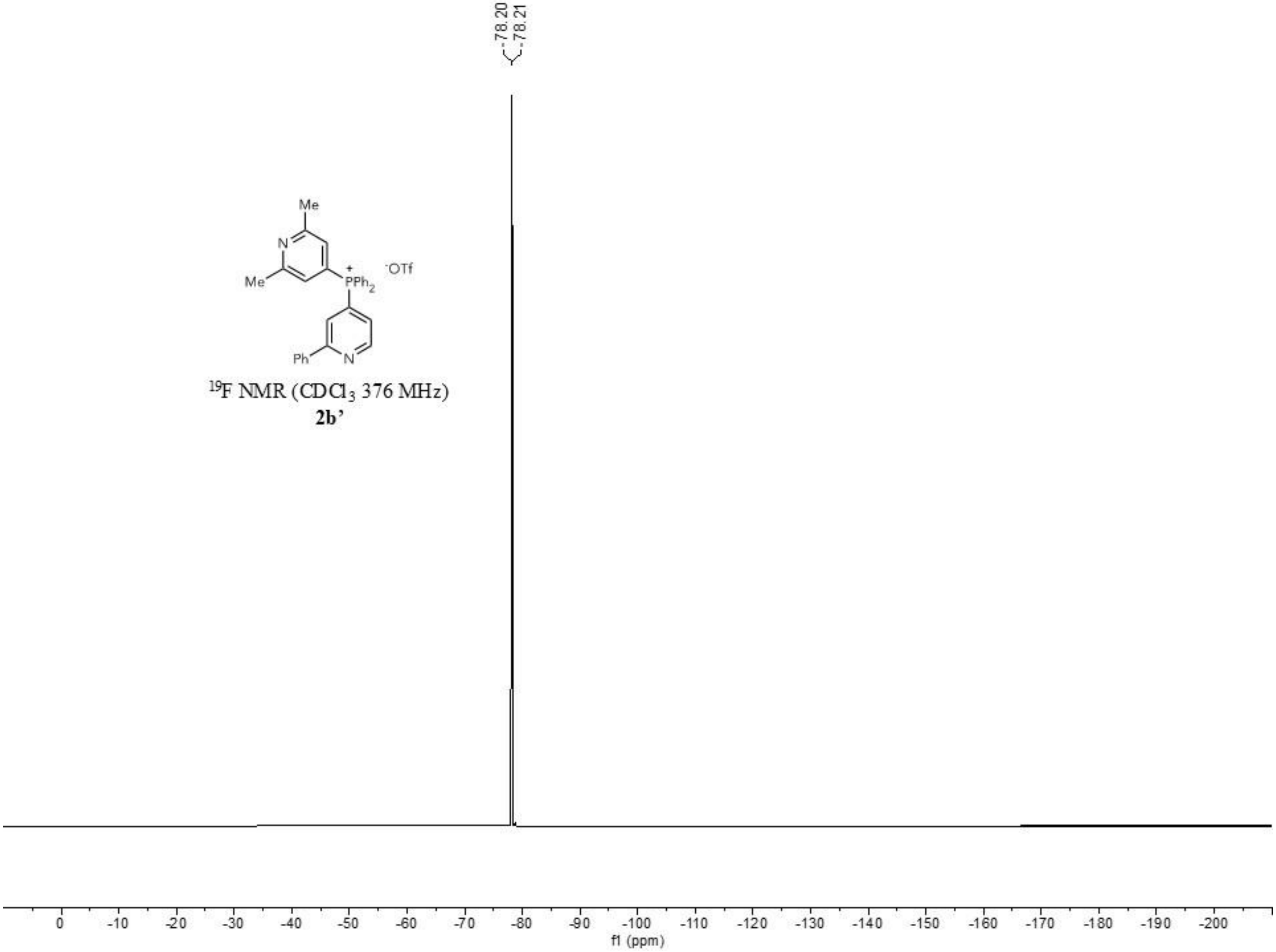

^{19}F NMR ($CDCl_3$ 376 MHz)
2b'

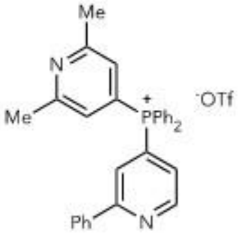

^{31}P NMR (CDCl$_3$ 162 MHz)
2b’

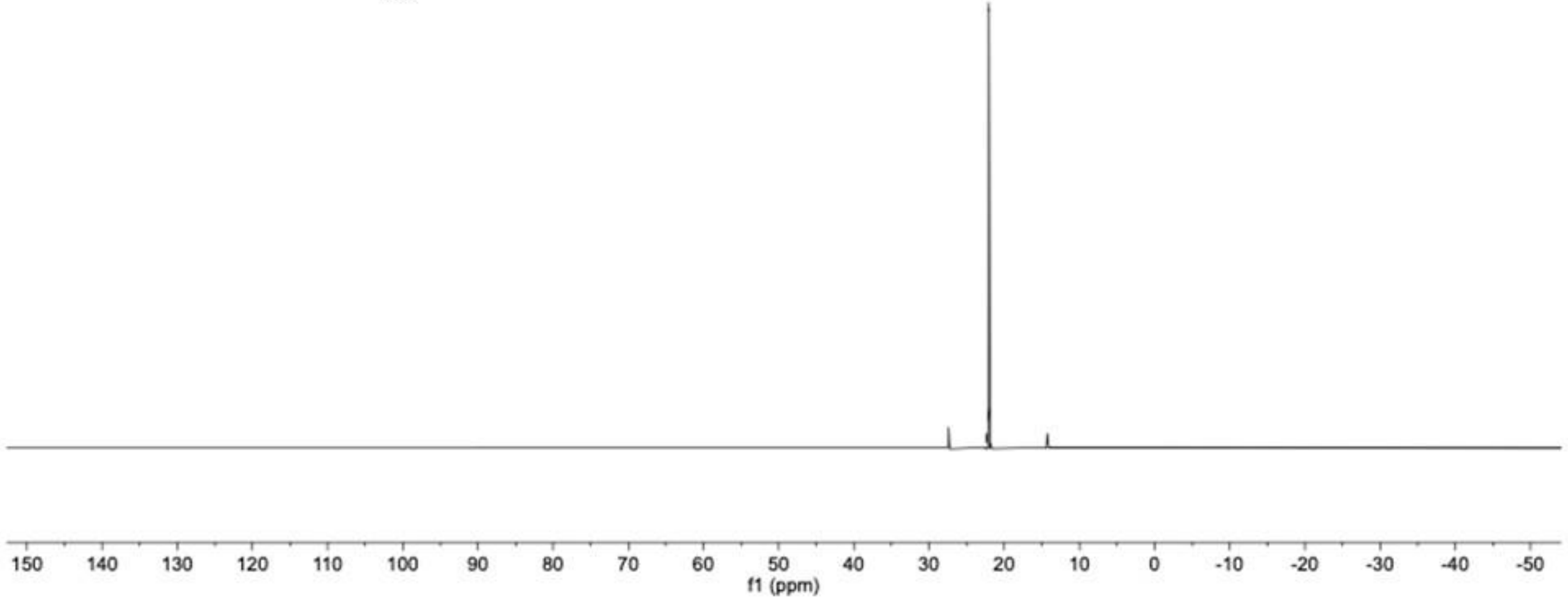

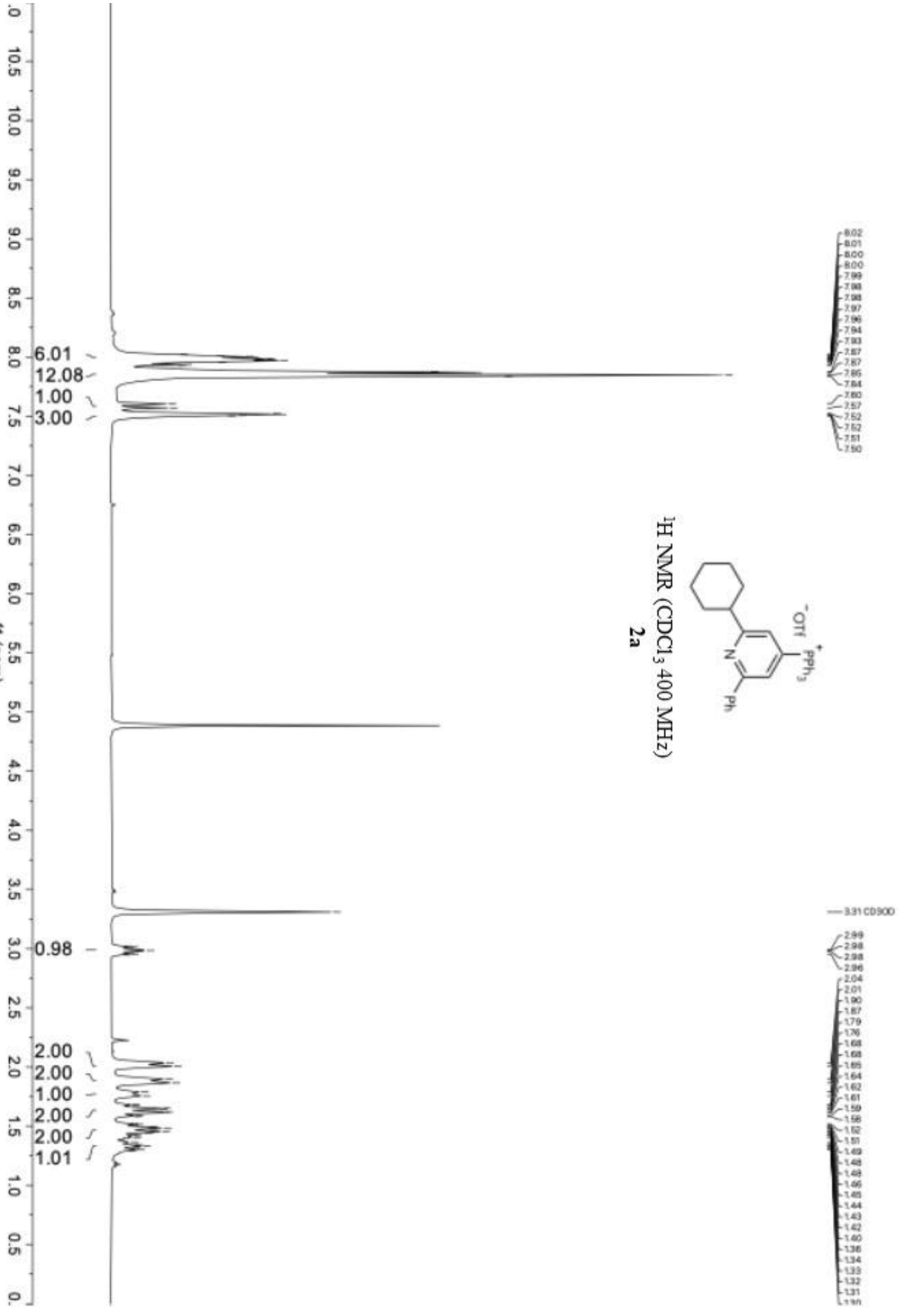

1H NMR (CDCl3 400 MHz)
2a
f1 (ppm)

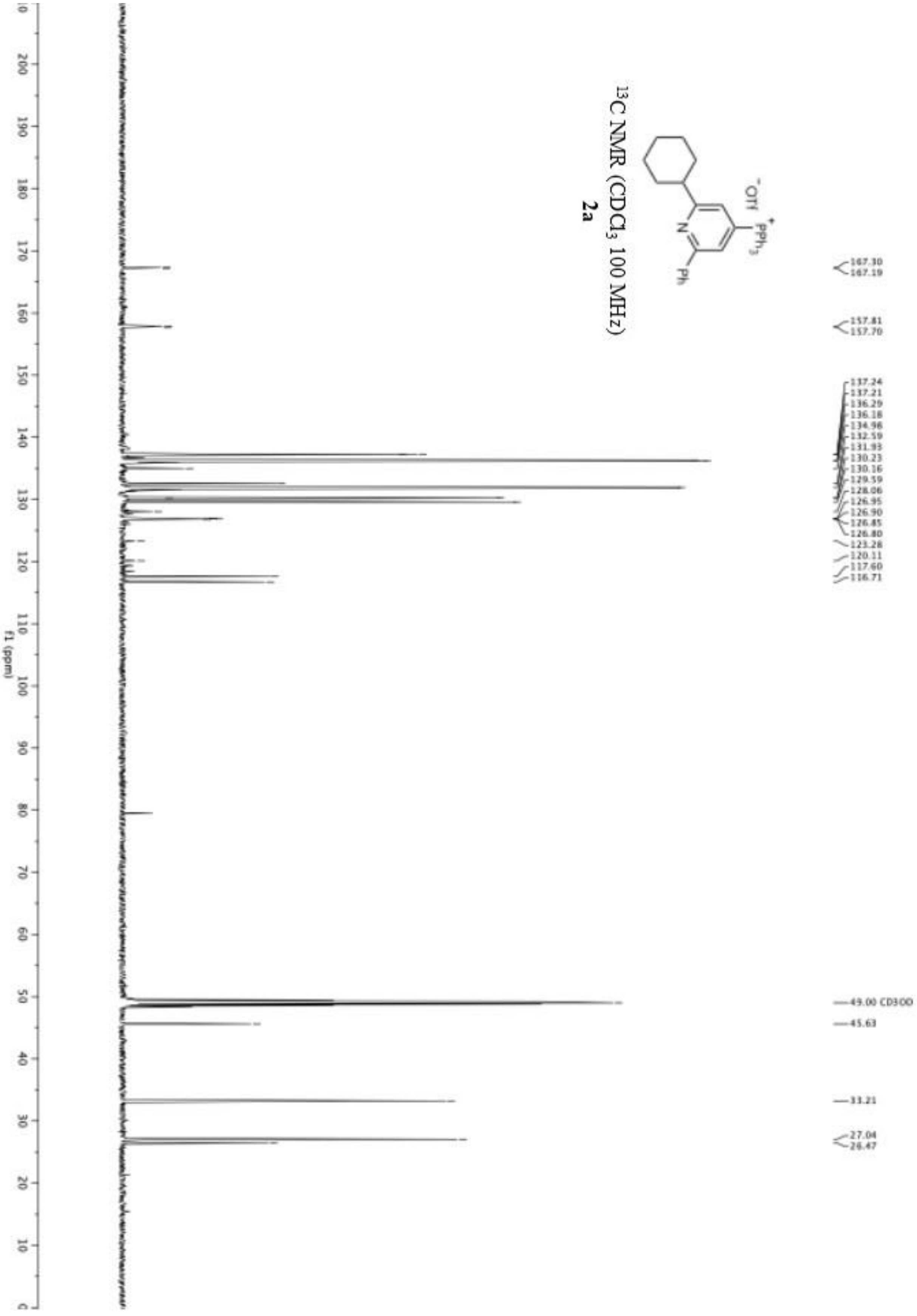

13C NMR (CDCl3 100 MHz)
2a
-OTf
+PPh3
N
Ph
167.30
167.19
157.81
157.70
137.24
137.21
136.29
136.18
134.96
132.59
131.93
130.23
130.16
129.59
128.06
126.95
126.90
126.85
126.80
123.28
120.11
117.60
116.71
49.00 CD3OD
45.63
33.21
27.04
26.47
f1 (ppm)

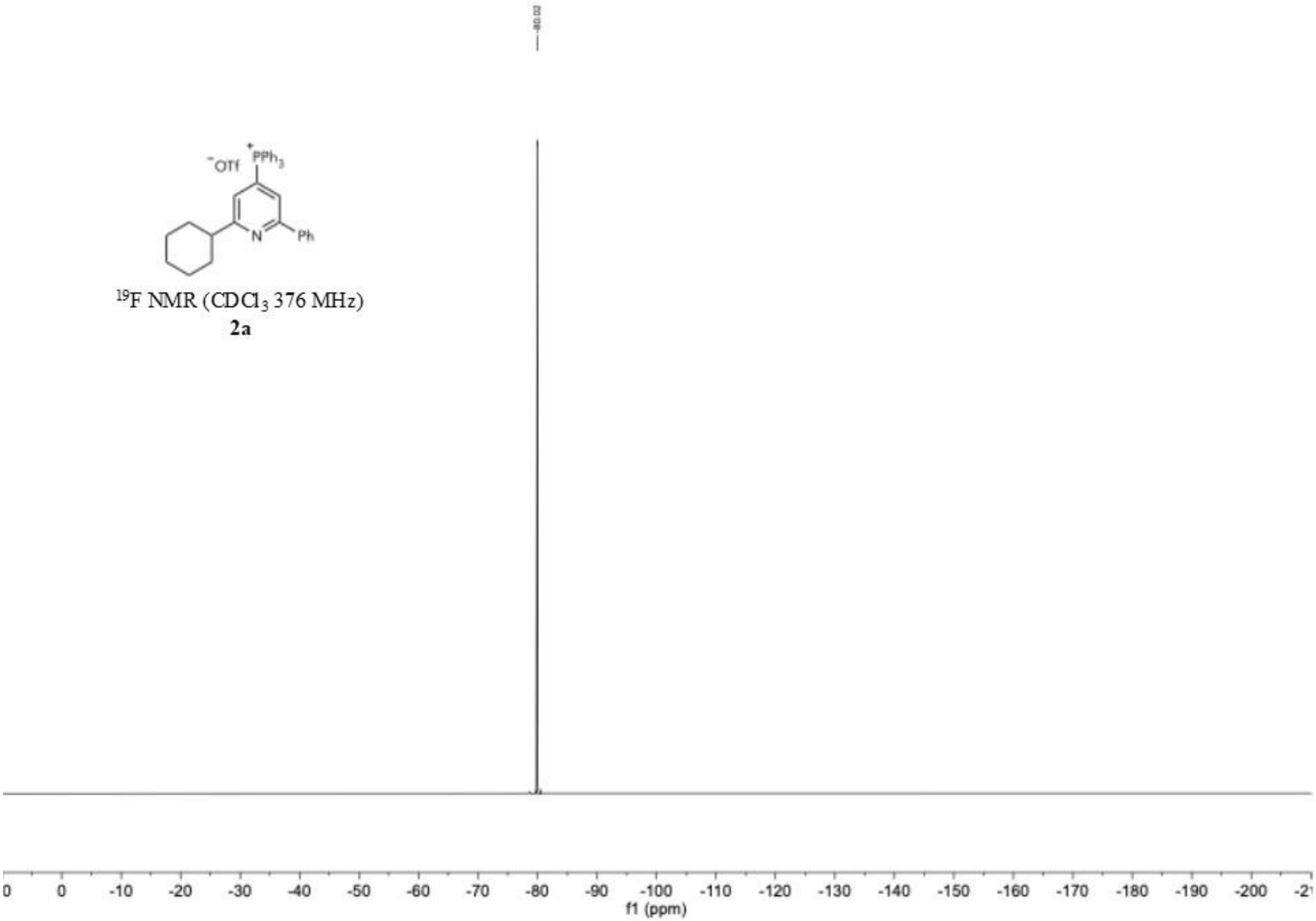

^{19}F NMR (CDCl$_3$ 376 MHz)
2a

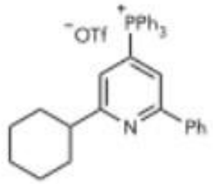

^{31}P NMR ($CDCl_3$ 162 MHz)

2a

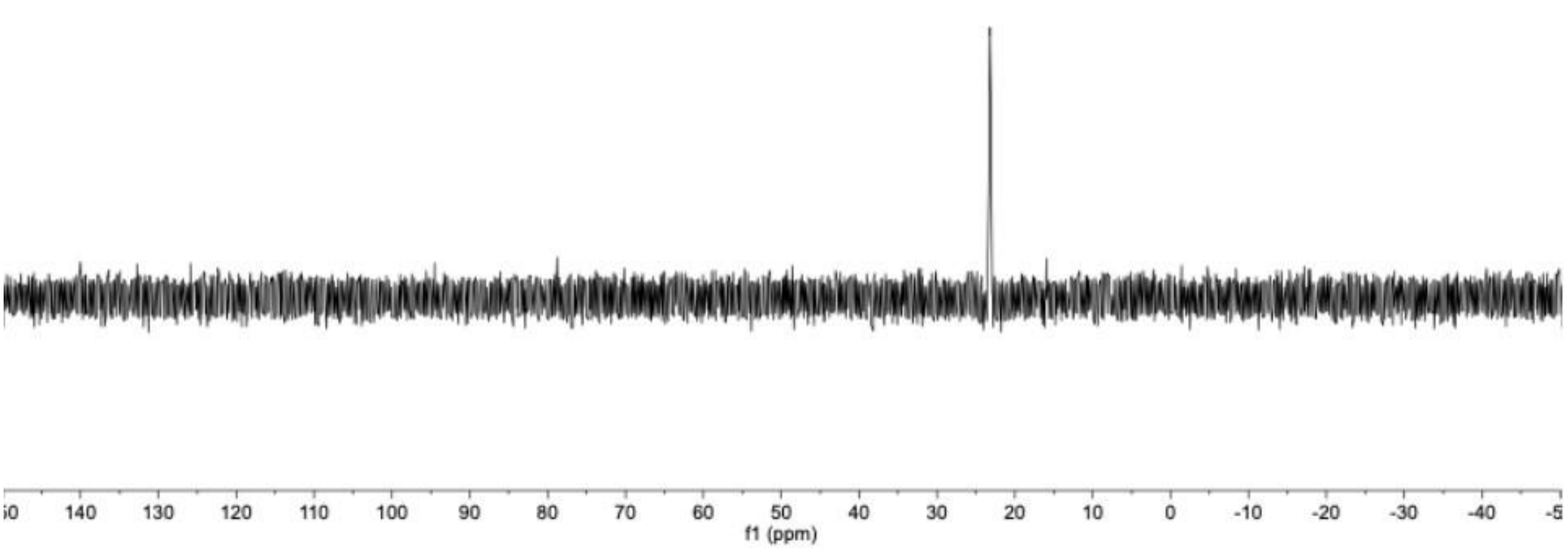

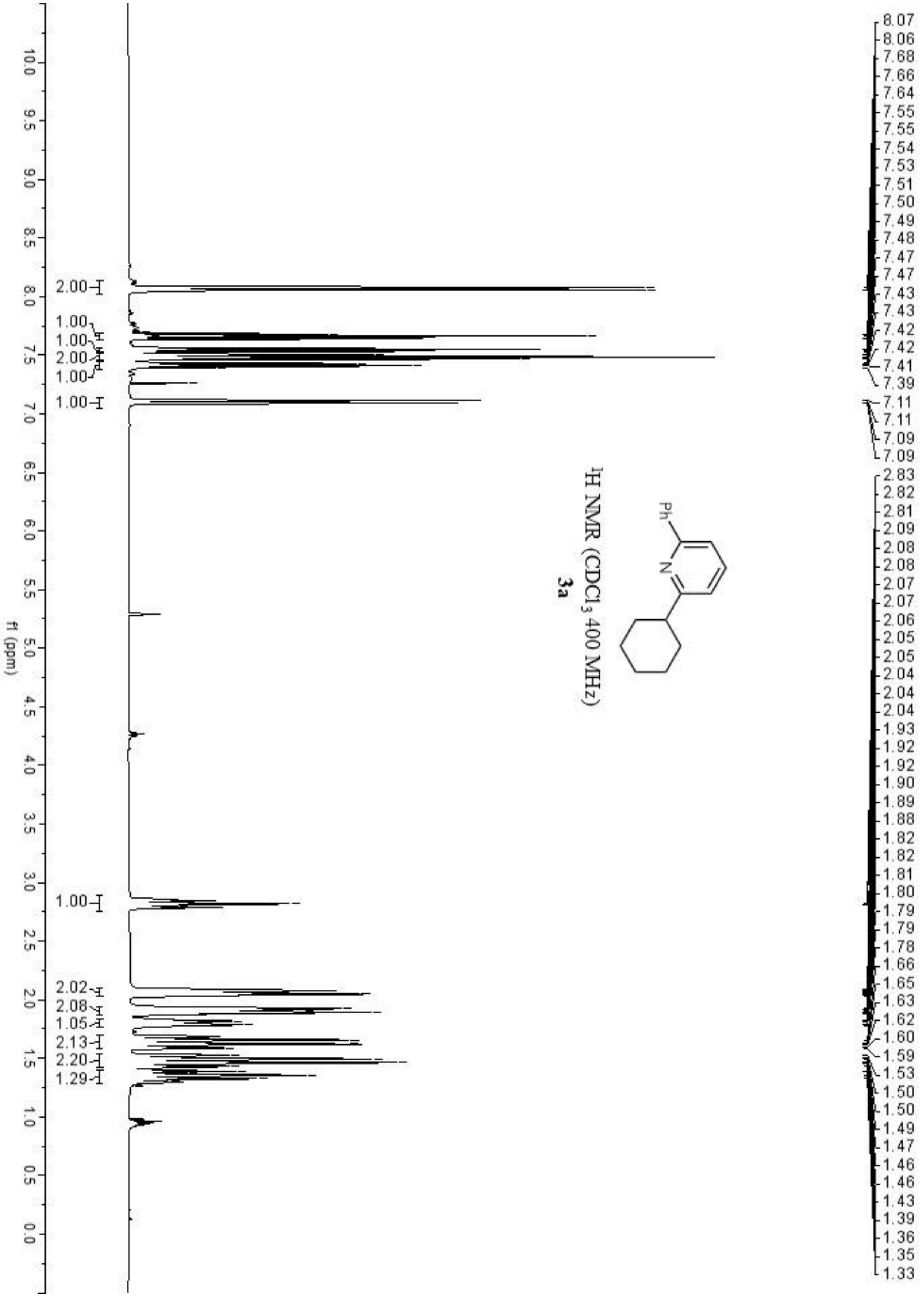

8.07
8.06
7.68
7.66
7.64
7.55
7.55
7.54
7.53
7.51
7.50
7.49
7.48
7.47
7.47
7.43
7.43
7.42
7.42
7.41
7.39
7.11
7.11
7.09
7.09
2.83
2.82
2.81
2.09
2.08
2.08
2.07
2.07
2.06
2.05
2.05
2.04
2.04
2.04
1.93
1.92
1.92
1.90
1.89
1.88
1.82
1.82
1.81
1.80
1.79
1.79
1.78
1.66
1.65
1.63
1.62
1.60
1.59
1.53
1.50
1.50
1.49
1.47
1.46
1.46
1.43
1.39
1.36
1.35
1.33
Ph
N
^{1}H NMR (CDCl$_3$ 400 MHz)
3a
2.00
1.00
1.00
2.00
1.00
1.00
1.00
2.02
2.08
1.05
2.13
2.20
1.29
f1 (ppm)

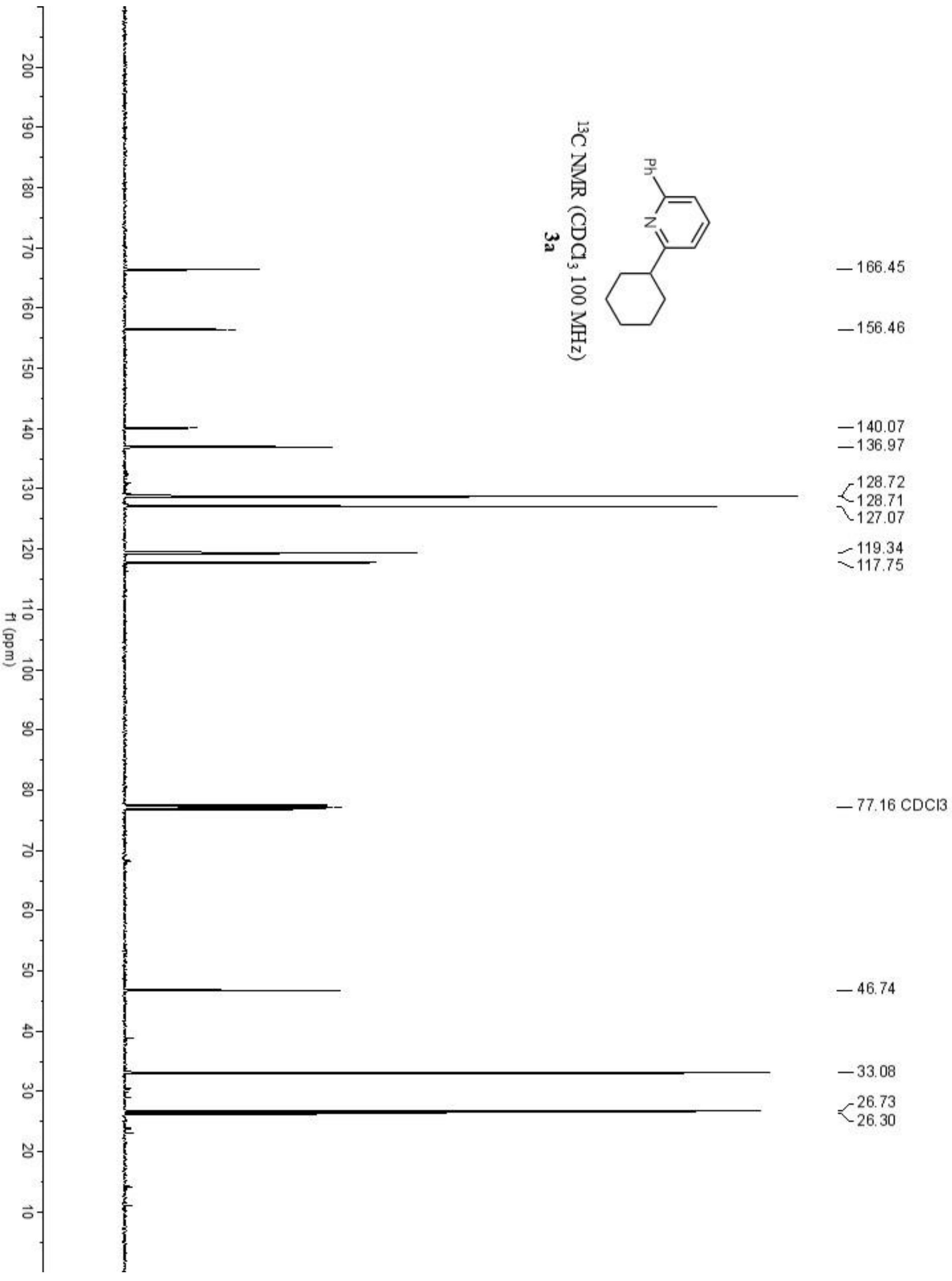

13C NMR (CDCl3 100 MHz)
3a
Ph
N
166.45
156.46
140.07
136.97
128.72
128.71
127.07
119.34
117.75
77.16 CDCl3
46.74
33.08
26.73
26.30
f1 (ppm)

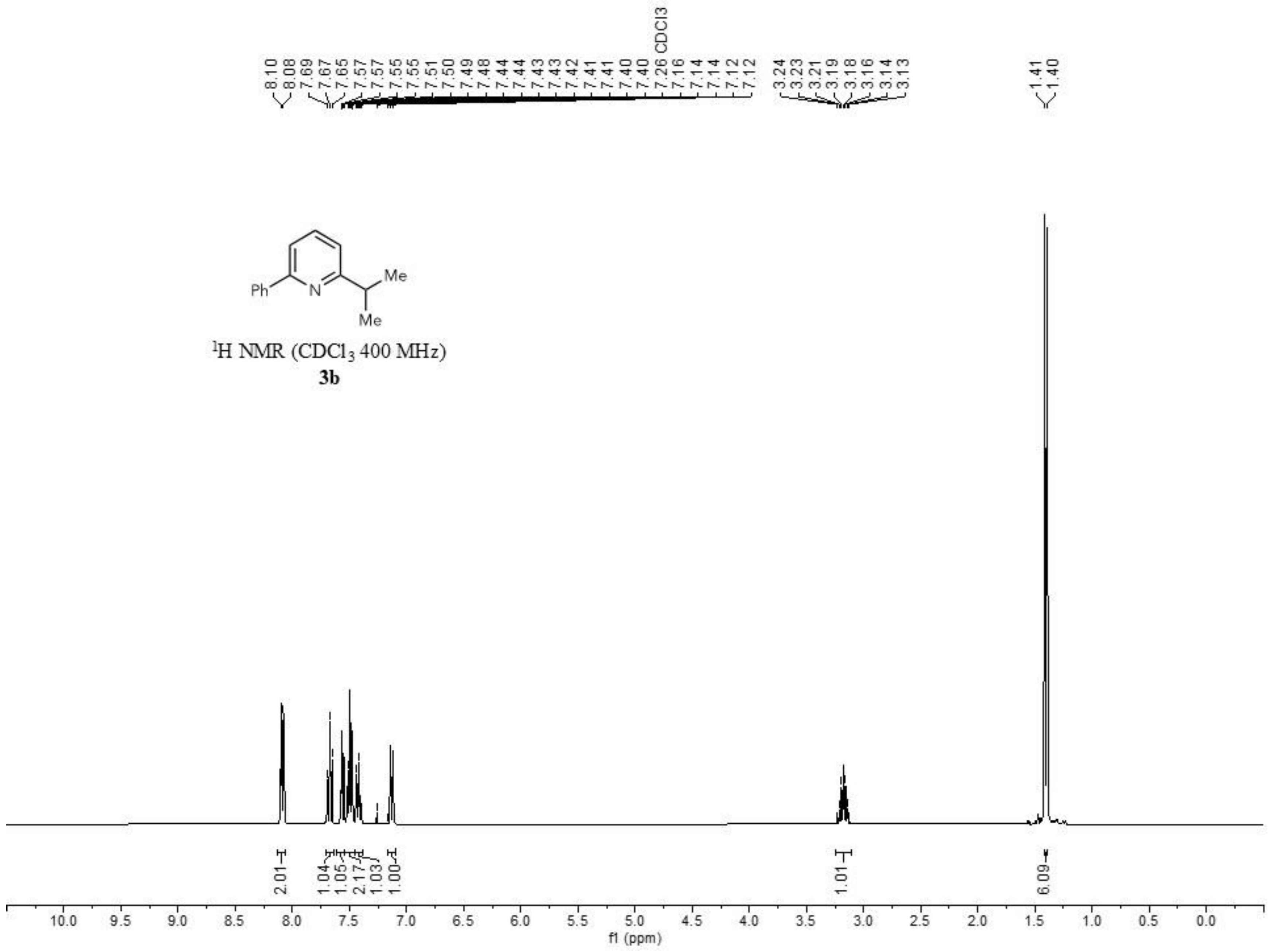
8.10
8.08
7.69
7.67
7.65
7.57
7.57
7.55
7.55
7.51
7.50
7.49
7.48
7.44
7.44
7.43
7.43
7.42
7.41
7.41
7.40
7.40
7.26 CDCl3
7.16
7.14
7.14
7.12
7.12
3.24
3.23
3.21
3.19
3.18
3.16
3.14
3.13
1.41
1.40
Ph
N
Me
Me
^{1}H NMR ($CDCl_3$ 400 MHz)
3b
2.01
1.04
1.05
2.17
1.03
1.00
1.01
6.09
10.0
9.5
9.0
8.5
8.0
7.5
7.0
6.5
6.0
5.5
5.0
4.5
4.0
3.5
3.0
2.5
2.0
1.5
1.0
0.5
0.0
f1 (ppm)

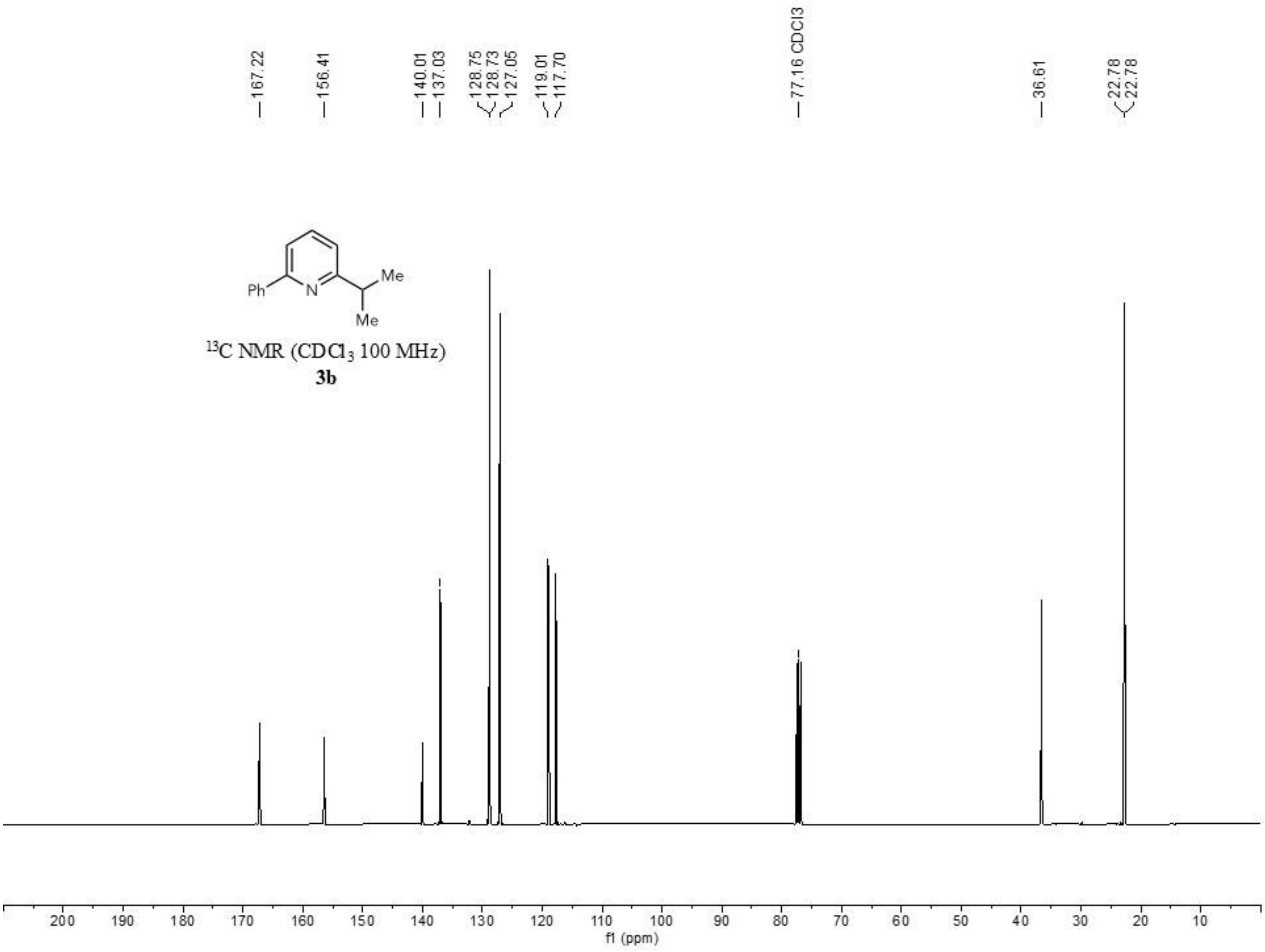

^{13}C NMR ($CDCl_3$ 100 MHz)

3b

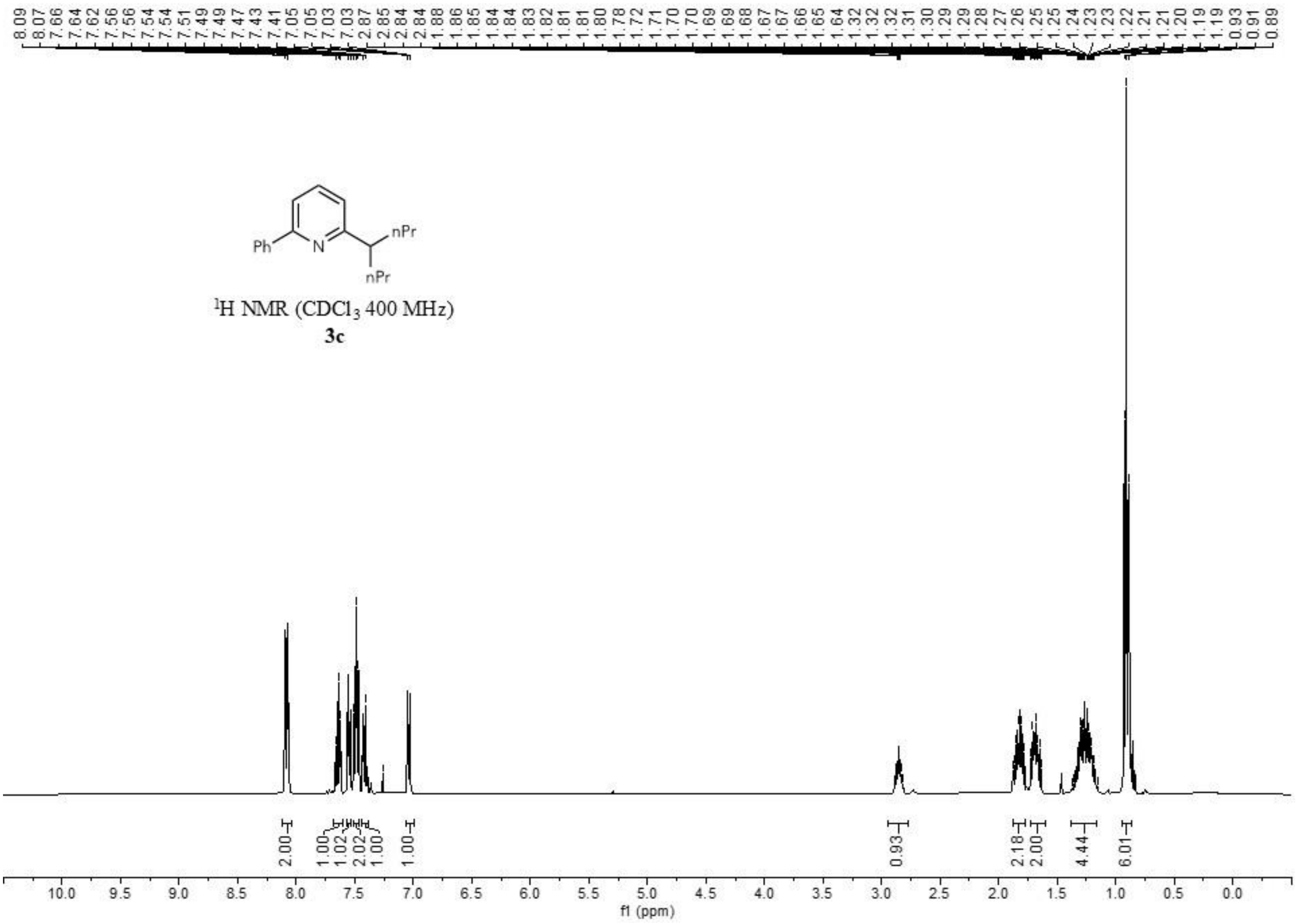
8.09 8.07 7.66 7.64 7.62 7.56 7.56 7.54 7.54 7.51 7.49 7.49 7.47 7.43 7.41 7.05 7.05 7.03 7.03 2.87 2.85 2.84 2.84 1.88 1.86 1.85 1.84 1.84 1.83 1.82 1.81 1.81 1.80 1.78 1.72 1.71 1.70 1.70 1.69 1.69 1.68 1.67 1.67 1.66 1.65 1.64 1.32 1.32 1.32 1.31 1.30 1.29 1.29 1.28 1.27 1.26 1.25 1.25 1.24 1.23 1.23 1.22 1.21 1.21 1.20 1.19 1.19 0.93 0.91 0.89
Ph
N
nPr
nPr
^{1}H NMR (CDCl$_3$ 400 MHz)
3c
2.00
1.00
1.02
2.02
1.00
1.00
0.93
2.18
2.00
4.44
6.01
10.0 9.5 9.0 8.5 8.0 7.5 7.0 6.5 6.0 5.5 5.0 4.5 4.0 3.5 3.0 2.5 2.0 1.5 1.0 0.5 0.0
f1 (ppm)

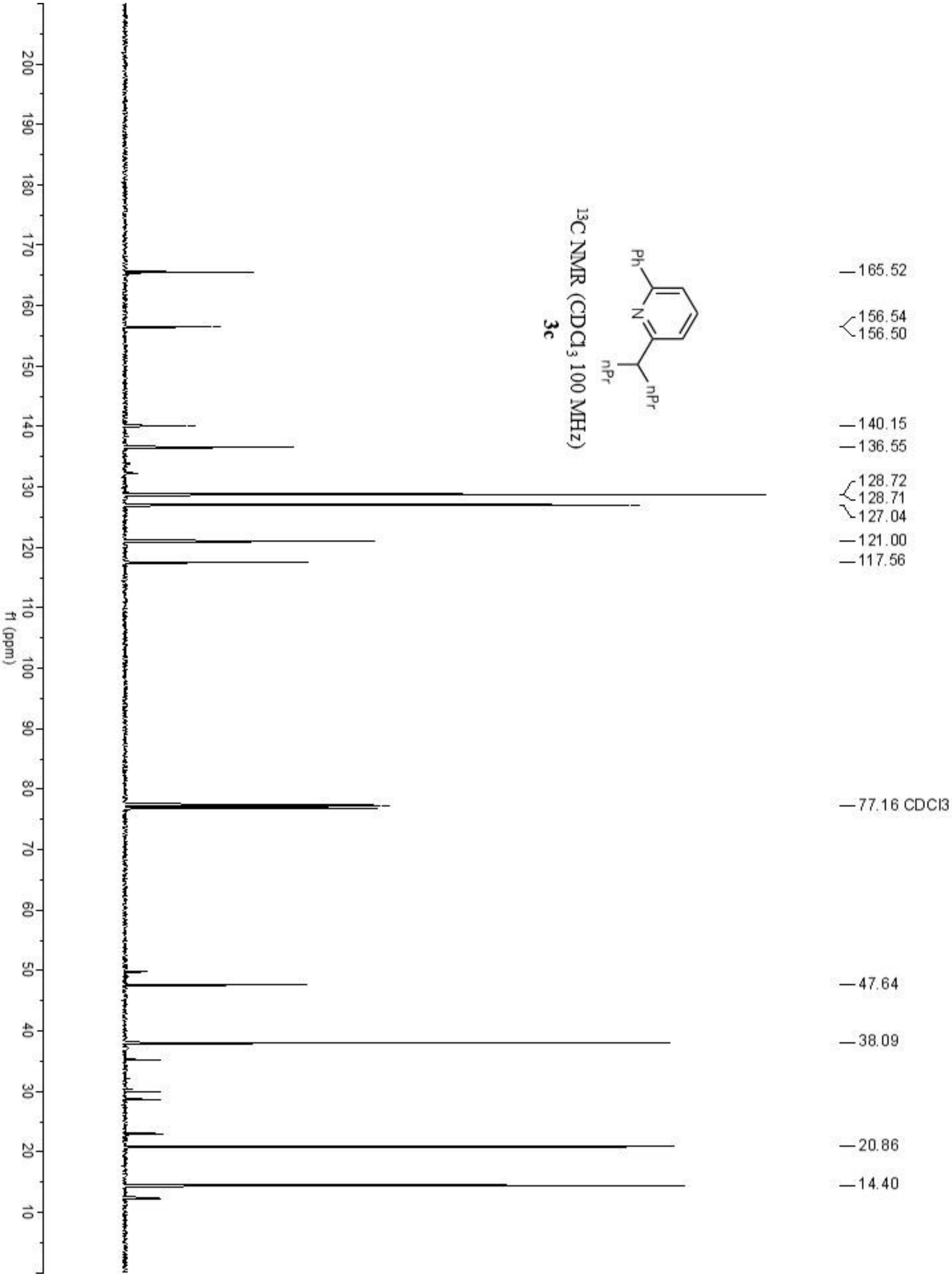
^{13}C NMR (CDCl$_3$ 100 MHz)
3c
Ph
N
nPr
nPr
165.52
156.54
156.50
140.15
136.55
128.72
128.71
127.04
121.00
117.56
77.16 CDCl3
47.64
38.09
20.86
14.40
f1 (ppm)
200
190
180
170
160
150
140
130
120
110
100
90
80
70
60
50
40
30
20
10

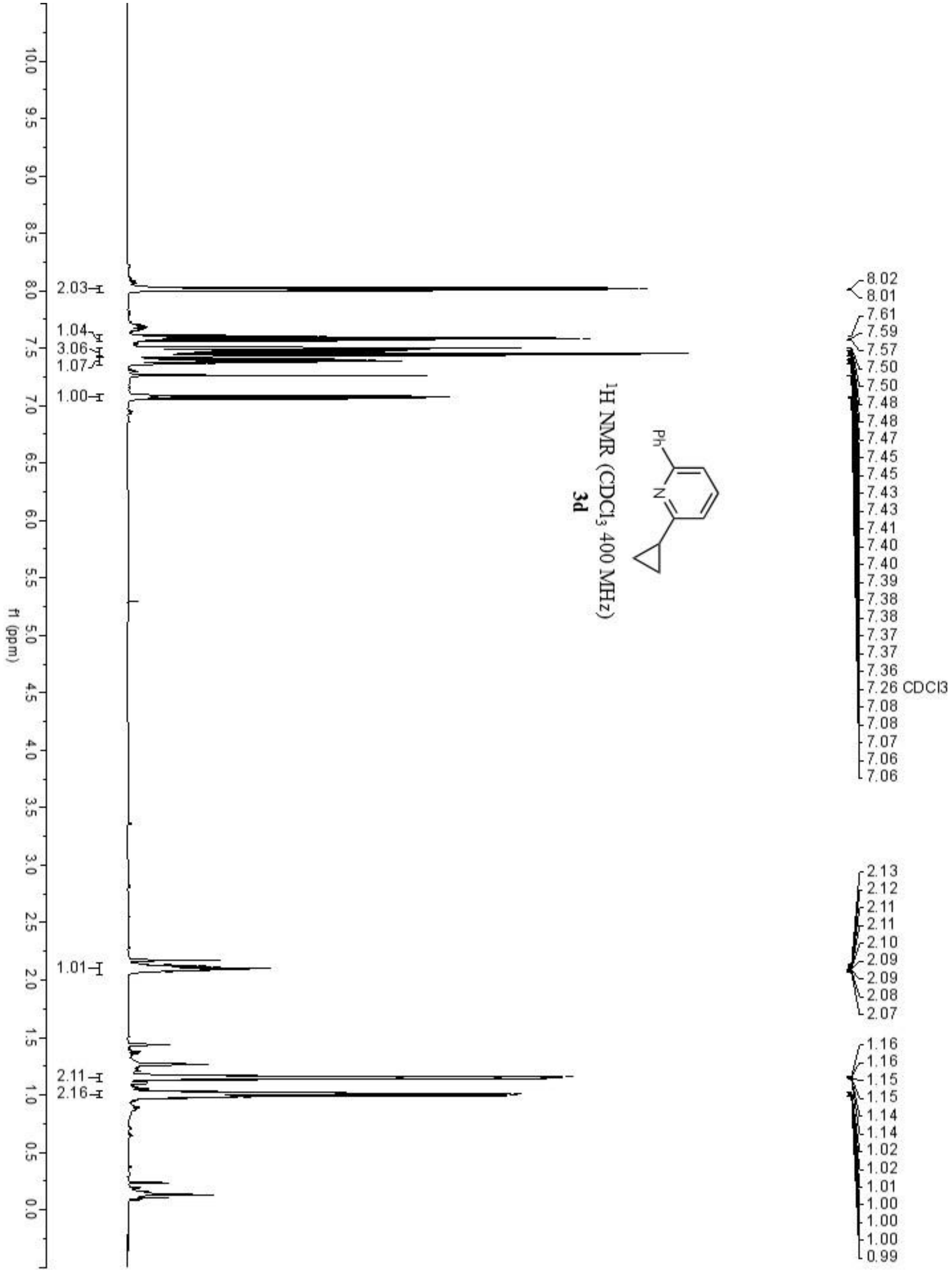
1H NMR (CDCl3 400 MHz)
3d
Ph
N
ppm
f1 (ppm)
8.02
8.01
7.61
7.59
7.57
7.50
7.50
7.48
7.48
7.47
7.45
7.45
7.43
7.43
7.41
7.40
7.40
7.39
7.38
7.38
7.37
7.37
7.36
7.26 CDCl3
7.08
7.08
7.07
7.06
7.06
2.13
2.12
2.11
2.11
2.10
2.09
2.09
2.08
2.07
1.16
1.16
1.15
1.15
1.14
1.14
1.02
1.02
1.01
1.00
1.00
1.00
0.99
2.03
1.04
3.06
1.07
1.00
1.01
2.11
2.16

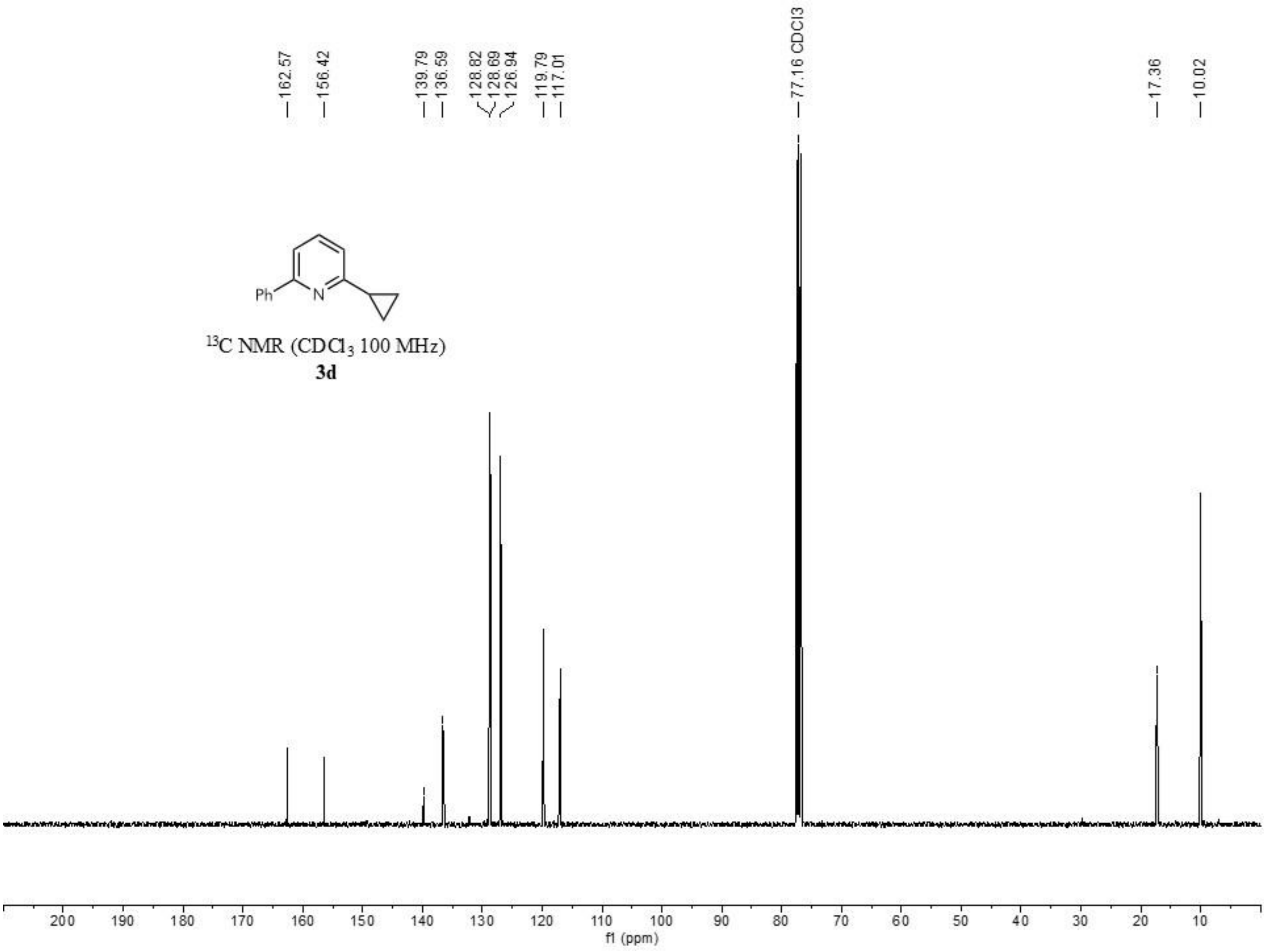

162.57
156.42
139.79
136.59
128.82
128.69
126.94
119.79
117.01
77.16 CDCl3
17.36
10.02
Ph
N
^{13}C NMR (CDCl$_3$ 100 MHz)
3d
200
190
180
170
160
150
140
130
120
110
100
90
80
70
60
50
40
30
20
10
f1 (ppm)

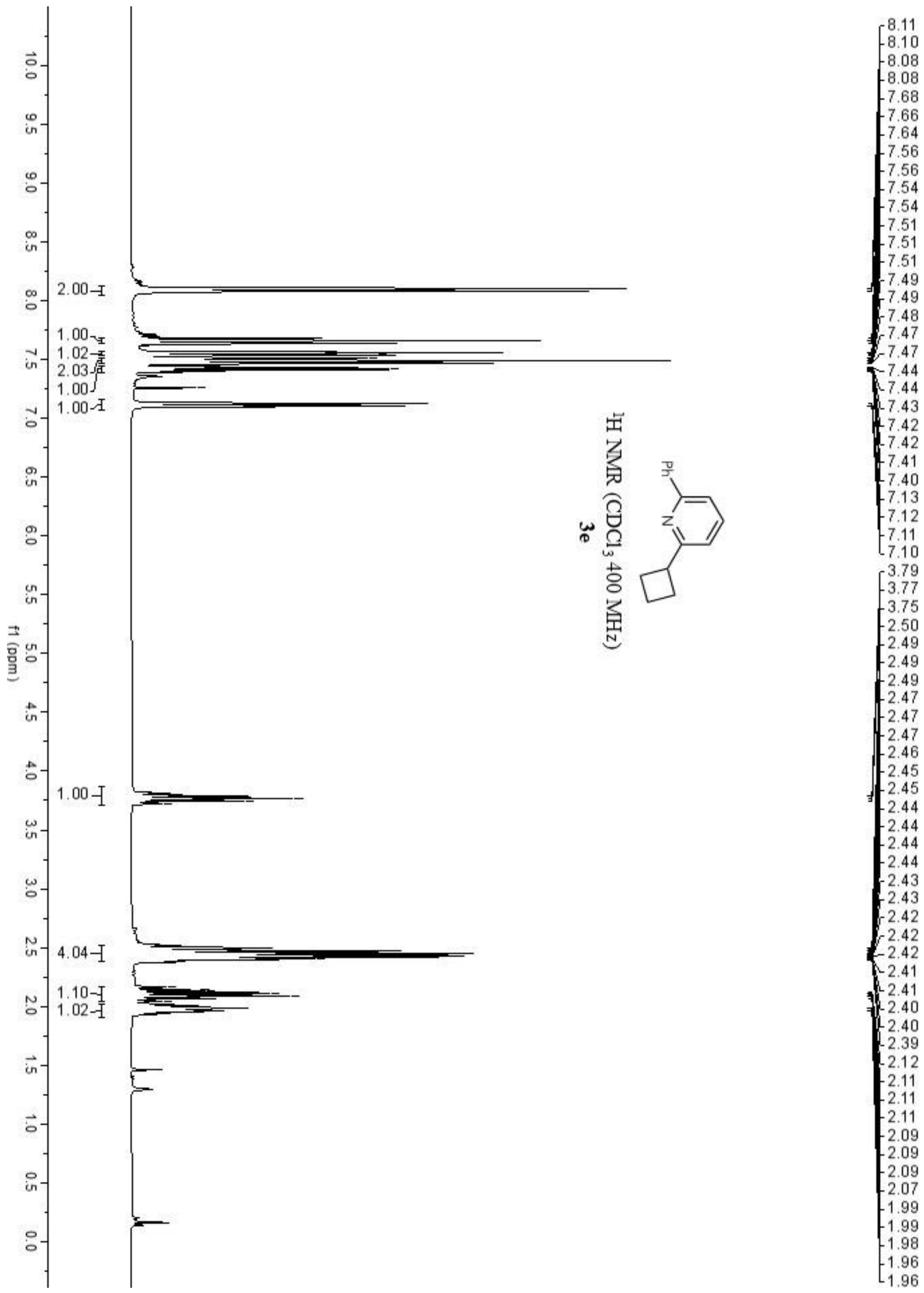

1H NMR (CDCl3 400 MHz)
3e
Ph
N
8.11
8.10
8.08
8.08
7.68
7.66
7.64
7.56
7.56
7.54
7.54
7.51
7.51
7.51
7.49
7.49
7.48
7.47
7.47
7.44
7.44
7.43
7.42
7.42
7.41
7.40
7.13
7.12
7.11
7.10
3.79
3.77
3.75
2.50
2.49
2.49
2.49
2.47
2.47
2.47
2.46
2.45
2.45
2.44
2.44
2.44
2.44
2.43
2.43
2.42
2.42
2.42
2.41
2.41
2.40
2.40
2.39
2.12
2.11
2.11
2.11
2.09
2.09
2.09
2.07
1.99
1.99
1.98
1.96
1.96
2.00
1.00
1.02
2.03
1.00
1.00
1.00
4.04
1.10
1.02
10.0
9.5
9.0
8.5
8.0
7.5
7.0
6.5
6.0
5.5
5.0
4.5
4.0
3.5
3.0
2.5
2.0
1.5
1.0
0.5
0.0
f1 (ppm)

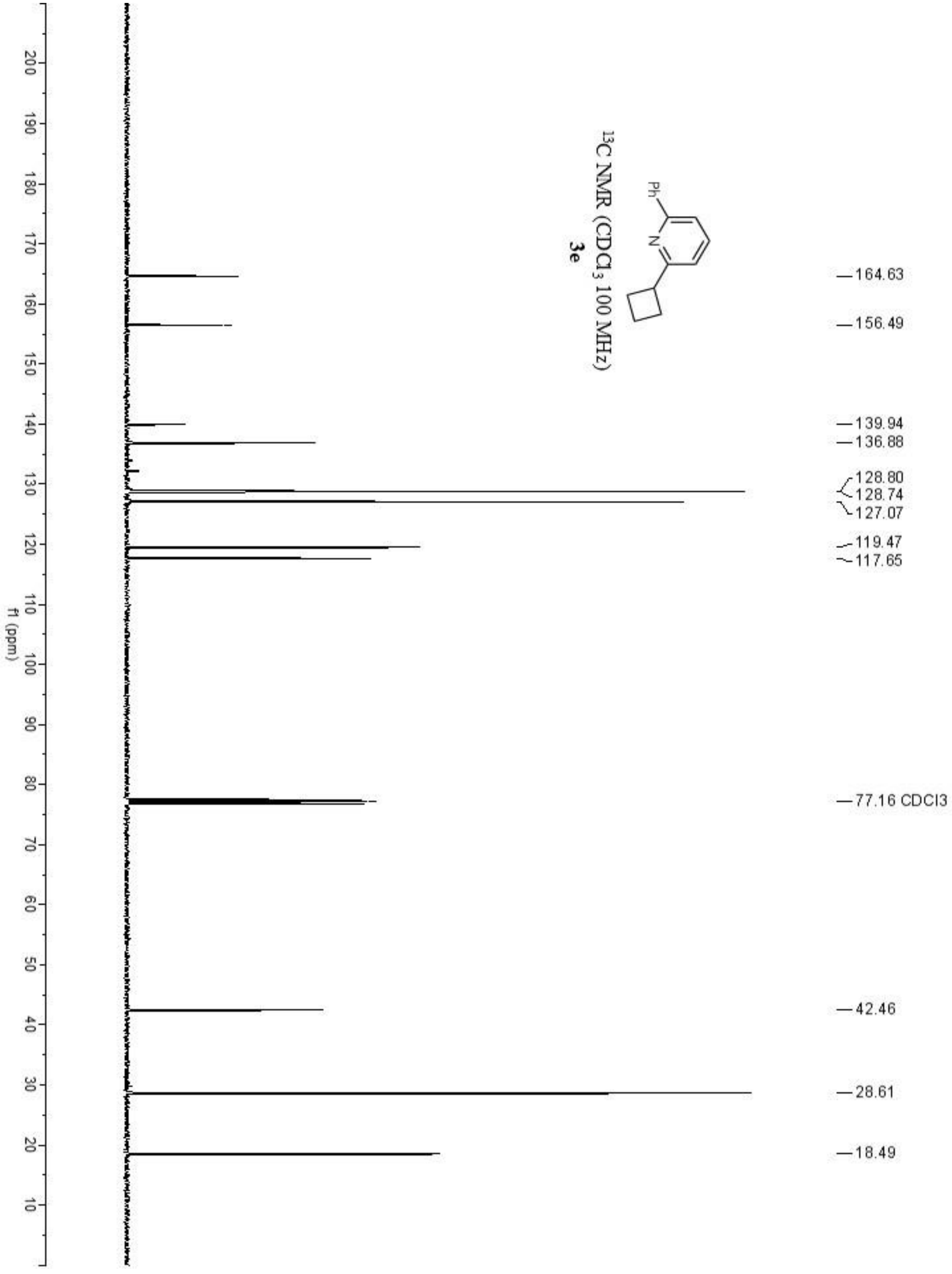

Ph
N
^{13}C NMR (CDCl$_3$ 100 MHz)
3e
164.63
156.49
139.94
136.88
128.80
128.74
127.07
119.47
117.65
77.16 CDCl3
42.46
28.61
18.49
f1 (ppm)
200
190
180
170
160
150
140
130
120
110
100
90
80
70
60
50
40
30
20
10

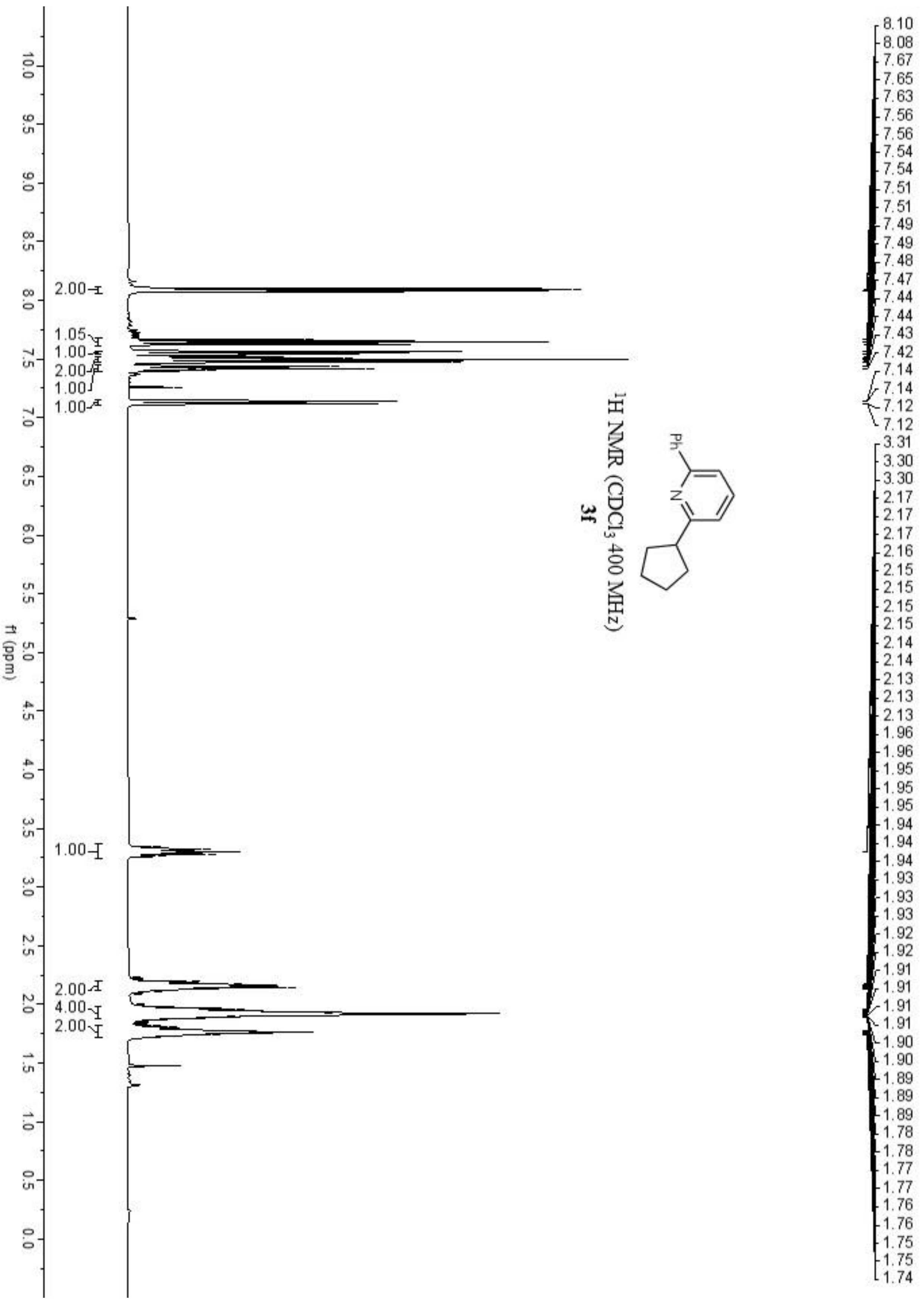

^{1}H NMR (CDCl$_3$ 400 MHz)
3f
Ph
N
8.10
8.08
7.67
7.65
7.63
7.56
7.56
7.54
7.54
7.51
7.51
7.49
7.49
7.48
7.47
7.44
7.44
7.43
7.42
7.14
7.14
7.12
7.12
3.31
3.30
3.30
2.17
2.17
2.17
2.16
2.15
2.15
2.15
2.15
2.14
2.14
2.13
2.13
2.13
1.96
1.96
1.95
1.95
1.95
1.94
1.94
1.94
1.93
1.93
1.93
1.92
1.92
1.91
1.91
1.91
1.91
1.90
1.90
1.89
1.89
1.89
1.78
1.78
1.77
1.77
1.76
1.76
1.75
1.75
1.74
2.00
1.05
1.00
2.00
1.00
1.00
1.00
2.00
4.00
2.00
f1 (ppm)

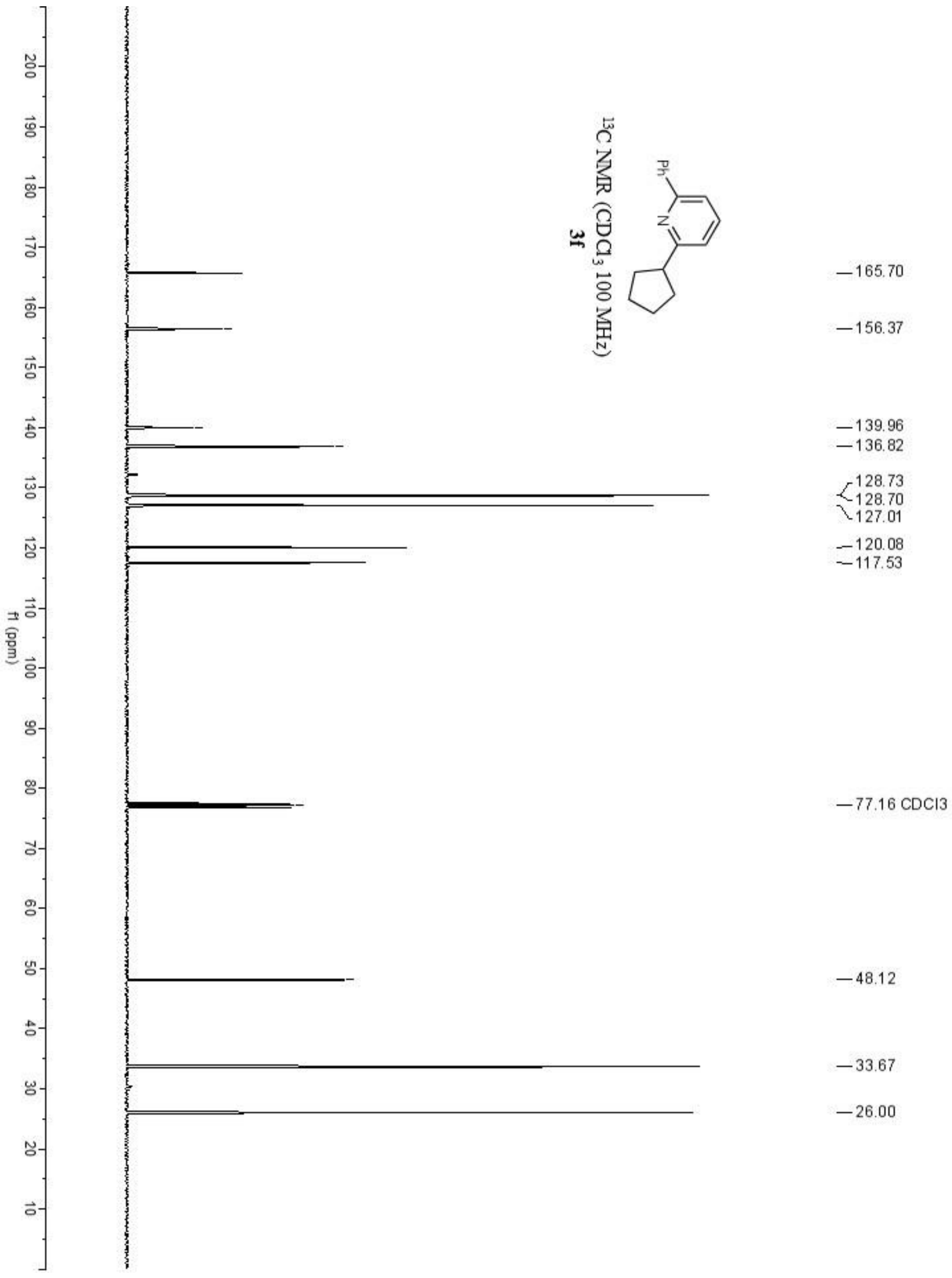

Ph
N
^{13}C NMR (CDCl$_3$ 100 MHz)
3f
165.70
156.37
139.96
136.82
128.73
128.70
127.01
120.08
117.53
77.16 CDCl3
48.12
33.67
26.00
f1 (ppm)
200
190
180
170
160
150
140
130
120
110
100
90
80
70
60
50
40
30
20
10

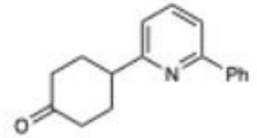

^{1}H NMR ($CDCl_3$ 400 MHz)

3g

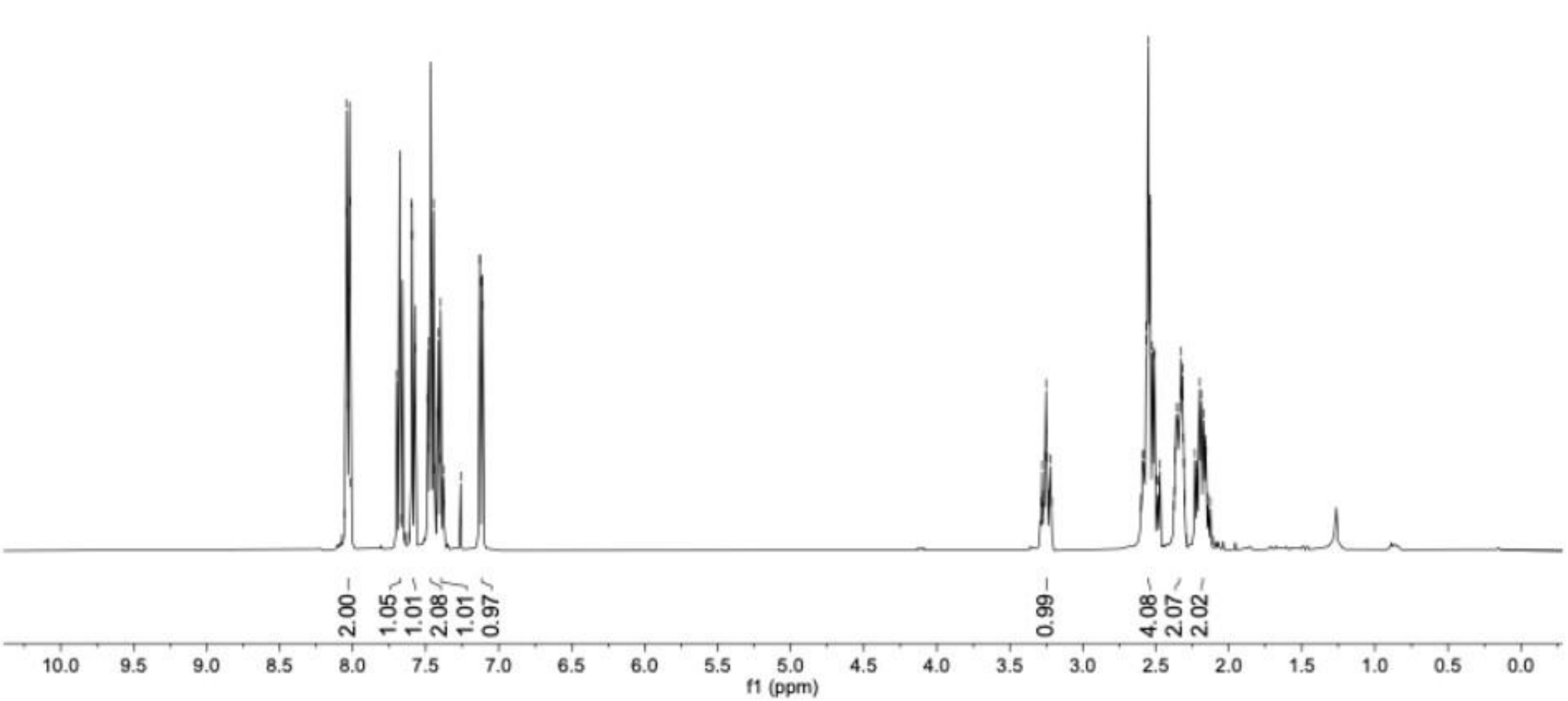

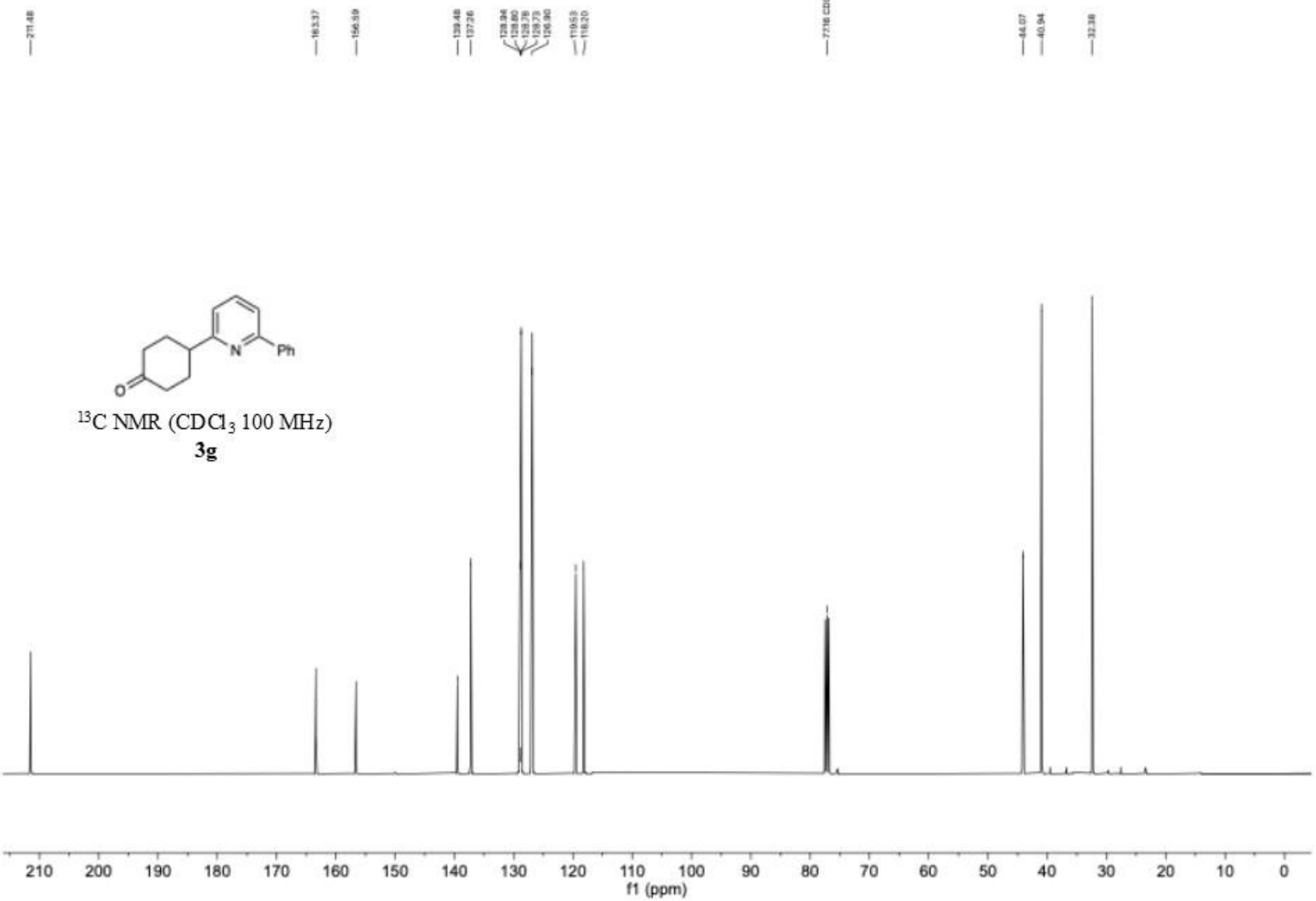

^{13}C NMR ($CDCl_3$ 100 MHz)
3g

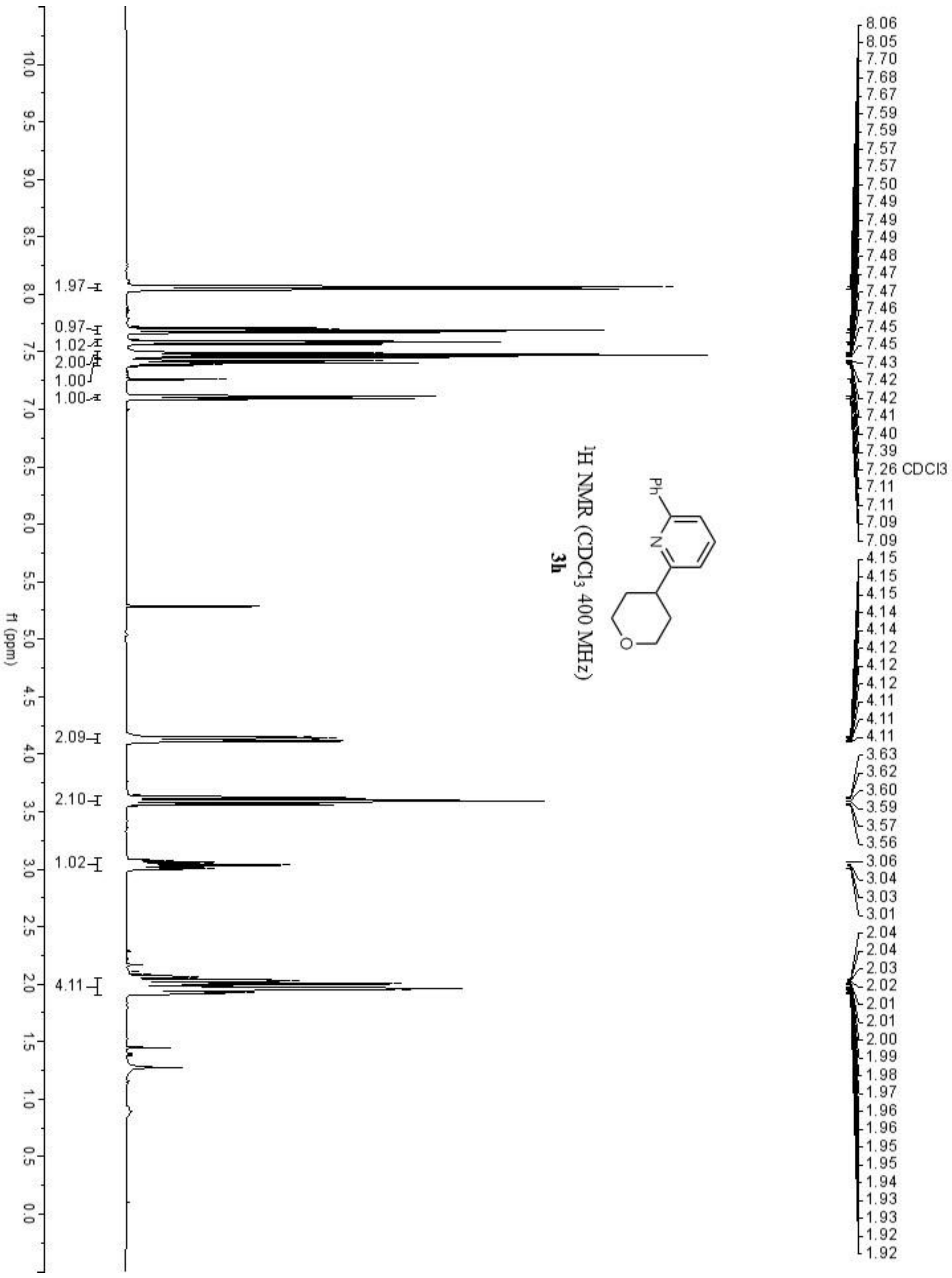

8.06
8.05
7.70
7.68
7.67
7.59
7.59
7.57
7.57
7.50
7.49
7.49
7.49
7.48
7.47
7.47
7.46
7.45
7.45
7.43
7.42
7.42
7.41
7.40
7.39
7.26 CDCl3
7.11
7.11
7.09
7.09
4.15
4.15
4.15
4.14
4.14
4.12
4.12
4.12
4.11
4.11
4.11
3.63
3.62
3.60
3.59
3.57
3.56
3.06
3.04
3.03
3.01
2.04
2.04
2.03
2.02
2.01
2.01
2.00
1.99
1.98
1.97
1.96
1.96
1.95
1.95
1.94
1.93
1.93
1.92
1.92
Ph
N
O
¹H NMR (CDCl3 400 MHz)
3h
1.97
0.97
1.02
2.00
1.00
1.00
2.09
2.10
1.02
4.11
10.0
9.5
9.0
8.5
8.0
7.5
7.0
6.5
6.0
5.5
5.0
4.5
4.0
3.5
3.0
2.5
2.0
1.5
1.0
0.5
0.0
f1 (ppm)

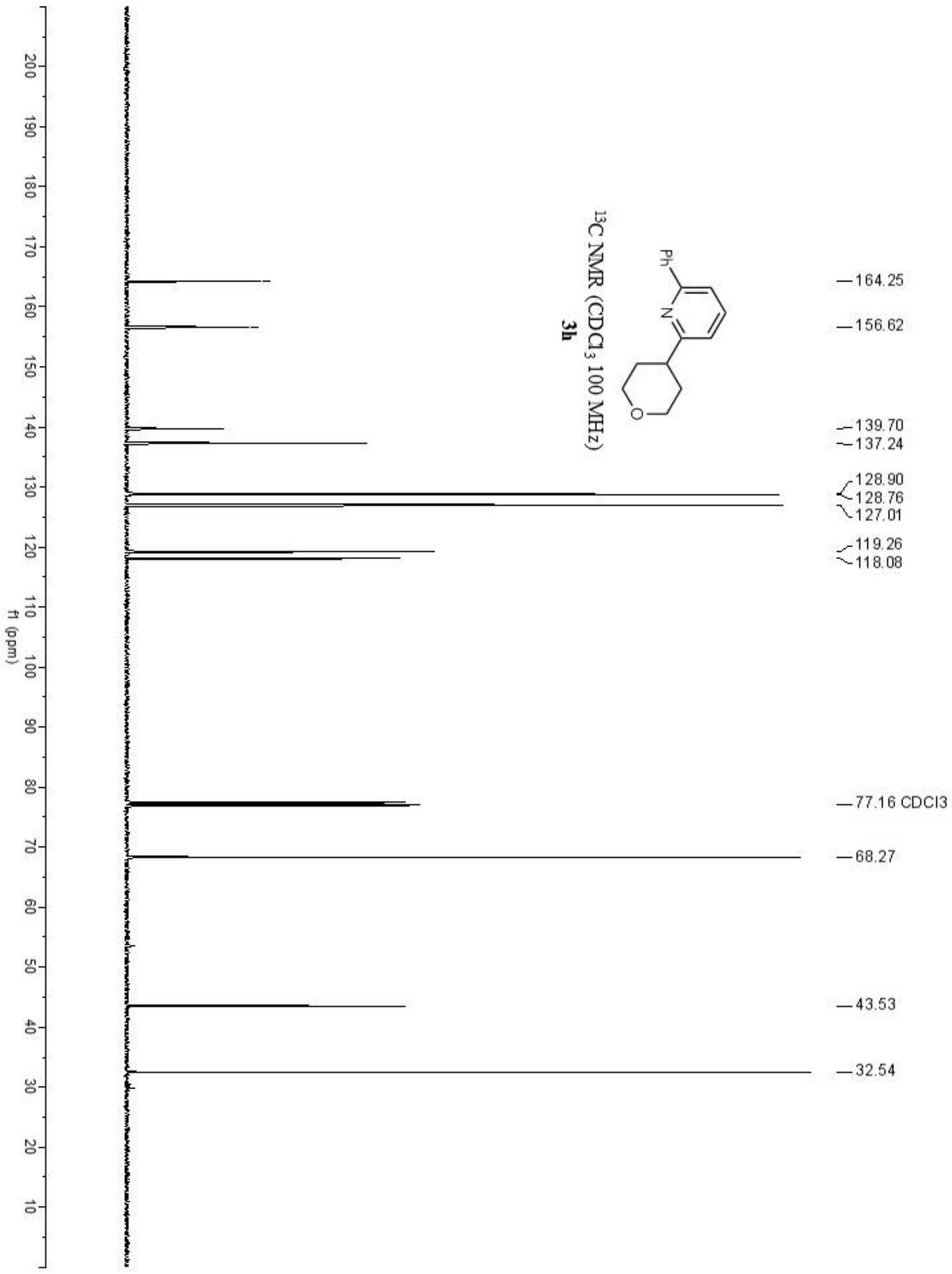
13C NMR (CDCl3 100 MHz)
3h
Ph
N
O
164.25
156.62
139.70
137.24
128.90
128.76
127.01
119.26
118.08
77.16 CDCl3
68.27
43.53
32.54
f1 (ppm)

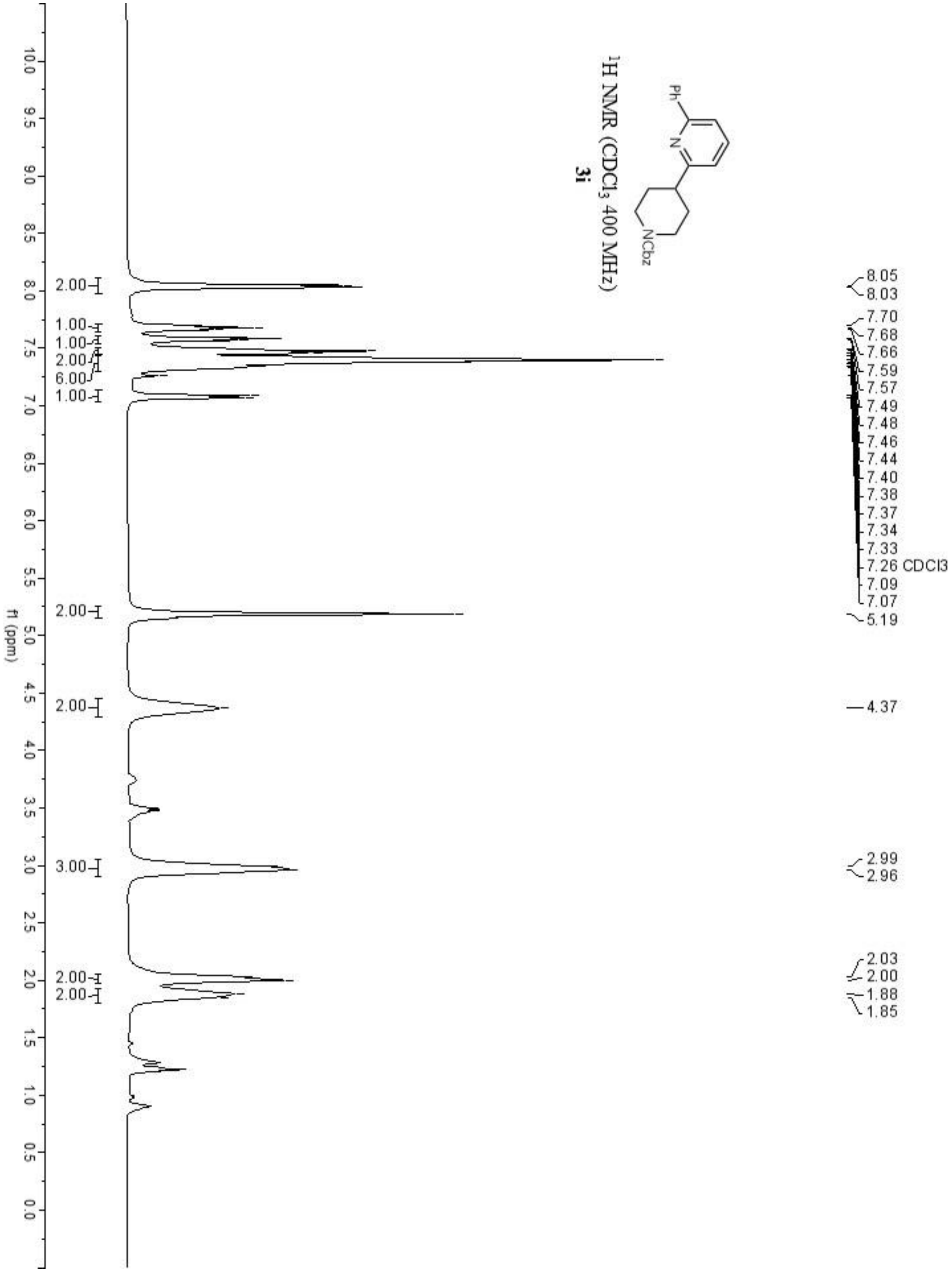
1H NMR (CDCl3 400 MHz)
3i
Ph
N
NCbz
8.05
8.03
7.70
7.68
7.66
7.59
7.57
7.49
7.48
7.46
7.44
7.40
7.38
7.37
7.34
7.33
7.26 CDCl3
7.09
7.07
5.19
4.37
2.99
2.96
2.03
2.00
1.88
1.85
f1 (ppm)

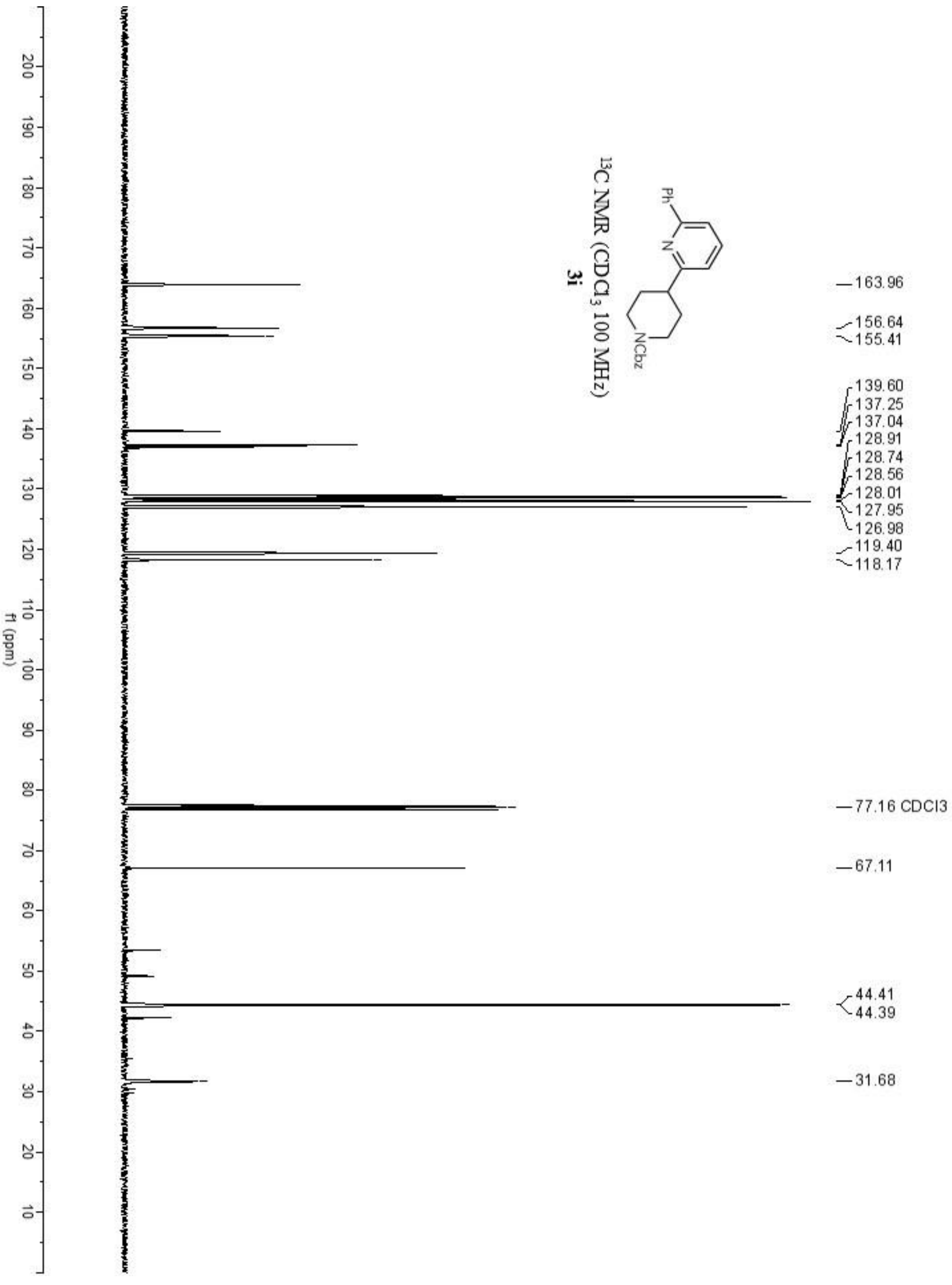

13C NMR (CDCl3 100 MHz)
3i
Ph
N
NCbz
163.96
156.64
155.41
139.60
137.25
137.04
128.91
128.74
128.56
128.01
127.95
126.98
119.40
118.17
77.16 CDCl3
67.11
44.41
44.39
31.68
f1 (ppm)

Crude ^{1}H NMR ($CDCl_3$ 400 MHz)

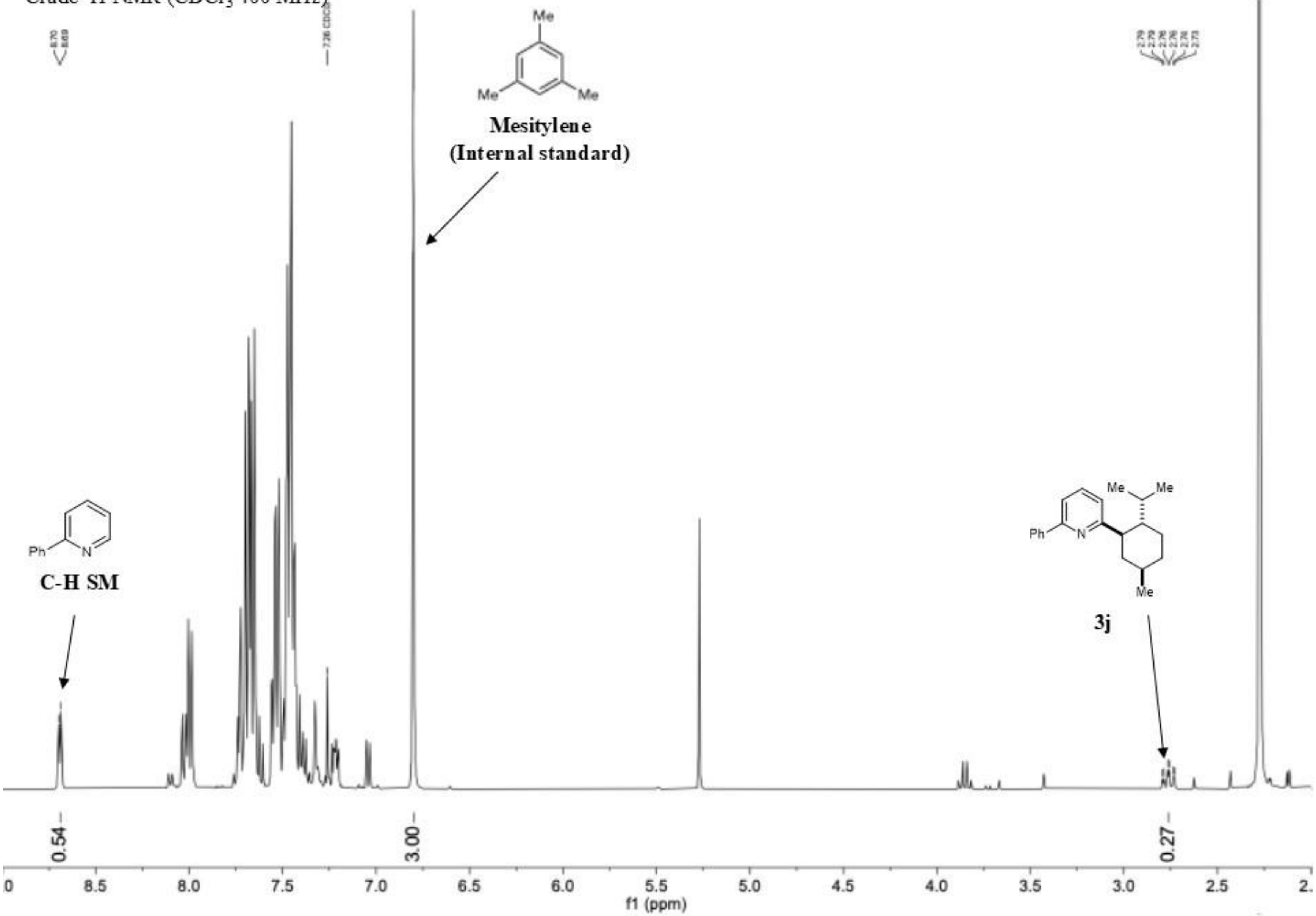

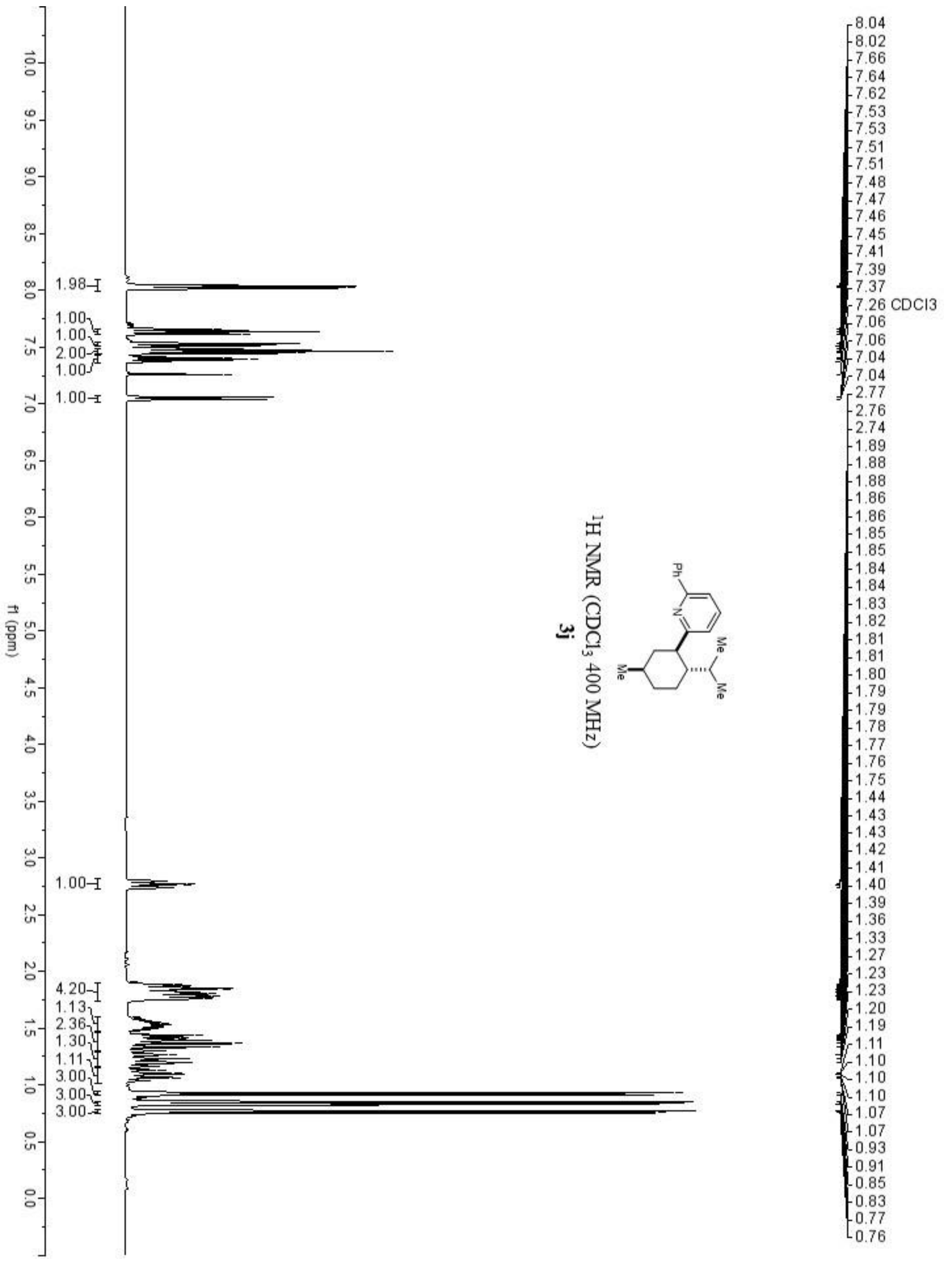

^{1}H NMR (CDCl$_3$ 400 MHz)
3j
Ph
N
Me
Me
Me
8.04
8.02
7.66
7.64
7.62
7.53
7.53
7.51
7.51
7.48
7.47
7.46
7.45
7.41
7.39
7.37
7.26 CDCl3
7.06
7.06
7.04
7.04
2.77
2.76
2.74
1.89
1.88
1.88
1.86
1.86
1.85
1.85
1.84
1.84
1.83
1.82
1.81
1.81
1.80
1.79
1.79
1.78
1.77
1.76
1.75
1.44
1.43
1.43
1.42
1.41
1.40
1.39
1.36
1.33
1.27
1.23
1.23
1.20
1.19
1.11
1.10
1.10
1.10
1.07
1.07
0.93
0.91
0.85
0.83
0.77
0.76
1.98
1.00
1.00
2.00
1.00
1.00
1.00
4.20
1.13
2.36
1.30
1.11
3.00
3.00
3.00
f1 (ppm)

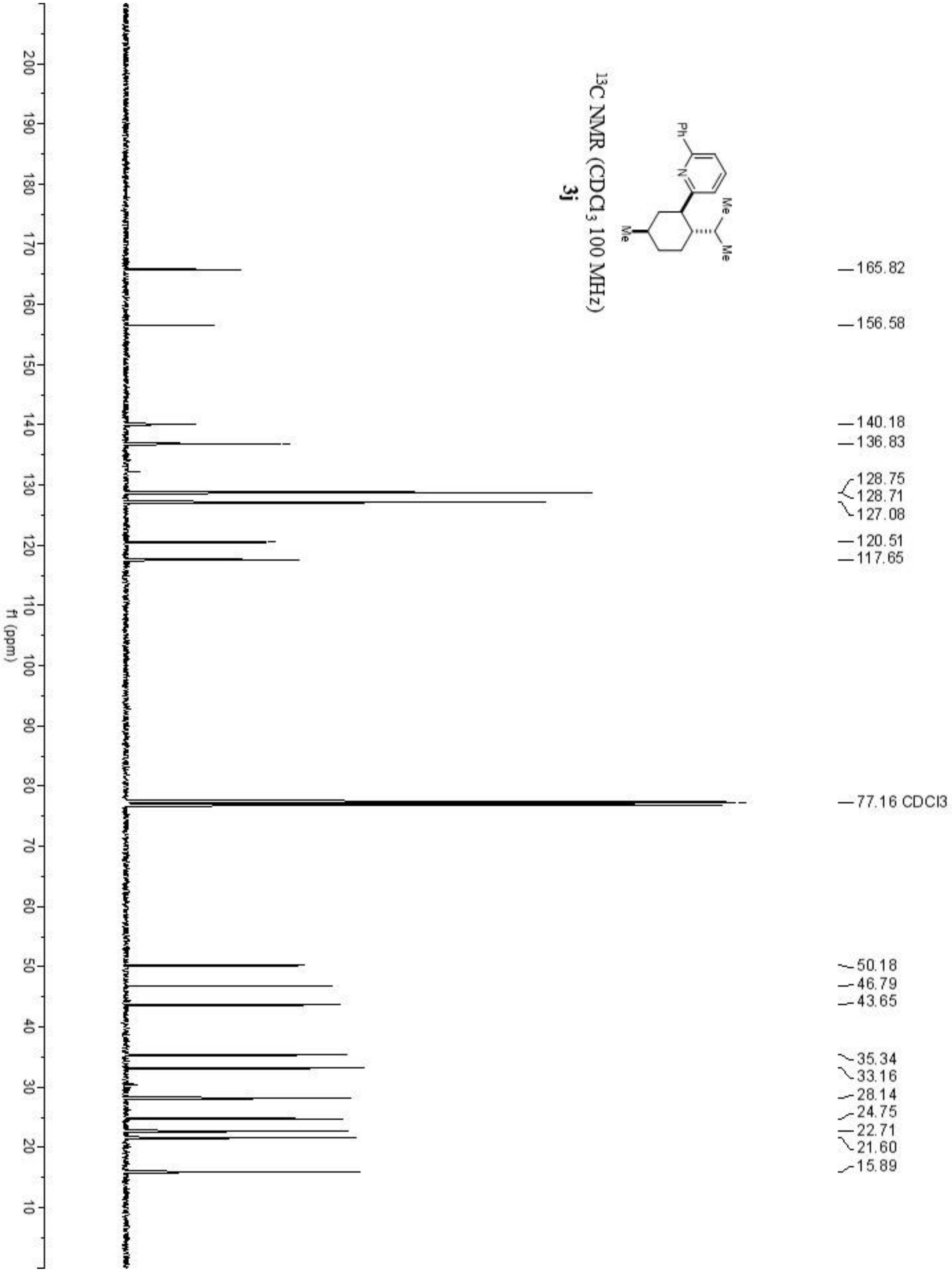

13C NMR (CDCl3 100 MHz)
3j
Ph
N
Me
Me
Me
165.82
156.58
140.18
136.83
128.75
128.71
127.08
120.51
117.65
77.16 CDCl3
50.18
46.79
43.65
35.34
33.16
28.14
24.75
22.71
21.60
15.89
f1 (ppm)
200
190
180
170
160
150
140
130
120
110
100
90
80
70
60
50
40
30
20
10

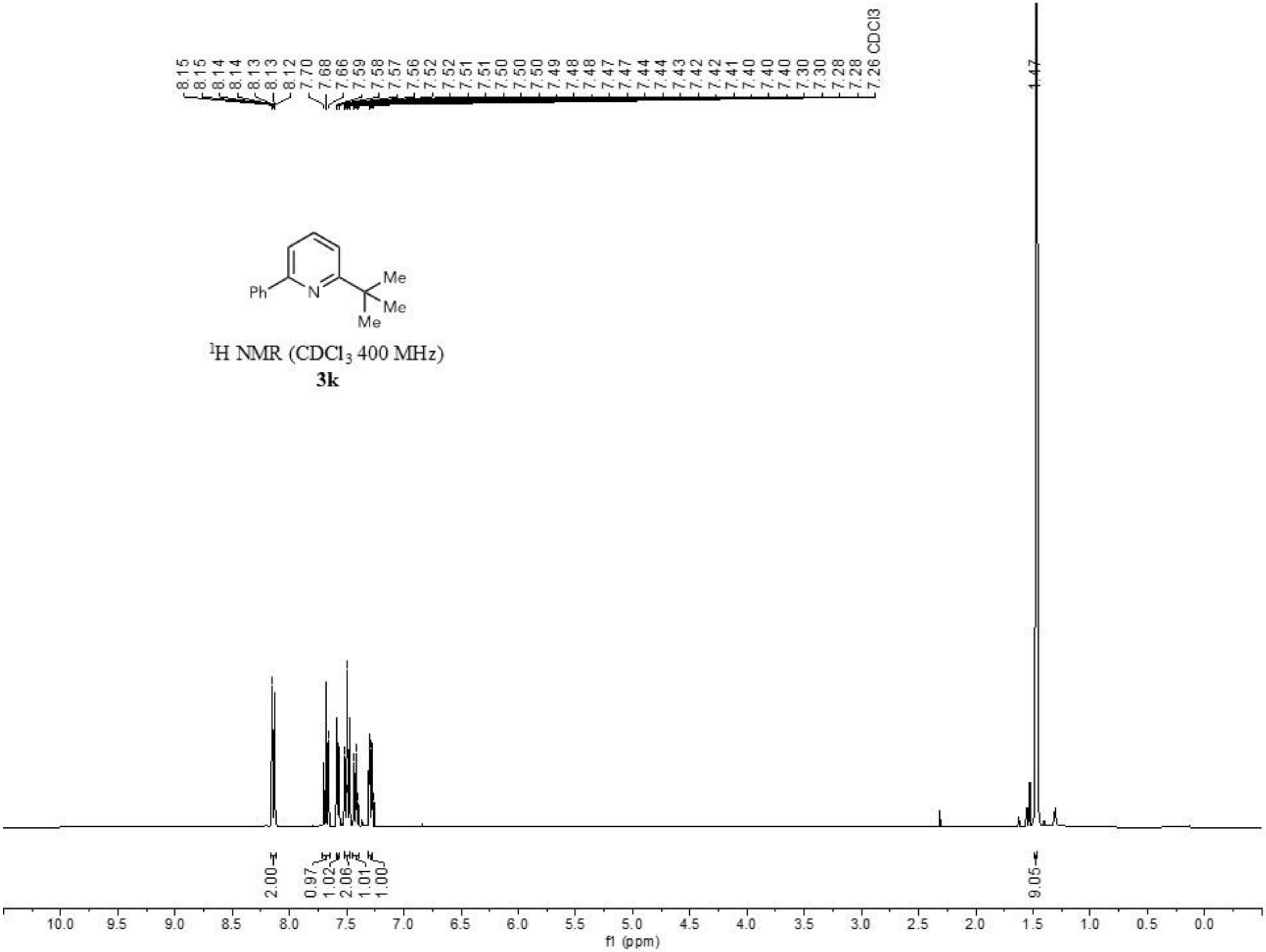

^{1}H NMR ($CDCl_3$ 400 MHz)

3k

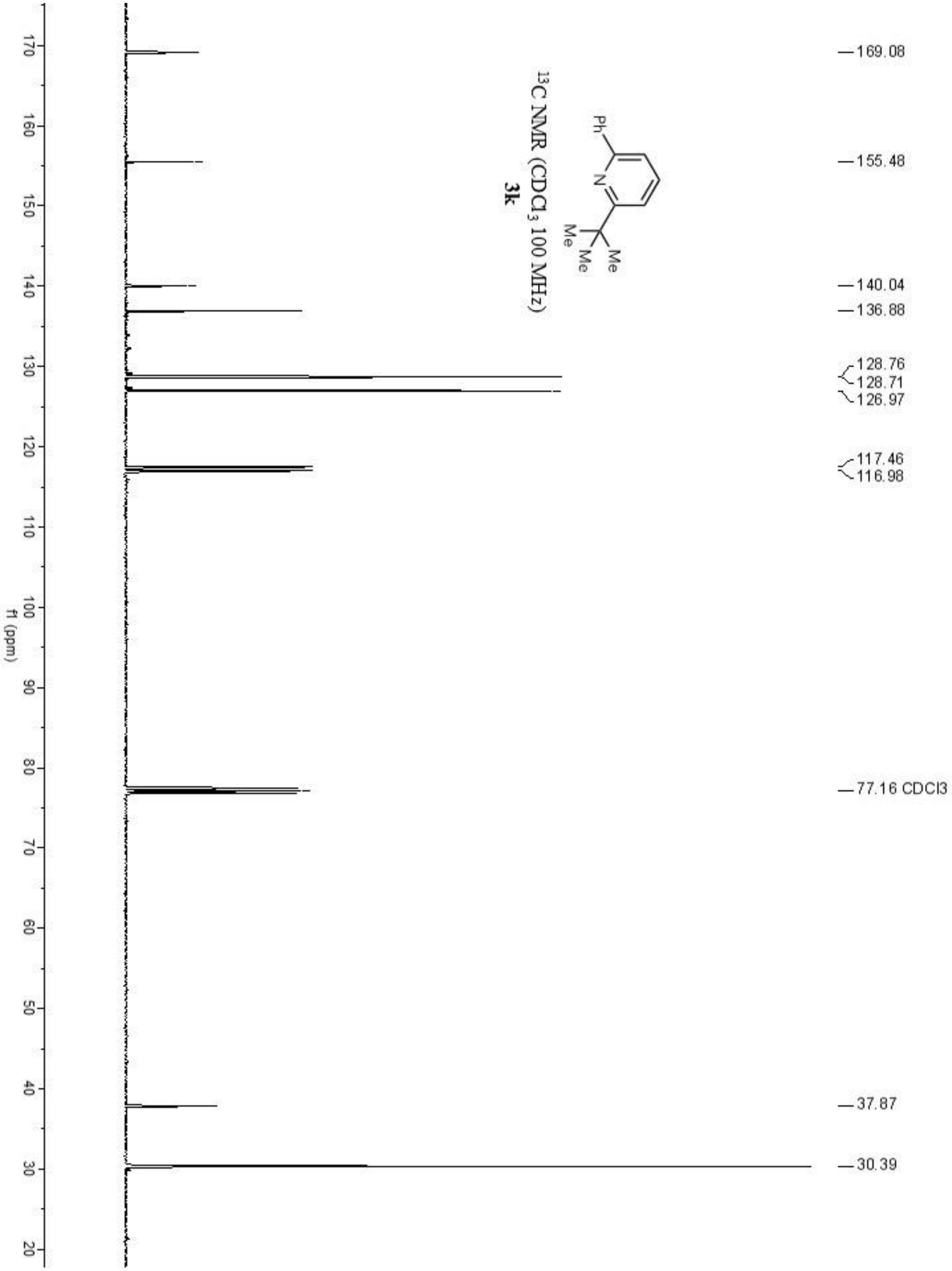

^{13}C NMR (CDCl$_3$ 100 MHz)
3k
Ph
N
Me
Me
Me
169.08
155.48
140.04
136.88
128.76
128.71
126.97
117.46
116.98
77.16 CDCl3
37.87
30.39
f1 (ppm)
170
160
150
140
130
120
110
100
90
80
70
60
50
40
30
20

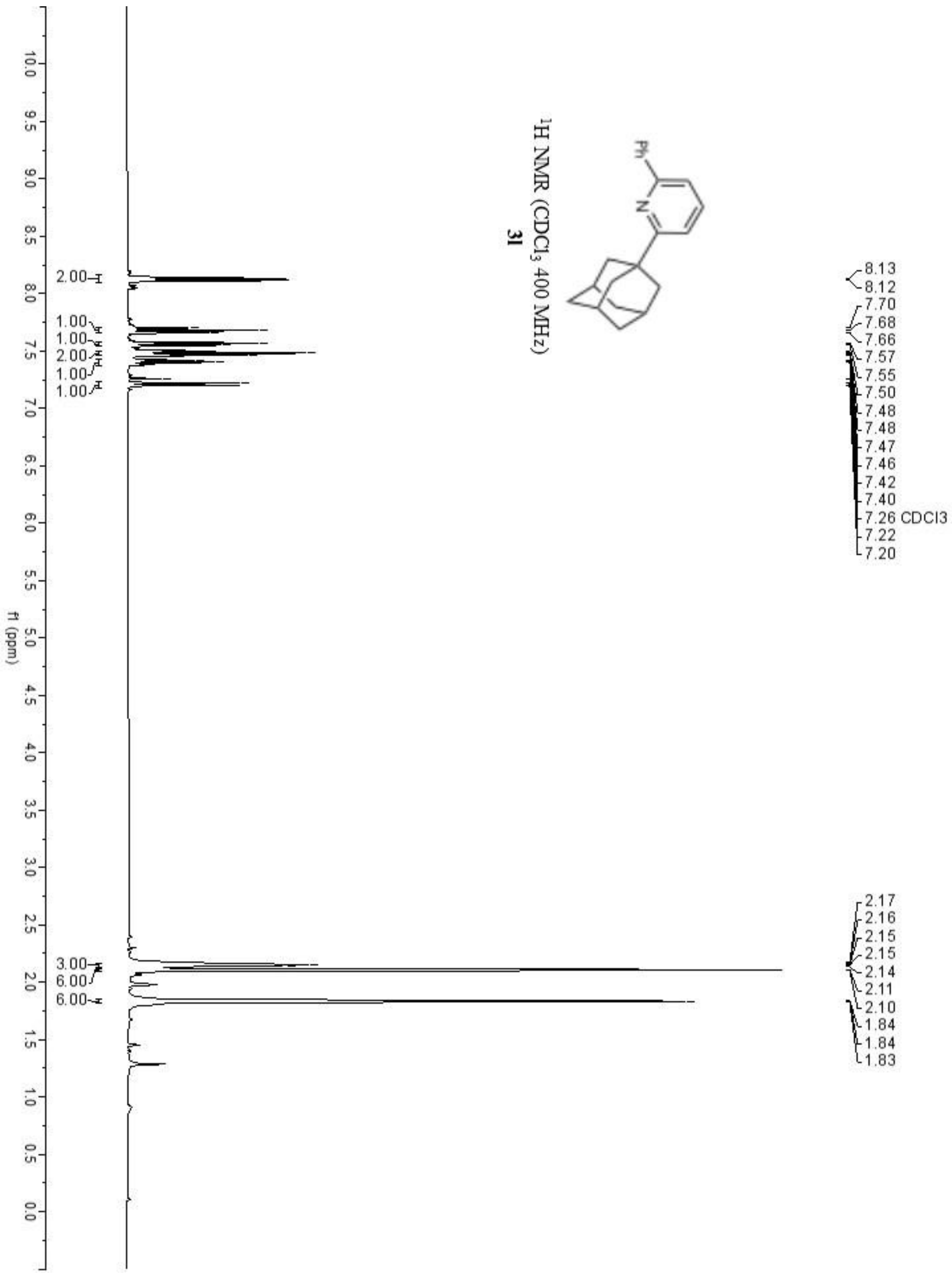
Ph
N
1H NMR (CDCl3 400 MHz)
3l
8.13
8.12
7.70
7.68
7.66
7.57
7.55
7.50
7.48
7.48
7.47
7.46
7.42
7.40
7.26 CDCl3
7.22
7.20
2.17
2.16
2.15
2.15
2.14
2.11
2.10
1.84
1.84
1.83
2.00
1.00
1.00
2.00
1.00
1.00
3.00
6.00
6.00
10.0
9.5
9.0
8.5
8.0
7.5
7.0
6.5
6.0
5.5
5.0
4.5
4.0
3.5
3.0
2.5
2.0
1.5
1.0
0.5
0.0
f1 (ppm)

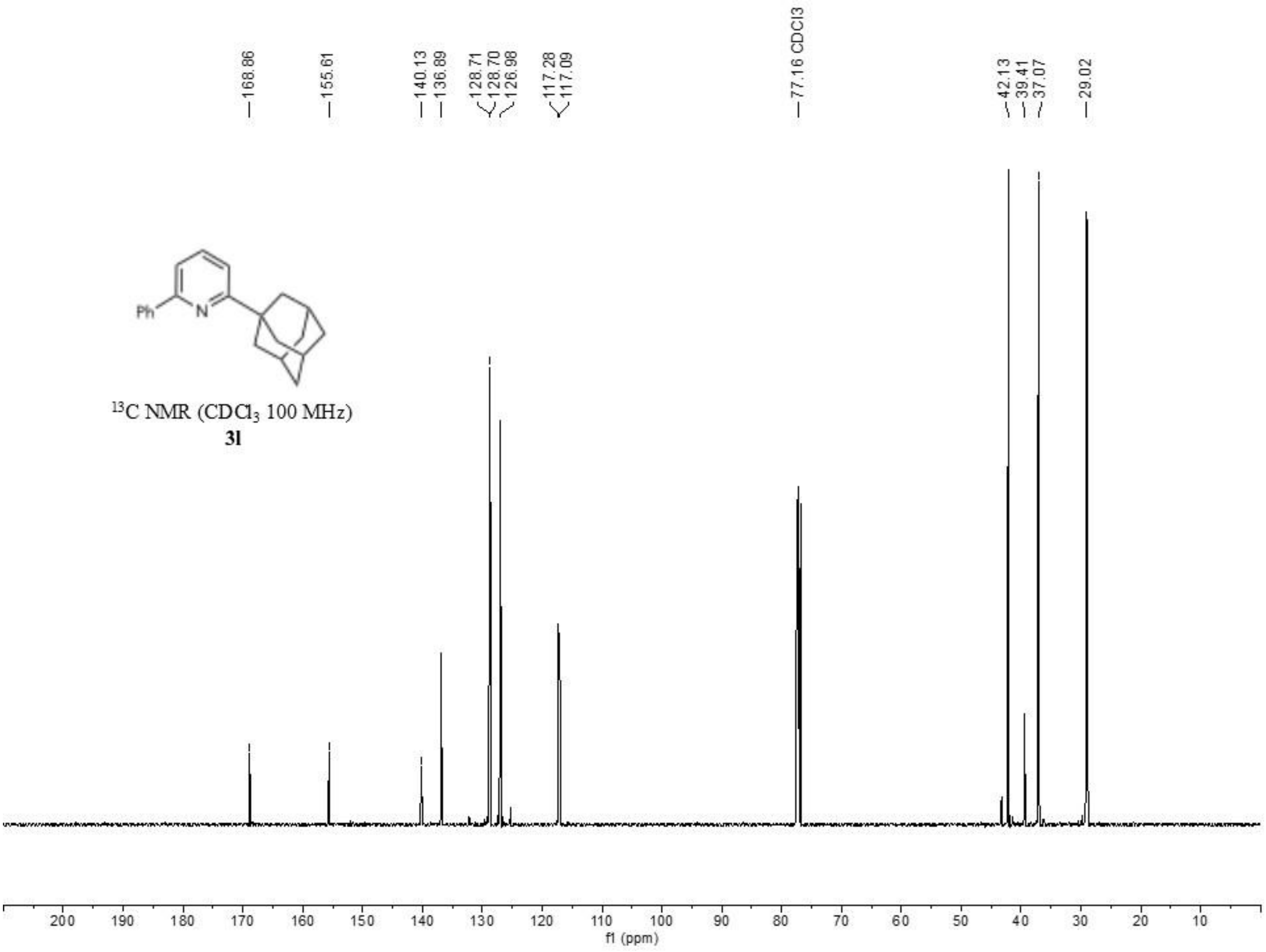

^{13}C NMR ($CDCl_3$ 100 MHz)
3l

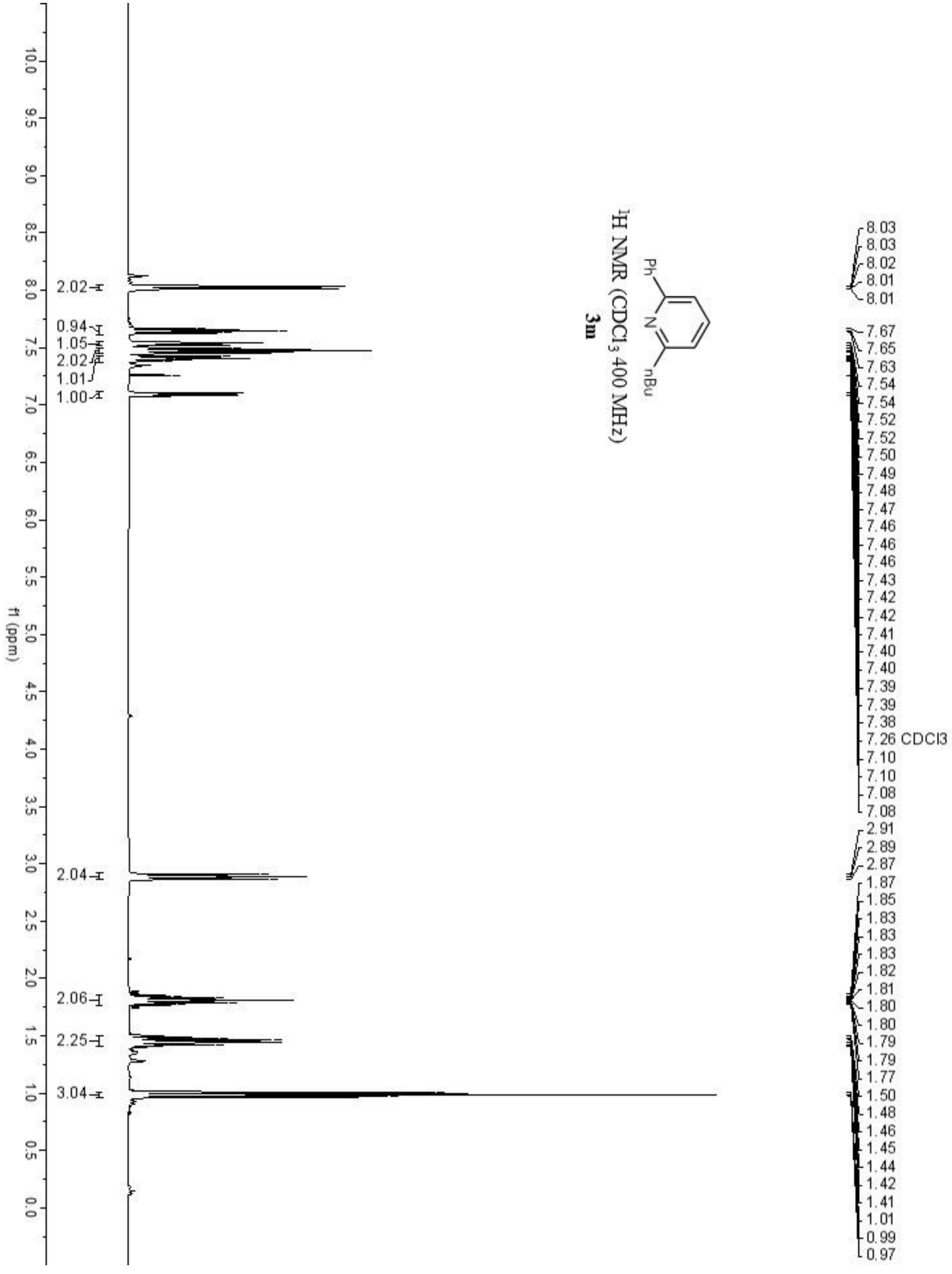

Ph
N
nBu
1H NMR (CDCl3 400 MHz)
3m
8.03 8.03 8.02 8.01 8.01
7.67 7.65 7.63 7.54 7.54 7.52 7.52 7.50 7.49 7.48 7.47 7.46 7.46 7.46 7.43 7.42 7.42 7.41 7.40 7.40 7.39 7.39 7.38 7.26 CDCl3 7.10 7.10 7.08 7.08
2.91 2.89 2.87
1.87 1.85 1.83 1.83 1.83 1.82 1.81 1.80 1.80 1.79 1.79 1.77
1.50 1.48 1.46 1.45 1.44 1.42 1.41
1.01 0.99 0.97
2.02
0.94
1.05
2.02
1.01
1.00
2.04
2.06
2.25
3.04
f1 (ppm)

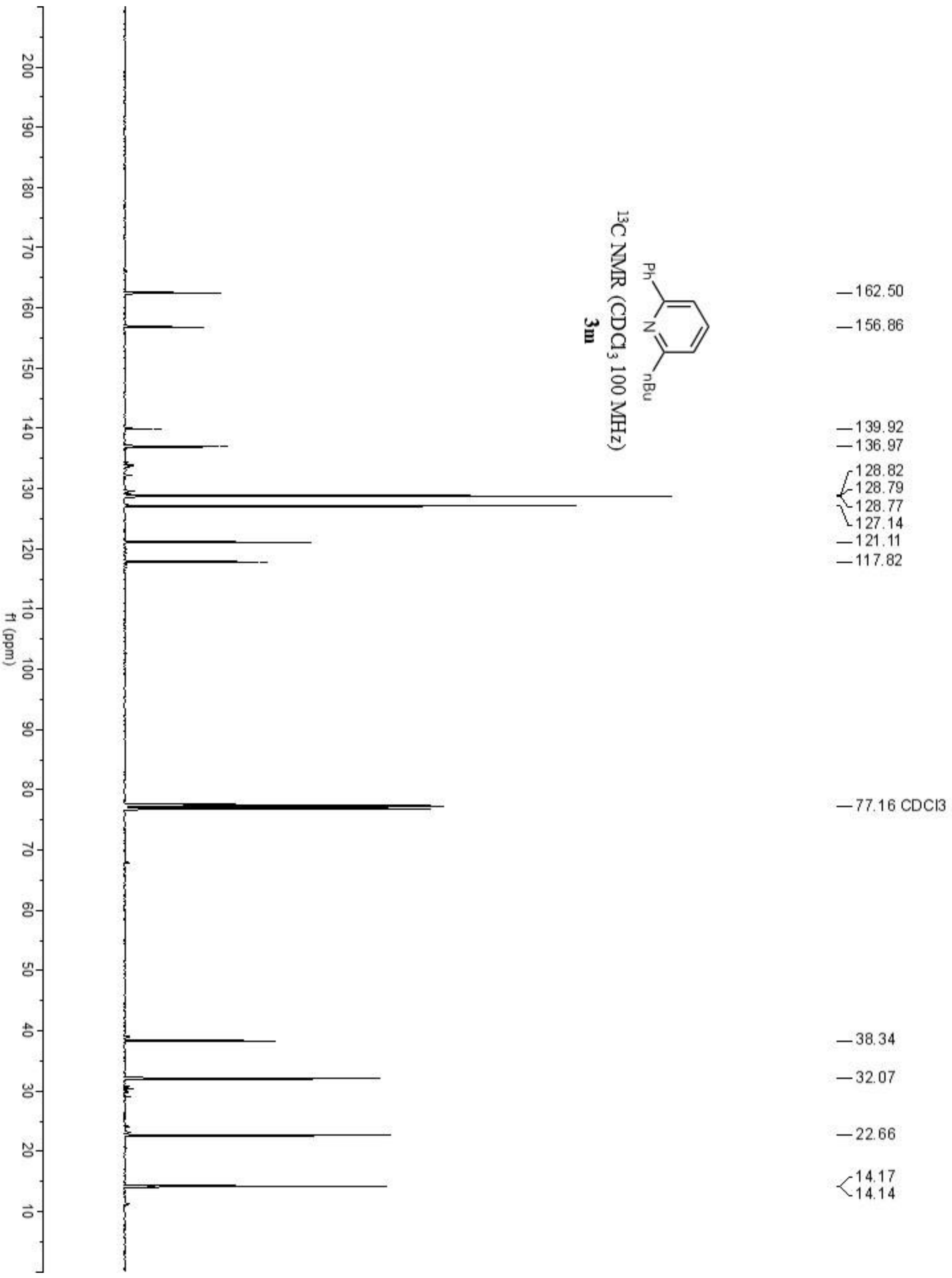

Ph
N
nBu
13C NMR (CDCl3 100 MHz)
3m
162.50
156.86
139.92
136.97
128.82
128.79
128.77
127.14
121.11
117.82
77.16 CDCl3
38.34
32.07
22.66
14.17
14.14
f1 (ppm)

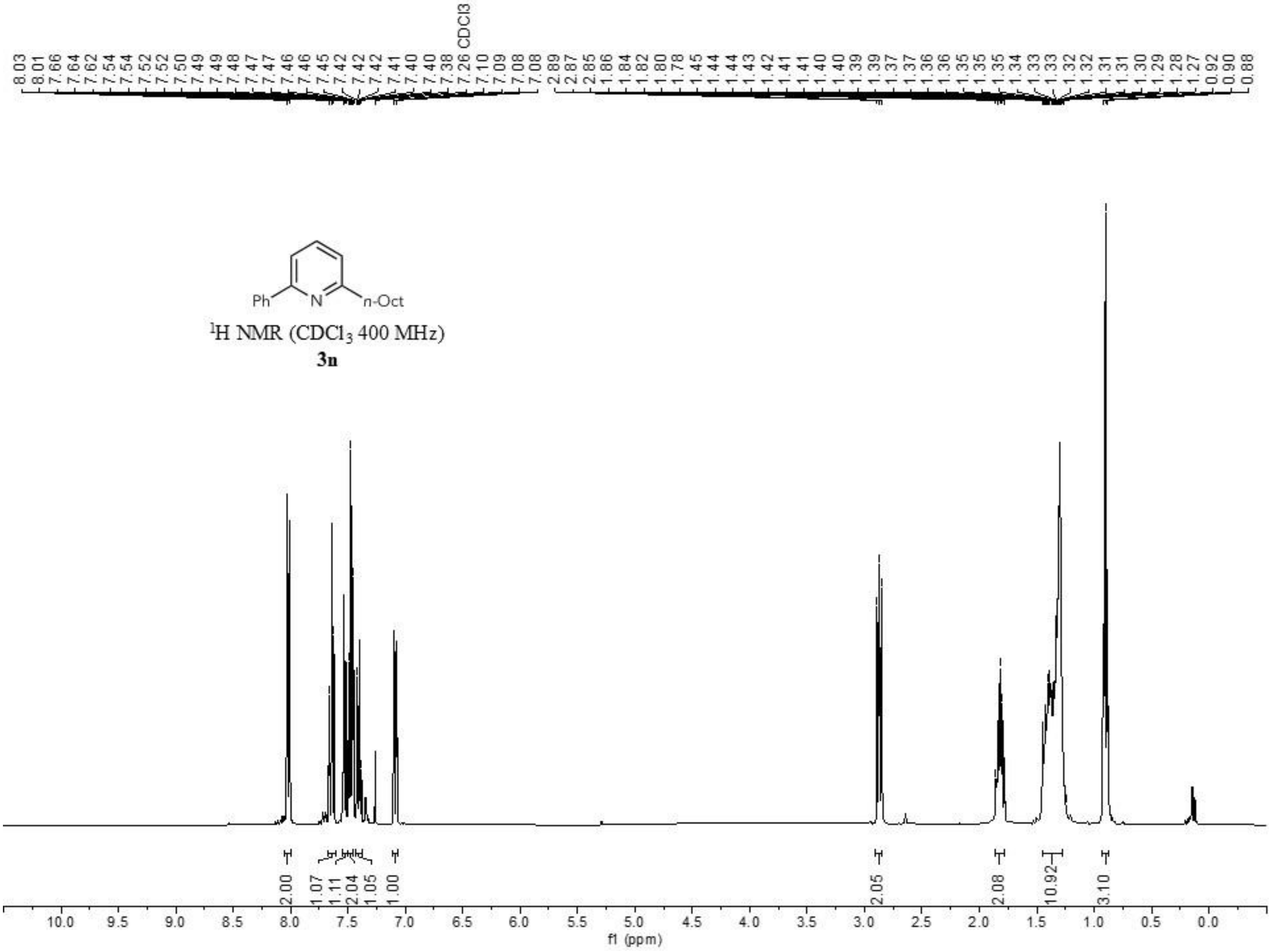
8.03
8.01
7.66
7.64
7.62
7.54
7.54
7.52
7.52
7.50
7.49
7.49
7.48
7.47
7.47
7.46
7.46
7.45
7.42
7.42
7.42
7.41
7.40
7.40
7.38
7.26 CDCl3
7.10
7.09
7.08
7.08
2.89
2.87
2.85
1.86
1.84
1.82
1.80
1.78
1.45
1.44
1.44
1.43
1.42
1.41
1.41
1.40
1.40
1.39
1.39
1.37
1.37
1.36
1.36
1.35
1.35
1.35
1.34
1.33
1.33
1.32
1.32
1.31
1.31
1.30
1.29
1.28
1.27
0.92
0.90
0.88
Ph
N
n-Oct
^{1}H NMR ($CDCl_3$ 400 MHz)
3n
2.00
1.07
1.11
2.04
1.05
1.00
2.05
2.08
10.92
3.10
10.0
9.5
9.0
8.5
8.0
7.5
7.0
6.5
6.0
5.5
5.0
4.5
4.0
3.5
3.0
2.5
2.0
1.5
1.0
0.5
0.0
f1 (ppm)

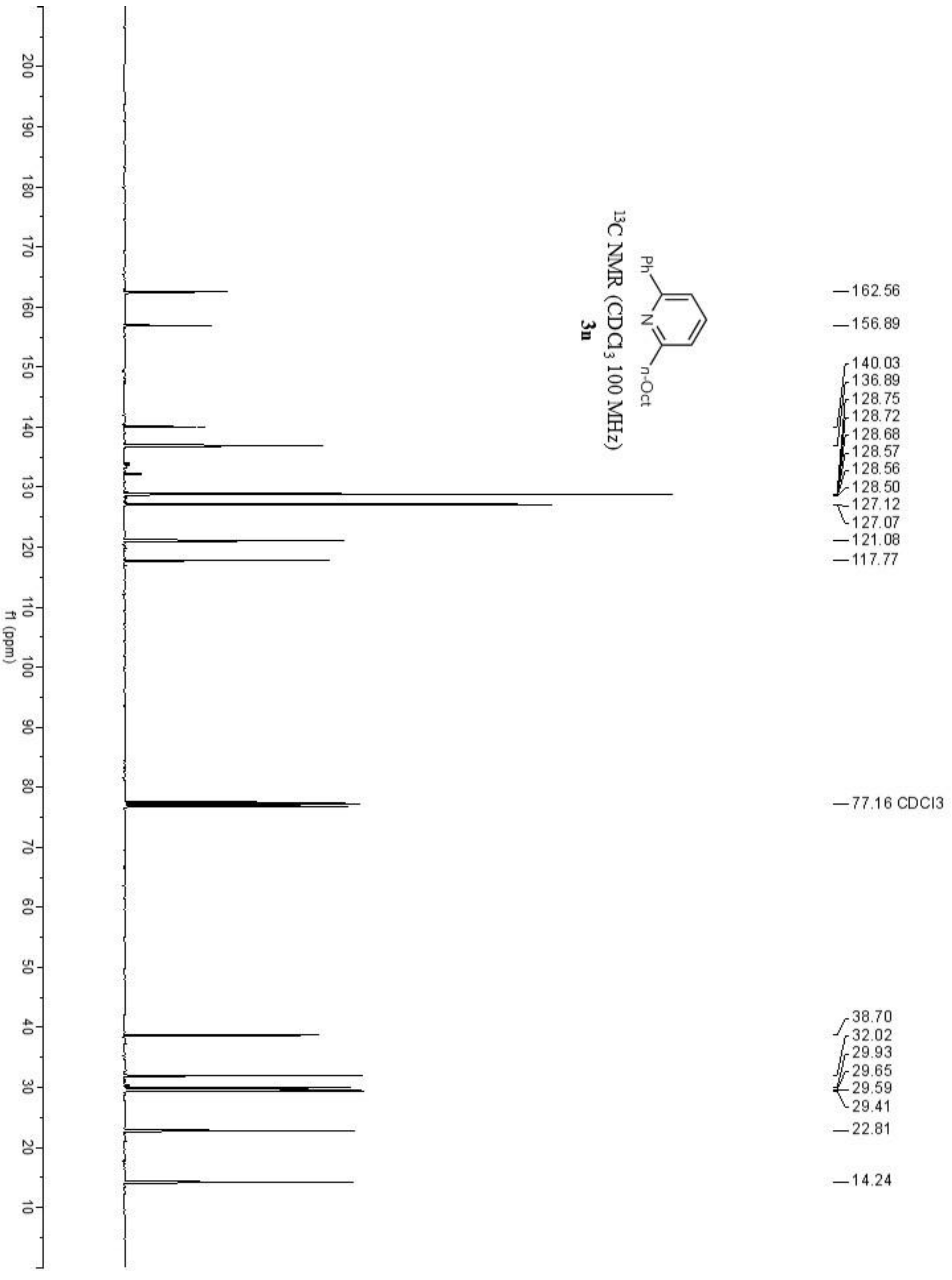
Ph
N
n-Oct
13C NMR (CDCl3 100 MHz)
3n
162.56
156.89
140.03
136.89
128.75
128.72
128.68
128.57
128.56
128.50
127.12
127.07
121.08
117.77
77.16 CDCl3
38.70
32.02
29.93
29.65
29.59
29.41
22.81
14.24
200
190
180
170
160
150
140
130
120
110
100
90
80
70
60
50
40
30
20
10
f1 (ppm)

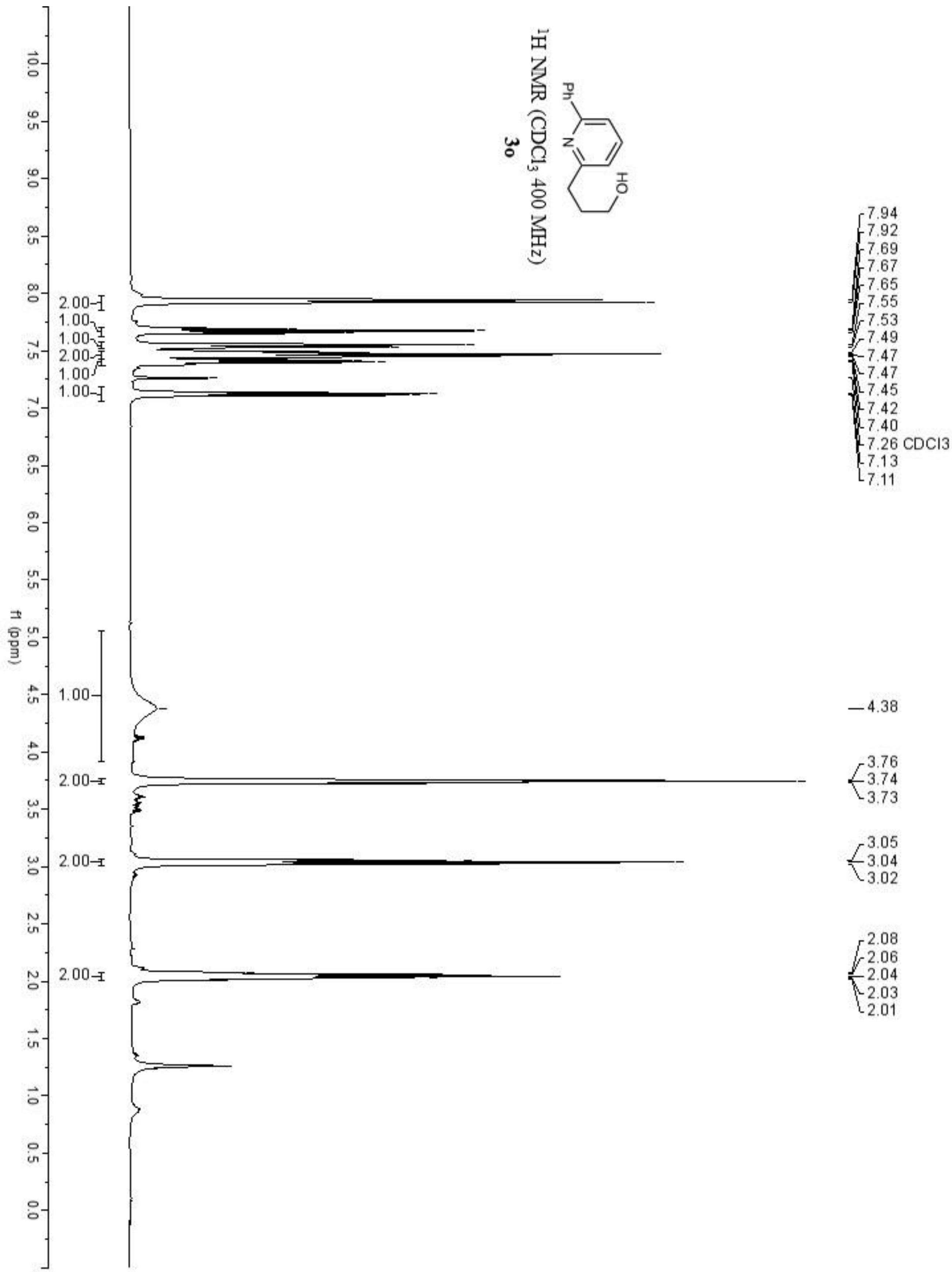

^{1}H NMR (CDCl$_3$ 400 MHz)
3o
HO
N
Ph
f1 (ppm)
7.94
7.92
7.69
7.67
7.65
7.55
7.53
7.49
7.47
7.47
7.45
7.42
7.40
7.26 CDCl3
7.13
7.11
4.38
3.76
3.74
3.73
3.05
3.04
3.02
2.08
2.06
2.04
2.03
2.01
2.00
1.00
1.00
2.00
1.00
1.00
1.00
2.00
2.00
2.00

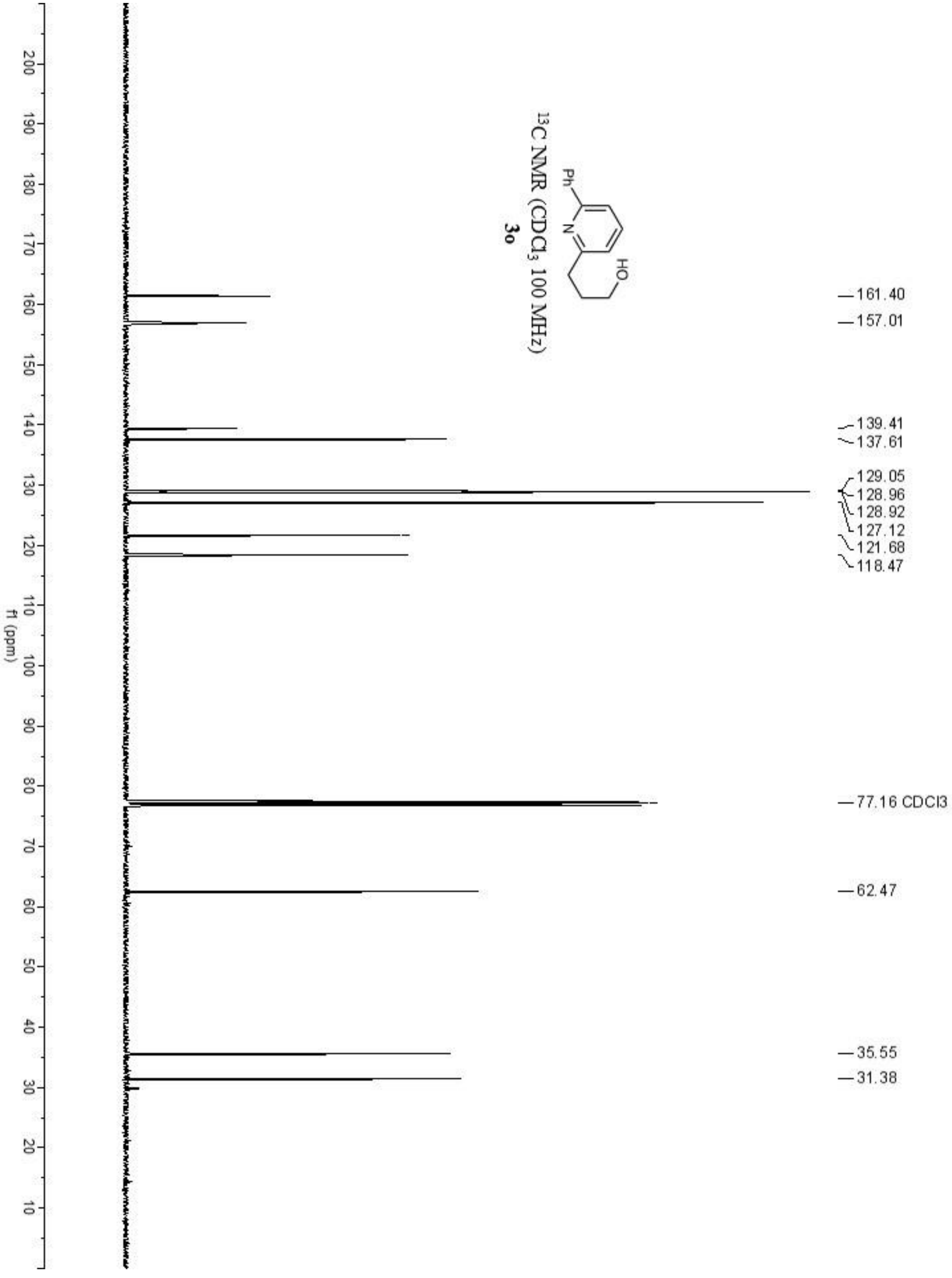

^{13}C NMR (CDCl$_3$ 100 MHz)
3o
Ph
N
HO
161.40
157.01
139.41
137.61
129.05
128.96
128.92
127.12
121.68
118.47
77.16 CDCl3
62.47
35.55
31.38
f1 (ppm)

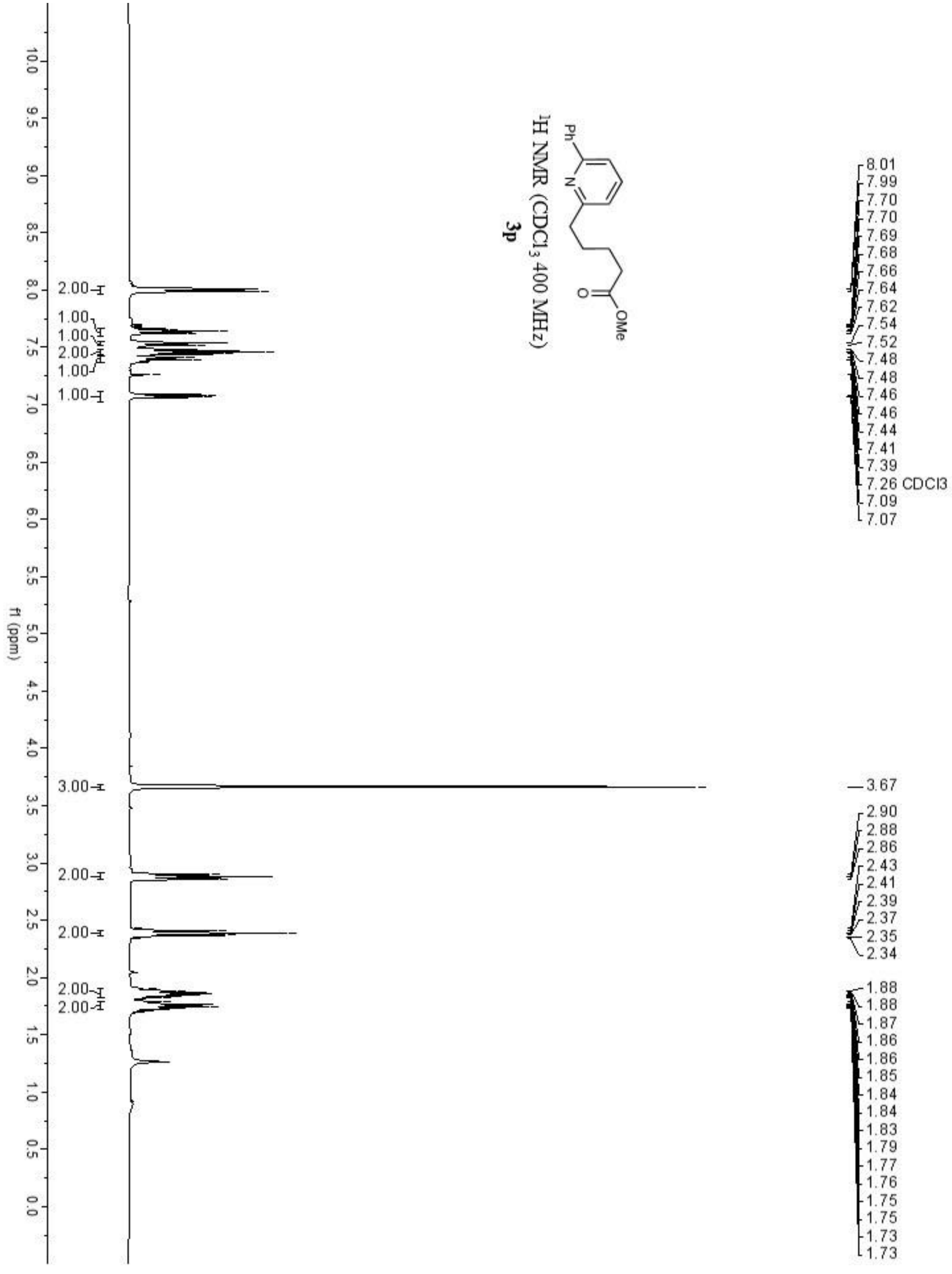

Ph
N
O
OMe
1H NMR (CDCl3 400 MHz)
3p
8.01
7.99
7.70
7.70
7.69
7.68
7.66
7.64
7.62
7.54
7.52
7.48
7.48
7.46
7.46
7.44
7.41
7.39
7.26 CDCl3
7.09
7.07
3.67
2.90
2.88
2.86
2.43
2.41
2.39
2.37
2.35
2.34
1.88
1.88
1.87
1.86
1.86
1.85
1.84
1.84
1.83
1.79
1.77
1.76
1.75
1.75
1.73
1.73
f1 (ppm)

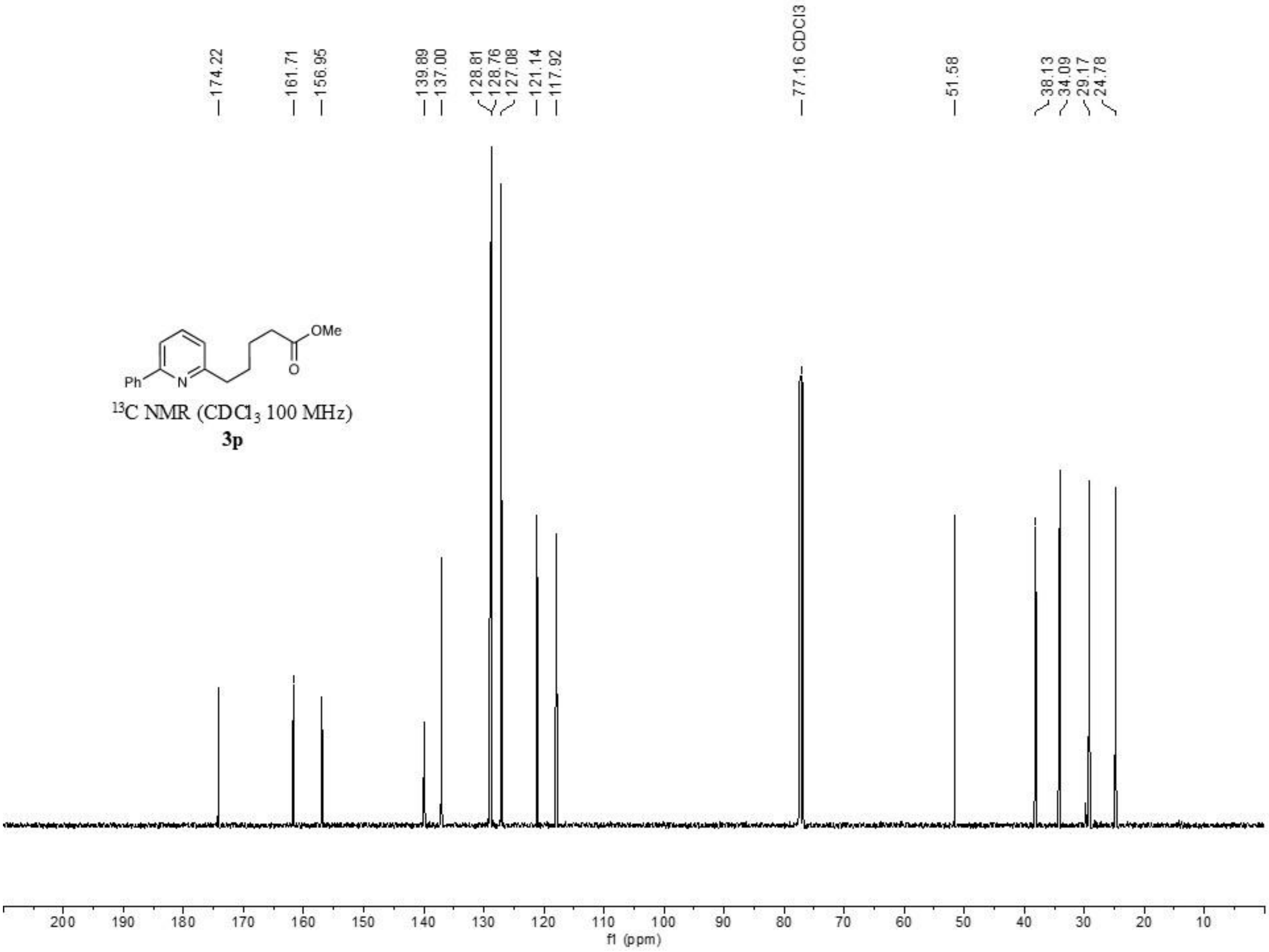

174.22
161.71
156.95
139.89
137.00
128.81
128.76
127.08
121.14
117.92
77.16 CDCl3
51.58
38.13
34.09
29.17
24.78
Ph
N
OMe
O
13C NMR (CDCl3 100 MHz)
3p
200
190
180
170
160
150
140
130
120
110
100
90
80
70
60
50
40
30
20
10
f1 (ppm)

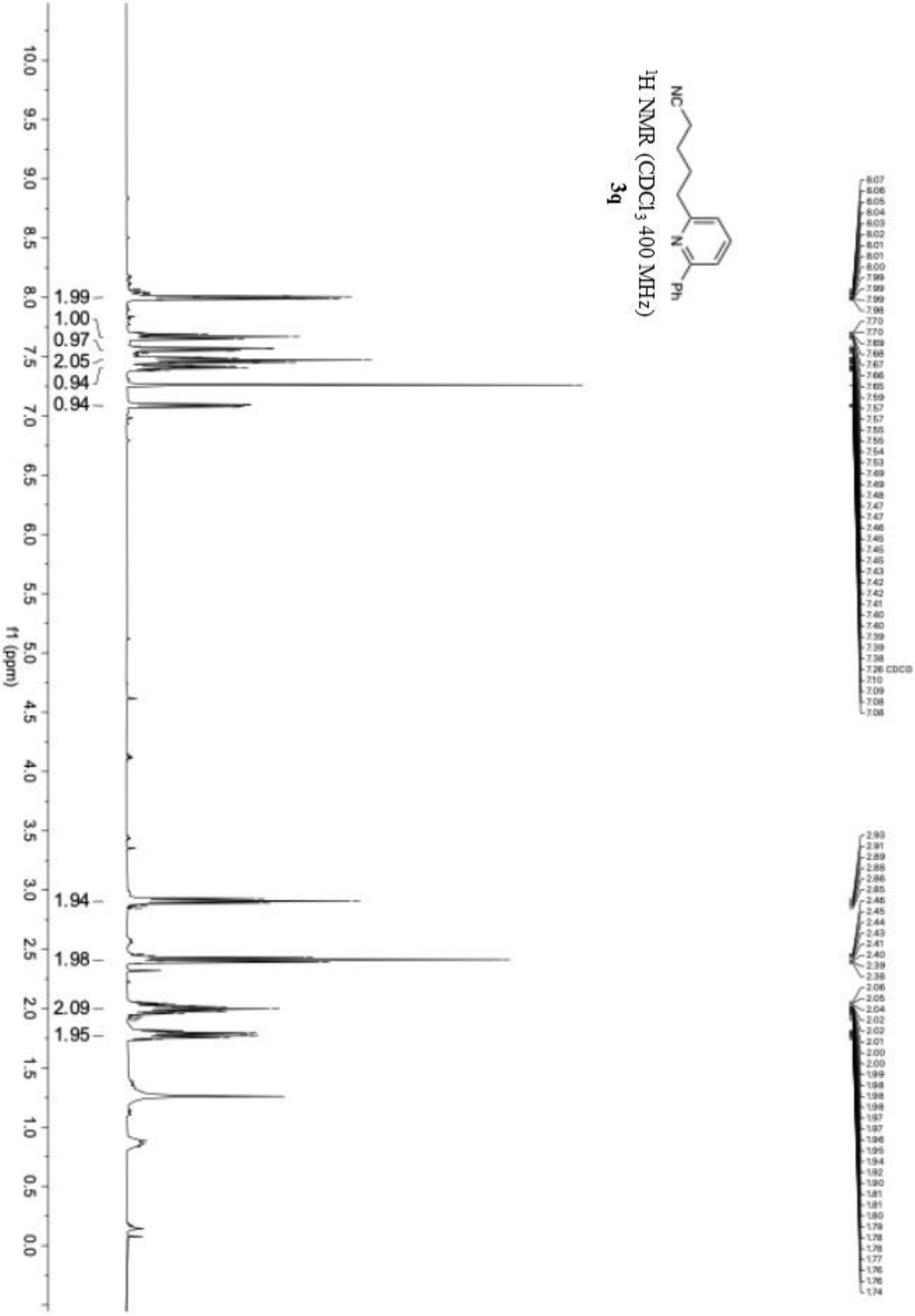

¹H NMR (CDCl₃ 400 MHz)
3q
NC
N
Ph
1.99
1.00
0.97
2.05
0.94
0.94
1.94
1.98
2.09
1.95
f1 (ppm)
7.26 CDCl3

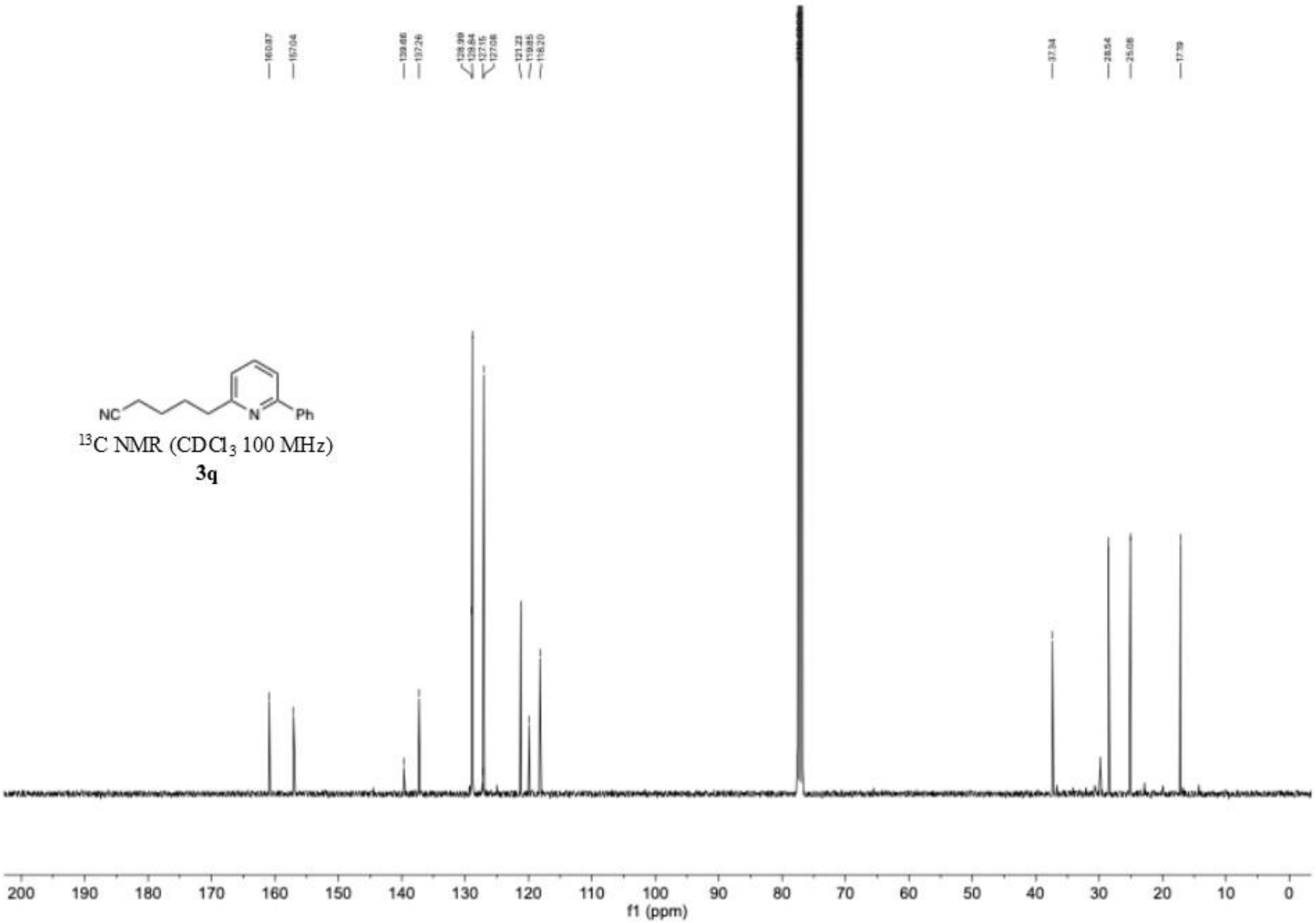

^{13}C NMR ($CDCl_3$ 100 MHz)

3q

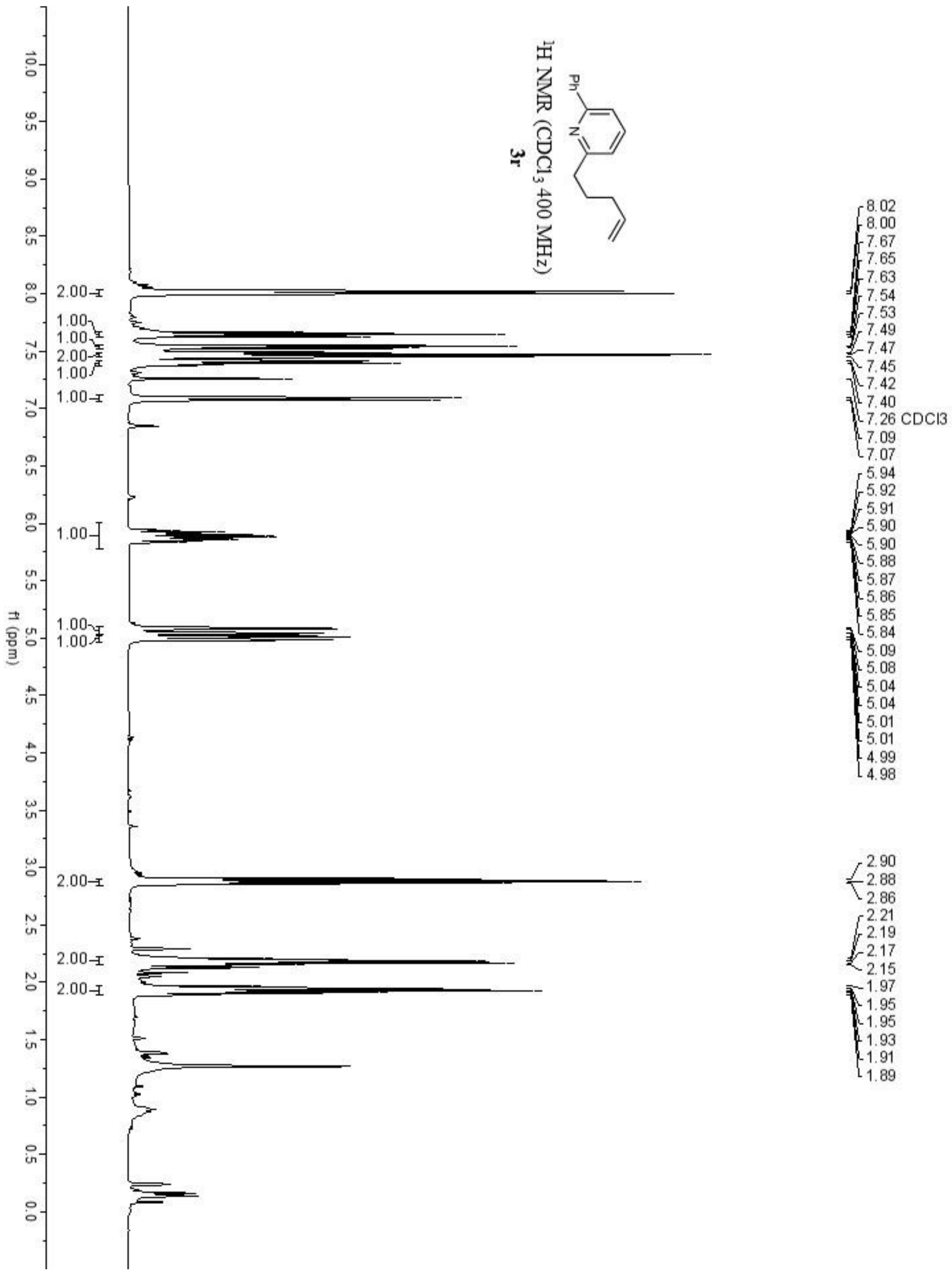

1H NMR (CDCl3 400 MHz)
3r
Ph
N
8.02
8.00
7.67
7.65
7.63
7.54
7.53
7.49
7.47
7.45
7.42
7.40
7.26 CDCl3
7.09
7.07
5.94
5.92
5.91
5.90
5.90
5.88
5.87
5.86
5.85
5.84
5.09
5.08
5.04
5.04
5.01
5.01
4.99
4.98
2.90
2.88
2.86
2.21
2.19
2.17
2.15
1.97
1.95
1.95
1.93
1.91
1.89
2.00
1.00
1.00
2.00
1.00
1.00
1.00
1.00
1.00
2.00
2.00
2.00
f1 (ppm)
10.0
9.5
9.0
8.5
8.0
7.5
7.0
6.5
6.0
5.5
5.0
4.5
4.0
3.5
3.0
2.5
2.0
1.5
1.0
0.5
0.0

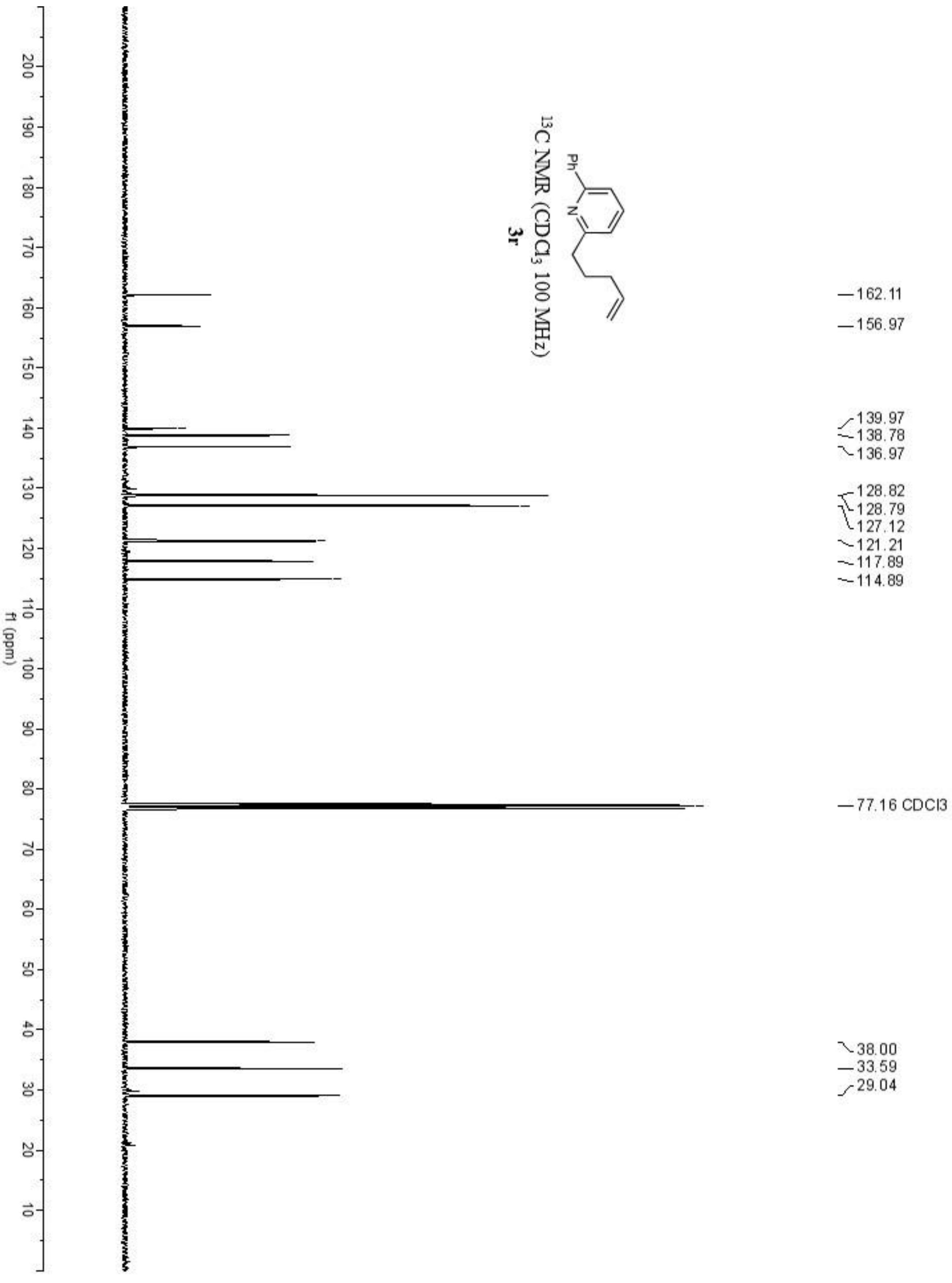

^{13}C NMR ($CDCl_3$ 100 MHz)
3r
Ph
N
162.11
156.97
139.97
138.78
136.97
128.82
128.79
127.12
121.21
117.89
114.89
77.16 CDCl3
38.00
33.59
29.04
f1 (ppm)
200
190
180
170
160
150
140
130
120
110
100
90
80
70
60
50
40
30
20
10

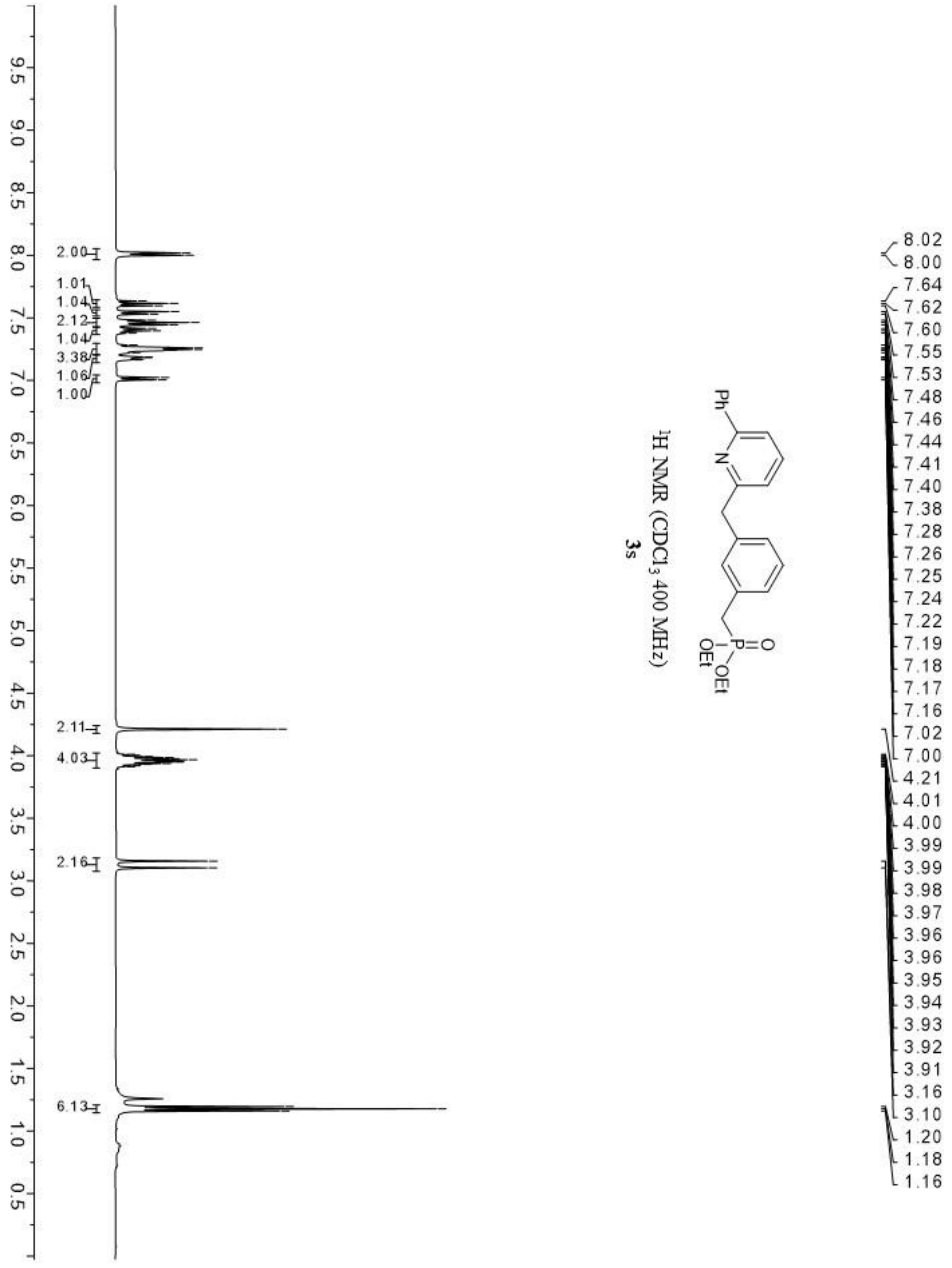

^{1}H NMR ($CDCl_3$ 400 MHz)

3s

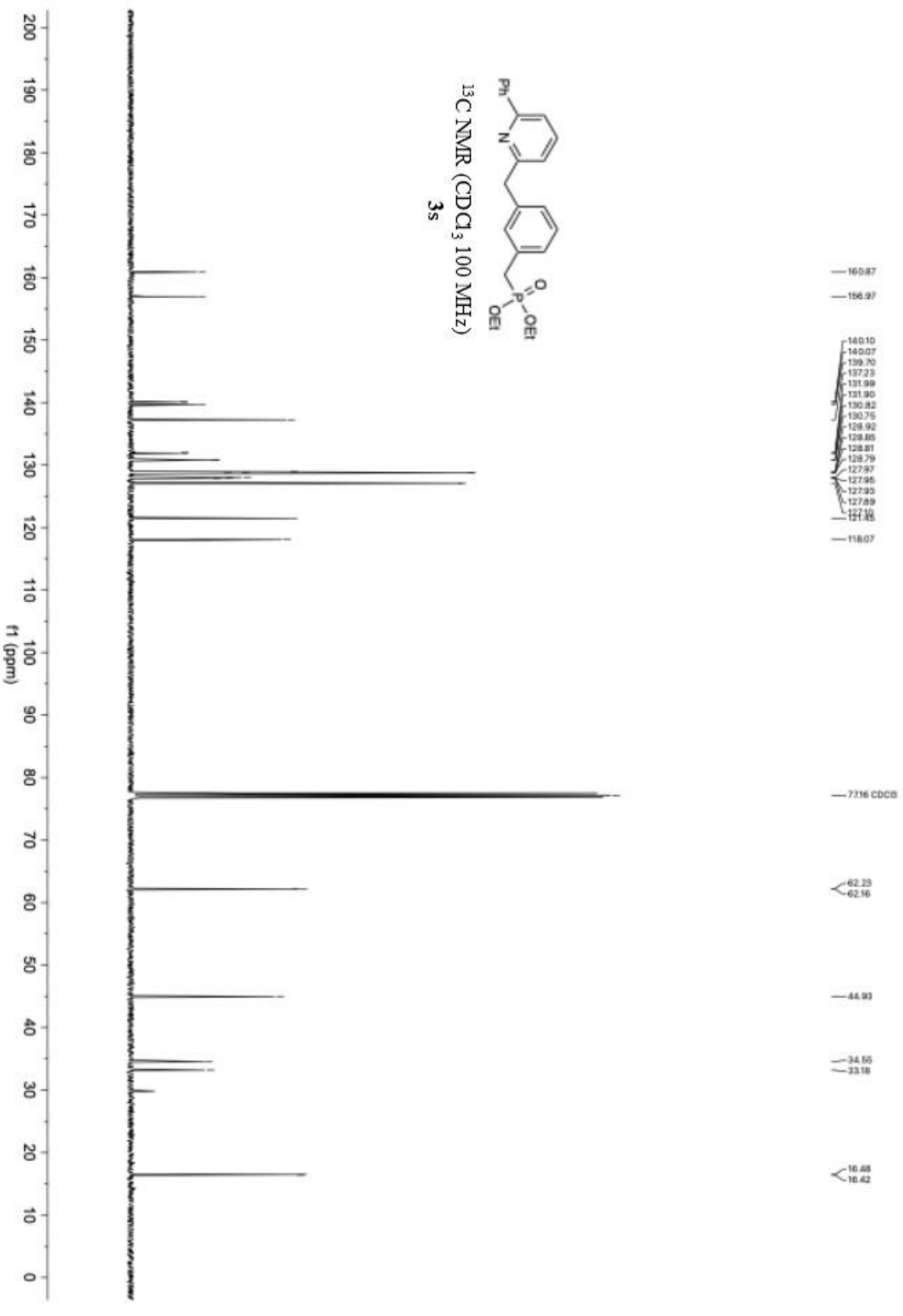
13C NMR (CDCl3 100 MHz)
3s
f1 (ppm)
160.87
156.97
77.16 CDCl3
62.23
62.16
44.93
34.55
33.18
16.48
16.42

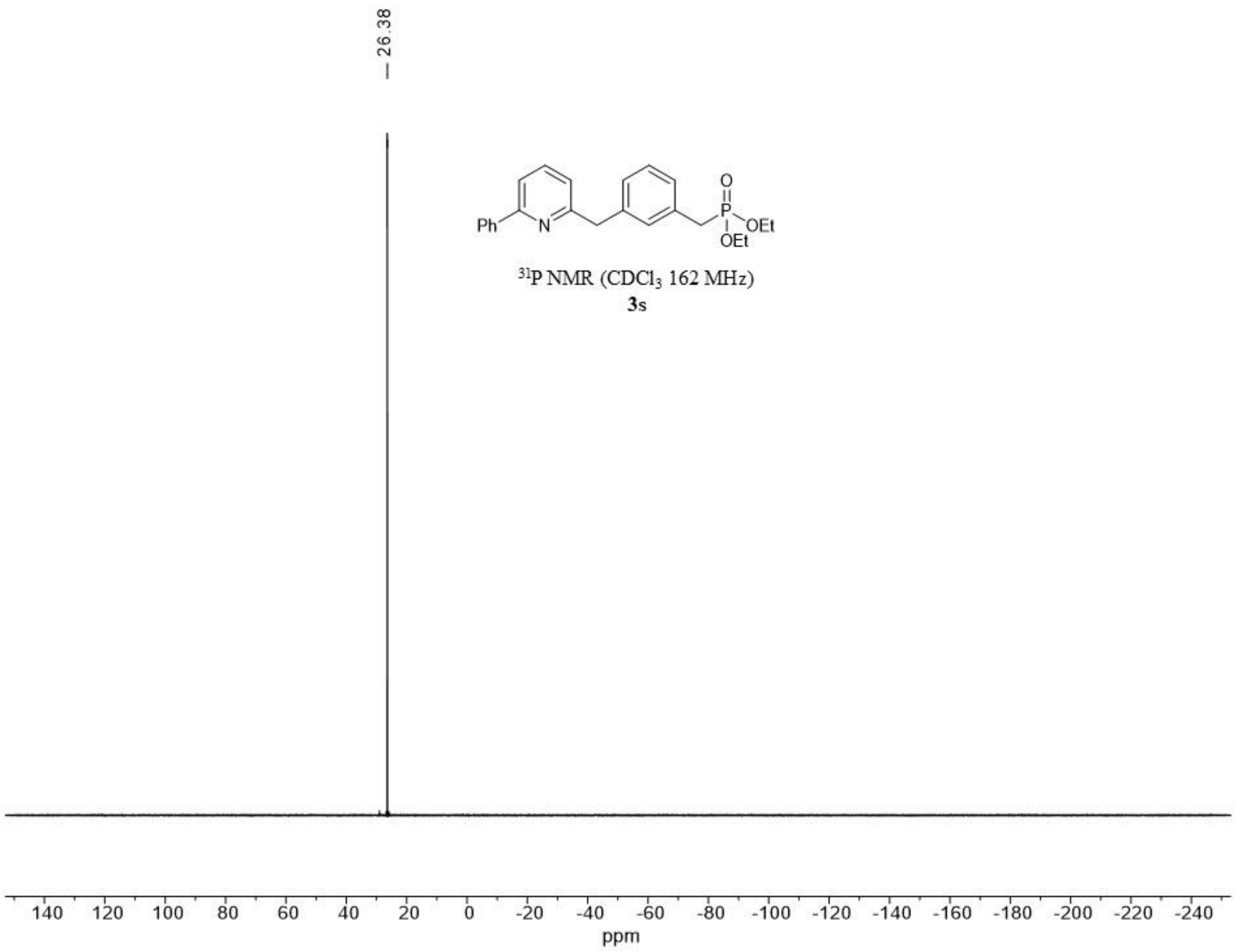
26.38
Ph
N
O
P
OEt
OEt
^{31}P NMR ($CDCl_3$ 162 MHz)
3s
140 120 100 80 60 40 20 0 -20 -40 -60 -80 -100 -120 -140 -160 -180 -200 -220 -240
ppm

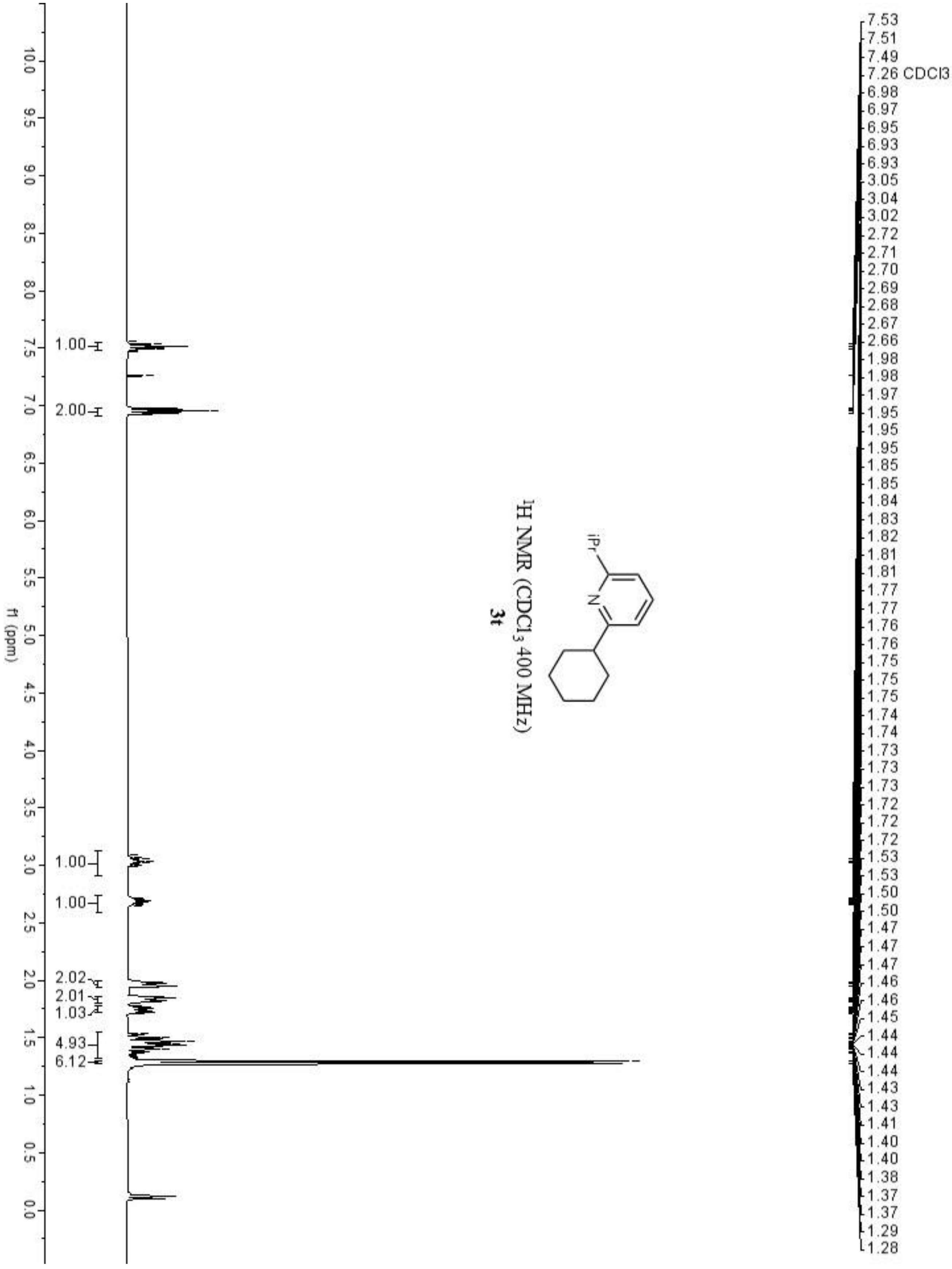

7.53
7.51
7.49
7.26 CDCl3
6.98
6.97
6.95
6.93
6.93
3.05
3.04
3.02
2.72
2.71
2.70
2.69
2.68
2.67
2.66
1.98
1.98
1.97
1.95
1.95
1.95
1.85
1.85
1.84
1.83
1.82
1.81
1.81
1.77
1.77
1.76
1.76
1.75
1.75
1.75
1.74
1.74
1.73
1.73
1.73
1.72
1.72
1.72
1.53
1.53
1.50
1.50
1.47
1.47
1.47
1.46
1.46
1.45
1.44
1.44
1.44
1.43
1.43
1.41
1.40
1.40
1.38
1.37
1.37
1.29
1.28
iPr
N
1H NMR (CDCl3 400 MHz)
3t
1.00
2.00
1.00
1.00
2.02
2.01
1.03
4.93
6.12
10.0
9.5
9.0
8.5
8.0
7.5
7.0
6.5
6.0
5.5
5.0
4.5
4.0
3.5
3.0
2.5
2.0
1.5
1.0
0.5
0.0
f1 (ppm)

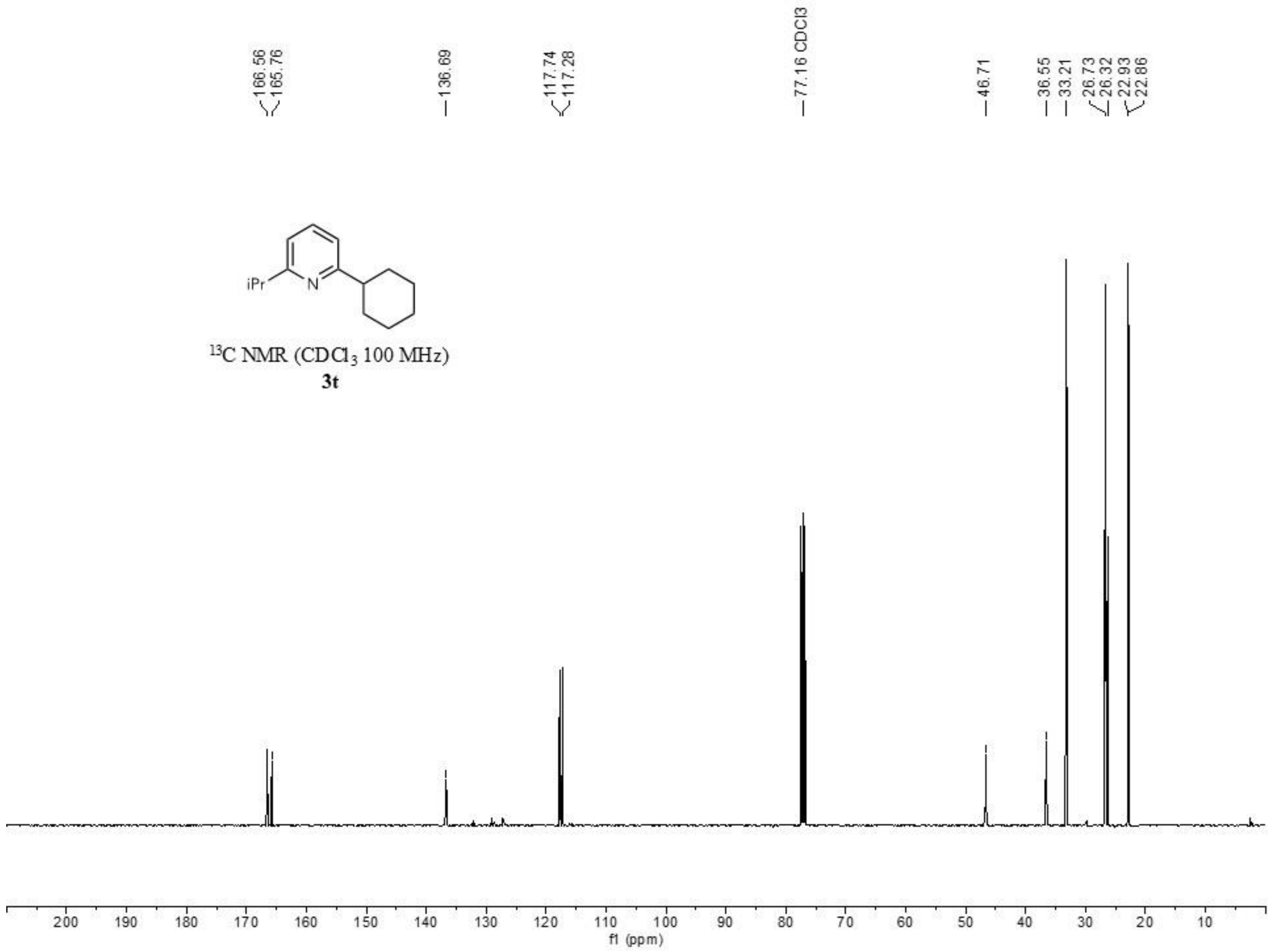
166.56
165.76
136.69
117.74
117.28
77.16 CDCl3
46.71
36.55
33.21
26.73
26.32
22.93
22.86
iPr
N
^{13}C NMR (CDCl$_3$ 100 MHz)
3t
200
190
180
170
160
150
140
130
120
110
100
90
80
70
60
50
40
30
20
10
f1 (ppm)

^{1}H NMR ($CDCl_3$ 400 MHz)

3u

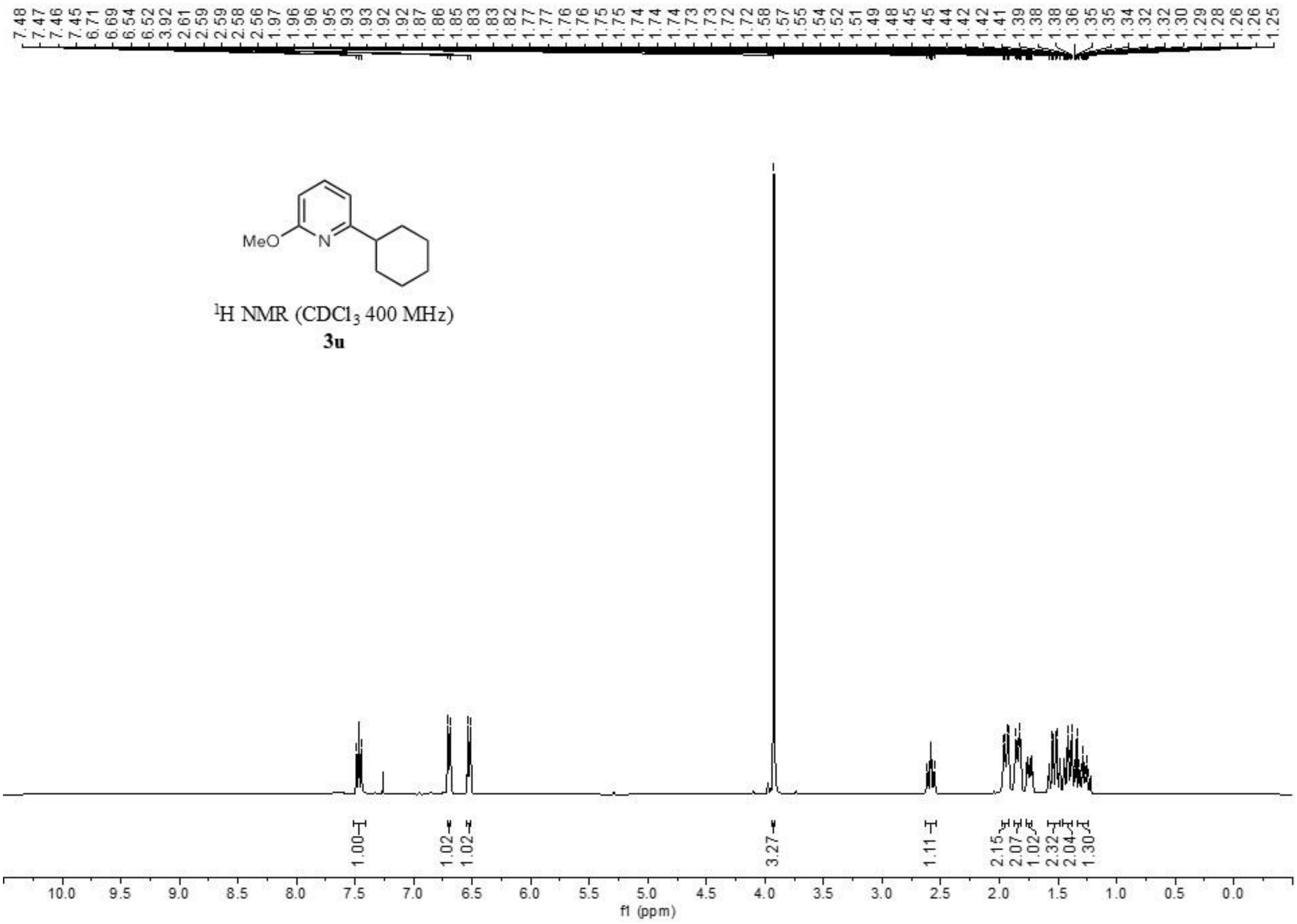

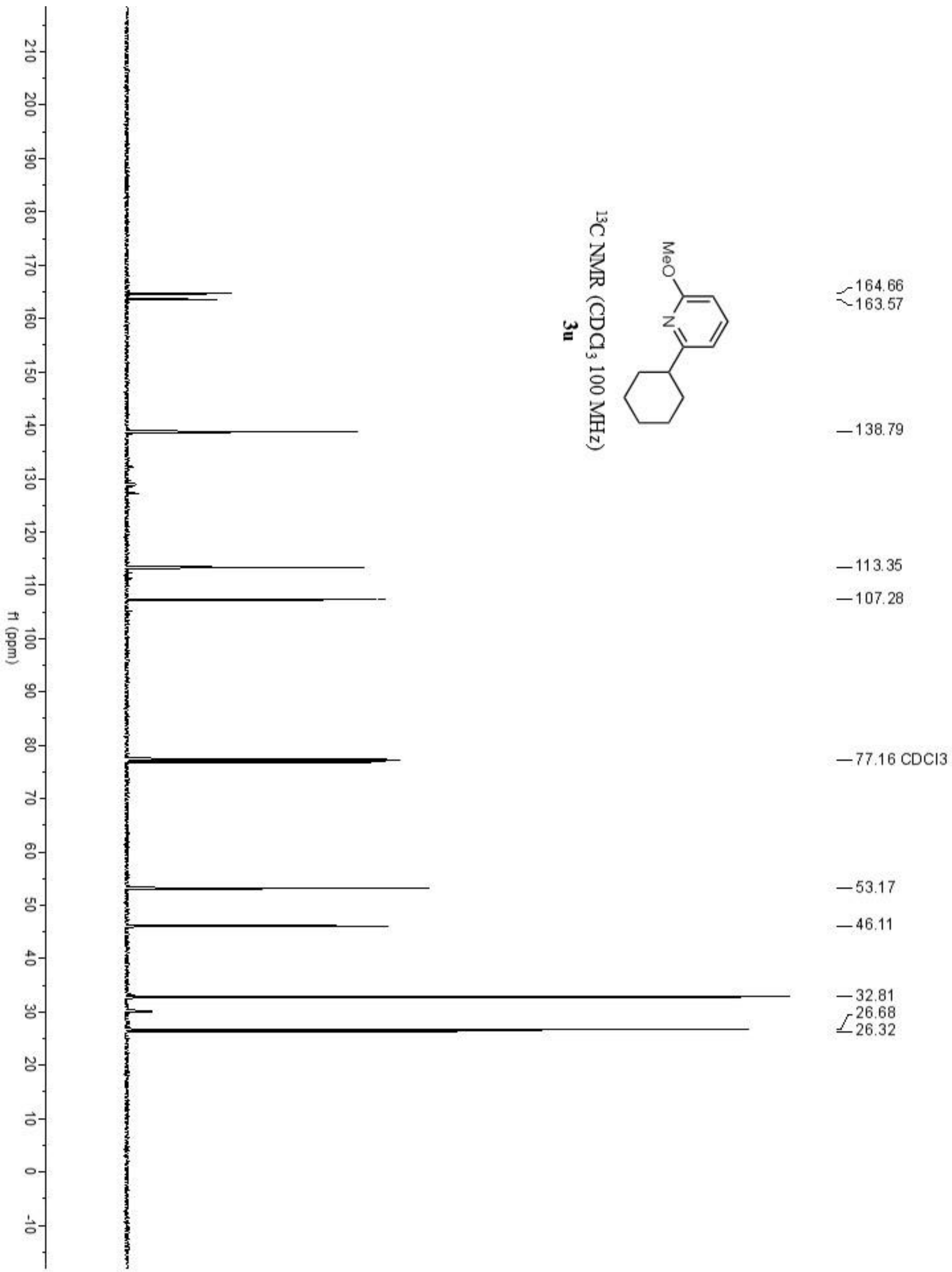
MeO
N
13C NMR (CDCl3 100 MHz)
3u
164.66
163.57
138.79
113.35
107.28
77.16 CDCl3
53.17
46.11
32.81
26.68
26.32
210
200
190
180
170
160
150
140
130
120
110
100
90
80
70
60
50
40
30
20
10
0
-10
f1 (ppm)

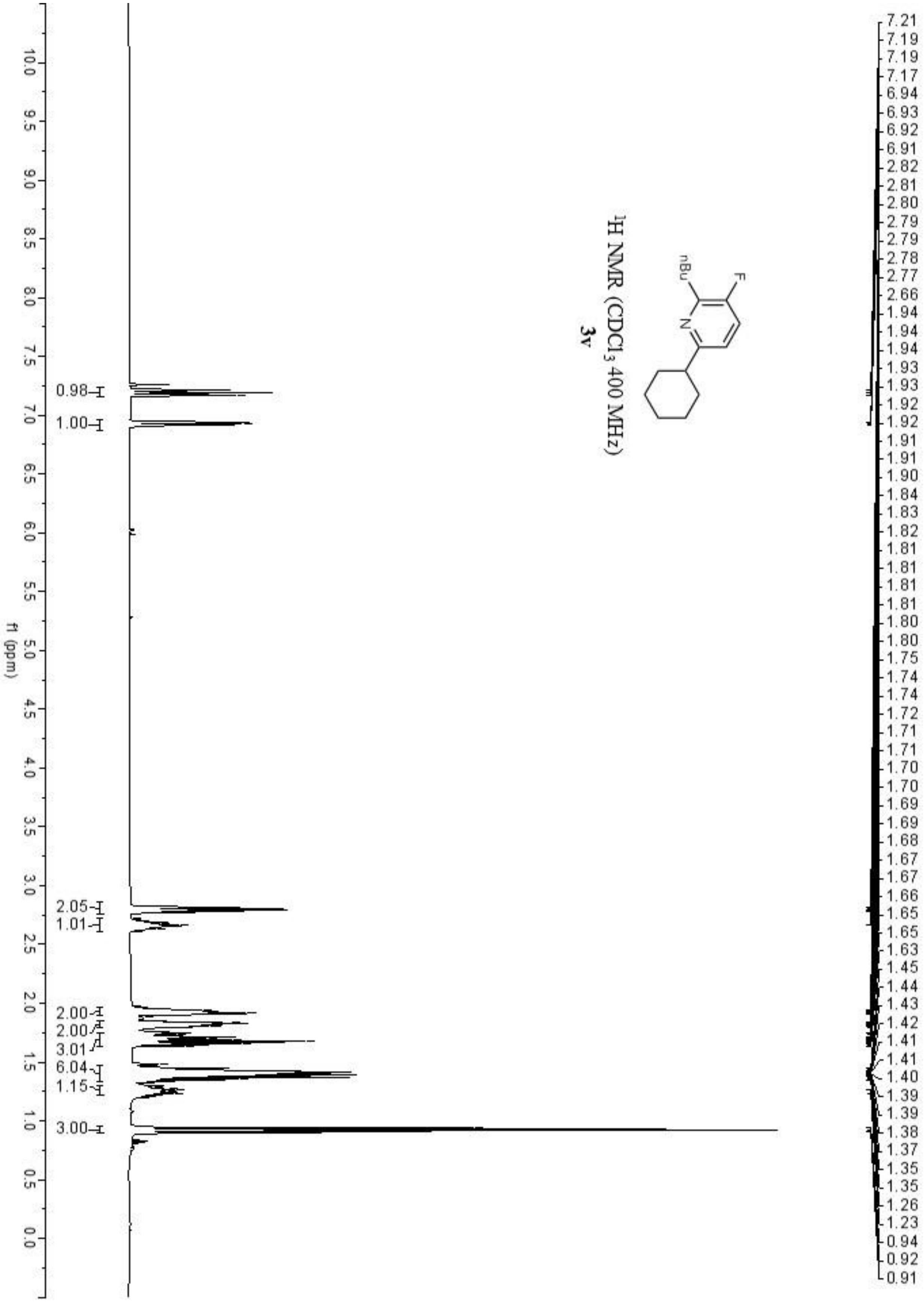

¹H NMR (CDCl₃ 400 MHz)
3v
nBu
N
F
7.21
7.19
7.19
7.17
6.94
6.93
6.92
6.91
2.82
2.81
2.80
2.79
2.79
2.78
2.77
2.66
1.94
1.94
1.94
1.93
1.93
1.92
1.92
1.91
1.91
1.90
1.84
1.83
1.82
1.81
1.81
1.81
1.81
1.80
1.80
1.75
1.74
1.74
1.72
1.71
1.71
1.70
1.70
1.69
1.69
1.68
1.67
1.67
1.66
1.65
1.65
1.63
1.45
1.44
1.43
1.42
1.41
1.41
1.40
1.39
1.39
1.38
1.37
1.35
1.35
1.26
1.23
0.94
0.92
0.91
0.98
1.00
2.05
1.01
2.00
2.00
3.01
6.04
1.15
3.00
10.0
9.5
9.0
8.5
8.0
7.5
7.0
6.5
6.0
5.5
5.0
4.5
4.0
3.5
3.0
2.5
2.0
1.5
1.0
0.5
0.0
f1 (ppm)

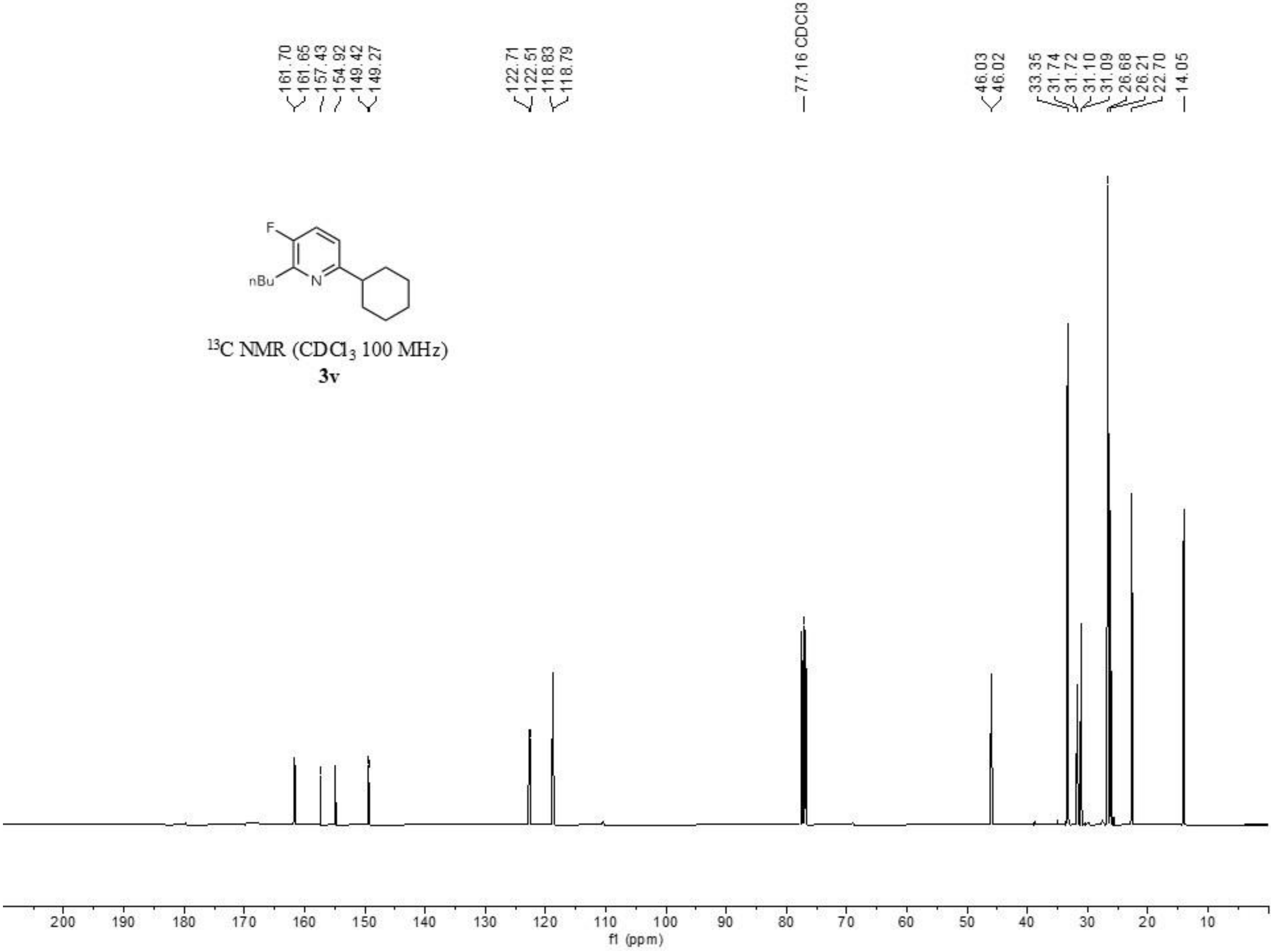
161.70
161.65
157.43
154.92
149.42
149.27
122.71
122.51
118.83
118.79
77.16 CDCl3
46.03
46.02
33.35
31.74
31.72
31.10
31.09
26.68
26.21
22.70
14.05
F
nBu
N
^{13}C NMR ($CDCl_3$ 100 MHz)
3v
200 190 180 170 160 150 140 130 120 110 100 90 80 70 60 50 40 30 20 10
f1 (ppm)

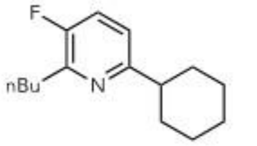

^{19}F NMR ($CDCl_3$ 376 MHz)
3v

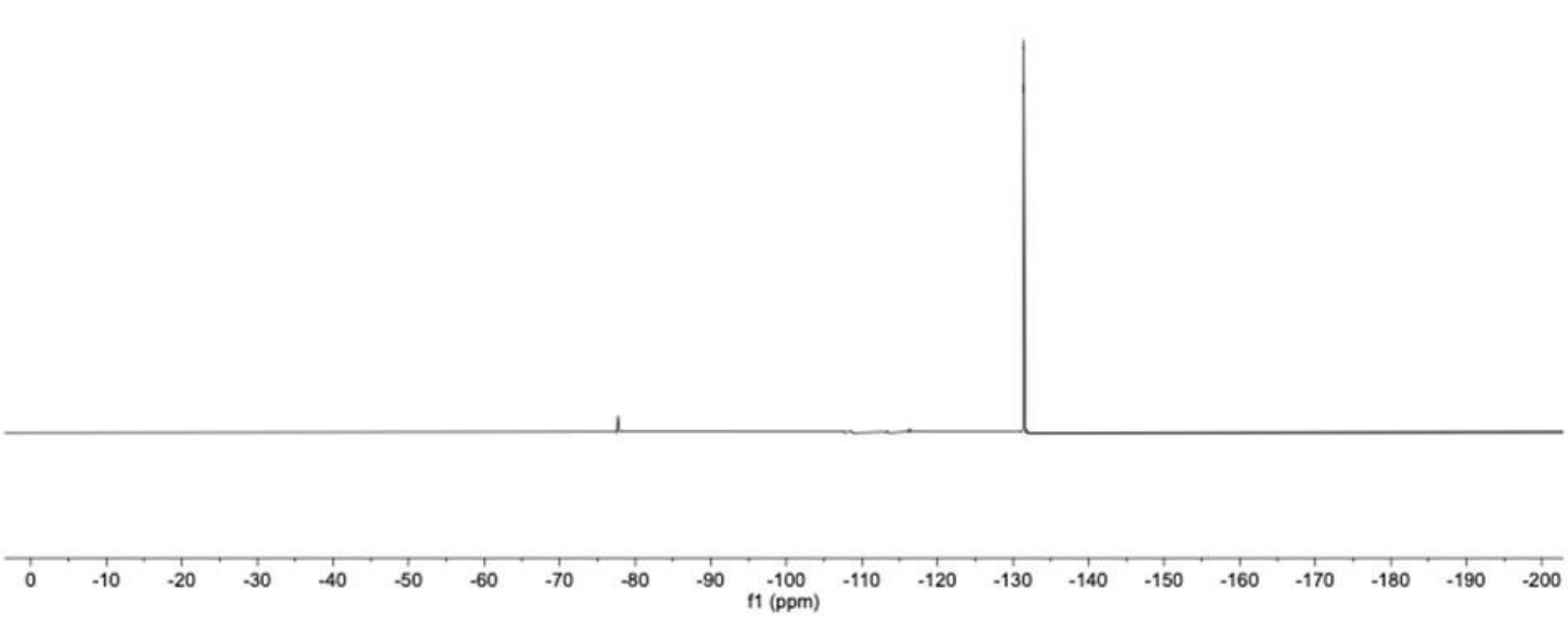

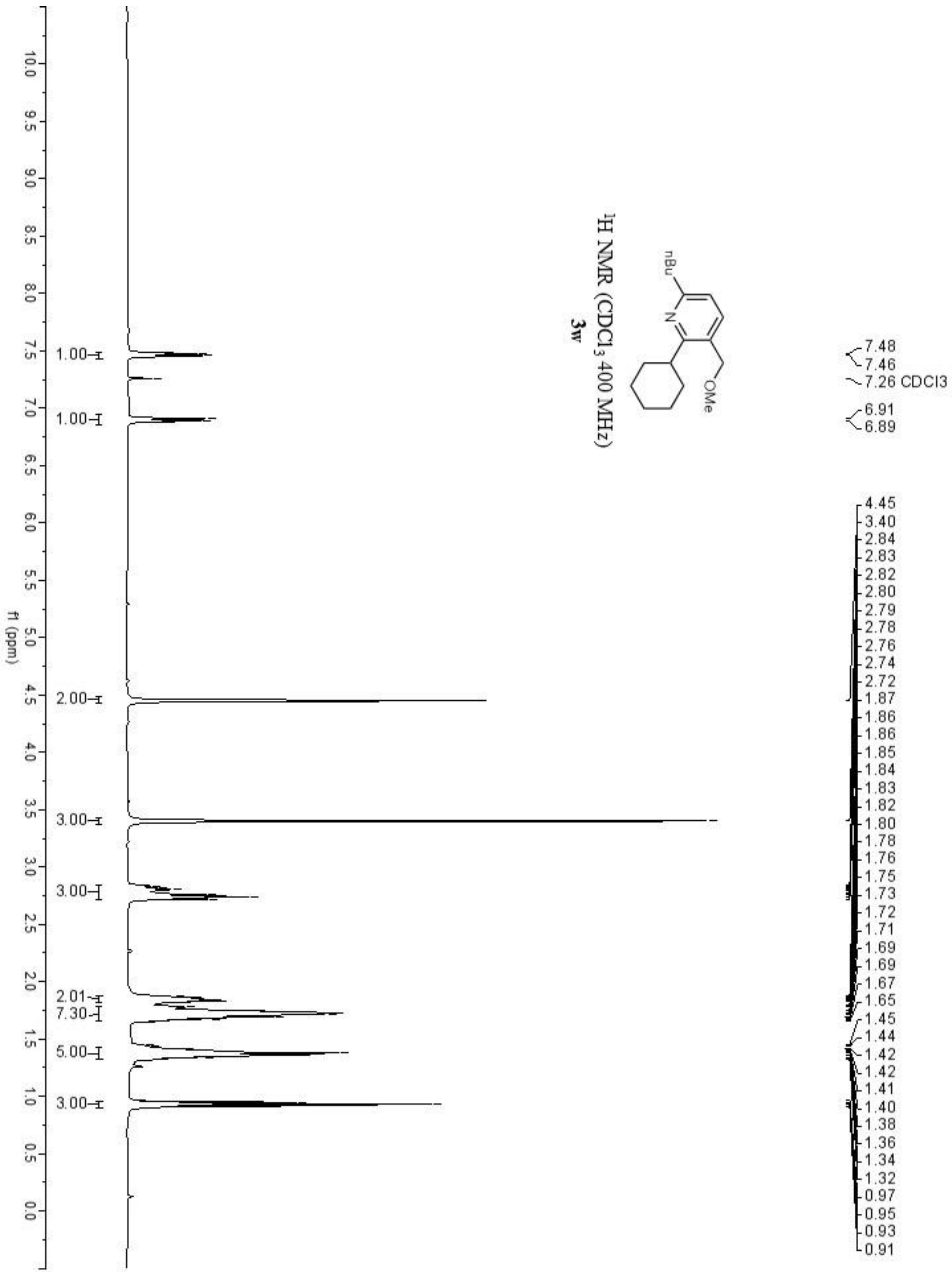

^{1}H NMR ($CDCl_3$ 400 MHz)
3w
nBu
N
OMe
7.48
7.46
7.26 CDCl3
6.91
6.89
4.45
3.40
2.84
2.83
2.82
2.80
2.79
2.78
2.76
2.74
2.72
1.87
1.86
1.86
1.85
1.84
1.83
1.82
1.80
1.78
1.76
1.75
1.73
1.72
1.71
1.69
1.69
1.67
1.65
1.45
1.44
1.42
1.42
1.41
1.40
1.38
1.36
1.34
1.32
0.97
0.95
0.93
0.91
1.00
1.00
2.00
3.00
3.00
2.01
7.30
5.00
3.00
10.0
9.5
9.0
8.5
8.0
7.5
7.0
6.5
6.0
5.5
5.0
4.5
4.0
3.5
3.0
2.5
2.0
1.5
1.0
0.5
0.0
f1 (ppm)

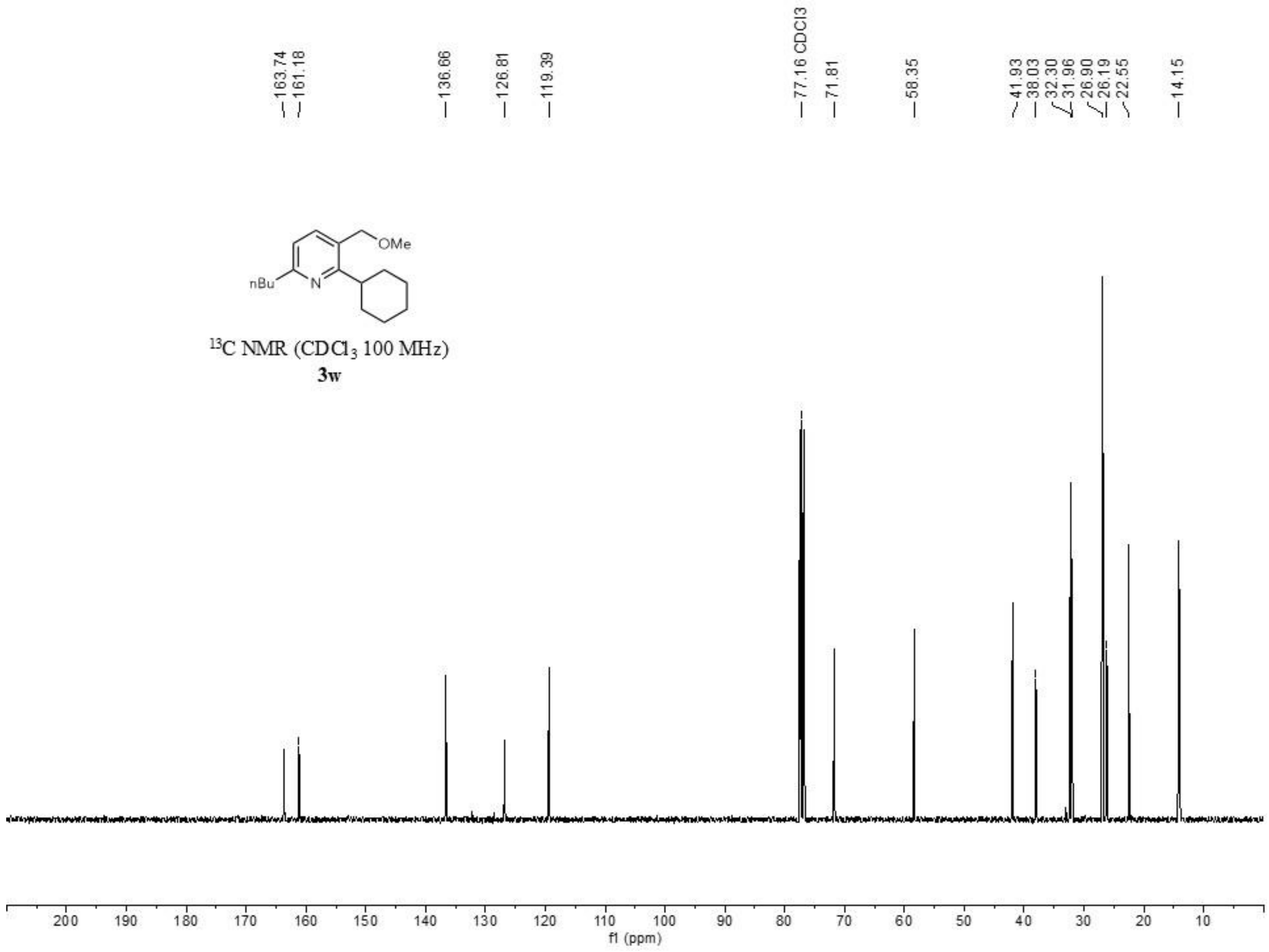
163.74
161.18
136.66
126.81
119.39
77.16 CDCl3
71.81
58.35
41.93
38.03
32.30
31.96
26.90
26.19
22.55
14.15
OMe
nBu
N
^{13}C NMR ($CDCl_3$ 100 MHz)
3w
200
190
180
170
160
150
140
130
120
110
100
90
80
70
60
50
40
30
20
10
f1 (ppm)

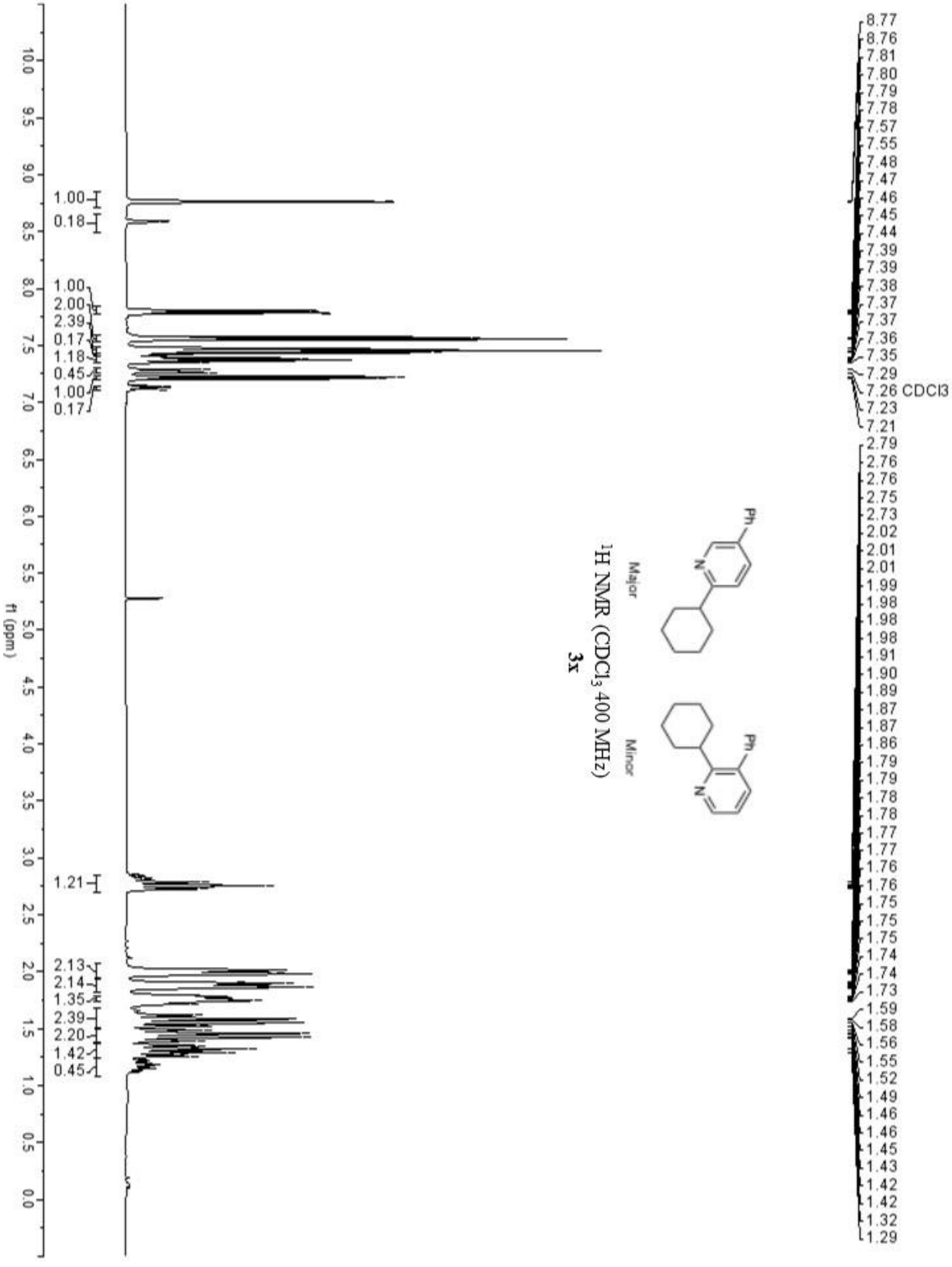
1H NMR (CDCl3 400 MHz)
3x
Major
Minor
Ph
N
8.77 8.76 7.81 7.80 7.79 7.78 7.57 7.55 7.48 7.47 7.46 7.45 7.44 7.39 7.39 7.38 7.37 7.37 7.36 7.35 7.29 7.26 CDCl3 7.23 7.21 2.79 2.76 2.76 2.75 2.73 2.02 2.01 2.01 1.99 1.98 1.98 1.98 1.91 1.90 1.89 1.87 1.87 1.86 1.79 1.79 1.78 1.78 1.77 1.77 1.76 1.76 1.75 1.75 1.75 1.74 1.74 1.73 1.59 1.58 1.56 1.55 1.52 1.49 1.46 1.46 1.45 1.43 1.42 1.42 1.32 1.29
f1 (ppm)

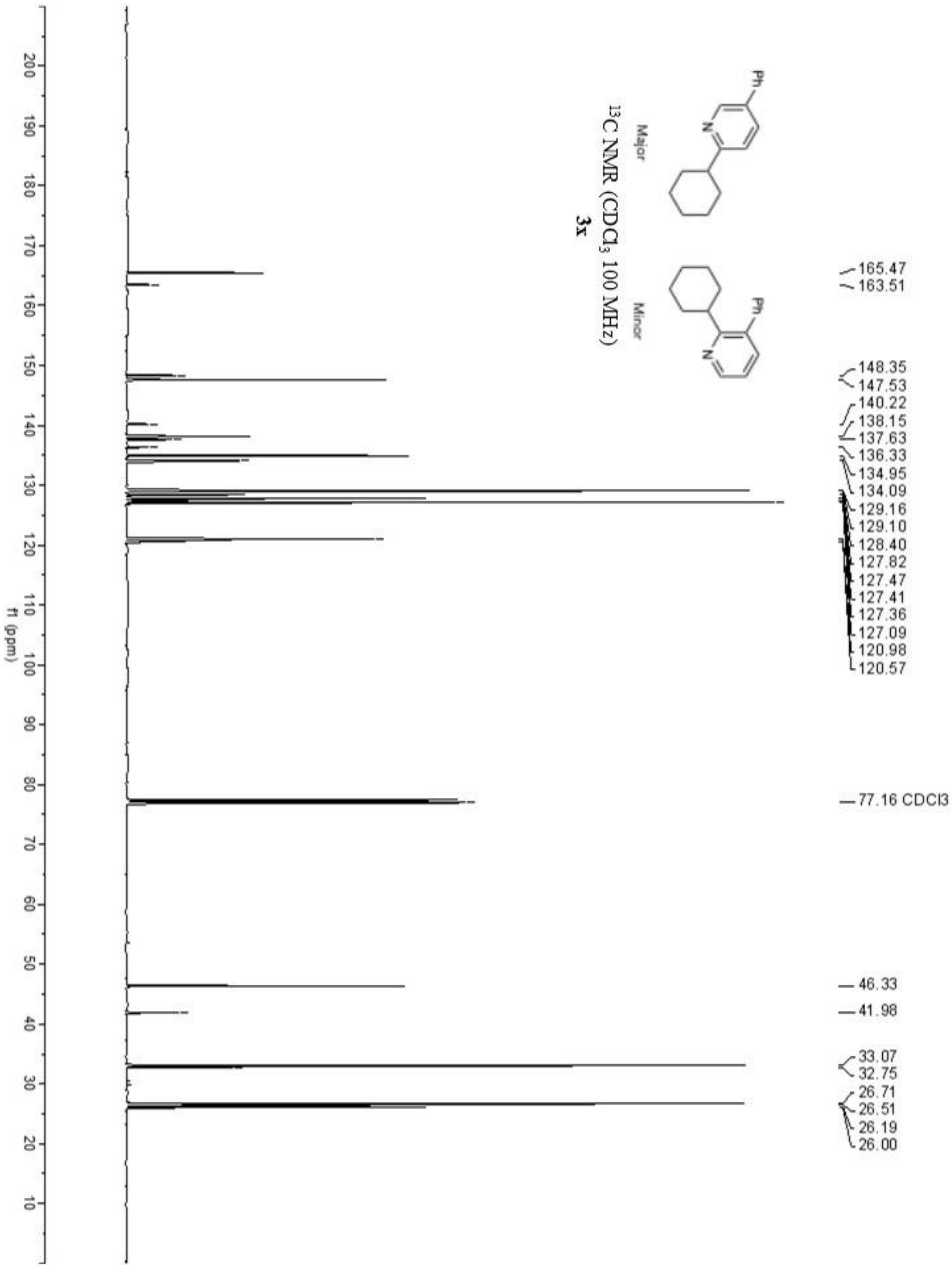

Major
Minor
13C NMR (CDCl3 100 MHz)
3x
165.47
163.51
148.35
147.53
140.22
138.15
137.63
136.33
134.95
134.09
129.16
129.10
128.40
127.82
127.47
127.41
127.36
127.09
120.98
120.57
77.16 CDCl3
46.33
41.98
33.07
32.75
26.71
26.51
26.19
26.00
ft (ppm)

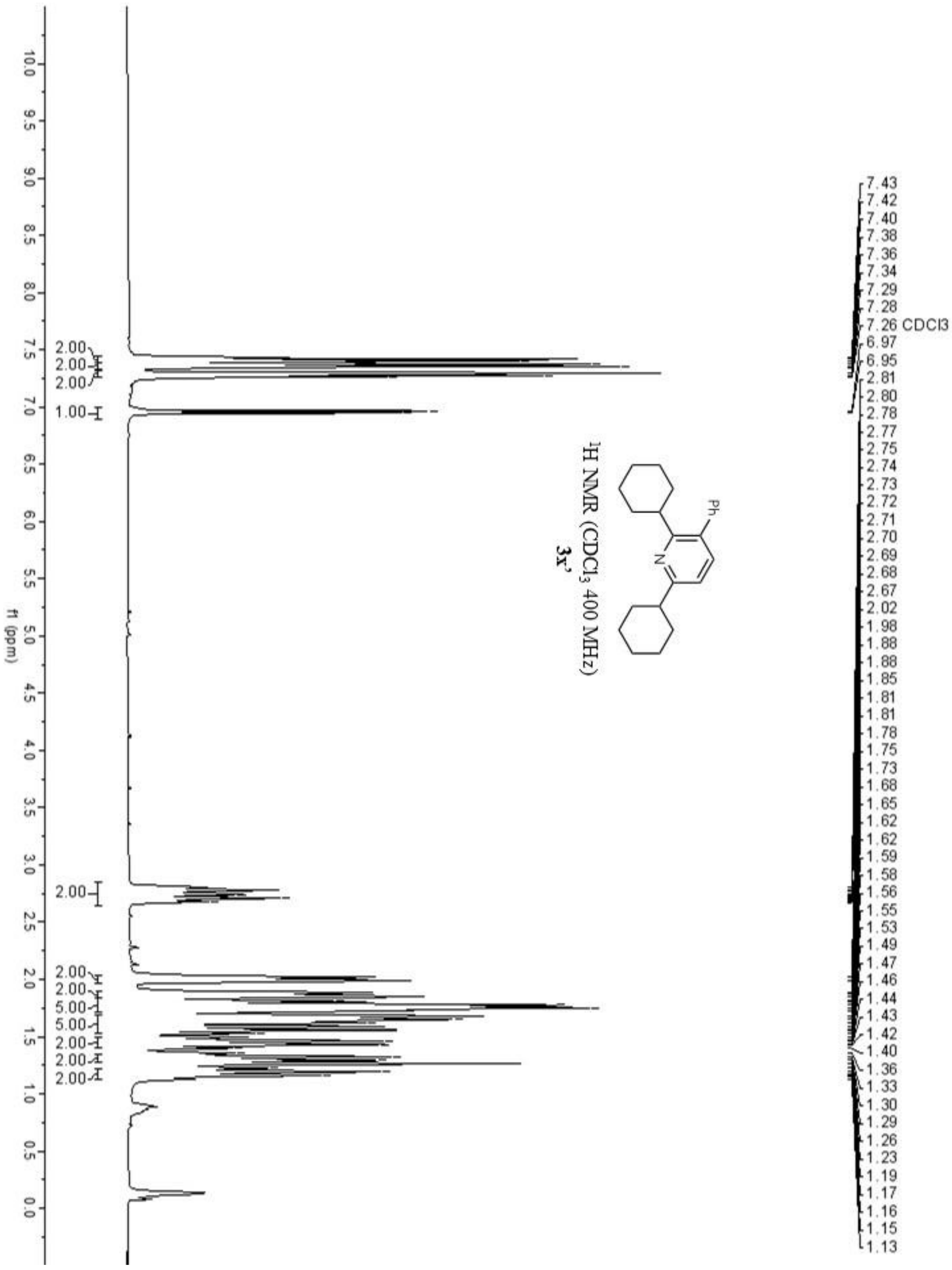

1H NMR (CDCl3 400 MHz)
3x'
Ph
N
7.43
7.42
7.40
7.38
7.36
7.34
7.29
7.28
7.26 CDCl3
6.97
6.95
2.81
2.80
2.78
2.77
2.75
2.74
2.73
2.72
2.71
2.70
2.69
2.68
2.67
2.02
1.98
1.88
1.88
1.85
1.81
1.81
1.78
1.75
1.73
1.68
1.65
1.62
1.62
1.59
1.58
1.56
1.55
1.53
1.49
1.47
1.46
1.44
1.43
1.42
1.40
1.36
1.33
1.30
1.29
1.26
1.23
1.19
1.17
1.16
1.15
1.13
f1 (ppm)

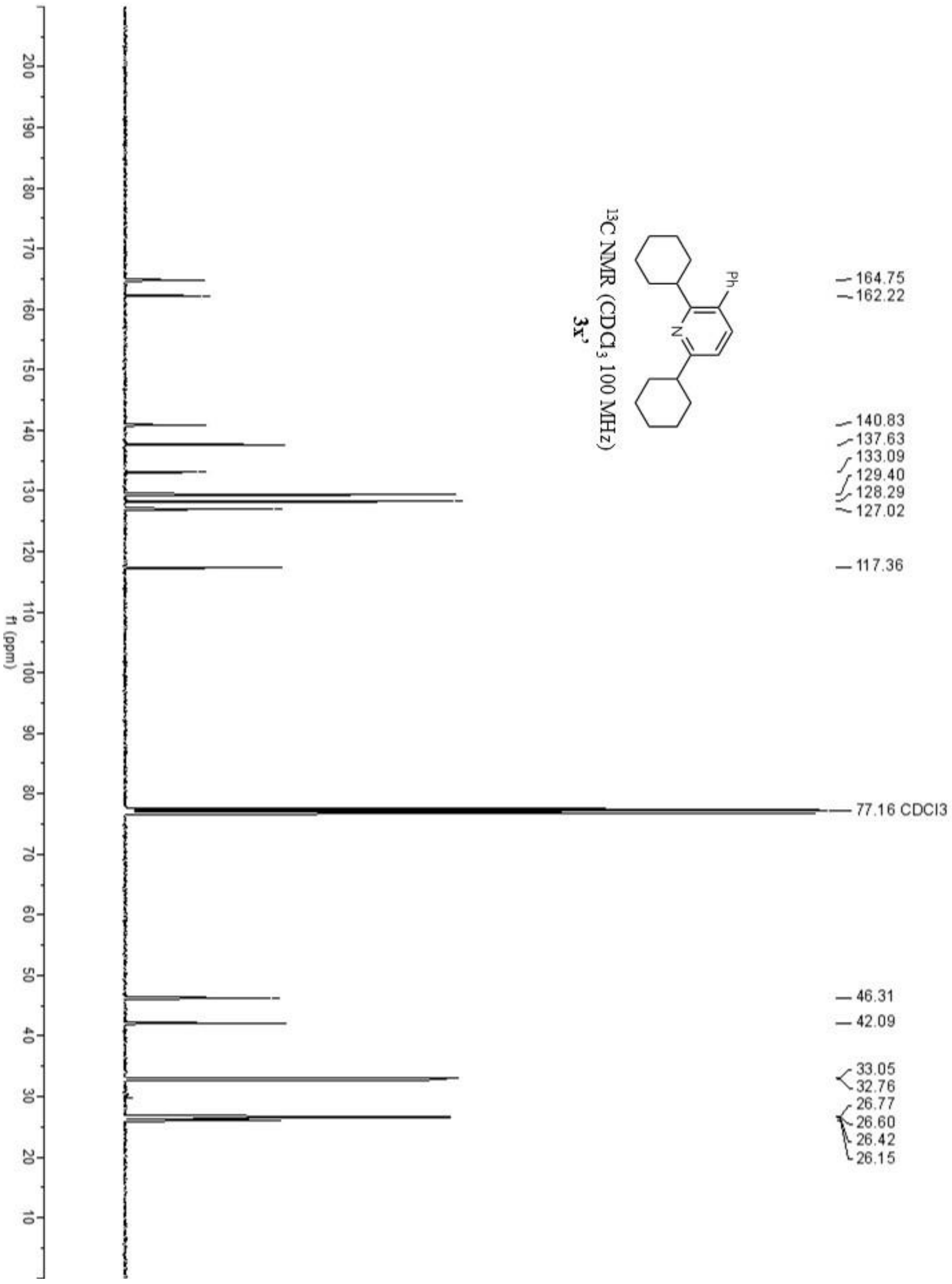

13C NMR (CDCl3 100 MHz)
3x'
Ph
N
164.75
162.22
140.83
137.63
133.09
129.40
128.29
127.02
117.36
77.16 CDCl3
46.31
42.09
33.05
32.76
26.77
26.60
26.42
26.15
f1 (ppm)

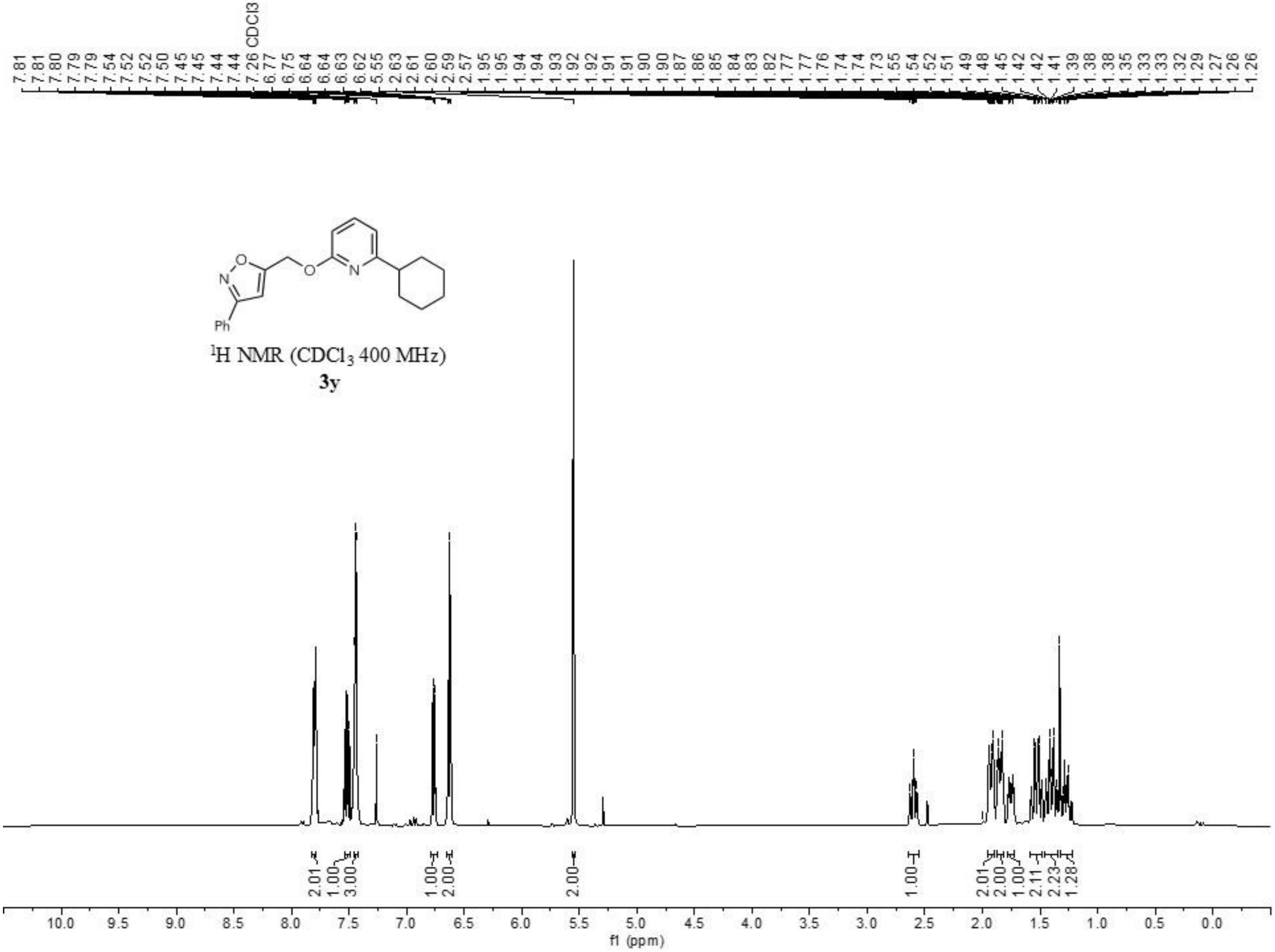
7.81 7.81 7.80 7.79 7.79 7.54 7.52 7.52 7.50 7.45 7.45 7.44 7.44 7.26 CDCl3 6.77 6.75 6.64 6.64 6.63 6.62 5.55 2.63 2.61 2.60 2.59 2.57 1.95 1.95 1.94 1.94 1.93 1.92 1.92 1.91 1.91 1.90 1.90 1.87 1.86 1.85 1.84 1.83 1.82 1.77 1.77 1.76 1.74 1.74 1.73 1.55 1.54 1.52 1.51 1.49 1.48 1.45 1.42 1.42 1.41 1.39 1.38 1.38 1.35 1.33 1.33 1.32 1.29 1.27 1.26 1.26
Ph
N
O
N
^{1}H NMR (CDCl$_3$ 400 MHz)
3y
2.01 1.00 3.00 1.00 2.00 2.00 1.00 2.01 2.00 1.00 2.11 2.23 1.28
10.0 9.5 9.0 8.5 8.0 7.5 7.0 6.5 6.0 5.5 5.0 4.5 4.0 3.5 3.0 2.5 2.0 1.5 1.0 0.5 0.0
f1 (ppm)

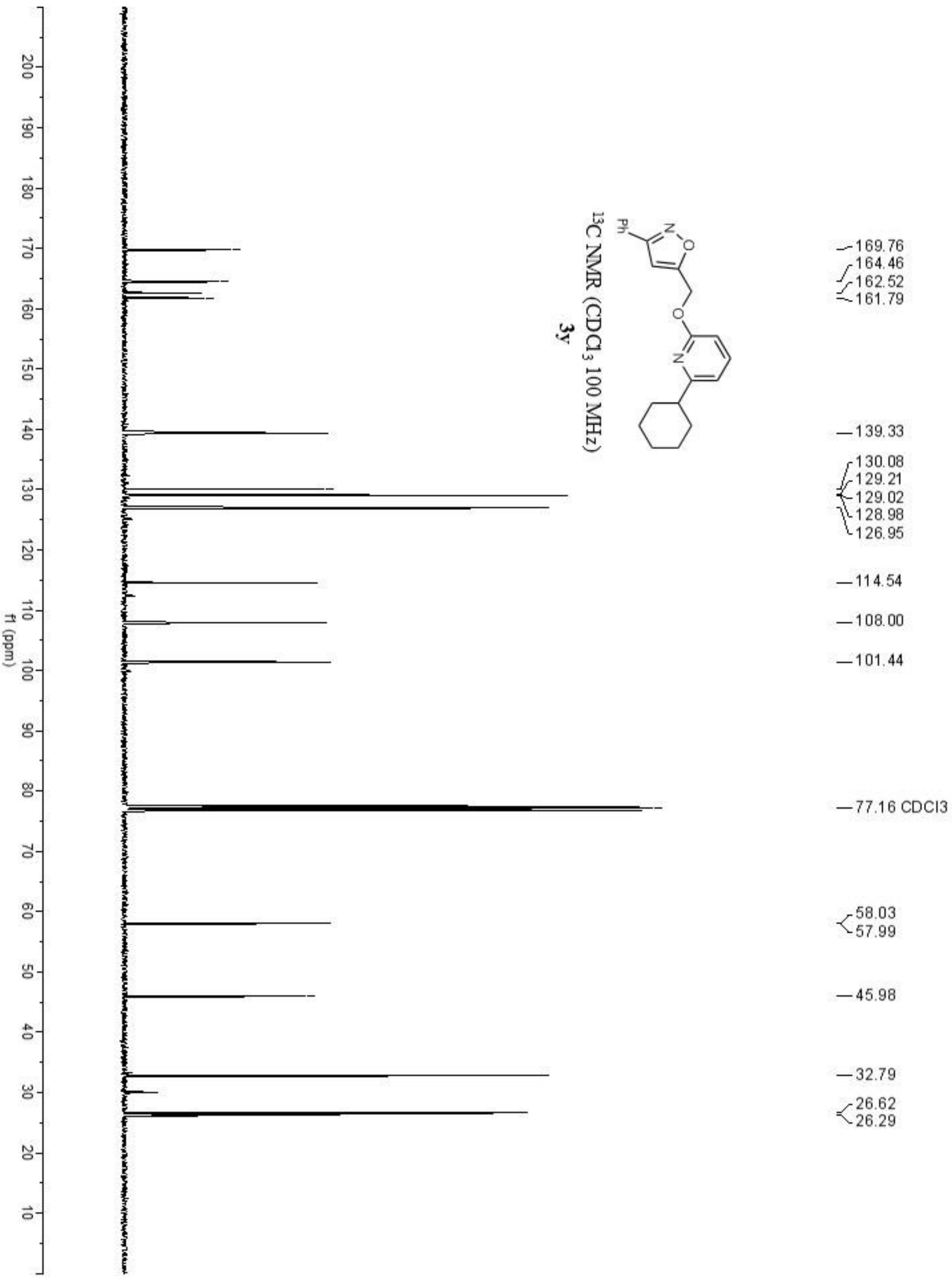

13C NMR (CDCl3 100 MHz)
3y
Ph
N
O
169.76
164.46
162.52
161.79
139.33
130.08
129.21
129.02
128.98
126.95
114.54
108.00
101.44
77.16 CDCl3
58.03
57.99
45.98
32.79
26.62
26.29
f1 (ppm)

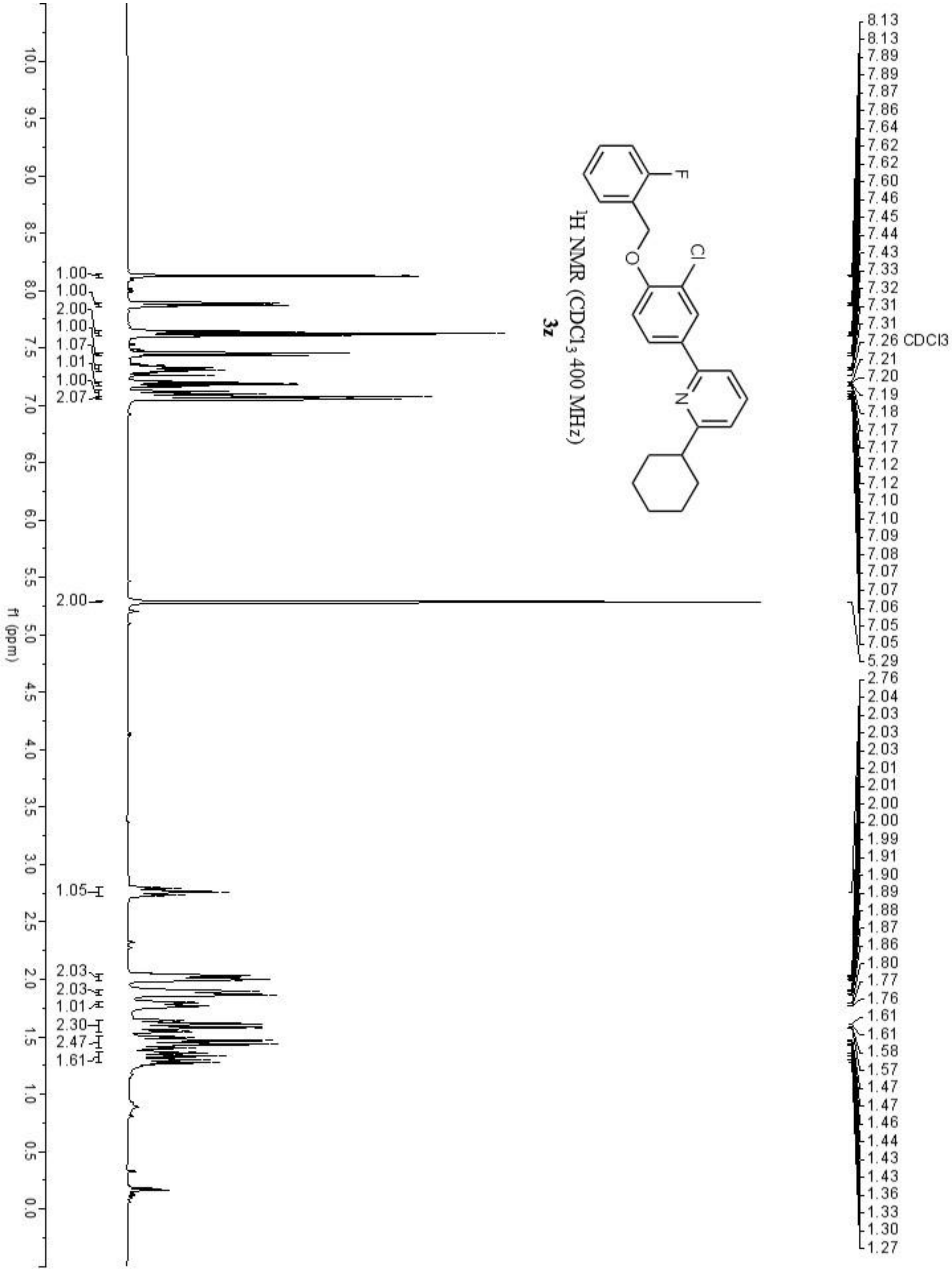

1H NMR (CDCl3 400 MHz)
3z
F
Cl
O
N
8.13
8.13
7.89
7.89
7.87
7.86
7.64
7.62
7.62
7.60
7.46
7.45
7.44
7.43
7.33
7.32
7.31
7.31
7.26 CDCl3
7.21
7.20
7.19
7.18
7.17
7.17
7.12
7.12
7.10
7.10
7.09
7.08
7.07
7.07
7.06
7.05
7.05
5.29
2.76
2.04
2.03
2.03
2.03
2.01
2.01
2.00
2.00
1.99
1.91
1.90
1.89
1.88
1.87
1.86
1.80
1.77
1.76
1.61
1.61
1.58
1.57
1.47
1.47
1.46
1.44
1.43
1.43
1.36
1.33
1.30
1.27
1.00
1.00
2.00
1.00
1.07
1.01
1.00
2.07
2.00
1.05
2.03
2.03
1.01
2.30
2.47
1.61
f1 (ppm)
10.0
9.5
9.0
8.5
8.0
7.5
7.0
6.5
6.0
5.5
5.0
4.5
4.0
3.5
3.0
2.5
2.0
1.5
1.0
0.5
0.0

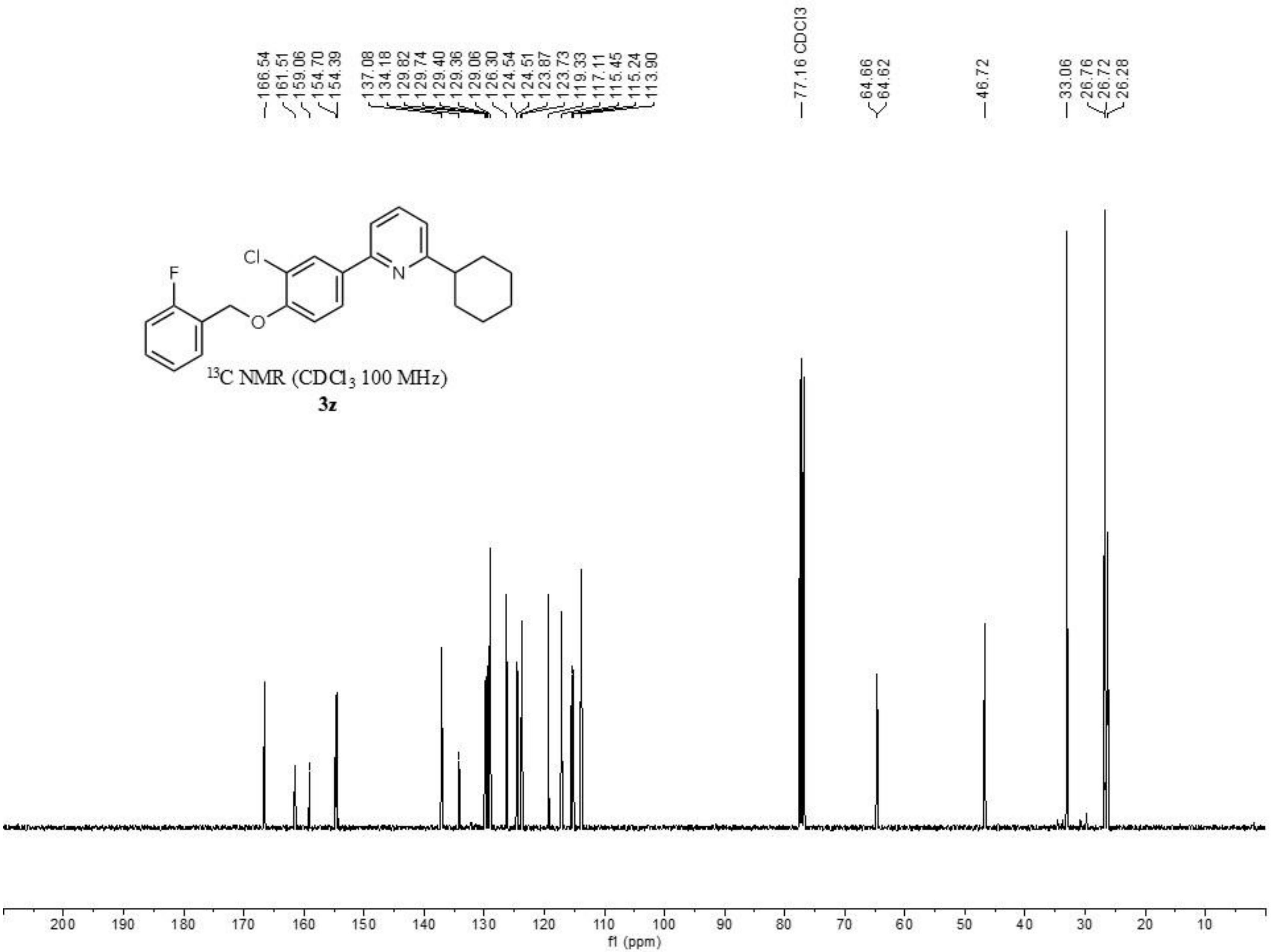

^{13}C NMR ($CDCl_3$ 100 MHz)

3z

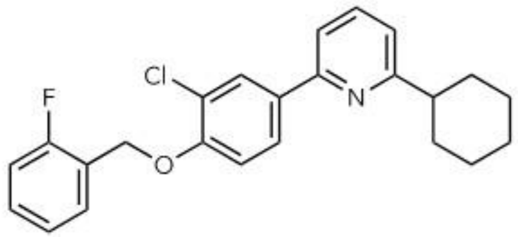

^{19}F NMR ($CDCl_3$ 376 MHz)

3z

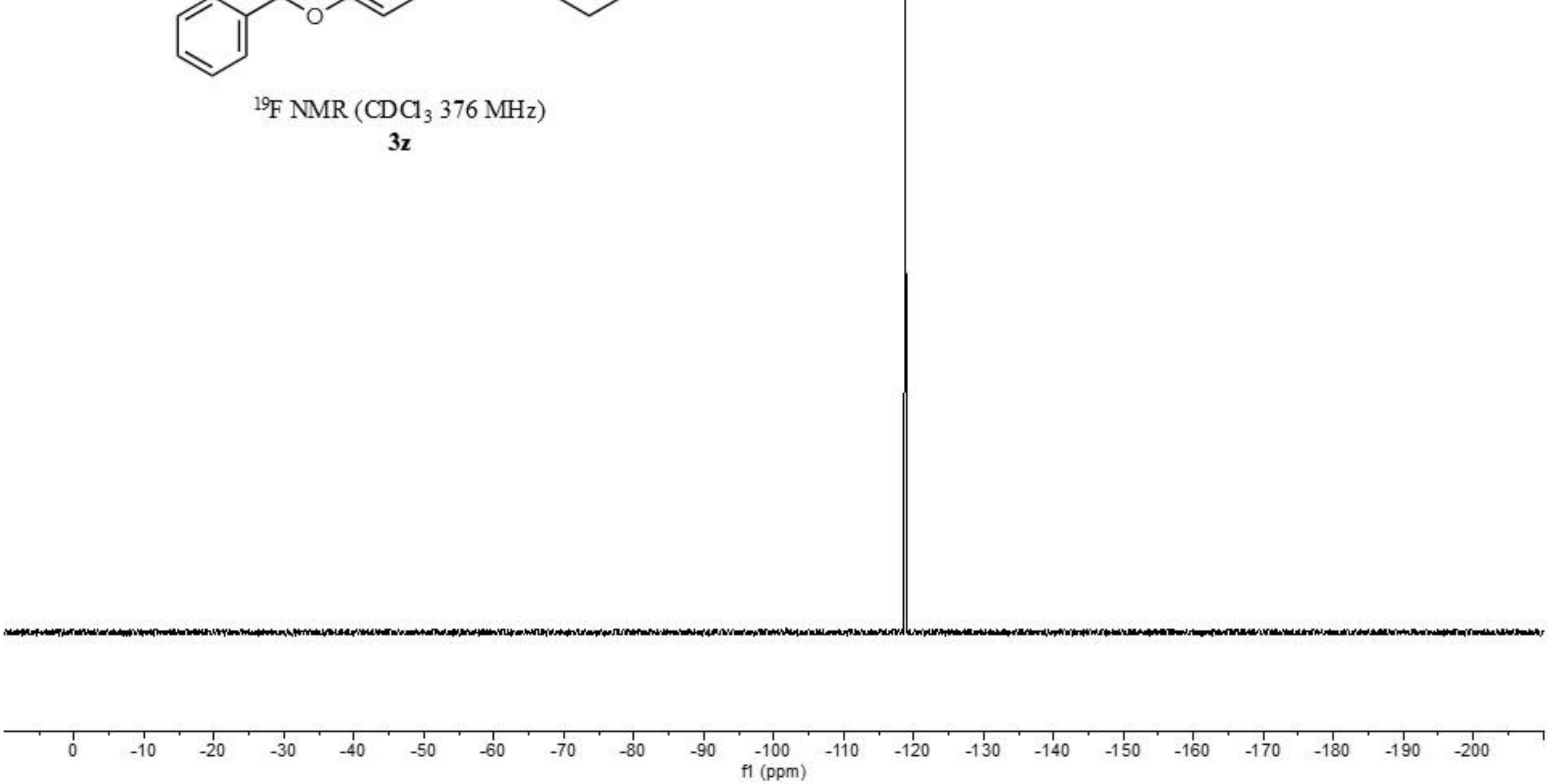

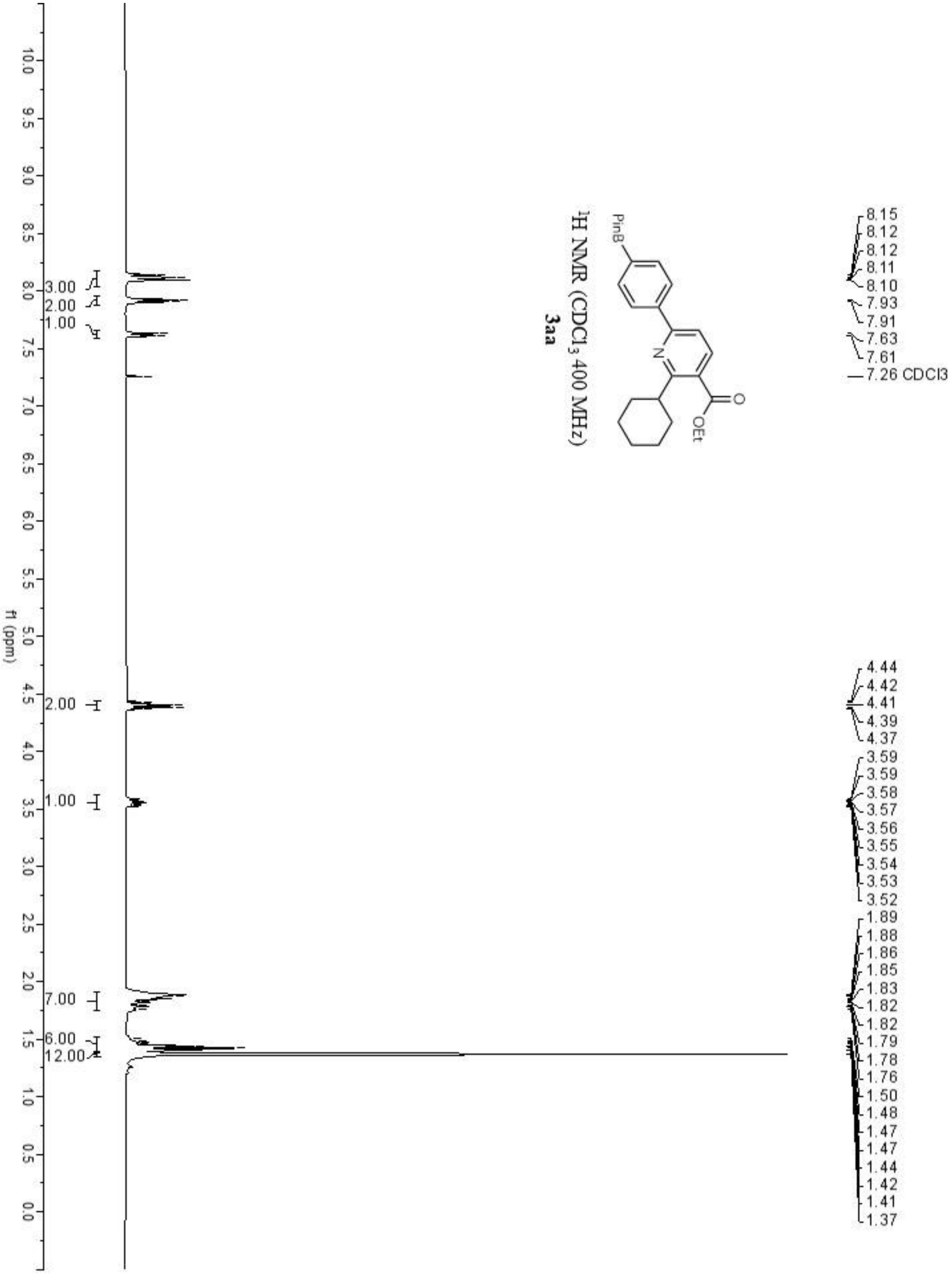

^{1}H NMR ($CDCl_3$ 400 MHz)

3aa

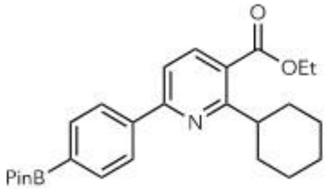

^{13}C NMR ($CDCl_3$ 100 MHz)
3aa

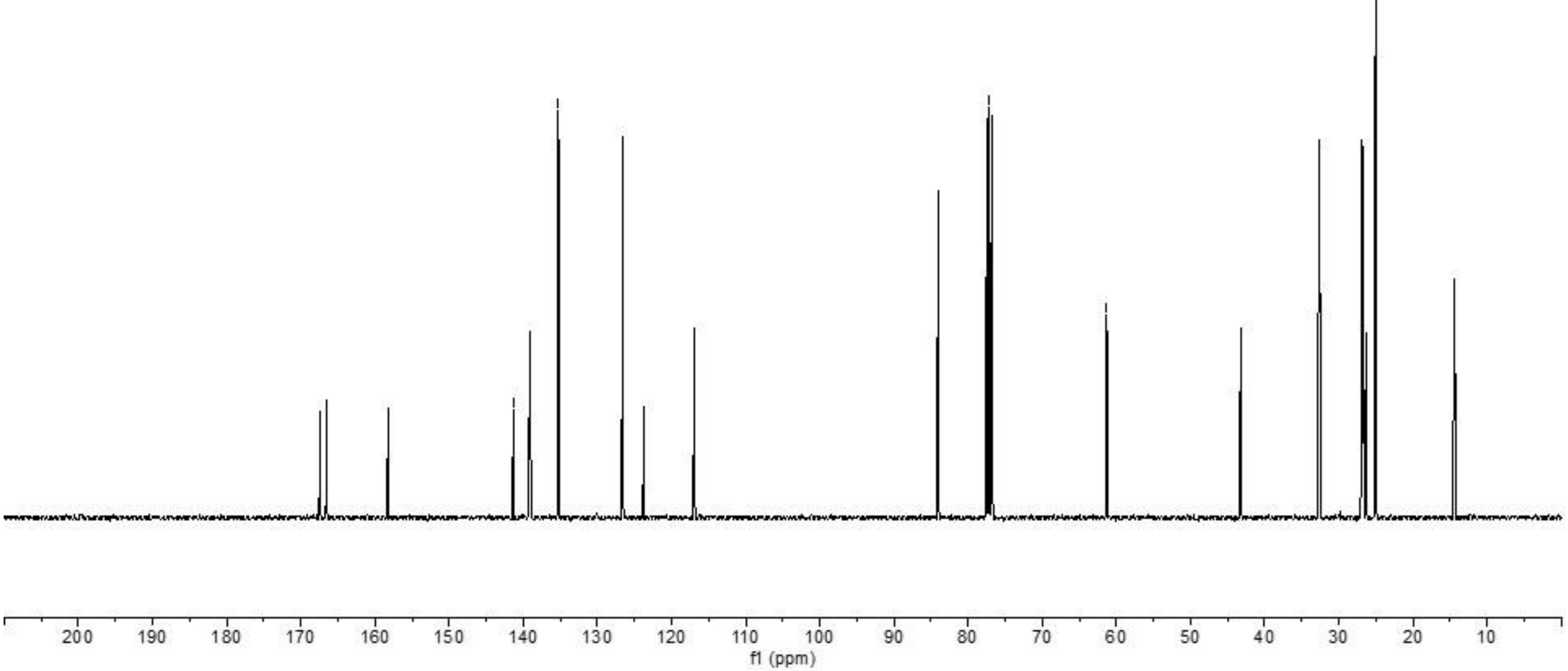

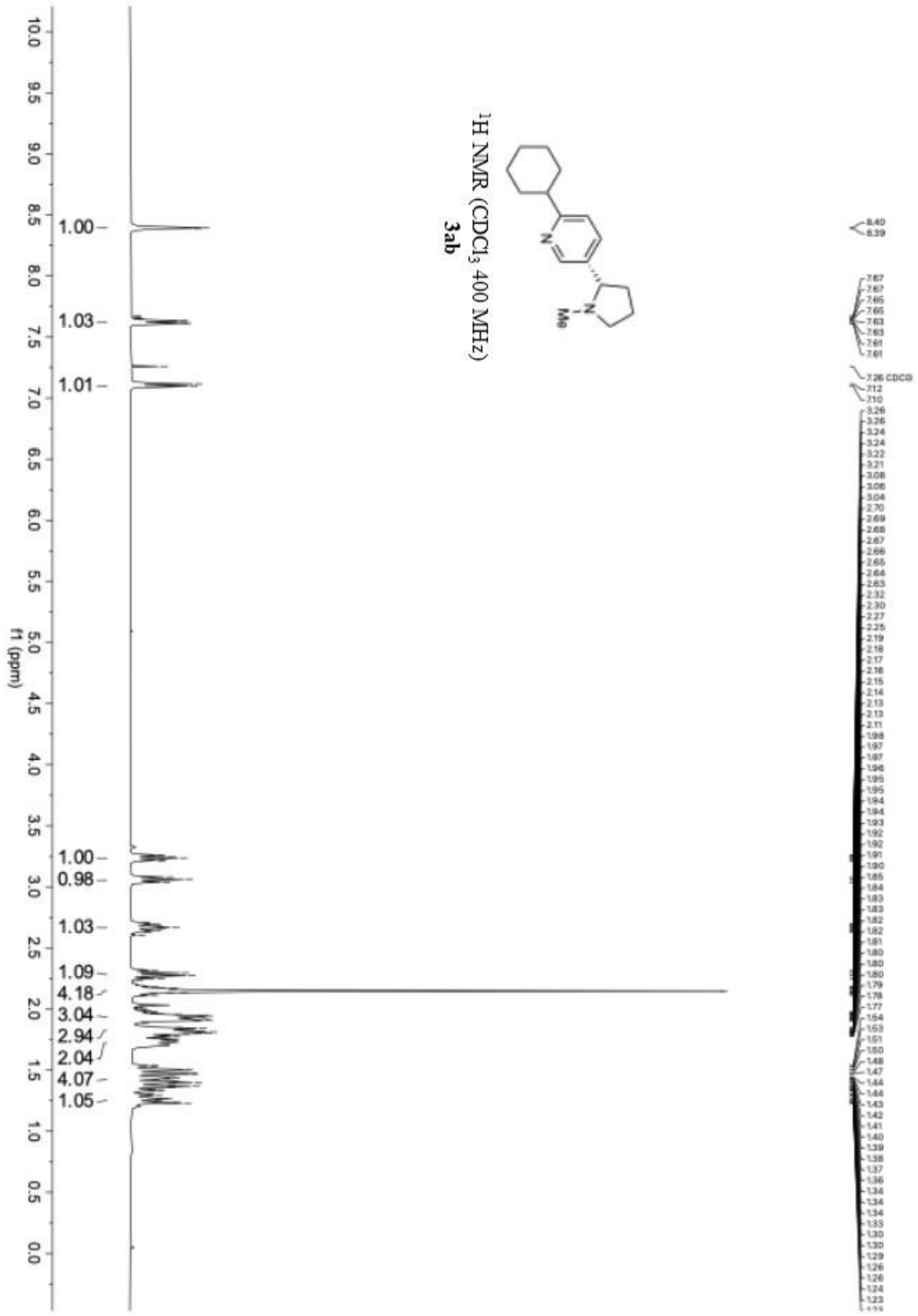
1H NMR (CDCl3 400 MHz)
3ab
f1 (ppm)

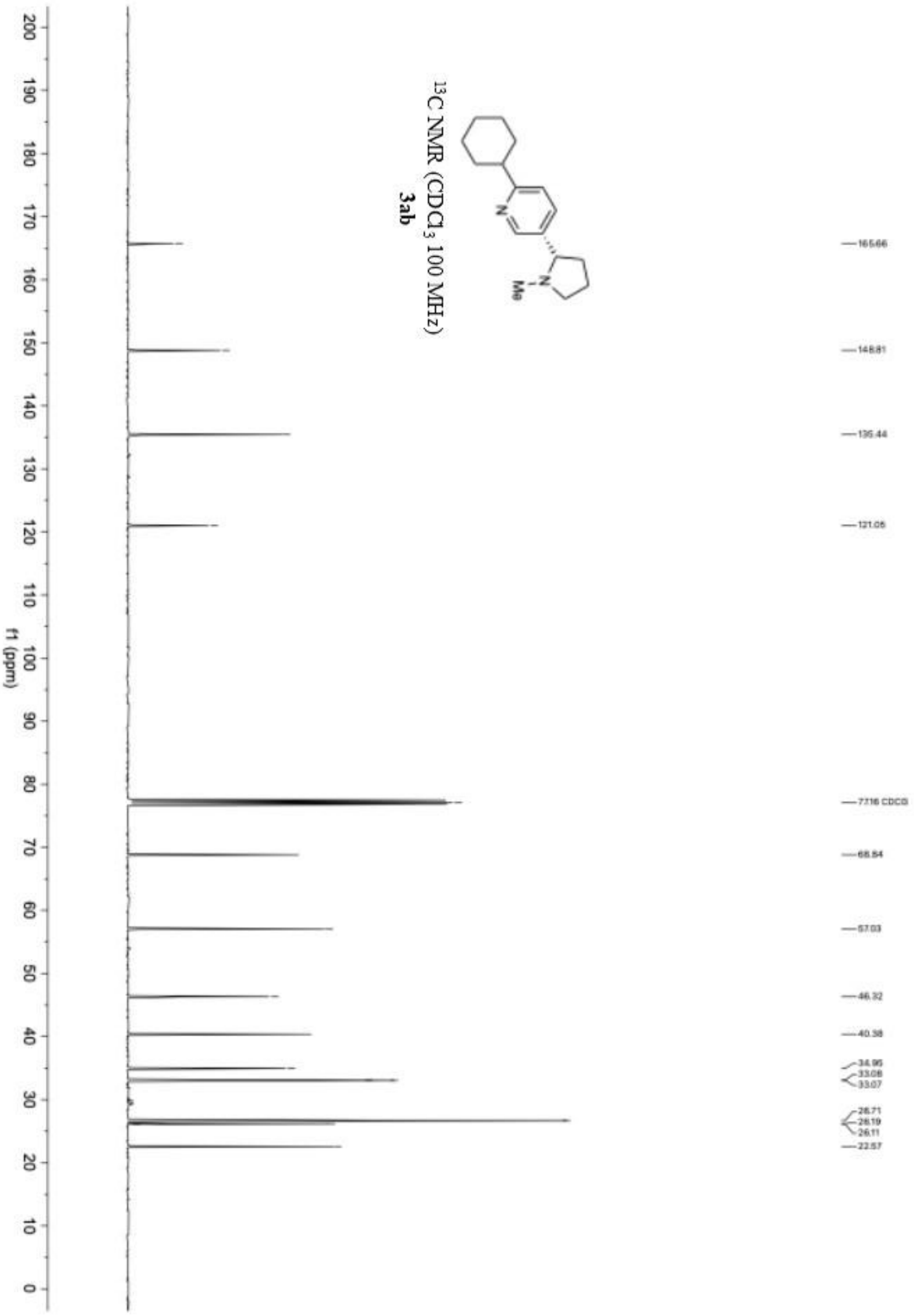

13C NMR (CDCl3 100 MHz)
3ab
N
N
Me
165.66
148.81
135.44
121.05
77.16 CDCl3
68.84
57.03
46.32
40.38
34.95
33.08
33.07
26.71
26.19
26.11
22.57
f1 (ppm)
200
190
180
170
160
150
140
130
120
110
100
90
80
70
60
50
40
30
20
10
0

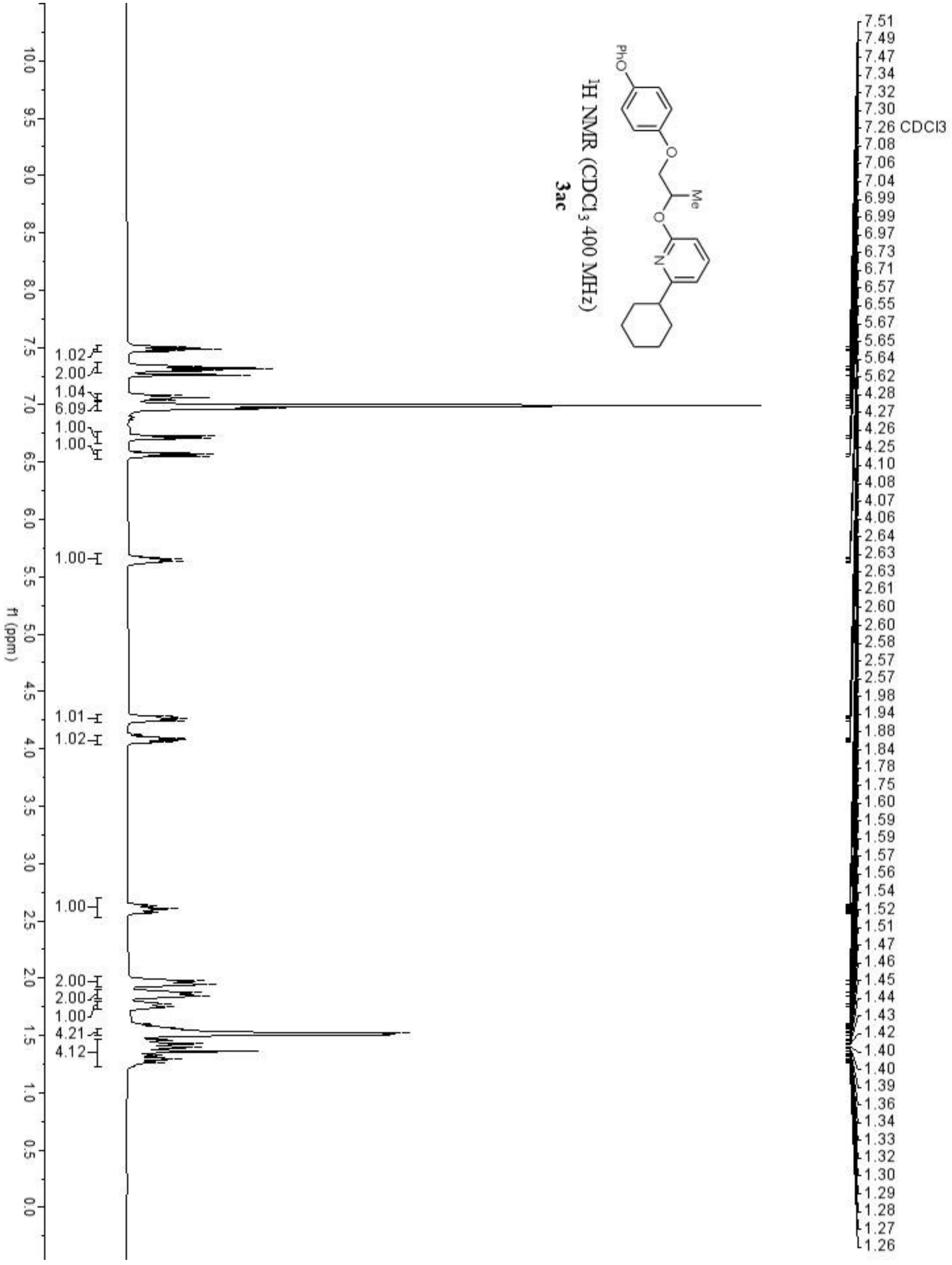

1H NMR (CDCl3 400 MHz)
3ac
PhO
Me
N
O
7.51
7.49
7.47
7.34
7.32
7.30
7.26 CDCl3
7.08
7.06
7.04
6.99
6.99
6.97
6.73
6.71
6.57
6.55
5.67
5.65
5.64
5.62
4.28
4.27
4.26
4.25
4.10
4.08
4.07
4.06
2.64
2.63
2.63
2.61
2.60
2.60
2.58
2.57
2.57
1.98
1.94
1.88
1.84
1.78
1.75
1.60
1.59
1.59
1.57
1.56
1.54
1.52
1.51
1.47
1.46
1.45
1.44
1.43
1.42
1.40
1.40
1.39
1.36
1.34
1.33
1.32
1.30
1.29
1.28
1.27
1.26
1.02
2.00
1.04
6.09
1.00
1.00
1.00
1.01
1.02
1.00
2.00
2.00
1.00
4.21
4.12
f1 (ppm)

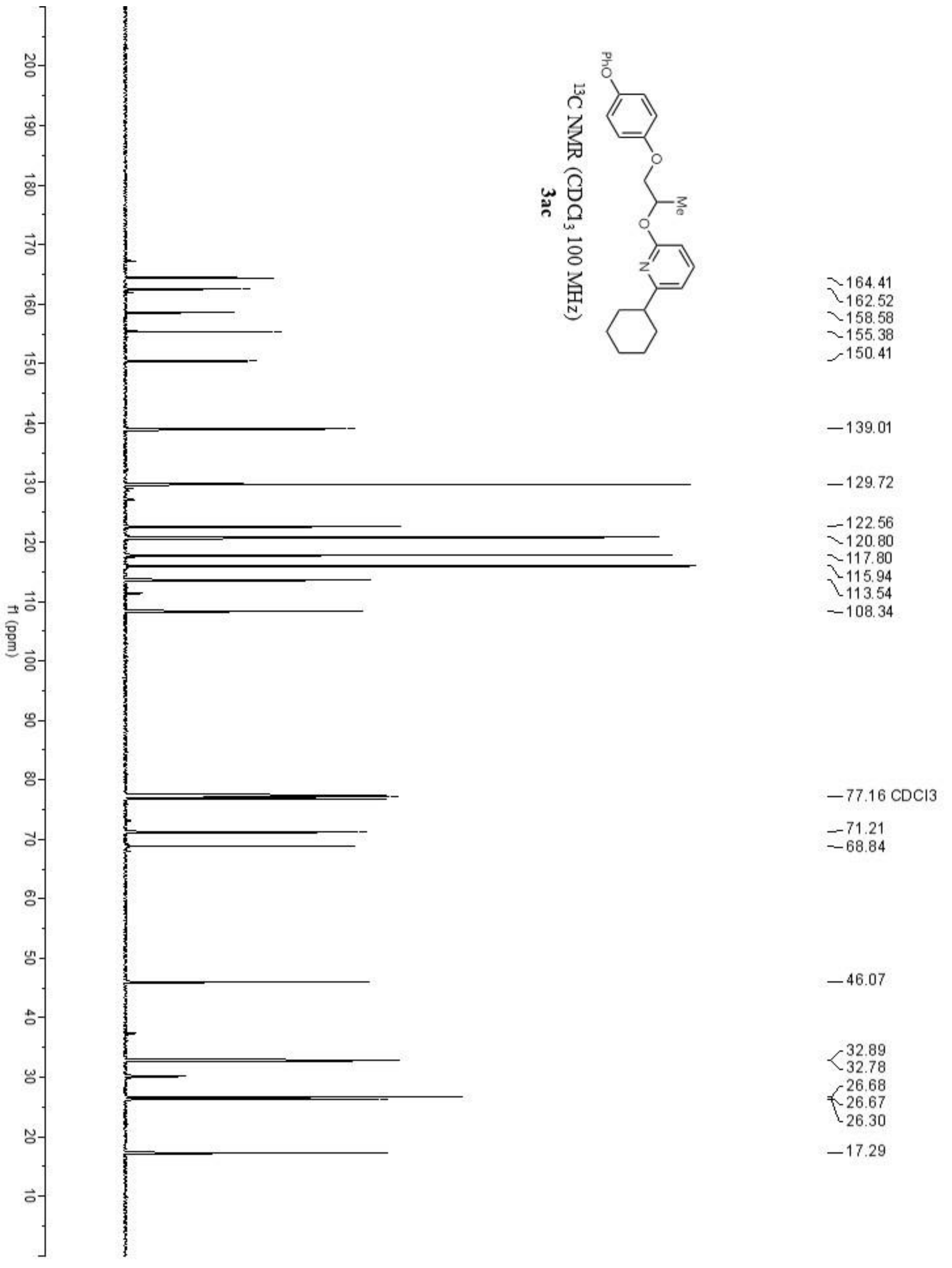

PhO
Me
N
^{13}C NMR (CDCl$_3$ 100 MHz)
3ac
164.41
162.52
158.58
155.38
150.41
139.01
129.72
122.56
120.80
117.80
115.94
113.54
108.34
77.16 CDCl3
71.21
68.84
46.07
32.89
32.78
26.68
26.67
26.30
17.29
f1 (ppm)

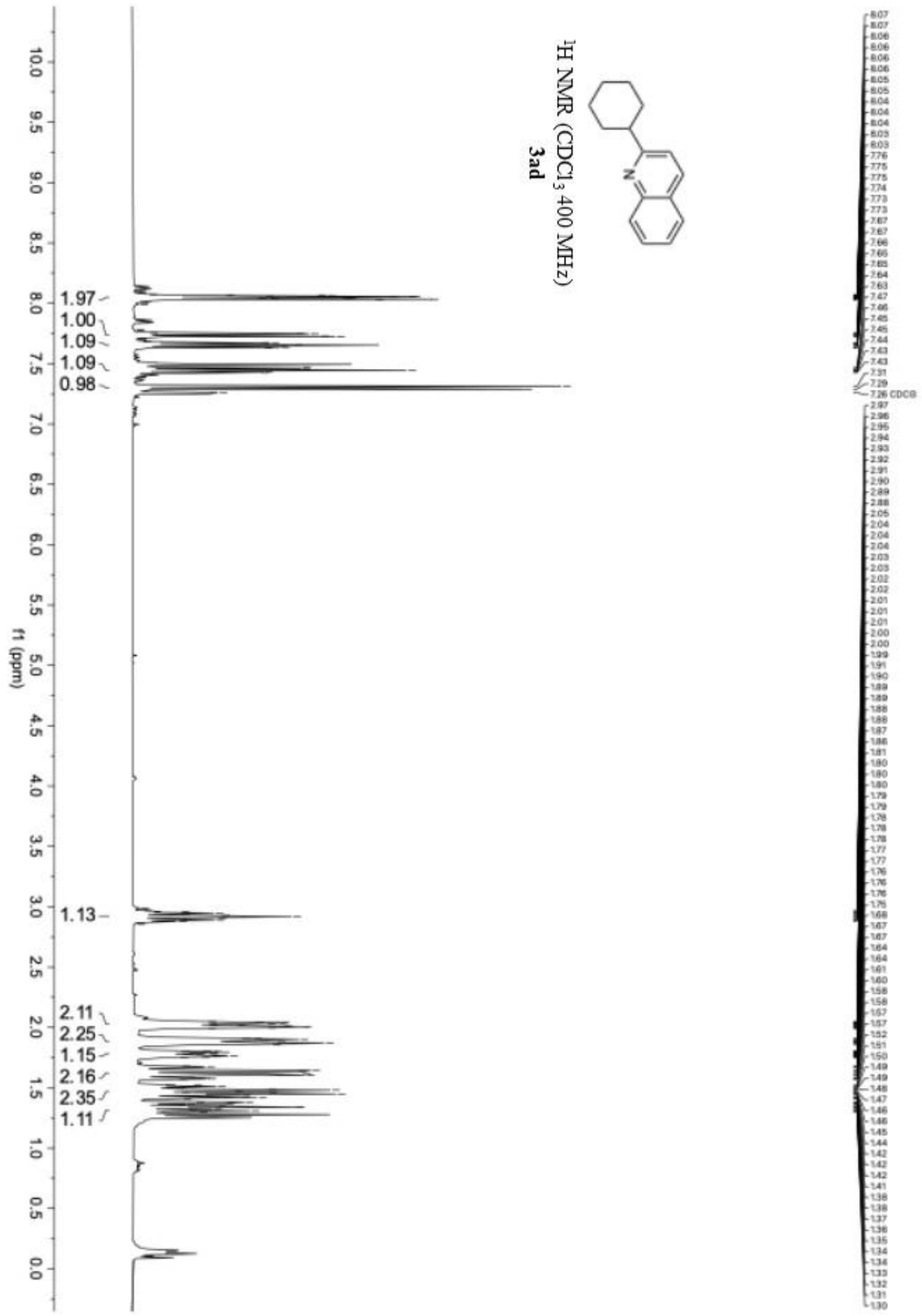

1H NMR (CDCl3 400 MHz)
3ad
f1 (ppm)

^{13}C NMR ($CDCl_3$ 100 MHz)
3ad

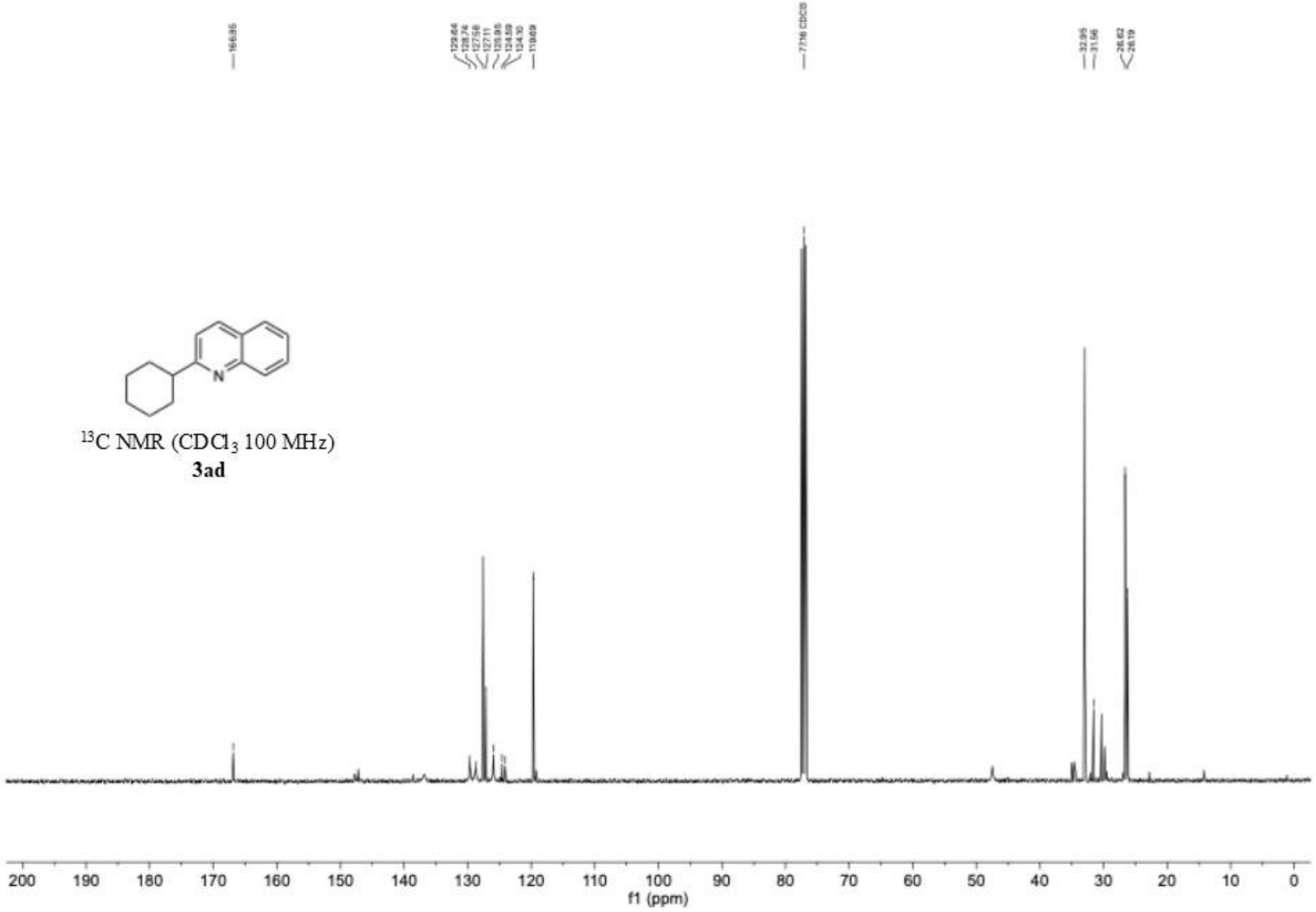

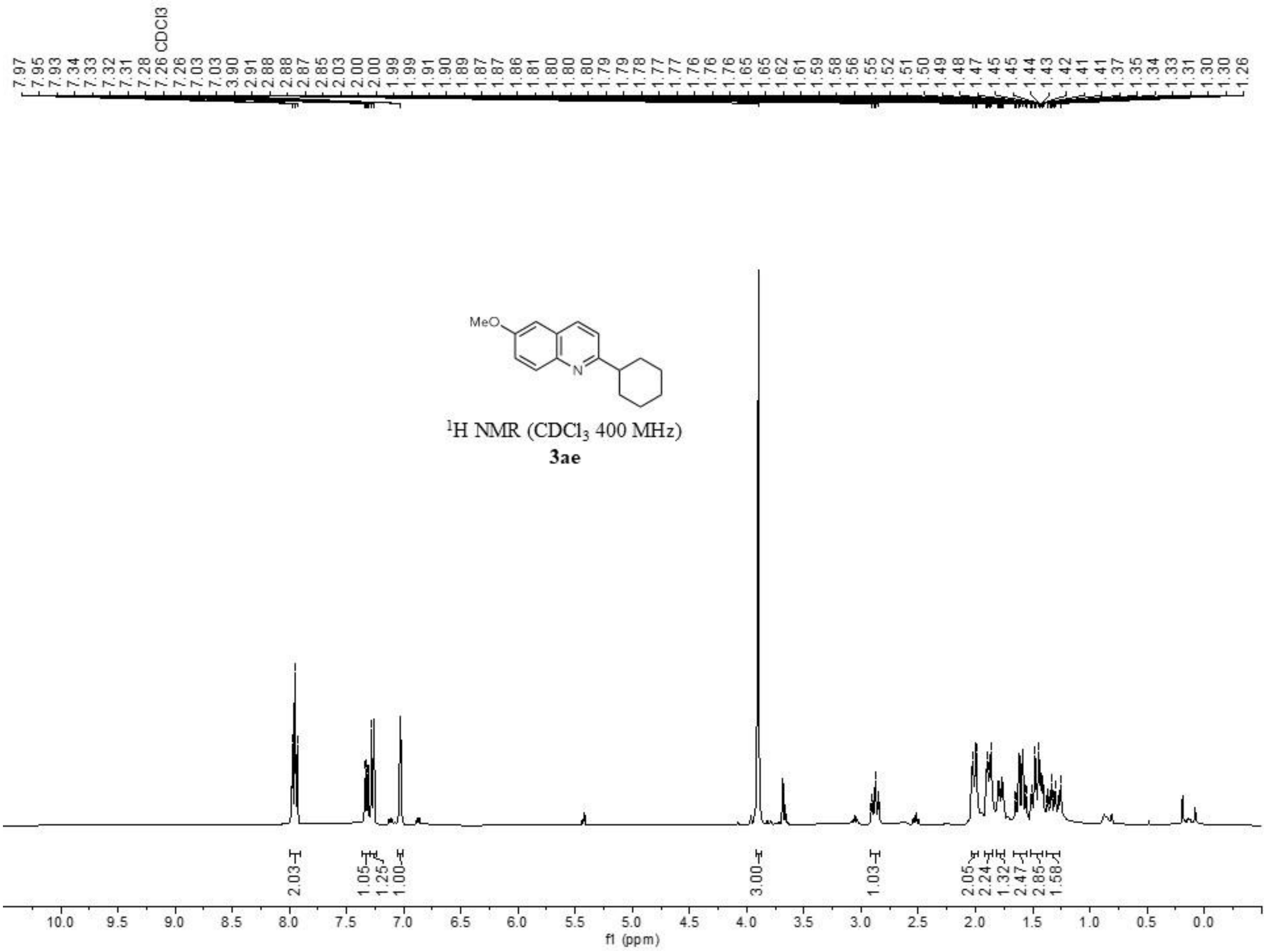
7.97 7.95 7.93 7.34 7.33 7.32 7.31 7.28 7.26 CDCl3 7.26 7.03 7.03 3.90 2.91 2.88 2.88 2.87 2.85 2.03 2.00 2.00 1.99 1.99 1.91 1.90 1.89 1.87 1.87 1.86 1.81 1.80 1.80 1.80 1.79 1.79 1.78 1.77 1.77 1.76 1.76 1.76 1.65 1.65 1.62 1.61 1.59 1.58 1.56 1.55 1.52 1.51 1.50 1.49 1.48 1.47 1.45 1.45 1.44 1.43 1.42 1.41 1.41 1.37 1.35 1.34 1.33 1.31 1.30 1.30 1.26
MeO
N
¹H NMR (CDCl3 400 MHz)
3ae
2.03 1.05 1.25 1.00 3.00 1.03 2.05 2.24 1.32 2.47 2.85 1.58
10.0 9.5 9.0 8.5 8.0 7.5 7.0 6.5 6.0 5.5 5.0 4.5 4.0 3.5 3.0 2.5 2.0 1.5 1.0 0.5 0.0
f1 (ppm)

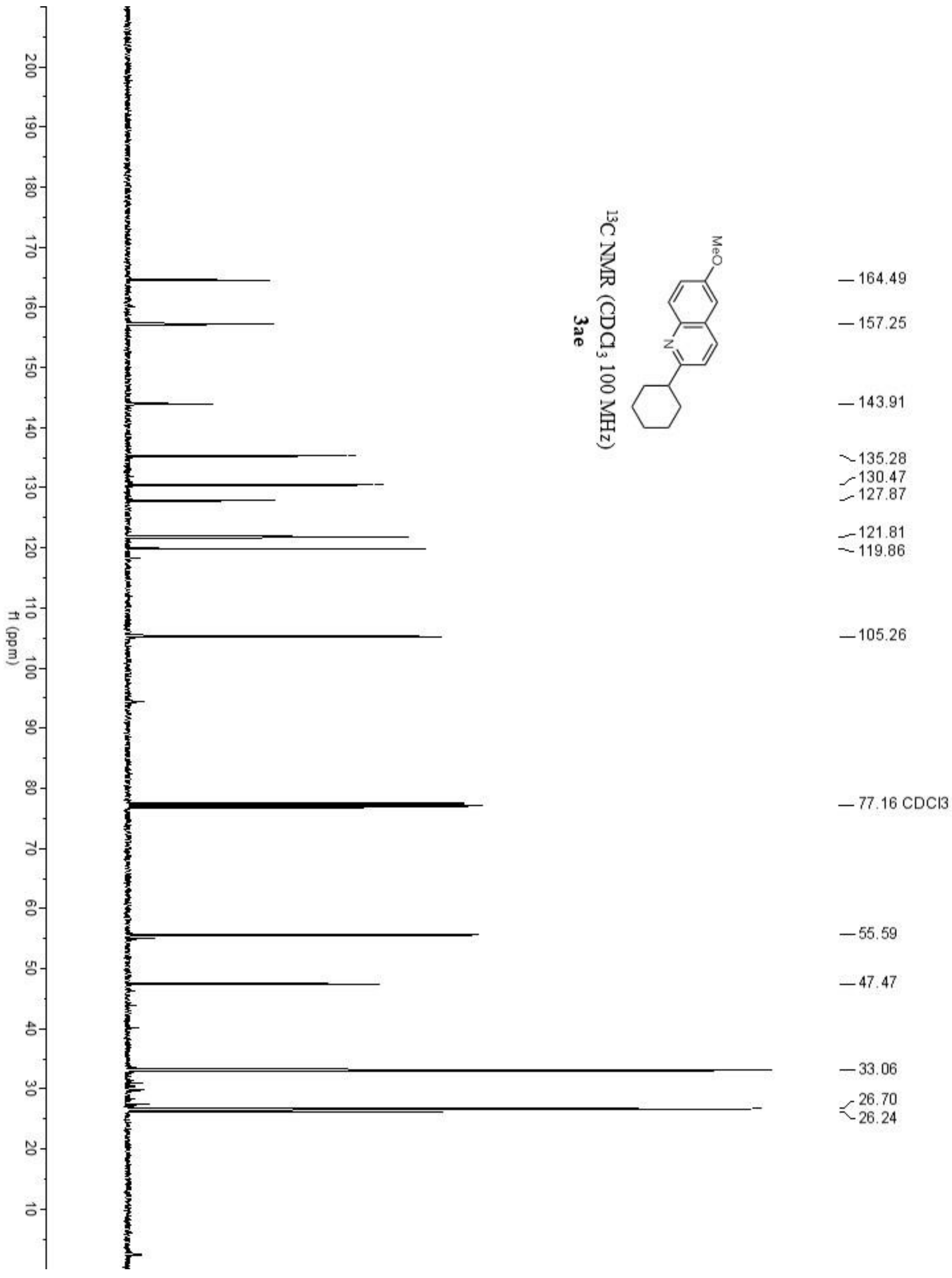

13C NMR (CDCl3 100 MHz)
3ae
MeO
N
164.49
157.25
143.91
135.28
130.47
127.87
121.81
119.86
105.26
77.16 CDCl3
55.59
47.47
33.06
26.70
26.24
f1 (ppm)

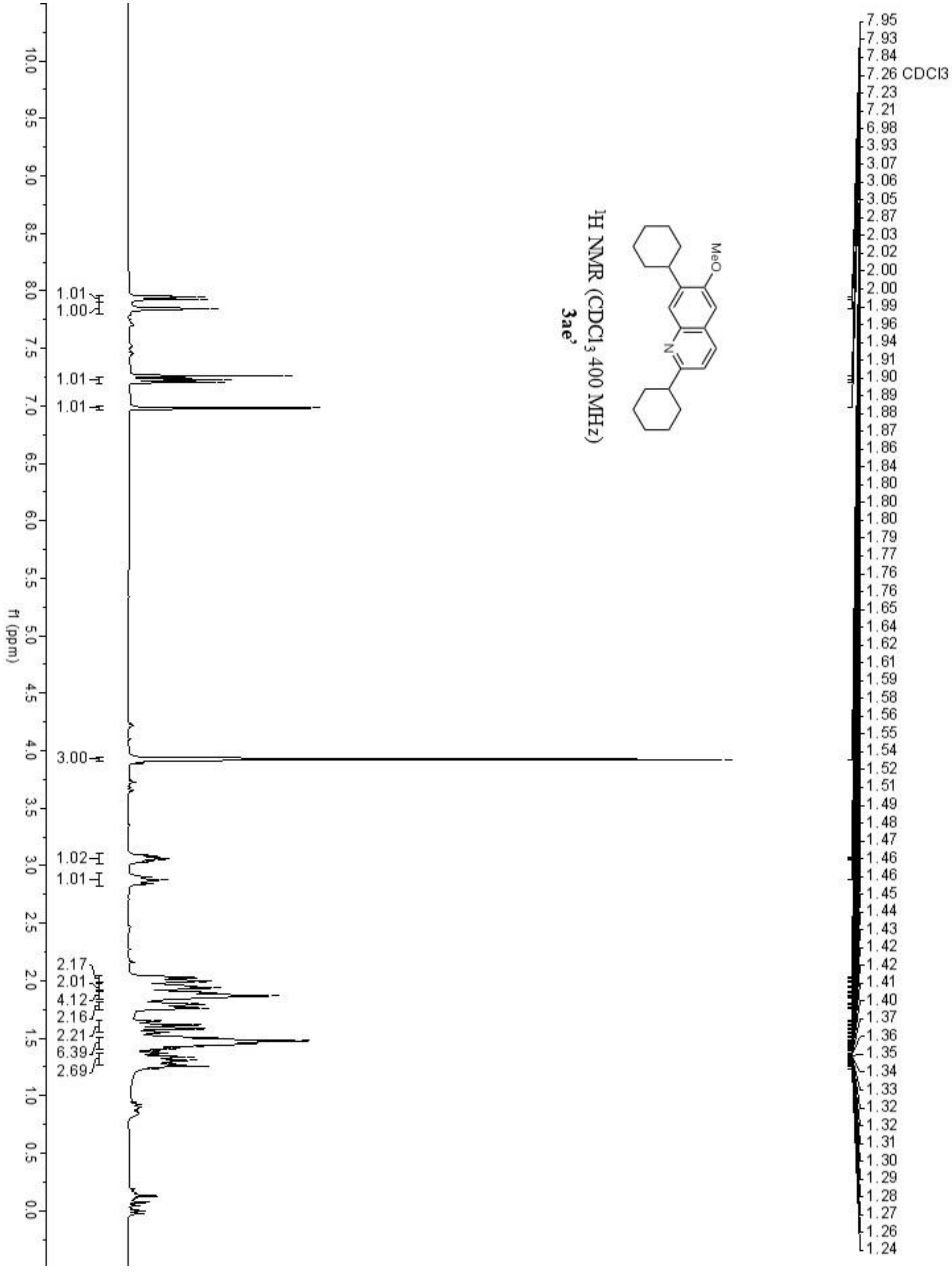

¹H NMR (CDCl₃ 400 MHz)
3ae'
MeO
N
7.95 7.93 7.84 7.26 CDCl3 7.23 7.21 6.98 3.93 3.07 3.06 3.05 2.87 2.03 2.02 2.00 2.00 1.99 1.96 1.94 1.91 1.90 1.89 1.88 1.87 1.86 1.84 1.80 1.80 1.80 1.79 1.77 1.76 1.76 1.65 1.64 1.62 1.61 1.59 1.58 1.56 1.55 1.54 1.52 1.51 1.49 1.48 1.47 1.46 1.46 1.45 1.44 1.43 1.42 1.42 1.41 1.40 1.37 1.36 1.35 1.34 1.33 1.32 1.32 1.31 1.30 1.29 1.28 1.27 1.26 1.24
1.01 1.00 1.01 1.01 3.00 1.02 1.01 2.17 2.01 4.12 2.16 2.21 6.39 2.69
f1 (ppm)

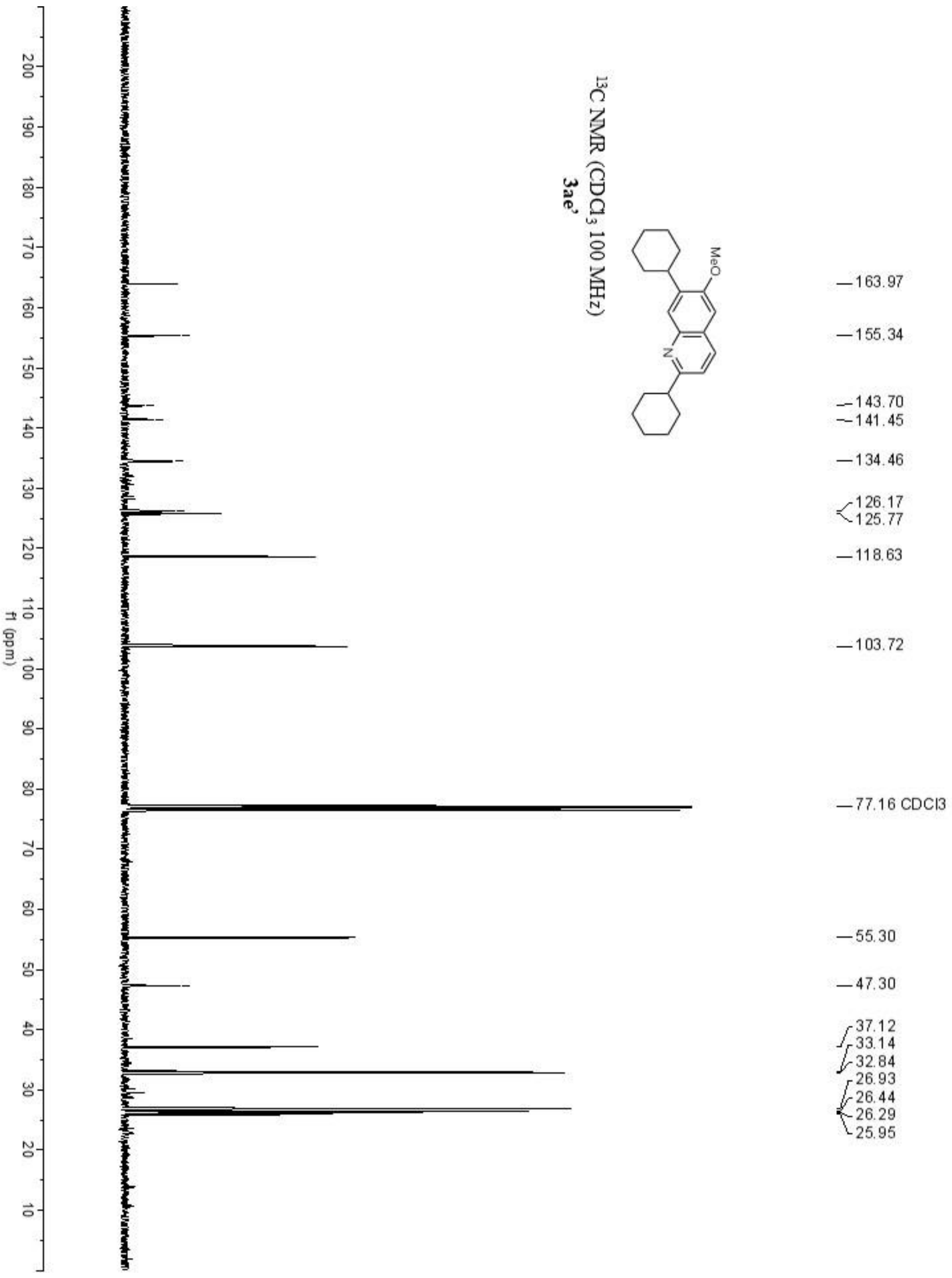

13C NMR (CDCl3 100 MHz)
3ae'
MeO
N
163.97
155.34
143.70
141.45
134.46
126.17
125.77
118.63
103.72
77.16 CDCl3
55.30
47.30
37.12
33.14
32.84
26.93
26.44
26.29
25.95
f1 (ppm)

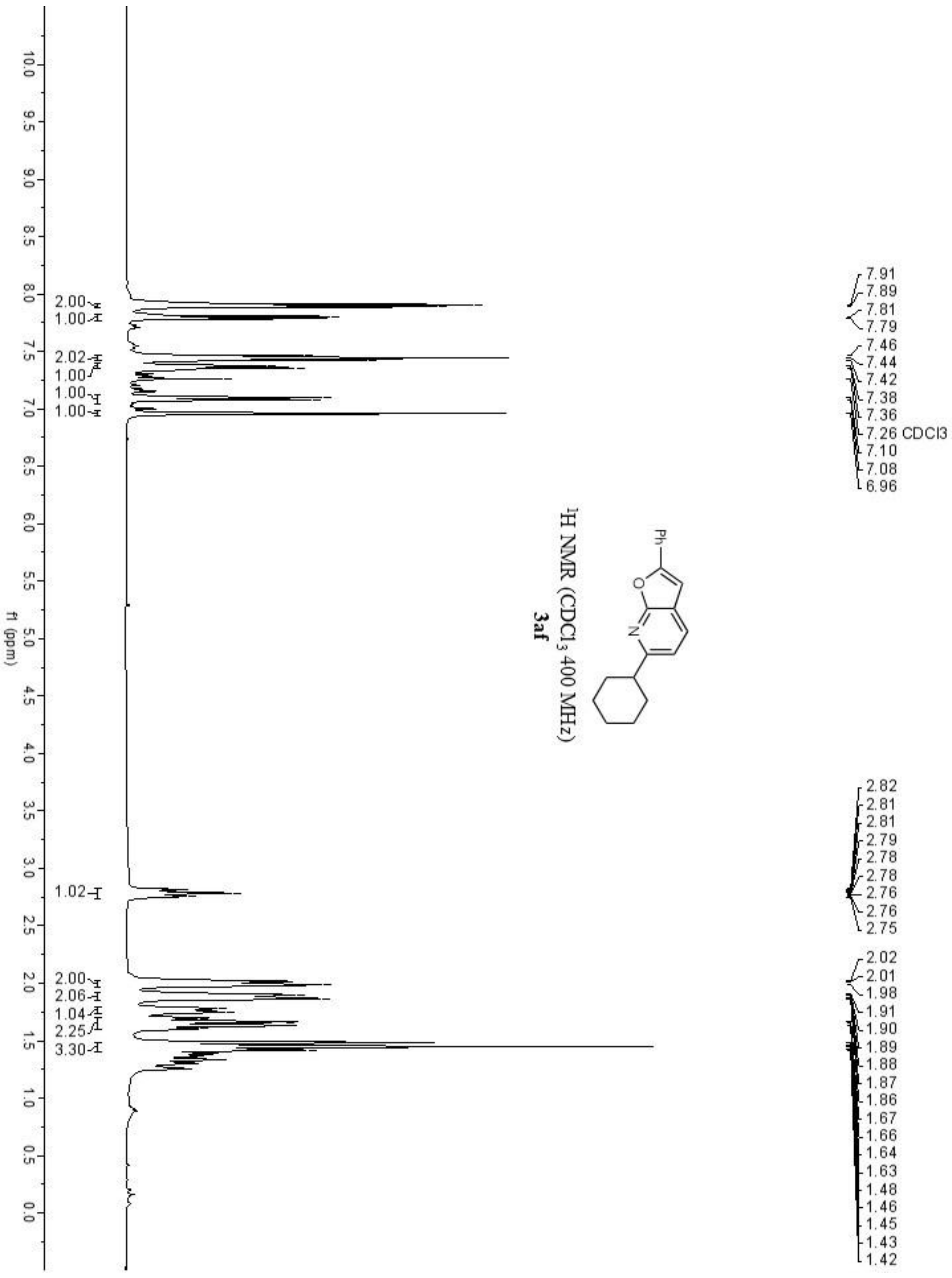
1H NMR (CDCl3 400 MHz)
3af
Ph
O
N
7.91
7.89
7.81
7.79
7.46
7.44
7.42
7.38
7.36
7.26 CDCl3
7.10
7.08
6.96
2.82
2.81
2.81
2.79
2.78
2.78
2.76
2.76
2.75
2.02
2.01
1.98
1.91
1.90
1.89
1.88
1.87
1.86
1.67
1.66
1.64
1.63
1.48
1.46
1.45
1.43
1.42
2.00
1.00
2.02
1.00
1.00
1.00
1.02
2.00
2.06
1.04
2.25
3.30
f1 (ppm)

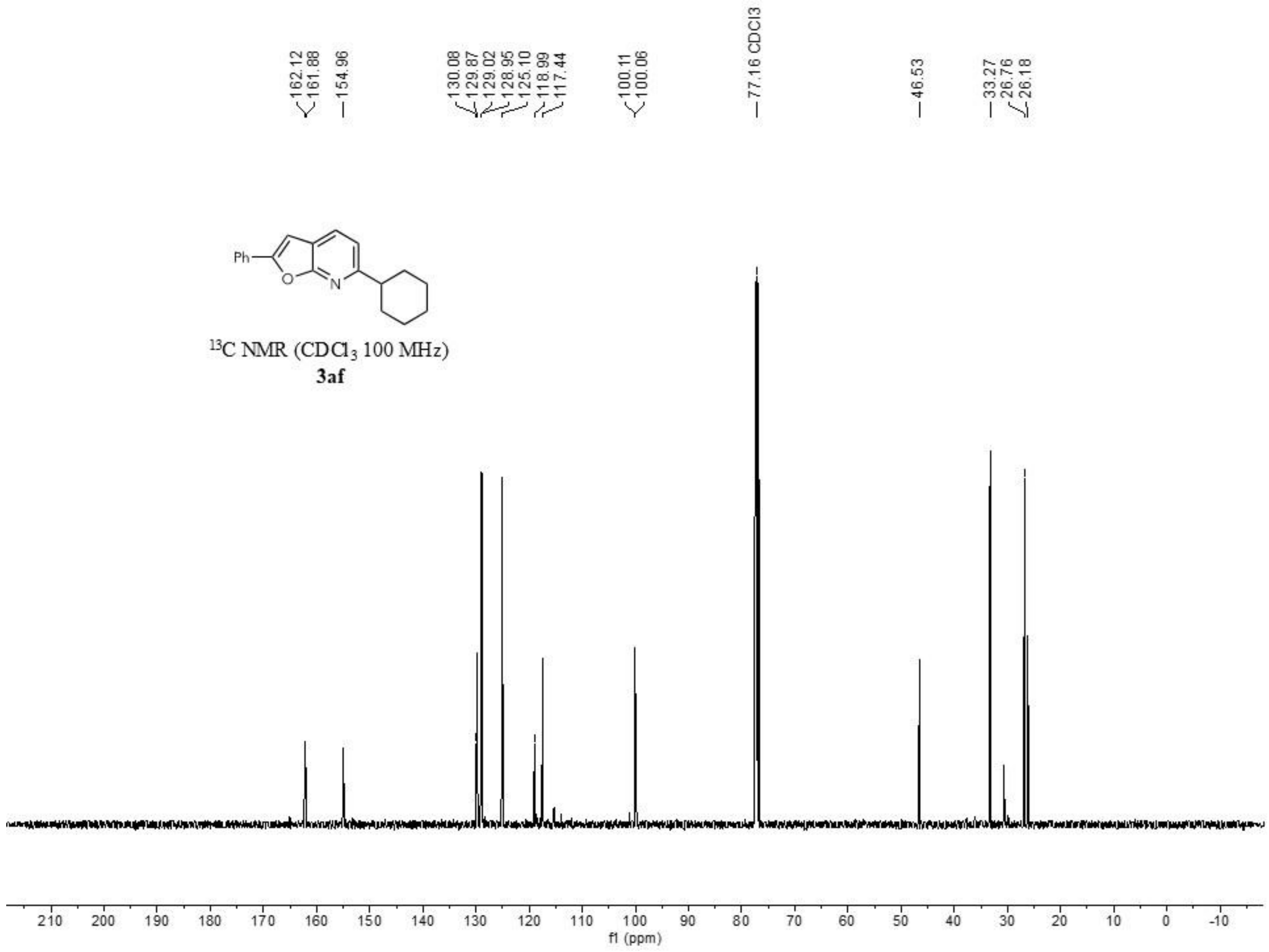
162.12
161.88
154.96
130.08
129.87
129.02
128.95
125.10
118.99
117.44
100.11
100.06
77.16 CDCl3
46.53
33.27
26.76
26.18
Ph
O
N
^{13}C NMR ($CDCl_3$ 100 MHz)
3af
210
200
190
180
170
160
150
140
130
120
110
100
90
80
70
60
50
40
30
20
10
0
-10
f1 (ppm)

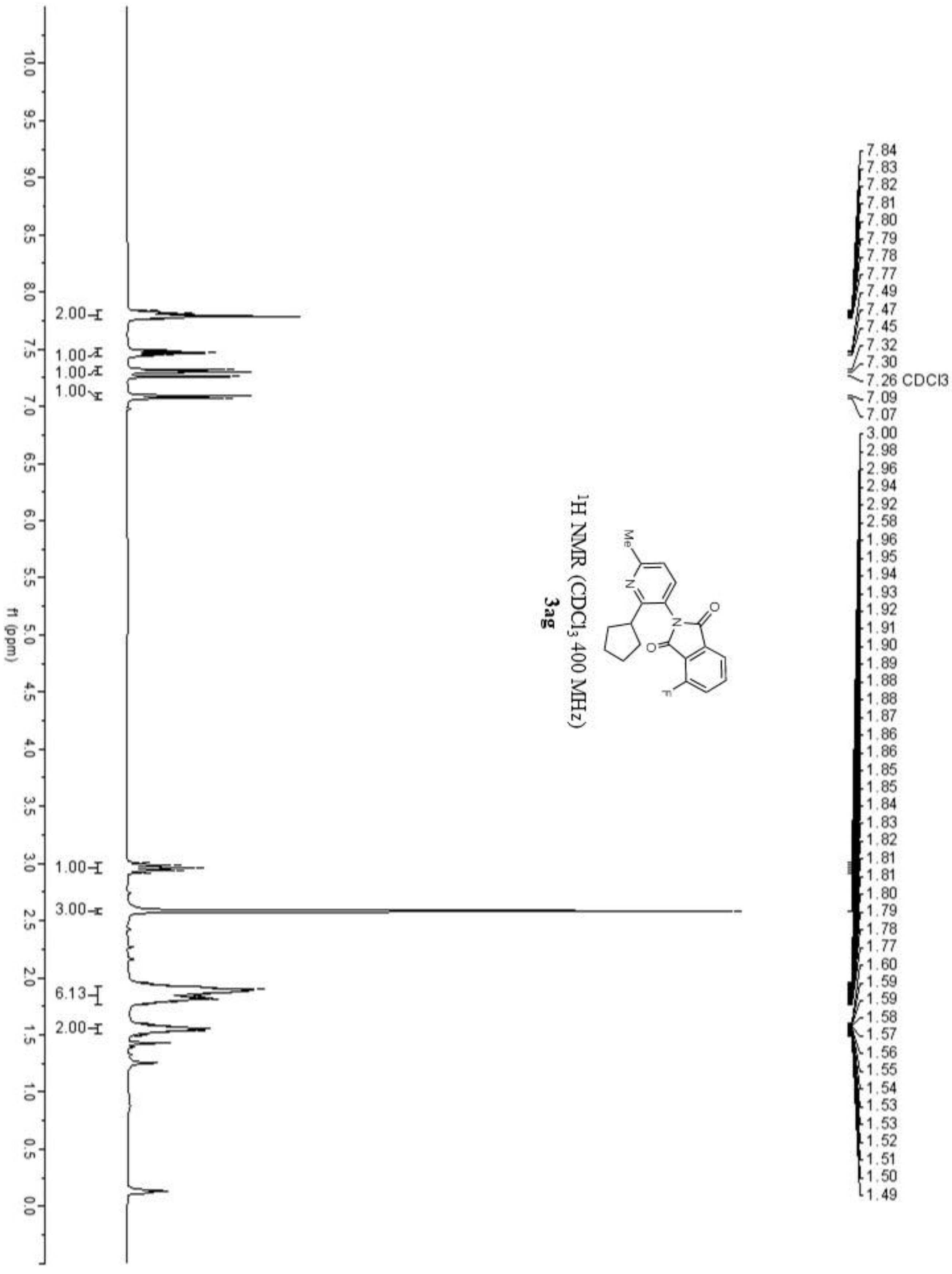

¹H NMR (CDCl3 400 MHz)
3ag
7.84
7.83
7.82
7.81
7.80
7.79
7.78
7.77
7.49
7.47
7.45
7.32
7.30
7.26 CDCl3
7.09
7.07
3.00
2.98
2.96
2.94
2.92
2.58
1.96
1.95
1.94
1.93
1.92
1.91
1.90
1.89
1.88
1.88
1.87
1.86
1.86
1.85
1.85
1.84
1.83
1.82
1.81
1.81
1.80
1.79
1.78
1.77
1.60
1.59
1.59
1.58
1.57
1.56
1.55
1.54
1.53
1.53
1.52
1.51
1.50
1.49
2.00
1.00
1.00
1.00
1.00
3.00
6.13
2.00
f1 (ppm)

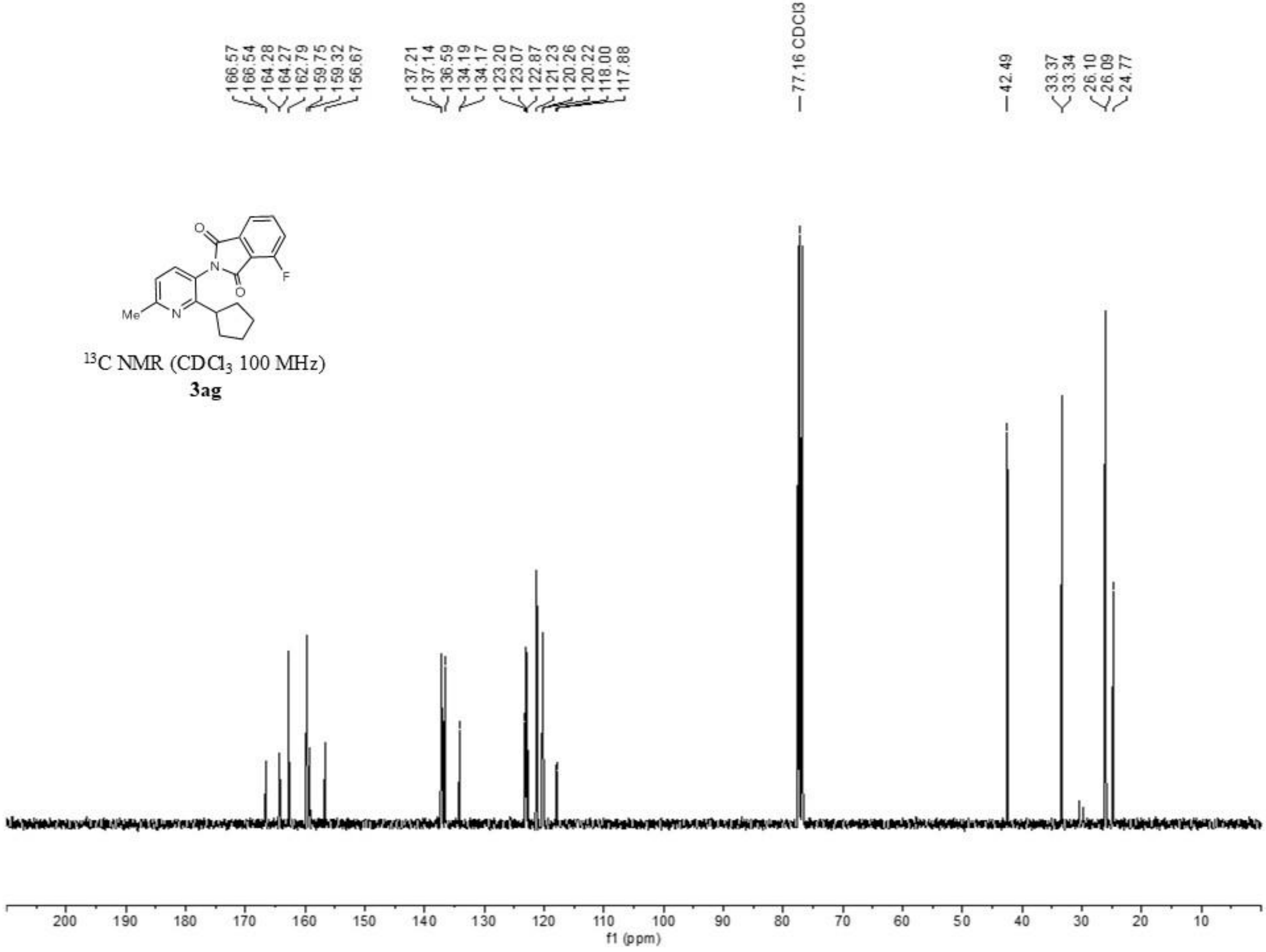

^{13}C NMR ($CDCl_3$ 100 MHz)

3ag

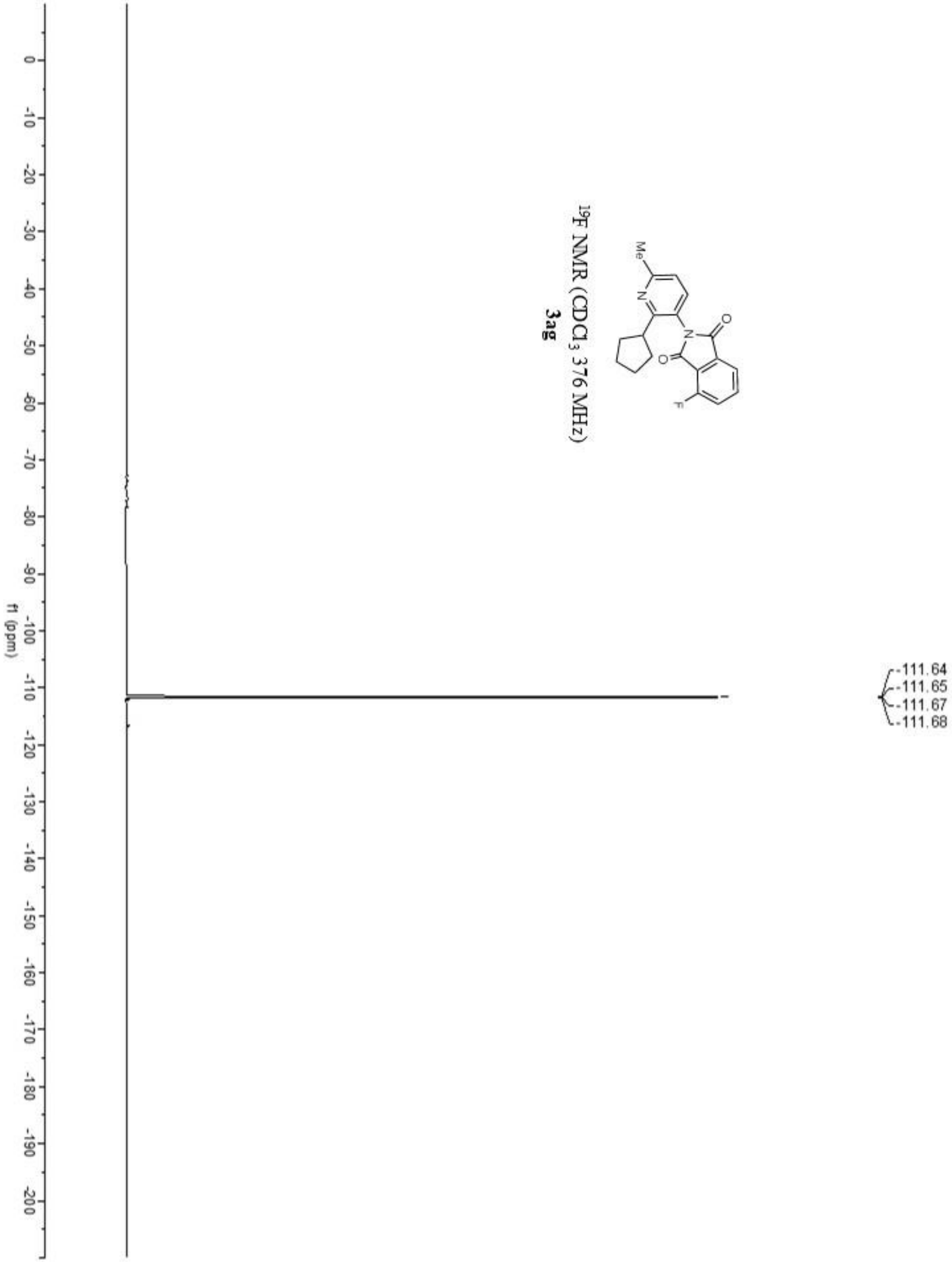

^{19}F NMR (CDCl$_3$ 376 MHz)
3ag
Me
N
O
F
-111.64
-111.65
-111.67
-111.68
f1 (ppm)

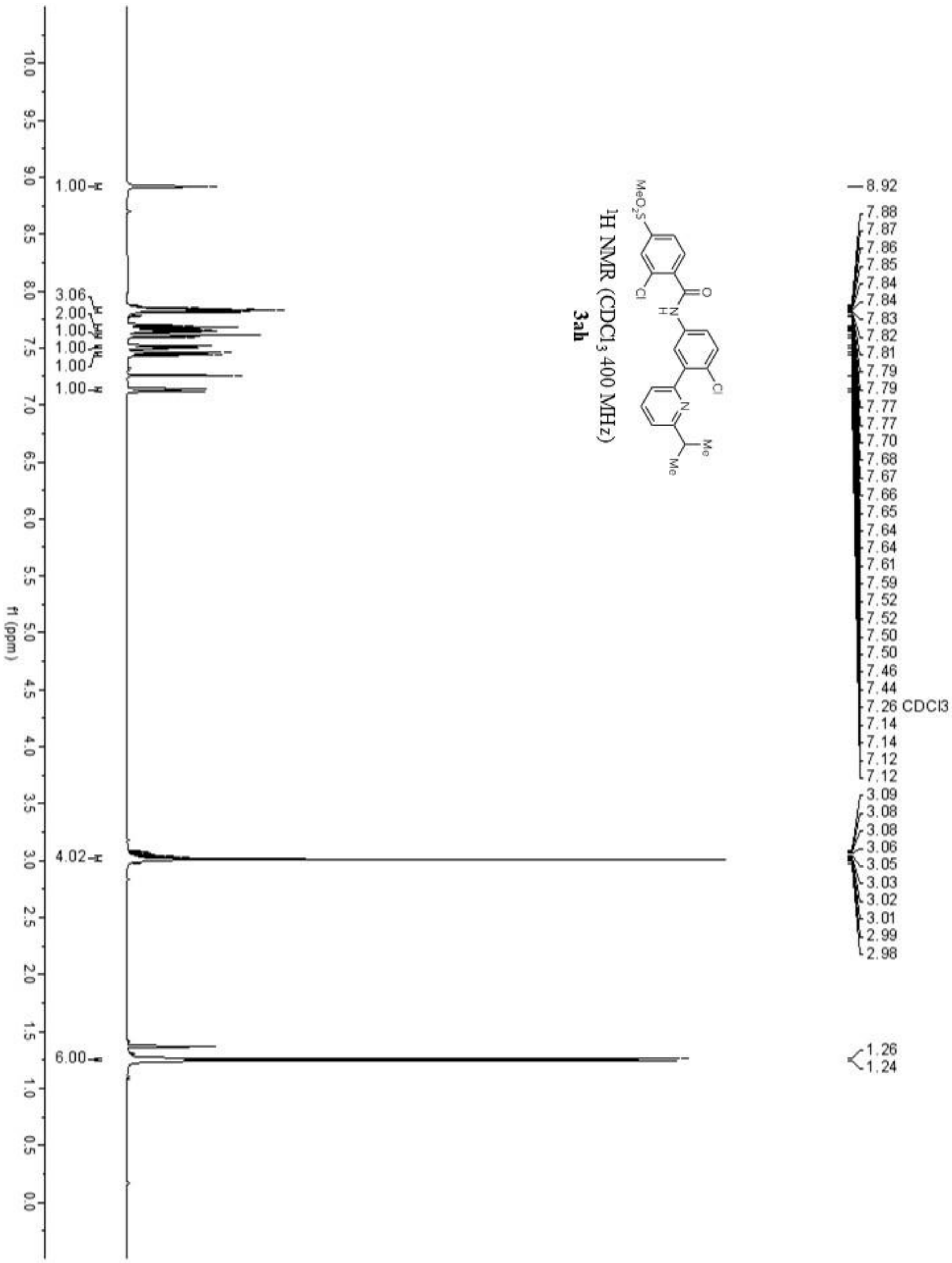
1H NMR (CDCl3 400 MHz)
3ah
MeO2S
Cl
O
N
H
Cl
N
Me
Me
8.92
7.88
7.87
7.86
7.85
7.84
7.84
7.83
7.82
7.81
7.79
7.79
7.77
7.77
7.70
7.68
7.67
7.66
7.65
7.64
7.64
7.61
7.59
7.52
7.52
7.50
7.50
7.46
7.44
7.26 CDCl3
7.14
7.14
7.12
7.12
3.09
3.08
3.08
3.06
3.05
3.03
3.02
3.01
2.99
2.98
1.26
1.24
1.00
3.06
2.00
1.00
1.00
1.00
1.00
4.02
6.00
10.0
9.5
9.0
8.5
8.0
7.5
7.0
6.5
6.0
5.5
5.0
4.5
4.0
3.5
3.0
2.5
2.0
1.5
1.0
0.5
0.0
f1 (ppm)

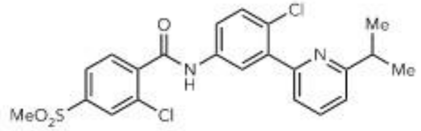

^{13}C NMR ($CDCl_3$ 100 MHz)

3ah

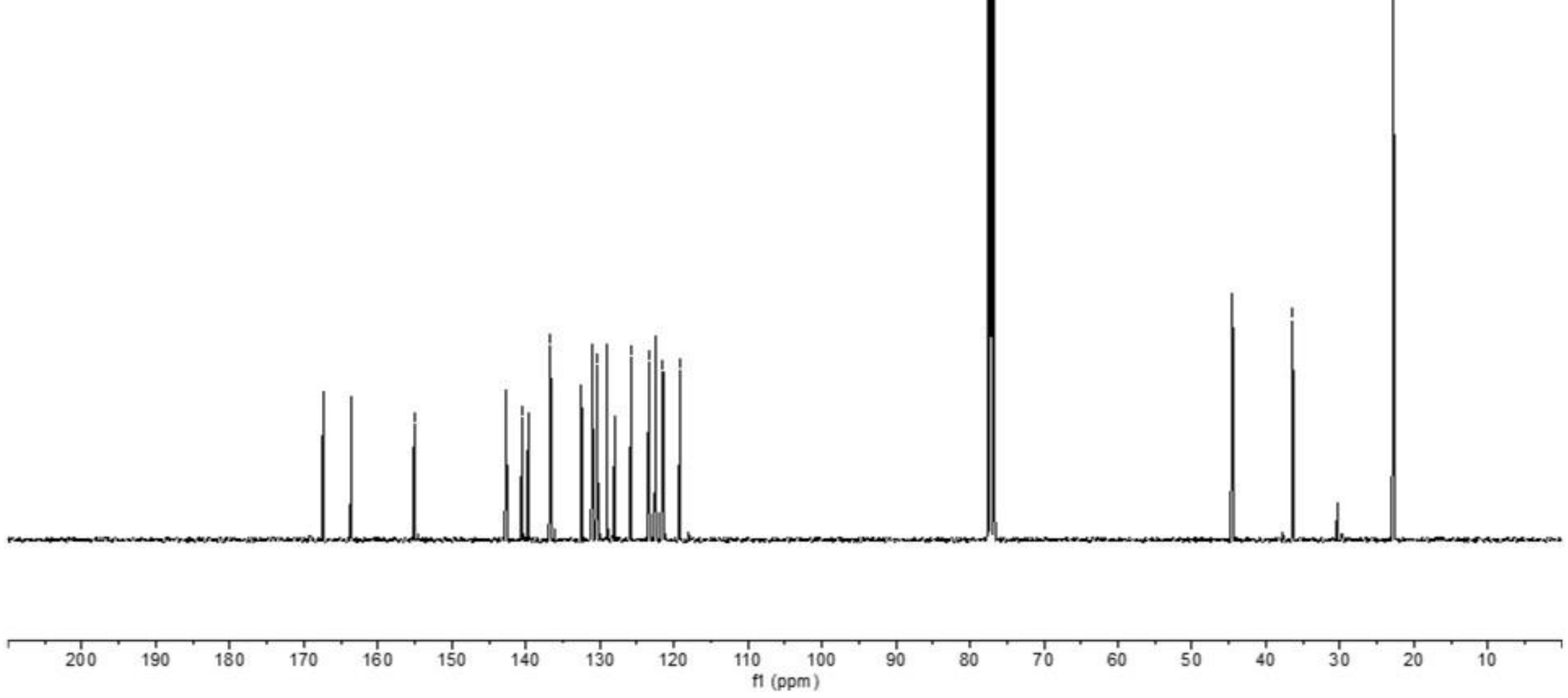

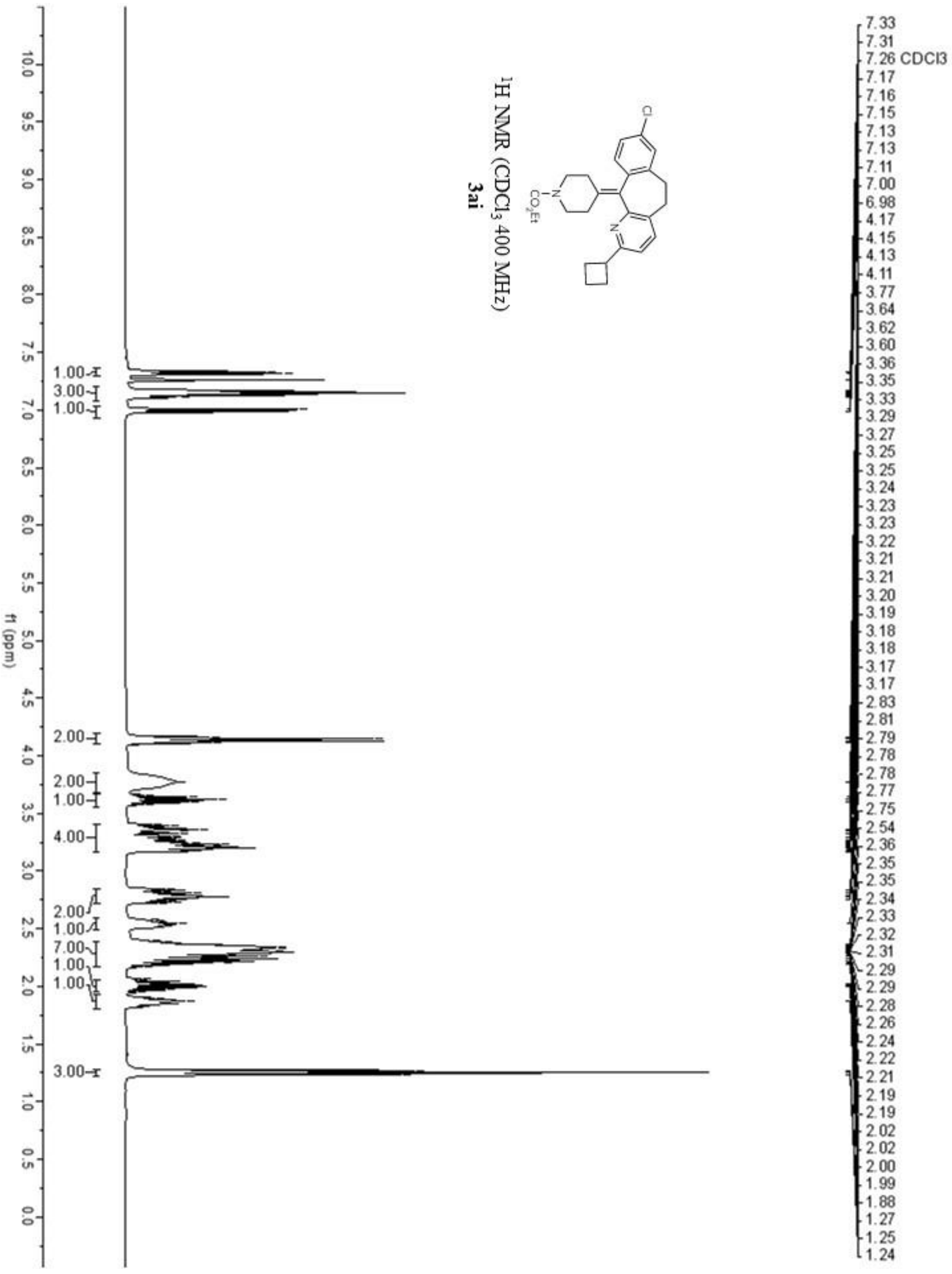

¹H NMR (CDCl₃ 400 MHz)
3ai
Cl
N
N
CO₂Et
7.33
7.31
7.26 CDCl3
7.17
7.16
7.15
7.13
7.13
7.11
7.00
6.98
4.17
4.15
4.13
4.11
3.77
3.64
3.62
3.60
3.36
3.35
3.33
3.29
3.27
3.25
3.25
3.24
3.23
3.23
3.22
3.21
3.21
3.20
3.19
3.18
3.18
3.17
3.17
2.83
2.81
2.79
2.78
2.78
2.77
2.75
2.54
2.36
2.35
2.35
2.34
2.33
2.32
2.31
2.29
2.29
2.28
2.26
2.24
2.22
2.21
2.19
2.19
2.02
2.02
2.00
1.99
1.88
1.27
1.25
1.24
f1 (ppm)

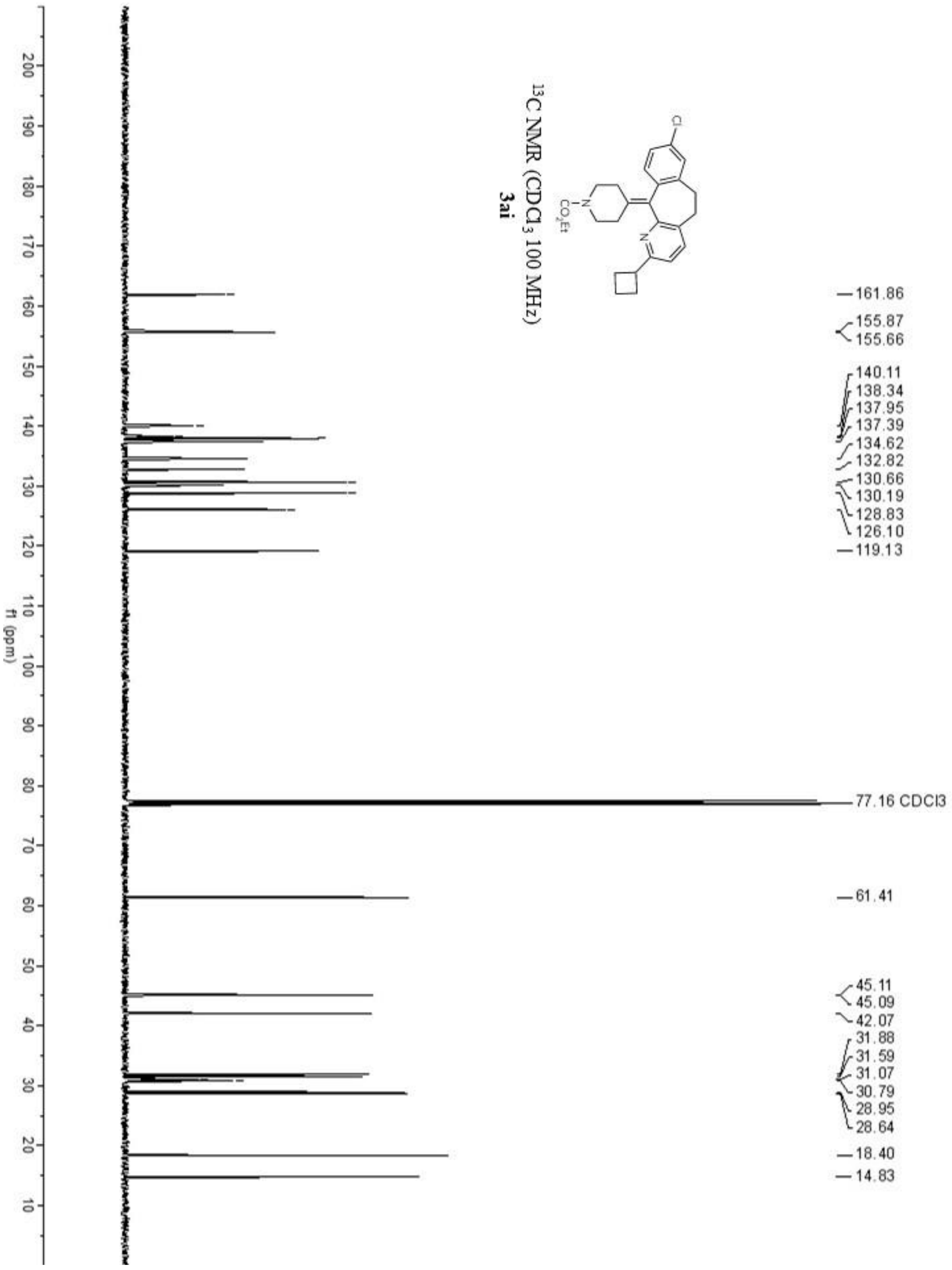
13C NMR (CDCl3 100 MHz)
3ai
Cl
N
N
CO2Et
161.86
155.87
155.66
140.11
138.34
137.95
137.39
134.62
132.82
130.66
130.19
128.83
126.10
119.13
77.16 CDCl3
61.41
45.11
45.09
42.07
31.88
31.59
31.07
30.79
28.95
28.64
18.40
14.83
f1 (ppm)

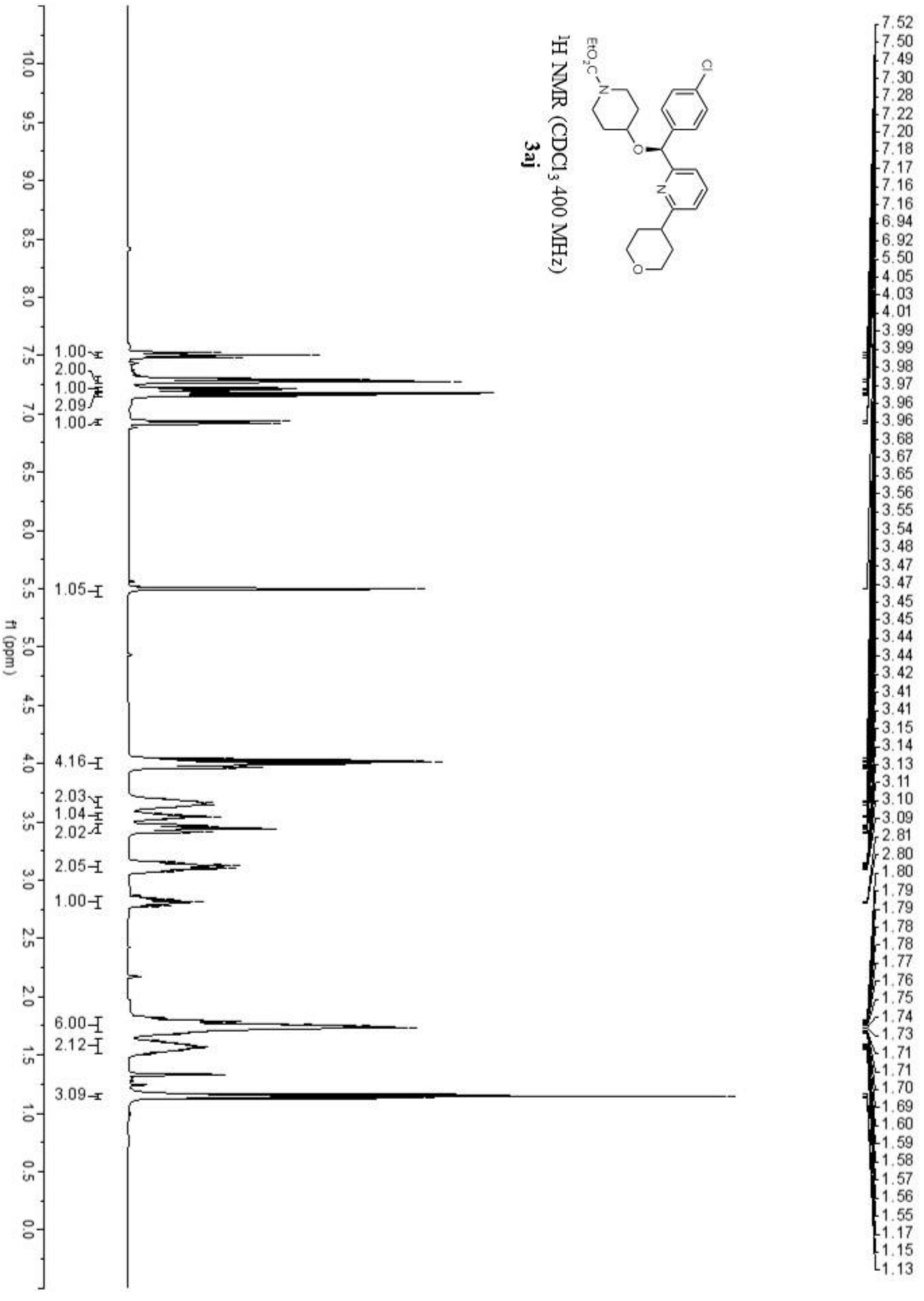

1H NMR (CDCl3 400 MHz)
3aj
EtO2C
Cl
N
O
7.52 7.50 7.49 7.30 7.28 7.22 7.20 7.18 7.17 7.16 7.16 6.94 6.92 5.50 4.05 4.03 4.01 3.99 3.99 3.98 3.97 3.96 3.96 3.68 3.67 3.65 3.56 3.55 3.54 3.48 3.47 3.47 3.45 3.45 3.44 3.44 3.42 3.41 3.41 3.15 3.14 3.13 3.11 3.10 3.09 2.81 2.80 1.80 1.79 1.79 1.78 1.78 1.77 1.76 1.75 1.74 1.73 1.71 1.71 1.70 1.69 1.60 1.59 1.58 1.57 1.56 1.55 1.17 1.15 1.13
1.00 2.00 1.00 2.09 1.00 1.05 4.16 2.03 1.04 2.02 2.05 1.00 6.00 2.12 3.09
f1 (ppm)
10.0 9.5 9.0 8.5 8.0 7.5 7.0 6.5 6.0 5.5 5.0 4.5 4.0 3.5 3.0 2.5 2.0 1.5 1.0 0.5 0.0

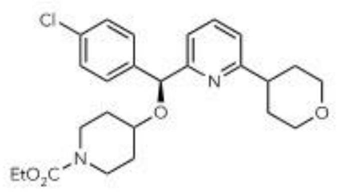

^{13}C NMR ($CDCl_3$ 100 MHz)
3aj

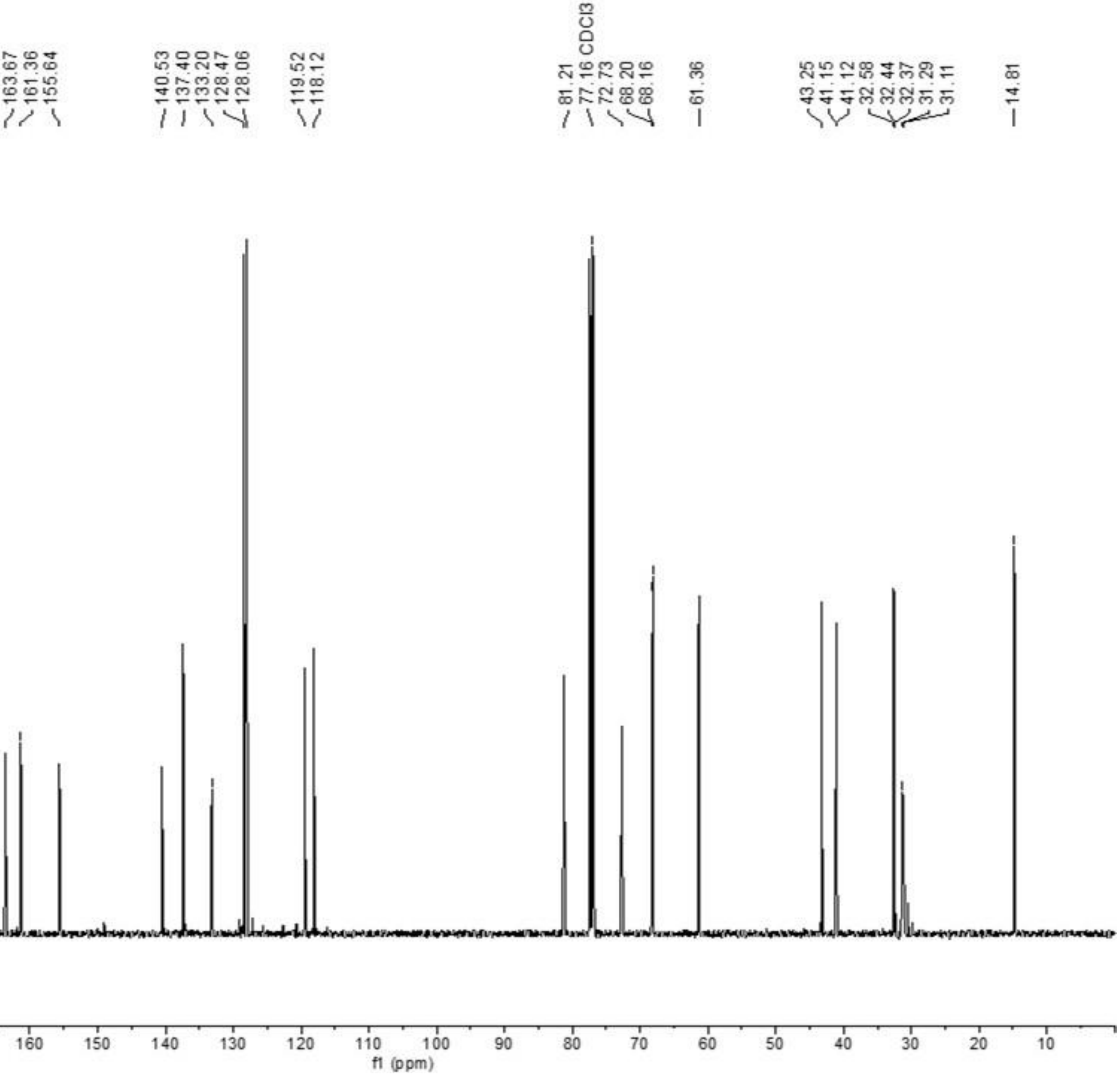

Crude ^1H NMR ($CDCl_3$ 400 MHz)

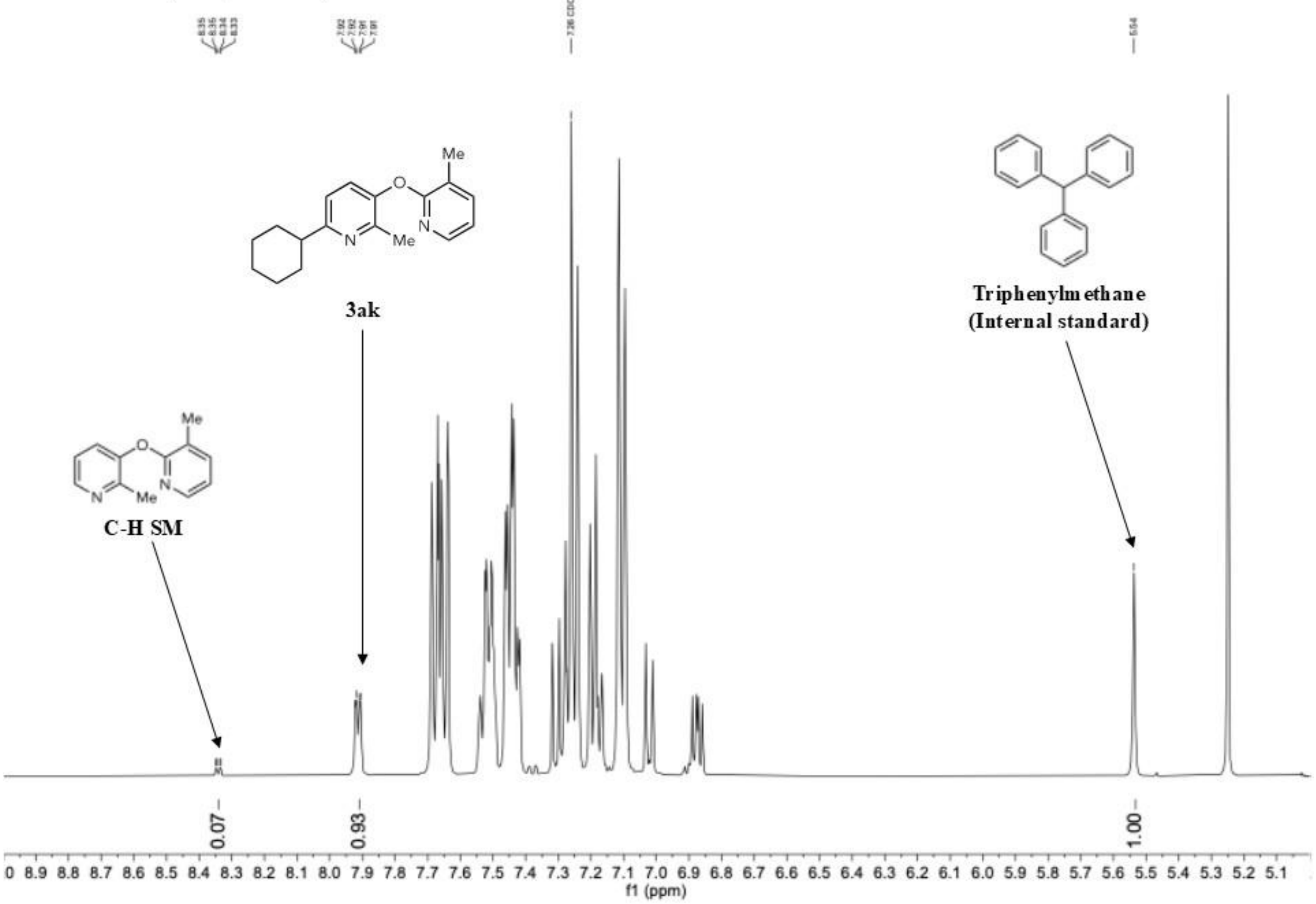

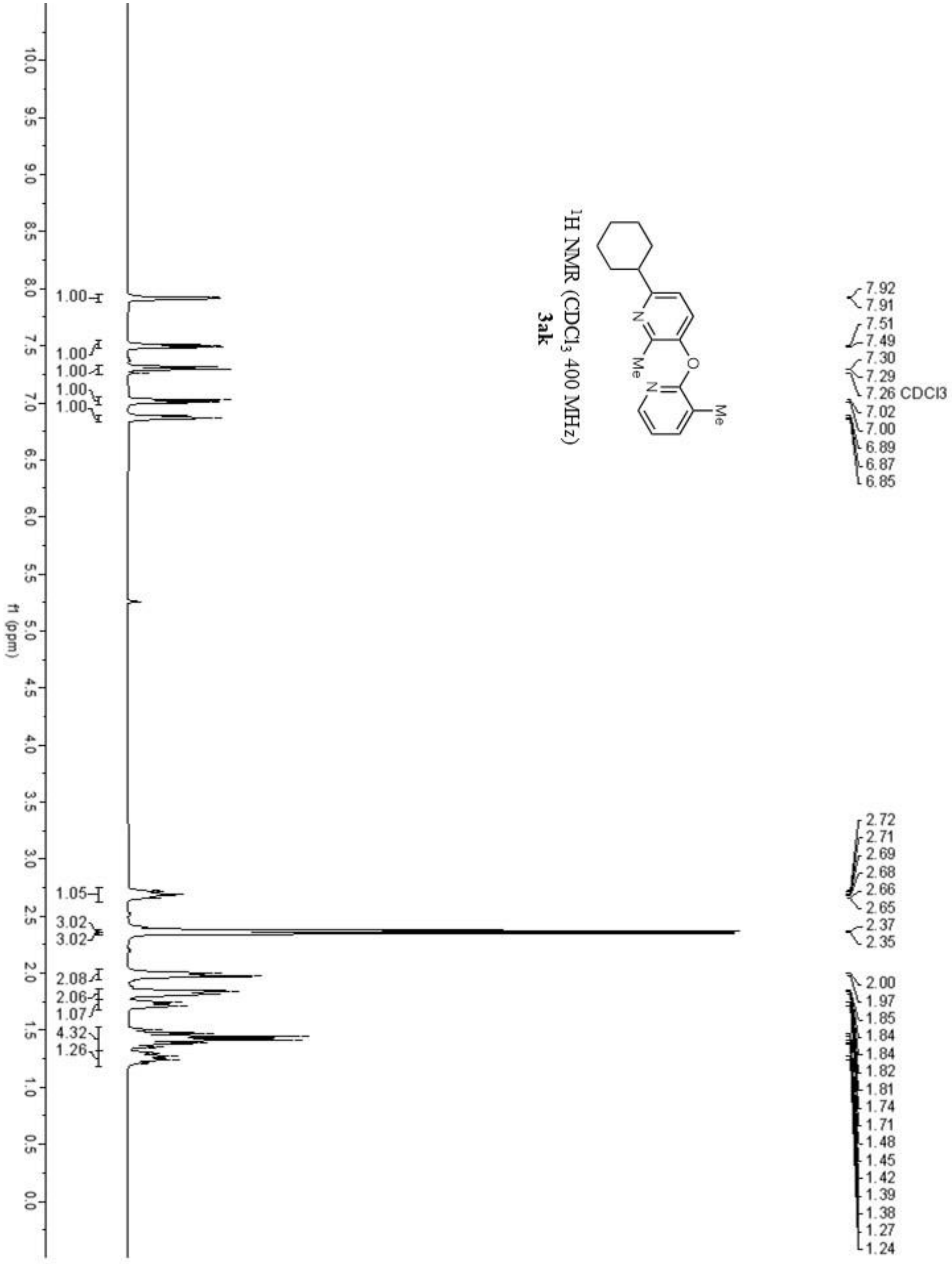

^{1}H NMR (CDCl$_3$ 400 MHz)
3ak
Me
O
N
N
Me
7.92
7.91
7.51
7.49
7.30
7.29
7.26 CDCl3
7.02
7.00
6.89
6.87
6.85
2.72
2.71
2.69
2.68
2.66
2.65
2.37
2.35
2.00
1.97
1.85
1.84
1.84
1.82
1.81
1.74
1.71
1.48
1.45
1.42
1.39
1.38
1.27
1.24
1.00
1.00
1.00
1.00
1.00
1.05
3.02
3.02
2.08
2.06
1.07
4.32
1.26
f1 (ppm)

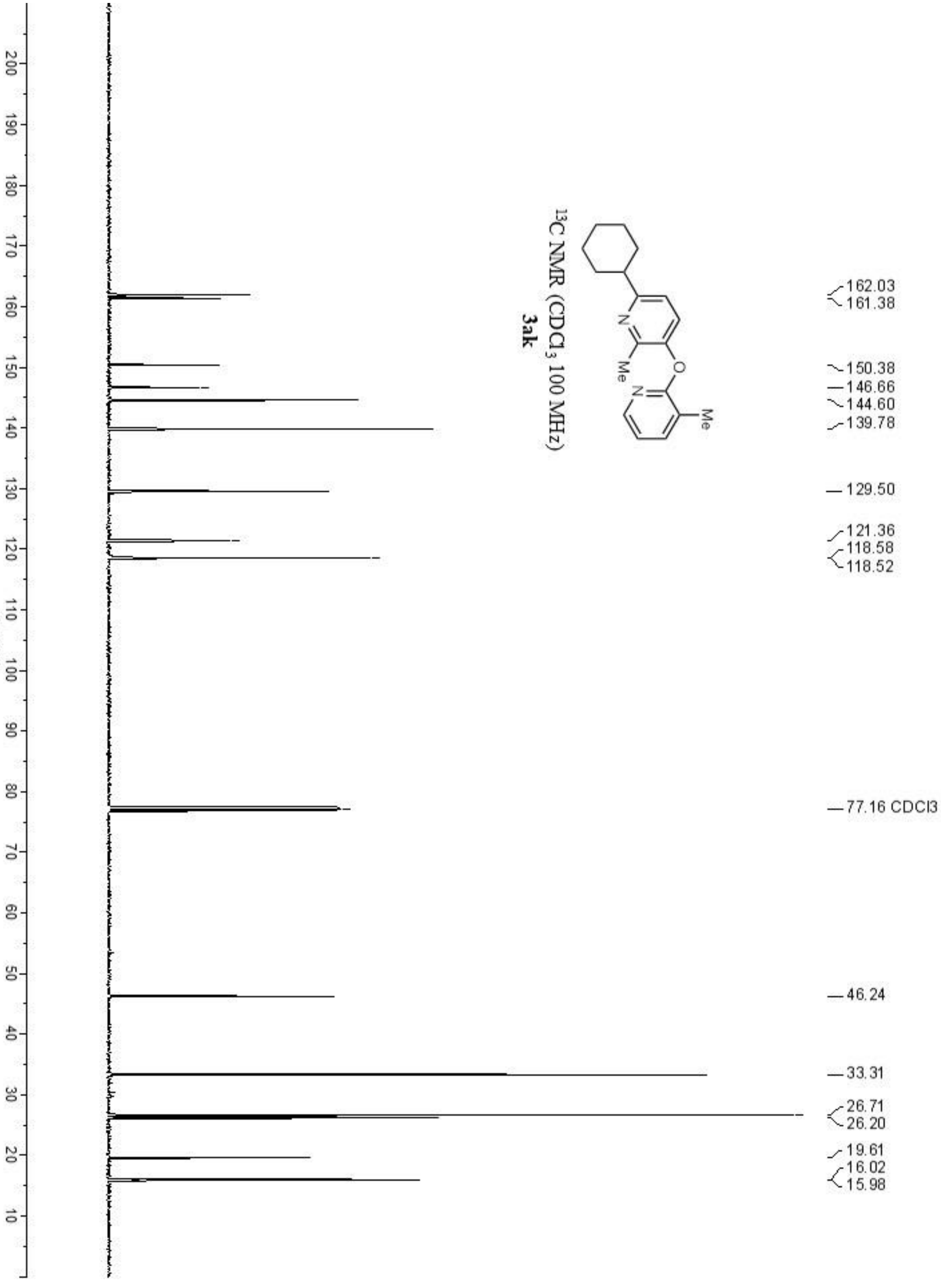

13C NMR (CDCl3 100 MHz)
3ak
Me
N
O
N
Me
162.03
161.38
150.38
146.66
144.60
139.78
129.50
121.36
118.58
118.52
77.16 CDCl3
46.24
33.31
26.71
26.20
19.61
16.02
15.98
200
190
180
170
160
150
140
130
120
110
100
90
80
70
60
50
40
30
20
10
f1 (ppm)

Crude ^{1}H NMR ($CDCl_3$ 400 MHz)

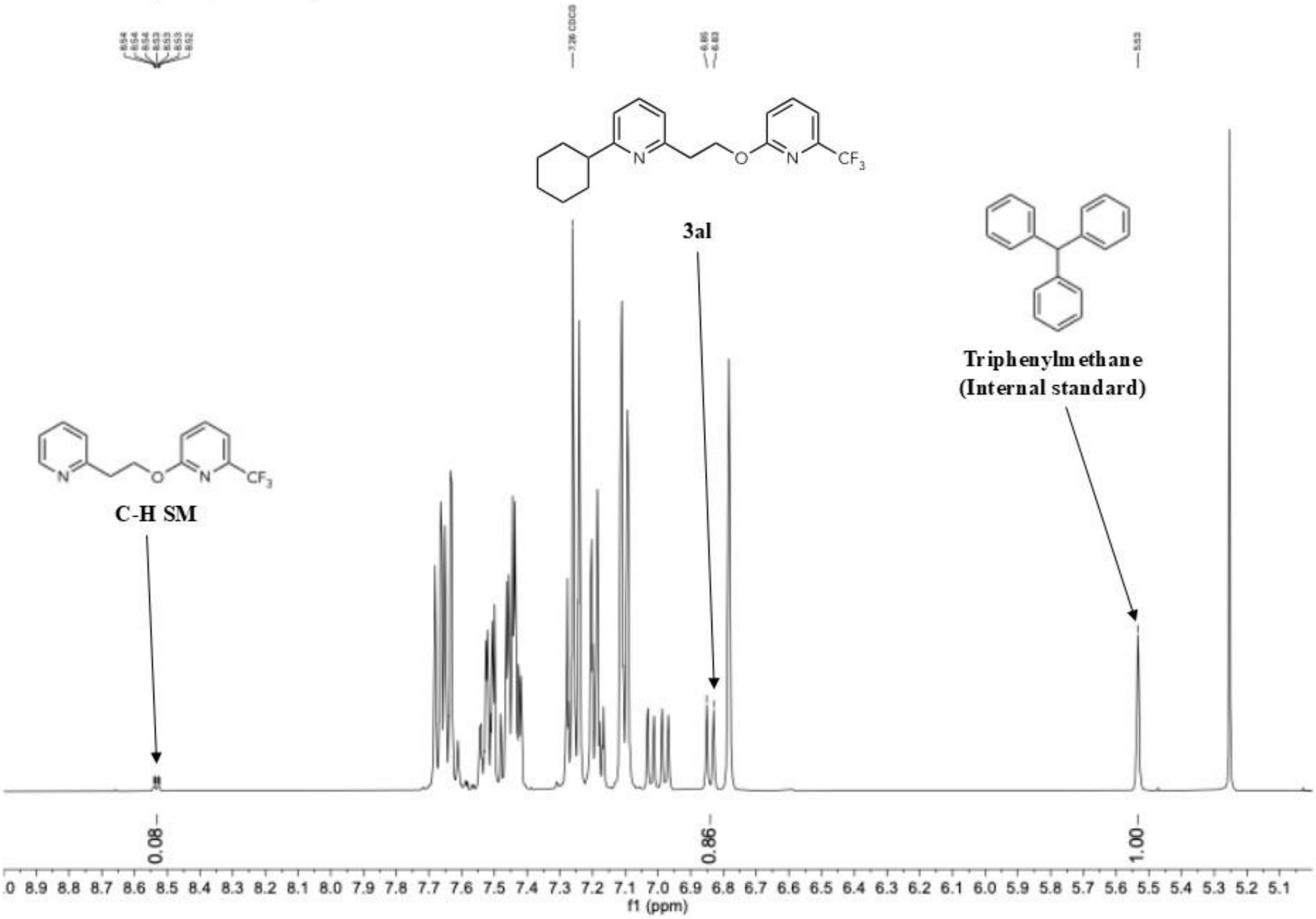

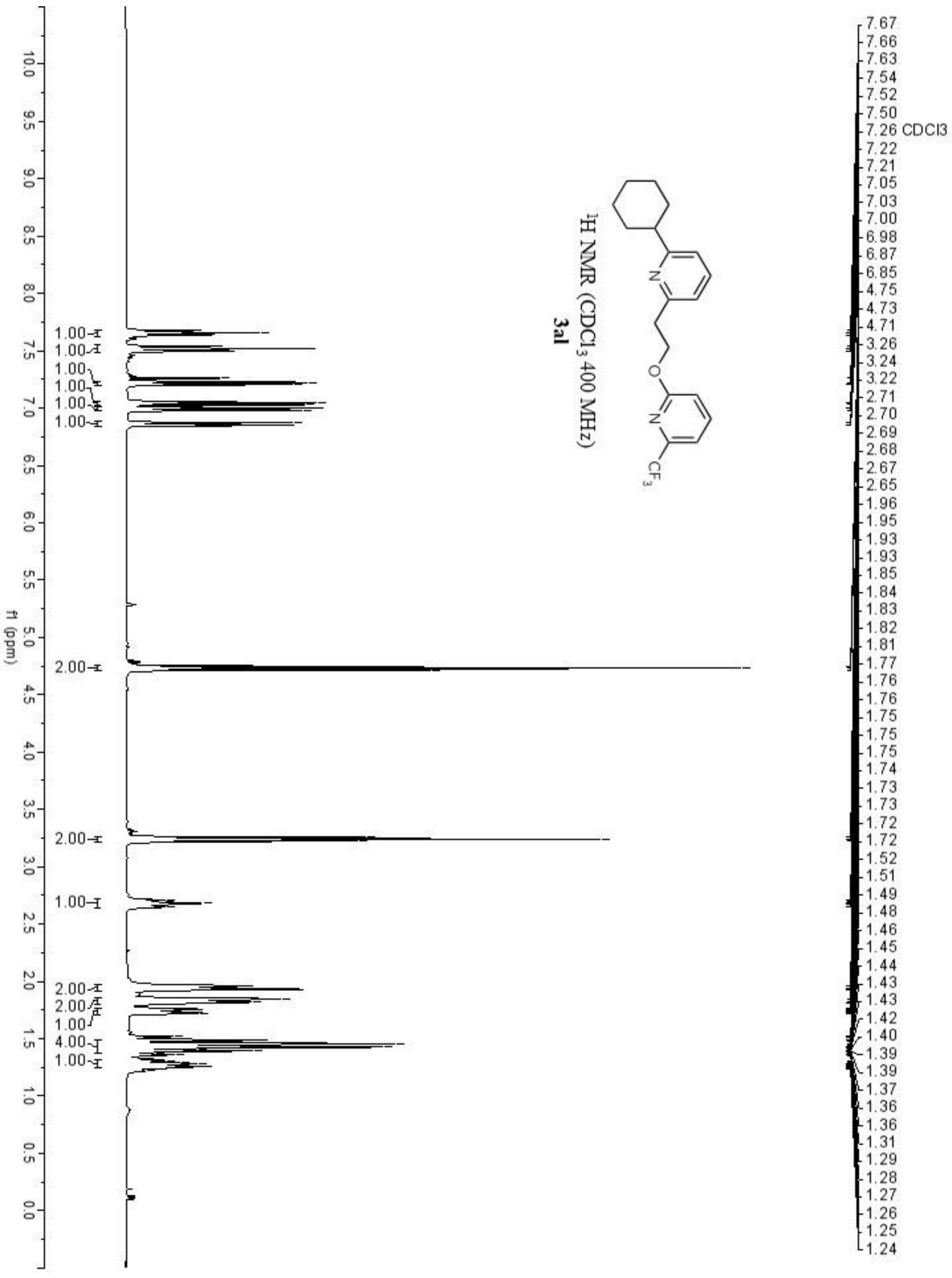
1H NMR (CDCl3 400 MHz)
3al
CDCl3
f1 (ppm)

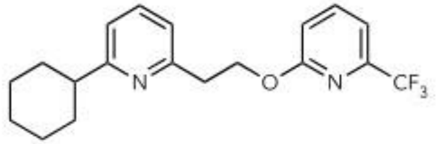

^{13}C NMR ($CDCl_3$ 100 MHz)

3al

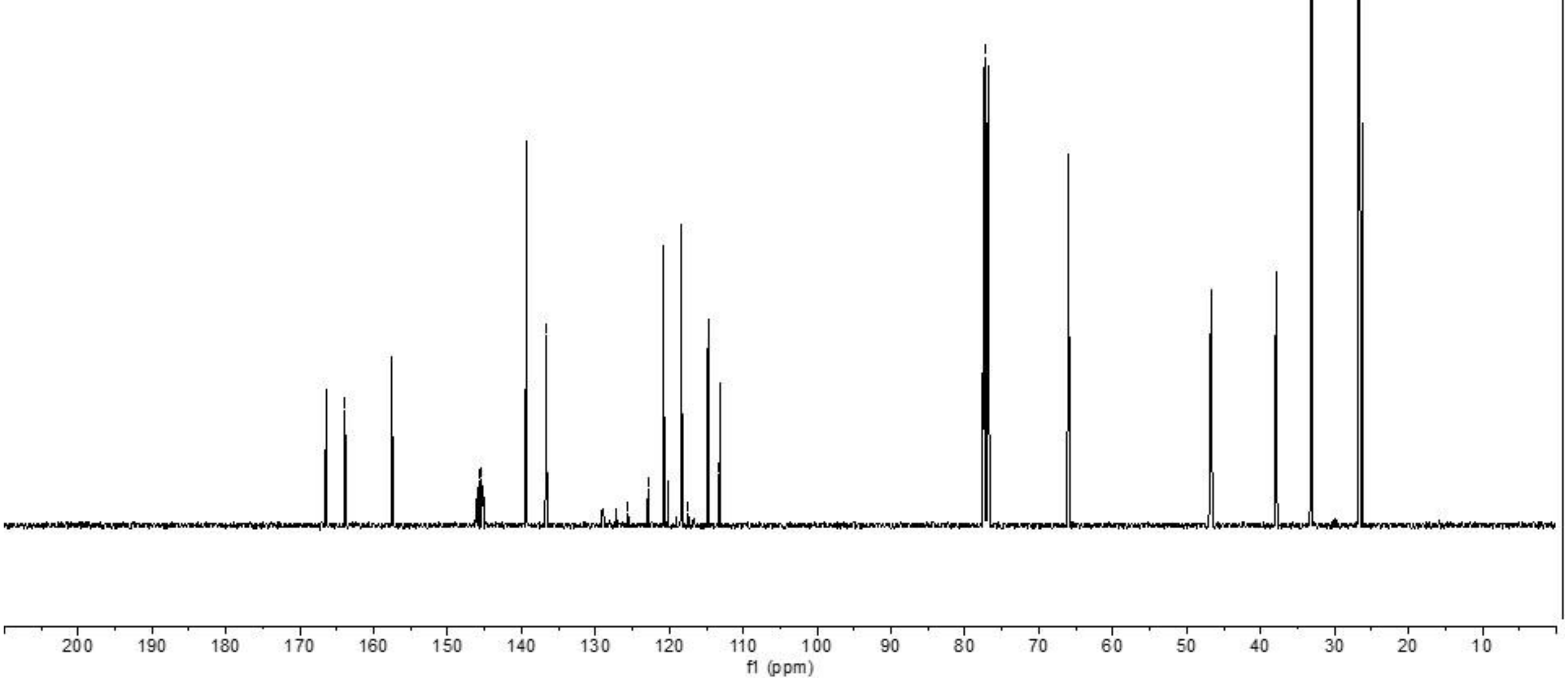

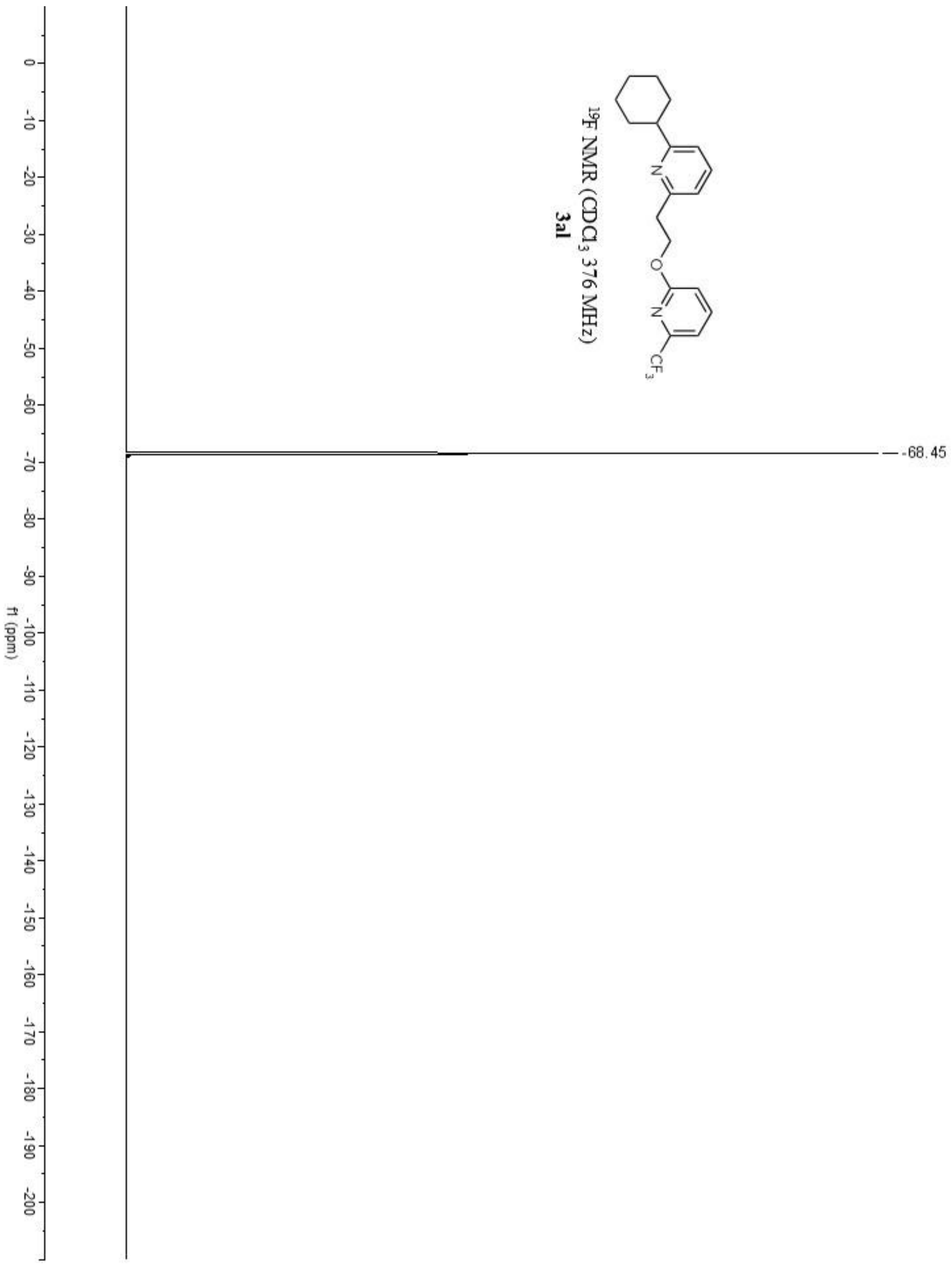

-68.45
^{19}F NMR ($CDCl_3$ 376 MHz)
3al
N
O
N
CF_3
0
-10
-20
-30
-40
-50
-60
-70
-80
-90
-100
-110
-120
-130
-140
-150
-160
-170
-180
-190
-200
f1 (ppm)

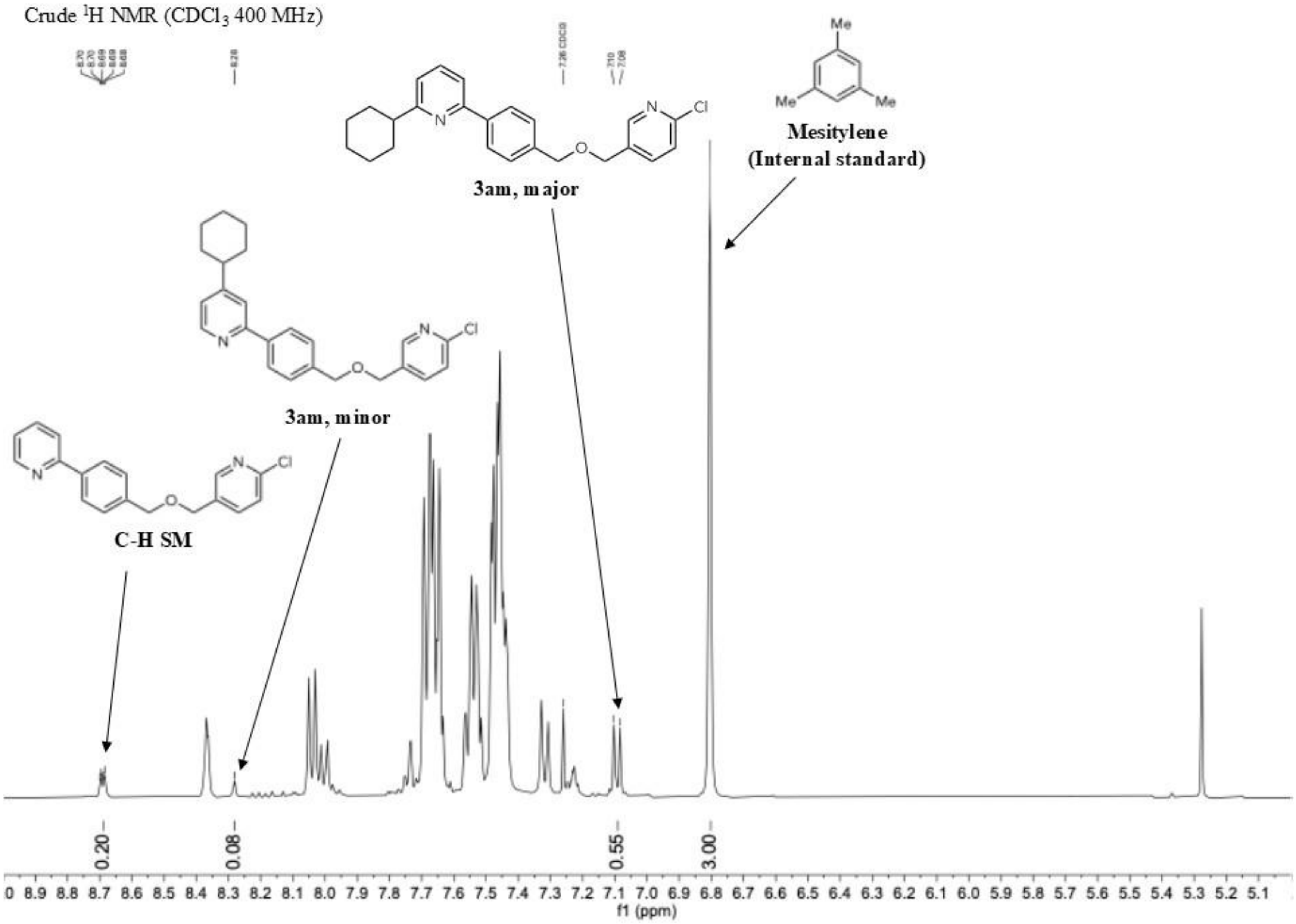

Crude ^{1}H NMR ($CDCl_3$ 400 MHz)
8.70
8.70
8.69
8.69
8.68
8.28
7.26 CDCl3
7.10
7.08
Mesitylene
(Internal standard)
3am, major
3am, minor
C-H SM
0.20
0.08
0.55
3.00
0 8.9 8.8 8.7 8.6 8.5 8.4 8.3 8.2 8.1 8.0 7.9 7.8 7.7 7.6 7.5 7.4 7.3 7.2 7.1 7.0 6.9 6.8 6.7 6.6 6.5 6.4 6.3 6.2 6.1 6.0 5.9 5.8 5.7 5.6 5.5 5.4 5.3 5.2 5.1
f1 (ppm)

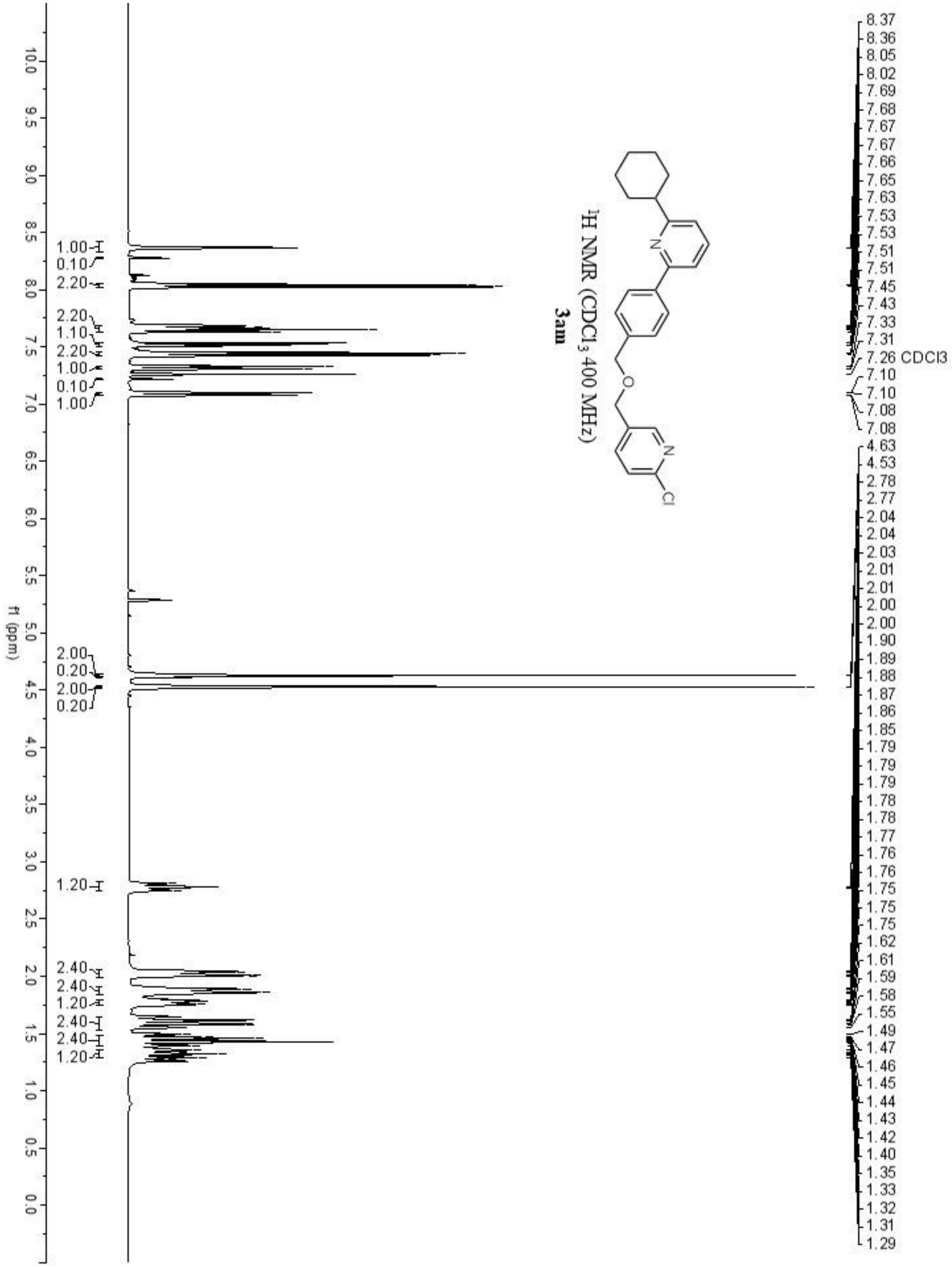

1H NMR (CDCl3 400 MHz)
3am
N
N
O
Cl
8.37
8.36
8.05
8.02
7.69
7.68
7.67
7.67
7.66
7.65
7.63
7.53
7.53
7.51
7.51
7.45
7.43
7.33
7.31
7.26 CDCl3
7.10
7.10
7.08
7.08
4.63
4.53
2.78
2.77
2.04
2.04
2.03
2.01
2.01
2.00
2.00
1.90
1.89
1.88
1.87
1.86
1.85
1.79
1.79
1.79
1.78
1.78
1.77
1.76
1.76
1.75
1.75
1.75
1.62
1.61
1.59
1.58
1.55
1.49
1.47
1.46
1.45
1.44
1.43
1.42
1.40
1.35
1.33
1.32
1.31
1.29
1.00
0.10
2.20
2.20
1.10
2.20
1.00
0.10
1.00
2.00
0.20
2.00
0.20
1.20
2.40
2.40
1.20
2.40
2.40
1.20
f1 (ppm)

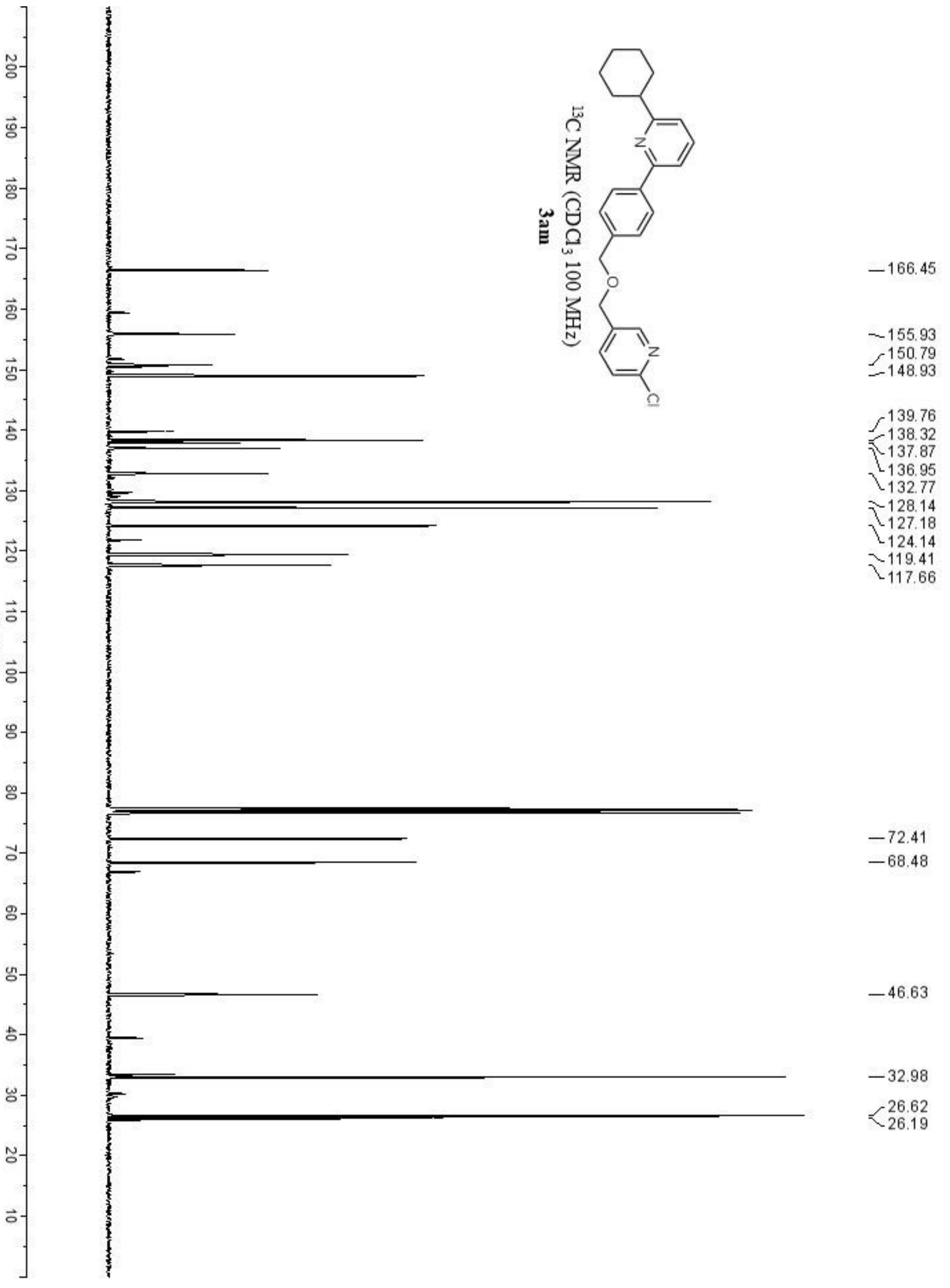
13C NMR (CDCl3 100 MHz)
3am
N
N
O
Cl
166.45
155.93
150.79
148.93
139.76
138.32
137.87
136.95
132.77
128.14
127.18
124.14
119.41
117.66
72.41
68.48
46.63
32.98
26.62
26.19
200
190
180
170
160
150
140
130
120
110
100
90
80
70
60
50
40
30
20
10
f1 (ppm)

Crude ^{1}H NMR ($CDCl_3$ 400 MHz)

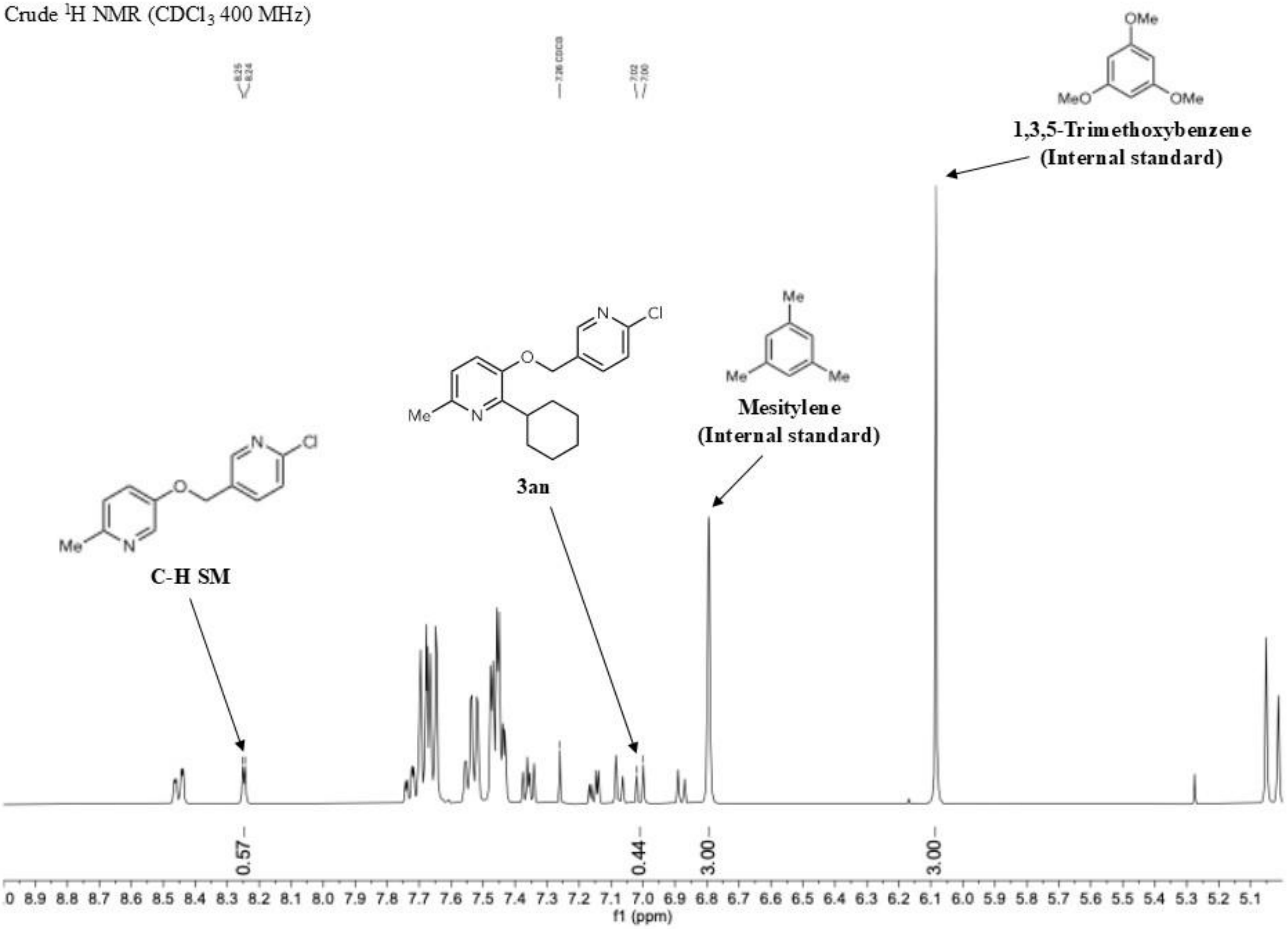

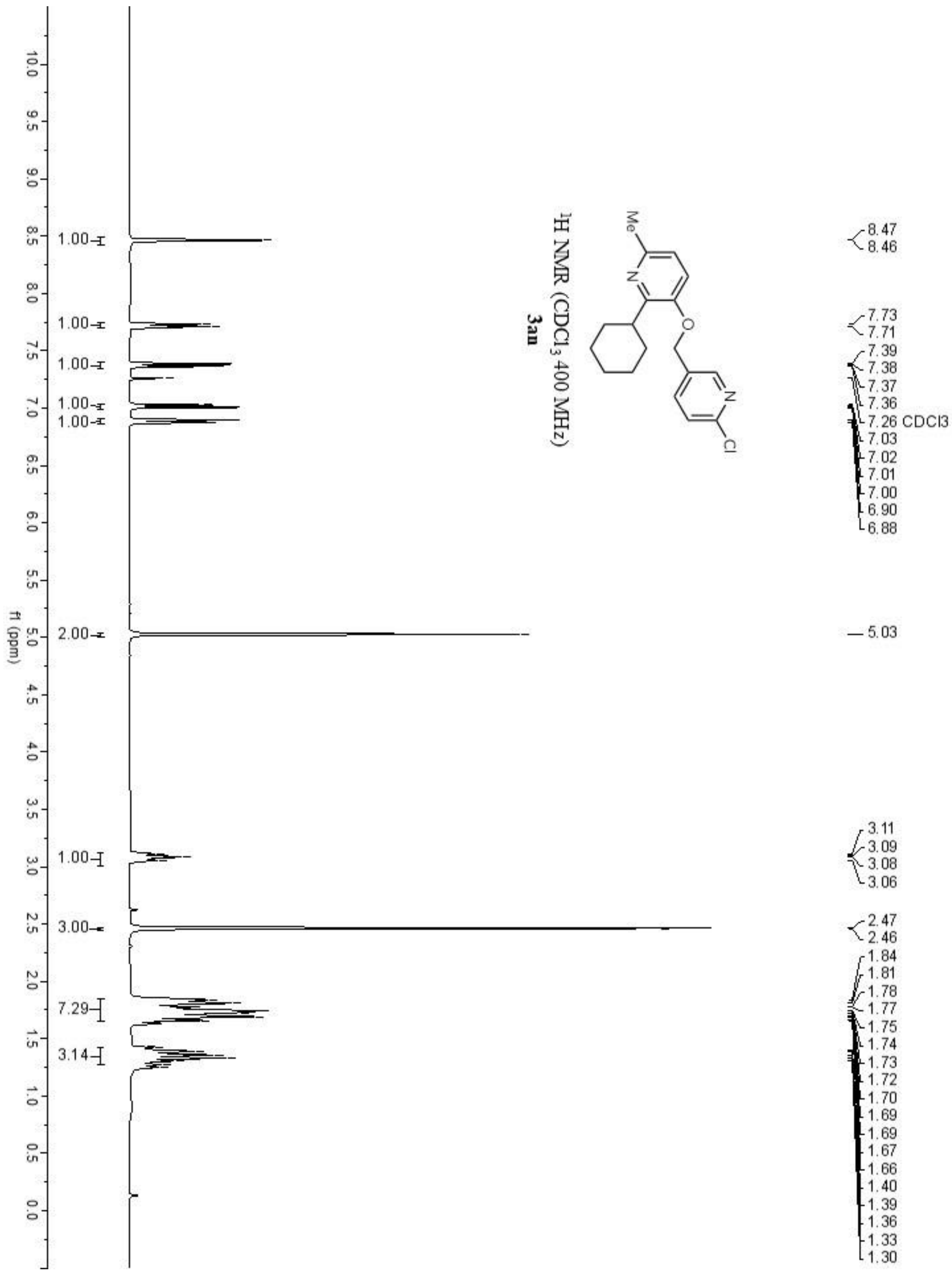
¹H NMR (CDCl3 400 MHz)
3an
Me
N
O
N
Cl
8.47
8.46
7.73
7.71
7.39
7.38
7.37
7.36
7.26 CDCl3
7.03
7.02
7.01
7.00
6.90
6.88
5.03
3.11
3.09
3.08
3.06
2.47
2.46
1.84
1.81
1.78
1.77
1.75
1.74
1.73
1.72
1.70
1.69
1.69
1.67
1.66
1.40
1.39
1.36
1.33
1.30
1.00
1.00
1.00
1.00
1.00
2.00
1.00
3.00
7.29
3.14
f1 (ppm)
10.0
9.5
9.0
8.5
8.0
7.5
7.0
6.5
6.0
5.5
5.0
4.5
4.0
3.5
3.0
2.5
2.0
1.5
1.0
0.5
0.0

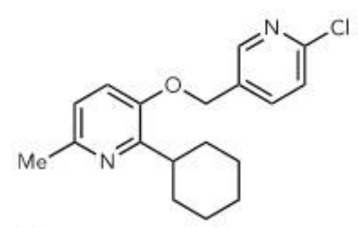

^{13}C NMR ($CDCl_3$ 100 MHz)
3an

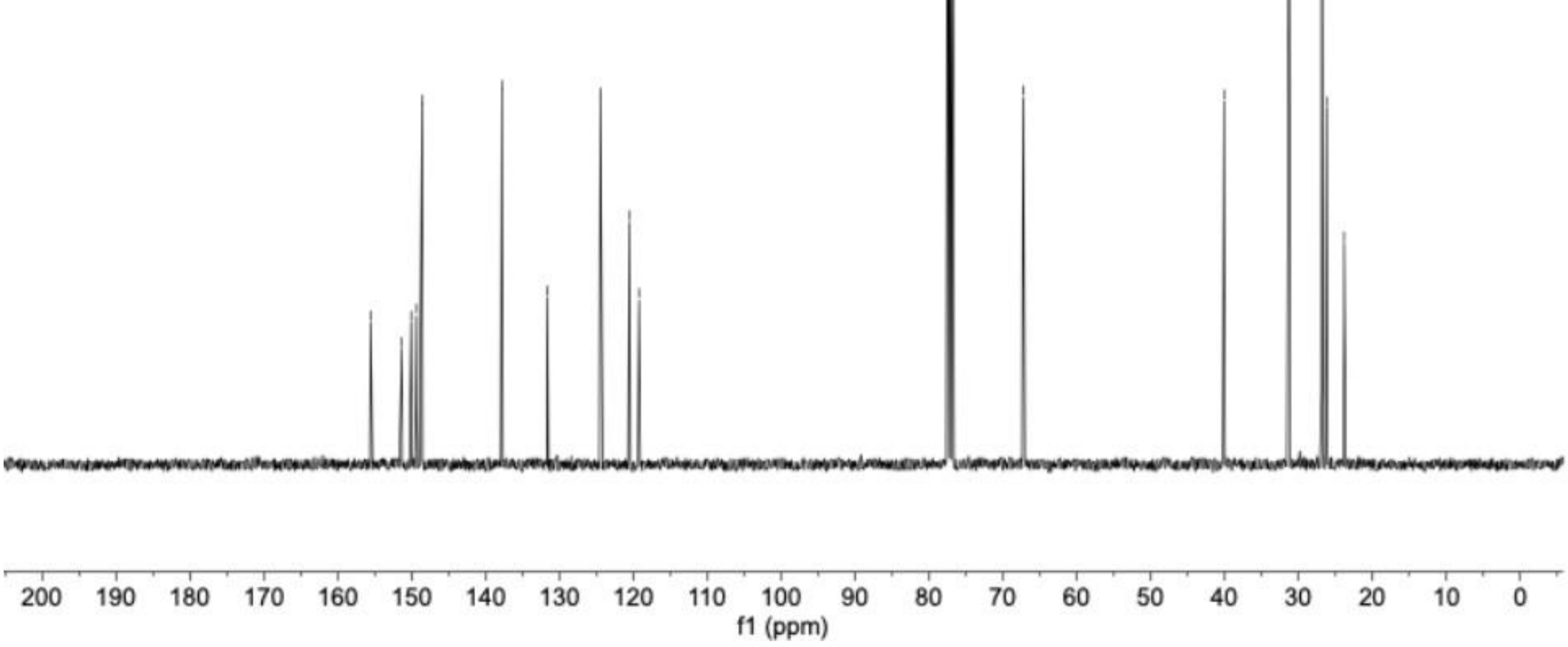

Crude ^{1}H NMR ($CDCl_3$ 400 MHz)

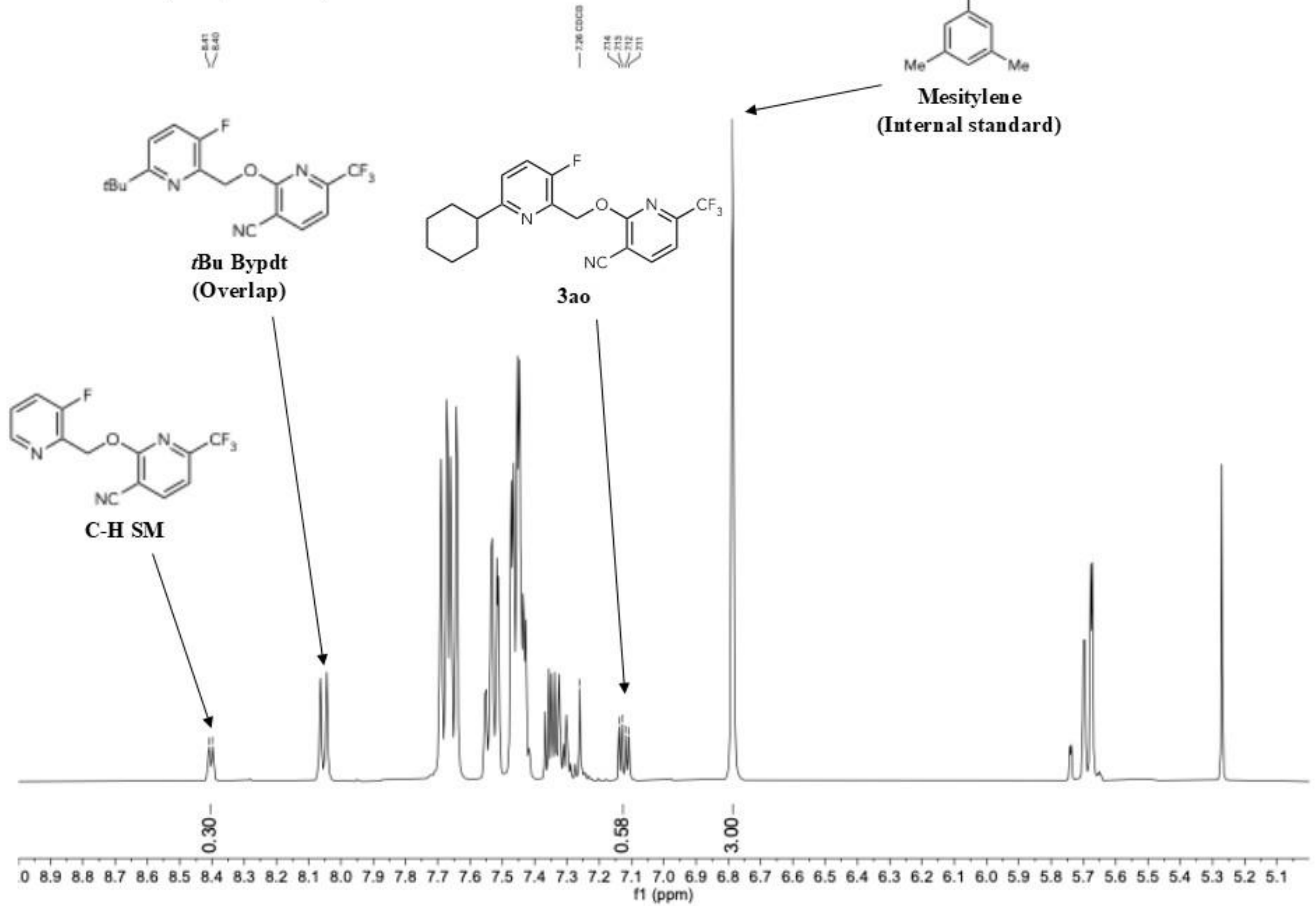

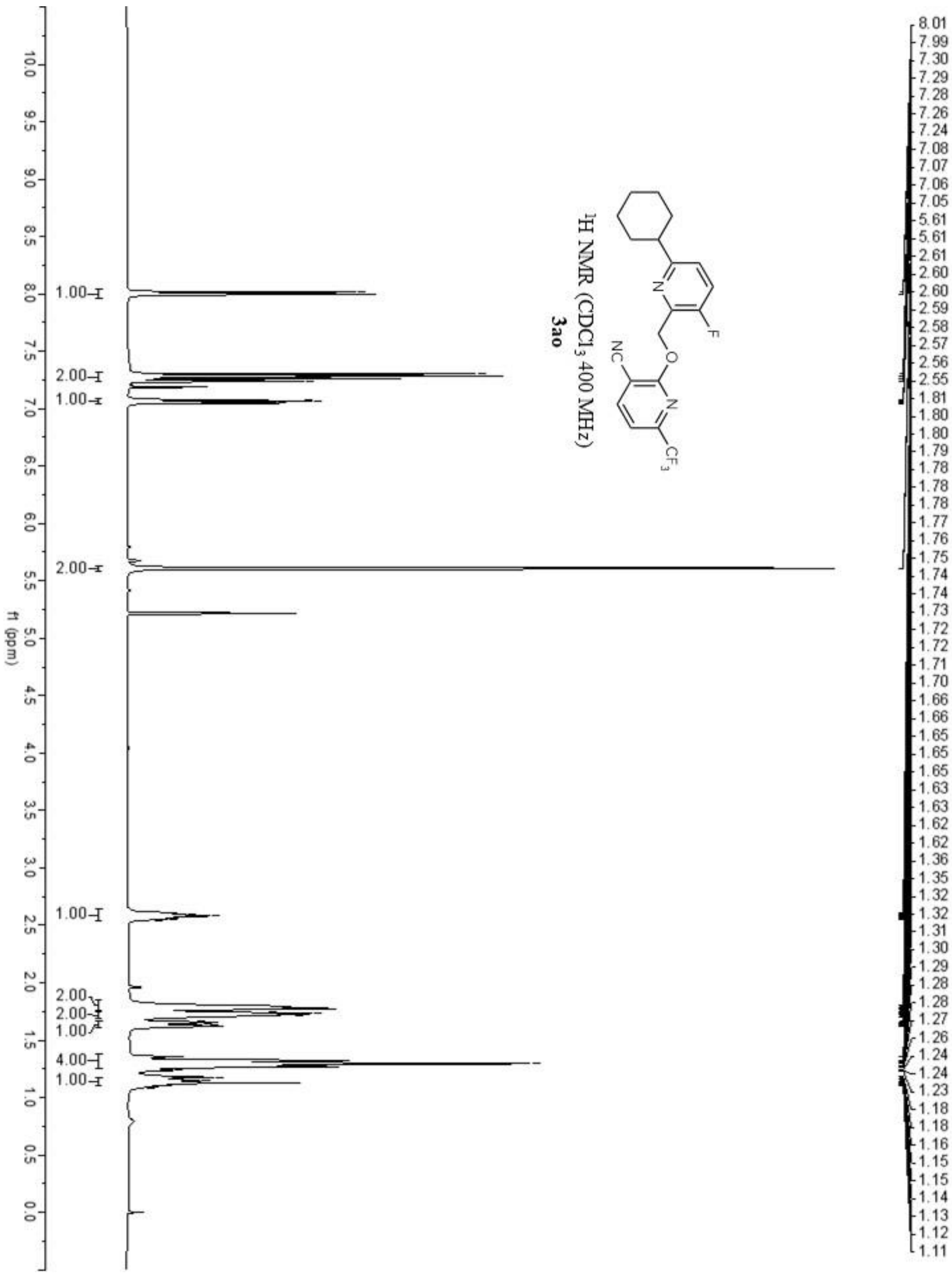

1H NMR (CDCl3 400 MHz)
3ao
NC
O
N
CF3
F
8.01
7.99
7.30
7.29
7.28
7.26
7.24
7.08
7.07
7.06
7.05
5.61
5.61
2.61
2.60
2.60
2.59
2.58
2.57
2.56
2.55
1.81
1.80
1.80
1.79
1.78
1.78
1.78
1.77
1.76
1.75
1.74
1.74
1.73
1.72
1.72
1.71
1.70
1.66
1.66
1.65
1.65
1.65
1.63
1.63
1.62
1.62
1.36
1.35
1.32
1.32
1.31
1.30
1.29
1.28
1.28
1.27
1.26
1.24
1.24
1.23
1.18
1.18
1.16
1.15
1.15
1.14
1.13
1.12
1.11
1.00
2.00
1.00
2.00
1.00
2.00
2.00
1.00
4.00
1.00
f1 (ppm)

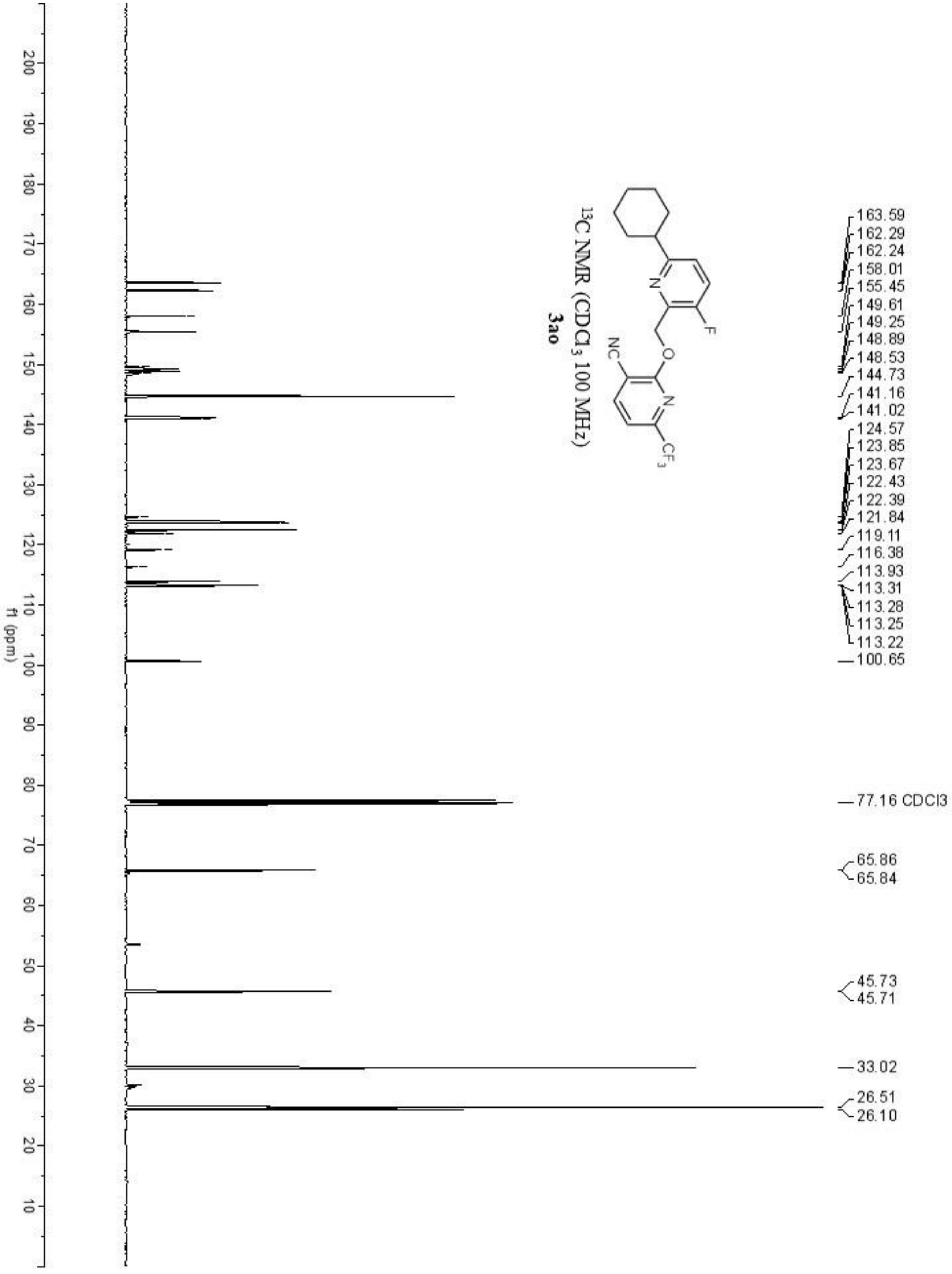

13C NMR (CDCl3 100 MHz)
3ao
NC
O
N
F
CF3
163.59
162.29
162.24
158.01
155.45
149.61
149.25
148.89
148.53
144.73
141.16
141.02
124.57
123.85
123.67
122.43
122.39
121.84
119.11
116.38
113.93
113.31
113.28
113.25
113.22
100.65
77.16 CDCl3
65.86
65.84
45.73
45.71
33.02
26.51
26.10
f1 (ppm)

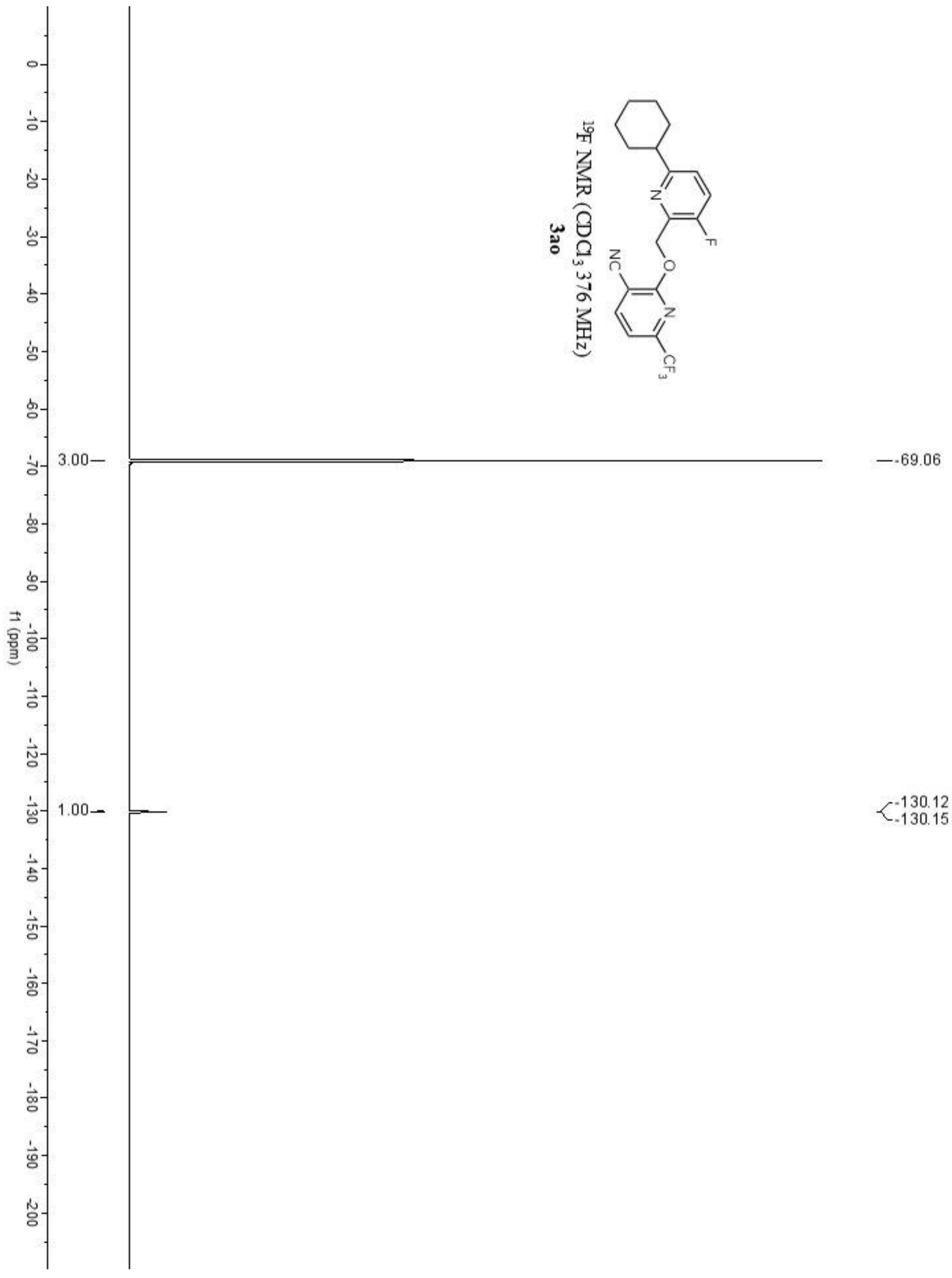
19F NMR (CDCl3 376 MHz)
3ao
NC
F
N
O
CF3
-69.06
-130.12
-130.15
3.00
1.00
f1 (ppm)

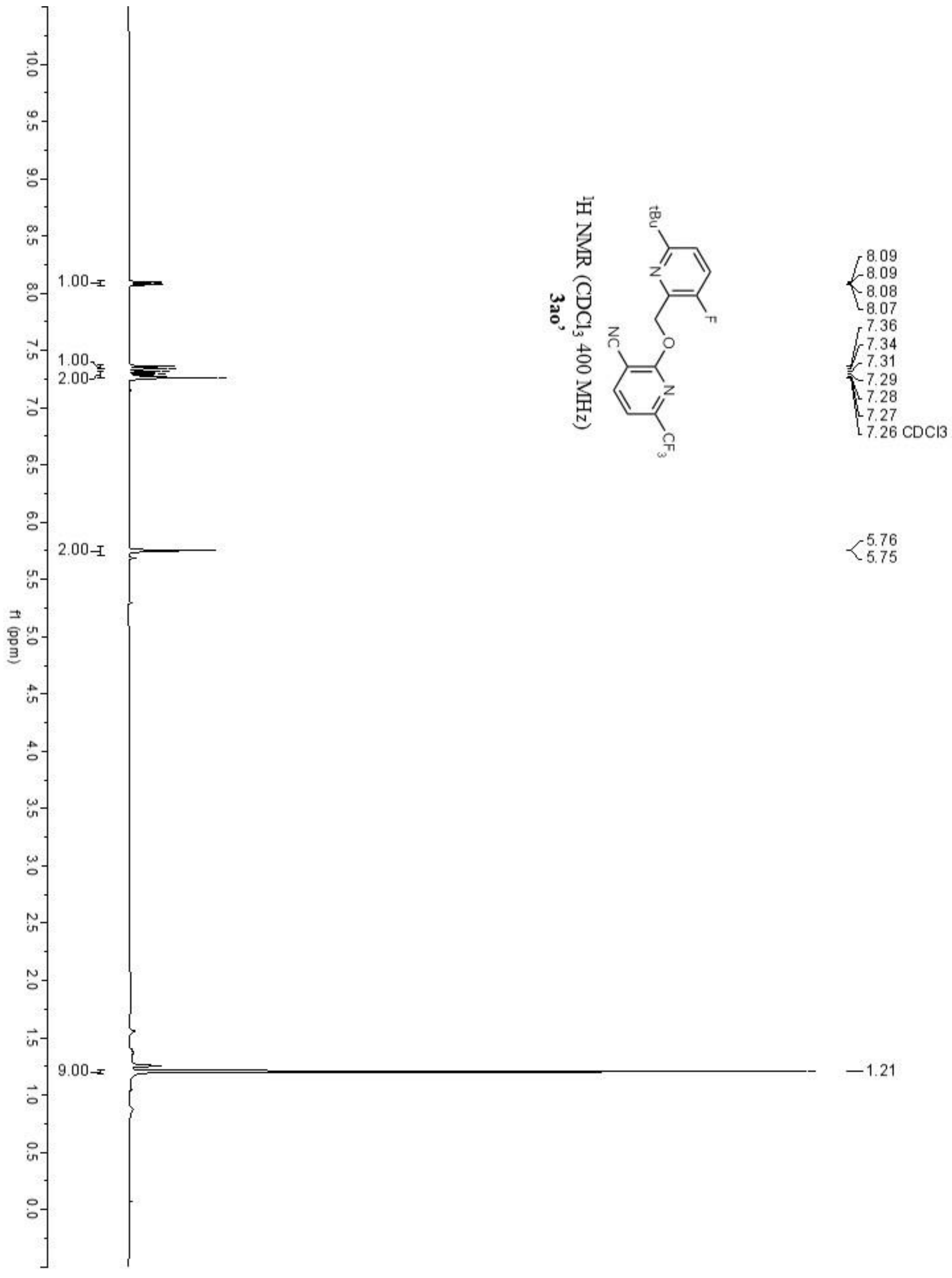

^{1}H NMR ($CDCl_3$ 400 MHz)

3ao'

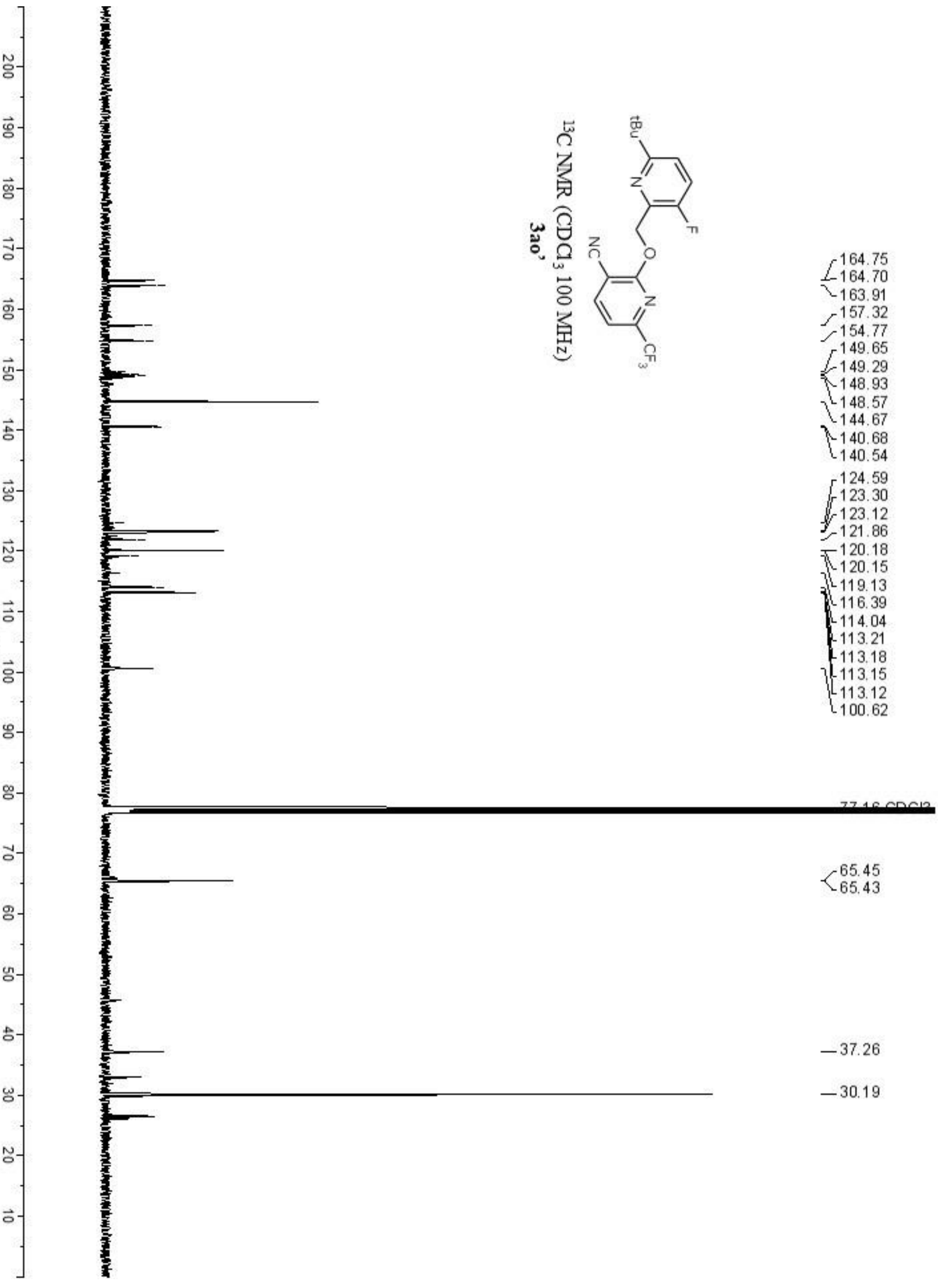
13C NMR (CDCl3 100 MHz)
3ao'
tBu
N
F
O
NC
N
CF3
164.75
164.70
163.91
157.32
154.77
149.65
149.29
148.93
148.57
144.67
140.68
140.54
124.59
123.30
123.12
121.86
120.18
120.15
119.13
116.39
114.04
113.21
113.18
113.15
113.12
100.62
77.16 CDCl3
65.45
65.43
37.26
30.19
f1 (ppm)

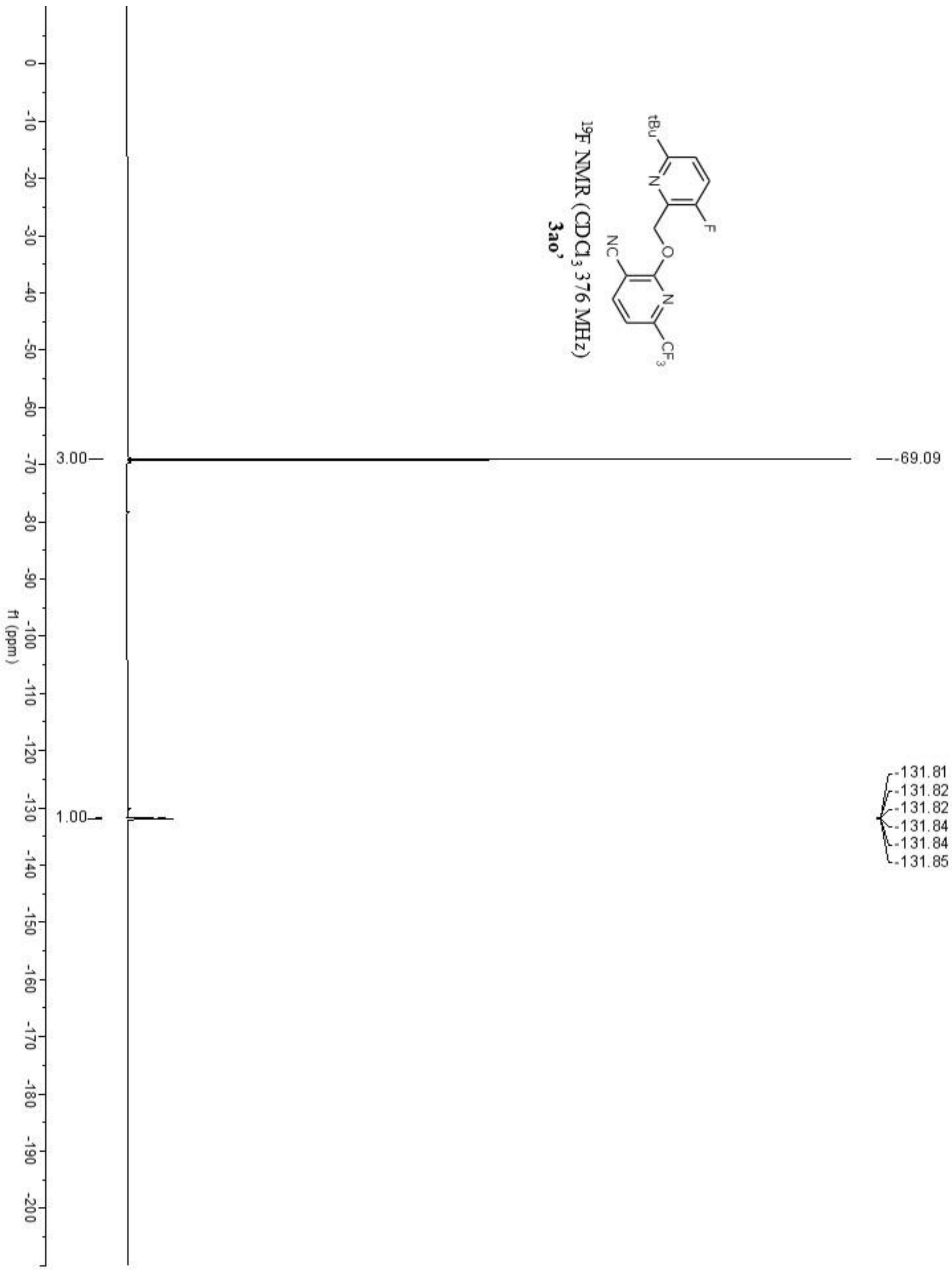

19F NMR (CDCl3 376 MHz)
3ao’
tBu
N
F
O
NC
N
CF3
-69.09
-131.81
-131.82
-131.82
-131.84
-131.84
-131.85
3.00
1.00
f1 (ppm)

Crude ^1H NMR ($CDCl_3$ 400 MHz)

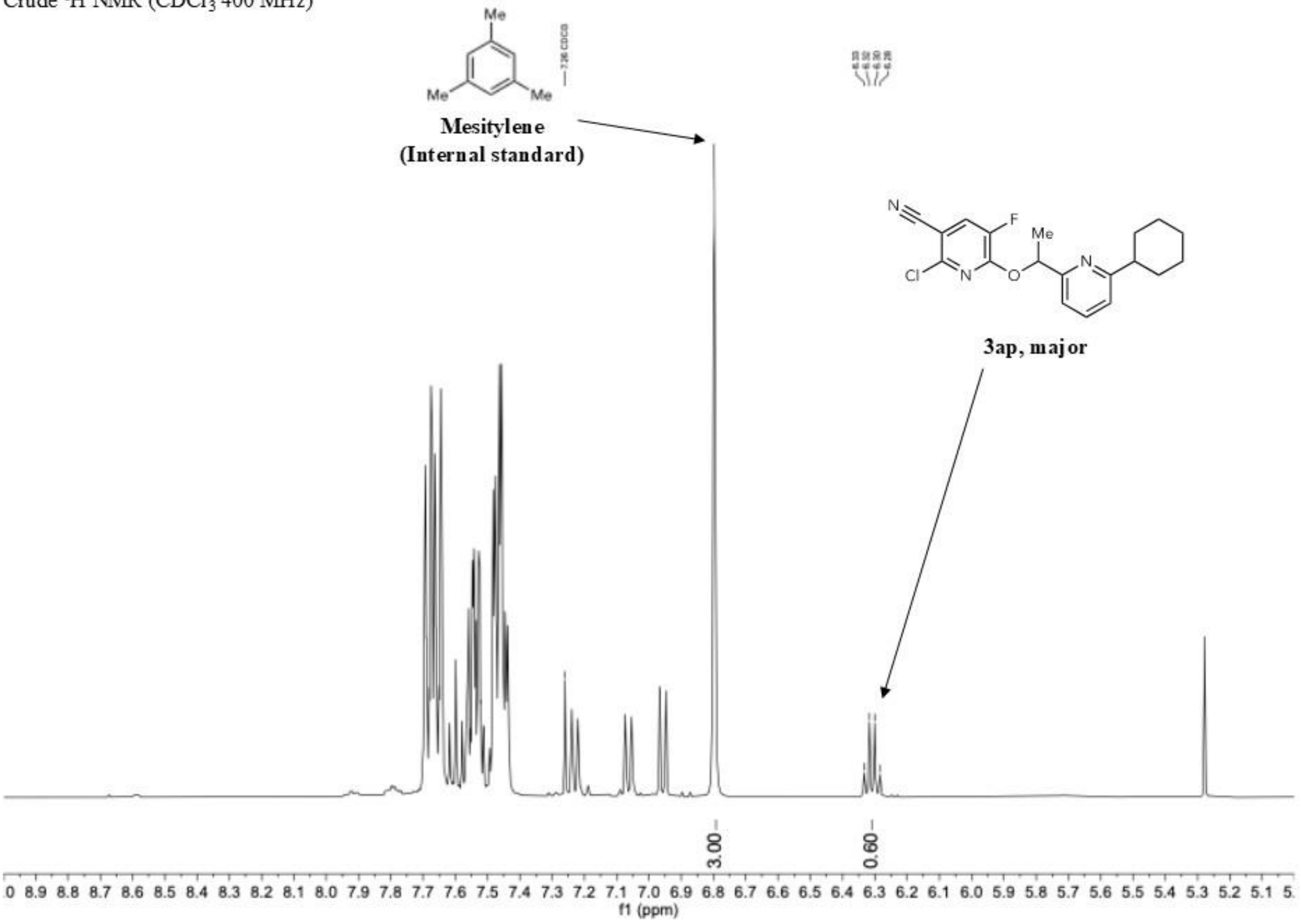

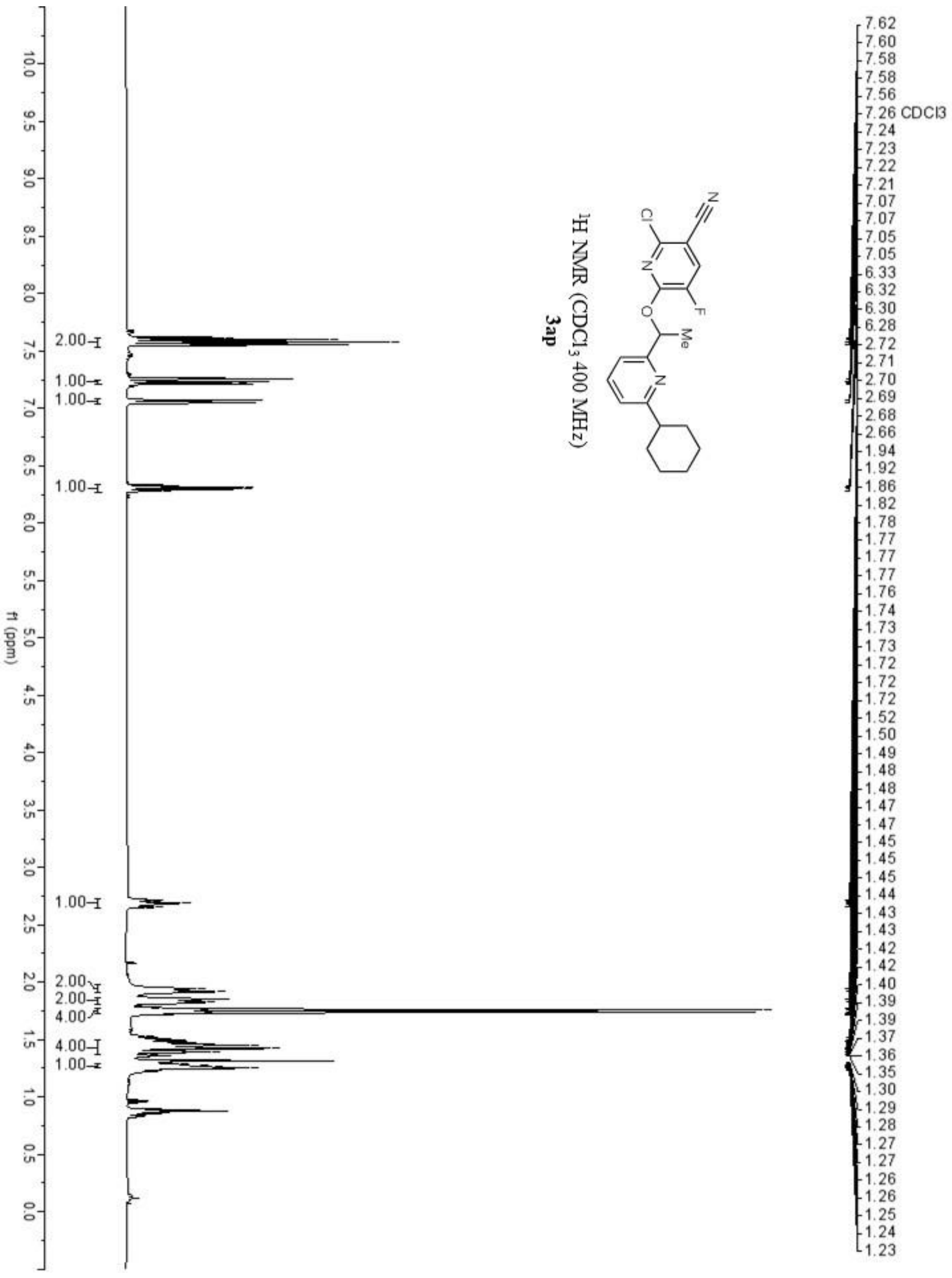

1H NMR (CDCl3 400 MHz)
3ap
Cl
N
O
Me
F
7.62
7.60
7.58
7.58
7.56
7.26 CDCl3
7.24
7.23
7.22
7.21
7.07
7.07
7.05
7.05
6.33
6.32
6.30
6.28
2.72
2.71
2.70
2.69
2.68
2.66
1.94
1.92
1.86
1.82
1.78
1.77
1.77
1.77
1.76
1.74
1.73
1.73
1.72
1.72
1.72
1.52
1.50
1.49
1.48
1.48
1.47
1.47
1.45
1.45
1.45
1.44
1.43
1.43
1.42
1.42
1.40
1.39
1.39
1.37
1.36
1.35
1.30
1.29
1.28
1.27
1.27
1.26
1.26
1.25
1.24
1.23
2.00
1.00
1.00
1.00
1.00
2.00
2.00
4.00
4.00
1.00
f1 (ppm)

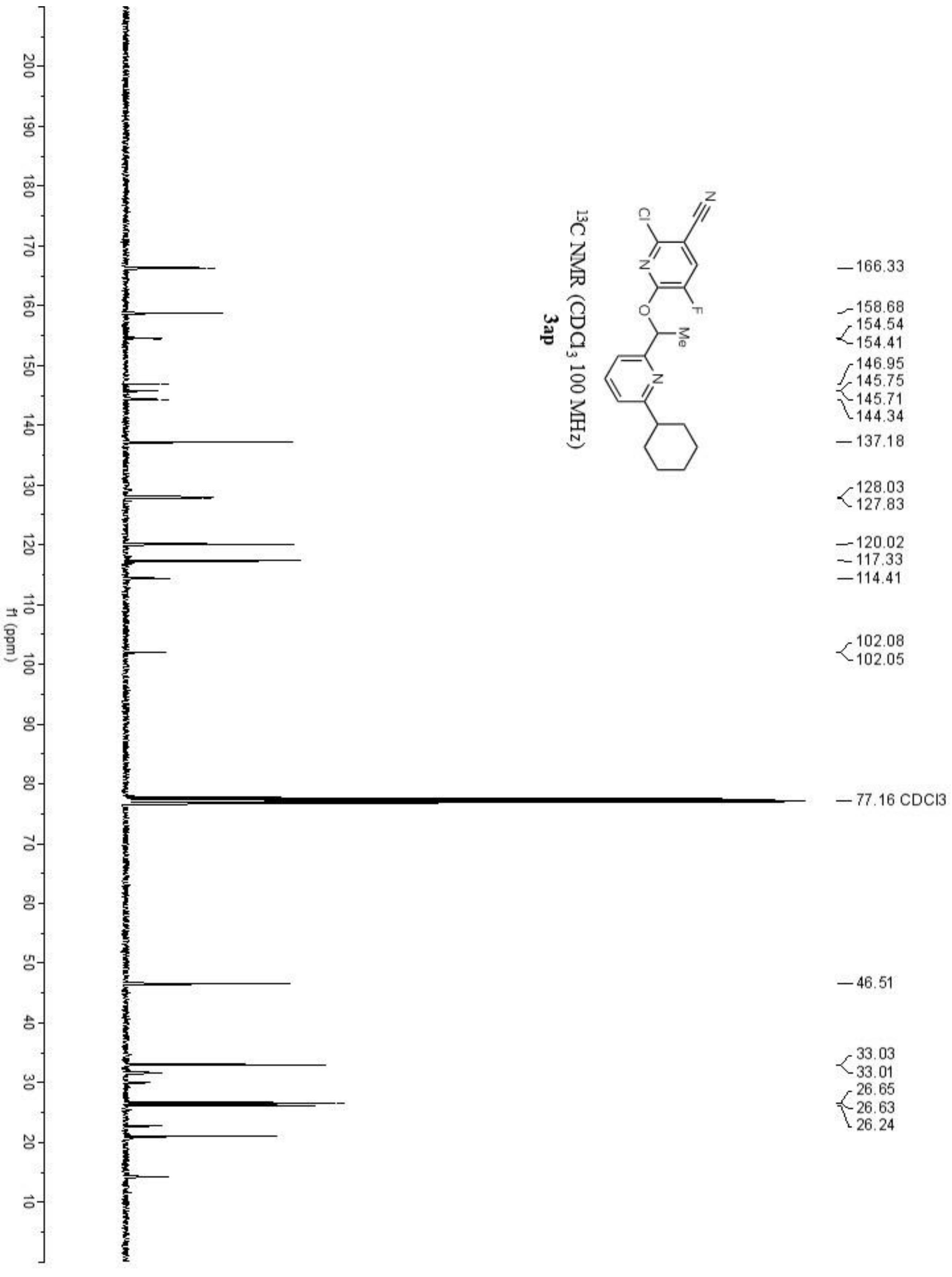

13C NMR (CDCl3 100 MHz)
3ap
Cl
N
O
F
Me
166.33
158.68
154.54
154.41
146.95
145.75
145.71
144.34
137.18
128.03
127.83
120.02
117.33
114.41
102.08
102.05
77.16 CDCl3
46.51
33.03
33.01
26.65
26.63
26.24
f1 (ppm)
200
190
180
170
160
150
140
130
120
110
100
90
80
70
60
50
40
30
20
10

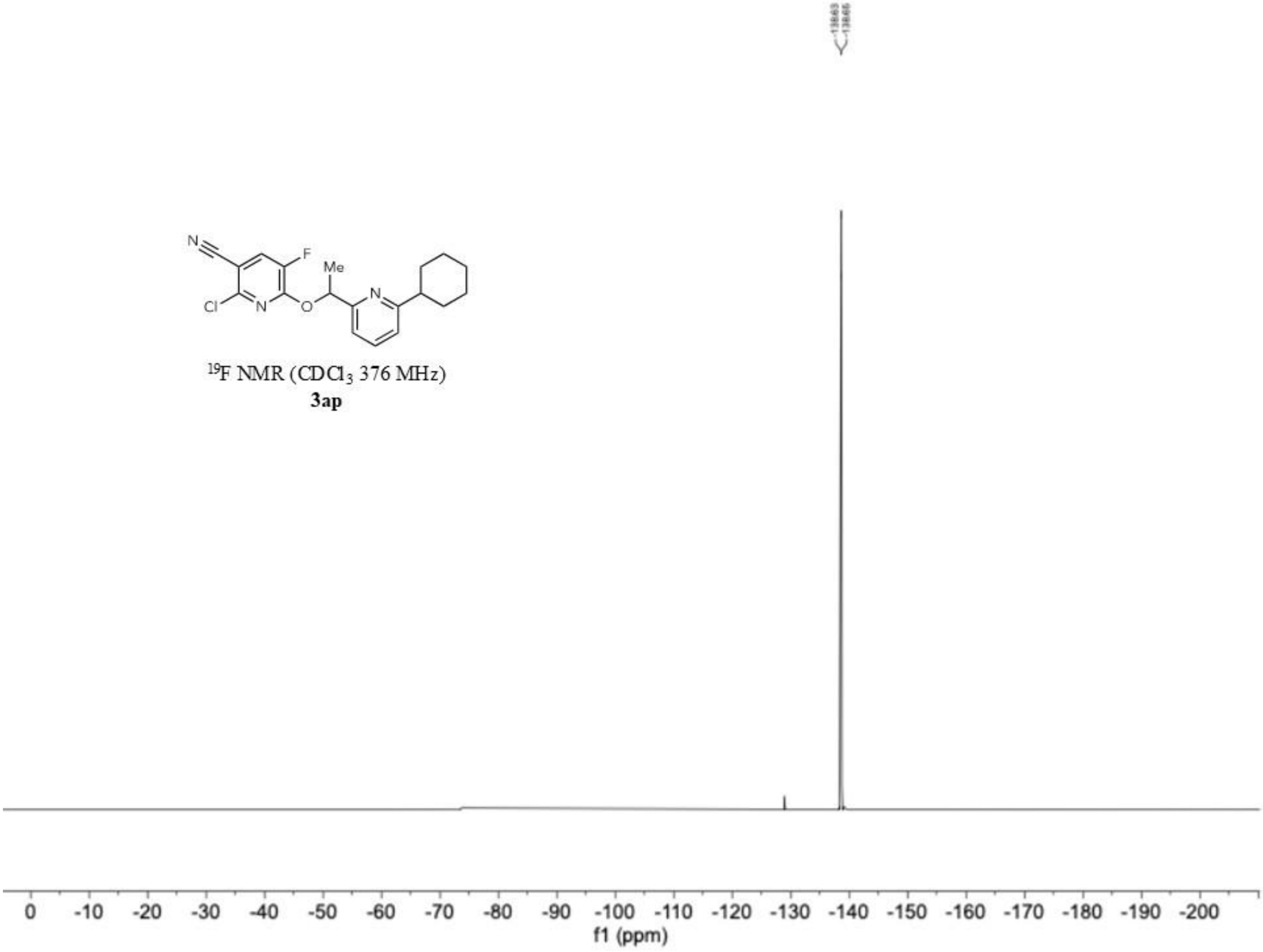

^{19}F NMR ($CDCl_3$ 376 MHz)
3ap

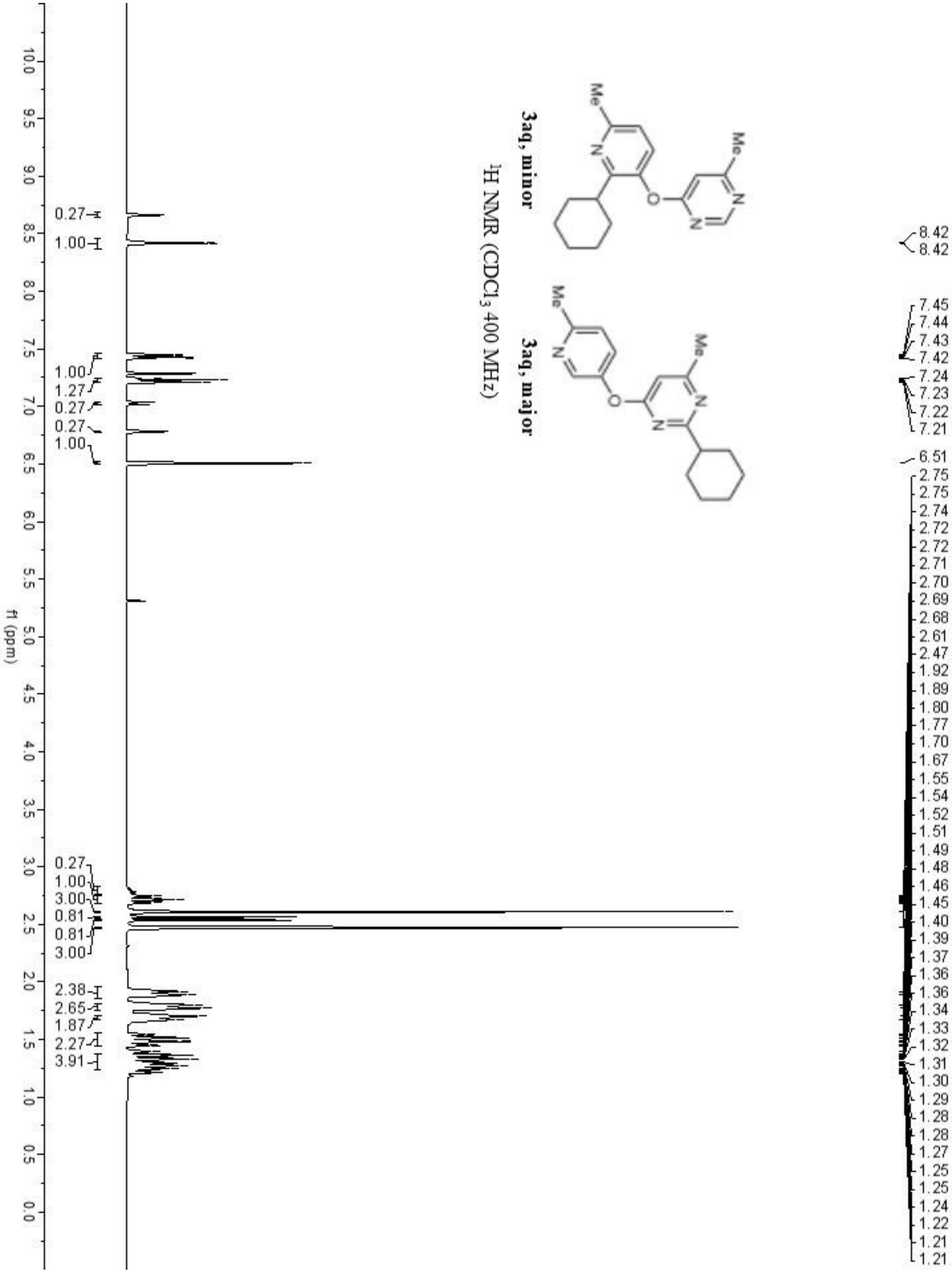
3aq, minor
3aq, major
1H NMR (CDCl3 400 MHz)
f1 (ppm)
8.42
8.42
7.45
7.44
7.43
7.42
7.24
7.23
7.22
7.21
6.51
2.75
2.75
2.74
2.72
2.72
2.71
2.70
2.69
2.68
2.61
2.47
1.92
1.89
1.80
1.77
1.70
1.67
1.55
1.54
1.52
1.51
1.49
1.48
1.46
1.45
1.40
1.39
1.37
1.36
1.36
1.34
1.33
1.32
1.31
1.30
1.29
1.28
1.28
1.27
1.25
1.25
1.24
1.22
1.21
1.21

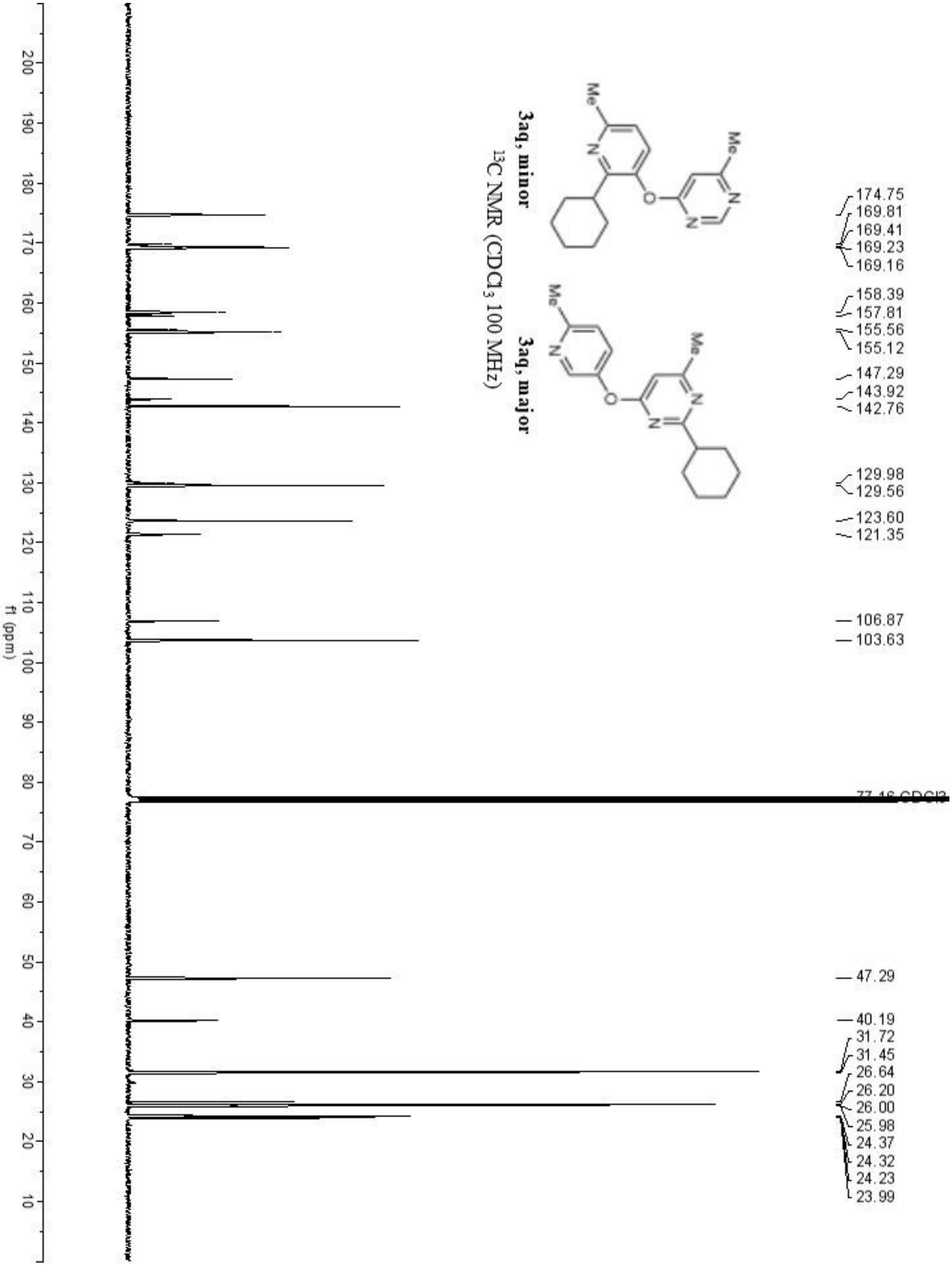
3aq, minor
3aq, major
13C NMR (CDCl3 100 MHz)
174.75
169.81
169.41
169.23
169.16
158.39
157.81
155.56
155.12
147.29
143.92
142.76
129.98
129.56
123.60
121.35
106.87
103.63
77.16 CDCl3
47.29
40.19
31.72
31.45
26.64
26.20
26.00
25.98
24.37
24.32
24.23
23.99
f1 (ppm)

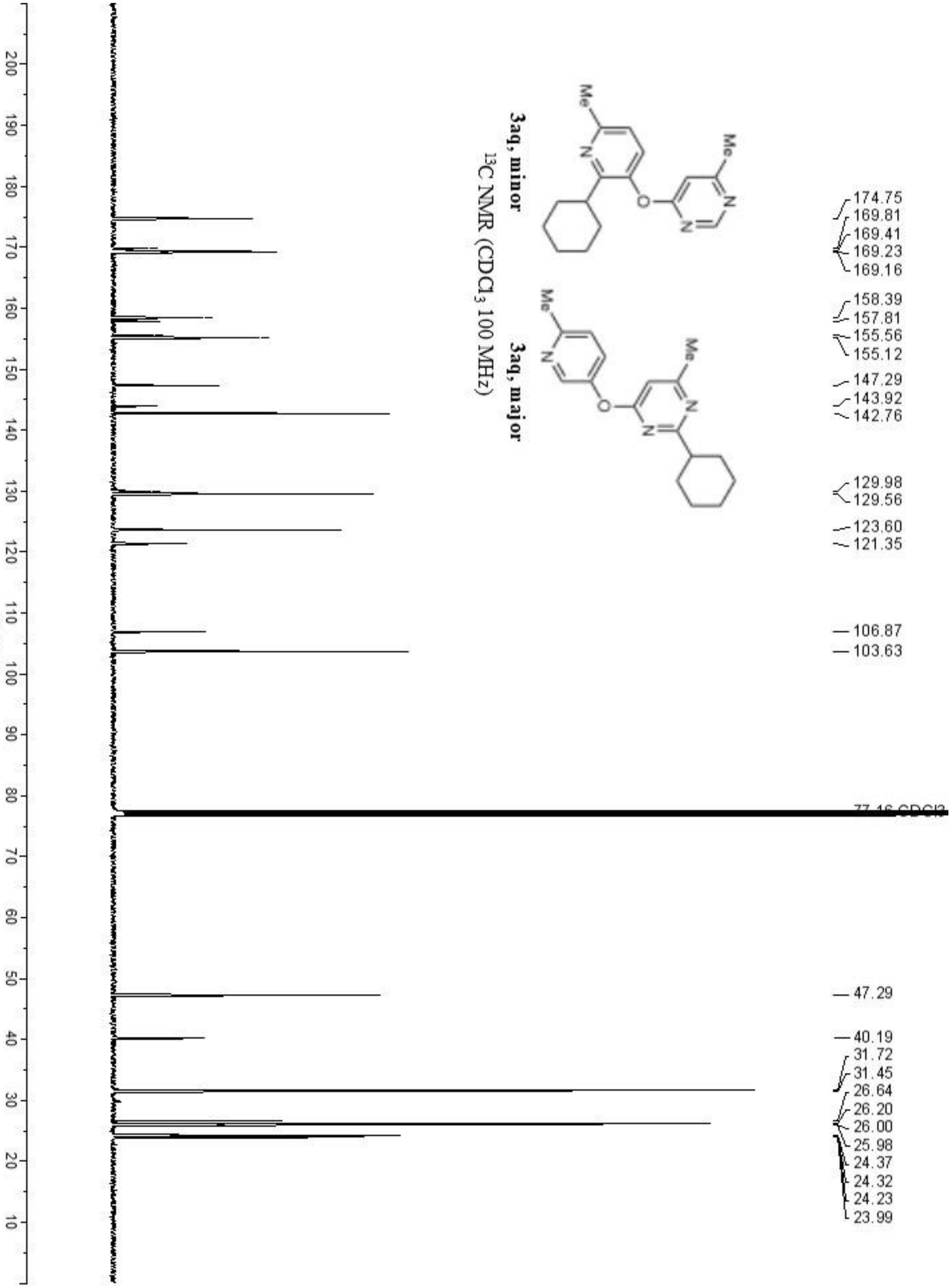
3aq, minor
3aq, major
13C NMR (CDCl3 100 MHz)
174.75
169.81
169.41
169.23
169.16
158.39
157.81
155.56
155.12
147.29
143.92
142.76
129.98
129.56
123.60
121.35
106.87
103.63
77.16 CDCl3
47.29
40.19
31.72
31.45
26.64
26.20
26.00
25.98
24.37
24.32
24.23
23.99
f1 (ppm)

Crude ^{1}H NMR (CD_3CN 400 MHz)

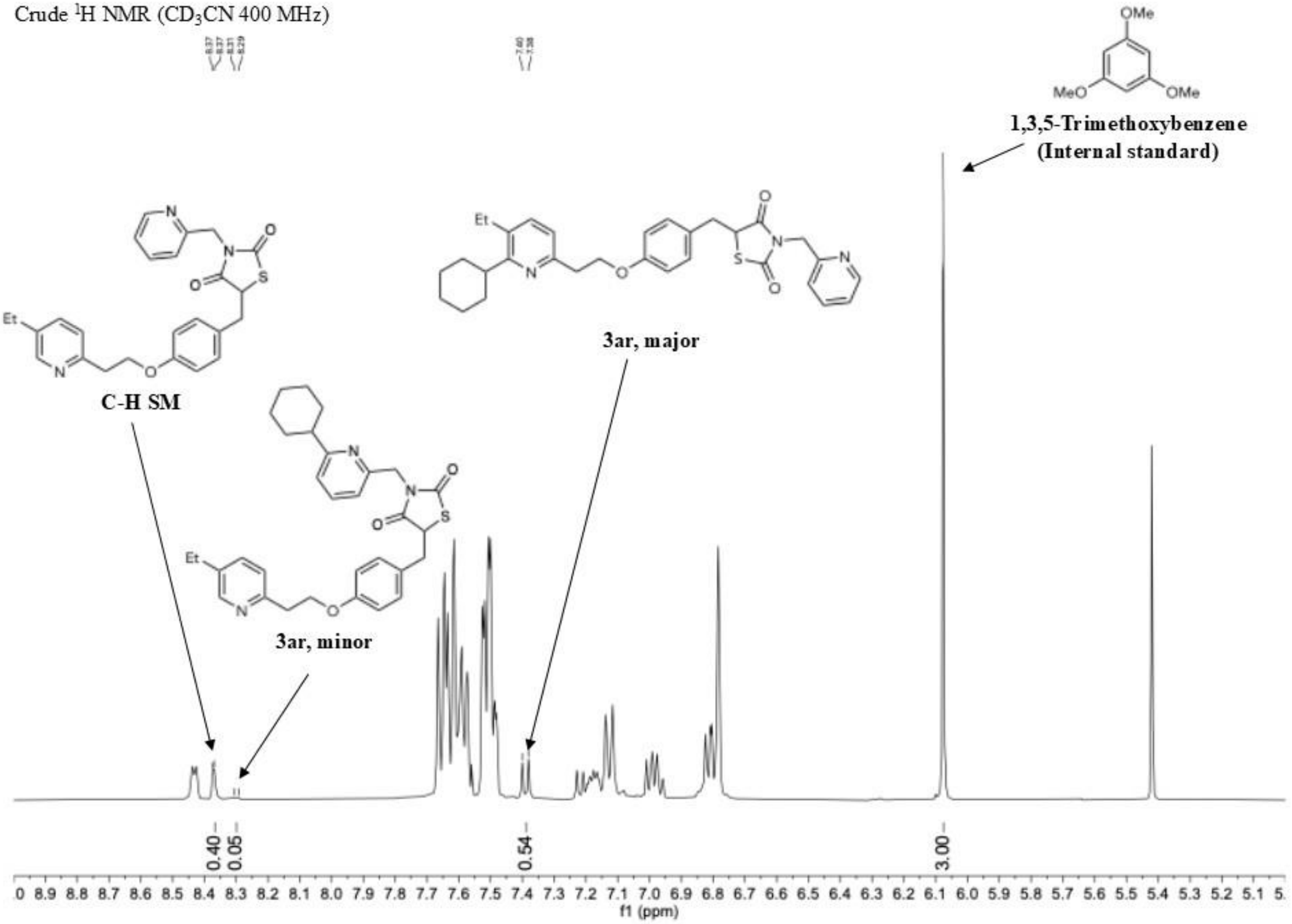

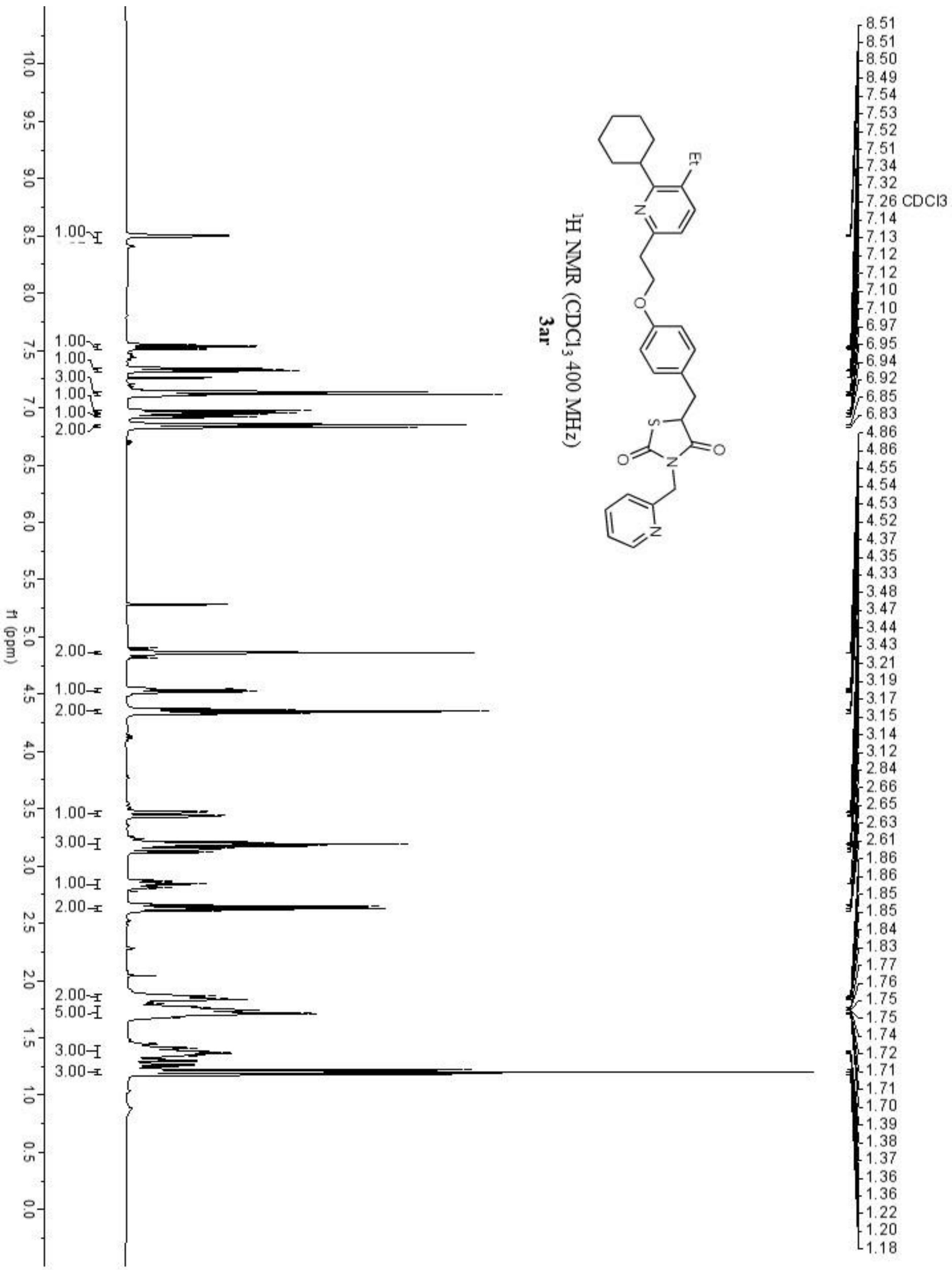

1H NMR (CDCl3 400 MHz)
3ar
Et
N
O
S
N
O
O
N
8.51
8.51
8.50
8.49
7.54
7.53
7.52
7.51
7.34
7.32
7.26 CDCl3
7.14
7.13
7.12
7.12
7.10
7.10
6.97
6.95
6.94
6.92
6.85
6.83
4.86
4.86
4.55
4.54
4.53
4.52
4.37
4.35
4.33
3.48
3.47
3.44
3.43
3.21
3.19
3.17
3.15
3.14
3.12
2.84
2.66
2.65
2.63
2.61
1.86
1.86
1.85
1.85
1.84
1.83
1.77
1.76
1.75
1.75
1.74
1.72
1.71
1.71
1.70
1.39
1.38
1.37
1.36
1.36
1.22
1.20
1.18
1.00
1.00
1.00
3.00
1.00
1.00
2.00
2.00
1.00
2.00
1.00
3.00
1.00
2.00
2.00
5.00
3.00
3.00
f1 (ppm)

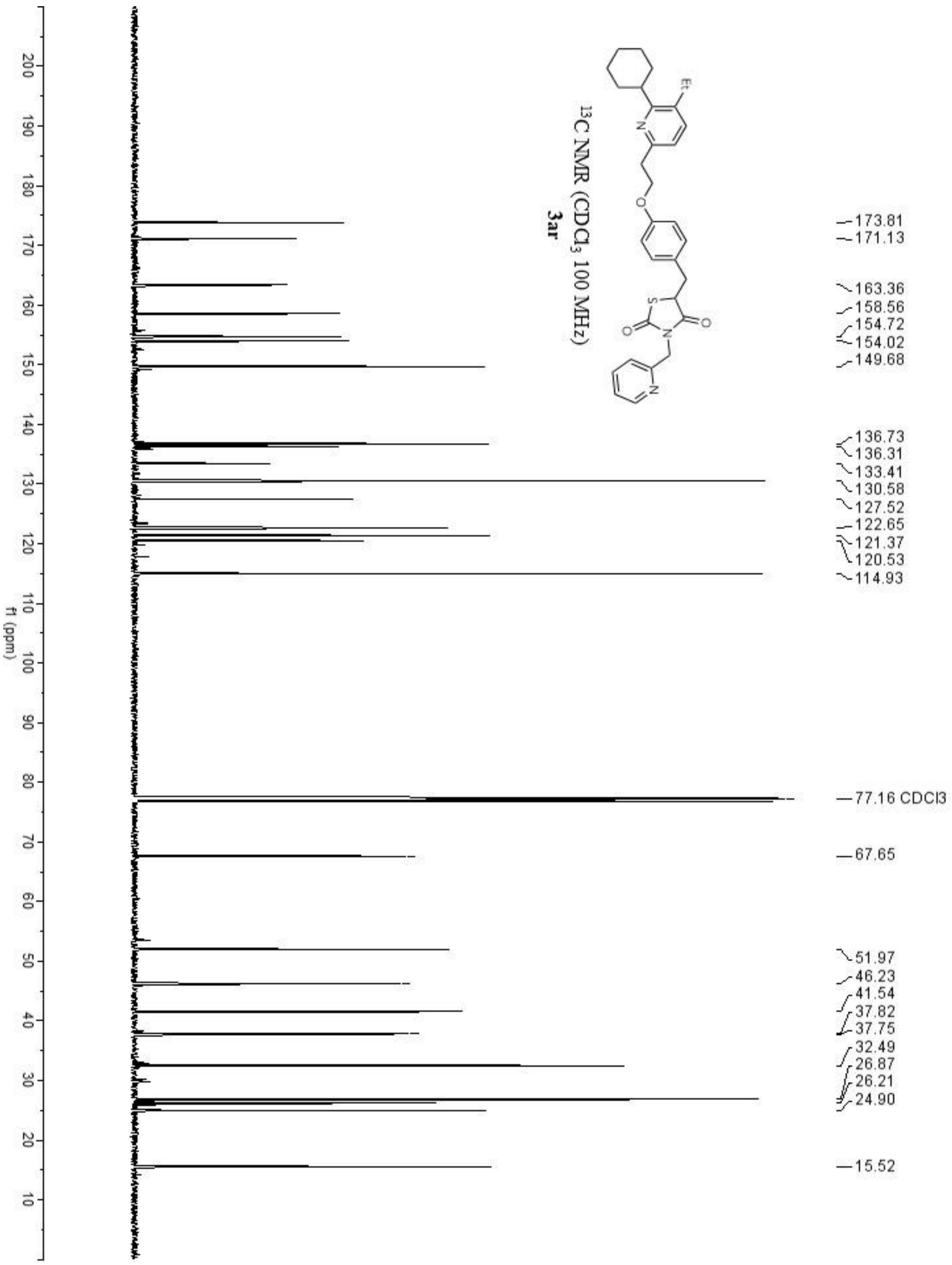

13C NMR (CDCl3 100 MHz)
3ar
Et
N
O
S
173.81
171.13
163.36
158.56
154.72
154.02
149.68
136.73
136.31
133.41
130.58
127.52
122.65
121.37
120.53
114.93
77.16 CDCl3
67.65
51.97
46.23
41.54
37.82
37.75
32.49
26.87
26.21
24.90
15.52
f1 (ppm)
200
190
180
170
160
150
140
130
120
110
100
90
80
70
60
50
40
30
20
10

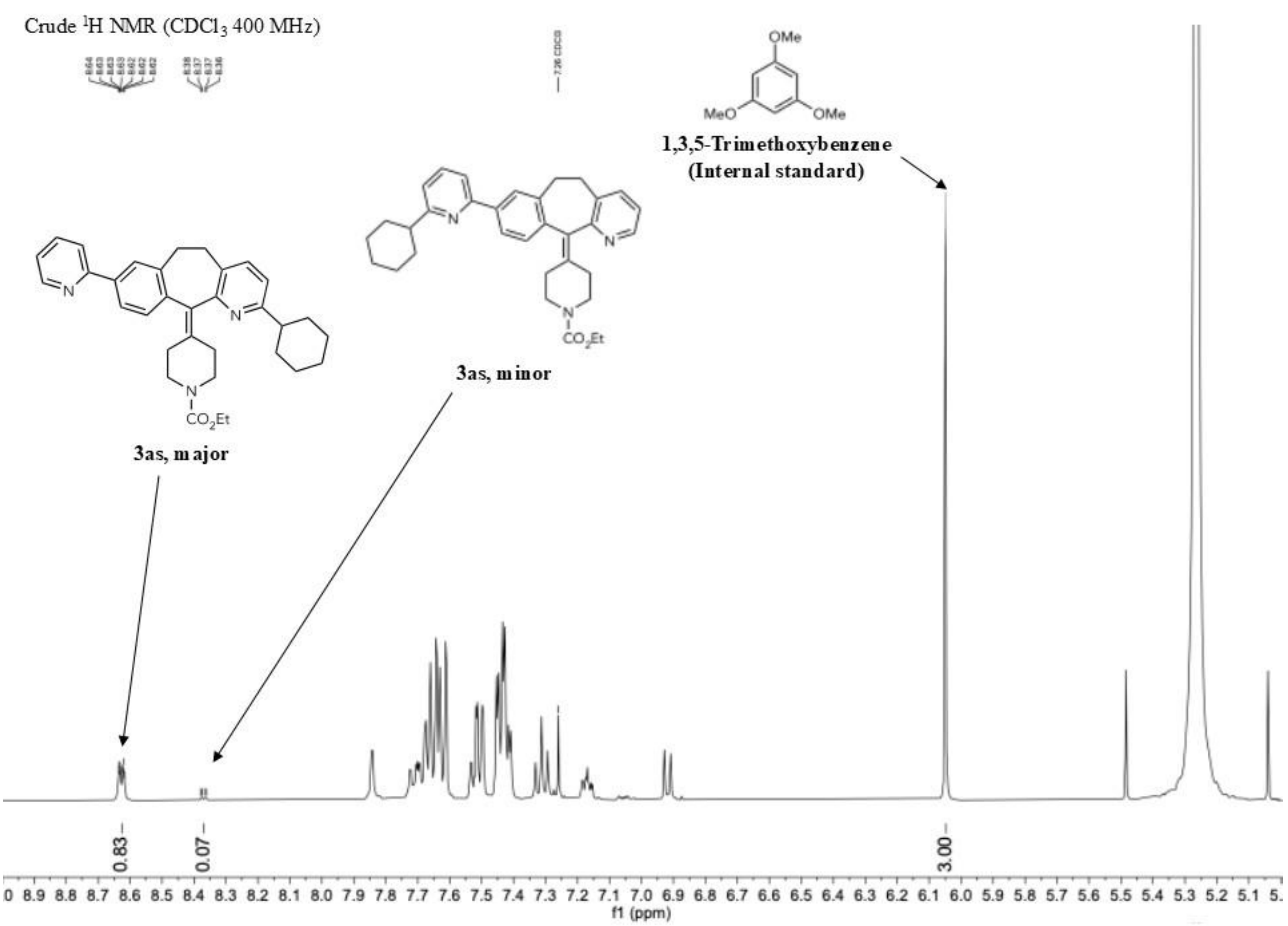
Crude ^1H NMR ($CDCl_3$ 400 MHz)
8.64 8.63 8.63 8.63 8.63 8.62 8.62 8.62
8.38 8.37 8.37 8.36
7.26 CDCl3
OMe
MeO
OMe
1,3,5-Trimethoxybenzene
(Internal standard)
N
N
N
CO$_2$Et
3as, major
N
N
N
CO$_2$Et
3as, minor
0.83
0.07
3.00
0 8.9 8.8 8.7 8.6 8.5 8.4 8.3 8.2 8.1 8.0 7.9 7.8 7.7 7.6 7.5 7.4 7.3 7.2 7.1 7.0 6.9 6.8 6.7 6.6 6.5 6.4 6.3 6.2 6.1 6.0 5.9 5.8 5.7 5.6 5.5 5.4 5.3 5.2 5.1 5.
f1 (ppm)

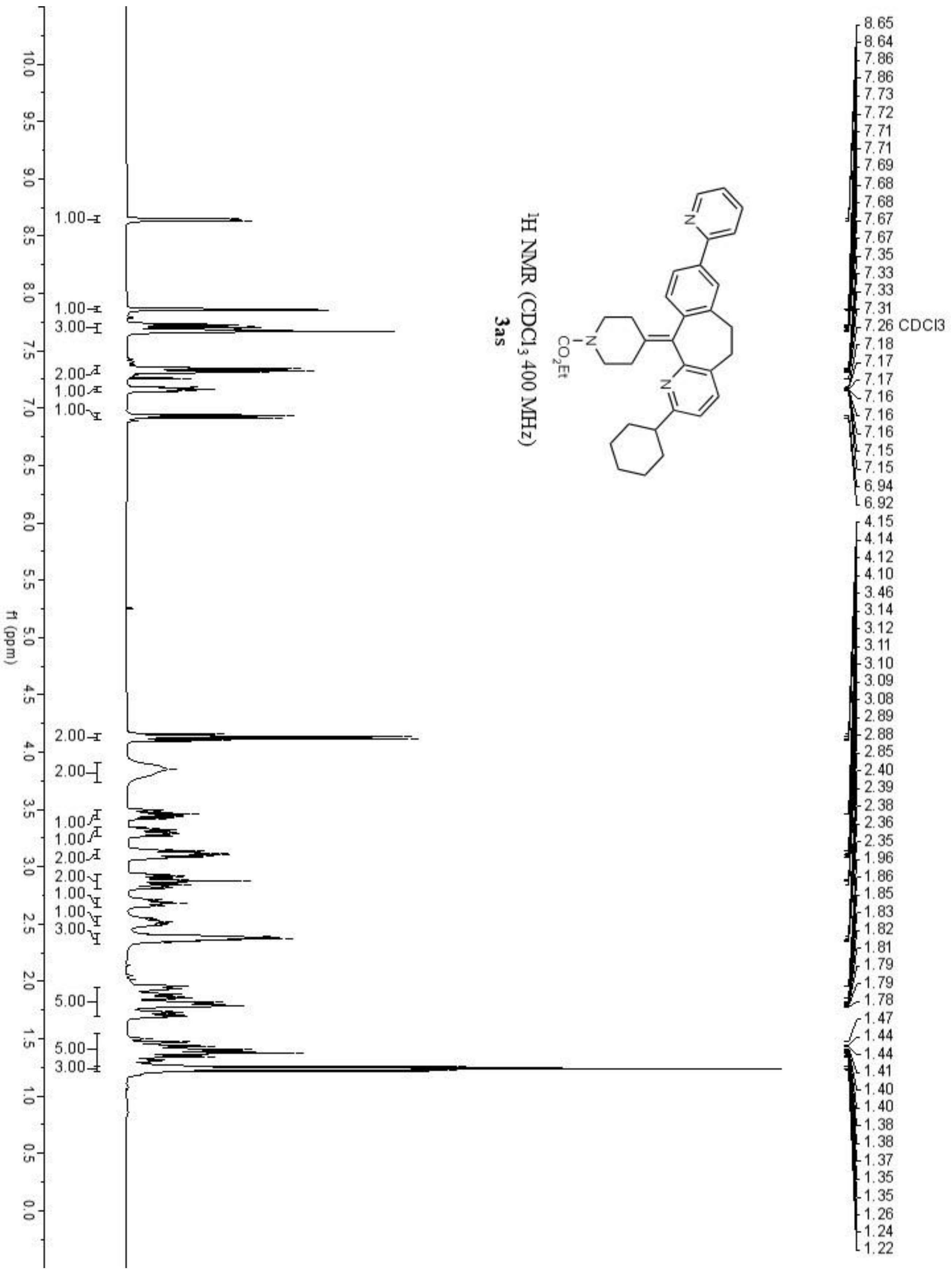

1H NMR (CDCl3 400 MHz)
3as
CO2Et
N
8.65
8.64
7.86
7.86
7.73
7.72
7.71
7.71
7.69
7.68
7.68
7.67
7.67
7.35
7.33
7.33
7.31
7.26 CDCl3
7.18
7.17
7.17
7.16
7.16
7.16
7.15
7.15
6.94
6.92
4.15
4.14
4.12
4.10
3.46
3.14
3.12
3.11
3.10
3.09
3.08
2.89
2.88
2.85
2.40
2.39
2.38
2.36
2.35
1.96
1.86
1.85
1.83
1.82
1.81
1.79
1.79
1.78
1.47
1.44
1.44
1.41
1.40
1.40
1.38
1.38
1.37
1.35
1.35
1.26
1.24
1.22
f1 (ppm)

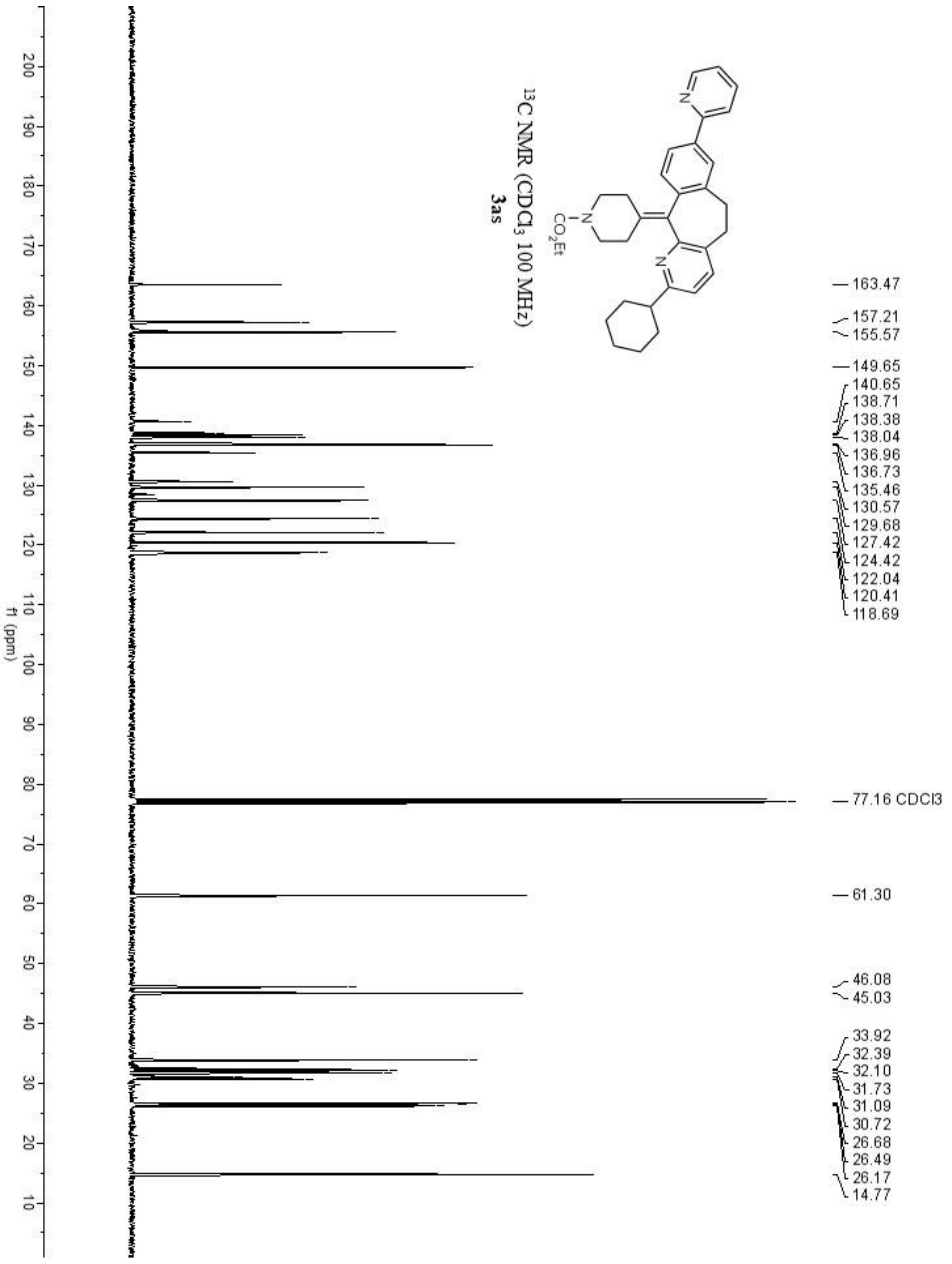

^{13}C NMR ($CDCl_3$ 100 MHz)
3as
N
N
N
CO_2Et
163.47
157.21
155.57
149.65
140.65
138.71
138.38
138.04
136.96
136.73
135.46
130.57
129.68
127.42
124.42
122.04
120.41
118.69
77.16 CDCl3
61.30
46.08
45.03
33.92
32.39
32.10
31.73
31.09
30.72
26.68
26.49
26.17
14.77
f1 (ppm)

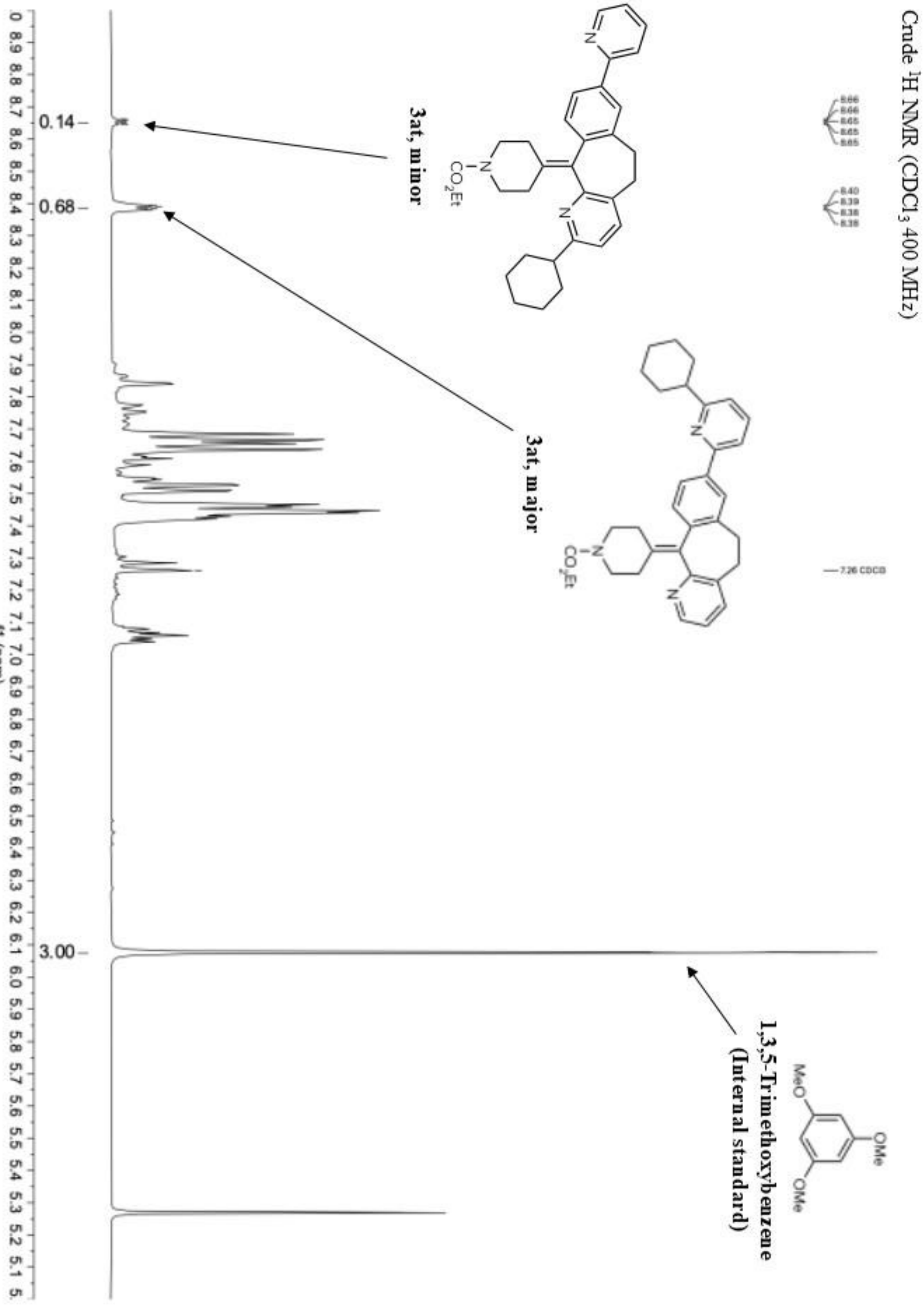
Crude 1H NMR (CDCl3 400 MHz)
3at, minor
3at, major
1,3,5-Trimethoxybenzene
(Internal standard)
0.14
0.68
3.00
7.26 CDCl3
f1 (ppm)

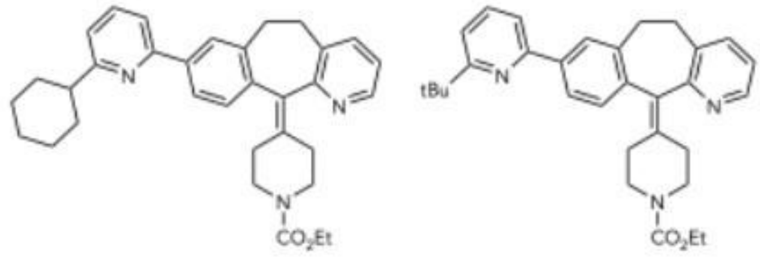

Major

tBu side pdt

^{1}H NMR ($CDCl_3$ 400 MHz)

3at

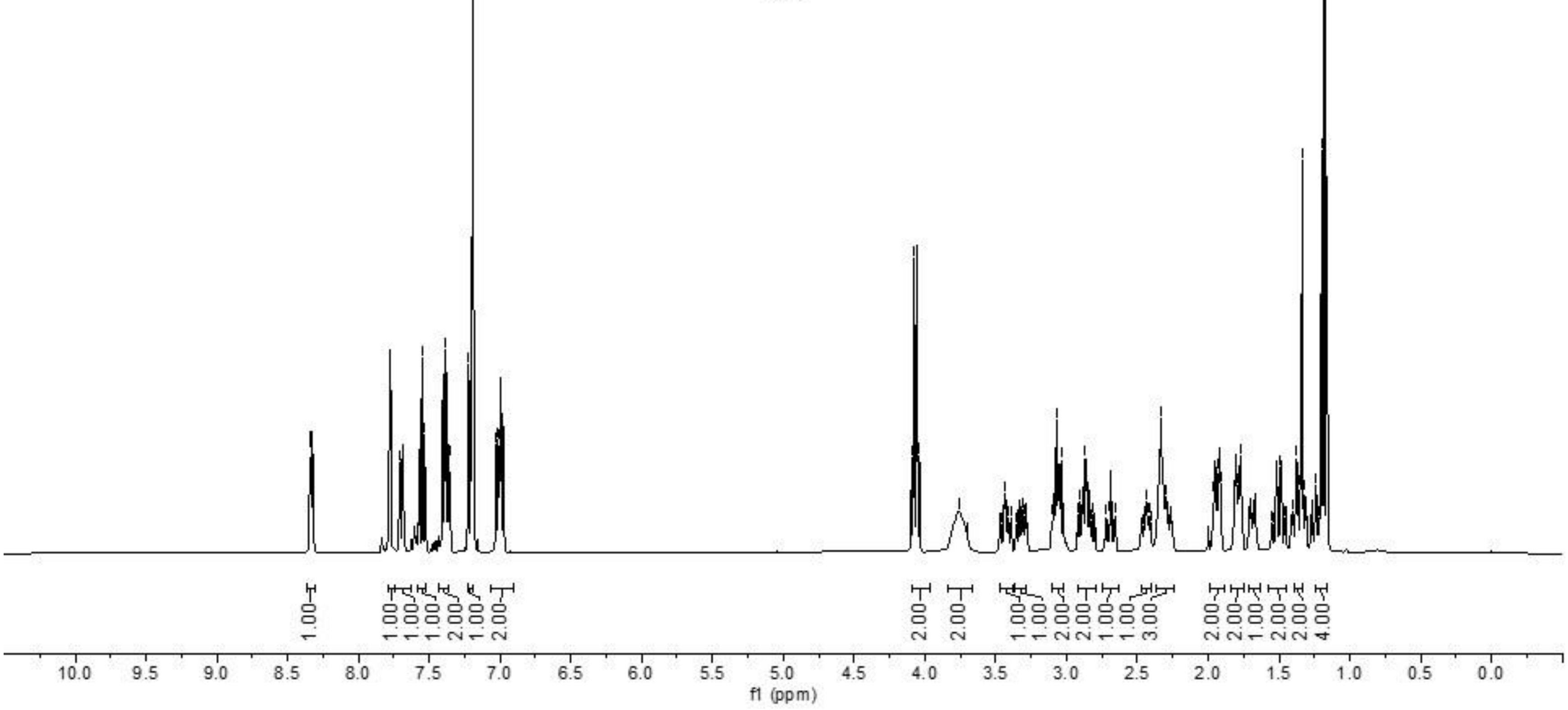

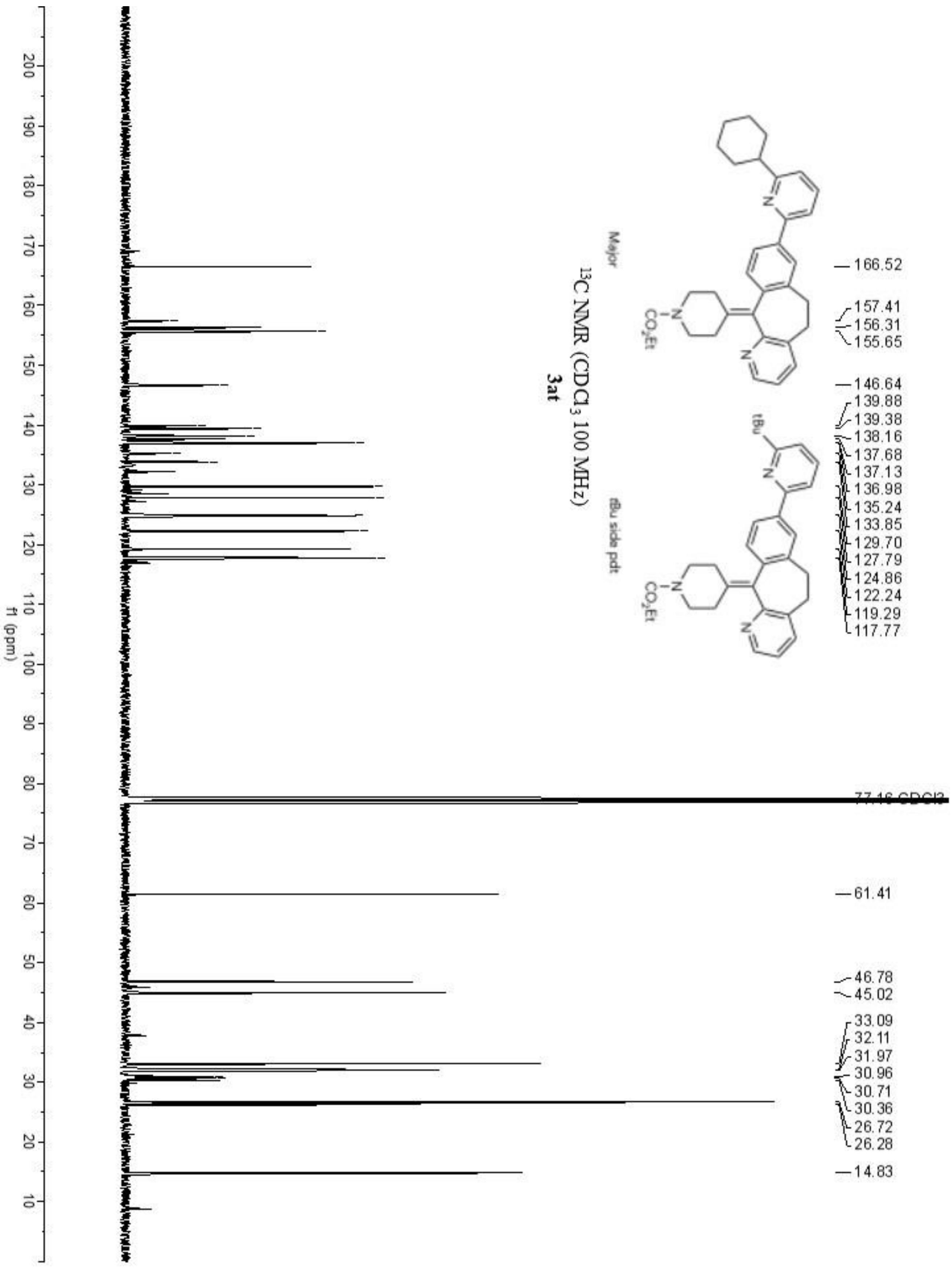

13C NMR (CDCl3 100 MHz)
3at
Major
tBu side pdt
tBu
CO2Et
N
166.52
157.41
156.31
155.65
146.64
139.88
139.38
138.16
137.68
137.13
136.98
135.24
133.85
129.70
127.79
124.86
122.24
119.29
117.77
77.16 CDCl3
61.41
46.78
45.02
33.09
32.11
31.97
30.96
30.71
30.36
26.72
26.28
14.83
f1 (ppm)
200
190
180
170
160
150
140
130
120
110
100
90
80
70
60
50
40
30
20
10

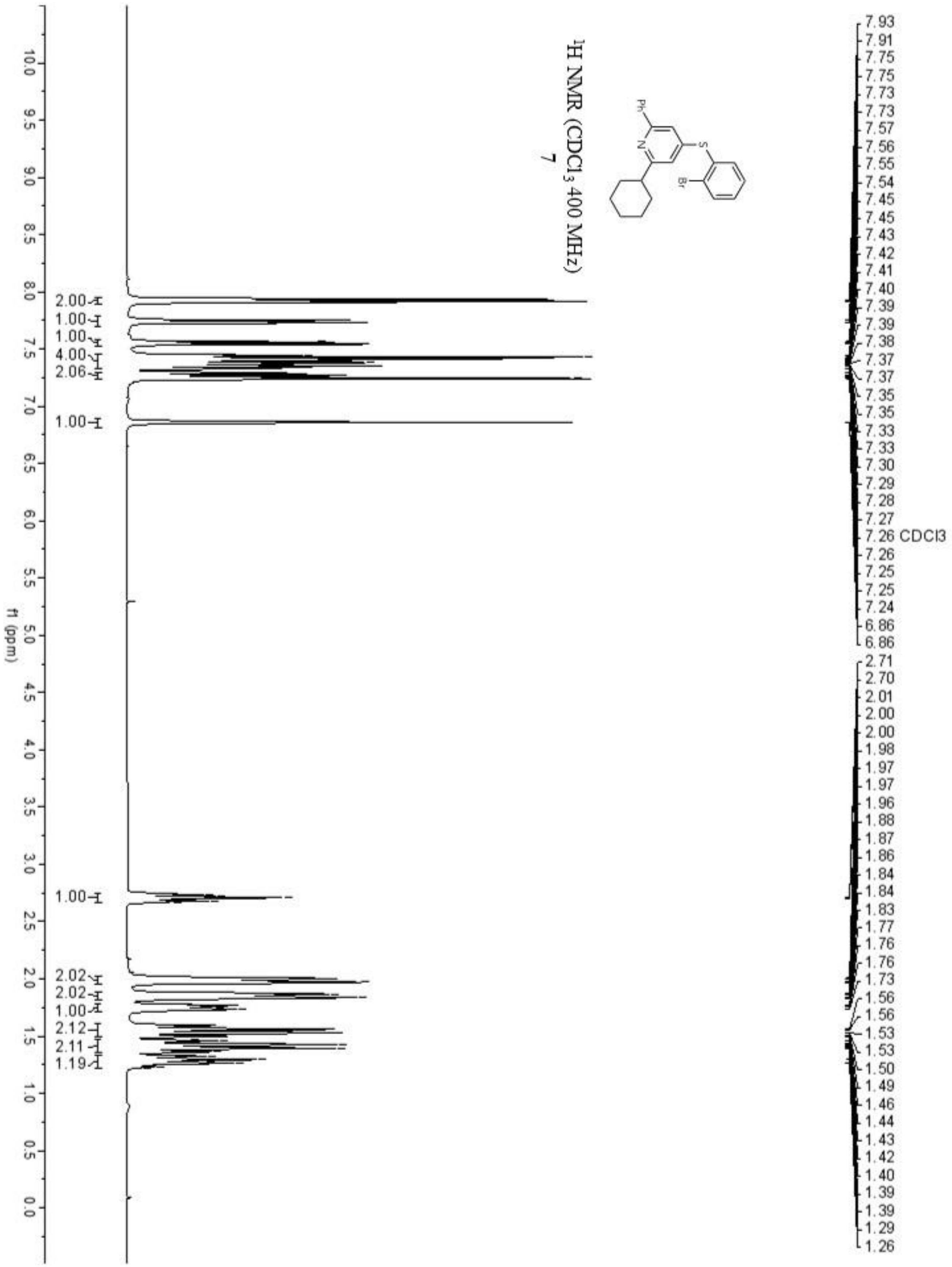
¹H NMR (CDCl₃ 400 MHz)
7
Ph
N
S
Br
f1 (ppm)
7.26 CDCl3

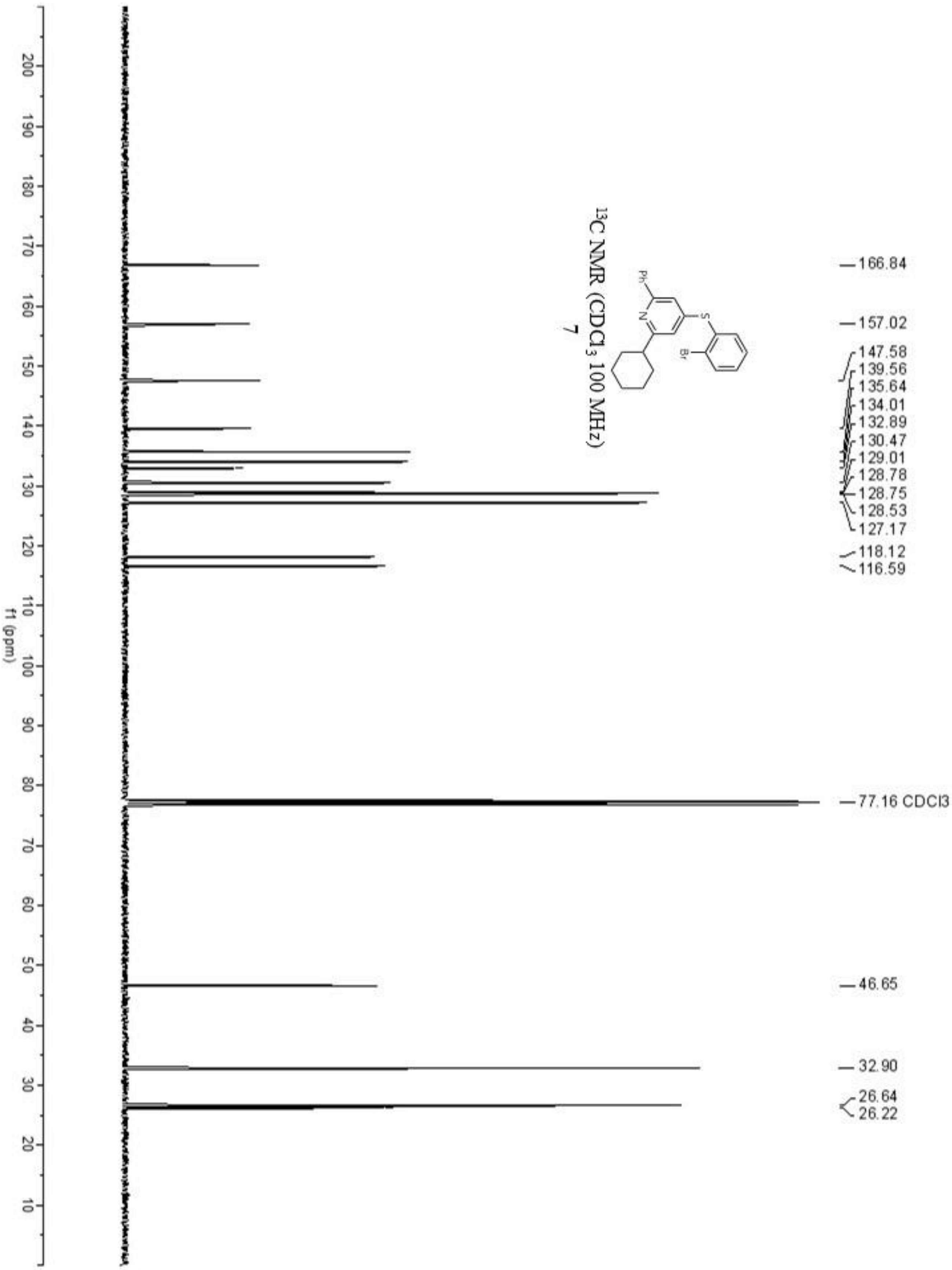
13C NMR (CDCl3 100 MHz)
7
Ph
N
S
Br
166.84
157.02
147.58
139.56
135.64
134.01
132.89
130.47
129.01
128.78
128.75
128.53
127.17
118.12
116.59
77.16 CDCl3
46.65
32.90
26.64
26.22
f1 (ppm)
200
190
180
170
160
150
140
130
120
110
100
90
80
70
60
50
40
30
20
10

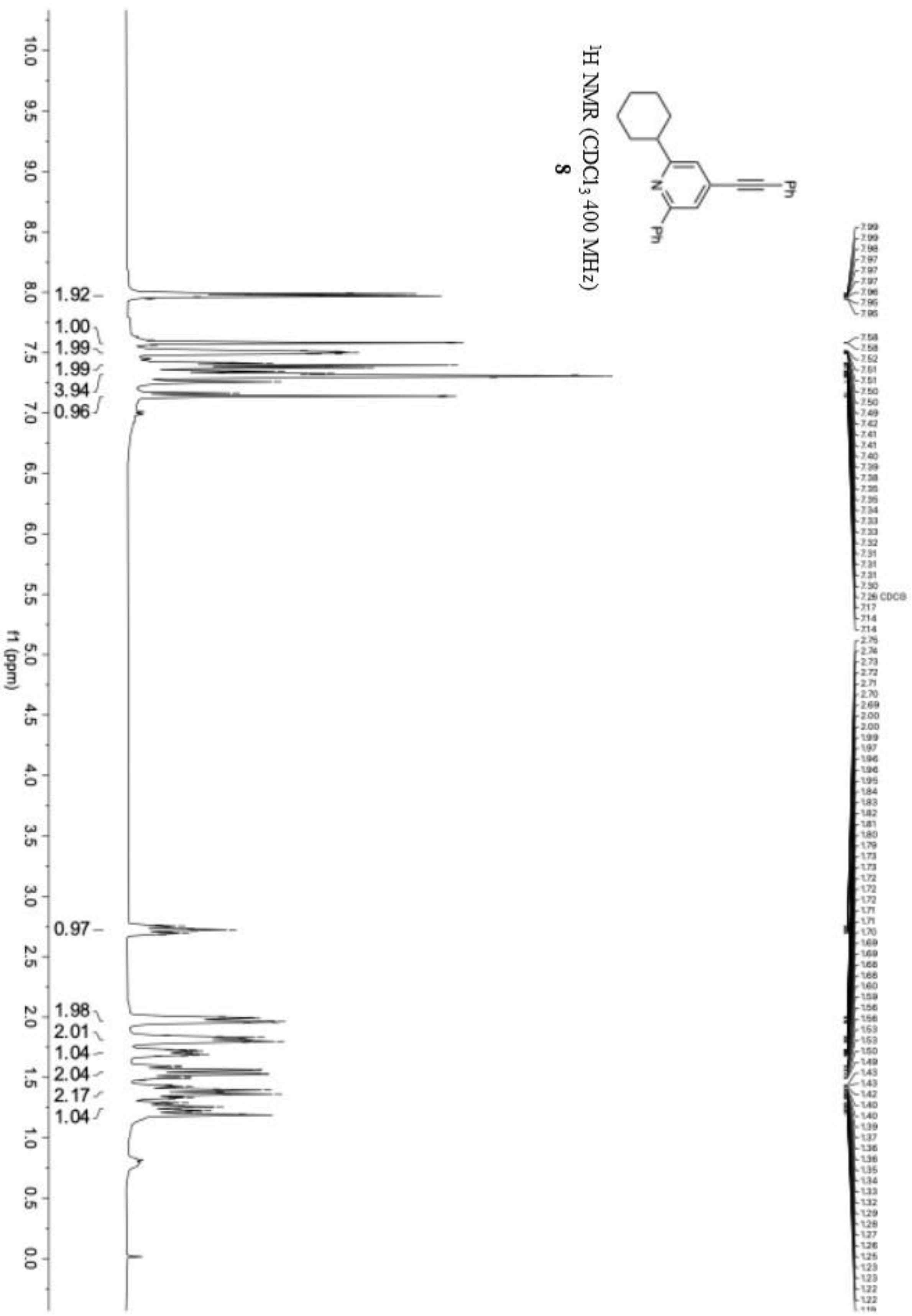

^{1}H NMR (CDCl$_3$ 400 MHz)
8
f1 (ppm)

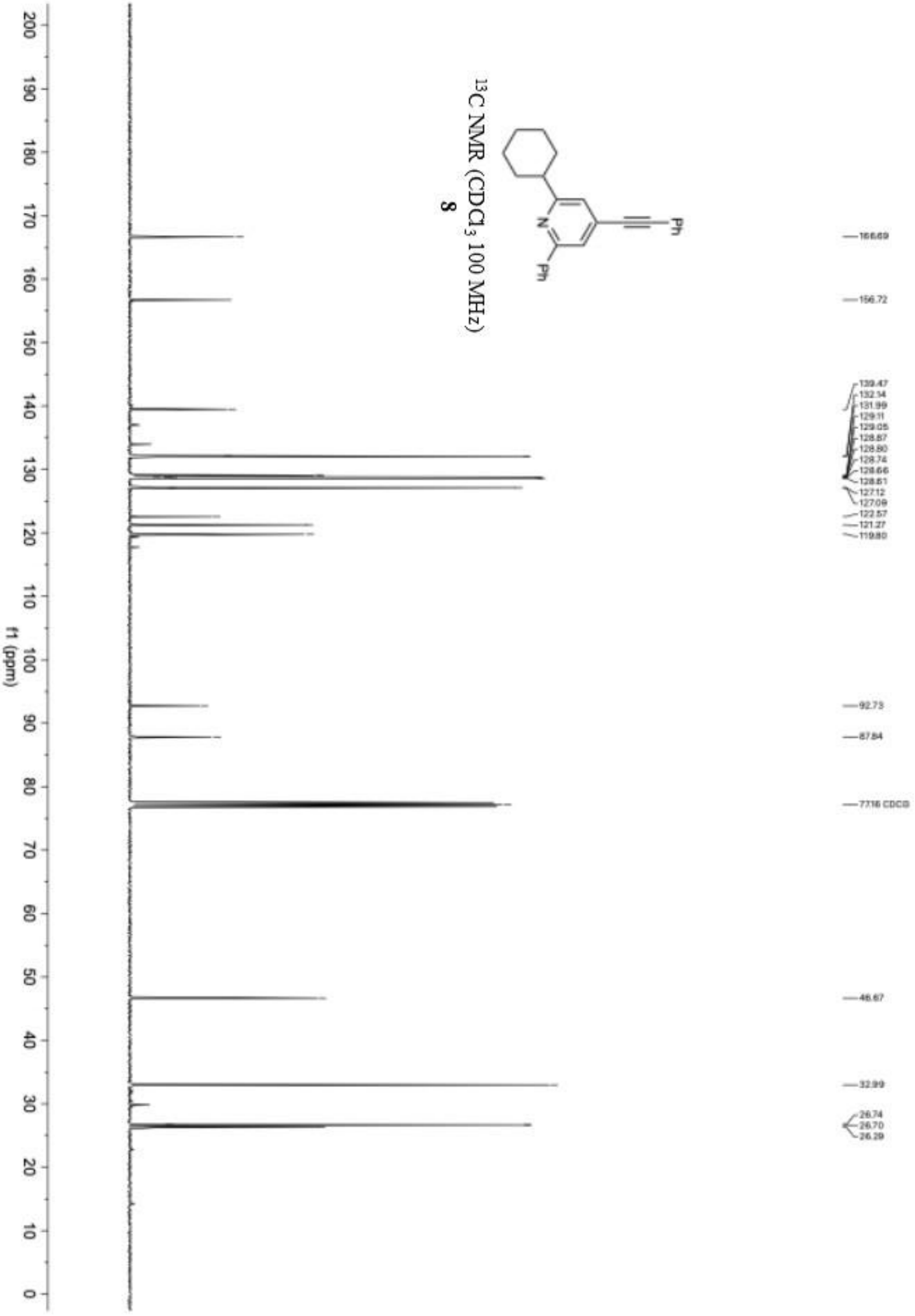

Ph
N
Ph
13C NMR (CDCl3 100 MHz)
8
166.69
156.72
139.47
132.14
131.99
129.11
129.05
128.87
128.80
128.74
128.66
128.61
127.12
127.09
122.57
121.27
119.80
92.73
87.84
77.16 CDCl3
46.67
32.99
26.74
26.70
26.29
f1 (ppm)

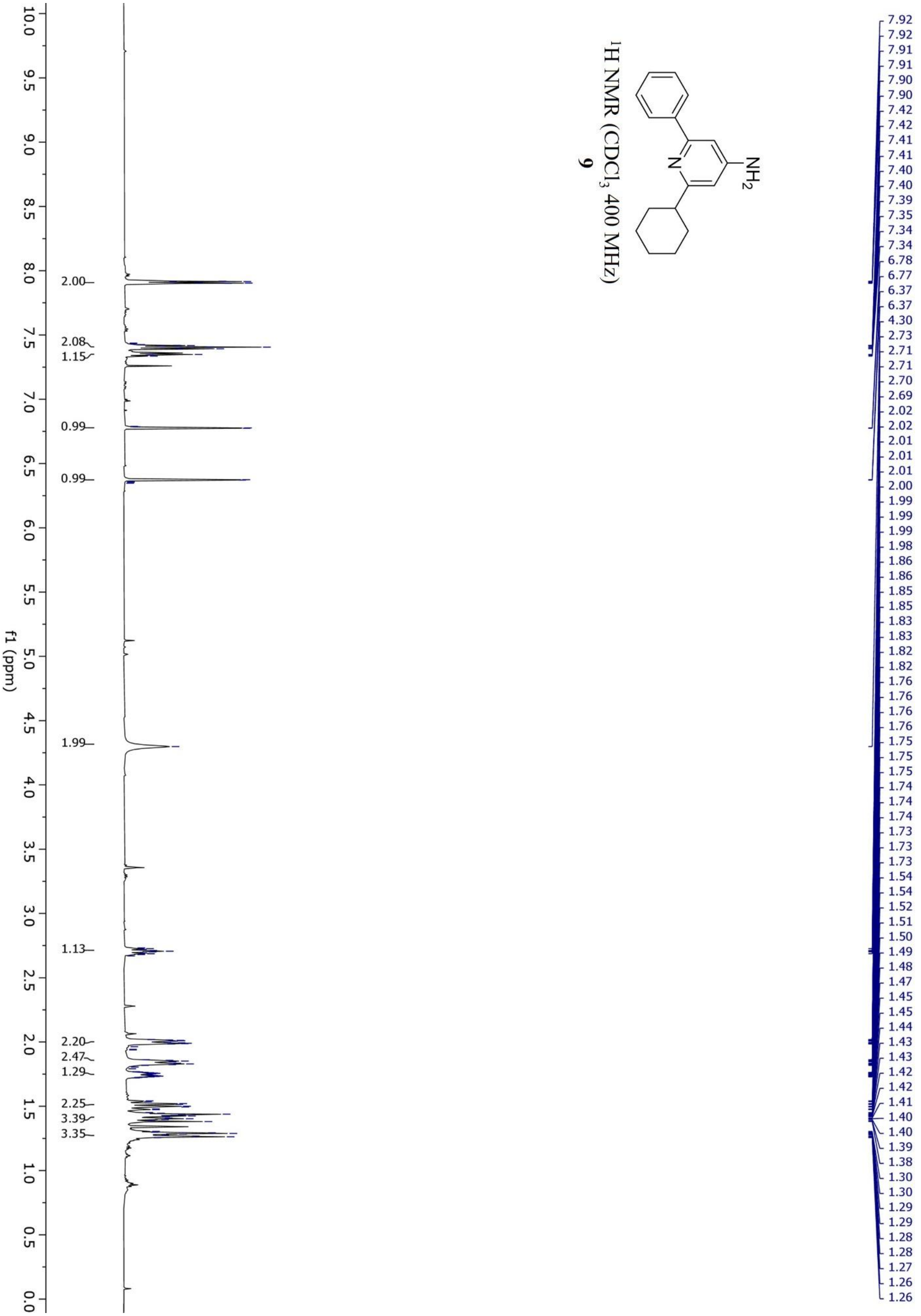

¹H NMR (CDCl3 400 MHz)
9
NH2
N
7.92
7.92
7.91
7.91
7.90
7.90
7.42
7.42
7.41
7.41
7.40
7.40
7.39
7.35
7.34
7.34
6.78
6.77
6.37
6.37
4.30
2.73
2.71
2.71
2.70
2.69
2.02
2.02
2.01
2.01
2.01
2.00
1.99
1.99
1.99
1.98
1.86
1.86
1.85
1.85
1.83
1.83
1.82
1.82
1.76
1.76
1.76
1.76
1.75
1.75
1.75
1.74
1.74
1.74
1.73
1.73
1.73
1.54
1.54
1.52
1.51
1.50
1.49
1.48
1.47
1.45
1.45
1.44
1.43
1.43
1.42
1.42
1.41
1.40
1.40
1.39
1.38
1.30
1.30
1.29
1.29
1.28
1.28
1.27
1.26
1.26
2.00
2.08
1.15
0.99
0.99
1.99
1.13
2.20
2.47
1.29
2.25
3.39
3.35
10.0
9.5
9.0
8.5
8.0
7.5
7.0
6.5
6.0
5.5
5.0
4.5
4.0
3.5
3.0
2.5
2.0
1.5
1.0
0.5
0.0
f1 (ppm)

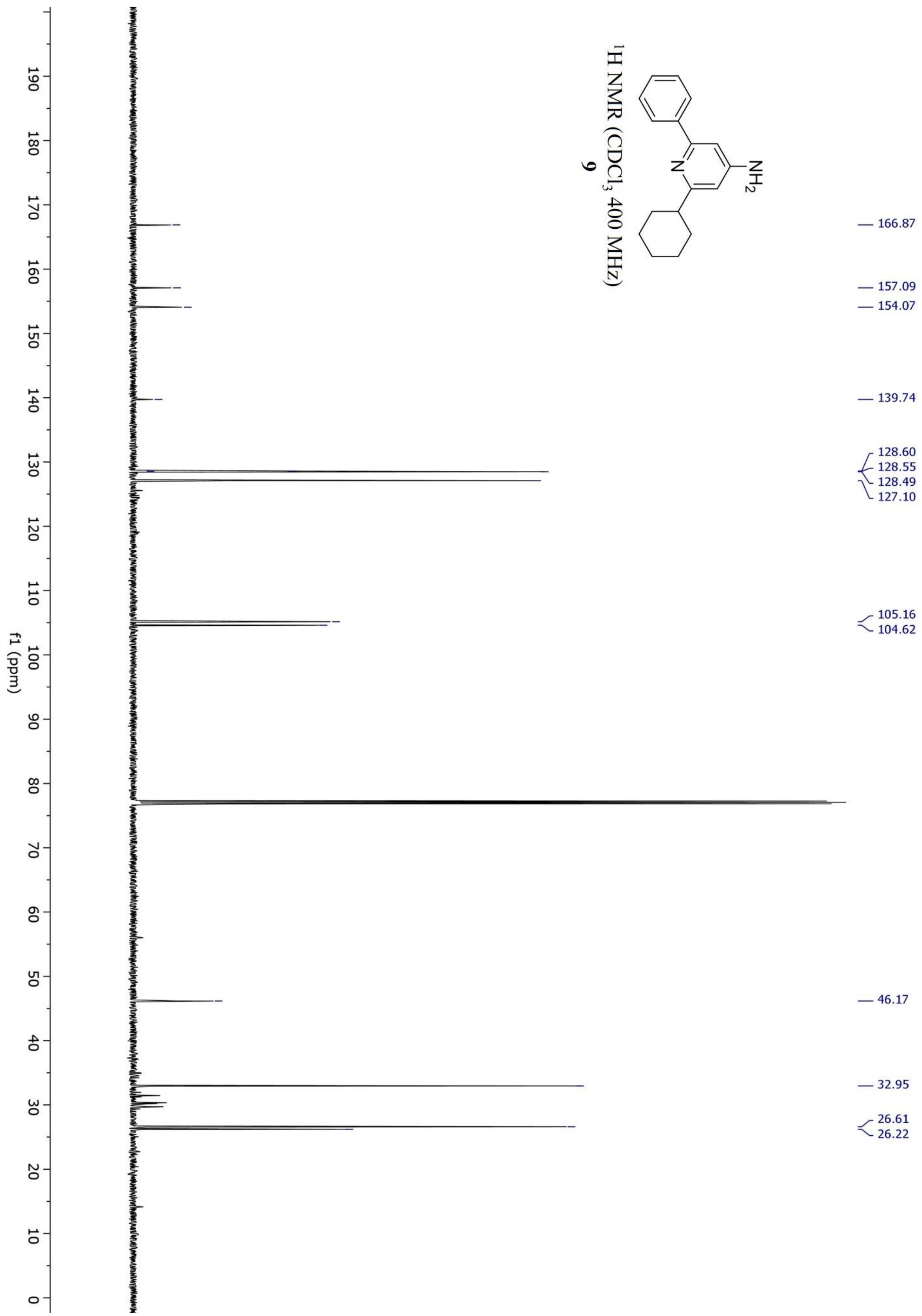

NH2
N
1H NMR (CDCl3 400 MHz)
9
166.87
157.09
154.07
139.74
128.60
128.55
128.49
127.10
105.16
104.62
46.17
32.95
26.61
26.22
190
180
170
160
150
140
130
120
110
100
90
80
70
60
50
40
30
20
10
0
f1 (ppm)

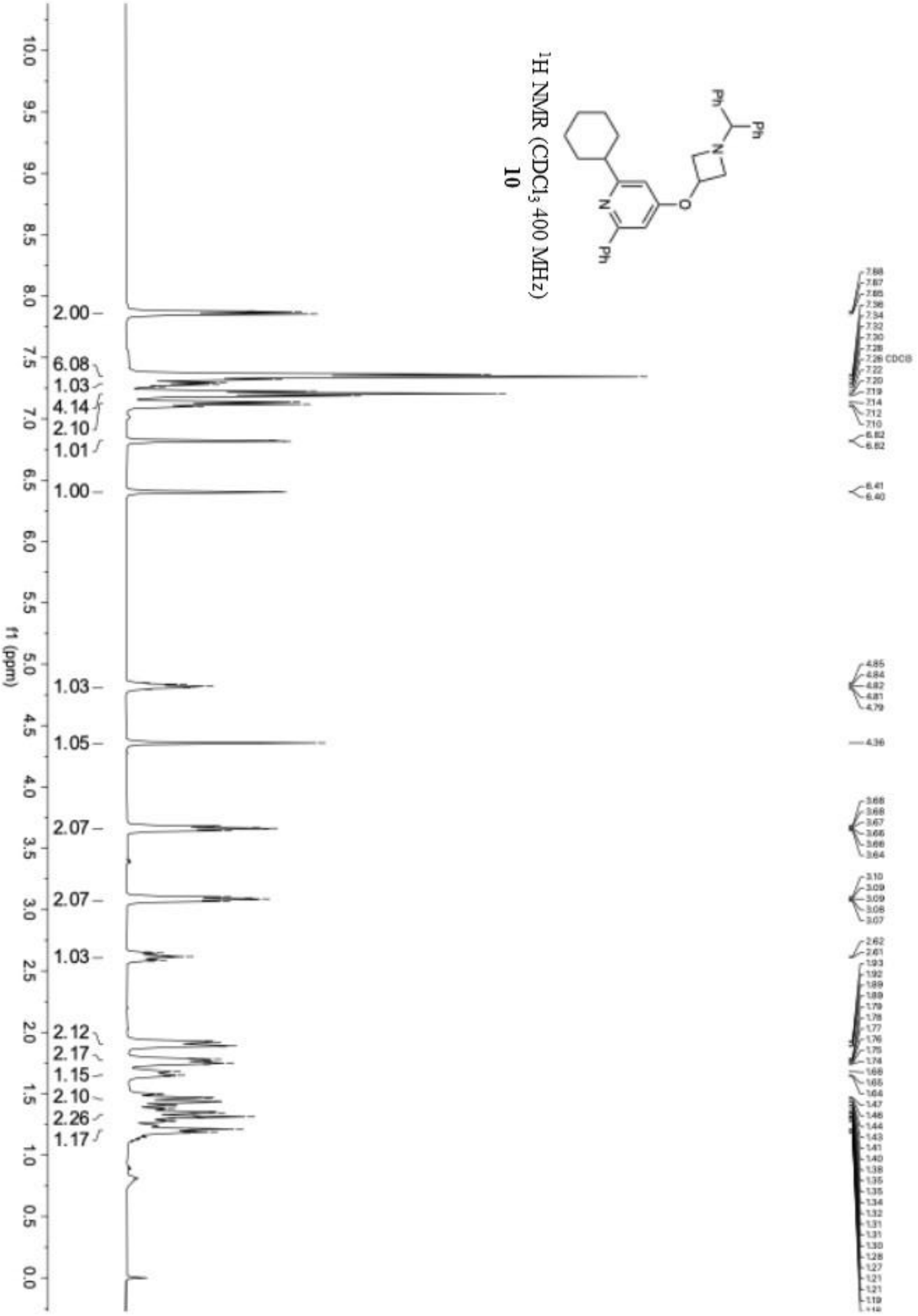

^{1}H NMR (CDCl$_3$ 400 MHz)
10
f1 (ppm)

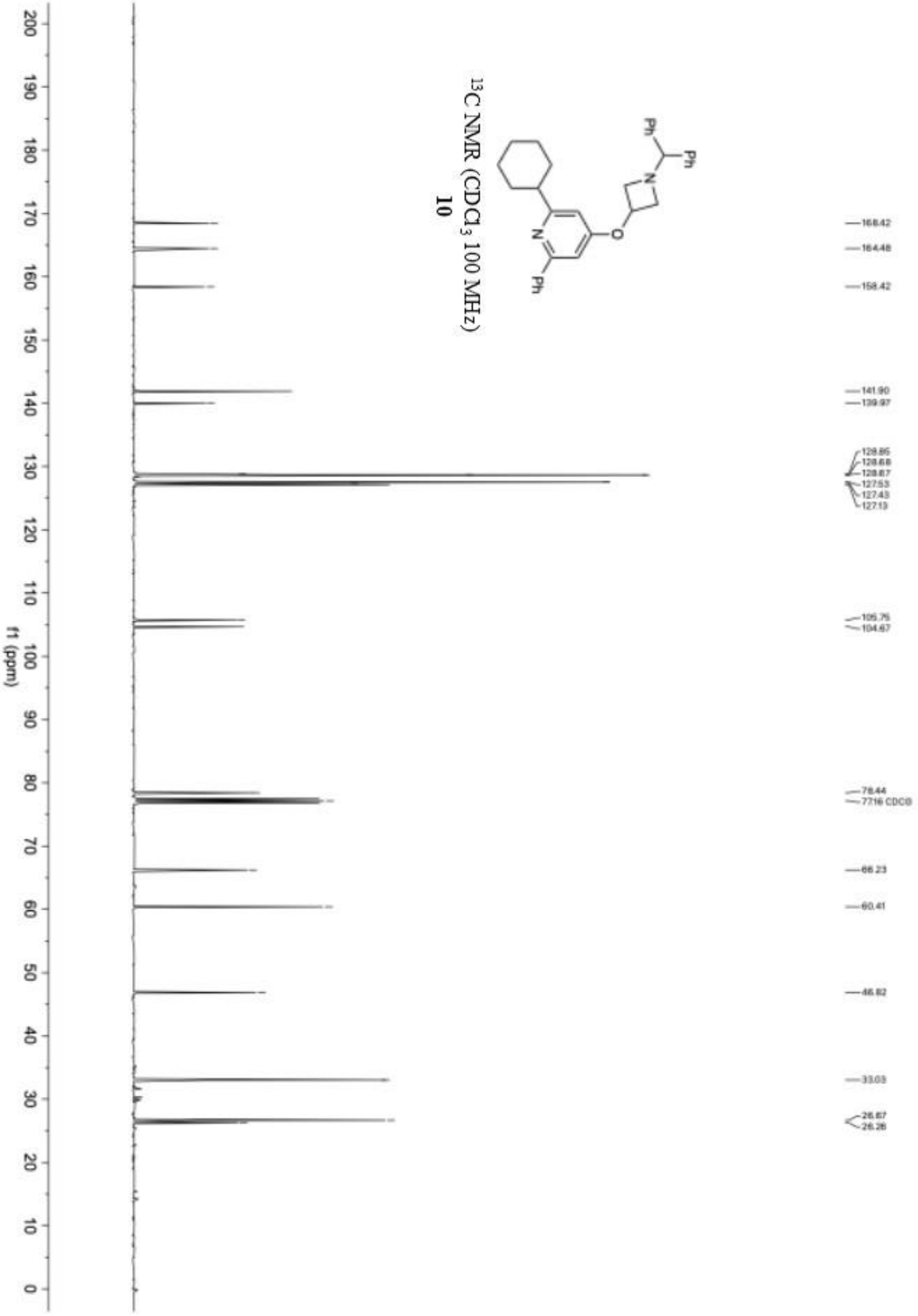
13C NMR (CDCl3 100 MHz)
10
Ph
Ph
N
O
N
Ph
168.42
164.48
158.42
141.90
139.97
128.85
128.68
128.67
127.53
127.43
127.13
105.75
104.67
78.44
77.16 CDCl3
66.23
60.41
46.82
33.03
26.67
26.26
f1 (ppm)
200
190
180
170
160
150
140
130
120
110
100
90
80
70
60
50
40
30
20
10
0

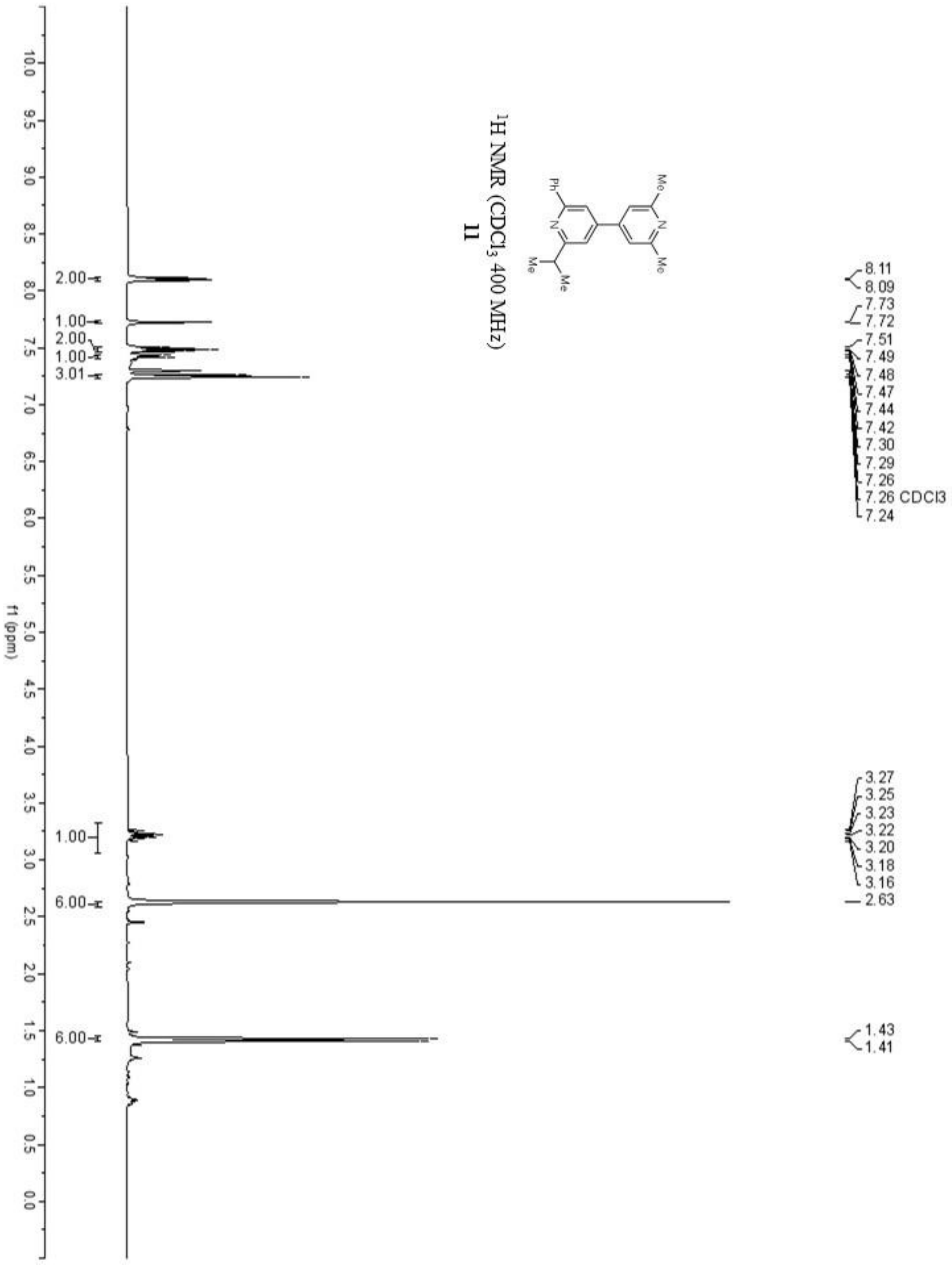
1H NMR (CDCl3 400 MHz)
II
Ph
N
Me
Me
Me
N
Me
8.11
8.09
7.73
7.72
7.51
7.49
7.48
7.47
7.44
7.42
7.30
7.29
7.26
7.26 CDCl3
7.24
3.27
3.25
3.23
3.22
3.20
3.18
3.16
2.63
1.43
1.41
2.00
1.00
2.00
1.00
3.01
1.00
6.00
6.00
f1 (ppm)
10.0
9.5
9.0
8.5
8.0
7.5
7.0
6.5
6.0
5.5
5.0
4.5
4.0
3.5
3.0
2.5
2.0
1.5
1.0
0.5
0.0

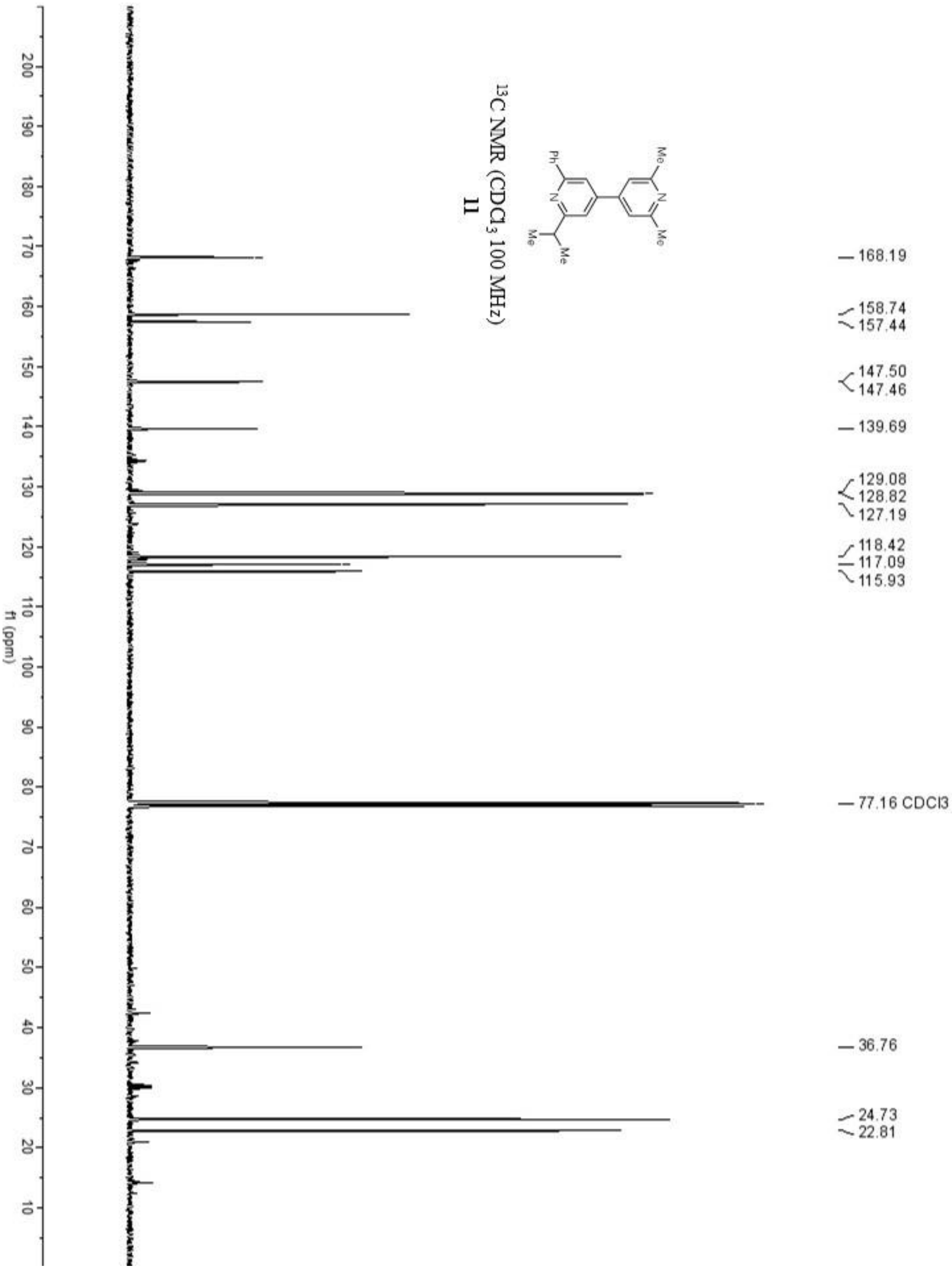

13C NMR (CDCl3 100 MHz)
11
168.19
158.74
157.44
147.50
147.46
139.69
129.08
128.82
127.19
118.42
117.09
115.93
77.16 CDCl3
36.76
24.73
22.81
f1 (ppm)

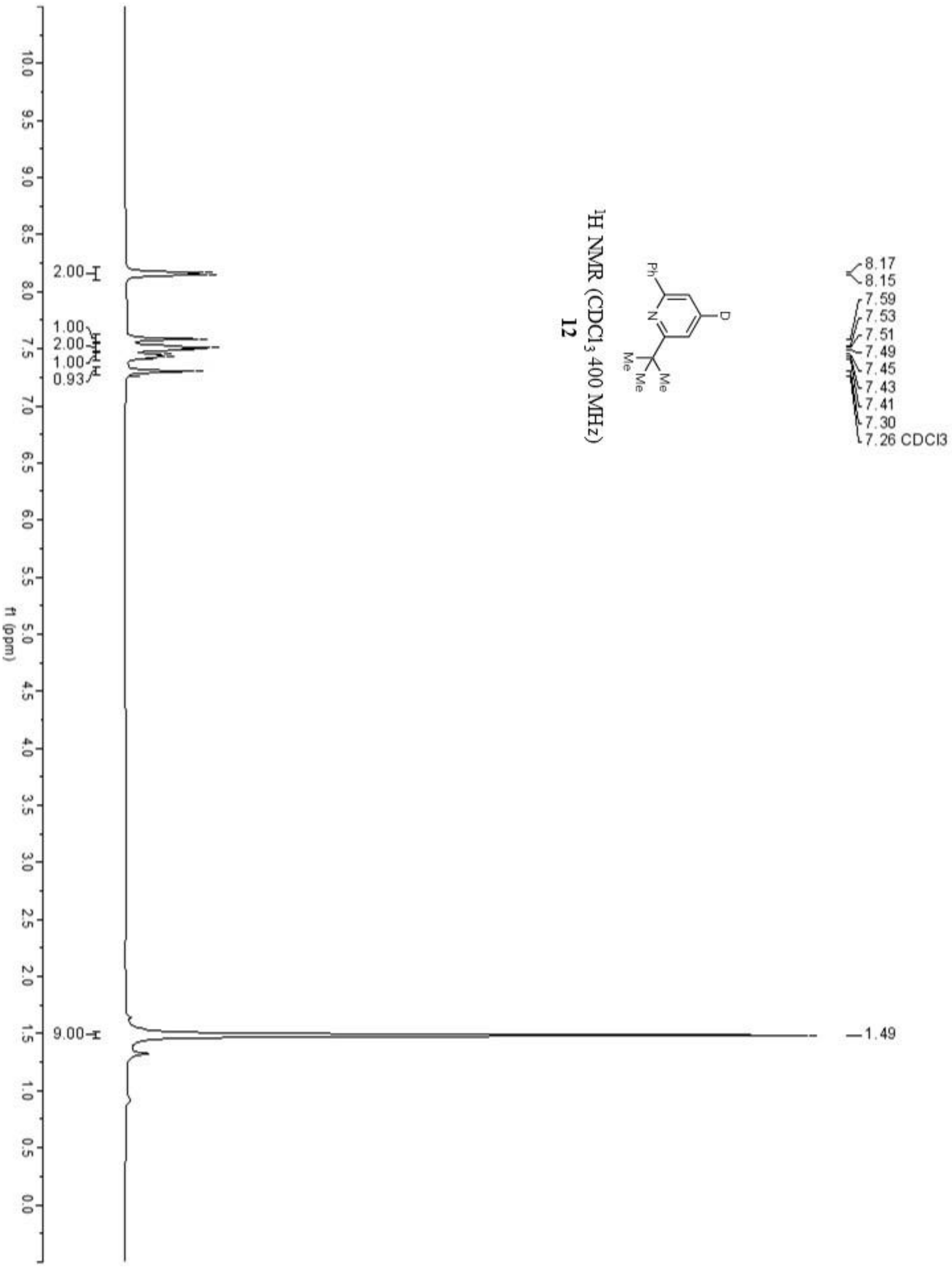

¹H NMR (CDCl3 400 MHz)
12
Ph
N
D
Me
Me
Me
8.17
8.15
7.59
7.53
7.51
7.49
7.45
7.43
7.41
7.30
7.26 CDCl3
1.49
2.00
1.00
2.00
1.00
0.93
9.00
f1 (ppm)
10.0
9.5
9.0
8.5
8.0
7.5
7.0
6.5
6.0
5.5
5.0
4.5
4.0
3.5
3.0
2.5
2.0
1.5
1.0
0.5
0.0

2 Computational Studies

Computational studies were carried out using the Rowan Scientific website using the "orbitals and electronic properties" and "Fukui Index" calculation workflows.[4] All analyses were performed on the lowest every conformer generated using CREST followed by geometry optimization using AIMNet2.[5,6] Fukui Index calculations were performed at the r²SCAN-3c/CPCM(Acetonitrile) level of theory following geometry reoptimization at the same level of theory.[7,8] The orbitals and electronic properties calculations were then performed at the r²SCAN-3c/COSMO(Acetonitrile) level of theory using these optimized structures.

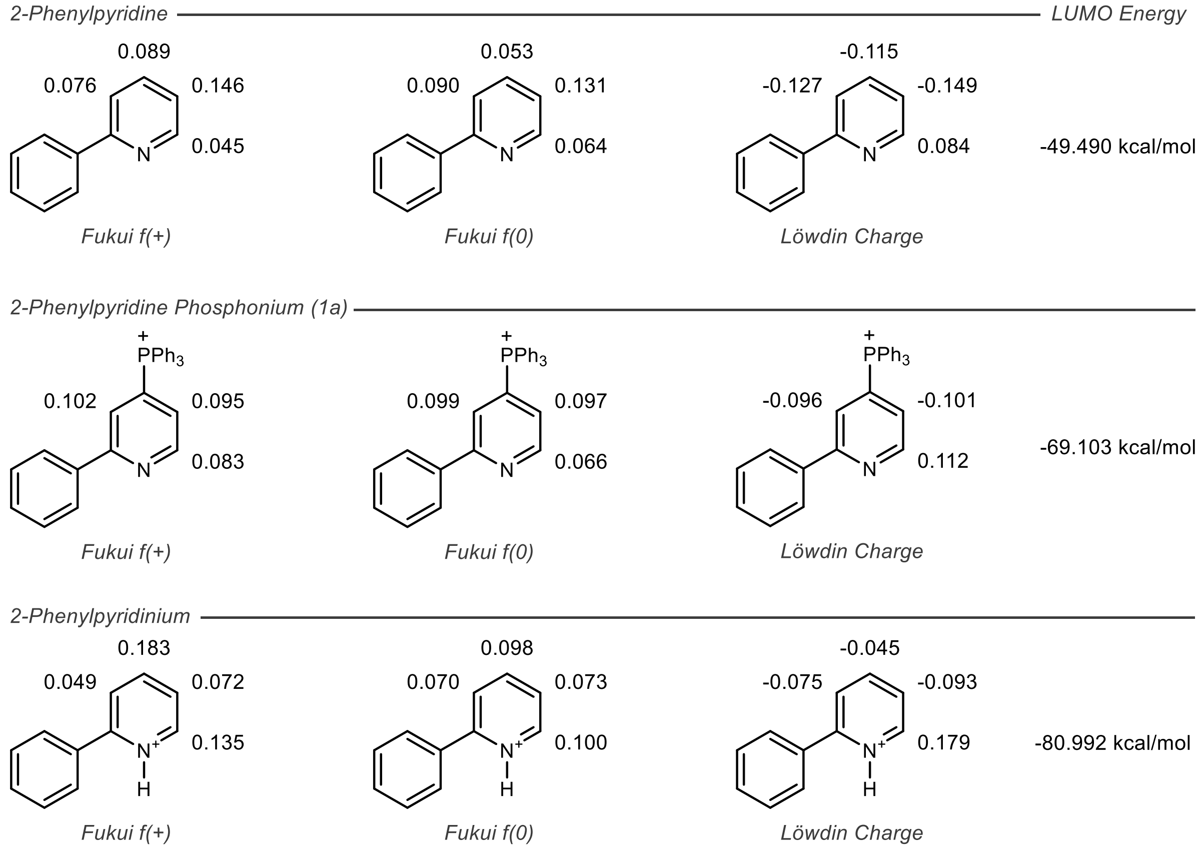

Figure S4: Fukui indices, *f*(+) and *f*(0), Löwdin charges, and LUMO energies for 2-phenylpyridine, phosphonium salt **1a**, and 2-phenylpyridinium

LUMO energy calculations suggest that pyridylphosphonium salt **1a** is significantly more electrophilic than 2-phenylpyridine, specifically a decrease in 19.6 kcal/mol is observed. In comparison, protonated pyridine, which is implicated in many Minisci reaction mechanisms, has a LUMO 31.5 kcal/mol lower than 2-phenylpyridine. Additionally, both the Fukui *f*(+) index and the Löwdin charge are increased at the pyridine 2-position when the phosphonium group is present.

The regioselectivity of this Minisci reaction is less well explained using the calculated descriptors. While both Fukui indices *f*(+) and *f*(0) are increased at the pyridine 2-position when the phosphonium is installed, there is a modest preference for reactivity is at the pyridine 3-position, which contrasts with the observed 2-position selectivity. This suggests that the phosphonium group serves to sterically inhibit radical addition to the 3-position. Therefore, a more in-depth analysis of the reaction mechanism is needed to confirm this hypothesis, as the selectivity determining step may occur after radical addition.

3 References

(1) Armarego, W. L. F.; Armarego, W. L. F.; Perrin, D. D. *Purification of Laboratory Chemicals*, 4. ed., reprint.; Butterworth-Heinemann: Oxford, 2002.

(2) *UVEX Amber safety glasses - HepatoChem*. https://hepatochem.com/product/hck1015-01-001/ (accessed 2025-05-07).

(3) *Amazon.com: SUPERNIGHT LED Strip Lights, RGB Color Changing 16.4Ft SMD5050 LEDs Flexible Light Strip Rope Lighting Kit with 44 Key Remote Controller and 12V Power Supply for Bedroom TV Backlight Christmas : Tools & Home Improvement*. https://www.amazon.com/SUPERNIGHT-5-Meter-Waterproof-FlexibleChanging/dp/B00ASHQQKI/ref=sr_1_6?crid=3YPKV403V6A2&keywords=supernight%%202Bled%2Blights&qid=1695225304&sprefix=supernight%2Bled%2Blights%2Caps%2C150&sr=8-6&th=1 (accessed 2025-05-07).

(4) *Rowan Scientific.* https://www.rowansci.com (accessed 2026-05-18).

(5) Pracht, P.; Bohle, F.; Grimme, S. Automated Exploration of the Low-Energy Chemical Space with Fast Quantum Chemical Methods. *Phys. Chem. Chem. Phys.* **2020**, *22* (14), 7169–7192. https://doi.org/10.1039/C9CP06869D.

(6) Anstine, D. M.; Zubatyuk, R.; Isayev, O. AIMNet2: A Neural Network Potential to Meet Your Neutral, Charged, Organic, and Elemental-Organic Needs. *Chem. Sci.* **2025**, *16* (23), 10228–10244. https://doi.org/10.1039/D4SC08572H.

(7) *Psi4: an open-source ab initio electronic structure program - Turney - 2012 - WIREs Computational Molecular Science - Wiley Online Library*. https://wires.onlinelibrary.wiley.com/doi/10.1002/wcms.93 (accessed 2026-05-18).

(8) Grimme, S.; Hansen, A.; Ehlert, S.; Mewes, J.-M. r2SCAN-3c: A “Swiss Army Knife” Composite Electronic-Structure Method. *J. Chem. Phys.* **2021**, *154* (6), 064103. https://doi.org/10.1063/5.0040021.

(9) Ravn, A. K.; Johansen, M. B.; Skrydstrup, T. Regioselective Hydroalkylation of Vinylarenes by Cooperative Cu and Ni Catalysis. *Angewandte Chemie International Edition* **2022**, *61* (4), e202112390. https://doi.org/10.1002/anie.202112390.

(10) Wang, X.; Meyer, M.; Yao, B.; Guo, T.; Wei, G. P.; Wang, L. Chemoking Receptor Antagonists, January 24, 2013. https://patentscope.wipo.int/search/en/WO2013010453 (accessed 2025-05-06).

(11) Boyle, B. T.; Hilton, M. C.; McNally, A. Nonsymmetrical Bis-Azine Biaryls from Chloroazines: A Strategy Using Phosphorus Ligand-Coupling. *J. Am. Chem. Soc.* **2019**, *141* (38), 15441–15449. https://doi.org/10.1021/jacs.9b08504.

(12) Zhang, X.; McNally, A. Phosphonium Salts as Pseudohalides: Regioselective Nickel-Catalyzed Cross-Coupling of Complex Pyridines and Diazines. *Angew. Chem. Int. Ed.* **2017**, *56* (33), 9833–9836. https://doi.org/10.1002/anie.201704948.

(13) Hilton, M. C.; Dolewski, R. D.; McNally, A. Selective Functionalization of Pyridines via Heterocyclic Phosphonium Salts. *J. Am. Chem. Soc.* **2016**, *138* (42), 13806–13809. https://doi.org/10.1021/jacs.6b08662.

(14) Zhang, X.; McNally, A. Cobalt-Catalyzed Alkylation of Drug-Like Molecules and Pharmaceuticals Using Heterocyclic Phosphonium Salts. *ACS Catal.* **2019**, *9* (6), 4862–4866. https://doi.org/10.1021/acscatal.9b00851.

(15) Koniarczyk, J. L.; Hesk, D.; Overgard, A.; Davies, I. W.; McNally, A. A General Strategy for Site-Selective Incorporation of Deuterium and Tritium into Pyridines, Diazines, and Pharmaceuticals. *J. Am. Chem. Soc.* **2018**, *140* (6), 1990–1993. https://doi.org/10.1021/jacs.7b11710.

(16) Wang, Q.-D.; Zhang, S.-X.; Zhang, Z.-W.; Wang, Y.; Ma, M.; Chu, X.-Q.; Shen, Z.-L. Palladium-Catalyzed Sonogashira Coupling of a Heterocyclic Phosphonium Salt with a Terminal Alkyne. *Org. Lett.* **2022**, *24* (27), 4919–4924. https://doi.org/10.1021/acs.orglett.2c01800.

(17) Patel, C.; Mohnike, M.; Hilton, M. C.; McNally, A. A Strategy to Aminate Pyridines, Diazines, and Pharmaceuticals via Heterocyclic Phosphonium Salts. *Org. Lett.* **2018**, *20* (9), 2607–2610. https://doi.org/10.1021/acs.orglett.8b00813.
